# Global Burden of Cardiovascular Diseases and Risk Factors, 1990–2019: Update From the GBD 2019 Study

**DOI:** 10.1016/j.jacc.2020.11.010

**Published:** 2020-12-22

**Authors:** Gregory A. Roth, George A. Mensah, Catherine O. Johnson, Giovanni Addolorato, Enrico Ammirati, Larry M. Baddour, Noël C. Barengo, Andrea Z. Beaton, Emelia J. Benjamin, Catherine P. Benziger, Aimé Bonny, Michael Brauer, Marianne Brodmann, Thomas J. Cahill, Jonathan Carapetis, Alberico L. Catapano, Sumeet S. Chugh, Leslie T. Cooper, Josef Coresh, Michael Criqui, Nicole DeCleene, Kim A. Eagle, Sophia Emmons-Bell, Valery L. Feigin, Joaquim Fernández-Solà, Gerry Fowkes, Emmanuela Gakidou, Scott M. Grundy, Feng J. He, George Howard, Frank Hu, Lesley Inker, Ganesan Karthikeyan, Nicholas Kassebaum, Walter Koroshetz, Carl Lavie, Donald Lloyd-Jones, Hong S. Lu, Antonio Mirijello, Awoke Misganaw Temesgen, Ali Mokdad, Andrew E. Moran, Paul Muntner, Jagat Narula, Bruce Neal, Mpiko Ntsekhe, Glaucia Moraes de Oliveira, Catherine Otto, Mayowa Owolabi, Michael Pratt, Sanjay Rajagopalan, Marissa Reitsma, Antonio Luiz P. Ribeiro, Nancy Rigotti, Anthony Rodgers, Craig Sable, Saate Shakil, Karen Sliwa-Hahnle, Benjamin Stark, Johan Sundström, Patrick Timpel, Imad M. Tleyjeh, Marco Valgimigli, Theo Vos, Paul K. Whelton, Magdi Yacoub, Liesl Zuhlke, Christopher Murray, Valentin Fuster, GBD-NHLBI-JACC Global Burden of Cardiovascular Diseases Writing Group, Gregory A. Roth, George A. Mensah, Catherine O. Johnson, Giovanni Addolorato, Enrico Ammirati, Larry M. Baddour, Noel C. Barengo, Andrea Beaton, Emelia J. Benjamin, Catherine P. Benziger, Aime Bonny, Michael Brauer, Marianne Brodmann, Thomas J. Cahill, Jonathan R. Carapetis, Alberico L. Catapano, Sumeet Chugh, Leslie T. Cooper, Josef Coresh, Michael H. Criqui, Nicole K. DeCleene, Kim A. Eagle, Sophia Emmons-Bell, Valery L. Feigin, Joaquim Fernández-Sola, F. Gerry R. Fowkes, Emmanuela Gakidou, Scott M. Grundy, Feng J. He, George Howard, Frank Hu, Lesley Inker, Ganesan Karthikeyan, Nicholas J. Kassebaum, Walter J. Koroshetz, Carl Lavie, Donald Lloyd-Jones, Hong S. Lu, Antonio Mirijello, Awoke T. Misganaw, Ali H. Mokdad, Andrew E. Moran, Paul Muntner, Jagat Narula, Bruce Neal, Mpiko Ntsekhe, Gláucia M.M. Oliveira, Catherine M. Otto, Mayowa O. Owolabi, Michael Pratt, Sanjay Rajagopalan, Marissa B. Reitsma, Antonio Luiz P. Ribeiro, Nancy A. Rigotti, Anthony Rodgers, Craig A. Sable, Saate S. Shakil, Karen Sliwa, Benjamin A. Stark, Johan Sundström, Patrick Timpel, Imad I. Tleyjeh, Marco Valgimigli, Theo Vos, Paul K. Whelton, Magdi Yacoub, Liesl J. Zuhlke, Mohsen Abbasi-Kangevari, Alireza Abdi, Aidin Abedi, Victor Aboyans, Woldu A. Abrha, Eman Abu-Gharbieh, Abdelrahman I. Abushouk, Dilaram Acharya, Tim Adair, Oladimeji M. Adebayo, Zanfina Ademi, Shailesh M. Advani, Khashayar Afshari, Ashkan Afshin, Gina Agarwal, Pradyumna Agasthi, Sohail Ahmad, Sepideh Ahmadi, Muktar B. Ahmed, Budi Aji, Yonas Akalu, Wuraola Akande-Sholabi, Addis Aklilu, Chisom J. Akunna, Fares Alahdab, Ayman Al-Eyadhy, Khalid F. Alhabib, Sheikh M. Alif, Vahid Alipour, Syed M. Aljunid, François Alla, Amir Almasi-Hashiani, Sami Almustanyir, Rajaa M. Al-Raddadi, Adeladza K. Amegah, Saeed Amini, Arya Aminorroaya, Hubert Amu, Dickson A. Amugsi, Robert Ancuceanu, Deanna Anderlini, Tudorel Andrei, Catalina Liliana Andrei, Alireza Ansari-Moghaddam, Zelalem A. Anteneh, Ippazio Cosimo Antonazzo, Benny Antony, Razique Anwer, Lambert T. Appiah, Jalal Arabloo, Johan Ärnlöv, Kurnia D. Artanti, Zerihun Ataro, Marcel Ausloos, Leticia Avila-Burgos, Asma T. Awan, Mamaru A. Awoke, Henok T. Ayele, Muluken A. Ayza, Samad Azari, Darshan B. B, Nafiseh Baheiraei, Atif A. Baig, Ahad Bakhtiari, Maciej Banach, Palash C. Banik, Emerson A. Baptista, Miguel A. Barboza, Lingkan Barua, Sanjay Basu, Neeraj Bedi, Yannick Béjot, Derrick A. Bennett, Isabela M. Bensenor, Adam E. Berman, Yihienew M. Bezabih, Akshaya S. Bhagavathula, Sonu Bhaskar, Krittika Bhattacharyya, Ali Bijani, Boris Bikbov, Mulugeta M. Birhanu, Archith Boloor, Luisa C. Brant, Hermann Brenner, Nikolay I. Briko, Zahid A. Butt, Florentino Luciano Caetano dos Santos, Leah E. Cahill, Lucero Cahuana-Hurtado, Luis A. Cámera, Ismael R. Campos-Nonato, Carlos Cantu-Brito, Josip Car, Juan J. Carrero, Felix Carvalho, Carlos A. Castañeda-Orjuela, Ferrán Catalá-López, Ester Cerin, Jaykaran Charan, Vijay Kumar Chattu, Simiao Chen, Ken L. Chin, Jee-Young J. Choi, Dinh-Toi Chu, Sheng-Chia Chung, Massimo Cirillo, Sean Coffey, Sara Conti, Vera M. Costa, David K. Cundiff, Omid Dadras, Baye Dagnew, Xiaochen Dai, Albertino A.M. Damasceno, Lalit Dandona, Rakhi Dandona, Kairat Davletov, Vanessa De la Cruz-Góngora, Fernando P. De la Hoz, Jan-Walter De Neve, Edgar Denova-Gutiérrez, Meseret Derbew Molla, Behailu T. Derseh, Rupak Desai, Günther Deuschl, Samath D. Dharmaratne, Meghnath Dhimal, Raja Ram Dhungana, Mostafa Dianatinasab, Daniel Diaz, Shirin Djalalinia, Klara Dokova, Abdel Douiri, Bruce B. Duncan, Andre R. Duraes, Arielle W. Eagan, Sanam Ebtehaj, Aziz Eftekhari, Sahar Eftekharzadeh, Michael Ekholuenetale, Nevine El Nahas, Islam Y. Elgendy, Muhammed Elhadi, Shaimaa I. El-Jaafary, Sadaf Esteghamati, Atkilt E. Etisso, Oghenowede Eyawo, Ibtihal Fadhil, Emerito Jose A. Faraon, Pawan S. Faris, Medhat Farwati, Farshad Farzadfar, Eduarda Fernandes, Carlota Fernandez Prendes, Pietro Ferrara, Irina Filip, Florian Fischer, David Flood, Takeshi Fukumoto, Mohamed M. Gad, Shilpa Gaidhane, Morsaleh Ganji, Jalaj Garg, Abadi K. Gebre, Birhan G. Gebregiorgis, Kidane Z. Gebregzabiher, Gebreamlak G. Gebremeskel, Lemma Getacher, Abera Getachew Obsa, Alireza Ghajar, Ahmad Ghashghaee, Nermin Ghith, Simona Giampaoli, Syed Amir Gilani, Paramjit S. Gill, Richard F. Gillum, Ekaterina V. Glushkova, Elena V. Gnedovskaya, Mahaveer Golechha, Kebebe B. Gonfa, Amir Hossein Goudarzian, Alessandra C. Goulart, Jenny S. Guadamuz, Avirup Guha, Yuming Guo, Rajeev Gupta, Vladimir Hachinski, Nima Hafezi-Nejad, Teklehaimanot G. Haile, Randah R. Hamadeh, Samer Hamidi, Graeme J. Hankey, Arief Hargono, Risky K. Hartono, Maryam Hashemian, Abdiwahab Hashi, Shoaib Hassan, Hamid Y. Hassen, Rasmus J. Havmoeller, Simon I. Hay, Khezar Hayat, Golnaz Heidari, Claudiu Herteliu, Ramesh Holla, Mostafa Hosseini, Mehdi Hosseinzadeh, Mihaela Hostiuc, Sorin Hostiuc, Mowafa Househ, Junjie Huang, Ayesha Humayun, Ivo Iavicoli, Charles U. Ibeneme, Segun E. Ibitoye, Olayinka S. Ilesanmi, Irena M. Ilic, Milena D. Ilic, Usman Iqbal, Seyed Sina N. Irvani, Sheikh Mohammed Shariful Islam, Rakibul M. Islam, Hiroyasu Iso, Masao Iwagami, Vardhmaan Jain, Tahereh Javaheri, Sathish Kumar Jayapal, Shubha Jayaram, Ranil Jayawardena, Panniyammakal Jeemon, Ravi P. Jha, Jost B. Jonas, Jitendra Jonnagaddala, Farahnaz Joukar, Jacek J. Jozwiak, Mikk Jürisson, Ali Kabir, Tanvir Kahlon, Rizwan Kalani, Rohollah Kalhor, Ashwin Kamath, Ibrahim Kamel, Himal Kandel, Amit Kandel, André Karch, Ayele Semachew Kasa, Patrick D.M.C. Katoto, Gbenga A. Kayode, Yousef S. Khader, Mohammad Khammarnia, Muhammad S. Khan, Md Nuruzzaman Khan, Maseer Khan, Ejaz A. Khan, Khaled Khatab, Gulam M.A. Kibria, Yun Jin Kim, Gyu Ri Kim, Ruth W. Kimokoti, Sezer Kisa, Adnan Kisa, Mika Kivimäki, Dhaval Kolte, Ali Koolivand, Vladimir A. Korshunov, Sindhura Lakshmi Koulmane Laxminarayana, Ai Koyanagi, Kewal Krishan, Vijay Krishnamoorthy, Barthelemy Kuate Defo, Burcu Kucuk Bicer, Vaman Kulkarni, G. Anil Kumar, Nithin Kumar, Om P. Kurmi, Dian Kusuma, Gene F. Kwan, Carlo La Vecchia, Ben Lacey, Tea Lallukka, Qing Lan, Savita Lasrado, Zohra S. Lassi, Paolo Lauriola, Wayne R. Lawrence, Avula Laxmaiah, Kate E. LeGrand, Ming-Chieh Li, Bingyu Li, Shanshan Li, Stephen S. Lim, Lee-Ling Lim, Hualiang Lin, Ziqiang Lin, Ro-Ting Lin, Xuefeng Liu, Alan D. Lopez, Stefan Lorkowski, Paulo A. Lotufo, Alessandra Lugo, Nirmal K. M, Fabiana Madotto, Morteza Mahmoudi, Azeem Majeed, Reza Malekzadeh, Ahmad A. Malik, Abdullah A. Mamun, Navid Manafi, Mohammad Ali Mansournia, Lorenzo G. Mantovani, Santi Martini, Manu R. Mathur, Giampiero Mazzaglia, Suresh Mehata, Man Mohan Mehndiratta, Toni Meier, Ritesh G. Menezes, Atte Meretoja, Tomislav Mestrovic, Bartosz Miazgowski, Tomasz Miazgowski, Irmina Maria Michalek, Ted R. Miller, Erkin M. Mirrakhimov, Hamed Mirzaei, Babak Moazen, Masoud Moghadaszadeh, Yousef Mohammad, Dara K. Mohammad, Shafiu Mohammed, Mohammed A. Mohammed, Yaser Mokhayeri, Mariam Molokhia, Ahmed A. Montasir, Ghobad Moradi, Rahmatollah Moradzadeh, Paula Moraga, Lidia Morawska, Ilais Moreno Velásquez, Jakub Morze, Sumaira Mubarik, Walter Muruet, Kamarul Imran Musa, Ahamarshan J. Nagarajan, Mahdi Nalini, Vinay Nangia, Atta Abbas Naqvi, Sreenivas Narasimha Swamy, Bruno R. Nascimento, Vinod C. Nayak, Javad Nazari, Milad Nazarzadeh, Ruxandra I. Negoi, Sandhya Neupane Kandel, Huong L.T. Nguyen, Molly R. Nixon, Bo Norrving, Jean Jacques Noubiap, Brice E. Nouthe, Christoph Nowak, Oluwakemi O. Odukoya, Felix A. Ogbo, Andrew T. Olagunju, Hans Orru, Alberto Ortiz, Samuel M. Ostroff, Jagadish Rao Padubidri, Raffaele Palladino, Adrian Pana, Songhomitra Panda-Jonas, Utsav Parekh, Eun-Cheol Park, Mojtaba Parvizi, Fatemeh Pashazadeh Kan, Urvish K. Patel, Mona Pathak, Rajan Paudel, Veincent Christian F. Pepito, Arokiasamy Perianayagam, Norberto Perico, Hai Q. Pham, Thomas Pilgrim, Michael A. Piradov, Farhad Pishgar, Vivek Podder, Roman V. Polibin, Akram Pourshams, Dimas R.A. Pribadi, Navid Rabiee, Mohammad Rabiee, Amir Radfar, Alireza Rafiei, Fakher Rahim, Vafa Rahimi-Movaghar, Mohammad Hifz Ur Rahman, Muhammad Aziz Rahman, Amir Masoud Rahmani, Ivo Rakovac, Pradhum Ram, Sudha Ramalingam, Juwel Rana, Priyanga Ranasinghe, Sowmya J. Rao, Priya Rathi, Lal Rawal, Wasiq F. Rawasia, Reza Rawassizadeh, Giuseppe Remuzzi, Andre M.N. Renzaho, Aziz Rezapour, Seyed Mohammad Riahi, Ross L. Roberts-Thomson, Leonardo Roever, Peter Rohloff, Michele Romoli, Gholamreza Roshandel, Godfrey M. Rwegerera, Seyedmohammad Saadatagah, Maha M. Saber-Ayad, Siamak Sabour, Simona Sacco, Masoumeh Sadeghi, Sahar Saeedi Moghaddam, Saeed Safari, Amirhossein Sahebkar, Sana Salehi, Hamideh Salimzadeh, Mehrnoosh Samaei, Abdallah M. Samy, Itamar S. Santos, Milena M. Santric-Milicevic, Nizal Sarrafzadegan, Arash Sarveazad, Thirunavukkarasu Sathish, Monika Sawhney, Mete Saylan, Maria I. Schmidt, Aletta E. Schutte, Subramanian Senthilkumaran, Sadaf G. Sepanlou, Feng Sha, Saeed Shahabi, Izza Shahid, Masood A. Shaikh, Mahdi Shamali, Morteza Shamsizadeh, Md Shajedur Rahman Shawon, Aziz Sheikh, Mika Shigematsu, Min-Jeong Shin, Jae Il Shin, Rahman Shiri, Ivy Shiue, Kerem Shuval, Soraya Siabani, Tariq J. Siddiqi, Diego A.S. Silva, Jasvinder A. Singh, Ambrish Singh Mtech, Valentin Y. Skryabin, Anna A. Skryabina, Amin Soheili, Emma E. Spurlock, Leo Stockfelt, Stefan Stortecky, Saverio Stranges, Rizwan Suliankatchi Abdulkader, Hooman Tadbiri, Eyayou G. Tadesse, Degena B. Tadesse, Masih Tajdini, Md Tariqujjaman, Berhane F. Teklehaimanot, Mohamad-Hani Temsah, Ayenew K. Tesema, Bhaskar Thakur, Kavumpurathu R. Thankappan, Rekha Thapar, Amanda G. Thrift, Binod Timalsina, Marcello Tonelli, Mathilde Touvier, Marcos R. Tovani-Palone, Avnish Tripathi, Jaya P. Tripathy, Thomas C. Truelsen, Guesh M. Tsegay, Gebiyaw W. Tsegaye, Nikolaos Tsilimparis, Biruk S. Tusa, Stefanos Tyrovolas, Krishna Kishore Umapathi, Brigid Unim, Bhaskaran Unnikrishnan, Muhammad S. Usman, Muthiah Vaduganathan, Pascual R. Valdez, Tommi J. Vasankari, Diana Z. Velazquez, Narayanaswamy Venketasubramanian, Giang T. Vu, Isidora S. Vujcic, Yasir Waheed, Yanzhong Wang, Fang Wang, Jingkai Wei, Robert G. Weintraub, Abrha H. Weldemariam, Ronny Westerman, Andrea S. Winkler, Charles S. Wiysonge, Charles D.A. Wolfe, Befikadu Legesse Wubishet, Gelin Xu, Ali Yadollahpour, Kazumasa Yamagishi, Lijing L. Yan, Srikanth Yandrapalli, Yuichiro Yano, Hiroshi Yatsuya, Tomas Y. Yeheyis, Yigizie Yeshaw, Christopher S. Yilgwan, Naohiro Yonemoto, Chuanhua Yu, Hasan Yusefzadeh, Geevar Zachariah, Sojib Bin Zaman, Muhammed S. Zaman, Maryam Zamanian, Ramin Zand, Alireza Zandifar, Afshin Zarghi, Mikhail S. Zastrozhin, Anasthasia Zastrozhina, Zhi-Jiang Zhang, Yunquan Zhang, Wangjian Zhang, Chenwen Zhong, Zhiyong Zou, Yves Miel H. Zuniga, Christopher J.L. Murray, Valentin Fuster

**Affiliations:** a University of Washington, Seattle, Washington, USA; b National Heart, Lung, and Blood Institute (NHLBI), Bethesda, Maryland, USA; c University of Washington, Institute for Health Metrics and Evaluation, Seattle, Washington, USA; d Catholic University of Rome, Rome, Italy; e De Gasperis Cardio Center and Transplant Center, Niguarda Hospital, Milan, Italy; f Mayo Clinic, Rochester, Minnesota, USA; g Herbert Wertheim College of Medicine, Florida International University, Miami, Florida, USA; h Cincinnati Children’s Hospital, Cincinnati, Ohio, USA; i Boston University School of Public Health, Boston, Massachusetts, USA; j Essentia Health, Duluth, Minnesota, USA; k District Hospital of Bonassama-University of Douala, Douala, Cameroon; l University of British Columbia, Vancouver, British Columbia, Canada; m Medical University of Graz, Graz, Austria; n Newpath Partners LLC, Boston, Massachusetts, USA; o Telethon Kids Institute, Nedlands, Western Australia, Australia; p University of Milano, Milan, Italy; q Cedars-Sinai, Smidt Heart Institute, Los Angeles, California, USA; r Mayo Clinic, Jacksonville, Florida, USA; s Johns Hopkins Bloomberg School of Public Health, Baltimore, Maryland, USA; t University of California at San Diego, San Diego, California, USA; u The University of Michigan Samuel and Jean Frankel Cardiovascular Center, Ann Arbor, Michigan, USA; v Barnaclinic+ Grup Hospital Clinic, Barcelona, Spain; w University of Edinburgh, Edinburgh, United Kingdom; x University of Texas Southwestern Medical Center, Dallas, Texas, USA; y Queen Mary University of London, London, United Kingdom; z University of Alabama at Birmingham School of Public Health, Birmingham, Alabama, USA; aa Harvard Medical School, Boston, Massachusetts, USA; bb Tufts Medical Center, Boston, Massachusetts, USA; cc Cardiothoracic Sciences Centre, All India Institute of Medical Sciences, New Delhi, India; dd National Institute of Neurological Disorders and Stroke, Bethesda, Maryland, USA; ee Ochsner Health, New Orleans, Louisiana, USA; ff Northwestern University Feinberg School of Medicine, Chicago, Illinois, USA; gg University of Kentucky College of Medicine, Lexington, Kentucky, USA; hh IRCCS Casa Sollievo della Sofferenza Hospital, Department of Medical Sciences, San Giovanni Rotondo, Italy; ii Columbia University Irving Medical Center, New York, New York, USA; jj Icahn School of Medicine at Mount Sinai, New York, New York, USA; kk The University of Sydney School of Medicine, Sydney, New South Wales, Australia; ll University of Cape Town, Cape Town, South Africa; mm Universidade Federal do Rio de Janeiro, Rio de Janeiro, Brazil; nn University of Ibadan, Ibadan, Oyo State, Nigeria; oo Case Western Reserve University School of Medicine, Cleveland, Ohio, USA; pp Stanford University School of Medicine, Stanford, California, USA; qq Universidade Federal de Minas Gerais, Minas Gerais, Brazil; rr Massachusetts General Hospital, Boston, Massachusetts, USA; ss The George Institute for Global Health, Newtown, New South Wales, Australia; tt Imperial College of London, London, United Kingdom; uu Children’s National Hospital, Washington, DC, USA; vv Uppsala University, Uppsala, Sweden; ww Dresden University of Technology, Dresden, Germany; xx King Fahd Medical City, Riyadh, Saudi Arabia; yy Inselspital, University Hospital Bern, Bern, Switzerland; zz Tulane University School of Public Health and Tropical Medicine, New Orleans, Louisiana, USA; aaa Centro Nacional de Investigaciones Cardiovasculares, Madrid, Spain

**Keywords:** cardiovascular diseases, global health, health policy, population health, AC, alcoholic cardiomyopathy, AF, atrial fibrillation, AFL, atrial flutter, BMI, body mass index, CAVD, calcific aortic valve disease, CHA, congenital heart anomalies, CKD, chronic kidney disease, CVD, cardiovascular disease, DALYs, disability-adjusted life years, GBD, Global Burden of Diseases, Injuries, and Risk Factors Study, HAP, household air pollution, HHD, hypertensive heart disease, HICs, high-income countries, ICD, International Classification of Diseases, IHD, ischemic heart disease, IKF, impaired kidney function, IS, ischemic stroke, LDL, low-density lipoprotein, LMICs, low- and middle-income countries, LPA, low physical activity, MV, mitral valve, PAD, peripheral artery disease, PM, particulate matter, RHD, rheumatic heart disease, SBP, systolic blood pressure, SDI, sociodemographic index, TMREL, theoretical minimum risk exposure level, UI, uncertainty interval, YLDs, years lived with disability, YLLs, years of life lost

## Abstract

Cardiovascular diseases (CVDs), principally ischemic heart disease (IHD) and stroke, are the leading cause of global mortality and a major contributor to disability. This paper reviews the magnitude of total CVD burden, including 13 underlying causes of cardiovascular death and 9 related risk factors, using estimates from the Global Burden of Disease (GBD) Study 2019. GBD, an ongoing multinational collaboration to provide comparable and consistent estimates of population health over time, used all available population-level data sources on incidence, prevalence, case fatality, mortality, and health risks to produce estimates for 204 countries and territories from 1990 to 2019.

Prevalent cases of total CVD nearly doubled from 271 million (95% uncertainty interval [UI]: 257 to 285 million) in 1990 to 523 million (95% UI: 497 to 550 million) in 2019, and the number of CVD deaths steadily increased from 12.1 million (95% UI:11.4 to 12.6 million) in 1990, reaching 18.6 million (95% UI: 17.1 to 19.7 million) in 2019. The global trends for disability-adjusted life years (DALYs) and years of life lost also increased significantly, and years lived with disability doubled from 17.7 million (95% UI: 12.9 to 22.5 million) to 34.4 million (95% UI:24.9 to 43.6 million) over that period. The total number of DALYs due to IHD has risen steadily since 1990, reaching 182 million (95% UI: 170 to 194 million) DALYs, 9.14 million (95% UI: 8.40 to 9.74 million) deaths in the year 2019, and 197 million (95% UI: 178 to 220 million) prevalent cases of IHD in 2019. The total number of DALYs due to stroke has risen steadily since 1990, reaching 143 million (95% UI: 133 to 153 million) DALYs, 6.55 million (95% UI: 6.00 to 7.02 million) deaths in the year 2019, and 101 million (95% UI: 93.2 to 111 million) prevalent cases of stroke in 2019.

Cardiovascular diseases remain the leading cause of disease burden in the world. CVD burden continues its decades-long rise for almost all countries outside high-income countries, and alarmingly, the age-standardized rate of CVD has begun to rise in some locations where it was previously declining in high-income countries. There is an urgent need to focus on implementing existing cost-effective policies and interventions if the world is to meet the targets for Sustainable Development Goal 3 and achieve a 30% reduction in premature mortality due to noncommunicable diseases.

## Highlights

• The burden of CVD, in number of DALYs and deaths, continues to increase globally.
• CVD burden attributable to modifiable risk factors continues to increase globally.
• Countries should invest in existing cost-effective public health programs and clinical interventions to target modifiable risks, promote healthy aging across the lifespan, and reduce disability and premature death due to CVD.

Cardiovascular disease (CVD) remains a major cause of premature mortality and rising health care costs (1,2). Cardiometabolic, behavioral, environmental, and social risk factors are major drivers of CVD. Consistent, comparable, and systematic analysis of long-term trends and patterns in global CVD are essential to guide public policy and provide benchmarks for decision makers. Beginning with ischemic heart disease (IHD) and stroke, this article provides information on the burden of CVD, including 13 underlying causes of cardiovascular death and 9 related risk factors at the global, regional, and national levels (Supplemental Figure 1).

This paper explores CVD trends from 1990 to 2019 and examines the extent to which population growth and aging explain the observed trends, sex differences, and regional patterns and how the epidemiology of the disease itself is changing. These trends show us where in the world CVD mortality and burden are increasing or declining and where progress has stalled (3). For each of the contributing causes of cardiovascular death and risk factors examined, we identify which regions and countries have the highest and lowest estimates of prevalent cases and number of deaths, as well as summary measures including number of years of life lost (YLLs), number of years lived with disability (YLDs), and the magnitude and temporal trends in disability-adjusted life years (DALYs) (1). For each section, the article also addresses how the summary measures of health and disease discussed inform investments in cardiovascular research, their implications for clinical and public health practice, and implications for health system development and national and regional policy.

To improve accessibility across a wide range of audiences, we have structured the review such that each section can be read independently for those most interested in a subset of causes or risks. Discussion pertinent to each topic is included within every section. This article is a collaborative effort involving the *Journal of the American College of Cardiology*, the National Heart, Lung, and Blood Institute, and the Institute for Health Metrics and Evaluation at the University of Washington designed to provide crucial population-level information that can guide action for CVD and risk factor prevention, treatment, and control (2).

## Global Burden of Disease Study Methods

GBD 2019 is a multinational collaborative research study that estimates disease burden for every country in the world (1,4). The study is an ongoing effort, updated annually, and is designed to allow for consistent comparison over time from 1990 to 2019, by age and sex, and across locations. The study produces standard epidemiological measures such as incidence, prevalence, and death rates as well as summary measures of health, such as DALYs. DALYs represent the sum of years of life lost prematurely and years lived with disability; can be estimated from life tables, estimates of prevalence, and disability weights; and may be expressed as counts or rates. Annual updates to the study include new diseases, new data sources, and updates to methods. All results are available via the GBD Compare website (5), and all input data is identified via the Global Health Data Exchange website (6). The study is performed in compliance with Guidelines for Accurate and Transparent Health Estimates Reporting (GATHER) guidelines for reporting health estimates (1). Detailed methods of GBD 2019 are reported in the Supplemental Appendix and summarized here.

Each CVD cause and related health states were identified with standard case definitions. IHD represented acute myocardial infarction, chronic stable angina, chronic IHD, and heart failure due to IHD. Myocardial infarction was defined according to the Fourth Universal Definition of Myocardial Infarction and was adjusted to include out-of-hospital sudden cardiac death. Stable angina was defined according to the Rose Angina Questionnaire. Stroke was defined according to the World Health Organization definition and was estimated separately for 3 subcategories: 1) ischemic stroke (IS); 2) intracerebral hemorrhage; and 3) subarachnoid hemorrhage (7). Lower extremity peripheral artery disease (PAD) was defined by an ankle brachial index of <0.9. Symptomatic PAD was defined as self-report of symptoms of claudication among those with an ankle brachial index of <0.9. Atrial fibrillation (AF) and atrial flutter (AFL) were defined by electrocardiogram. Hypertensive heart disease (HHD) was defined as symptomatic heart failure due to the direct and long-term effects of hypertension. Cardiomyopathy was defined as symptomatic heart failure due to primary myocardial disease or toxin exposure to the myocardium, such as alcohol. Acute myocarditis was defined as an acute and time-limited condition due to myocardial inflammation using health system administrative data. Endocarditis and rheumatic heart disease (RHD) were defined by their clinical diagnosis. Estimates of RHD include cases identified by clinical history and physical examination, including auscultation or standard echocardiographic criteria for definite disease.

Mortality was estimated by using vital registration data coded to the International Classification of Disease (ICD) system or household mortality surveys known as verbal autopsy. Statistical methods were used to increase the comparability of mortality data sources, including the reclassification of codes that are nonspecific or unspecified, noise reduction algorithms, and Bayesian geospatial regression software (CODem, the cause of death ensemble model, Institute for Health Metrics and Evaluation, Seattle, Washington) that used location-specific covariates to create smoothed time trends for 204 countries and territories by borrowing strength over age, space, and time. GBD 2019 allowed for the production of estimates with uncertainty intervals (UIs) for all locations in every year, even when data were sparse or missing. Disease incidence and prevalence were produced by using a broad range of population-representative data sources identified by literature review and via study collaborations, including scientific reports of cohorts and registries, population surveys, microdata from registry and cohort studies, and health system administrative data. Consistent disease estimates were produced by using epidemiologic state-transition disease modeling software, DisMod-MR (Institute for Health Metrics and Evaluation), and Bayesian meta-regression software, MR-BRT (Institute for Health Metrics and Evaluation), that adjusted for study-level differences in measurement methods and case definitions. Risk factor exposures were estimated by using population-representative survey and surveillance data and geospatial Gaussian process regression models that borrowed strength across time and geography.

DALYs were calculated as the sum of YLLs, based on a reference maximum observed life expectancy, and YLDs based on standardized disability weights for each health state. Population-attributable fractions were calculated independently by risk factor by using risk exposures, estimates of relative risk based on meta-analyses, and theoretical minimum risk levels determined for each risk-outcome pair. Adjustment was made for comorbidity by simulating 40,000 individuals in each age-sex-country-year exposed to the independent probability of acquiring conditions based on their prevalence. The 95% UIs reported for each estimate used 1,000 draws from the posterior distribution of models, reported as the 2.5th and 97.5th values of the distribution. Age standardization was performed via the direct method, applying a global age structure from the year 2019. The University of Washington Institutional Review Board Committee approved the Global Burden of Diseases, Injuries, and Risk Factors Study (STUDY00009060).

## Cardiovascular Diseases

### Total CVDs

**Figure 1:**
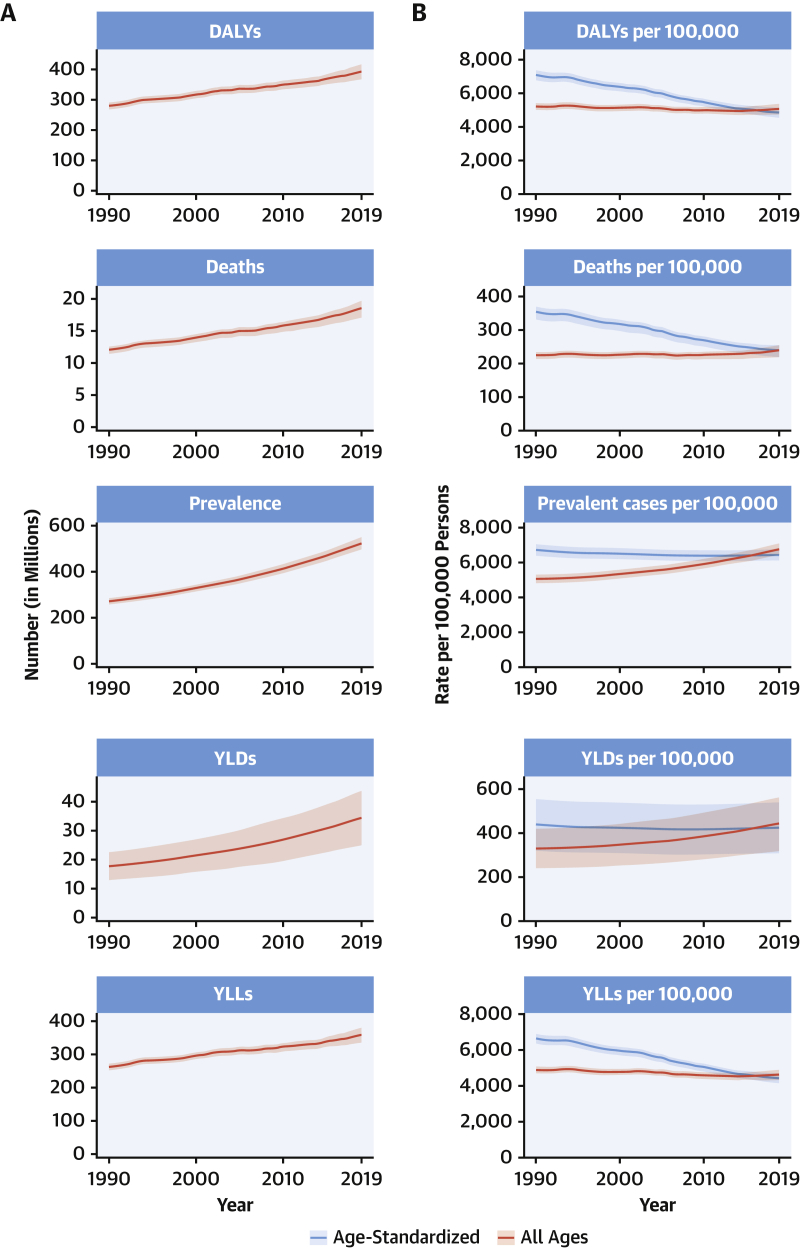
Total Numbers and Rates of Cardiovascular Diseases **(A)** Total number of DALYs, deaths, prevalent cases, YLDs, and YLLs due to cardiovascular diseases, 1990 to 2019. **Shaded region**s represent 95% uncertainty intervals. **(B)** Age-standardized and all-ages DALY, death, prevalence, YLD, and YLL rates of cardiovascular diseases, 1990 to 2019. **Shaded regions** represent 95% uncertainty intervals. DALYs = disability-adjusted life years; YLDs = years lived with disability; YLLs = years of life lost.

CVDs are common, have poor survival, and are increasing worldwide (Central Illustration). Prevalent cases of total CVD nearly doubled from 271 million (95% UI: 257 to 285 million) in 1990 to 523 million (95% UI: 497 to 550 million) in 2019, and the number of CVD deaths steadily increased from 12.1 million (95% UI: 11.4 to 12.6 million) in 1990, reaching 18.6 million (95% UI: 17.1 to 19.7 million) in 2019 (Figure 1A). The global trends for DALYs and YLLs also increased significantly, and YLDs doubled from 17.7 million (95% UI: 12.9 to 22.5 million) to 34.4 million (95% UI: 24.9 to 43.6 million) over that period.

**Central Illustration:**
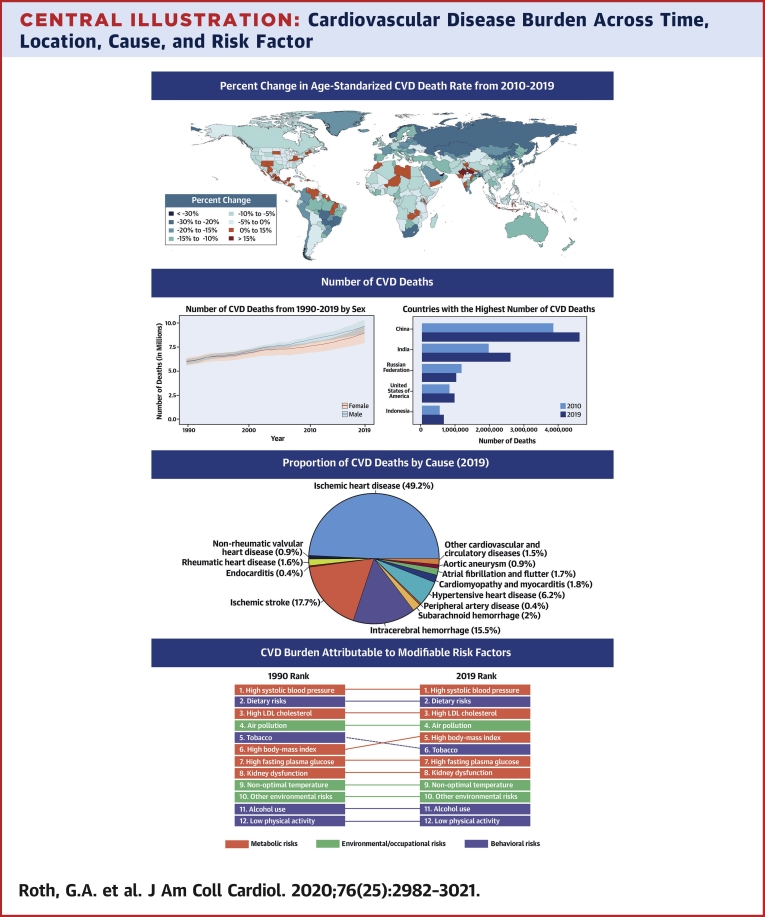
Cardiovascular Disease Burden Across Time, Location, Cause, and Risk Factor **Percent Change in Age-Standardized CVD Death Rate from 2010-2019.** Map of the percent change in age-standardized CVD mortality rate from 2010 to 2019.**Number of CVD Deaths.** Total number of deaths due to CVD by sex, 1990 to 2019; total number of deaths due to CVD in 2010 and 2019 among the countries with the highest number of CVD deaths in 2019. **Proportion of CVD Deaths by Cause (2019)**. Proportion of total CVD deaths in 2019 by underlying causes. **CVD Burden Attributable to Modifiable Risk Factors**. Comparison of the rankings of CVD DALYs attributable to modifiable risk factors in 1990 and 2019. CVD = cardiovascular disease; DALYs = disability-adjusted life years; LDL = low-density lipoprotein.

At the country level, age-standardized mortality rates for total CVD were highest in Uzbekistan, Solomon Islands, and Tajikistan and were lowest in France, Peru, and Japan, where rates were 6-fold lower in 2019. From 1990 to 2019, large declines in the age-standardized rates of death, DALYs, and YLLs, together with small gradual reductions in age-standardized rates for prevalent cases and YLDs, suggest that population growth and aging are major drivers of the increase in total CVD (Figure 1B).

In 2019, total CVD DALYs were higher in men than women before age 80 to 84 years (Supplemental Figure 2). After this age, the pattern reverses. The sex differences in DALYs is most striking between ages 30 and 60 years (men greater) and age >80 years (women greater). The excess CVD deaths in women beginning at ages 80 to 84 years should focus attention to cause-specific mortality at older ages and have implications for secondary prevention strategies.

Among women, the age-standardized rates for DALYs were highest in Central Asia, Oceania, North Africa and the Middle East, and Eastern Europe; and lowest in High-Income Asia Pacific, Australasia, and Western Europe (Supplemental Figure 3). Among men, age-standardized rates for DALYs were highest in Central Asia, Eastern Europe, and Oceania; and lowest in High-Income Asia Pacific, Australasia, Western Europe, and Andean Latin America. At the country level, the highest age-standardized rates were estimated for many of the islands of Oceania, Uzbekistan, and Afghanistan, while the lowest rates for DALYs were seen in Japan, France, and Israel (Supplemental Figure 4). These regional and national differences in total CVD burden and mortality reflect differences in prevalence of CVD risk factors as well as access to health care (8). Differences in access to effective primary and secondary prevention strategies may also play a role in differences in total CVD burden, especially in low- and middle-income countries (LMICs) (9).

Global patterns of total CVD have significant implications for clinical practice and public health policy development (10). Prevalent cases of total CVD are likely to increase substantially as a result of population growth and aging, especially in Northern Africa and Western Asia, Central and Southern Asia, Latin America and the Caribbean, and Eastern and Southeastern Asia, where the share of older persons is projected to double between 2019 and 2050 (11,12). Increased attention to promoting ideal cardiovascular health and healthy aging across the lifespan is necessary (13). Equally importantly, the time has come to implement feasible and affordable strategies for the prevention and control of CVD and to monitor results (14).

### Ischemic heart disease

**Figure 2:**
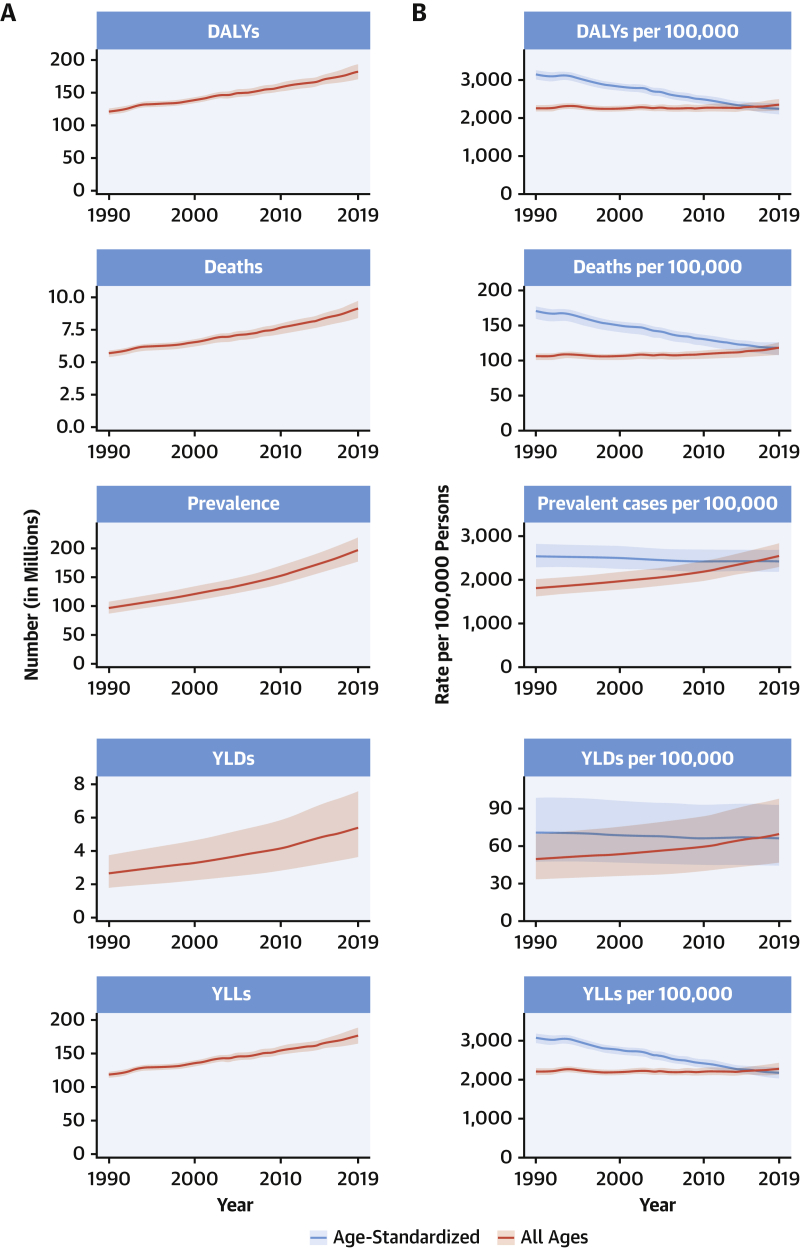
Total Numbers and Rates of Ischemic Heart Disease **(A)** Total number of DALYs, deaths, prevalent cases, YLDs, and YLLs due to ischemic heart disease, 1990 to 2019. **Shaded regions** represent 95% uncertainty intervals. **(B)** Age-standardized and all-ages DALY, death, prevalence, YLD, and YLL rates of ischemic heart disease, 1990 to 2019**. Shaded regions** represent 95% uncertainty intervals. Abbreviations as in Figure 1.

The total number of DALYs due to IHD has risen steadily since 1990, reaching 182 million (95% UI: 170 to 194 million) DALYs and 9.14 million (95% UI: 8.40 to 9.74 million) deaths in the year 2019 (Figure 2A). GBD 2019 estimated 197 million (95% UI: 178 to 220 million) prevalent cases of IHD in 2019.

Global age-standardized rates for DALYs, deaths, and prevalent cases declined over this time period, indicating that, on average, global increases in IHD have been due to population growth and aging, although even the age-standardized death rate is estimated to be increasing in many locations across South, East, and Southeastern Asia, including China (Figure 2B). Increasing absolute numbers of incident and prevalent IHD cases in most countries mean that national health systems will need to address increasing demand for IHD-related preventive and therapeutic services as these trends continue.

At the global level, substantially more total DALYs due to IHD were experienced by men than women. IHD DALYs rose rapidly for men beginning at age 30 years (Supplemental Figure 5). Men ages 45 to 49 years had almost as many DALYs due to IHD as women ages 65 to 69 years.

Age-standardized DALY rates due to IHD were highest in Eastern Europe, Central Asia, Oceania, and the Middle East/North Africa regions (Supplemental Figure 6). At the country level, extremely high rates were estimated for Uzbekistan, Ukraine, Tajikistan, and many of the islands of Oceania, with the lowest levels in Japan, Republic of Korea, and France (Supplemental Figure 7). Higher exposure to risks including tobacco and excessive alcohol use and restricted access to preventive health care may partly explain these patterns.

IHD remains a major threat to public health, and overall burden is increasing globally. In some locations over the past 5 years, including parts of the United States and United Kingdom, age-standardized IHD death rates are increasing, suggesting that long-term declines in IHD due to improved prevention and health care are no longer occurring in these locations. Health systems and countries need to focus on delivering effective interventions that will reverse these trends, including those that prevent and control diabetes, decrease obesity and high cholesterol, improve diet and physical activity, reduce tobacco and excessive alcohol use, integrate “polypills” for elevated blood pressure and related conditions, improve pre- and in-hospital care for acute coronary syndrome, and improve survival and quality of life for those living with the long-term sequelae of IHD, including heart failure. Social and economic factors remain fundamental drivers of IHD, suggesting that multisectoral interventions are needed to eradicate this disease (15).

### Stroke

The total number of prevalent strokes, deaths, and DALYs due to stroke increased steadily from 1990, reaching 101 million (95% UI: 93.2 to 111 million) prevalent (85.3% [95% UI: 82.6% to 88.2%] increase) stroke survivors, 6.55 million (95% UI: 6.00 to 7.02 million) deaths from stroke (43.3% [95% UI: 31.0% to 55.4%] increase), and 143 million (95% UI: 133 to 153 million) DALYs due to stroke (32.4% [95% UI: 22.0% to 42.2%] increase) in 2019, with the bulk of the burden outside of the high-income world (Supplemental Figures 8A and 9A). Of 12.2 million (95% UI: 11.0 to 13.6 million) incident stroke cases, 7.63 million (95% UI: 6.57 to 8.96 million) (62.4%) were IS, 3.41 million (95% UI: 2.97 to 3.91 million) (27.9%) were intracerebral hemorrhages, and 1.18 million (95% UI: 1.01 to 1.39 million) (9.7%) were subarachnoid hemorrhages (Supplemental Figures 10A, 11A, and 12A).

Globally, age-standardized rates for deaths and DALYs due to stroke substantially declined over the same period of time (Supplemental Figures 8B, 10B, 11B, and 12B), suggesting that: 1) preventive measures are very effective at lowering risk of both ischemic and hemorrhagic stroke; and 2) on average, global increases in stroke burden have been largely due to population growth and aging. Importantly, age-standardized rates for prevalence of stroke survivors have increased since 1990 in several locations including in China, Indonesia, and parts of the United States. Age-standardized death rates also increased in some locations, including Indonesia and the Philippines.

Age-standardized rates of DALYs and deaths due to stroke were substantially greater in men compared to women, but prevalence was greater in women, suggesting the possibility of greater risk of death and disability in men but better stroke survival in women. Similar patterns were observed in men and women with IS, intracerebral hemorrhage, and subarachnoid hemorrhage.

**Figure 3:**
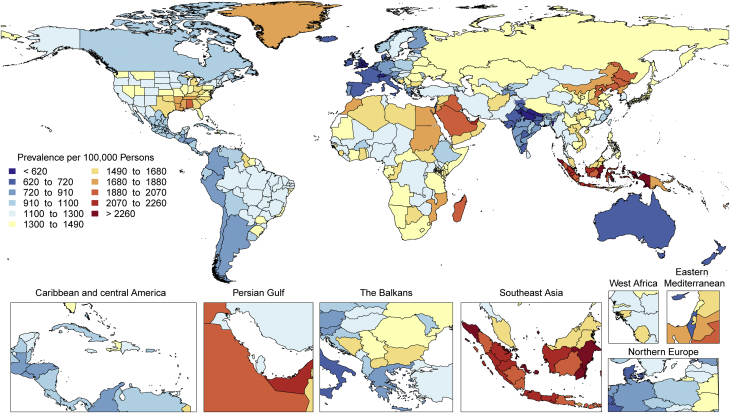
Map of Age-Standardized Prevalence of Stroke Survivors in 2019

There is tremendous regional disparity in the burden of stroke. Age-standardized rates of deaths and DALYs due to stroke were highest in Oceania, Central Asia, East Asia, Southeast Asia, Eastern Europe, and sub-Saharan Africa (Supplemental Figure 13), and prevalence of stroke survivors was highest in Oceania, Southeast Asia, East Asia, and the Middle East/North Africa regions (Figure 3). These data suggest that applying preventive strategies, such as treatment of elevated blood pressure and cholesterol levels, can have major health benefits where stroke burden remains high, especially in those regions that did not show substantial decline over the past decades (i.e., Central Asia, Southern sub-Saharan Africa).

Stroke remains the second-leading cause of death, and stroke burden in terms of DALYs is increasing. Primary stroke prevention strategies are not sufficiently effective as currently implemented in many countries. Large (15- to 20-fold) geographic variations in age-standardized stroke DALYs and mortality rates and moderate (4-fold) geographic variations in age-standardized prevalence may be related to the variations in stroke risk factors or the quality of and/or access to preventative care, acute stroke care, and stroke rehabilitation across the globe. Global and national health systems need to focus on new approaches for delivering stroke prevention that can reverse these trends, including strategies to provide equal access to quality health care and prevention across all populations, and proven effective interventions that reduce mortality and improve outcomes post-stroke.

### Hypertensive heart disease

The global prevalence of HHD has risen steadily over the last 3 decades, as have the total number of deaths, DALYs, YLLs, and YLDs due to this disease. In 2019, HHD was the main cause of 1.16 million (95% UI: 0.86 to 1.28 million) deaths and 21.5 million (95% UI: 16.4 to 23.9 million) DALYs annually, with a global prevalence of 18.6 million (95% UI: 13.5 to 24.9 million) cases (Supplemental Figure 14A).

The age-standardized prevalence and YLDs of HHD per 100,000 persons have been constant over time, and corresponding age-standardized rates of deaths, DALYs, and YLLs declined steadily until the mid-2000s and have leveled off since then (Supplemental Figure 14B). The trends for the absolute and age-standardized estimates closely resemble those for high blood pressure. Taken together, the increasing global prevalence and rates of HHD can be explained by population growth and aging, but the declining age-standardized rates of adverse outcomes are similar to those for IHD and stroke and are likely an effect of improved secondary prevention.

The age distributions of DALYs due to HHD are very similar in women and men until age 70 years (Supplemental Figure 15). This contrasts to the age distributions for high blood pressure, in which men are overrepresented at younger ages. The similarities by sex in DALYs calls for an equal attention to the clinical prevention and treatment of HHD in women and men.

**Figure 4:**
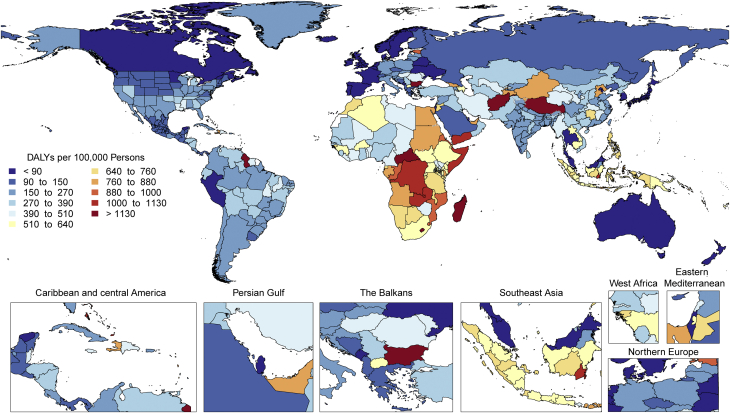
Map of Age-Standardized DALYs Due to Hypertensive Heart Disease in 2019 DALYs = disability-adjusted life years.

The highest age-standardized rates of DALYs due to HHD are noted in Africa (Figure 4). DALYs due to HHD are particularly higher in Central sub-Saharan Africa, followed by most of Africa except Western sub-Saharan Africa, and Oceania. A similar geographic pattern exists for high blood pressure (with the exception of high rates of DALYs in men from high blood pressure in Central Asia and Eastern Europe). An overrepresentation of DALYs due to HHD in women can be noted in Central and Eastern sub-Saharan Africa (Supplemental Figure 16). The significance of this is uncertain because no such peaks are noted for high blood pressure or other contributing factors, and it can represent regional differences in disease coding, including that of peripartum cardiomyopathy. Inequalities in access to both primary and secondary prevention are a potential cause of these regional and sex differences.

The global prevalence of HHD and absolute rates of adverse outcomes are expected to continue to rise due to population growth and aging. The age-standardized rates of adverse outcomes due to HHD are no longer decreasing, and with continuing global increases in obesity and diabetes, corresponding increases in left ventricular hypertrophy and other aspects of HHD are likely. Intensified global and regional efforts to lower blood pressure and control other risk factors are needed, for example with population-wide reductions in dietary sodium intake. Continued surveillance of inequalities in adverse outcomes due to high blood pressure are necessary to inform such efforts.

### Congenital heart anomalies

**Figure 5:**
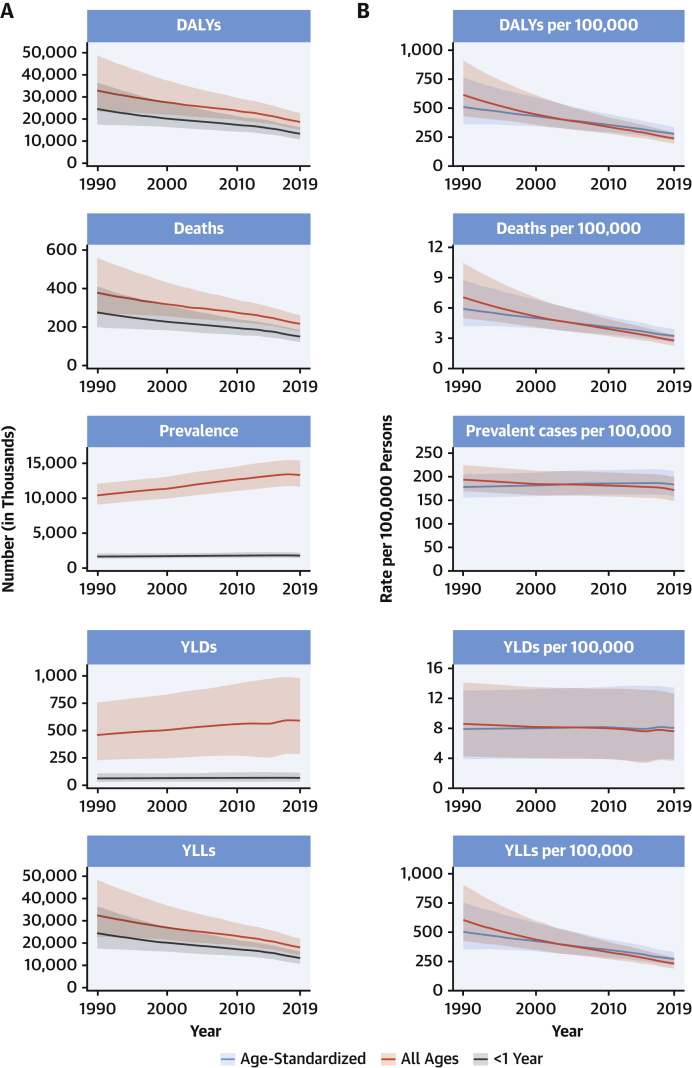
Total Numbers and Rates of Congenital Heart Anomalies **(A)** Total number and number among children younger than 1 year of DALYs, deaths, prevalent cases, YLDs, and YLLs due to congenital heart anomalies, 1990 to 2019. **Shaded regions** represent 95% uncertainty intervals. **(B)** Age-standardized and all-ages DALY, death, prevalence, YLD, and YLL rates of congenital heart anomalies, 1990 to 2019. **Shaded regions** represent 95% uncertainty intervals. Abbreviations as in Figure 1.

A total of 3.12 million (95% UI: 2.40 to 4.11 million) babies were born with congenital heart anomalies (CHA) in 2019, representing 2,305.2 per 100,000 live births (95% UI: 1,772.9 to 3,039.2 per 100,000 live births), a total of 13.3 million (95% UI: 11.5 to 15.4 million) people were living with CHA, and CHA was the underlying cause of 217,000 deaths (95% UI: 177,000 to 262,000 deaths), of which 150,000 deaths (95% UI: 120,000 to 184,000 deaths) were in infants <1 year (Figure 5A). The all-ages rates of DALYs, YLLs, and YLDs for CHA in 2019 were 241.6 per 100,000 (95% UI: 196.1 to 292.7 per 100,000), 234.0 per 100,000 (95% UI: 189.8 to 285.7 per 100,000), and 7.6 per 100,000 (95% UI: 3.7 to 12.7 per 100,000), respectively (Figure 5B).

Between 1990 and 2019, the global CHA birth rate was largely unchanged, but the all-ages death rate due to CHA declined 60.4% (95% UI: –71.9% to –41.4%) from 7.1 per 100,000 (95% UI: 5.0 to 10.5 per 100,000) in 1990 to 2.8 per 100,000 (95% UI: 2.3 to 3.4 per 100,000) in 2019. Age-standardized prevalence, death, and DALY rates remain lower than all-ages rates in low-income countries because of younger populations, higher birth rates of CHA, and much less access to the intervention that is needed to allow for the survival of children born with CHA.

The proportion of all infant deaths caused by CHA increased in all quintiles of the sociodemographic index (SDI) since 1990 except the highest because improvement in CHA mortality has lagged behind that of other causes, most notably infectious, respiratory, and diarrheal diseases (16). In low-SDI regions, CHA death rates have declined by 42.1% (95% UI: –61.9% to 12.1%) since 1990, compared with declines of 71.3% (95% UI: –74.7% to –64.6%) in high-SDI subgroups. Unlike other CVDs, prevention has limited ability to reduce the burden of CHA after birth; improvements in early diagnosis and access to cardiac surgery are the only solutions.

The total number of global CHA births remained steady, global CHA deaths decreased by 42.7% (95% UI: –59.3% to –15.2%), and the global number of individuals living with CHA increased by 28.2% (95% UI: 26.3% to 30.1%). There were increases in the 15- to 49-year and the 50- to 69-year age groups, in which the number of individuals living with CHA grew by 41.6% (95% UI: 40.0% to 43.2%) and 117.3% (95% UI: 114.7% to 120.0%) to 4.21 million (95% UI: 3.67 to 4.76 million) and 1.31 million (95% UI: 1.13 to 1.51 million), respectively, reflecting a cohort effect from the time of birth. Most of the prevalence increase occurred outside of high-income countries (HICs) and was due to improvements in survival and population growth. Health systems will be increasingly burdened with adolescents and adults needing care for their congenital heart conditions.

The very young age distribution in LMICs and large burden of YLLs due to CHA should drive a global mandate for investment in data collection and resources to expand access to infant heart surgery in regions in most need. In low-income countries with very young populations, the prevalence and mortality from RHD parallel lack of access to cardiac surgery services for CHA, and pooling resources to care for both of these diseases makes a stronger case for investment in surgical services (that includes both in-country surgical personnel and nongovernmental organizations) than for either disease alone.

### Rheumatic heart disease

The prevalence of RHD has been rising steadily since 1990, reaching 40.5 million (95% UI: 32.1 to 50.1 million) currently affected in 2019 (Supplemental Figure 17A). Deaths decreased until 2012 but have stabilized since then and even started increasing since 2017 (306,000 [95% UI: 259,000 to 340,000] in 2019). DALYs and YLLs have slowly decreased to 10.7 million (95% UI: 9.21 to 12.1 million) and 8.68 million (95% UI: 7.43 to 9.77 million), respectively, in 2019, whereas YLDs have increased to 1.99 million (95% UI: 1.20 to 3.04 million).

Age-standardized rates for RHD prevalence have closely tracked with all-age rates, but age-standardized mortality has exceeded all-age mortality until the past few years, highlighting the differential mortality risk due to RHD observed in regions with differing age structures and levels of development (Supplemental Figure 17B). The narrowing of this differential tells a positive story about RHD care, although the dominance of China and India in the overall figures may mask persistent differentials in other high-prevalence countries and regions. The continuing increase in prevalence cannot be attributed to changes in age structure but likely reflects increased global awareness, the increasing availability of echocardiography for case definition, improved survival in some places, and the chronic nature of RHD.

Globally, the prevalence of RHD is estimated to peak between 20 and 29 years, remain relatively stable until 40 years, and then begin a steady decline, likely reflecting decreasing survival at older ages. Sex distribution is equal until the age of 15 years, after which women bear a higher burden in terms of prevalence across nearly all world regions. Higher rates of RHD in post-pubertal females are well described, but incompletely understood, and may reflect the interaction of biological, social, and environmental risk factors.

**Figure 6:**
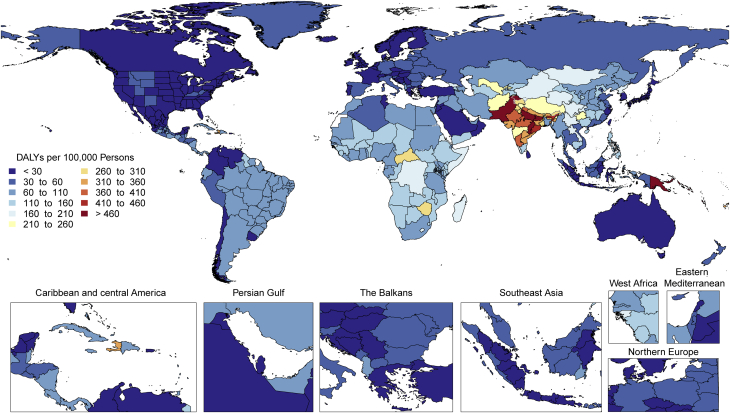
Map of Age-Standardized DALYs Due to Rheumatic Heart Disease in 2019 DALYs = disability-adjusted life years.

RHD burden continues to show substantial global heterogeneity (Supplemental Figure 18). The highest age-standardized DALY rates are seen in Oceania and South Asia (627.4 per 100,000 [95% UI: 404.1 to 918.0 per 100,000] and 348.5 per 100,000 [95% UI: 272.4 to 412.2 per 100,000], respectively) with the lowest in the highest-income regions (<25 per 100,000). Furthermore, although some regions have shown large declines between 1990 and 2019 (Eastern and Central Europe, East Asia, Central Latin America, high-income Asia Pacific, and North Africa and the Middle East), there are also regions with only modest improvements and regions where very little improvement has been seen (Oceania, South Asia, the Caribbean, and sub-Saharan Africa). There is also substantial within-region national and subnational variability in RHD DALYs, some examples of which include substantially higher RHD DALYs in Guyana and Haiti compared to the rest of Latin America and the Caribbean and the heterogeneity seen in the subnational data on RHD DALYs from India (Figure 6).

RHD burden is highest among the world’s most disadvantaged populations. The most marginalized and poorest populations regionally, nationally, and at a subnational level are not showing signs of improvement and continue to die early from RHD. Health systems in LMICs should support the recommendations of the 2018 World Health Assembly Global RHD Resolution with increased multisector investment in primary health care, improved sanitation and housing, infrastructure, secure medication supply chains, evidence-based RHD screening, prevention and management, and tertiary capacity to care for patients at the severe end of the RHD spectrum. RHD burden could be greatly decreased with an effective group A streptococcal vaccine, with several promising candidates currently in development (17). Future data collection is needed at subnational levels to identify and monitor populations with ongoing high RHD burdens.

### Cardiomyopathy and myocarditis

DALYs due to cardiomyopathy and myocarditis have increased from 7.06 million (95% UI: 6.30 to 8.63 million) to 9.14 million (95% UI: 7.86 to 10.0 million) over the past 30 years, a pattern that is also seen in the rise of deaths from 238,000 (95% UI: 212,000 to 257,000) to 340,000 (95% UI: 285,000 to 371,000) (Supplemental Figure 19A). However, over the same period, the age-standardized rate of death has decreased from 8.0 per 100,000 (95% UI: 6.4 to 8.6 per 100,000) to 5.6 per 100,000 (95% UI: 4.5 to 6.3 per 100,000) in men and 5.8 per 100,000 (95% UI: 4.4 to 6.4 per 100,000) to 3.3 per 100,000 (95% UI: 2.7 to 3.6 per 100,000) in women. The age-standardized morbidity and mortality for men and women in 2019 remains different, at 6.5 per 100,000 YLDs (95% UI: 4.3 to 9.3 per 100,000 YLDs) and 4.2 per 100,000 YLDs (95% UI: 2.8 to 6.0 per 100,000 YLDs) and 148.9 per 100,000 YLLs (95% UI: 120.2 to 168.7 per 100,000 YLLs) and 71.4 per 100,000 YLLs (95% UI: 61.0 to 79.9 per 100,000 YLLs), respectively.

The prevalence and related mortality of cardiomyopathy and myocarditis increase throughout adulthood in both sexes, with a larger proportion of cases in men than in women. When myocarditis alone is considered, a similar trend was observed. Referring to 2019, in the age between 35 and 39 years, when myocarditis can commonly occur (18,19), the rate of myocarditis is 6.1 per 100,000 (95% UI: 4.2 to 8.7 per 100,000) in men and 4.4 per 100,000 (95% UI: 3.0 to 6.3 per 100,000) in women. Similar figures can be found in the range of ages between 20 and 44 years.

The increased prevalence associated with aging is more pronounced in cardiomyopathies than in myocarditis. Approximately a 6-fold higher age-related increase of other cardiomyopathy was observed in men between 35 and 39 years (10.8 per 100,000 [95% UI: 6.5 to 17.4 per 100,000]) versus those between 80 and 84 years (698.5 per 100,000 [95% UI: 429.6 to 1064.8 per 100,000]) compared with myocarditis in men of 35 to 39 years (6.1 per 100,000 [95% UI: 4.2 to 8.7 per 100,00]) versus those of 80 to 84 years (63.0 per 100,000 [95% UI: 43.6 to 87.9 per 100,000]). The relatively greater rise of other cardiomyopathy compared to myocarditis prevalence with age is constant, from 1990 to 2019.

Myocarditis-related mortality rate between 35 and 39 years was 0.2 per 100,000 (95% UI: 0.2 to 0.3 per 100,000) in men compared to 0.1 per 100,000 (95% UI: 0.1 to 0.2 per 100,000) in women in 2019. Alternatively, myocarditis resulted in death in 1 in 72 men (585 deaths per 42,200 incident cases) and 1 in 87 (324 deaths per 28,100 incident cases) women in this age bracket who were diagnosed in 2019. Between ages 80 and 84 years, the rate was higher, at 1 death for every 19 incident cases of myocarditis in men (1,800 deaths per 34,400 incident cases) and 1 death for every 15 incident cases of myocarditis in women (2,260 deaths per 34,900 incident cases).

Marked regional variations in other cardiomyopathy in age-standardized DALYs in both sexes were observed in 2019 (Supplemental Figure 20). These ranged from 5 to 46 DALYs per 100,000 in South Asia, East Asia, Andean Latin America, high-income Asia Pacific, and Central Latin America to 96 to 147 per 100,000 in Oceania, the Caribbean, high-income North America, and Western and Eastern sub-Saharan Africa to 230 to 271 per 100,000 in Central Asia, Eastern Europe, and Southern and Central sub-Saharan Africa. These regional variations may be explained by a higher prevalence of peripartum cardiomyopathy in Africa, Chagas disease in Central and South America, and alcohol-related cardiomyopathy in parts of Central Europe and Russia (20).

**Figure 7:**
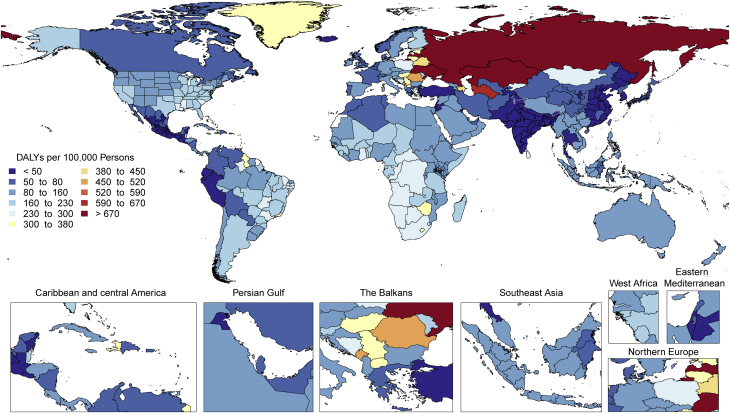
Map of Age-Standardized DALYs Due to Cardiomyopathy and Myocarditis in 2019 DALYs = disability-adjusted life years.

Although the morbidity and mortality rates from cardiomyopathies and myocarditis collectively present a substantial global disease burden in 2019, the regional differences in the burden (Figure 7) suggest that public health interventions should be tailored to the specific etiologies of cardiomyopathy to lower these rates in the future.

### Alcoholic cardiomyopathy

The global prevalence of alcoholic cardiomyopathy (AC) estimated by GBD 2019 was 708,000 cases (95% UI: 545,000 to 924,000 cases), approximately 9.1 cases per 100,000 (95% UI: 7.0 to 11.9 cases per 100,000) (Supplemental Figure 21A). Globally, AC was responsible for 71,700 deaths (95% UI: 60,200 to 82,000 deaths), 2.38 million YLLs (95% UI: 2.00 to 2.73 million YLLs), and 60,100 YLDs (95% UI: 38,500 to 88,300 YLDs). The total number of DALYs due to AC was 2.44 million (95% UI: 2.05 to 2.78 million). After a rapid increase from 1999, DALYs, deaths, and YLLs started decreasing from 2005 to 2019. The global prevalence and YLDs due to AC are increasing.

All-age and age-standardized rates of DALYs, deaths, prevalent cases, YLDs, and YLLs declined from 2005 to 2019 (Supplemental Figure 21B). This indicates that the global increase in AC prevalence is related, in part, to population growth and aging. Several countries in East Asia and the Caribbean regions showed opposite trends, with increasing age-standardized values for almost all indicators. Factors that might explain these regional differences remain incompletely understood.

At the global level, substantially more total DALYs due to AC were experienced by men than women. DALYs from AC rose rapidly among men beginning from age 25 years, being significantly higher in men than women across all ages (Supplemental Figure 22). Women are generally considered more susceptible to alcohol-induced damages than men, which may reflect sex-specific differences in alcohol consumption, type, blood level, distribution, or metabolism. However, the higher level of alcohol consumption and the higher frequency of alcohol problems among men could justify the observed higher rate of DALYs.

**Figure 8:**
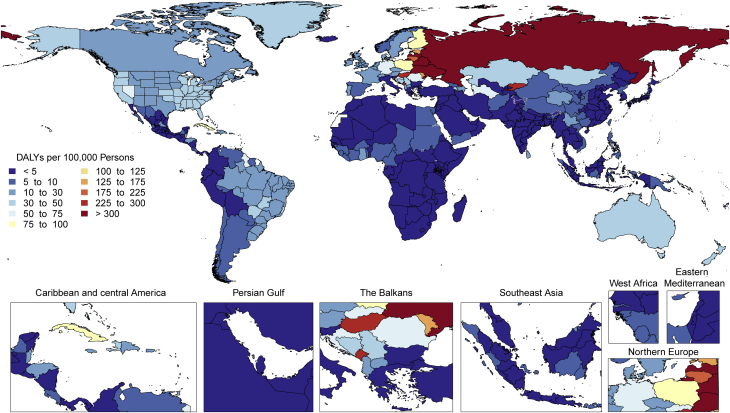
Map of Age-Standardized DALYs Due to Alcoholic Cardiomyopathy in 2019 DALYs = disability-adjusted life years.

Age-standardized DALYs due to AC were higher in Central Europe as well as in the Caribbean (Cuba), followed by Australasia, North America, Central Asia, Western Europe, and tropical Latin America (Figure 8). Extremely high rates were seen in Eastern Europe, particularly in Russia and Kyrgyzstan. The lowest levels were observed in sub-Saharan Africa, Andean Latin America, and North Africa and the Middle East. Geographic differences reflect drinking patterns among regions, for example, rates of binge drinking, as well as social and cultural behaviors, such as abstaining from alcohol.

AC represents substantial morbidity and mortality among young and middle-age people, particularly men. The age-standardized DALYs are high in several countries, and the true burden of disease could be underestimated because alcohol consumption is often underreported (21). Additional policy support for public education and awareness is needed to reduce the harmful use of alcohol. Because alcohol negatively affects cardiovascular function, increased clinician emphasis on eliminating alcohol use among people with coexisting CVD is advisable. Research should be aimed at understanding the exact mechanisms of disease, susceptibility to alcohol damage, effective public health interventions, and treatments for AC.

### AF and AFL

The total number of DALYs due to AF and AFL increased progressively from 3.79 million (95% UI: 2.96 to 4.83 million) in 1990 to 8.39 million (95% UI: 6.69 to 10.5 million) in 2019 (Supplemental Figure 23A). GBD 2019 estimated 59.7 million (95% UI: 45.7 to 75.3 million) prevalent cases of AF/AFL in 2019, about a doubling compared to the prevalent cases in 1990.

When standardized for age, prevalence of AF/AFL, DALYS, and death rates did not display marked changes between 1990 and 2019 (Supplemental Figure 23B). The age-standardized AF/AFL prevalence rate was 775.9 per 100,000 (95% UI: 592.4 to 990.8 per 100,000) in 1990 and 743.5 per 100,000 (95% UI: 571.2 to 938.3 per 100,000) in 2019. Age-standardized rates for DALYs did not change substantially between 1990, when they were 110.0 per 100,000 (95% UI: 87.7 to 139.2 per 100,000), to 2019, when they were 107.1 per 100,000 (95% UI: 86.2 to 133.7 per 100,000). Similarly, the age-standardized death rates per 100,000 were similar in 1990 at 4.3 per 100,000 (95% UI: 3.7 to 5.1 per 100,000) and 4.4 per 100,000 (95% UI: 3.7 to 5.0 per 100,000).

Comparing total numbers with age-standardized numbers suggests that global increases in AF/AFL are largely attributable to aging of the population and population growth.

Globally, under the age of 70 years, more total DALYs due to AF were experienced by men than women (Supplemental Figure 24). Between 70 and 74 years, men and women had similar DALYs. However, at age 75 years and older, this trend was reversed, with higher total DALYs experienced by women. These trends were observed in 1990 as well as 2019. These findings point toward sex-specific, age-related targets for the prevention and management of AF/AFL.

**Figure 9:**
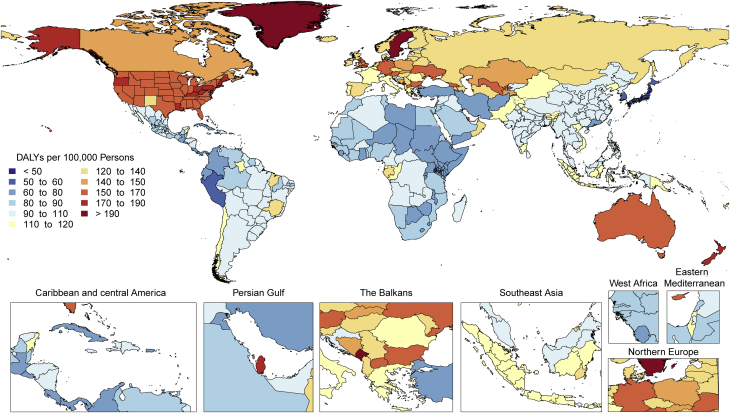
Map of Age-Standardized DALYs Due to Atrial Fibrillation and Flutter in 2019 DALYs = disability-adjusted life years.

Age-standardized DALY rates due to AF/AFL were highest in high-income North America, Australasia, Central Asia, and Europe and were observed to be lowest in the high-income Asia Pacific region (Figure 9). Also, Andean and Central Latin America, the Caribbean, sub-Saharan Africa, and North Africa and the Middle East displayed low rates. However, because of a general lack of data from these regions, more detailed studies should be performed. The reasons for this significant regional variation may include increased ascertainment in high-income regions, differential control of risk factors, and genetic factors. A detailed comparison of potential differentiating features between highest DALY rate versus lowest DALY rate regions could yield valuable additional information.

AF/AFL are the most common arrhythmias and continue to make a progressive and substantial impact on public health at a global level. In most regions, prevalence rates are increasing, suggesting that more effort is needed to improve prevention and health care for AF/AFL at a global level. Health systems and countries will need to focus their efforts to reverse these trends by aggressive attention to the reduction of risk factors such as hypertension, diabetes, and obesity; better treatment of individuals with IHD and heart failure; and improved access to medications for thromboembolism prophylaxis.

### Aortic aneurysm

**Figure 10:**
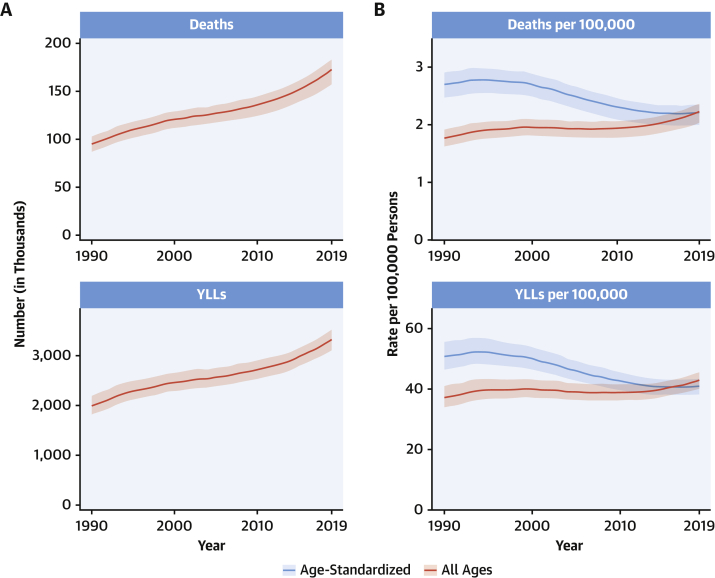
Total Numbers and Rates of Aortic Aneurysm **(A)** Total number of deaths and YLLs due to aortic aneurysm, 1990 to 2019. **Shaded regions** represent 95% uncertainty intervals. **(B)** Age-standardized and all-ages death and YLL rates of aortic aneurysm, 1990 to 2019. **Shaded regions** represent 95% uncertainty intervals. YLLs = years of life lost.

The total number of YLLs due to aortic aneurysm, including both thoracic and abdominal types, has increased steadily since 1990, reaching 3.32 million YLLs (95% UI: 3.11 to 3.52 million YLLs) and 172,000 deaths (95% UI: 157,000 to 183,000 deaths) in 2019 (Figure 10A).

Age-standardized rates for YLLs and deaths declined over this period, indicating that, overall, global increases in aortic aneurysm have been due to population growth and aging (Figure 10B). This likely reflects improvement in diagnosis and treatment of risk factors.

At the global level, men bore a substantially higher burden of YLLs due to aortic aneurysm compared with women. YLLs from aortic aneurysm rose most steeply in the sixth decade of life for men, peaking by age 70 years (Supplemental Figure 25). Women younger than age 75 years accounted for less than one-half of YLLs due to aortic aneurysm. The gap between men and women narrowed with increasing age, with YLLs in women overtaking those of men at age 90 years. This could be explained by the generally longer lifespan of women compared with men. Despite the lower prevalence of abdominal aortic aneurysm than men, growth rate in women has been shown to be more rapid, rupture during surveillance 4 times more likely, and likelihood of fatal rupture 3 times as high, even after adjustment for age (22, 23, 24). For thoracic aortic aneurysms, women have a lower incidence but are more likely to have fatal consequences than men (25).

Age-standardized YLLs due to aortic aneurysm were highest in Eastern and Central Europe as well as Southern and Tropical Latin America, with extremely high levels noted in Montenegro and parts of Brazil (Supplemental Figure 26). The lowest levels were observed in Andean Latin America, North Africa and the Middle East, and East Asia, including Mexico, Iraq, and China. Risk factors, including tobacco use and hypertension, as well as access to screening and preventive health care, may partially explain these patterns.

Aortic aneurysm remains a major public health issue, with the overall burden in terms of number of YLLs and deaths increasing globally. Because age-standardized rates for YLLs and death due to aortic aneurysm declined from 1990 to 2019, the increase may be attributable to population growth and aging. However, because the risk factors for this disease remain common, most national health systems will need to address increasing demand for preventive care, including low-cost screening modalities for abdominal aneurysms in the high-burden regions mentioned earlier. The risk factors underlying the development of aortic aneurysm are common to other CVDs, such as IHD. As such, investing health system resources in these risk factors, including hypertension management and smoking cessation, could reduce the burden of other diseases in addition to aortic aneurysm. Combined with implementation of inexpensive screening technology, such as ultrasonography, where indicated, the morbidity and mortality due to aortic aneurysm can be significantly decreased globally.

### Nonrheumatic valvular heart disease: calcific aortic valve disease

Calcific aortic valve disease (CAVD) occurs commonly among older adults with a normal trileaflet aortic valve and with a greater frequency among those with a congenital bicuspid aortic valve. CAVD is clinically important because severe obstruction causes symptoms and left ventricular dysfunction warranting surgical or transcatheter valve replacement (26). The prevalence of CAVD increases with age and is >1,000 per 100,000 beyond the age of 75 years.

Globally, both the prevalence and age-standardized prevalence of CAVD have increased steadily over the last 3 decades (Supplemental Figure 27B). In 2019, the age-standardized prevalence rose to approximately 116.3 cases per 100,000 (95% UI: 100.4 to 134.5 cases per 100,000) people, from about 45.5 cases per 100,000 (95% UI: 37.6 to 54.7 cases per 100,000) people in 1990. This increase may be attributable to increased prevalence of atherosclerotic risk factors associated with the development and progression of CAVD (27). Although age-standardized YLDs due to CAVD have increased in parallel with prevalence, there has been no significant change in age-standardized DALYs, most likely because most of the DALYs due to CAVD accrue from YLLs, whereas deaths due to CAVD (and consequently, age-standardized YLLs) have not increased over the years. The ready availability of aortic valve replacement in regions of the world where CAVD is most prevalent may be an important check on mortality.

In HICs, the age-standardized prevalence of CAVD is similar among both men and women, while the prevalence among men exceeds that among women to varying degrees in the other regions of the world. Population-based studies of the prevalence of nonrheumatic valvular heart disease remain rare, and further research is needed to understand if sex differences in prevalence are due to differences in disease burden, health-seeking behavior, or rates of diagnosis.

**Figure 11:**
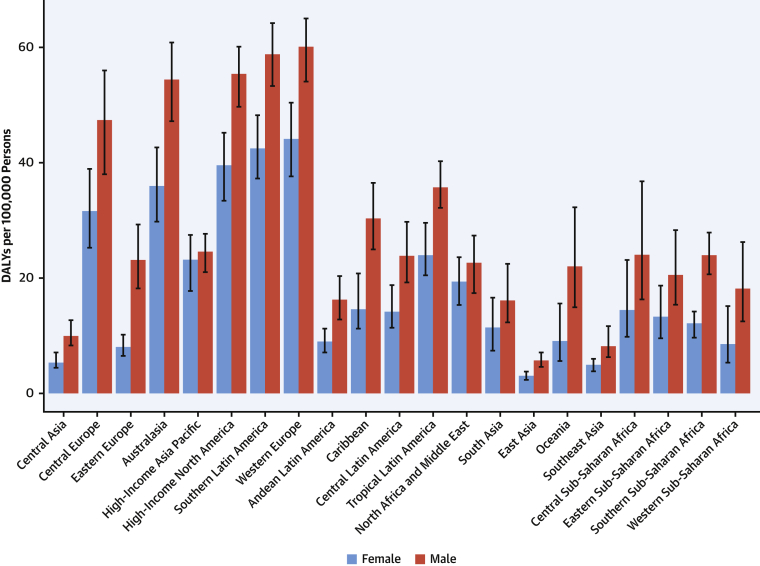
Age-Standardized DALYs Due to Nonrheumatic Calcific Aortic Valve Disease in 2019 by Region Age-standardized DALY rate of nonrheumatic calcific aortic valve disease by region and sex with 95% uncertainty intervals, 2019. DALYs = disability-adjusted life years.

There is substantial heterogeneity among countries in age-standardized prevalence and DALYs due to CAVD (Figure 11). Prevalence is low (<20 per 100,000) in several regions of the world, notably, sub-Saharan Africa (excluding the south), most of Asia (including South and Southeast Asia), North Africa and the Middle East, and Oceania. In contrast, age-standardized prevalence is much higher (>200 per 100,000) in Australasia; high-income Asia Pacific and North America; and Central, Eastern, and Western Europe. The difference may reflect higher prevalence of other competing illnesses and lack of testing for CAVD in LMICs. Despite the low prevalence of CAVD in LMICs, DALYs due to CAVD are disproportionately high, possibly reflecting the limited access to timely aortic valve replacement (28).

As populations age, CAVD becomes an increasingly important cause of morbidity and mortality. The increase in reported prevalence of CAVD may be a consequence of both an increase in the prevalence of risk factors for atherosclerosis and an increased awareness and investigation to detect disease. Although major advances have been made in the treatment of end-stage CAVD, research on ways to prevent the onset and progression of disease requires additional investments. In the populous regions of the world such as South Asia and Africa, despite the low prevalence of CAVD, the absolute number of people with CAVD is large. Countries will need to invest in health care infrastructure and health workers to provide timely valve interventions for these patients to reduce related morbidity and mortality.

### Nonrheumatic valvular heart disease: degenerative mitral valve disease

The major cause of nonrheumatic degenerative mitral valve (MV) disease is MV prolapse (29,30). Untreated, this can lead to chronic mitral regurgitation, AF, and heart failure (31). The total number of DALYs due to degenerative MV disease has increased since 1990 and is responsible for 883,000 DALYs (95% UI: 754,000 to 1,090,000 DALYs) and 34,200 deaths (95% UI: 28,300 to 43,300 deaths) in 2019 (Supplemental Figure 28A). GBD 2019 estimated 24.2 million (95% UI: 23.1 to 25.4 million) prevalent cases of degenerative MV disease in 2019.

Age-standardized rates for DALYs, deaths, and prevalent cases of degenerative MV disease declined over this time period, suggesting that the global increases in MV disease have been due to population growth and aging (Supplemental Figure 28B). The regions with the largest reductions in age-standardized DALYs between 1990 and 2019 are high-income Asia Pacific, North Africa and the Middle East, and Australasia. However, for some regions—for example, in Eastern Europe and Central Asia—age-standardized rates have not declined.

**Figure 12:**
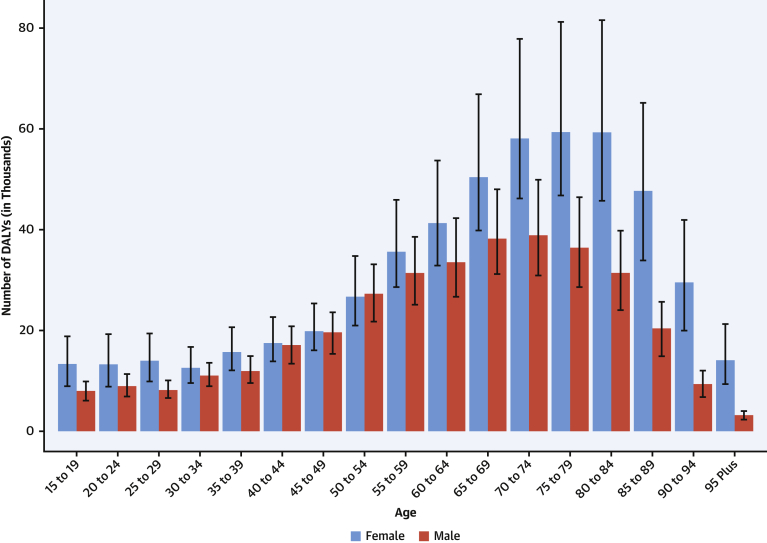
DALYs Due to Nonrheumatic Degenerative Mitral Valve Disease in 2019 by Age Number of DALYs due to nonrheumatic degenerative mitral valve disease by age and sex with 95% uncertainty intervals, 2019. Ages younger than 15 years were removed from the figure because they are not modeled for this cause. DALYs = disability-adjusted life years.

At the global level, substantially more total DALYs due to MV disease were experienced by women than men. For almost all age groups, MV disease DALYs were higher in women than men (Figure 12). Globally, women and men had similar DALYs in the 40- to 44-year age groups, but the levels diverged after age 65 years, when women had more than one-third more DALYs due to MV disease than men, peaking at age 75 to 79 years. Prior studies have found that women with severe regurgitation have higher mortality and lower surgery rates than men, which may account for some of the increased DALYs in older women (32).

Age-standardized DALYs due to MV disease were highest for both men and women in Central Europe; specifically, high rates were seen in Serbia, Bosnia and Herzegovina, and Hungary, with the lowest levels seen in Southeast Asia, East Asia, and Andean Latin America (Supplemental Figure 29). Women had higher DALYs than men in most regions except Central Asia, Eastern Europe, Australasia, the Caribbean, and Western sub-Saharan Africa (Supplemental Figure 30).

Degenerative MV disease continues to be a major threat to public health, and the overall burden in terms of number of DALYs, deaths, and prevalent cases is increasing globally. Prevention of MV disease through reduction in risk factors remains a key public health issue. In addition, given an aging population in many regions, health systems should focus on improving access to diagnostic imaging (i.e., echocardiography) and subspecialty care for close follow-up. Improved access to surgical and percutaneous interventions is also needed to decrease mortality and long-term sequelae and improve quality of life for those with MV disease (33).

### Endocarditis

**Figure 13:**
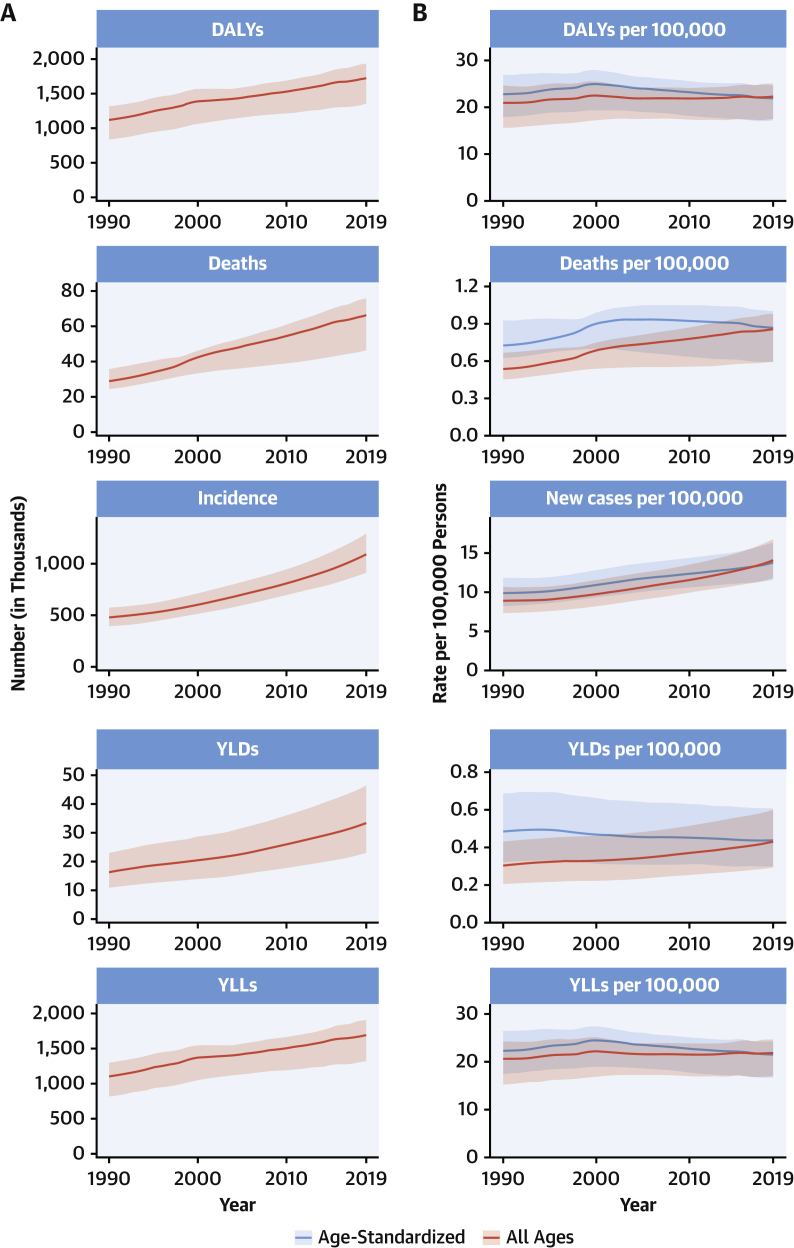
Total Numbers and Rates of Endocarditis **(A)** Total number of DALYs, deaths, incident cases, YLDs, and YLLs due to endocarditis, 1990 to 2019. **Shaded regions** represent 95% uncertainty intervals. **(B)** Age-standardized and all-ages DALY, death, incidence, YLD, and YLL rates of endocarditis, 1990 to 2019. **Shaded regions** represent 95% uncertainty intervals. Abbreviations as in Figure 1.

The total number of DALYs due to endocarditis has risen steadily since 1990, reaching 1.72 million (95% UI: 1.36 to 1.94 million) DALYs and 66,300 deaths (95% UI: 46,200 to 75,900 deaths) in 2019 (Figure 13A). GBD 2019 estimated 1.09 million (95% UI: 0.913 to 1.30 million) incident cases of endocarditis in 2019.

Age-standardized rates show an increase in incidence, from 9.9 per 100,000 (95% UI: 8.2 to 11.8 per 100,000) to 13.8 per 100,000 (95% UI: 11.6 to 16.3 per 100,000), as well as deaths, from 0.7 per 100,000 (95% UI: 0.6 to 0.9 per 100,000) to 0.9 per 100,000 (95% UI: 0.6 to 1.0 per 100,000) (Figure 13B). Although there has been a slight increase in the DALYs, YLLs, and YLDs caused by endocarditis across all ages, once standardized for age structure and growth, these metrics are largely static between 1990 and 2019. The increase in death may be explained by an increasing proportion of endocarditis caused by virulent organisms such as staphylococci or complex infection in patients not fit for surgical intervention.

At the global level, more total DALYs due to endocarditis were experienced by men than women in 2019 (973,000 DALYs [95% UI: 736,000 to 1,120,000 DALYs] versus 751,000 DALYs [95% UI: 537,000 to 871,000 DALYs]). DALYs from endocarditis rose rapidly for both sexes from birth and reached a peak at age 55 to 59 years for men and age 65 to 69 years for women (Supplemental Figure 31). Women ages 75 years and older had more DALYs due to endocarditis than men in the same age group.

There is wide variation in the regional age-standardized DALY rate due to endocarditis (Supplemental Figures 32 and 33). In 2019, the highest rates were seen in Oceania, with 44.9 DALYs per 100,000 (95% UI: 30.7 to 60.7 DALYs per 100,000) (across men and women), followed by Southern Latin America (40.1 DALYs per 100,000 [95% UI: 32.7 to 49.6 DALYs per 100,000]) and Southeast Asia (38.6 DALYs per 100,000 [95% UI: 31.6 to 53.8 DALYs per 100,000]). There were also high rates in Eastern Europe (especially in men), the Caribbean, Tropical Latin America, and Africa. The lowest DALYs for endocarditis were seen in Central and East Asia. In large part, areas with high age-standardized DALY rates for endocarditis mirror those areas with a high prevalence of RHD, for example, in Oceania, Southeast Asia, and sub-Saharan Africa.

The epidemiology of endocarditis, including predisposing conditions, is heterogeneous. Therefore, it is not surprising that the endocarditis-associated YLDs and DALYs vary considerably by location. Both the endocarditis-associated YLDs and DALYs increased in 76.2% and 52.4% of locations, respectively. *Staphylococcus aureus* has become the predominant endocarditis pathogen in many areas of the world and is extremely virulent and a well-recognized marker of worse outcomes (34). As a result, mounting human and health care burdens mandate that an individualized approach be developed for each location to better understand the unique epidemiology of endocarditis so that efforts can be focused on management and prevention of this life-threatening condition.

### Peripheral artery disease

The global numbers of prevalent cases and deaths due to PAD have risen consistently each year since 1990, resulting in a 2-fold increase to 113 million cases (95% UI: 99.2 to 128 million cases) and 74,100 deaths (95% UI: 41,200 to 128,000 deaths) in 2019 (Supplemental Figure 34A). Comparable trends in DALYs, YLLs, and YLDs have resulted in 1.54 million (95% UI: 1.01 to 2.37 million), 1.04 million (95% UI: 0.604 to 1.78 million), and 501,000 (95% UI: 235,000 to 898,000) respectively in 2019.

In keeping with the increase in numbers of prevalent cases, deaths, DALYS, YLLs, and YLDs, rates per 100,000 persons of all ages increased between 1990 and 2019 (Supplemental Figure 34B). The trends in age-standardized rates of deaths and YLLs were flat from 1990 to 2019, and prevalence, YLDs, and DALYs declined slightly. Overall, the PAD prevalence increase reflects population growth rather than a major change in age-specific incidence. In both 1990 and 2019, cross-sectional analyses show that the numbers of prevalent cases increased with age in both men and women up to 70 years of age and prevalence rates increased throughout the whole age spectrum. Numbers and rates of cases were higher in women than men at all ages. Deaths and DALYs showed increases with age comparable to prevalence except that deaths and DALYs were higher in men than women up to very old age.

The numbers of prevalent cases increased in both men and women at all ages by up to 2-fold since 1990, but age-standardized prevalence rates were slightly lower in 2019 than in 1990. Numbers of deaths and DALYs were higher in 2019 compared to 1990 in both men and women at all ages, but over the same period, age-standardized death rates remained much the same, and DALY rates fell slightly.

**Figure 14:**
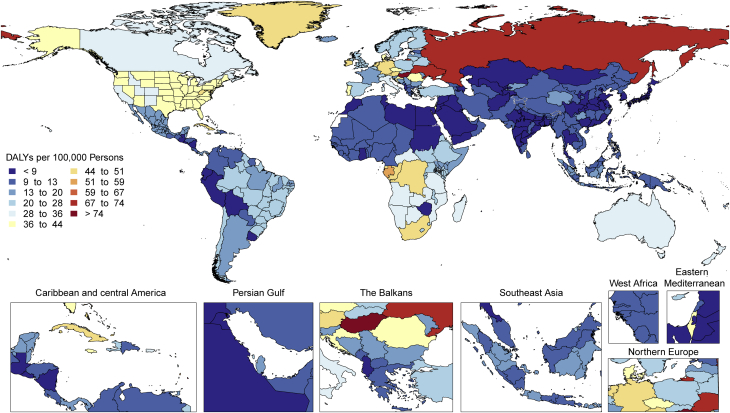
Map of Age-Standardized DALYs Due to Peripheral Artery Disease in 2019 DALYs = disability-adjusted life years.

Age-standardized DALY rates varied by world region in 2019 (Figure 14). Higher rates (>25 per 100,000 persons) were found in both men and women in Europe (western, central, and eastern), North America, Australasia, the Caribbean, and sub-Saharan Africa (central, eastern, and southern) (Supplemental Figure 35). Especially high rates occurred in men in Eastern Europe and areas of sub-Saharan Africa. Lowest DALY rates were found in Asia, Andean countries, West and North Africa and the Middle East. The pattern was similar in men and women, with most regions showing higher rates in men, but in regions with low overall rates, the sex differences were small. The burden of PAD is increasing not only in developed HICs but also in LMICs, where concomitant risk factors such as diabetes and obesity are increasing.

Despite decreases in PAD age-standardized prevalence, deaths, DALYs, YLDs, and YLLs, overall rates have increased because of worldwide increases in life expectancy. High DALY rates of PAD in Eastern Europe and parts of Africa, particularly in men, require investigation. PAD is highly prevalent and is both underrecognized and inadequately studied.

## Modifiable Risk Factors

### High systolic blood pressure

With aging of the population and population growth, the number of adults worldwide affected by high systolic blood pressure (SBP) increased from 2.18 billion (95% UI: 95% UI: 2.11 to 2.26 billion) to 4.06 billion (95% UI: 3.96 to 4.15 billion) from 1990 to 2019 (Supplemental Table 1). High SBP is defined by GBD 2019 according to a theoretical minimum risk exposure level (TMREL) of ≥110 to 115 mm Hg, which is the level of exposure that minimizes risk at the population level. In 2019, there were 828 million (95% UI: 768 to 888 million) adults with SBP >140 mm Hg, a common threshold for treatment with pharmacotherapies. The prevalence of adults worldwide with high SBP increased from 84,481.1 per 100,000 persons (95% UI: 81,641.5 to 87,579.2 per 100,000 persons) in 1990 to 88,971.1 per 100,000 persons (95% UI: 86,950.0 to 91,028.8 per 100,000 persons) in 2019.

From 1990 to 2019, the total number of DALYs due to high SBP increased from 154 million (95% UI: 139 to 169 million) to 235 million (95% UI: 211 to 261 million) (Supplemental Figure 36A). Additionally, between 1990 and 2019, the number of deaths (6.79 million [95% UI: 6.07 to 7.50 million] to 10.8 million [95% UI: 9.51 to 12.1 million]), YLDs (10.1 million [95% UI: 7.30 to 13.1 million] to 21.2 million [95% UI: 15.2 to 27.2 million]), and YLLs (144 million [95% UI: 129 to 158 million] to 214 million [95% UI: 191 to 237 million]) attributed to high SBP increased. The age-standardized rates of DALYs, deaths, and YLLs attributed to high SBP declined from 1990 to 2019, whereas the age-standardized rate of YLDs did not change substantially over this time period (Supplemental Figure 36B). These data indicate that the increases in total number of disease events can be attributed to population growth and aging. However, in some regions—for example, Oceania—the age-standardized rates of DALYs, deaths, YLDs, and YLLs increased between 1990 and 2019.

**Figure 15:**
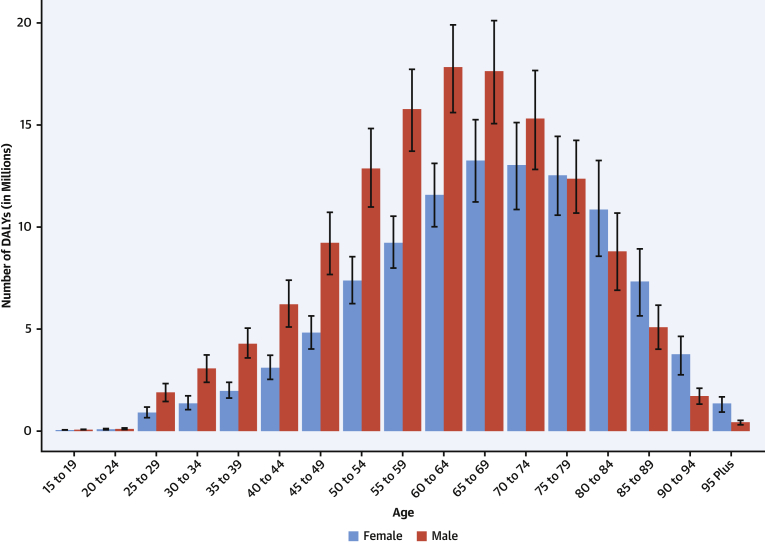
DALYs Due to High Systolic Blood Pressure in 2019 by Age Number of DALYs due to high systolic blood pressure by age and sex with 95% uncertainty intervals, 2019. Ages younger than 15 years were removed from the figure because they are not modeled for this risk. DALYs = disability-adjusted life years.

Globally, DALYs due to high SBP in 2019 were higher among men compared with women from ages 15 to 19 through 70 to 74 years, but higher among women compared to men in those 80 to 84 years or older (Figure 15). Age-standardized DALYs were higher among men compared with women in all regions of the world (Supplemental Figure 37).

In 2019, there was an approximately 8-fold variation across regions in age-standardized DALYs attributed to high SBP (Supplemental Figure 38). Rates of DALYs were lowest for both women and men in the high-income Asia Pacific region and highest in Central Asia. Marked variation was also noted for rates of death, YLLs, and YLDs. The highest age-standardized rates of death and YLLs were noted in Central Asia and Eastern Europe among men and in Central Asia, North Africa and the Middle East, and Central sub-Saharan Africa among women. The highest rate of YLDs was noted in Central Asia, Central Europe, and Southeast Asia for men and in North Africa and the Middle East, East Asia, and Southeast Asia for women.

High SBP is a major public health challenge, affecting approximately 9 of 10 adults worldwide, and is associated with high rates of DALYs, death, YLDs, and YLLs. High SBP and its adverse health consequences can be prevented by eating a heart-healthy diet that includes <1 teaspoon of salt per day and adequate potassium from fruits and vegetables, maintaining a normal weight, increasing physical activity, and avoiding unhealthy alcohol intake (35).

### High fasting plasma glucose

There are large differences between and within countries in the burden of high fasting plasma glucose, defined as above the TMREL of 4.8 to 5.4 mmol/l. The findings showed little improvement in age-standardized mortality rates from high fasting plasma glucose and significant increases in age-standardized YLDs between 1990 and 2019. Age-standardized mortality due to high fasting plasma glucose actually increased from 1990 to 2005, with a downward trend thereafter.

**Figure 16:**
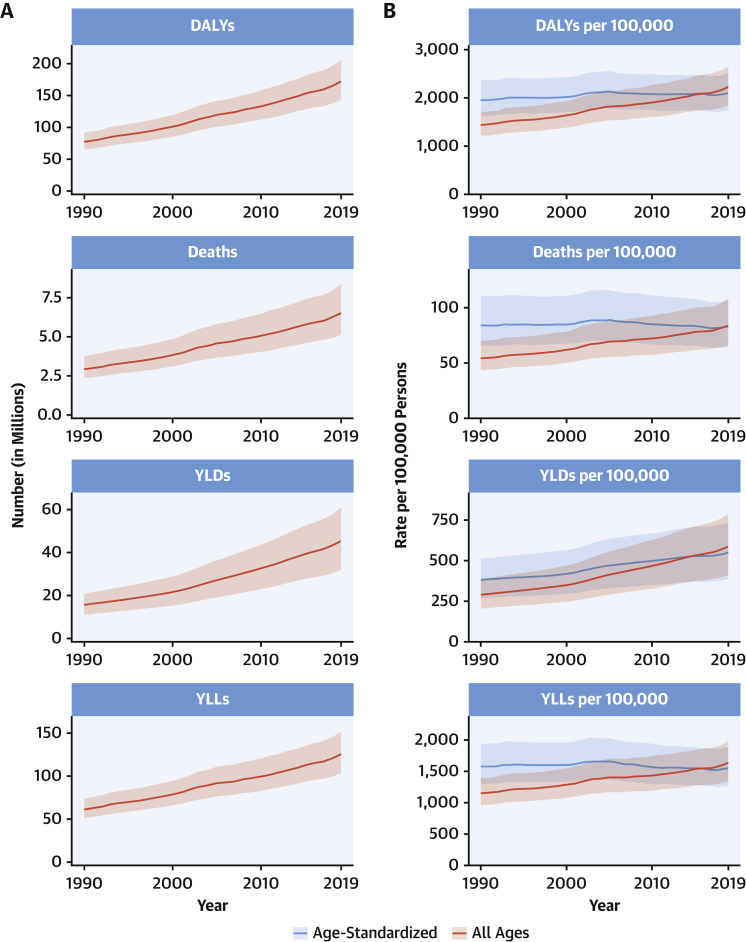
Total Numbers and Rates of High Fasting Plasma Glucose **(A)** Total number of DALYs, deaths, YLDs, and YLLs due to high fasting plasma glucose, 1990 to 2019. **Shaded regions** represent 95% uncertainty intervals. **(B)** Age-standardized and all-ages DALY, death, YLD, and YLL rates of high fasting plasma glucose, 1990 to 2019. **Shaded regions** represent 95% uncertainty intervals. Abbreviations as in Figure 1.

Between 1990 and 2019, a total of 134 million (95% UI: 107 to 171 million) deaths due to high fasting plasma glucose were recorded globally. The number of deaths increased from 2.91 million (95% UI: 2.34 to 3.75 million) in 1990 to 6.50 million (95% UI: 5.11 to 8.36 million) in 2019 (Figure 16A). The age-standardized mortality rate due to high fasting plasma glucose increased between 1990 and 2005 from 84.2 per 100,000 deaths (95% UI: 65.9 to 111.1 per 100,000 deaths) to 89.2 per 100,000 deaths (95% UI: 70.2 to 115.9 per 100,000 deaths) then declined to 83.0 per 100,000 deaths (95% UI: 64.5 to 107.1 per 100,000 deaths) in 2019 (Figure 16B). Age-standardized DALYs followed the same patterns with a peak in 2005 and then a decline until 2019. In contrast, age-standardized DALY rates were higher in 2019 at 2,104.3 per 100,000 (95% UI: 1,740.7 to 2,520.7 per 100,000) than in 1990 at 1,959.6 per 100,000 (95% UI: 1,638.7 to 2,362.4 per 100,000). Of course, this is the result of a sharp increase in age-standardized YLDs from 1990 to 2019. Men had higher DALY burden than women throughout the study period until the age of 80 years, after which women had a higher number of DALYs (Supplemental Figure 39). DALYs due to high fasting plasma glucose increased with age, peaked at 70 years, and then declined.

Age-standardized DALYs due to high fasting plasma glucose were higher in Oceania, followed by Central Asia (Supplemental Figure 40). There were large variations between countries in high fasting plasma glucose DALY burden, with notably high rates in Uzbekistan, Afghanistan, Papua New Guinea, Egypt, and Oman (Supplemental Figure 41). The highest age-standardized DALYs in 2019 were observed in Kiribati at 12,255.2 per 100,000 (95% UI: 9,799.7 to 15,073.0 per 100,000), and the highest age-standardized death rates were also observed in Kiribati at 435.4 per 100,000 (95% UI: 347.0 to 540.5 per 100,000). Large variations were also observed within countries. For example, the age-standardized DALY rate varied in Brazil from 1,571.8 per 100,000 (95% UI: 1,293.6 to 1,909.1 per 100,000) in Minas Gerais to 3,286.5 per 100,000 (95% UI: 2,735.5 to 3,907.3 per 100,000) in Alagoas, a 70.6% difference in the country.

These population-based estimates of plasma glucose level help track the global rise in diabetes. GBD 2019 showed low rates of physical activity (LPA) and poor diet in many countries. Both are major risk factors for high fasting plasma glucose and have led to caloric imbalance and higher rates of obesity, which has rapidly increased globally since 1990 and affects most geographic areas. There is a need to develop and implement both public health policies and clinical programs to reduce health disparities and disease burden due to diabetes.

### High low-density lipoprotein cholesterol

The total number of DALYs due to high low-density lipoprotein (LDL) cholesterol, defined by a TMREL of 0.7 to 1.3 mmol/l, has risen steadily since 1990, reaching 98.6 million (95% UI: 80.3 to 119 million) DALYs and 4.40 million (95% UI: 3.30 to 5.65 million) deaths in 2019 (Supplemental Figure 42A). Over the same period, the YLDs and YLLs rose to 5.71 million (95% UI: 3.68 to 8.27 million) and 92.9 million (95% UI: 75.6 to 111 million), respectively. This indicates that the direction of the global trend for high LDL is increasing.

Over the study period, the global all-age rates for DALYs, deaths, and YLLs remained relatively static and increased for YLDs (Supplemental Figure 42B). During this time, the age-standardized rates for the same measures all declined. This difference between all-age rates and age-standardized rates suggests that although the global burden of LDL-related disease remains unacceptably high, on average, the observed increases in the burden of DALYs in locations like China and India have been driven primarily by population growth and aging. This trend is important for global health because it implies that there has been at least modest progress in reducing the burden of LDL-related disease globally. Age-standardized DALY rates attributable to elevated LDL cholesterol, however, are estimated to be increasing in some locations, including Pakistan and Saudi Arabia.

At the global level, significantly more total DALYs from elevated LDL were experienced by men than women. LDL-related DALYs increased rapidly for men beginning at age 30 years (Supplemental Figure 43). Men ages 40 to 44 years had as many DALYs due to LDL as women ages 60 to 64 and 80 to 84 years and more than women in all other age groups. In aging post-menopausal women, the LDL cholesterol levels are comparable to those of men of similar age. The difference in DALYs by sex and age suggests that the burden of premature LDL-related disease in men younger than 65 years may need focused public health attention.

**Figure 17:**
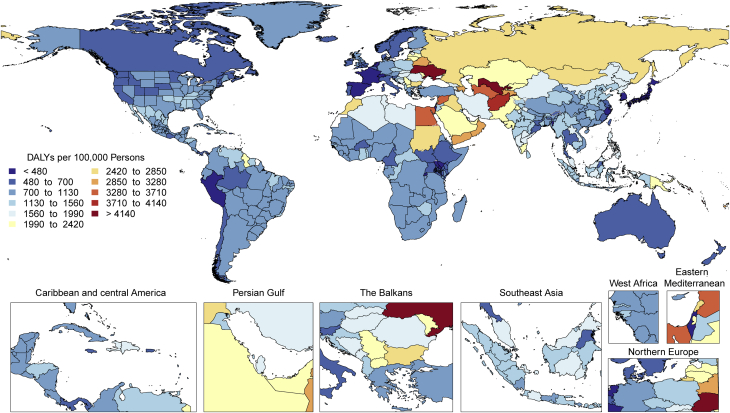
Map of Age-Standardized DALYs Due to High LDL Cholesterol in 2019 DALYs = disability-adjusted life years; LDL = low-density lipoprotein.

Age-standardized DALYs due to high LDL cholesterol were highest in Eastern Europe, North Africa and the Middle East, Oceania, and Central Asia (Supplemental Figure 44). The lowest levels are present in high-income Asia Pacific, Australasia, Western Europe, and Andean Latin America and, by country, in the Republic of Korea, Japan, Rwanda, France, Israel, and Spain (Figure 17). Potential explanations for these differences in regional patterns include the correspondingly LPA, increased high body mass index (BMI), dietary patterns, and increased tobacco use in the same regions and countries.

High LDL cholesterol remains a major threat to public health, and the overall burden in terms of number of DALYs, deaths, YLDs, and YLLs is increasing globally. In some locations, the risk associated with high LDL cholesterol is especially high and deserves immediate public health attention. Health systems and countries may need to focus on new approaches that can reverse these trends. These might include improved government policies on diet and tobacco, school physical activity programs, and, when needed, the use of lipid-lowering therapy in keeping with contemporary guidelines in which statins are the first choice. More global investment in research to address LDL cholesterol–related knowledge and therapeutic gaps is needed to address this persistent global health threat.

### High BMI

Obesity, defined by elevated BMI (≥30 kg/m^2^), has reached epidemic levels worldwide. Elevated BMI worsens most of the CVD risk factors, including adverse effects on blood pressure, blood sugar, lipids, and inflammation and has adverse effects on cardiac structure and function. Not surprisingly, hypertension, coronary heart disease, heart failure, and AF are increased with obesity (36).

**Figure 18:**
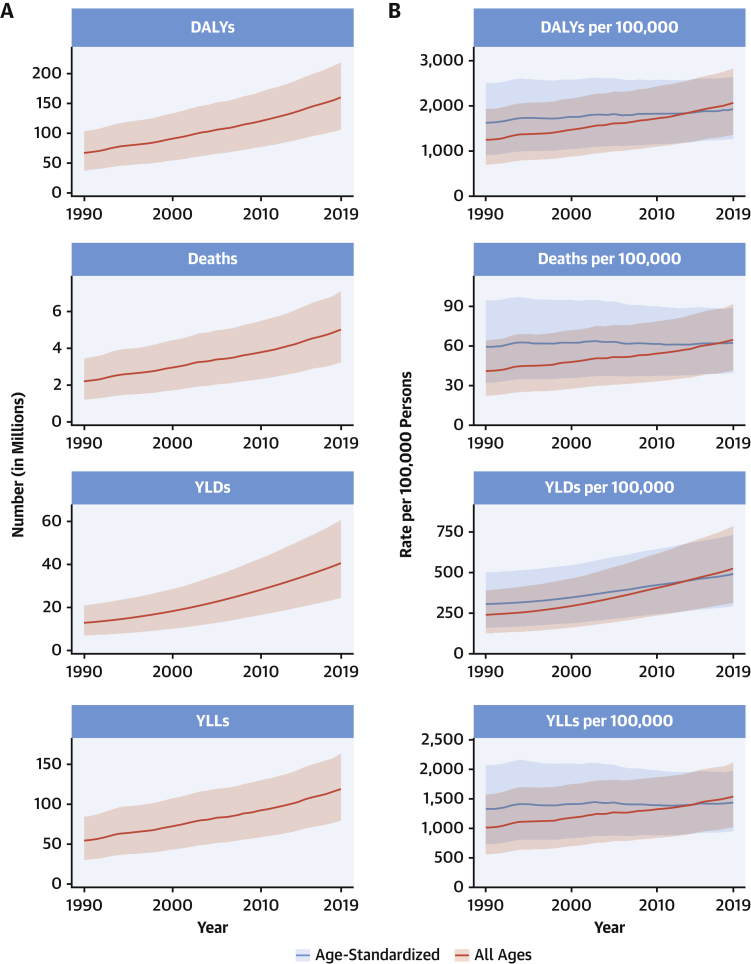
Total Numbers and Rates of High Body Mass Index **(A)** Total number of DALYs, deaths, YLDs, and YLLs due to high body mass index, 1990 to 2019. **Shaded regions** represent 95% uncertainty intervals. **(B)** Age-standardized and all-ages DALY, death, YLD, and YLL rates of high body mass index, 1990 to 2019. **Shaded regions** represent 95% uncertainty intervals. Abbreviations as in Figure 1.

Globally, 5.02 million (95% UI: 3.22 to 7.11 million) deaths and 160 million (95% UI: 106 to 219 million) DALYs were attributed to high BMI in 2019 (Figure 18A). High BMI, defined as above a TMREL of 20 to 25 kg/m^2^ for adults and above normal weight for children, contributed to more YLLs (119 million [95% UI: 79.6 to 164 million]) than YLDs (40.9 million [95% UI: 24.5 to 60.9 million]) in 2019. Globally, between 1990 and 2019, the absolute numbers of deaths attributable to high BMI increased (2.20 million [95% UI: 1.21 to 3.43 million] to 5.02 million [95% UI: 3.22 to 7.11 million]), as did DALYs (67.3 million [95% UI: 38.0 to 104 million] to 160 million [95% UI: 106 to 219 million]), YLDs (12.9 million [95% UI: 6.9 to 21.0 million] to 40.9 million [95% UI: 24.5 to 60.9 million]), and YLLs (54.4 million [95% UI: 30.2 to 84.4 million] to 119 million [95% UI: 79.6 to 164 million]).

After standardization for population growth and aging, rates of deaths attributable to high BMI have increased only modestly from 1990 to 2019 (4.9% increase [95% UI: –7.3% to 24.6%]), DALYs (18.0% [95% UI: 2.2% to 42.3%]), and YLLs (8.3% [95% UI: –6.6% to 31.2%]), suggesting that population growth and aging of the population have contributed substantially to the global trends in obesity-associated burden of disease (Figure 18B). YLDs have increased more sharply (60.2% [95% UI: 41.3% to 90.2%]) from 1990 to 2019. Since population growth and aging from 1990 account for much of the absolute increases attributable to high BMI, the focus may need to be on strategies targeting prevention of weight gain at younger ages to decrease the burden of all measures, including YLDs.

Global DALYs attributable to high BMI are highest between ages 45 and 75 years and are slightly higher for men at younger and middle ages and for women at older ages (Supplemental Figure 45).

Age-standardized DALY rates due to high BMI are highest in Oceania, Central Asia, North Africa and the Middle East, Southern sub-Saharan Africa, Eastern Europe, Central Latin America, the Caribbean, and Central Europe (Supplemental Figure 46). Among countries, Japan has the lowest and Kiribati the highest age-standardized DALYs in the world (Supplemental Figure 47). The global findings are generally similar between men and women in most regions, but age-standardized DALYs are notably higher for men in Central Asia and Central and Eastern Europe.

Great efforts are needed to promote the prevention of obesity and its progression to more severe forms (36). A multifactorial approach is required to promote improving dietary quality, especially reductions in simple sugars, complex carbohydrates, and total calories. Community prevention programs, like the Diabetes Prevention Program, duplicated in many programs (37), are needed (36). Optimizing efforts to promote physical activity and exercise training are needed, as well as reductions in sedentary behavior and making neighborhoods, communities, and workplaces more suitable to allow for these healthier lifestyle behaviors. Potential governmental involvement, such as mandating caloric/nutrition menu labeling and regulation of food ingredients, as well as controlling advertisements and certain forms of beverage and food item taxation, etc., could be instituted. Governmental intervention was very successful decades ago in the North Karelia Project in Finland (36,38), and potentially, similar efforts could be successful for obesity intervention worldwide. Research is needed to identify effective methods of health promotion that will reduce obesity and align all allied health professions toward this important goal (36).

### Impaired kidney function

GBD 2019 estimates use the term chronic kidney disease (CKD) to refer to the morbidity and mortality that can be directly attributed to all stages of CKD and the term impaired kidney function (IKF) to capture the additional risk of CKD from other associated conditions such as CVD and gout. The DALYs due to IKF nearly doubled from 1990 to 2019, reaching 76.5 million (95% UI: 67.8 to 86.3 million) DALYs and 3.16 million (95% UI: 2.72 to 3.62 million) deaths globally in 2019 (Supplemental Figure 48A). The estimated global prevalence of CKD is 9,011.9 per 100,000 (95% UI: 8,401.3 to 9,577.8 per 100,000) (697 million [95% UI: 650 to 741 million] people), 41.1% (95% UI: 38.9% to 43.4%) higher than in 1990 due to aging (Supplemental Figures 49A and 49B).

Age-standardized DALYs due to IKF declined slightly from 1,024.1 DALYs per 100,000 people (95% UI: 898.4 to 1,154.8 DALYs per 100,000 people) to 945.3 DALYs per 100,000 people (95% UI: 836.3 to 1,066.8 DALYs per 100,000 people) from 1990 to 2019 (Supplemental Figure 48B). This 7.7% (95% UI: –12.9% to –2.4%) decline in DALYs lags behind the 39.3% (95% UI: –43.5% to –34.9%) decline in DALYs for all risk factors combined. The age-standardized rate per 100,000 people for YLDs from IKF increased from 115.9 (95% UI: 84.7 to 150.5) to 139.4 (95% UI: 101.8 to 181.8) from 1990 to 2019. Growth and aging of the global population resulted in an increase in all unadjusted disease burden measures for IKF.

CKD and its consequences generally rise markedly with age and are higher among men than women, but trends over time were similar across age and sex groups (Supplemental Figures 50 and 51).

**Figure 19:**
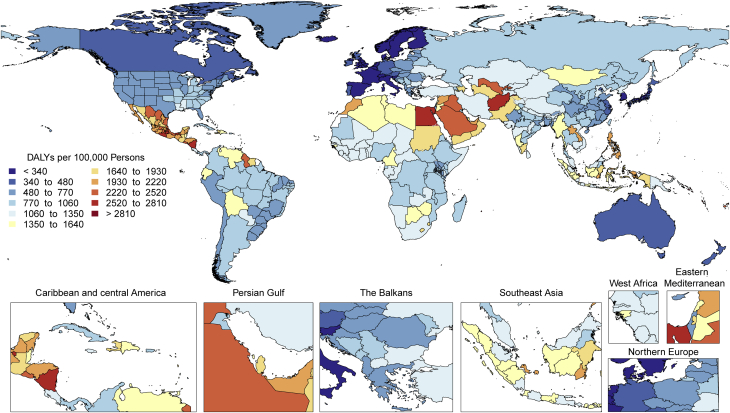
Map of Age-Standardized DALYs Due to Impaired Kidney Function in 2019 DALYs = disability-adjusted life years.

Geographically, the highest age-standardized rates of DALYs due to IKF are now seen in Central Latin America, Central Asia, the Caribbean, North Africa and the Middle East, Oceania, and Southeast Asia (Figure 19). Men in Central Latin America have among the highest age-standardized DALYs at 2,091.2 per 100,000 people (95% UI: 1,791.6 to 2,424.8 per 100,000 people), double the global rate and 60% higher than in 1990 (Supplemental Figure 52), which represents an epidemic of CKD of unknown origin that appears to be common in coastal lowlands, with repeated heat stress, pesticide, and heavy metal exposure implicated as potential causes (39).

CKD is emerging as a growing global problem, with marked growth related to the aging population. Age-standardized DALYs for IKF have declined by only 7.7%, compared to 39.3% for all risk factors, and they increased in Central and Andean Latin America, the Caribbean, Central and Southeast Asia, Oceania, and Southern sub-Saharan Africa. Much of CKD remains undiagnosed and undertreated. Governments, health systems, physicians, and patient attention are needed to improve the diagnosis of CKD by measuring proteinuria and estimated glomerular filtration rate. There is also a need for earlier markers of kidney injury. Diabetes and hypertension are leading factors for CKD, its progression, and associated risk. Improving their prevention and control will reduce the burden of CKD. Therapeutic options for CKD are increasing with the proven benefits of renin-angiotensin system blockade in proteinuria and possibly sodium-glucose cotransporter-2 inhibitors and mineralocorticoid receptor antagonists. Trials are underway to test prevention of kidney events in patients with CKD without diabetes. A combination of population-based public health measures for increased CKD recognition and awareness and improved therapies to reduce risk is needed to reduce the burden of CKD.

### Ambient and household air pollution

Air pollution is the leading environmental risk factor for global health and the fourth largest risk factor for global mortality. Two main forms of air pollution quantified in GBD 2019 contribute substantially to the burden of CVD: ambient particulate matter with an aerodynamic diameter smaller than 2.5 μm (PM_2.5_) and household air pollution (HAP) from the use of solid fuels for cooking. In 2019, CVD accounted for 51.5% and 30.5% of the total DALYS attributable to PM_2.5_ and HAP, respectively.

**Figure 20:**
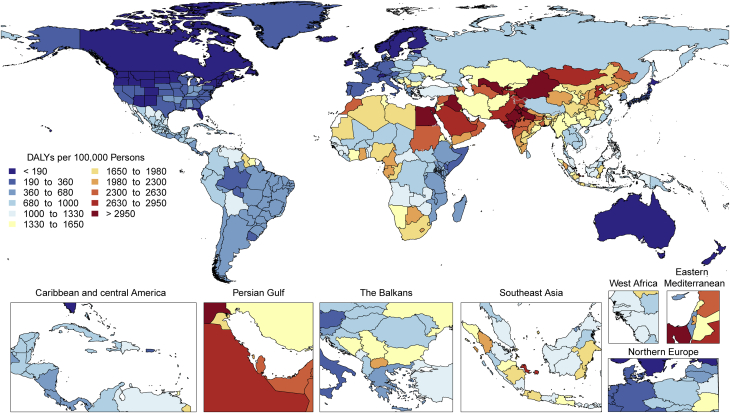
Map of Age-Standardized DALYs Due to Ambient Particulate Matter Pollution in 2019 DALYs = disability-adjusted life years.

Despite substantial reductions in ambient PM_2.5_ concentrations in North America and Europe, levels remain high throughout most of the world (40). Global annual average population-weighted PM_2.5_ levels increased slightly from 40.8 μg/m^3^ in 1990 to 42.6 μg/m^3^ in 2019. However, the global disease burden attributable to ambient PM_2.5_ increased from 70.5 million (95% UI: 47.3 to 98.9 million) DALYs and 2.05 million (95% UI: 1.45 to 2.74 million) deaths in 1990 to 118 million (95% UI: 95.9 to 138 million) DALYs and 4.14 million (95% UI: 3.45 to 4.80 million) deaths, respectively, in 2019 (Supplemental Figure 53A). Age-standardized rates for DALYs and deaths attributable to PM_2.5_ have remained relatively constant between 1990 and 2019, indicating that, on average, global increases in attributable disease have been due to population growth and aging, especially in locations with high exposures (Supplemental Figure 53B). Age-standardized DALYs attributable to PM_2.5_ were higher than the global mean in East, Central, and South Asia; North Africa and the Middle East; and Southern and Western sub-Saharan Africa (Figure 20).

In contrast to ambient PM_2.5_, the burden attributable to HAP decreased sharply from 208 million (95% UI: 162 to 259 million) DALYs and 4.36 million (95% UI: 3.33 to 5.40 million) deaths in 1990 to 91.5 million (95% UI: 67.0 to 119 million) DALYs and 2.31 million (95% UI: 1.63 to 3.12 million) deaths in 2019 (Supplemental Figure 54A). Trends for both all-age and age-standardized DALYs and death rates attributable to HAP have also declined sharply, indicating the impact of exposure reduction (Supplemental Figure 54B). However, age-standardized rates of DALYs attributable to HAP remain high throughout much of sub-Saharan Africa, which has yet to experience the benefits of improved access to clean household energy sources seen in India, China, and much of Southeast Asia (Supplemental Figure 55). The highest rates of DALYs for HAP were in Oceania; Eastern, Western and Central sub-Saharan Africa; and South Asia, driven by differences in exposure.

DALYs attributable to PM_2.5_ and HAP were higher in men, driven by sex differences in rates of the diseases affected by air pollution (Supplemental Figures 56 and 57). These sex differences were less pronounced for HAP given that women tend to be more highly exposed. DALYs attributable to HAP had the largest impacts in infancy and early childhood, whereas for PM_2.5_, it was between ages 30 and 90 years.

Increased attention to air pollution as a CVD risk factor is warranted and will require concerted action and collaboration between government health and environmental agencies. This is especially relevant given high levels of exposure in many parts of the world and links between PM_2.5_ and health risks at levels substantially below those of current regulatory levels (41). As demonstrated in North America and Western Europe, air quality management, coupled with regulation and enforcement, is effective at reducing ambient air pollution and its disease burden (42). Similar approaches applied elsewhere, in combination with actions to reduce emissions of climate-forcing agents will be necessary. In the interim, personal-level interventions, such as the use of portable air cleaners, may be effective (43). Continued reductions in HAP exposure through programs to improve access to clean energy sources will lead to both health and climate benefits (44).

### Tobacco

**Figure 21:**
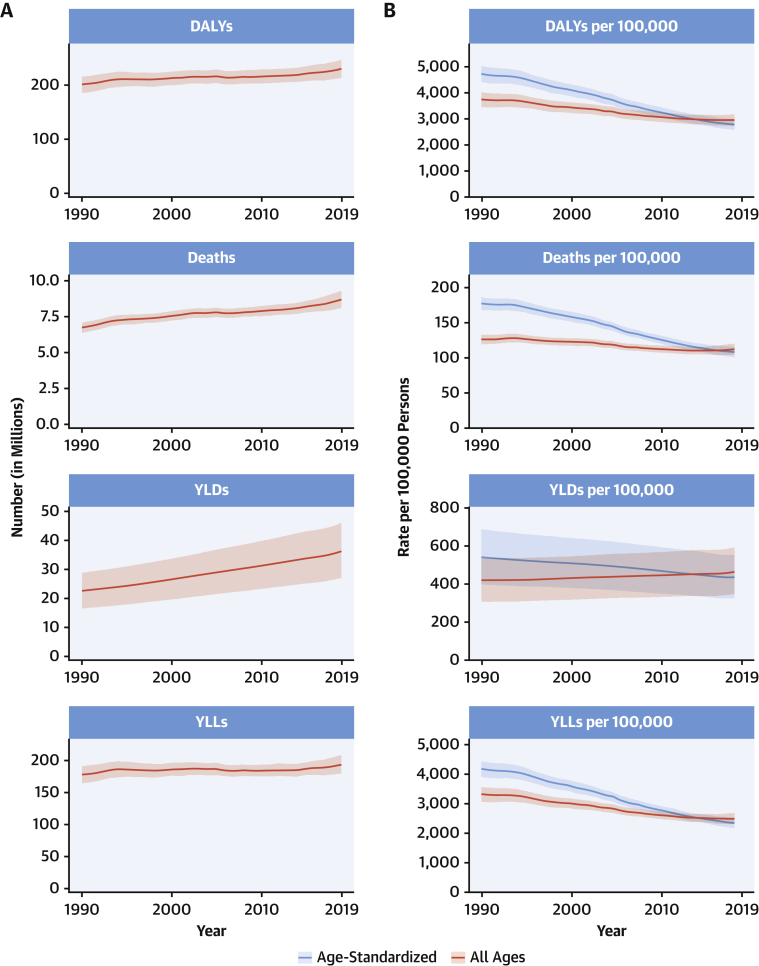
Total Numbers and Rates of Tobacco **(A)** Total number of DALYs, deaths, YLDs, and YLLs due to tobacco, 1990 to 2019. **Shaded regions** represent 95% uncertainty intervals. **(B)** Age-standardized and all-ages DALY, death, YLD, and YLL rates of tobacco, 1990 to 2019. **Shaded regions** represent 95% uncertainty intervals. Abbreviations as in Figure 1.

Tobacco, including primary smoking, secondhand smoke, and use of chewing tobacco, accounted for 8.71 million (95% UI: 8.12 to 9.31 million) deaths and 230 million (95% UI: 213 to 246 million) DALYs in 2019 (Figure 21A). Of tobacco-attributable deaths, 36.7% were due to CVD. The number of YLLs attributable to tobacco (194 million [95% UI: 180 to 209 million]) far exceeded the number of attributable YLDs (36.1 million [95% UI: 27.0 to 46.0 million]), highlighting the outsized effect of tobacco on premature mortality.

Progress in reducing tobacco use and exposure has not kept pace with competing demographic forces, namely, population growth and population aging. Population growth, particularly in countries with a large population of tobacco users, has resulted in an increasing number of tobacco-attributable deaths and DALYs since 1990. When controlling for population growth, decreases in both death and DALY rates are observed. However, population aging has resulted in smaller decreases in all-age rates compared to age-standardized rates (Figure 21B). With approximately 80% of current smokers living in LMICs, these demographic forces will continue to counter progress in tobacco control for years to come.

In 2019, global smoking prevalence among men was 33.5% (95% UI: 33.1% to 33.9%), and smoking prevalence among women was 6.8% (95% UI: 6.6% to 7.0%). As a result of this difference, 75.4% of smoking attributable deaths occurred among men. The age pattern of tobacco use and exposure, combined with the age distribution of associated health outcomes, results in age-specific DALYs peaking among men ages 60 to 64 years and among women ages 65 to 69 years (Supplemental Figure 58).

The highest age-standardized tobacco-attributable DALY rates among men were observed in Eastern Europe, Central Asia, and Oceania (Supplemental Figure 59). Among women, the highest rates were observed in Oceania, high-income North America, and Central Europe. Thirty percent of all current smokers live in China, and nearly one-third of tobacco-attributable deaths occurred in China in 2019.

With more than 1 billion active tobacco users, the tobacco epidemic remains a central challenge to global health. The global disease burden of tobacco use continues to increase, attributable in large part to population growth and falling most heavily on LMICs, where the large majority of tobacco users live. Effective tobacco control measures available to governments and private sector institutions include policies that increase taxes on tobacco, fund mass media education campaigns, ban tobacco marketing and sponsorship, require prominent warning labels on tobacco products, require environments to be smoke-free, and ensure access to and delivery of tobacco cessation treatments in health care systems. The evidence-based measures are included in the World Health Organization’s Framework Convention on Tobacco Control, a public health treaty that took effect in 2005 and has been ratified by 182 countries. Despite this global commitment, adoption of the Framework Convention on Tobacco Control measures has varied widely across countries. Increasing adoption and effective implementation of evidence-based tobacco control policies is a key worldwide public health priority. Looking ahead toward non-communicable disease targets set in the Sustainable Development Goals, accelerated progress in implementing proven policies and interventions is necessary to substantially reduce tobacco-attributable burden.

### Dietary risks

In GBD 2019, dietary risks comprise the sum of adverse effects of diets in which 15 food types are either underconsumed (fruits, vegetables, legumes, whole grains, nuts and seeds, milk, fiber, calcium, omega-3 fatty acids from seafood, and polyunsaturated fatty acids) or overconsumed (red meat, processed meat, sugar-sweetened beverages, *trans*-fatty acids, and sodium).

**Figure 22:**
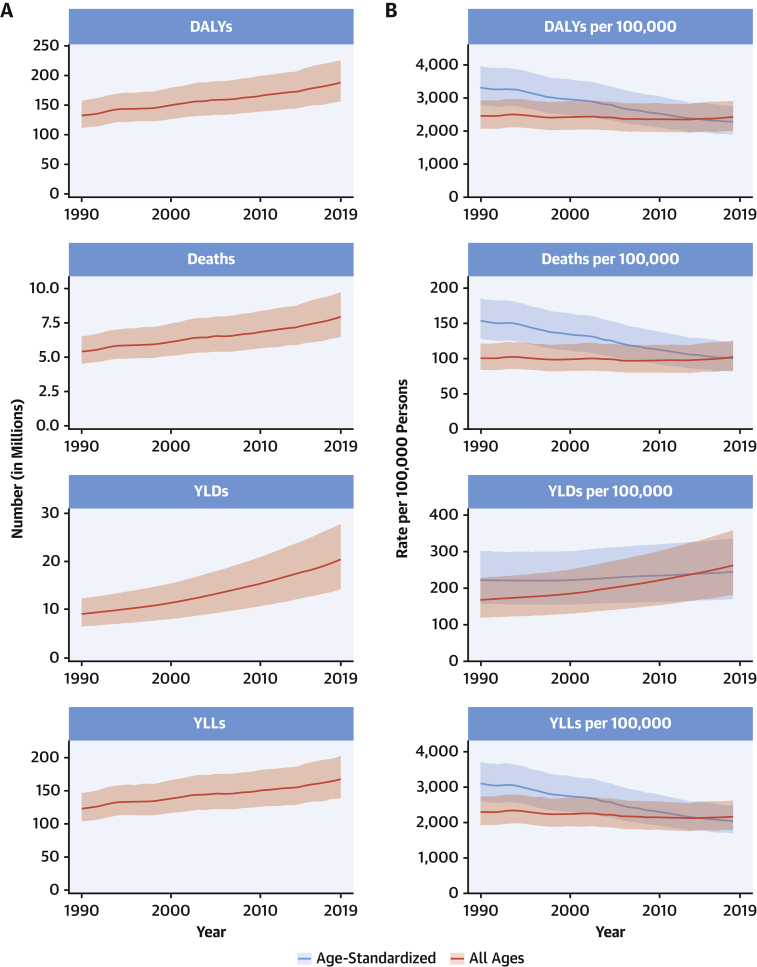
Total Numbers and Rates of Dietary Risks **(A)** Total number of DALYs, deaths, YLDs, and YLLs due to dietary risks, 1990 to 2019. **Shaded regions** represent 95% uncertainty intervals. **(B)** Age-standardized and all-ages DALY, death, YLD, and YLL rates of dietary risks, 1990 to 2019. **Shaded regions** represent 95% uncertainty intervals. Abbreviations as in Figure 1.

CVDs are the primary consequence of these dietary risks, with 7.94 million (95% UI: 6.47 to 9.76 million) annual deaths and 188 million (95% UI: 156 to 225 million) annual DALYs attributed to dietary risks (Figure 22A). Worldwide, the absolute disease burden caused by dietary risks has risen for 30 years, regardless of how measured. Over the same period, age-standardized rates have fallen for most measures, indicating the central role of population growth and population aging on the observed increases (Figure 22B). The exception is age-standardized rates of YLDs caused by dietary risks, which have grown in both absolute and proportional terms.

Worldwide, the number of DALYs caused by dietary risks peaks between 55 and 70 years of age for both men and women, with a large excess of DALYs among men compared to women in every age group to 80 years old (Supplemental Figure 60). Age-standardized rates of DALYs caused by dietary risks are highest in Central Asia, Oceania, and Eastern Europe and lowest in Australasia, HICs of the Asia Pacific, and Western Europe (Supplemental Figures 61 and 62). Differences in age-standardized DALY rates among populations reflect a complex interplay between variations in diet quality, levels of exposure to different dietary risks, and access to interventions targeting the downstream clinical effects of CVDs.

The detailed effects of many dietary risks are poorly understood because assessments are hampered by the challenges of accurately quantifying exposures and separating the effects of each risk from those of important covariates. However, the effects of dietary risks on CVD remain large whether assessed as individual dietary factors or measures of overall diet quality, and there is little doubt about the importance of dietary risks as a cause of global disease burden.

Strong commercial interests driving the sales of unhealthy foods have made it challenging to persuade policy makers, clinicians, or community members to act decisively on diet quality. Easy access to healthy fruits, vegetables, and whole grains remains limited for much of the world. Many countries have adopted policies to limit consumption of added sugar, sodium, and harmful fats, but implementation has mostly been weak, and the impact on global health has been limited. Exceptions are regulatory and fiscal interventions such as soda taxes and beverage reformulation programs that have led to lower intakes of added sugars, proving the feasibility of determined intervention. Likewise, the removal of artificial *trans*-fat from the food supply in the United States has improved diet quality, although the amount of *trans*-fat in the global food supply remains high. Reliance on drug therapies targeting downstream metabolic risk factors caused by obesity may be inadequate. Ill health caused by dietary risks is anticipated to rise rapidly in LMICs over the coming years, and behavioral and policy interventions suited for implementation at scale in diverse settings around the world are urgently required.

### Low Physical Activity

LPA is an important risk factor for IHD, stroke, diabetes, breast and colon cancers, and other noncommunicable diseases. The combination of increased risk for important causes of mortality and significant levels of LPA in most countries has led to physical inactivity being characterized as a pandemic. LPA is an important contributor of premature mortality, morbidity, and DALYs for adults in most of the world.

Globally, the total number of DALYs due to LPA, defined as <3,000 to 4,500 metabolic equivalent minutes per week, continuously increased from 8.61 million (95% UI: 4.28 to 15.9 million) in 1990 to 15.7 million (95% UI: 8.52 to 28.6 million) in 2019 (Supplemental Figure 63A). A linear trend was observed in the numbers of deaths due to LPA, reaching 832,000 premature deaths (95% UI: 427,000 to 1,470,000 premature deaths) in 2019. During the same time period, the estimated YLLs grew steadily from 7.51 million (95% UI: 3.62 to 14.4 million) in 1990 to 12.7 million (95% UI: 6.58 to 23.8 million) YLLs in 2019.

Over time, the all-ages rate of DALYs, deaths, and YLLs due to LPA has increased, and the age-standardized rate has decreased (Supplemental Figure 63B). This pattern reflects increased burden due to growth and aging of the population, an effect that is removed by age standardization.

Total global DALYs due to LPA were similar for men and women until the 65- to 69-year age group, with men consistently higher (Supplemental Figure 64). This pattern reversed in the 70- to 74-year age group as DALYs for women surpassed those for men. At all ages beyond 75 years, women had more DALYs than men, presumably driven by greater survival among women. LPA DALYs rose steadily for both men and women beginning at age 25 years, peaking for both sexes in the 80- to 84-year age group and declining thereafter.

**Figure 23:**
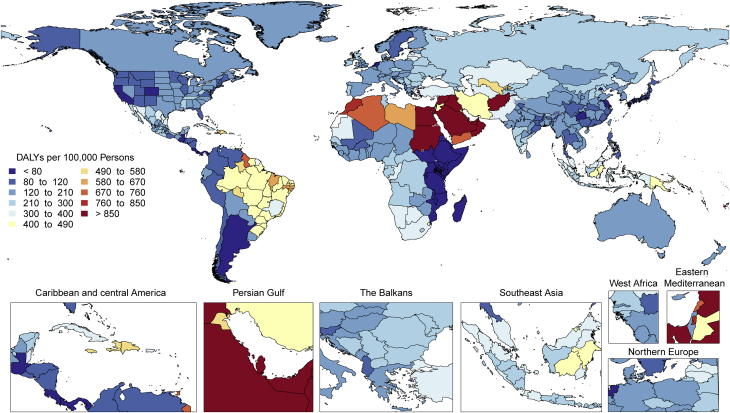
Map of Age-Standardized DALYs Due to Low Physical Activity in 2019 DALYs = disability-adjusted life years.

Age-standardized DALYs due to LPA were highly variable by both region and sex. The highest rates were seen for both men and women in North Africa and the Middle East, Oceania, tropical Latin America, and the Caribbean (Figure 23), with high rates for men also observed in Central Asia (Supplemental Figure 65). The lowest age-standardized DALYs for LPA were seen for both men and women in high-income Asia Pacific, Southern Latin America, and Eastern sub−Saharan Africa; for men in high-income North America; and for women in Andean Latin America.

LPA remains a major threat to public health, and the overall burden is increasing globally even as age-standardized rates are declining. Although few health systems have historically focused on LPA, there is increased awareness and attention being paid to LPA as exemplified by the 2018 World Health Organization Global Action Plan for Physical Activity. However, the factors that influence population physical activity largely reside outside of the health sector. Increasing physical activity will depend on effective partnerships and collaboration between health and sectors such as transportation, education, urban planning, workplace health strategies, and environmental protection.

## Subnational Disease Burden

**Figure 24:**
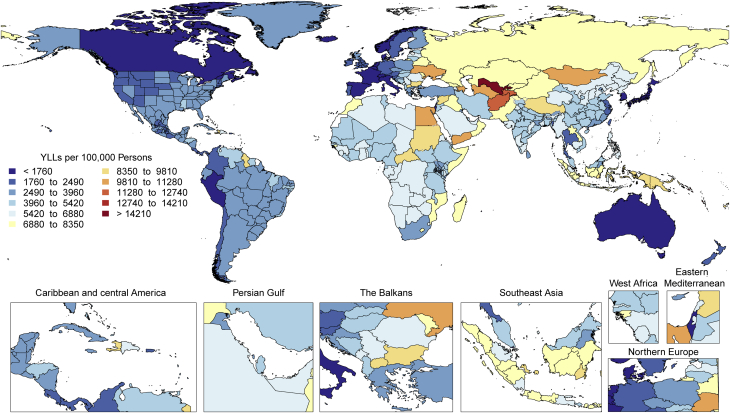
Map of Age-Standardized YLLs Due to Cardiovascular Diseases in 2019 YLLs = years of life lost.

GBD 2019 estimates disease burden within countries as well as between countries. Figure 24 shows the age-standardized rate of YLLs due to CVD for all countries, with Brazil, China, India, Indonesia, Japan, Kenya, Mexico, the United States, and the United Kingdom shown at the first subnational administrative level and Stockholm County shown separately from the rest of Sweden. Subnational disease estimation is made possible through collaboration with these governments. Subnational data reveal wide differences in DALYs within countries. For example, in Kenya in 2019, IHD DALYs were estimated to vary from 342.7 per 100,000 (95% UI: 212.4 to 531.7 per 100,000) in Turkana to 1,215.4 per 100,000 (95% UI: 856.8 to 1,612.4 per 100,000) in Nyeri, a 112.0% difference within Kenya. IHD DALYs in India ranged from 506.6 per 100,000 (95% UI: 379.6 to 819.0 per 100,000) in Mizoram to 5,413.2 per 100,000 (95% UI: 4,316.9 to 6,389.9 per 100,000) in Punjab, a 165.8% difference within the country. In Brazil, IHD DALYs varied from 771.2 per 100,000 (95% UI: 679.4 to 866.3 per 100,000) in Amazonas to 2,416.2 per 100,000 (95% UI: 2,176.7 to 2,686.2 per 100,000) in the Rio de Janeiro state, a 103.2% difference within the country. Within the United States, there was a 99.6% difference in IHD DALYs, ranging from 1,438.3 per 100,000 (95% UI: 1,210.7 to 1,699.0 per 100,000) in Utah to 4,293.1 per 100,000 (95% UI: 3,636.0 to 5,033.0 per 100,000) in West Virginia. In Mexico, they ranged from 890.5 per 100,000 (95% UI: 752.2 to 1,073.3 per 100,000) in Quintana Roo to 2,315.1 per 100,000 (95% UI: 1,932.1 to 2,716.3 per 100,000) in Chihuahua, an 88.9% difference within Mexico. Disease burden attributable to elevated levels of metabolic risk factors also varies significantly within countries.

Further analysis of the variation of CVD burden within countries will be vital to implementing health policies that reduce burden but also decrease disparities. The strategies to deal with these inequalities include: 1) targeting reduction in key modifiable risks including diet, tobacco, alcohol, physical activity, and obesity; 2) improved access and quality of health care; and 3) a renewed focus on the social determinants of health. Addressing risk factors may prove to be an important initial opportunity to reduce disparities in parallel with broader efforts targeting social determinants, with potential indirect benefits for improving those social determinants, such as reduced financial hardship due to fewer acute cardiac events among working-age adults.

Several risks are increasing globally and warrant increased policy and health system attention, including high BMI, blood pressure, unhealthy cholesterol, and poor diet. Targeting subnational locations where metabolic risks are rising rapidly may be an important approach for testing new interventions and delivering resources where they will have the biggest impact. There is often a gap between the identification of a problem, the location of a solution, and the translation of that solution to an entire population. We currently lack a systematic, scalable process for determining which interventions are most likely to succeed in a given location, and research funding should be directed at addressing this gap.

## Study limitations

Estimates from GBD 2019 should be interpreted in light of several limitations. Death certificates, although an essential source of data for public health, may be misclassified given the difficulty of identifying an underlying cause of death. GBD 2019 applies statistical methods to improve the comparability of health information coded using the ICD system and continuously evaluates, improves on, and reports the details of these methods. The use of different subnational population-based studies may lead to compositional bias of national estimates, although GBD 2019 adjusts variance and weighting to reflect this possibility. ICD system codes from administrative health facility data may represent a group of patients that differs from the standard case definition for a disease, although GBD 2019 applies adjustments to these data to account for differential access to health care and alternate case definitions. Access to CVD diagnostic technologies is likely to influence estimates of some conditions, with lower access resulting in lower observed prevalence. Some commonly used diagnostic tools—for example, electrocardiogram for AF—will underestimate true disease prevalence. Estimates are reported with an uncertainty interval that should always be carefully considered, given that it represents both data sparsity and differences in sample size across data sources and study locations. However, uncertainty may be underestimated in locations where no data on CVD of any kind are available. Limited data on CVD and resulting wider uncertainty intervals for parts of sub-Saharan Africa, Oceania, and Asia may restrict our ability to interpret trends in burden over time. An important use of GBD results is therefore to identify gaps in CVD health data and guide investments in future disease surveillance. In particular, better data are needed on the incidence of coronary events, cardiac arrest, and the severity and associated disability of chronic heart and vascular diseases. Comorbidity of CVD and the joint effects of multiple risk factors remains a topic where further investigation is also needed.

## Conclusions and Recommendations

CVDs remain the leading cause of disease burden in the world. CVD continues its decades-long rise for almost all countries outside the high-income world and, alarmingly, age-standardized CVD rates have begun to rise in many locations where they were previously declining. There remains a large gap between identifying CVD as a problem, identifying the best package of solutions, and delivering them to the entire population.

GBD 2019 continues to be a platform that allows tracking and benchmarking of progress in the reduction of CVD burden. However, large investments are still needed to improve disease surveillance, including the expansion of robust vital registration systems, reliable electronic health records, and health surveys to all countries.

High rates of excess mortality are currently being observed because of the global severe acute respiratory syndrome coronavirus 2 pandemic, and much of this additional disease burden may be CVD due to the effects of both viral infection but also the unintended consequence of social distancing behaviors, such as changes in the delivery of health care (45,46). Further research in this area is urgently needed.

CVD was the cause of 6.2 million deaths occurring between the ages of 30 and 70 years in 2019. There is a pressing need to focus on implementing existing cost-effective interventions and health policies if the world is to meet the targets for Sustainable Development Goal 3 and achieve at least a 30% reduction in premature mortality due to noncommunicable disease by 2030. In the face of a global viral pandemic, we still must emphasize global commitments to reduce the suffering and premature death caused by CVD, which limits healthy and sustainable development for every country in the world.

## Author Disclosures

This study was funded by the Bill and Melinda Gates Foundation. Dr. Benjamin has received funding from the National Institutes of Health (NIH)/National Heart, Lung, and Blood Institute (NHLBI) (R01HL092577, 1R01HL128914) and American Heart Association (18SFRN34110082). Dr. Brauer has received a grant from the Bill and Melinda Gates Foundation. Dr. Catapano has received support from Fondazione Cariplo 2015-0524 and 2015-0564, H2020 REPROGRAM PHC-03-2015/667837-2, ERA NET ER-2017-2364981, GR-2011-02346974, Ministry of Health - Ricerca Corrente Multimedica; has received research grant/support from Sanofi, Sanofi Regeneron, Amgen, Mylan, Menarini, and Eli Lilly; has served on the speakers bureau for Akcea, Amgen, Sanofi, Esperion, Kowa, Novartis, Ionis Pharmaceuticals, Mylan, Menarini, Merck, Recordati, Regeneron, Daiichi-Sankyo, AstraZeneca, Aegerion, Amryt, and Sandoz; has received honoraria from Akcea, Amgen, Sanofi, Esperion, Kowa, Novartis, Ionis Pharmaceuticals, Mylan, Menarini, Merck, Recordati, Regeneron Daiichi-Sankyo, AstraZeneca, Aegerion, Amryt, and Sandoz; and has served as a consultant/on the Advisory Board for Akcea, Amgen, Sanofi, Esperion, Kowa, Novartis, Ionis Pharmaceuticals, Mylan, Menarini, Merck, Recordati, Regeneron Daiichi-Sankyo, Genzyme, Aegerion, and Sandoz. Dr. Coresh has received funding from the NIH and National Kidney Foundation; and has served as an advisor to Healthy.io. Dr. Fowkes has served on the Advisory Board for AstraZeneca. Dr. Muntner has received grants and personal fees from Amgen Inc. Dr. Ribeiro has received partial support by CNPq (310679/2016-8 and 465518/2014-1) and by FAPEMIG (PPM-00428-17). Dr. Zuhlke has received funding by the UK Medical Research Council (MRC) and the UK Department for International Development (DFID) under the MRC/DFID Concordat agreement and the National Research Foundation of South Africa. Dr. Rigotti has served as a consultant to Achieve Life Sciences; and has received royalties from UpToDate, Inc. Dr. Rodgers is employed by The George Institute for Global Health (TGI) and seconded part time to George Medicines Pty Ltd (GM); TGI has submitted patent applications with respect to low-fixed-dose combination products for the treatment of cardiovascular or cardiometabolic disease and is listed as one of the inventors; George Health Enterprises Pty. Ltd. (GHE) and its subsidiary, GM, have received investment funds to develop fixed-dose combination products, including combinations of blood pressure-lowering drugs; GHE is the social enterprise arm of TGI (Dr. Rodgers does not have a direct financial interest in these patent applications or investments). Dr. Sundström holds ownership in companies providing services to Itrim, Amgen, Janssen, Novo Nordisk, Eli Lilly, Boehringer Ingelheim, Bayer, Pfizer, and AstraZeneca, outside the submitted work. All other authors have reported that they have no relationships relevant to the contents of this paper to disclose.
