# Correction

**DOI:** 10.1016/j.jacc.2021.02.039

**Published:** 2021-04-20

Roth GA, Mensah GA, Johnson CO, Addolorato G, Ammirati E, Baddour LM, Barengo NC, Beaton AZ, Benjamin EJ, Benziger CP, Bonny A, Brauer M, Brodmann M, Cahill TJ, Carapetis J, Catapano AL, Chugh SS, Cooper LT, Coresh J, Criqui M, DeCleene N, Eagle KA, Emmons-Bell S, Feigin VL, Fernández-Solà J, Fowkes G, Gakidou E, Grundy SM, He FJ, Howard G, Hu F, Inker L, Karthikeyan G, Kassebaum N, Koroshetz W, Lavie C, Lloyd-Jones D, Lu HS, Mirijello A, Temesgen AM, Mokdad A, Moran AE, Muntner P, Narula J, Neal B, Ntsekhe M, Moraes de Oliveira G, Otto C, Owolabi M, Pratt M, Rajagopalan S, Reitsma M, Ribeiro ALP, Rigotti N, Rodgers A, Sable C, Shakil S, Sliwa-Hahnle K, Stark B, Sundström J, Timpel P, Tleyjeh IM, Valgimigli M, Vos T, Whelton PK, Yacoub M, Zuhlke L, Murray C, Fuster V, for the GBD-NHLBI-JACC Global Burden of Cardiovascular Diseases Writing Group

**Global Burden of Cardiovascular Diseases and Risk Factors, 1990–2019: Update From the GBD 2019 Study**

**J Am Coll Cardiol 2020;76:2982–3021.**

On page 2994, the last sentence in the left column read:

Health systems in LMICs should support the recommendations of the 2018 World Health Assembly Global RHD Resolution with increased multisector investment in primary health care, improved sanitation and housing, infrastructure, secure medication supply chains, evidence-based RHD screening, prevention and management, and tertiary capacity to care for patients at the severe end of the RHD spectrum.

But should have read:

The 2018 World Health Assembly Global RHD Resolution recommends to increase multisector investment in primary health care, improve sanitation and housing, infrastructure, secure medication supply chains, evidence-based RHD screening, prevention and management, and tertiary capacity to care for patients at the severe end of the RHD spectrum.

On page 2996, the last sentence in the left column read:

Additional policy support for public education and awareness is needed to reduce the harmful use of alcohol. Because alcohol negatively affects cardiovascular function, increased clinician emphasis on eliminating alcohol use among people with coexisting CVD is advisable. Research should be aimed at understanding the exact mechanisms of disease, susceptibility to alcohol damage, effective public health interventions, and treatments for AC.

But should have read:

Additional support for public education and awareness can help to reduce the harmful use of alcohol. Since alcohol negatively impacts cardiovascular function, increased clinician emphasis on eliminating alcohol use among people with co-existing CVD is advisable. A deeper understanding of the exact mechanisms of disease, susceptibility to alcohol damage, effective public health interventions and treatments for AC, can optimize preventive measures.

On page 2999, the last sentence in the left column read:

As such, investing health system resources in these risk factors, including hypertension management and smoking cessation, could reduce the burden of other diseases in addition to aortic aneurysm. Combined with implementation of inexpensive screening technology, such as ultrasonography, where indicated, the morbidity and mortality due to aortic aneurysm can be significantly decreased globally.

But should have read:

As such, mitigating risk factors through, for example, hypertension management and smoking cessation, could reduce the burden of other diseases in addition to aortic aneurysm. Combined with effective and accessible screening technology, such as ultrasound, where indicated, the morbidity and mortality due to aortic aneurysm can be significantly decreased globally.

On page 3001, the last sentence in the second paragraph in the left column read:

Countries will need to invest in health care infrastructure and health workers to provide timely valve interventions for these patients to reduce related morbidity and mortality.

But should have read:

An improved health care infrastructure and an augmented healthcare workforce could provide timely valve interventions for these patients and thereby reduce related morbidity and mortality.

On page 3015, the third sentence in the third paragraph in the left column read:

Effective tobacco control measures available to governments and private sector institutions include policies that increase taxes on tobacco, fund mass media education campaigns, ban tobacco marketing and sponsorship, require prominent warning labels on tobacco products, require environments to be smoke-free, and ensure access to and delivery of tobacco cessation treatments in health care systems.

But should have read:

Tobacco control measures include increased taxes on tobacco, mass media education campaigns, bans on tobacco marketing and sponsorship, required prominent warning labels on tobacco products, required environments to be smoke-free, and ensuring access to and delivery of tobacco cessation treatments in health care systems.

On page 3015, the first sentence in the fourth paragraph in the right column read:

Strong commercial interests driving the sales of unhealthy foods have made it challenging to persuade policy makers, clinicians, or community members to act decisively on diet quality.

But should have read:

Strong commercial interests driving sales of unhealthy foods have made it challenging to make significant progress toward improving diet quality.

The authors apologize for these errors.

The online version of the article has been corrected to reflect these changes.

