# Supplementary material for: Global Burden of Cardiovascular Diseases and Risk Factors, 1990–2019: Update From the GBD 2019 Study
Source: J Am Coll Cardiol. 2020 Dec 22;76(25):2982–3021. doi: 10.1016/j.jacc.2020.11.010 (PMC7755038; doi:10.1016/j.jacc.2020.11.010)
Supplement: Supplemental Data 1 [file mmc1.pdf]

Supplementary Appendix to "Global Burden of Cardiovascular Diseases and Risk Factors, 1990-2019: Update from the Global Burden of Disease 2019 Study"

GBD-NHLBI-JACC Global Burden of Cardiovascular Diseases Writing Group

Gregory A. Roth, MD,[1,2,3] George A. Mensah, MD,[4,5] Catherine O. Johnson, PhD,[2] Giovanni Addolorato, MD,[6] Enrico Ammirati, MD, PhD,[7] Larry M. Baddour, MD,[8] Noel C. Barengo, PhD,[9] Andrea Beaton, MD,[10] Emelia J. Benjamin, MD, ScM,[11] Catherine P. Benziger, MD,[12] Aime Bonny, MD,[13,14] Michael Brauer, DSc,[15,2] Marianne Brodmann, MD,[16] Thomas J. Cahill, PhD,[17] Jonathan R. Carapetis, PhD,[18,19] Alberico L. Catapano, PhD,[20,21] Sumeet Chugh, MD,[22] Leslie T. Cooper, MD,[23] Josef Coresh, MD, PhD,[24] Michael H. Criqui, MD,[25] Nicole K. DeCleene, BS,[2] Kim A. Eagle, MD,[26] Sophia Emmons-Bell, BA,[2] Valery L. Feigin, PhD,[27,2,28] Joaquim Fernández-Sola, DrPH,[29,30] F Gerry R. Fowkes, PhD,[31] Emmanuela Gakidou, PhD,[2,3] Scott M. Grundy, MD,[32] Feng J. He, PhD,[33] George Howard, DrPH,[34] Frank Hu, MD, PhD,[35] Lesley Inker, MD,[36] Ganesan Karthikeyan, MD,[37] Nicholas J. Kassebaum, MD,[2,38] Walter J. Koroshetz, MD,[39] Carl Lavie, MD,[40] Donald Lloyd-Jones, MD,[41] Hong S. Lu, PhD,[42] Antonio Mirijello, MD,[43] Awoke T. Misganaw, PhD,[3,44] Ali H. Mokdad, PhD,[2,3] Andrew E. Moran, MD,[45,46] Paul Muntner, PhD,[47] Jagat Narula, MD,[48] Bruce Neal, PhD,[49,50] Mpiko Ntsekhe, PhD,[51,52] Gláucia M. M. Oliveira, PhD,[53] Catherine M. Otto, MD,[1] Mayowa O. Owolabi, DrM,[54,55] Michael Pratt, MD,[56] Sanjay Rajagopalan, MD,[57] Marissa B. Reitsma, BS,[2] Antonio Luiz P. Ribeiro, MD,[58,59] Nancy A. Rigotti, MD,[60,61] Anthony Rodgers, PhD,[62] Craig A. Sable, MD,[63] Saate S. Shakil, MD,[2,1] Karen Sliwa, PhD,[64] Benjamin A. Stark, MA,[2] Johan Sundström, PhD,[65,66] Patrick Timpel, MD,[67] Imad I. Tleyjeh, MD,[68,69] Marco Valgimigli, PhD,[70,71] Theo Vos, PhD,[2,3] Paul K. Whelton, MD,[72] Magdi Yacoub, MD,[73] Liesl J. Zuhlke, PhD,[74,5] Mohsen Abbasi-Kangevari, MD,[75] Alireza Abdi, PhD,[76] Aidin Abedi, MD,[77] Victor Aboyans, MD,[78,79] Woldu A. Abrha, MSc,[80] Eman Abu-Gharbieh, PhD,[81] Abdelrahman I. Abushouk, MD,[82,83] Dilaram Acharya, PhD,[84,85] Tim Adair, PhD,[86] Oladimeji M. Adebayo, MD,[87] Zanfina Ademi, PhD,[88] Shailesh M. Advani, PhD,[89,90] Khashayar Afshari, MD,[91,92] Ashkan Afshin, MD,[2,3] Gina Agarwal, PhD,[93] Pradyumna Agasthi, MD,[94] Sohail Ahmad, MSc,[95] Sepideh Ahmadi, PhD,[96] Muktar B. Ahmed, MPH,[97,98] Budi Aji, DrPH,[99] Yonas Akalu, MSc,[100] Wuraola Akande-Sholabi, PhD,[101] Addis Aklilu, MSc,[102] Chisom J. Akunna, DMD,[103,104] Fares Alahdab, MSc,[105] Ayman Al-Eyadhy, MD,[106] Khalid F. Alhabib, MD,[107] Sheikh M. Alif, PhD,[108] Vahid Alipour, PhD,[109,110] Syed M. Aljunid, PhD,[111,112] François Alla, PhD,[113] Amir Almasi-Hashiani, PhD,[114] Sami Almustanyir, MD,[115,116] Rajaa M. Al-Raddadi, PhD,[117] Adeladza K. Amegah, PhD,[118] Saeed Amini, PhD,[119] Arya Aminorroaya, MD,[120,121] Hubert Amu, PhD,[122] Dickson A. Amugsi, PhD,[123] Robert Ancuceanu, PhD,[124] Deanna Anderlini, MD,[125,126] Tudorel Andrei, PhD,[127] Catalina Liliana Andrei, PhD,[128] Alireza Ansari-Moghaddam, PhD,[129] Zelalem A. Anteneh, MPH,[130] Ippazio Cosimo Antonazzo, PhD,[131] Benny Antony, PhD,[132] Razique Anwer, PhD,[133] Lambert T. Appiah, FWACP,[134,135] Jalal Arabloo, PhD,[109] Johan Ärnlöv, PhD,[136,137] Kurnia D. Artanti, MSc,[138] Zerihun Ataro, MSc,[139] Marcel Ausloos, PhD,[140,127] Leticia Avila-Burgos, ScD,[141] Asma T. Awan, DrPH,[142,143] Mamaru A. Awoke, MPH,[144] Henok T. Ayele, PhD,[145,146] Muluken A. Ayza, MSc,[147] Samad Azari, PhD,[109] Darshan B. B, MD,[148] Nafiseh Baheiraei, PhD,[149] Atif A. Baig, PhD,[150] Ahad Bakhtiari, PhD,[151] Maciej Banach, PhD,[152,153] Palash C. Banik, MPhil,[154] Emerson A. Baptista, PhD,[155] Miguel A. Barboza, MD,[156,157] Lingkan Barua, MPH,[154] Sanjay Basu, PhD,[158,159] Neeraj Bedi, MD,[160,161] Yannick Béjot, PhD,[162,163] Derrick A. Bennett, PhD,[164] Isabela M. Bensenor, PhD,[165] Adam E. Berman, MD,[166] Yihienew M. Bezabih, MD,[167,168] Akshaya S. Bhagavathula, PharmD,[169,170] Sonu Bhaskar, PhD,[171,172] Krittika Bhattacharyya, MSc,[173,174] Ali Bijani, PhD,[175] Boris Bikbov, MD,[176] Mulugeta M. Birhanu, MSc,[177,178] Archith Boloor, MD,[179] Luisa C. Brant, PhD,[58] Hermann Brenner, MD,[180] Nikolay I. Briko, DSc,[181] Zahid A. Butt, PhD,[182,183] Florentino Luciano Caetano dos Santos, PhD,[184] Leah E. Cahill, PhD,[185,186] Lucero Cahuana-Hurtado, PhD,[187] Luis A. Cámera, MD,[188,189] Ismael R. Campos-Nonato, PhD,[190] Carlos Cantu-Brito, PhD,[191] Josip Car, PhD,[192,193]

Juan J. Carrero, PhD,[194] Felix Carvalho, PhD,[195] Carlos A. Castañeda-Orjuela, MD,[196,197] Ferrán Catalá-López, PhD,[198,199] Ester Cerin, PhD,[200,201] Jaykaran Charan, MD,[202] Vijay Kumar Chattu, MD,[203,204] Simiao Chen, DSc,[205] Ken L. Chin, PhD,[108,206] Jee-Young J. Choi, PhD,[207] Dinh-Toi Chu, PhD,[208] Sheng-Chia Chung, PhD,[209,210] Massimo Cirillo, MD,[211] Sean Coffey, MB,[212] Sara Conti, PhD,[213] Vera M. Costa, PhD,[195] David K. Cundiff, MD,[214] Omid Dadras, DrPH,[215] Baye Dagnew, MSc,[216] Xiaochen Dai, PhD,[2] Albertino A. M. Damasceno, PhD,[217] Lalit Dandona, MD,[218,2,219] Rakhi Dandona, PhD,[218,2,3] Kairat Davletov, PhD,[220] Vanessa De la Cruz-Góngora, PhD,[221] Fernando P. De la Hoz, PhD,[222] Jan-Walter De Neve, MD,[205] Edgar Denova-Gutiérrez, DSc,[190] Meseret Derbew Molla, MSc,[223] Behailu T. Derseh, MPH,[224] Rupak Desai, MBBS,[225] Günther Deuschl, PhD,[226,227] Samath D. Dharmaratne, MD,[228,3,2] Meghnath Dhimal, PhD,[229] Raja Ram Dhungana, MPhil,[230] Mostafa Dianatinasab, MSc,[231,232] Daniel Diaz, PhD,[233,234] Shirin Djalalinia, PhD,[235] Klara Dokova, PhD,[236] Abdel Douiri, PhD,[237] Bruce B. Duncan, PhD,[238] Andre R. Duraes, PhD,[239,240] Arielle W. Eagan, MSW,[241,242] Sanam Ebtehaj, PhD,[243] Aziz Eftekhari, PhD,[244,245] Sahar Eftekharzadeh, MD,[246] Michael Ekholuenetale, MSc,[247,248] Nevine El Nahas, MD,[249] Islam Y. Elgendy, MD,[250,251] Muhammed Elhadi, MD,[252] Shaimaa I. El-Jaafary, MD,[253] Sadaf Esteghamati, MD,[254] Atkilt E. Etisso, MSc,[255] Oghenowede Eyawo, PhD,[256] Ibtihal Fadhil, PhD,[257] Emerito Jose A. Faraon, MD,[258] Pawan S. Faris, PhD,[259,260] Medhat Farwati, MD,[261,243] Farshad Farzadfar, DSc,[120] Eduarda Fernandes, PhD,[262] Carlota Fernandez Prendes, MD,[263,264] Pietro Ferrara, MD,[131] Irina Filip, MD,[265,266] Florian Fischer, PhD,[267] David Flood, MD,[268,26] Takeshi Fukumoto, PhD,[269] Mohamed M. Gad, MD,[270,271] Shilpa Gaidhane, PhD,[272] Morsaleh Ganji, MD,[254] Jalaj Garg, MD,[273] Abadi K. Gebre, MSc,[274,275] Birhan G. Gebregiorgis, MSc,[276] Kidane Z. Gebregzabiher, MSc,[80] Gebreamlak G. Gebremeskel, MSc,[277,278] Lemma Getacher, MPH,[224] Abera Getachew Obsa, MA,[279] Alireza Ghajar, MD,[280] Ahmad Ghashghaee, BSc,[109,281] Nermin Ghith, PhD,[282] Simona Giampaoli, MD,[283] Syed Amir Gilani, PhD,[284,285] Paramjit S. Gill, DM,[286] Richard F. Gillum, MD,[287,288] Ekaterina V. Glushkova, PhD,[181] Elena V. Gnedovskaya, PhD,[289] Mahaveer Golechha, PhD,[290] Kebebe B. Gonfa, MD,[291] Amir Hossein Goudarzian, MSc,[292] Alessandra C. Goulart, PhD,[293,165] Jenny S. Guadamuz, PhD,[294] Avirup Guha, MD,[295,296] Yuming Guo, PhD,[108,297] Rajeev Gupta, MD,[298,299] Vladimir Hachinski, DSc,[300,301] Nima Hafezi-Nejad, MD,[302,303] Teklehaimanot G. Haile, MSc,[277] Randah R. Hamadeh, PhD,[304] Samer Hamidi, DrPH,[305] Graeme J. Hankey, MD,[306,307] Arief Hargono, MD,[308] Risky K. Hartono, MPH,[309] Maryam Hashemian, PhD,[310,311] Abdiwahab Hashi, PhD,[312] Shoaib Hassan, MPhil,[313,314] Hamid Y. Hassen, MPH,[315,316] Rasmus J. Havmoeller, PhD,[317] Simon I. Hay, FMedSci,[2,3] Khezar Hayat, MS,[318,319] Golnaz Heidari, MD,[320] Claudiu Herteliu, PhD,[127,321] Ramesh Holla, MD,[148] Mostafa Hosseini, PhD,[322,323] Mehdi Hosseinzadeh, PhD,[324,325] Mihaela Hostiuc, PhD,[326] Sorin Hostiuc, PhD,[327,328] Mowafa Househ, PhD,[329] Junjie Huang, MD,[330] Ayesha Humayun, PhD,[331] Ivo Iavicoli, PhD,[211] Charles U. Ibeneme, MPH,[332,333] Segun E. Ibitoye, MPH,[334] Olayinka S. Ilesanmi, PhD,[335,336] Irena M. Ilic, PhD,[337] Milena D. Ilic, PhD,[338] Usman Iqbal, PhD,[339] Seyed Sina N. Irvani, MD,[340] Sheikh Mohammed Shariful Islam, PhD,[341,342] Rakibul M. Islam, PhD,[108] Hiroyasu Iso, MD,[343] Masao Iwagami, PhD,[344,345] Vardhmaan Jain, MD,[261] Tahereh Javaheri, PhD,[346] Sathish Kumar Jayapal, PhD,[347] Shubha Jayaram, MD,[348] Ranil Jayawardena, PhD,[349,350] Panniyammakal Jeemon, PhD,[351] Ravi P. Jha, MSc,[352,353] Jost B. Jonas, MD,[354,355] Jitendra Jonnagaddala, PhD,[356,357] Farahnaz Joukar, PhD,[358,359] Jacek J. Jozwiak, PhD,[360] Mikk Jürisson, PhD,[361] Ali Kabir, MD,[362] Tanvir Kahlon, MD,[363,364] Rizwan Kalani, MD,[365] Rohollah Kalhor, PhD,[366,367] Ashwin Kamath, MD,[368] Ibrahim Kamel, MD,[369,370] Himal Kandel, PhD,[371,372] Amit Kandel, MD,[373] André Karch, MD,[374] Ayele Semachew Kasa, MSc,[375] Patrick D.M.C. Katoto, PhD,[376,377] Gbenga A. Kayode, PhD,[378,379] Yousef S. Khader, PhD,[380] Mohammad Khammarnia, PhD,[381] Muhammad S. Khan, MD,[382,383] Md Nuruzzaman Khan, PhD,[384] Maseer Khan, MD,[385] Ejaz A. Khan, MPH,[386] Khaled Khatab, PhD,[387,388] Gulam M. A. Kibria, MS,[389,390] Yun Jin Kim, PhD,[391] Gyu Ri Kim, PhD,[392] Ruth W. Kimokoti, MD,[393] Sezer Kisa, PhD,[394] Adnan Kisa, PhD,[395,396] Mika Kivimäki, PhD,[397,398] Dhaval Kolte, MD,[250,60] Ali Koolivand, PhD,[399] Vladimir A. Korshunov, PhD,[181] Sindhura Lakshmi Koulmane Laxminarayana, MD,[400] Ai Koyanagi, MD,[401,402] Kewal Krishan, PhD,[403] Vijay Krishnamoorthy, MD,[404,405] Barthelemy Kuate Defo, PhD,[406,407] Burcu Kucuk Bicer, PhD,[408] Vaman Kulkarni, MD,[409] G Anil Kumar, PhD,[218] Nithin Kumar, MD,[409] Om P. Kurmi, PhD,[410,411] Dian

Kusuma, DSc,[412,413] Gene F. Kwan, MD,[11,414] Carlo La Vecchia, MD,[415] Ben Lacey, PhD,[164,416] Tea Lallukka, PhD,[398] Qing Lan, PhD,[417] Savita Lasrado, MS,[418] Zohra S. Lassi, PhD,[419] Paolo Lauriola, MD,[420] Wayne R. Lawrence, DrPH,[421,422] Avula Laxmaiah, PhD,[423] Kate E. LeGrand, MPH,[2] Ming-Chieh Li, PhD,[424] Bingyu Li, PhD,[425] Shanshan Li, PhD,[88] Stephen S. Lim, PhD,[2,3] Lee-Ling Lim, MRCP,[426,427] Hualiang Lin, PhD,[428] Ziqiang Lin, PhD,[429] Ro-Ting Lin, PhD,[430,431] Xuefeng Liu, PhD,[432] Alan D. Lopez, PhD,[86,2,3] Stefan Lorkowski, PhD,[433,434] Paulo A. Lotufo, DrPH,[435] Alessandra Lugo, PhD,[436] Nirmal K. M, MD,[437] Fabiana Madotto, PhD,[438] Morteza Mahmoudi, PhD,[439] Azeem Majeed, MD,[193] Reza Malekzadeh, MD,[311,440] Ahmad A. Malik, PhD,[441,442] Abdullah A. Mamun, PhD,[443] Navid Manafi, MD,[444,445] Mohammad Ali Mansournia, PhD,[322] Lorenzo G. Mantovani, DSc,[213,438] Santi Martini, PhD,[446,447] Manu R. Mathur, PhD,[448,449] Giampiero Mazzaglia, PhD,[450] Suresh Mehata, PhD,[451] Man Mohan Mehndiratta, MD,[452,453] Toni Meier, PhD,[454,455] Ritesh G. Menezes, MD,[456] Atte Meretoja, MD,[457,458] Tomislav Mestrovic, PhD,[459,460] Bartosz Miazgowski, MD,[461,462] Tomasz Miazgowski, MD,[463] Irmina Maria Michalek, PhD,[464] Ted R. Miller, PhD,[465,466] Erkin M. Mirrakhimov, PhD,[467,468] Hamed Mirzaei, PhD,[469] Babak Moazen, MSc,[205,470] Masoud Moghadaszadeh, PhD,[471,472] Yousef Mohammad, MD,[473] Dara K. Mohammad, PhD,[474,475] Shafiu Mohammed, PhD,[476,205] Mohammed A. Mohammed, PhD,[477] Yaser Mokhayeri, PhD,[478] Mariam Molokhia, PhD,[479] Ahmed A. Montasir, FMD,[480,481] Ghobad Moradi, PhD,[482,483] Rahmatollah Moradzadeh, PhD,[114] Paula Moraga, PhD,[484] Lidia Morawska, PhD,[485] Ilais Moreno Velásquez, PhD,[486] Jakub Morze, MD,[487] Sumaira Mubarik, MS,[488] Walter Muruet, MSc,[237] Kamarul Imran Musa, PhD,[489] Ahamarshan J. Nagarajan, MTech,[490,491] Mahdi Nalini, MD,[492,493] Vinay Nangia, MD,[494] Atta Abbas Naqvi, PhD,[495,496] Sreenivas Narasimha Swamy, MD,[497] Bruno R. Nascimento, PhD,[498,499] Vinod C. Nayak, MD,[500] Javad Nazari, MD,[501] Milad Nazarzadeh, PhD,[502] Ruxandra I. Negoi, PhD,[503,504] Sandhya Neupane Kandel, BSN,[505] Huong L. T. Nguyen, MPH,[506] Molly R. Nixon, PhD,[2] Bo Norrving, PhD,[507] Jean Jacques Noubiap, MD,[508] Brice E. Nouthe, MDCM,[509] Christoph Nowak, PhD,[510] Oluwakemi O. Odukoya, MSc,[511,512] Felix A. Ogbo, PhD,[513] Andrew T. Olagunju, MD,[514,515] Hans Orru, PhD,[361,516] Alberto Ortiz, MD,[517,518] Samuel M. Ostroff, PhD,[2,519] Jagadish Rao Padubidri, MD,[520] Raffaele Palladino, MD,[211,193] Adrian Pana, MD,[127,521] Songhomitra Panda-Jonas, MD,[354] Utsav Parekh, MD,[522] Eun-Cheol Park, PhD,[392,523] Mojtaba Parvizi, PhD,[524] Fatemeh Pashazadeh Kan, BSN,[525] Urvish K. Patel, MD,[526] Mona Pathak, PhD,[527] Rajan Paudel, MPH,[528] Veincent Christian F. Pepito, MSc,[529] Arokiasamy Perianayagam, PhD,[530] Norberto Perico, MD,[531] Hai Q. Pham, MD,[506] Thomas Pilgrim, MD,[532] Michael A. Piradov, DSc,[28] Farhad Pishgar, MD,[120,533] Vivek Podder, HSC,[534,535] Roman V. Polibin, PhD,[181] Akram Pourshams, MD,[311] Dimas R. A. Pribadi, MSc,[536] Navid Rabiee, MSc,[537] Mohammad Rabiee, PhD,[538] Amir Radfar, MD,[539] Alireza Rafiei, PhD,[540,541] Fakher Rahim, PhD,[542,543] Vafa Rahimi-Movaghar, MD,[544] Mohammad Hifz Ur Rahman, PhD,[545] Muhammad Aziz Rahman, PhD,[546,547] Amir Masoud Rahmani, PhD,[324,548] Ivo Rakovac, PhD,[549] Pradhum Ram, MD,[550] Sudha Ramalingam, MD,[551] Juwel Rana, MPH,[552,553] Priyanga Ranasinghe, PhD,[554] Sowmya J Rao, MDS,[555] Priya Rathi, MD,[148] Lal Rawal, PhD,[556] Wasiq F. Rawasia, MD,[557] Reza Rawassizadeh, PhD,[558] Giuseppe Remuzzi, MD,[531] Andre M. N. Renzaho, PhD,[559,560] Aziz Rezapour, PhD,[109] Seyed Mohammad Riahi, PhD,[561] Ross L. Roberts-Thomson, MB,[562] Leonardo Roever, PhD,[563] Peter Rohloff, MD,[564,565] Michele Romoli, MD,[566,567] Gholamreza Roshandel, PhD,[568] Godfrey M. Rwegerera, MD,[569] Seyedmohammad Saadatagah, MD,[243] Maha M. Saber-Ayad, MD,[81,570] Siamak Sabour, PhD,[571] Simona Sacco, MD,[572] Masoumeh Sadeghi, MD,[573] Sahar Saeedi Moghaddam, MSc,[120] Saeed Safari, MD,[574] Amirhossein Sahebkar, PhD,[575,576] Sana Salehi, MD,[577] Hamideh Salimzadeh, PhD,[311] Mehrnoosh Samaei, MD,[578] Abdallah M. Samy, PhD,[579] Itamar S. Santos, PhD,[165,580] Milena M. Santric-Milicevic, PhD,[337,581] Nizal Sarrafzadegan, MD,[582,15] Arash Sarveazad, PhD,[583] Thirunavukkarasu Sathish, PhD,[584] Monika Sawhney, PhD,[585] Mete Saylan, MD,[586] Maria I. Schmidt, PhD,[238] Aletta E. Schutte, PhD,[587,66] Subramanian Senthilkumaran, MD,[588] Sadaf G. Sepanlou, MD,[311,440] Feng Sha, PhD,[589] Saeed Shahabi, PhD,[590] Izza Shahid, MBBS,[591] Masood A. Shaikh, MD,[592] Mahdi Shamali, MSc,[593] Morteza Shamsizadeh, MSc,[594] Md Shajedur Rahman Shawon, PhD,[595] Aziz Sheikh, MD,[596,597] Mika Shigematsu, PhD,[598] Min-Jeong Shin, PhD,[599] Jae Il Shin, MD,[600] Rahman Shiri, PhD,[601] Ivy Shiue, PhD,[602] Kerem Shuval, PhD,[603,604] Soraya Siabani, PhD,[605,606] Tariq J. Siddiqi, MB,[607] Diego A. S. Silva,

PhD,[608] Jasvinder A. Singh, MD,[609,610] Ambrish Singh, Mtech,[132] Valentin Y. Skryabin, MD,[611] Anna A. Skryabina, MD,[612] Amin Soheili, PhD,[613] Emma E. Spurlock, BA,[2] Leo Stockfelt, PhD,[614] Stefan Stortecky, MD,[532] Saverio Stranges, MD,[615,616] Rizwan Suliankatchi Abdulkader, MD,[617,618] Hooman Tadbiri, MD,[619] Eyayou G. Tadesse, MSc,[620] Degena B. Tadesse, MSc,[277] Masih Tajdini, MD,[121] Md Tariqujjaman, MSc,[621] Berhane F. Teklehaimanot, MPH,[622] Mohamad-Hani Temsah, MD,[106] Ayenew K. Tesema, MPH,[623] Bhaskar Thakur, PhD,[624,625] Kavumpurathu R. Thankappan, MD,[626] Rekha Thapar, MD,[409] Amanda G. Thrift, PhD,[627] Binod Timalsina, MSc,[628] Marcello Tonelli, MD,[629] Mathilde Touvier, PhD,[630,631] Marcos R. Tovani-Palone, PhD,[632,633] Avnish Tripathi, PhD,[363] Jaya P. Tripathy, MD,[634] Thomas C. Truelsen, PhD,[635] Guesh M. Tsegay, MSc,[277] Gebiyaw W. Tsegaye, MPH,[636] Nikolaos Tsilimparis, PhD,[637] Biruk S. Tusa, MPH,[638] Stefanos Tyrovolas, PhD,[639,640] Krishna Kishore Umapathi, MD,[641] Brigid Unim, PhD,[642] Bhaskaran Unnikrishnan, MD,[643] Muhammad S. Usman, MB,[383] Muthiah Vaduganathan, MD,[644] Pascual R. Valdez, Med,[645,646] Tommi J. Vasankari, MD,[647] Diana Z. Velazquez, MSc,[234] Narayanaswamy Venketasubramanian, MBBS,[648,649] Giang T. Vu, BA,[650] Isidora S. Vujcic, PhD,[337] Yasir Waheed, PhD,[651] Yanzhong Wang, PhD,[237] Fang Wang, MA,[652] Jingkai Wei, PhD,[653] Robert G. Weintraub, MB,[654,655] Abrha H. Weldemariam, MSc,[80] Ronny Westerman, DSc,[656] Andrea S. Winkler, PhD,[657,658] Charles S. Wiysonge, MD,[659,660] Charles D. A. Wolfe, MD,[237,661] Befikadu Legesse Wubishet, MPH,[662,274] Gelin Xu, MD,[663] Ali Yadollahpour, PhD,[664] Kazumasa Yamagishi, MD,[665,666] Lijing L. Yan, PhD,[667,668] Srikanth Yandrapalli, MD,[669,670] Yuichiro Yano, MD,[671] Hiroshi Yatsuya, PhD,[672,673] Tomas Y. Yeheyis, MSc,[674] Yigizie Yeshaw, MPH,[675] Christopher S. Yilgwan, MD,[676,677] Naohiro Yonemoto, MPH,[678,679] Chuanhua Yu, PhD,[488] Hasan Yusefzadeh, PhD,[680] Geevar Zachariah, MD,[681] Sojib Bin Zaman, MPH,[682,683] Muhammed S. Zaman, MSc,[684] Maryam Zamanian, PhD,[114] Ramin Zand, MD,[685,686] Alireza Zandifar, MD,[687] Afshin Zarghi, PhD,[688] Mikhail S. Zastrozhin, PhD,[689,690] Anasthasia Zastrozhina, PhD,[691] Zhi-Jiang Zhang, PhD,[692] Yunquan Zhang, PhD,[693,694] Wangjian Zhang, PhD,[695] Chenwen Zhong, MD,[330] Zhiyong Zou, MD,[696] Yves Miel H. Zuniga, BS,[697,698] Christopher J. L. Murray, PhD,[2,3] Valentin Fuster, MD, PhD.[699,700]

GBD-NHLBI-JACC Global Burden of Cardiovascular Diseases Writing Group Affiliations

From the [1]Division of Cardiology, University of Washington, Seattle, WA, USA; [2]Institute for Health Metrics and Evaluation, University of Washington, Seattle, WA, USA; [3]Department of Health Metrics Sciences, School of Medicine, University of Washington, Seattle, WA, USA; [4]Center for Translation Research and Implementation Science, National Institutes of Health, Bethesda, MD, USA; [5]Department of Medicine, University of Cape Town, Cape Town, South Africa; [6]Department of Internal Medicine, Catholic University of Rome, Rome, Italy; [7]De Gasperis Cardio Center, Niguarda Hospital, Milano, Italy; [8]School of Medicine, Mayo Clinic College of Medicine, Rochester, MN, USA; [9]Department of Translational Medicine, Florida International University, Miami, FL, USA; [10]Cardiology Services, Children's National Medical Center, Washington, DC, USA; [11]School of Medicine, Boston University, Boston, MA, USA; [12]Heart and Vascular Center, Essentia Health, Duluth, MN, USA; [13]Faculty of Medicine and Pharmaceutical Sciences, University of Douala, Douala, Cameroon; [14]Department of Cardiology, Centre Hospitalier Montfermeil, Montfermeil, France; [15]School of Population and Public Health, University of British Columbia, Vancouver, BC, Canada; [16]Department of Internal Medicine, Medical University of Graz, Graz, Austria; [17]Department of Cardiology, Oxford University Hospitals NHS Foundation Trust, Oxford University, Oxford, UK; [18]Telethon Kids Institute, Nedlands, WA, Australia; [19]Centre for Child Health Research (CCHR), University of Western Australia, Nedlands, WA, Australia; [20]Department of Pharmacological and Biomolecular Sciences, University of Milan, Milan, Italy; [21]IRCCS MultiMedica, Sesto San Giovanni, Italy; [22]Heart Rhythm Center of Excellence, Cedars-Sinai Medical Center, Los Angeles, CA, USA; [23]Department of Cardiovascular Medicine, Mayo Clinic, Jacksonville, FL, USA; [24]Department of

Epidemiology, Johns Hopkins University, Baltimore, MD, USA; [25]Department of Family Medicine and Public Health, University of California San Diego, La Jolla, CA, USA; [26]Department of Internal Medicine, University of Michigan, Ann Arbor, MI, USA; [27]National Institute for Stroke and Applied Neurosciences, Auckland University of Technology, Auckland, New Zealand; [28]Research Center of Neurology, Moscow, Russia; [29]Department of Medicine, University of Barcelona, Barcelona, Spain; [30]Department of Internal Medicine, Hospital Clinic, Barcelona, Spain; [31]Usher Institute of Population Health Sciences and Informatics, University of Edinburgh, Edinburgh, UK; [32]Department of Internal Medicine, University of Texas, Dallas, TX, USA; [33]Wolfson Institute of Preventive Medicine, Queen Mary University of London, London, UK; [34]Department of Biostatistics, University of Alabama at Birmingham, Birmingham, AL, USA; [35]Department of Nutrition, Harvard T.H. Chan School of Public Health, Boston, MA, USA; [36]Kidney and Blood Pressure Center, Tufts Medical Center, Boston, MA, USA; [37]Department of Cardiology, All India Institute of Medical Sciences, New Delhi, India; [38]Department of Anesthesiology & Pain Medicine, University of Washington, Seattle, WA, USA; [39]National Institute of Neurological Disorders and Stroke, National Institute of Health, Bethesda, MD, USA; [40]Cardiac Rehabilitation and Prevention, Ochsner Health, New Orleans, LA, USA; [41]Department of Preventive Medicine, Northwestern, Chicago, IL, USA; [42]Physiology and Cardiovascular Research Center, University of Kentucky, Lexington, KY, USA; [43]Department of Medical Sciences, IRCCS Home for the Relief of Suffering General Hospital, San Giovanni Rotondo, Italy; [44]National Data Management Center, Ethiopian Public Health Institute, Addis Ababa, Ethiopia; [45]Department of Medicine, Columbia University Medical Center, New York, NY, USA; [46]Global Hypertension Control, Resolve to Save Lives, New York, NY, USA; [47]Department of Epidemiology, University of Alabama at Birmingham, Birmingham, AL, USA; [48]Division of Cardiology, Mount Sinai Hospital, New York, NY, USA; [49]Cardio-Metabolic Group, The George Institute for Global Health, Newtown, NSW, Australia; [50]School of Medicine, University of New South Wales, Randwick, NSW, Australia; [51]Division of Cardiology, University of Cape Town, Cape Town, South Africa; [52]The Cardiac Clinic, Groote Schuur Hospital, Cape Town, South Africa; [53]Cardiology Department, Federal University of Rio de Janeiro, Rio De Janeiro, Brazil; [54]Department of Medicine, University of Ibadan, Ibadan, Nigeria; [55]Department of Medicine, University College Hospital, Ibadan, Ibadan, Nigeria; [56]Institute for Public Health, University of California San Diego, San Diego, CA, USA; [57]Cardiovascular Research Institute, School of Medicine, Case Western Reserve University, Cleveland, OH, USA; [58]Department of Internal Medicine, Federal University of Minas Gerais, Belo Horizonte, Brazil; [59]Centre of Telehealth, Federal University of Minas Gerais, Belo Horizonte, Brazil; [60]Department of Medicine, Harvard University, Boston, MA, USA; [61]Department of Medicine, Massachusetts General Hospital, Boston, MA, USA; [62]Cardiovascular Division, The George Institute for Global Health, Sydney, NSW, Australia; [63]Department of Cardiology, Children's National Medical Center, Washington, DC, USA; [64]Hatter Institute Department of Medicine, University of Cape Town, Cape Town, South Africa; [65]Department of Medical Sciences, Uppsala University, Uppsala, Sweden; [66]The George Institute for Global Health, Sydney, NSW, Australia; [67]Department for Prevention and Care of Diabetes, Dresden University of Technology, Dresden, Germany; [68]Infectious Diseases Section, King Fahad Medical City, Riyadh, Saudi Arabia; [69]Division of Infectious Diseases, Mayo Clinic, Rochester, MN, USA; [70]Cardiology Department, Cardiocentro Ticino, Lugano, Switzerland; [71]University Clinic for Cardiology, University of Bern, Bern, Switzerland; [72]Department of Epidemiology, Tulane University, New Orleans, USA; [73]Heart Science Centre, Imperial College London, London, UK; [74]Department of Paediatrics and Child Health, University of Cape Town, Cape Town, South Africa; [75]Social Determinants of Health Research Center, Shahid Beheshti University of Medical Sciences, Tehran, Iran; [76]School of Nursing and Midwifery, Kermanshah University of Medical

Sciences, Kermanshah, Iran; [77]Department of Orthopaedic Surgery, University of Southern California, Los Angeles, CA, USA; [78]Department of Cardiology, Dupuytren University Hospital, Limoges, France; [79]University of Limoges, Limoges, France; [80]Department of Adult Health Nursing, Aksum University, Aksum, Ethiopia; [81]Department of Clinical Sciences, University of Sharjah, Sharjah, United Arab Emirates; [82]Harvard Medical School, Harvard University, Boston, MA, USA; [83]Department of Medicine, Ain Shams University, Cairo, Egypt; [84]Department of Preventive Medicine, Dongguk University, Gyeongju, South Korea; [85]Department of Community Medicine, Kathmandu University, Devdaha, Nepal; [86]Melbourne School of Population and Global Health, University of Melbourne, Melbourne, VIC, Australia; [87]College of Medicine, University College Hospital, Ibadan, Nigeria; [88]School of Public Health and Preventive Medicine, Monash University, Melbourne, VIC, Australia; [89]Social Behavioral Research Branch, National Institute of Health, Bethesda, MD, USA; [90]Department of Oncology, Georgetown University, Washington, DC, USA; [91]Brain and Spinal Cord Injury Research Center, Tehran University of Medical Sciences, Tehran, Iran; [92]Department of Pharmacology, Tehran University of Medical Sciences, Tehran, Iran; [93]Department of Family Medicine, McMaster University, Hamilton, ON, Canada; [94]Department of Cardiovascular Medicine, Mayo Clinic, Scottsdale, AZ, USA; [95]Faculty of Pharmacy, MAHSA University, Kuala Langat, Malaysia; [96]School of Advanced Technologies in Medicine, Shahid Beheshti University of Medical Sciences, Tehran, Iran; [97]Department of Epidemiology, Jimma University, Jimma, Ethiopia; [98]Australian Center for Precision Health, University of South Australia, Adelaide, SA, Australia; [99]Faculty of Medicine and Public Health, Jenderal Soedirman University, Purwokerto, Indonesia; [100]Department of Medical Physiology, University of Gondar, Gondar, Ethiopia; [101]Department of Clinical Pharmacy and Pharmacy Administration, University of Ibadan, Ibadan, Nigeria; [102]Department of Medical Laboratory Sciences, Arba Minch University, Arba Minch, Ethiopia; [103]Department of Public Health, The Intercountry Centre for Oral Health (ICOH) for Africa, Jos, Nigeria; [104]Department of Public Health, Federal Ministry of Health, Garki, Nigeria; [105]Mayo Evidence-based Practice Center, Mayo Clinic Foundation for Medical Education and Research, Rochester, MN, USA; [106]Pediatric Intensive Care Unit, King Saud University, Riyadh, Saudi Arabia; [107]Department of Cardiac Sciences, King Saud University, Riyadh, Saudi Arabia; [108]Department of Epidemiology and Preventive Medicine, Monash University, Melbourne, VIC, Australia; [109]Health Management and Economics Research Center, Iran University of Medical Sciences, Tehran, Iran; [110]Health Economics Department, Iran University of Medical Sciences, Tehran, Iran; [111]Department of Health Policy and Management, Kuwait University, Safat, Kuwait; [112]International Centre for Casemix and Clinical Coding, National University of Malaysia, Bandar Tun Razak, Malaysia; [113]Bordeaux School of Public Health, University of Bordeaux, Bordeaux, France; [114]Department of Epidemiology, Arak University of Medical Sciences, Arak, Iran; [115]College of Medicine, Alfaisal University, Riyadh, Saudi Arabia; [116]Ministry of Health, Riyadh, Saudi Arabia; [117]Department of Community Medicine, King Abdulaziz University, Jeddah, Saudi Arabia; [118]Department of Biomedical Science, University of Cape Coast, Cape Coast, Ghana; [119]Department of Health Services Management, Arak University of Medical Sciences, Arak, Iran; [120]Non-communicable Diseases Research Center, Tehran University of Medical Sciences, Tehran, Iran; [121]Tehran Heart Center, Tehran University of Medical Sciences, Tehran, Iran; [122]Department of Population and Behavioural Sciences, University of Health and Allied Sciences, Ho, Ghana; [123]Maternal and Child Wellbeing Center, African Population and Health Research Center, Nairobi, Kenya; [124]Department of Pharmacy, Carol Davila University of Medicine and Pharmacy, Bucharest, Romania; [125]Centre for Sensorimotor Performance, The University of Queensland, Brisbane, QLD, Australia; [126]Neurology Department, Royal Brisbane and Women's Hospital, Brisbane, QLD, Australia; [127]Department of Statistics and Econometrics, Bucharest University of Economic Studies, Bucharest,

Romania; [128]Department of Cardiology, Carol Davila University of Medicine and Pharmacy, Bucharest, Romania; [129]Department of Epidemiology and Biostatistics, Zahedan University of Medical Sciences, Zahedan, Iran; [130]Department of Epidemiology, Bahir Dar University, Bahir Dar, Ethiopia; [131]Research Center on Public Health, University of Milan Bicocca, Monza, Italy; [132]Menzies Institute for Medical Research, University of Tasmania, Hobart, TAS, Australia; [133]Department of Pathology, Imam Mohammad Ibn Saud Islamic University, Riyadh, Saudi Arabia; [134]Department of Internal Medicine, Komfo Anokye Teaching Hospital, Adum, Ghana; [135]School of Medical Sciences, Kwame Nkrumah University of Science and Technology, Kumasi, Ghana; [136]Department of Neurobiology, Care Sciences and Society, Karolinska Institute, Stockholm, Sweden; [137]School of Health and Social Studies, Dalarna University, Falun, Sweden; [138]Department of Epidemiology, Airlangga University, Surabaya, Indonesia; [139]Department of Medical Laboratory Science, Haramaya University, Harar, Ethiopia; [140]School of Business, University of Leicester, Leicester, UK; [141]Center for Health Systems Research, National Institute of Public Health, Cuernavaca, Mexico; [142]School of Nursing and Health Sciences, Capella University, Minneapolis, MN, USA; [143]Continuing Education- Grant Writing Academy, University of Nevada, Las Vegas, NV, USA; [144]Department of Epidemiology and Preventive Medicine, University of Melbourne, Melbourne, VIC, Australia; [145]Department of Epidemiology, Biostatistics, and Occupational Health, McGill University, Montreal, QC, Canada; [146]Public Health Department, Dilla University, Dilla, Ethiopia; [147]Department of Pharmacology and Toxicology, Mekelle University, Mekelle, Ethiopia; [148]Kasturba Medical College, Mangalore, Manipal Academy of Higher Education, Manipal, India; [149]Department of Hematology, Tarbiat Modares University, Tehran, Iran; [150]Unit of Biochemistry, Sultan Zainal Abidin University, Kuala Terengganu, Malaysia; [151]Department of Health Policy, Management, and Economics, Tehran University of Medical Sciences, Tehran, Iran; [152]Department of Hypertension, Medical University of Lodz, Lodz, Poland; [153]Polish Mothers' Memorial Hospital Research Institute, Lodz, Poland; [154]Department of Non-communicable Diseases, Bangladesh University of Health Sciences, Dhaka, Bangladesh; [155]Asian Demographic Research Institute, Shanghai University, Shanghai, China; [156]Department of Neurosciences, Costa Rican Department of Social Security, San Jose, Costa Rica; [157]School of Medicine, University of Costa Rica, San Pedro, Costa Rica; [158]Center for Primary Care, Harvard University, Boston, MA, USA; [159]School of Public Health, Imperial College London, London, UK; [160]Department of Community Medicine, Gandhi Medical College Bhopal, Bhopal, India; [161]Jazan University, Jazan, Saudi Arabia; [162]Department of Neurology, University Hospital of Dijon, Dijon, France; [163]Dijon Stroke Registry - UFR Sciences Santé, University of Burgundy, Dijon, France; [164]Nuffield Department of Population Health, University of Oxford, Oxford, UK; [165]Department of Internal Medicine, University of São Paulo, São Paulo, Brazil; [166]Department of Medicine, Medical College of Georgia at Augusta University, Augusta, GA, USA; [167]Department of Internal Medicine, Bahir Dar University, Bahir Dar, Ethiopia; [168]One Health, University of Nantes, Nantes, France; [169]Department of Social and Clinical Pharmacy, Charles University, Hradec Kralova, Czech Republic; [170]Institute of Public Health, United Arab Emirates University, Al Ain, United Arab Emirates; [171]Neurovascular Imaging, NSW Brain Clot Bank, Sydney, NSW, Australia; [172]Department of Neurology and Neurophysiology, South West Sydney Local Heath District and Liverpool Hospital, Sydney, NSW, Australia; [173]Department of Statistical and Computational Genomics, National Institute of Biomedical Genomics, Kalyani, India; [174]Department of Statistics, University of Calcutta, Kolkata, India; [175]Social Determinants of Health Research Center, Babol University of Medical Sciences, Babol, Iran; [176]Mario Negri Institute for Pharmacological Research, Ranica, Italy; [177]Stroke and Ageing Research Group, Epidemiology and Prevention Division, Monash University, Melbourne, VIC, Australia; [178]Department of Nursing, St. Paul's Hospital Millennium Medical

College, Addis Ababa, Ethiopia; [179]Department of Internal Medicine, Manipal Academy of Higher Education, Mangalore, India; [180]Division of Clinical Epidemiology and Aging Research, German Cancer Research Center, Heidelberg, Germany; [181]Department of Epidemiology and Evidence-Based Medicine, I.M. Sechenov First Moscow State Medical University, Moscow, Russia; [182]School of Public Health and Health Systems, University of Waterloo, Waterloo, ON, Canada; [183]Al Shifa School of Public Health, Al Shifa Trust Eye Hospital, Rawalpindi, Pakistan; [184]Institute of Microengineering, Federal Polytechnic School of Lausanne, Lausanne, Switzerland; [185]Department of Medicine, Dalhousie University, Halifax, NS, Canada; [186]Department of Nutrition, Harvard University, Boston, MA, USA; [187]School of Public Health and Administration, Peruvian University Cayetano Heredia, Lima, Peru; [188]Internal Medicine Department, Hospital Italiano de Buenos Aires, Buenos Aires, Argentina; [189]Board of Directors, Argentine Society of Medicine, Buenos Aires, Argentina; [190]Center for Nutrition and Health Research, National Institute of Public Health, Cuernavaca, Mexico; [191]Department of Neurology, Salvador Zubiran National Institute of Medical Sciences and Nutrition, Mexico City, Mexico; [192]Centre for Population Health Sciences, Nanyang Technological University, Singapore, Singapore; [193]Department of Primary Care and Public Health, Imperial College London, London, UK; [194]Department of Medical Epidemiology and Biostatistics, Karolinska Institute, Stockholm, Sweden; [195]Research Unit on Applied Molecular Biosciences (UCIBIO), University of Porto, Porto, Portugal; [196]Colombian National Health Observatory, National Institute of Health, Bogota, Colombia; [197]Epidemiology and Public Health Evaluation Group, National University of Colombia, Bogota, Colombia; [198]National School of Public Health, Institute of Health Carlos III, Madrid, Spain; [199]Clinical Epidemiology Program, Ottawa Hospital Research Institute, Ottawa, ON, Canada; [200]Mary MacKillop Institute for Health Research, Australian Catholic University, Melbourne, VIC, Australia; [201]School of Public Health, University of Hong Kong, Hong Kong, China; [202]Department of Pharmacology, All India Institute of Medical Sciences, Jodhpur, India; [203]Faculty of Medical Sciences, University of the West Indies, St Augustine, Trinidad and Tobago; [204]Independent Consultant, Athens, Greece; [205]Heidelberg Institute of Global Health (HIGH), Heidelberg University, Heidelberg, Germany; [206]Melbourne Medical School, University of Melbourne, Parkville, VIC, Australia; [207]Biomedical Informatics, Seoul National University Hospital, Seoul, South Korea; [208]Faculty of Biology, Hanoi National University of Education, Hanoi, Vietnam; [209]Department of Health Informatics, University College London, London, UK; [210]Health Data Research UK, London, UK; [211]Department of Public Health, University of Naples Federico II, Naples, Italy; [212]Department of Medicine, University of Otago, Dunedin, New Zealand; [213]School of Medicine and Surgery, University of Milan Bicocca, Monza, Italy; [214]Department of Internal Medicine, University of Southern California, Los Angeles, CA, USA; [215]Department of Global Health and Socioepidemiology, Kyoto University, Kyoto, Japan; [216]Department of Human Physiology, University of Gondar, Gondar, Ethiopia; [217]Faculty of Medicine, Eduardo Mondlane University, Maputo, Mozambique; [218]Public Health Foundation of India, Gurugram, India; [219]Indian Council of Medical Research, New Delhi, India; [220]Health Research Institute, Al Farabi Kazakh National University, Almaty, Kazakhstan; [221]Center for Evaluation and Surveys Research, National Institute of Public Health, Cuernavaca, Mexico; [222]Department of Public Health, National University of Colombia, Bogota, Colombia; [223]Department of Biochemistry, University of Gondar, Gondar, Ethiopia; [224]Department of Public Health, Debre Berhan University, Debre Berhan, Ethiopia; [225]Division of Cardiology, Atlanta Veterans Affairs Medical Center, Decatur, GA, USA; [226]Department of Neurology, Christian-Albrechts University, Kiel, Germany; [227]Universitätsklinikum Schleswig-Holstein, Kiel Campus (Schleswig-Holstein University Hospital, Kiel Campus), Christian-Albrechts University, Kiel, Germany; [228]Department of Community Medicine, University of Peradeniya, Peradeniya, Sri Lanka; [229]Health

Research Section, Nepal Health Research Council, Kathmandu, Nepal; [230]Institute for Health and Sport, Victoria University, Footscray, VIC, Australia; [231]Department of Epidemiology and Biostatistics, Shahroud University of Medical Sciences, Shahroud, Iran; [232]Department of Epidemiology, Shiraz University of Medical Sciences, Shiraz, Iran; [233]Center of Complexity Sciences, National Autonomous University of Mexico, Mexico City, Mexico; [234]Faculty of Veterinary Medicine and Zootechnics, Autonomous University of Sinaloa, Culiacán Rosales, Mexico; [235]Development of Research and Technology Center, Ministry of Health and Medical Education, Tehran, Iran; [236]Department of Social Medicine and Health Care Organisation, Medical University of Varna, Varna, Bulgaria; [237]School of Population Health and Environmental Sciences, King's College London, London, UK; [238]Postgraduate Program in Epidemiology, Federal University of Rio Grande do Sul, Porto Alegre, Brazil; [239]School of Medicine, Federal University of Bahia, Salvador, Brazil; [240]Department of Internal Medicine, Escola Bahiana de Medicina e Saúde Pública (BAHIANA School of Medicine and Public Health), Salvador, Brazil; [241]Department of Global Health and Social Medicine, Harvard University, Boston, MA, USA; [242]Department of Social Services, Tufts Medical Center, Boston, MA, USA; [243]Department of Cardiovascular Medicine, Mayo Clinic, Rochester, MN, USA; [244]Department of Pharmacology and Toxicology, Maragheh University of Medical Sciences, Maragheh, Iran; [245]Department of Pharmacology and Toxicology, The John Paul II Catholic University of Lublin, Lublin, Poland; [246]Division of Urology, Children's Hospital of Philadelphia, Philadelphia, PA, USA; [247]Department of Epidemiology and Medical Statistics, University of Ibadan, Ibadan, Nigeria; [248]Faculty of Public Health, University of Ibadan, Ibadan, Nigeria; [249]Neurology Department, Ain Shams University, Cairo, Egypt; [250]Division of Cardiology, Massachusetts General Hospital, Boston, MA, USA; [251]Division of Cardiology, Harvard University, Boston, MA, USA; [252]Faculty of Medicine, University of Tripoli, Tripoli, Libya; [253]Department of Neurology, Cairo University, Cairo, Egypt; [254]Endocrinology and Metabolism Research Center, Tehran University of Medical Sciences, Tehran, Iran; [255]Unit of Medical Physiology, Hawassa University, Hawassa, Ethiopia; [256]Faculty of Health, York University, Toronto, BC, Canada; [257]Division of Non-communicable Diseases, Ministry of Public Health and Population, Dubai, United Arab Emirates; [258]Department of Health Policy and Administration, University of the Philippines Manila, Manila, Philippines; [259]Department of Biology and Biotechnology "Lazzaro Spallanzani", University of Pavia, Pavia, Italy; [260]Department of Biology, Cihan University-Erbil, Erbil, Iraq; [261]Department of Internal Medicine, Cleveland Clinic, Cleveland, OH, USA; [262]Associated Laboratory for Green Chemistry (LAQV), University of Porto, Porto, Portugal; [263]Deparment of Vascular Surgery, Ludwig Maximilians University, Munich, Germany; [264]Department of Vascular Surgery, Uppsala University, Uppsala, Sweden; [265]Psychiatry Department, Kaiser Permanente, Fontana, CA, USA; [266]School of Health Sciences, A.T. Still University, Mesa, AZ, USA; [267]Institute of Gerontological Health Services and Nursing Research, Ravensburg-Weingarten University of Applied Sciences, Weingarten, Germany; [268]Center for Research in Indigenous Health, Maya Health Alliance, Tecpán, Guatemala; [269]Department of Dermatology, Kobe University, Kobe, Japan; [270]Department of Cardiovascular Medicine, Cleveland Clinic, Cleveland, OH, USA; [271]Gillings School of Global Public Health, University of North Carolina Chapel Hill, Chapel Hill, NC, USA; [272]Department of Medicine, Datta Meghe Institute of Medical Science, Wardha, India; [273]Division of Cardiovascular Medicine, Medical College of Wisconsin, Milwaukee, WI, USA; [274]School of Pharmacy, Mekelle University, Mekelle, Ethiopia; [275]School of Medical and Health Sciences, Edith Cowan University, Perth, WA, Australia; [276]Department of Nursing, Debre Berhan University, Debre Berhan, Ethiopia; [277]Department of Nursing, Aksum University, Aksum, Ethiopia; [278]Department of Nursing, Mekelle University, Mekelle, Ethiopia; [279]Department of Psychology, Ambo University, Oromia, Ethiopia; [280]Mount Auburn Hospital, Harvard Medical School, Cambridge, MA, USA; [281]Student Research

Committee, Iran University of Medical Sciences, Tehran, Iran; [282]Research Group for Genomic Epidemiology, Technical University of Denmark, Copenhagen, Denmark; [283]Department of Cardiovascular Endocrine-metabolic Diseases and Aging, Italian National Institute of Health, Rome, Italy; [284]Faculty of Allied Health Sciences, The University of Lahore, Lahore, Pakistan; [285]Afro-Asian Institute, Lahore, Pakistan; [286]Warwick Medical School, University of Warwick, Coventry, UK; [287]Division of General Internal Medicine, Howard University, Washington, DC, USA; [288]Department of Community and Family Medicine, Howard University, Washington, DC, USA; [289]Third Department of Neurology, Research Center of Neurology, Moscow, Russia; [290]Health Systems and Policy Research, Indian Institute of Public Health Gandhinagar, Gandhinagar, India; [291]Department of Surgery, Madda Walabu University, Bale Robe, Ethiopia; [292]Faculty of Nursing and Midwifery, Mazandaran University of Medical Sciences, Sari, Iran; [293]Center for Clinical and Epidemiological Research, University of São Paulo, Sao Paulo, Brazil; [294]Department of Pharmacy Systems, Outcomes, and Policy, University of Illinois at Chicago, Chicago, IL, USA; [295]Harrington Heart and Vascular Institute, Case Western Reserve University, Cleveland, OH, USA; [296]Division of Cardiovascular Medicine, Ohio State University, Columbus, OH, USA; [297]Department of Epidemiology, Binzhou Medical University, Yantai City, China; [298]Department of Preventive Cardiology, Eternal Heart Care Centre & Research Institute, Jaipur, India; [299]Department of Medicine, Mahatma Gandhi University Medical Sciences, Jaipur, India; [300]Department of Clinical Neurological Sciences, The University of Western Ontario, London, ON, Canada; [301]Lawson Health Research Institute, London, ON, Canada; [302]Department of Radiology and Radiological Sciences, Johns Hopkins University, Baltimore, MD, USA; [303]School of Medicine, Tehran University of Medical Sciences, Tehran, Iran; [304]Department of Family and Community Medicine, Arabian Gulf University, Manama, Bahrain; [305]School of Health and Environmental Studies, Hamdan Bin Mohammed Smart University, Dubai, United Arab Emirates; [306]Medical School, University of Western Australia, Perth, WA, Australia; [307]Department of Neurology, Sir Charles Gairdner Hospital, Perth, WA, Australia; [308]Department of Epidemiology, Universitas Airlangga (Airlangga University), Surabaya, Indonesia; [309]Sekolah Tinggi Ilmu Kesehatan Indonesia Maju (Indonesian Advanced College of Health Sciences), Institution of Public Health Sciences, Jakarta, Indonesia; [310]Biology Department, Utica College, Utica, NY, USA; [311]Digestive Diseases Research Institute, Tehran University of Medical Sciences, Tehran, Iran; [312]Department of Public Health, Jigjiga University, Jijiga, Ethiopia; [313]Center for International Health (CIH), University of Bergen, Bergen, Norway; [314]Bergen Center for Ethics and Priority Setting (BCEPS), University of Bergen, Bergen, Norway; [315]Department of Primary and Interdisciplinary Care, University Hospital Antwerp, Antwerp, Belgium; [316]Department of Public Health, Mizan-Tepi University, Mizan Teferi, Ethiopia; [317]Skaane University Hospital, Skaane County Council, Malmoe, Sweden; [318]Institute of Pharmaceutical Sciences, University of Veterinary and Animal Sciences, Lahore, Pakistan; [319]Department of Pharmacy Administration and Clinical Pharmacy, Xian Jiaotong University, Xian, China; [320]Independent Consultant, Santa Clara, CA, USA; [321]School of Business, London South Bank University, London, UK; [322]Department of Epidemiology and Biostatistics, Tehran University of Medical Sciences, Tehran, Iran; [323]Pediatric Chronic Kidney Disease Research Center, Tehran University of Medical Sciences, Tehran, Iran; [324]Institute of Research and Development, Duy Tan University, Da Nang, Vietnam; [325]Department of Computer Science, University of Human Development, Sulaymaniyah, Iraq; [326]Department of Internal Medicine, Carol Davila University of Medicine and Pharmacy, Bucharest, Romania; [327]Department of Legal Medicine and Bioethics, Carol Davila University of Medicine and Pharmacy, Bucharest, Romania; [328]Clinical Legal Medicine Department, National Institute of Legal Medicine Mina Minovici, Bucharest, Romania; [329]College of Science and Engineering, Hamad Bin Khalifa University, Doha, Qatar; [330]Jockey Club School of Public

Health and Primary Care, The Chinese University of Hong Kong, Hong Kong, China; [331]Department of Public Health and Community Medicine, Shaikh Khalifa Bin Zayed Al-Nahyan Medical College, Lahore, Pakistan; [332]Department of Public Health and Disease Control, Ministry of Health, Umuahia, Nigeria; [333]Nigerian Field Epidemiology and Laboratory Training Program, African Field Epidemiology Network, Abuja, Nigeria; [334]Department of Health Promotion and Education, University of Ibadan, Ibadan, Nigeria; [335]Department of Community Medicine, University of Ibadan, Ibadan, Nigeria; [336]Department of Community Medicine, University College Hospital, Ibadan, Ibadan, Nigeria; [337]Faculty of Medicine, University of Belgrade, Belgrade, Serbia; [338]Department of Epidemiology, University of Kragujevac, Kragujevac, Serbia; [339]College of Public Health, Taipei Medical University, Taipei, Taiwan; [340]Research Institute for Endocrine Sciences, Shahid Beheshti University of Medical Sciences, Tehran, Iran; [341]Institute for Physical Activity and Nutrition, Deakin University, Burwood, VIC, Australia; [342]Sydney Medical School, University of Sydney, Sydney, NSW, Australia; [343]Public Health Department of Social Medicine, Osaka University, Suita, Japan; [344]Department of Health Services Research, University of Tsukuba, Tsukuba, Japan; [345]Department of Non-communicable Disease Epidemiology, London School of Hygiene & Tropical Medicine, London, UK; [346]Health Informatics Lab, Boston University, Boston, MA, USA; [347]Centre of Studies and Research, Ministry of Health, Muscat, Oman; [348]Department of Biochemistry, Government Medical College, Mysuru, India; [349]Department of Physiology, University of Colombo, Colombo, Sri Lanka; [350]School of Exercise and Nutrition Sciences, Queensland University of Technology, Brisbane, QLD, Australia; [351]Achutha Menon Centre for Health Science Studies, Sree Chitra Tirunal Institute for Medical Sciences and Technology, Trivandrum, India; [352]Department of Community Medicine, Dr. Baba Saheb Ambedkar Medical College & Hospital, Delhi, India; [353]Department of Community Medicine, Banaras Hindu University, Varanasi, India; [354]Department of Ophthalmology, Heidelberg University, Heidelberg, Germany; [355]Beijing Institute of Ophthalmology, Beijing Tongren Hospital, Beijing, China; [356]School of Public Health and Community Medicine, University of New South Wales, Kensington, NSW, Australia; [357]New South Wales Health, Sydney, NSW, Australia; [358]Gastrointestinal and Liver Diseases Research Center, Guilan University of Medical Sciences, Rasht, Iran; [359]Caspian Digestive Disease Research Center, Guilan University of Medical Sciences, Rasht, Iran; [360]Department of Family Medicine and Public Health, University of Opole, Opole, Poland; [361]Institute of Family Medicine and Public Health, University of Tartu, Tartu, Estonia; [362]Minimally Invasive Surgery Research Center, Iran University of Medical Sciences, Tehran, Iran; [363]Division of Cardiovascular Medicine, University of Louisville, Louisville, KY, USA; [364]Department of Medicine, Albert Einstein College of Medicine, Bronx, NY, USA; [365]Department of Neurology, University of Washington, Seattle, WA, USA; [366]Institute for Prevention of Non-communicable Diseases, Qazvin University of Medical Sciences, Qazvin, Iran; [367]Health Services Management Department, Qazvin University of Medical Sciences, Qazvin, Iran; [368]Department of Pharmacology, Manipal Academy of Higher Education, Mangalore, India; [369]University of La Verne, La Verne, CA, USA; [370]Department of Medicine, October 6 University, Giza, Egypt; [371]Ophthalmology Department, University of Sydney, Sydney, NSW, Australia; [372]Ophthalmology Department, Sydney Local Health District, Sydney, NSW, Australia; [373]Department of Neurology, University at Buffalo, Buffalo, NY, USA; [374]Institute for Epidemiology and Social Medicine, University of Münster, Münster, Germany; [375]Department of Adult Health Nursing, Bahir Dar University, Bahir Dar, Ethiopia; [376]Internal Medicine and Centre for Tropical Medicine and Global Health, Catholic University of Bukavu, Bukavu, Democratic Republic of the Congo; [377]Department of Global Health, Stellenbosch University, Cape Town, South Africa; [378]International Research Center of Excellence, Institute of Human Virology Nigeria, Abuja, Nigeria; [379]Julius Centre for Health Sciences and Primary Care, Utrecht

University, Utrecht, Netherlands; [380]Department of Public Health, Jordan University of Science and Technology, Irbid, Jordan; [381]Health Promotion Research Center, Zahedan University of Medical Sciences, Zahedan, Iran; [382]Department of Internal Medicine, John H. Stroger, Jr. Hospital of Cook County , Chicago, IL, USA; [383]Department of Internal Medicine, Dow University of Health Sciences, Karachi, Pakistan; [384]Department of Population Science, Jatiya Kabi Kazi Nazrul Islam University, Mymensingh, Bangladesh; [385]Epidemiology Department, Jazan University, Jazan, Saudi Arabia; [386]Department of Epidemiology and Biostatistics, Health Services Academy, Islamabad, Pakistan; [387]Faculty of Health and Wellbeing, Sheffield Hallam University, Sheffield, UK; [388]College of Arts and Sciences, Ohio University, Zanesville, OH, USA; [389]Department of Epidemiology and Public Health, University of Maryland, Baltimore, MD, USA; [390]Department of International Health, Johns Hopkins University, Baltimore, MD, USA; [391]School of Traditional Chinese Medicine, Xiamen University Malaysia, Sepang, Malaysia; [392]Department of Preventive Medicine, Yonsei University, Seoul, South Korea; [393]Department of Nutrition, Simmons University, Boston, MA, USA; [394]Department of Nursing and Health Promotion, Oslo Metropolitan University, Oslo, Norway; [395]School of Health Sciences, Kristiania University College, Oslo, Norway; [396]Department of Global Community Health and Behavioral Sciences, Tulane University, New Orleans, LA, USA; [397]Department of Epidemiology and Public Health, University College London, London, UK; [398]Department of Public Health, University of Helsinki, Helsinki, Finland; [399]Department of Environmental Health Engineering, Arak University of Medical Sciences, Arak, Iran; [400]Kasturba Medical College, Udupi, India; [401]Biomedical Research Networking Center for Mental Health Network (CIBERSAM), San Juan de Dios Sanitary Park, Sant Boi de Llobregat, Spain; [402]Catalan Institution for Research and Advanced Studies (ICREA), Barcelona, Spain; [403]Department of Anthropology, Panjab University, Chandigarh, India; [404]Department of Anesthesiology, Duke University, Durham, NC, USA; [405]Department of Anesthesiology, University of Washington, Seattle, WA, USA; [406]Department of Demography, University of Montreal, Montreal, QC, Canada; [407]Department of Social and Preventive Medicine, University of Montreal, Montreal, QC, Canada; [408]Faculty of Medicine, Gazi University, Ankara, Turkey; [409]Department of Community Medicine, Manipal Academy of Higher Education, Mangalore, India; [410]Faculty of Health and Life Sciences, Coventry University, Coventry, UK; [411]Department of Medicine, McMaster University, Hamilton, ON, Canada; [412]Imperial College Business School, Imperial College London, London, UK; [413]Faculty of Public Health, University of Indonesia, Depok, Indonesia; [414]Partners In Health, Boston, MA, USA; [415]Department of Clinical Sciences and Community Health, University of Milan, Milan, Italy; [416]National Institute for Health Research (NIHR) Oxford Biomedical Research Centre, Oxford, UK; [417]Division of Cancer Epidemiology and Genetics, National Cancer Institute, Rockville, MD, USA; [418]Department of Otorhinolaryngology, Father Muller Medical College, Mangalore, India; [419]Robinson Research Institute, University of Adelaide, Adelaide, SA, Australia; [420]Institute of Clinical Physiology, National Research Council, Pisa, Italy; [421]Division of Cancer Prevention, National Institutes of Health, Rockville, MD, USA; [422]Department of Epidemiology and Biostatistics, State University of New York, Rensselaer, NY, USA; [423]National Institute of Nutrition, Indian Council of Medical Research, Hyderabad, India; [424]Department of Public Health, China Medical University, Taichung, Taiwan; [425]Department of Sociology, Shenzhen University, Shenzhen, China; [426]Department of Medicine, University of Malaya, Kuala Lumpur, Malaysia; [427]Department of Medicine and Therapeutics, The Chinese University of Hong Kong, Shatin, China; [428]School of Public Health, Zhengzhou University, Zhengzhou, China; [429]Department of Psychiatry, New York University, New York, NY, USA; [430]College of Public Health, China Medical University, Taichung, Taiwan; [431]Asbestos Diseases Research Institute, Asbestos Diseases Research Institute, Concord, NSW, Australia; [432]Department of Systems, Populations,

and Leadership, University of Michigan, Ann Arbor, MI, USA; [433]Institute of Nutritional Sciences, Friedrich Schiller University Jena, Jena, Germany; [434]Competence Cluster for Nutrition and Cardiovascular Health (nutriCARD), Jena, Germany; [435]Department of Medicine, University of São Paulo, Sao Paulo, Brazil; [436]Department of Environmental Health Sciences, Mario Negri Institute for Pharmacological Research, Milan, Italy; [437]Department of Forensic Medicine, Manipal Academy of Higher Education, Manipal, India; [438]Value-Based Healthcare Unit, IRCCS MultiMedica, Sesto San Giovanni, Italy; [439]Radiology and Precision Health Program, Michigan State University, East Lansing, MI, USA; [440]Non-communicable Disease Research Center, Shiraz University of Medical Sciences, Shiraz, Iran; [441]Rabigh Faculty of Medicine, King Abdulaziz University, Jeddah, Saudi Arabia; [442]University Institute of Public Health, The University of Lahore, Lahore, Pakistan; [443]Institute for Social Science Research, The University of Queensland, Indooroopilly, QLD, Australia; [444]School of Medicine, Iran University of Medical Sciences, Tehran, Iran; [445]School of Medicine, University of Manitoba, Winnipeg, MB, Canada; [446]Faculty of Public Health, Universitas Airlangga (Airlangga University), Surabaya, Indonesia; [447]Indonesian Public Health Association, Surabaya, Indonesia; [448]Health Policy Research, Public Health Foundation of India, Gurugram, India; [449]Institute of Population Health Sciences, University of Liverpool, Liverpool, UK; [450]Department of Medicine, University of Milan Bicocca, Monza, Italy; [451]Ministry of Health, Kathmandu, Nepal; [452]Neurology Department, Janakpuri Super Specialty Hospital Society, New Delhi, India; [453]Department of Neurology, Govind Ballabh Institute of Medical Education and Research, New Delhi, India; [454]Institute for Agricultural and Nutritional Sciences, Martin Luther University Halle-Wittenberg, Halle, Germany; [455]Office of Innovation, Competence Cluster for Nutrition and Cardiovascular Health (nutriCARD), Halle, Germany; [456]Forensic Medicine Division, Imam Abdulrahman Bin Faisal University, Dammam, Saudi Arabia; [457]Neurology Unit, Helsinki University Hospital, Helsinki, Finland; [458]School of Health Sciences, University of Melbourne, Melbourne, VIC, Australia; [459]Clinical Microbiology and Parasitology Unit, Dr. Zora Profozic Polyclinic, Zagreb, Croatia; [460]University Centre Varazdin, University North, Varazdin, Croatia; [461]Center for Innovation in Medical Education, Pomeranian Medical University, Szczecin, Poland; [462]Pomeranian Medical University, Szczecin, Poland; [463]Department of Propedeutics of Internal Diseases & Arterial Hypertension, Pomeranian Medical University, Szczecin, Poland; [464]Woman-Mother-Child Department, Lausanne University Hospital, Lausanne, Switzerland; [465]Pacific Institute for Research & Evaluation, Calverton, MD, USA; [466]School of Public Health, Curtin University, Perth, WA, Australia; [467]Internal Medicine Programme, Kyrgyz State Medical Academy, Bishkek, Kyrgyzstan; [468]Department of Atherosclerosis and Coronary Heart Disease, National Center of Cardiology and Internal Disease, Bishkek, Kyrgyzstan; [469]Research Center for Biochemistry and Nutrition in Metabolic Diseases, Kashan University of Medical Sciences, Kashan, Iran; [470]Institute of Addiction Research (ISFF), Frankfurt University of Applied Sciences, Frankfurt, Germany; [471]Biotechnology Research Center, Tabriz University of Medical Sciences, Tabriz, Iran; [472]Molecular Medicine Research Center, Tabriz University of Medical Sciences, Tabriz, Iran; [473]Department of Internal Medicine, King Saud University, Riyadh, Saudi Arabia; [474]Department of Forestry, Salahaddin University-Erbil, Erbil, Iraq; [475]Department of Medicine-Huddinge, Karolinska Institute, Stockholm, Sweden; [476]Health Systems and Policy Research Unit, Ahmadu Bello University, Zaria, Nigeria; [477]Faculty of Medical and Health Sciences, University of Auckland, Auckland, New Zealand; [478]Department of Epidemiology and Biostatistics, Lorestan University of Medical Sciences, Khorramabad, Iran; [479]Faculty of Life Sciences and Medicine, King's College London, London, UK; [480]Department of Medicine, TMSS Medical College, Bogura, Bangladesh; [481]Department of Medicine, Sofia Ismail Memorial Medical Centre, Bogura, Bangladesh; [482]Social Determinants of Health Research Center, Kurdistan University of Medical Sciences, Sanandaj, Iran; [483]Department of Epidemiology and

Biostatistics, Kurdistan University of Medical Sciences, Sanandaj, Iran; [484]Computer, Electrical, and Mathematical Sciences and Engineering Division, King Abdullah University of Science and Technology, Thuwal, Saudi Arabia; [485]International Laboratory for Air Quality and Health, Queensland University of Technology, Brisbane, QLD, Australia; [486]Gorgas Memorial Institute for Health Studies, Panama City, Panama; [487]Department of Cardiology and Cardiac Surgery, University of Warmia and Mazury, Olsztyn, Poland; [488]Department of Epidemiology and Biostatistics, Wuhan University, Wuhan, China; [489]School of Medical Sciences, Science University of Malaysia, Kubang Kerian, Malaysia; [490]Research and Analytics Department, Initiative for Financing Health and Human Development, Chennai, India; [491]Department of Research and Analytics, Bioinsilico Technologies, Chennai, India; [492]Cardiovascular Research Center, Kermanshah University of Medical Sciences, Kermanshah, Iran; [493]Kermanshah University of Medical Sciences, Kermanshah, Iran; [494]Suraj Eye Institute, Nagpur, India; [495]Department of Pharmacy Practice, Imam Abdulrahman Bin Faisal University, Dammam, Saudi Arabia; [496]Discipline of Social & Administrative Pharmacy, University of Science, Malaysia, Penang, Malaysia; [497]Mysore Medical College and Research Institute, Government Medical College, Mysore, India; [498]Department of Clinical Medicine, Federal University of Minas Gerais, Belo Horizonte, Brazil; [499]Clinical Hospital, Federal University of Minas Gerais, Belo Horizonte, Brazil; [500]Department of Forensic Medicine and Toxicology, Manipal Academy of Higher Education, Manipal, India; [501]Department of Pediatrics, Arak University of Medical Sciences, Arak, Iran; [502]The Oxford Martin Programme on Deep Medicine, University of Oxford, Oxford, UK; [503]Department of Anatomy and Embryology, Carol Davila University of Medicine and Pharmacy, Bucharest, Romania; [504]Cardio-Aid, Bucharest, Romania; [505]Bupa Clemton Park, Bupa, Sydney, NSW, Australia; [506]Institute for Global Health Innovations, Duy Tan University, Hanoi, Vietnam; [507]Department of Clinical Sciences, Lund University, Lund, Sweden; [508]Centre for Heart Rhythm Disorders, University of Adelaide, Adelaide, SA, Australia; [509]Division of Community Internal Medicine, University of British Columbia, Vancouver, BC, Canada; [510]Department of Neurobiology, Care Sciences and Society, Karolinska Institute, Huddinge, Sweden; [511]Department of Community Health and Primary Care, University of Lagos, Idi Araba, Nigeria; [512]Department of Family and Preventive Medicine, University of Utah, Salt Lake City, UT, USA; [513]Translational Health Research Institute, Western Sydney University, Sydney, NSW, Australia; [514]Department of Psychiatry and Behavioural Neurosciences, McMaster University, Hamilton, ON, Canada; [515]Department of Psychiatry, University of Lagos, Lagos, Nigeria; [516]Section of Sustainable Health, Umeå University, Umeå, Sweden; [517]Department of Medicine, Autonomous University of Madrid, Madrid, Spain; [518]Department of Nephrology and Hypertension, The Institute for Health Research Foundation Jiménez Díaz University Hospital, Madrid, Spain; [519]Henry M Jackson School of International Studies, University of Washington, Seattle, WA, USA; [520]Department of Forensic Medicine, Manipal Academy of Higher Education, Mangalore, India; [521]Department of Health Metrics, Center for Health Outcomes & Evaluation, Bucharest, Romania; [522]Department of Forensic Medicine & Toxicology, Pramukhswami Medical College, Anand, India; [523]Institute of Health Services Research, Yonsei University, Seoul, South Korea; [524]Division of Endocrinology, Mayo Clinic, Rochester, MN, USA; [525]Iran University of Medical Sciences, Tehran, Iran; [526]Department of Neurology and Public Health, Icahn School of Medicine at Mount Sinai, New York, NY, USA; [527]Research & Development Department, Kalinga Institute of Medical Sciences, Bhubaneswar, India; [528]Central Department of Public Health, Tribhuvan University, Kathmandu, Nepal; [529]Center for Research and Innovation, Ateneo De Manila University, Pasig City, Philippines; [530]Department of Development Studies, International Institute for Population Sciences, Mumbai, India; [531]Mario Negri Institute for Pharmacological Research, Bergamo, Italy; [532]Department of Cardiology, University of Bern, Bern, Switzerland; [533]Uro-oncology Research Center,

Tehran University of Medical Sciences, Tehran, Iran; [534]Medical College, Tairunnessa Memorial Medical College and Hospital, Gazipur, Bangladesh; [535]School of Public Health, University of Adelaide, Adelaide, SA, Australia; [536]Health Sciences Department, Muhammadiyah University of Surakarta, Sukoharjo, Indonesia; [537]Department of Chemistry, Sharif University of Technology, Tehran, Iran; [538]Biomedical Engineering Department, Amirkabir University of Technology, Tehran, Iran; [539]College of Medicine, University of Central Florida, Orlando, FL, USA; [540]Department of Immunology, Mazandaran University of Medical Sciences, Sari, Iran; [541]Molecular and Cell Biology Research Center, Mazandaran University of Medical Sciences, Sari, Iran; [542]Thalassemia and Hemoglobinopathy Research Center, Ahvaz Jundishapur University of Medical Sciences, Ahvaz, Iran; [543]Metabolomics and Genomics Research Center, Tehran University of Medical Sciences, Tehran, Iran; [544]Sina Trauma and Surgery Research Center, Tehran University of Medical Sciences, Tehran, Iran; [545]Department of Community Medicine, Maharishi Markandeshwar Medical College & Hospital, Solan, India; [546]School of Nursing and Healthcare Professions, Federation University Australia, Berwick, VIC, Australia; [547]School of Nursing and Midwifery, La Trobe University, Melbourne, VIC, Australia; [548]Department of Computer Science, Khazar University, Baku, Azerbaijan; [549]European Office for the Prevention and Control of Non-communicable Diseases, World Health Organization (WHO), Moscow, Russia; [550]Department of Cardiology, Emory University, Atlanta, GA, USA; [551]Department of Community Medicine, PSG Institute of Medical Sciences and Research, Coimbatore, India; [552]Department of Public Health, North South University, Dhaka, Bangladesh; [553]Department of Biostatistics and Epidemiology, University of Massachusetts Amherst, Amherst, MA, USA; [554]Department of Pharmacology, University of Colombo, Colombo, Sri Lanka; [555]Department of Oral Pathology, Srinivas Institute of Dental Sciences, Mangalore, India; [556]School of Health, Medical and Applied Sciences, CQ University, Sydney, NSW, Australia; [557]River Region Cardiology Associates, Montgomery, WV, USA; [558]Department of Computer Science, Boston University, Boston, MA, USA; [559]School of Social Sciences and Psychology, Western Sydney University, Penrith, NSW, Australia; [560]Translational Health Research Institute, Western Sydney University, Penrith, NSW, Australia; [561]Cardiovascular Diseases Research Center, Birjand University of Medical Sciences, Birjand, Iran; [562]Aboriginal Research Unit, South Australian Health and Medical Research Institute, Adelaide, SA, Australia; [563]Department of Clinical Research, Federal University of Uberlândia, Uberlândia, Brazil; [564]Department of Global Health and Population, Harvard University, Boston, MA, USA; [565]Center for Indigenous Health Research, Wuqu' Kawoq Maya Health Alliance, Tecpan, Guatemala; [566]Department of Neuroscience, University of Perugia, Perugia, Italy; [567]Department of Neurology, Rimini "Infermi" Hospital - AUSL Romagna, Rimini, Italy; [568]Golestan Research Center of Gastroenterology and Hepatology (GRCGH), Golestan University of Medical Sciences, Gorgan, Iran; [569]Department of Internal Medicine, University of Botswana, Gaborone, Botswana; [570]Department of Medical Pharmacology, Cairo University, Giza, Egypt; [571]Department of Epidemiology, Shahid Beheshti University of Medical Sciences, Tehran, Iran; [572]Department of Neurology, University of L'Aquila, L'Aquila, Italy; [573]Cardiac Rehabilitation Research Center, Isfahan University of Medical Sciences, Isfahan, Iran; [574]Department of Emergency Medicine, Shahid Beheshti University of Medical Sciences, Tehran, Iran; [575]Halal Research Center, Food and Drug Administration of the Islamic Republic of Iran, Tehran, Iran; [576]Neurogenic Inflammation Research Center, Mashhad University of Medical Sciences, Mashhad, Iran; [577]Department of Radiology, University of Southern California, Los Angeles, CA, USA; [578]Emergency Department, Brown University, Providence, RI, USA; [579]Department of Entomology, Ain Shams University, Cairo, Egypt; [580]Center for Clinical and Epidemiological Research, University of São Paulo, São Paulo, Brazil; [581]School of Public Health and Health Management, University of Belgrade, Belgrade, Serbia; [582]Isfahan Cardiovascular

Research Institute, Isfahan University of Medical Sciences, Isfahan, Iran; [583]Colorectal Research Center, Iran University of Medical Sciences, Tehran, Iran; [584]Population Health Research Institute, McMaster University, Hamilton, ON, Canada; [585]Department of Public Health Sciences, University of North Carolina at Charlotte, Charlotte, NC, USA; [586]Market Access, Bayer, Istanbul, Turkey; [587]School of Public Health and Community Medicine, University of New South Wales, Sydney, NSW, Australia; [588]Emergency Department, Manian Medical Centre, Erode, India; [589]Center for Biomedical Information Technology, Shenzhen Institutes of Advanced Technology, Shenzhen, China; [590]Health Policy Research Center, Shiraz University of Medical Sciences, Shiraz, Iran; [591]Department of Internal Medicine, Ziauddin University, Karachi, Pakistan; [592]Independent Consultant, Karachi, Pakistan; [593]Department of Clinical Research, University of Southern Denmark, Odense, Denmark; [594]Faculty of Caring Science, Work Life, and Social Welfare, University of Borås, Borås, Sweden; [595]Centre for Big Data Research in Health, University of New South Wales, Sydney, NSW, Australia; [596]Centre for Medical Informatics, University of Edinburgh, Edinburgh, UK; [597]Division of General Internal Medicine, Harvard University, Boston, MA, USA; [598]National Institute of Infectious Diseases, Tokyo, Japan; [599]Korea University, Seoul, South Korea; [600]College of Medicine, Yonsei University, Seoul, South Korea; [601]Finnish Institute of Occupational Health, Helsinki, Finland; [602]Research Executive Agency, European Commission, Brussels, Belgium; [603]School of Public Health, University of Haifa, Haifa, Israel; [604]The Cooper Institute, Dallas, TX, USA; [605]Department of Health Education and Health Promotion, Kermanshah University of Medical Sciences, Kermanshah, Iran; [606]School of Health, University of Technology Sydney, Sydney, NSW, Australia; [607]Department of Medicine, Dow University of Health Sciences, Karachi, Pakistan; [608]Department of Physical Education, Federal University of Santa Catarina, Florianopolis, Brazil; [609]School of Medicine, University of Alabama at Birmingham, Birmingham, AL, USA; [610]Medicine Service, US Department of Veterans Affairs (VA), Birmingham, AL, USA; [611]Department No.16, Moscow Research and Practical Centre on Addictions, Moscow, Russia; [612]Therapeutic Department, Balashiha Central Hospital, Balashikha, Russia; [613]Nursing Care Research Center, Semnan University of Medical Sciences, Semnan, Iran; [614]Occupational and Environmental Medicine Department, University of Gothenburg, Gothenburg, Sweden; [615]Department of Epidemiology & Biostatistics, The University of Western Ontario, London, ON, Canada; [616]Department of Population Health, Luxembourg Institute of Health, Strassen, Luxembourg; [617]Department of Statistics, Manonmaniam Sundaranar University, Tirunelveli, India; [618]National Institute of Epidemiology, Indian Council of Medical Research, Chennai, India; [619]Johns Hopkins University, Baltimore, MD, USA; [620]Department of Biomedical Sciences, Arba Minch University, Arba Minch, Ethiopia; [621]Nutrition and Clinical Services Division, International Centre for Diarrhoeal Disease Research, Bangladesh, Dhaka, Bangladesh; [622]Department of Public Health, Adigrat University, Adigrat, Ethiopia; [623]Health Education and Behavioral Science Unit, University of Gondar, Gondar, Ethiopia; [624]Division of Biostatistics and Epidemiology, Texas Tech University, El Paso, TX, USA; [625]Massachusetts Veterans Epidemiology Research and Information Center (MAVERIC) & CSP Coordinating Center, Harvard University, Boston, MA, USA; [626]Department of Public Health and Community Medicine, Central University of Kerala, Kasaragod, India; [627]Department of Medicine, Monash University, Melbourne, VIC, Australia; [628]Department of Anatomy, Dongguk University, Gyeongju, South Korea; [629]Department of Medicine, University of Calgary, Calgary, AB, Canada; [630]Nutritional Epidemiology Research Team (EREN), National Institute for Health and Medical Research (INSERM), Paris, France; [631]Health, Medicine and Human Biology, Sorbonne Paris Nord University, Bobigny, France; [632]Department of Pathology and Legal Medicine, University of São Paulo, Ribeirão Preto, Brazil; [633]Modestum LTD, London, UK; [634]Department of Community Medicine, All India Institute of Medical Sciences, Nagpur, India; [635]Rigshospitalet,

University of Copenhagen, Copenhagen, Denmark; [636]College of Medicine and Health Sciences, Bahir Dar University, Bahir Dar, Ethiopia; [637]Department of Vascular Surgery, Ludwig Maximilians University, Munich, Germany; [638]Department of Epidemiology & Biostatistics, Haramaya University, Haramaya, Ethiopia; [639]St. John of God Health Park, San Juan de Dios Sanitary Park, Barcelona, Spain; [640]Biomedical Research Networking Center for Mental Health Network (CiberSAM), Madrid, Spain; [641]Pediatric Cardiology, Rush University, Chicago, IL, USA; [642]Department of Cardiovascular, Endocrine-metabolic Diseases and Aging, National Institute of Health, Rome, Italy; [643]Kasturba Medical College, Manipal Academy of Higher Education, Mangalore, India; [644]Division of Cardiovascular Medicine, Harvard University, Boston, MA, USA; [645]Argentine Society of Medicine, Buenos Aires, Argentina; [646]Velez Sarsfield Hospital, Buenos Aires, Argentina; [647]UKK Institute, Tampere, Finland; [648]Raffles Neuroscience Centre, Raffles Hospital, Singapore, Singapore; [649]Yong Loo Lin School of Medicine, National University of Singapore, Singapore, Singapore; [650]Center of Excellence in Behavioral Medicine, Nguyen Tat Thanh University, Ho Chi Minh City, Vietnam; [651]Foundation University Medical College, Foundation University Islamabad, Islamabad, Pakistan; [652]School of Health Sciences, Wuhan University, Wuhan, China; [653]Department of Epidemiology and Biostatistics, George Washington University, Washington, DC, USA; [654]Cardiology Department, Royal Children's Hospital, Melbourne, VIC, Australia; [655]Critical Care and Neurosciences Theme, Murdoch Children’s Research Institute, Melbourne, VIC, Australia; [656]Competence Center of Mortality-Follow-Up of the German National Cohort, Federal Institute for Population Research, Wiesbaden, Germany; [657]Institute of Health and Society, University of Oslo, Oslo, Norway; [658]Department of Neurology, Technical University of Munich, Munich, Germany; [659]South African Medical Research Council, Cape Town, South Africa; [660]School of Public Health and Family Medicine, University of Cape Town, Cape Town, South Africa; [661]NIHR Biomedical Research Centre, Guy's and St.Thomas' Hospital and Kings College London, London, UK; [662]Research Centre for Generational Health and Ageing, University of Newcastle, Newcastle, NSW, Australia; [663]School of Medicine, Nanjing University, Nanjing, China; [664]Psychology Department, University of Sheffield, Sheffield, UK; [665]Research and Development Center for Health Services, University of Tsukuba, Tsukuba, Japan; [666]Graduate School of Medicine, Osaka University, Suita, Japan; [667]Global Health Research Center, Duke Kunshan University, Kunshan, China; [668]Duke Global Health Institute, Duke University, Durham, NC, USA; [669]Division of Cardiology, New York Medical College, Valhalla, NY, USA; [670]Division of Cardiology, Westchester Medical Center, Valhalla, NY, USA; [671]Department of Family Medicine and Community Health, Duke University, Durham, NC, USA; [672]Department of Public Health, Fujita Health University, Toyoake, Japan; [673]Department of Public Health and Health Systems, Nagoya University, Nagoya, Japan; [674]School of Nursing, Hawassa University, Hawassa, Ethiopia; [675]Department of Epidemiology and Biostatistics, University of Gondar, Gondar, Ethiopia; [676]Pediatrics Department, University of Jos, Jos, Nigeria; [677]Department of Pediatrics, Jos University Teaching Hospital, Jos, Nigeria; [678]Department of Neuropsychopharmacology, National Center of Neurology and Psychiatry, Kodaira, Japan; [679]Department of Public Health, Juntendo University, Tokyo, Japan; [680]Department of Health care Management and Economics, Urmia University of Medical Science, Urmia, Iran; [681]Department of Cardiology, Mother Hospital, Thrissur, India; [682]The School of Clinical Sciences at Monash Health, Monash University, Melbourne, VIC, Australia; [683]Maternal and Child Health Division, International Centre for Diarrhoeal Disease Research, Bangladesh, Dhaka, Bangladesh; [684]School of Rehabilitation Therapy, Queen's University, Kingston, ON, Canada; [685]Department of Neuroscience, Geisinger Health System, Danville, PA, USA; [686]Department of Neurology, University of Tennessee, Memphis, TN, USA; [687]Department of Radiology, Children's Hospital of Philadelphia, Philadelphia, PA, USA; [688]Department of Medicinal and

Pharmaceutical Chemistry, Shahid Beheshti University of Medical Sciences, Tehran, Iran; [689]Laboratory of Genetics and Genomics, Moscow Research and Practical Centre on Addictions, Moscow, Russia; [690]Addictology Department, Russian Medical Academy of Continuous Professional Education, Moscow, Russia; [691]Pediatrics Department, Russian Medical Academy of Continuous Professional Education, Moscow, Russia; [692]School of Medicine, Wuhan University, Wuhan, China; [693]School of Public Health, Wuhan University of Science and Technology, Wuhan, China; [694]Hubei Province Key Laboratory of Occupational Hazard Identification and Control, Wuhan University of Science and Technology, Wuhan, China; [695]Department of Environmental Health Sciences, University at Albany, Rensselaer, NY, USA; [696]Institute of Child and Adolescent Health, Peking University, Beijing, China; [697]Health Technology Assessment Unit, Department of Health Philippines, Manila, Philippines; [698]#MentalHealthPH, Inc., Quezon City, Philippines; [699]Icahn School of Medicine, Mount Sinai Hospital, New York, NY, USA; [700]Journal of the American College of Cardiology, Washington, DC, USA.

Authors' Contributions

Managing the estimation or publications process

Nicole K. DeCleene

Writing the first draft of the manuscript

Gregory A. Roth, George A. Mensah, Catherine O. Johnson, Giovanni Addolorato, Enrico Ammirati, Larry M. Baddour, Noel C. Barengo, Andrea Beaton, Emelia J. Benjamin, Catherine P. Benziger, Aime Bonny, Michael Brauer, Marianne Brodmann, Thomas J. Cahill, Jonathan R. Carapetis, Alberico L. Catapano, Sumeet Chugh, Leslie T. Cooper, Josef Coresh, Michael H. Criqui, Nicole K. DeCleene, Kim A. Eagle, Sophia Emmons-Bell, Valery L. Feigin, Joaquim Fernández-Sola, F Gerry R. Fowkes, Emmanuela Gakidou, Scott M. Grundy, Feng J. He, George Howard, Frank Hu, Lesley Inker, Ganesan Karthikeyan, Nicholas J. Kassebaum, Walter J. Koroshetz, Carl Lavie, Donald Lloyd-Jones, Hong S. Lu, Antonio Mirijello, Awoke T. Misganaw, Ali H. Mokdad, Andrew E. Moran, Paul Muntner, Jagat Narula, Bruce Neal, Mpiko Ntsekhe, Gláucia M. M. Oliveira, Catherine M. Otto, Mayowa O. Owolabi, Michael Pratt, Sanjay Rajagopalan, Marissa B. Reitsma, Antonio Luiz P. Ribeiro, Nancy A. Rigotti, Anthony Rodgers, Craig A. Sable, Saate S. Shakil, Karen Sliwa, Benjamin A. Stark, Johan Sundström, Patrick Timpel, Imad I. Tleyjeh, Marco Valgimigli, Theo Vos, Paul K. Whelton, Magdi Yacoub, Christopher J. L. Murray, and Valentin Fuster.

Primary responsibility for this manuscript focused on: applying analytical methods to produce estimates

Gregory A. Roth, Catherine O. Johnson, Sophia Emmons-Bell, Saate S. Shakil, and Benjamin A. Stark.

Providing data or critical feedback on data sources

Gregory A. Roth, George A. Mensah, Catherine O. Johnson, Giovanni Addolorato, Michael Brauer, Michael H. Criqui, Antonio Mirijello, Ali H. Mokdad, Mpiko Ntsekhe, Marco Valgimigli, Liesl J. Zuhlke, Abdelrahman I. Abushouk, Oladimeji M. Adebayo, Zanfina Ademi, Khashayar Afshari, Ashkan Afshin, Gina Agarwal, Sohail Ahmad, Muktar B. Ahmed, Budi Aji, Addis Aklilu, Chisom J. Akunna, Fares Alahdab, Sheikh M. Alif, Syed M. Aljunid, François Alla, Amir Almasi-Hashiani, Sami Almustanyir, Saeed Amini, Hubert Amu, Deanna Anderlini, Benny Antony, Lambert T. Appiah, Jalal Arabloo, Johan Ärnlöv, Marcel Ausloos, Muluken A. Ayza, Samad Azari, Ahad Bakhtiari, Maciej Banach, Palash C. Banik, Sanjay Basu,

Neeraj Bedi, Derrick A. Bennett, Adam E. Berman, Akshaya S. Bhagavathula, Boris Bikbov, Archith Boloor, Luisa C. Brant, Luis A. Cámera, Carlos Cantu-Brito, Juan J. Carrero, Carlos A. Castañeda-Orjuela, Ferrán Catalá-López, Vijay Kumar Chattu, Dinh-Toi Chu, Massimo Cirillo, Vera M. Costa, Omid Dadras, Xiaochen Dai, Albertino A. M. Damasceno, Lalit Dandona, Rakhi Dandona, Kairat Davletov, Meseret Derbew Molla, Rupak Desai, Samath D. Dharmaratne, Meghnath Dhimal, Mostafa Dianatinasab, Klara Dokova, Abdel Douiri, Bruce B. Duncan, Sanam Ebtehaj, Aziz Eftekhari, Nevine El Nahas, Atkilt E. Etisso, Ibtihal Fadhil, Emerito Jose A. Faraon, Farshad Farzadfar, Irina Filip, David Flood, Takeshi Fukumoto, Mohamed M. Gad, Shilpa Gaidhane, Jalaj Garg, Lemma Getacher, Alireza Ghajar, Ahmad Ghashghaee, Nermin Ghith, Simona Giampaoli, Elena V. Gnedovskaya, Mahaveer Golechha, Alessandra C. Goulart, Avirup Guha, Yuming Guo, Rajeev Gupta, Nima Hafezi-Nejad, Teklehaimanot G. Haile, Hamid Y. Hassen, Claudiu Herteliu, Mehdi Hosseinzadeh, Mowafa Househ, Charles U. Ibeneme, Segun E. Ibitoye, Usman Iqbal, Seyed Sina N. Irvani, Sheikh Mohammed Shariful Islam, Vardhmaan Jain, Tahereh Javaheri, Sathish Kumar Jayapal, Shubha Jayaram, Panniyammakal Jeemon, Jost B. Jonas, Jitendra Jonnagaddala, Farahnaz Joukar, Jacek J. Jozwiak, Mikk Jürisson, Tanvir Kahlon, André Karch, Ayele Semachew Kasa, Gbenga A. Kayode, Yousef S. Khader, Mohammad Khammarnia, Md Nuruzzaman Khan, Maseer Khan, Khaled Khatab, Gulam M. A. Kibria, Gyu Ri Kim, Sezer Kisa, Adnan Kisa, Kewal Krishan, Barthelemy Kuate Defo, Burcu Kucuk Bicer, G Anil Kumar, Dian Kusuma, Tea Lallukka, Savita Lasrado, Kate E. LeGrand, Shanshan Li, Stephen S. Lim, Lee-Ling Lim, Hualiang Lin, Xuefeng Liu, Stefan Lorkowski, Nirmal K. M, Azeem Majeed, Reza Malekzadeh, Navid Manafi, Mohammad Ali Mansournia, Lorenzo G. Mantovani, Manu R. Mathur, Suresh Mehata, Man Mohan Mehndiratta, Ritesh G. Menezes, Atte Meretoja, Bartosz Miazgowski, Erkin M. Mirrakhimov, Hamed Mirzaei, Babak Moazen, Masoud Moghadaszadeh, Dara K. Mohammad, Shafiu Mohammed, Mariam Molokhia, Ghobad Moradi, Sumaira Mubarik, Walter Muruet, Kamarul Imran Musa, Sreenivas Narasimha Swamy, Bruno R. Nascimento, Javad Nazari, Ruxandra I. Negoi, Huong L. T. Nguyen, Jean Jacques Noubiap, Christoph Nowak, Felix A. Ogbo, Andrew T. Olagunju, Alberto Ortiz, Jagadish Rao Padubidri, Adrian Pana, Songhomitra Panda-Jonas, Eun-Cheol Park, Mojtaba Parvizi, Fatemeh Pashazadeh Kan, Urvish K. Patel, Veincent Christian F. Pepito, Arokiasamy Perianayagam, Hai Q. Pham, Michael A. Piradov, Vivek Podder, Amir Radfar, Alireza Rafiei, Fakher Rahim, Vafa Rahimi-Movaghar, Mohammad Hifz Ur Rahman, Amir Masoud Rahmani, Ivo Rakovac, Pradhum Ram, Sowmya J Rao, Priya Rathi, Lal Rawal, Reza Rawassizadeh, Andre M. N. Renzaho, Seyed Mohammad Riahi, Leonardo Roever, Siamak Sabour, Hamideh Salimzadeh, Abdallah M. Samy, Milena M. Santric-Milicevic, Arash Sarveazad, Monika Sawhney, Maria I. Schmidt, Sadaf G. Sepanlou, Masood A. Shaikh, Jae Il Shin, Soraya Siabani, Diego A. S. Silva, Jasvinder A. Singh, Valentin Y. Skryabin, Anna A. Skryabina, Amin Soheili, Degena B. Tadesse, Masih Tajdini, Berhane F. Teklehaimanot, Mathilde Touvier, Marcos R. Tovani-Palone, Thomas C. Truelsen, Guesh M. Tsegay, Nikolaos Tsilimparis, Krishna Kishore Umapathi, Bhaskaran Unnikrishnan, Pascual R. Valdez, Tommi J. Vasankari, Narayanaswamy Venketasubramanian, Giang T. Vu, Isidora S. Vujcic, Yasir Waheed, Ronny Westerman, Charles S. Wiysonge, Befikadu Legesse Wubishet, Gelin Xu, Ali Yadollahpour, Christopher S. Yilgwan, Chuanhua Yu, Hasan Yusefzadeh, Afshin Zarghi, Mikhail S. Zastrozhin, Anasthasia Zastrozhina, Yves Miel H. Zuniga, Christopher J. L. Murray, and Valentin Fuster.

Developing methods or computational machinery

Gregory A. Roth, Catherine O. Johnson, Michael Brauer, Ali H. Mokdad, Ashkan Afshin, Muktar B. Ahmed, Hubert Amu, Samad Azari, Palash C. Banik, Akshaya S. Bhagavathula, David K. Cundiff, Meseret Derbew Molla, Mostafa Dianatinasab, Aziz Eftekhari, Jalaj Garg, Mowafa Househ, Sathish Kumar Jayapal,

Adnan Kisa, Masoud Moghadaszadeh, Lal Rawal, Seyed Mohammad Riahi, Abdallah M. Samy, Degena B. Tadesse, Ronny Westerman, and Christopher J. L. Murray.

Providing critical feedback on methods or results

Gregory A. Roth, George A. Mensah, Catherine O. Johnson, Giovanni Addolorato, Aime Bonny, Michael Brauer, Jonathan R. Carapetis, Alberico L. Catapano, Michael H. Criqui, Valery L. Feigin, Walter J. Koroshetz, Hong S. Lu, Antonio Mirijello, Ali H. Mokdad, Bruce Neal, Mpiko Ntsekhe, Theo Vos, Liesl J. Zuhlke, Alireza Abdi, Victor Aboyans, Woldu A. Abrha, Eman Abu-Gharbieh, Abdelrahman I. Abushouk, Dilaram Acharya, Tim Adair, Zanfina Ademi, Shailesh M. Advani, Khashayar Afshari, Ashkan Afshin, Gina Agarwal, Pradyumna Agasthi, Sohail Ahmad, Muktar B. Ahmed, Budi Aji, Yonas Akalu, Wuraola Akande-Sholabi, Addis Aklilu, Chisom J. Akunna, Fares Alahdab, Ayman Al-Eyadhy, Khalid F. Alhabib, Sheikh M. Alif, Vahid Alipour, Syed M. Aljunid, Amir Almasi-Hashiani, Sami Almustanyir, Rajaa M. Al-Raddadi, Saeed Amini, Hubert Amu, Dickson A. Amugsi, Robert Ancuceanu, Deanna Anderlini, Tudorel Andrei, Catalina Liliana Andrei, Alireza Ansari-Moghaddam, Zelalem A. Anteneh, Ippazio Cosimo Antonazzo, Benny Antony, Razique Anwer, Lambert T. Appiah, Jalal Arabloo, Johan Ärnlöv, Kurnia D. Artanti, Marcel Ausloos, Leticia Avila-Burgos, Asma T. Awan, Mamaru A. Awoke, Muluken A. Ayza, Samad Azari, Darshan B. B, Nafiseh Baheiraei, Atif A. Baig, Ahad Bakhtiari, Maciej Banach, Palash C. Banik, Emerson A. Baptista, Miguel A. Barboza, Lingkan Barua, Sanjay Basu, Derrick A. Bennett, Isabela M. Bensenor, Adam E. Berman, Yihienew M. Bezabih, Akshaya S. Bhagavathula, Sonu Bhaskar, Krittika Bhattacharyya, Ali Bijani, Mulugeta M. Birhanu, Archith Boloor, Luisa C. Brant, Hermann Brenner, Zahid A. Butt, Florentino Luciano Caetano dos Santos, Lucero Cahuana-Hurtado, Luis A. Cámera, Ismael R. Campos-Nonato, Josip Car, Ferrán Catalá-López, Ester Cerin, Jaykaran Charan, Vijay Kumar Chattu, Ken L. Chin, Jee-Young J. Choi, Dinh-Toi Chu, Sheng-Chia Chung, Massimo Cirillo, Sara Conti, David K. Cundiff, Omid Dadras, Baye Dagnew, Xiaochen Dai, Albertino A. M. Damasceno, Lalit Dandona, Rakhi Dandona, Kairat Davletov, Vanessa De la Cruz-Góngora, Fernando P. De la Hoz, Jan-Walter De Neve, Meseret Derbew Molla, Behailu T. Derseh, Rupak Desai, Günther Deuschl, Samath D. Dharmaratne, Raja Ram Dhungana, Mostafa Dianatinasab, Daniel Diaz, Shirin Djalalinia, Abdel Douiri, Bruce B. Duncan, Arielle W. Eagan, Sanam Ebtehaj, Aziz Eftekhari, Sahar Eftekharzadeh, Michael Ekholuenetale, Nevine El Nahas, Islam Y. Elgendy, Muhammed Elhadi, Sadaf Esteghamati, Oghenowede Eyawo, Ibtihal Fadhil, Emerito Jose A. Faraon, Pawan S. Faris, Farshad Farzadfar, Carlota Fernandez Prendes, Irina Filip, Florian Fischer, Takeshi Fukumoto, Mohamed M. Gad, Shilpa Gaidhane, Morsaleh Ganji, Jalaj Garg, Abadi K. Gebre, Birhan G. Gebregiorgis, Kidane Z. Gebregzabiher, Lemma Getacher, Abera Getachew Obsa, Alireza Ghajar, Ahmad Ghashghaee, Nermin Ghith, Syed Amir Gilani, Paramjit S. Gill, Elena V. Gnedovskaya, Mahaveer Golechha, Kebebe B. Gonfa, Amir Hossein Goudarzian, Alessandra C. Goulart, Avirup Guha, Yuming Guo, Rajeev Gupta, Vladimir Hachinski, Nima Hafezi-Nejad, Teklehaimanot G. Haile, Randah R. Hamadeh, Samer Hamidi, Arief Hargono, Risky K. Hartono, Abdiwahab Hashi, Shoaib Hassan, Hamid Y. Hassen, Rasmus J. Havmoeller, Khezar Hayat, Golnaz Heidari, Claudiu Herteliu, Ramesh Holla, Mehdi Hosseinzadeh, Mihaela Hostiuc, Sorin Hostiuc, Mowafa Househ, Ayesha Humayun, Charles U. Ibeneme, Segun E. Ibitoye, Olayinka S. Ilesanmi, Irena M. Ilic, Milena D. Ilic, Usman Iqbal, Seyed Sina N. Irvani, Sheikh Mohammed Shariful Islam, Rakibul M. Islam, Hiroyasu Iso, Masao Iwagami, Vardhmaan Jain, Tahereh Javaheri, Sathish Kumar Jayapal, Shubha Jayaram, Ranil Jayawardena, Panniyammakal Jeemon, Ravi P. Jha, Jost B. Jonas, Jitendra Jonnagaddala, Farahnaz Joukar, Jacek J. Jozwiak, Mikk Jürisson, Ali Kabir, Tanvir Kahlon, Rizwan Kalani, Rohollah Kalhor, Ashwin Kamath, Ibrahim Kamel, Himal Kandel, Amit Kandel, André Karch, Ayele Semachew Kasa, Patrick D.M.C. Katoto, Gbenga A. Kayode, Yousef S. Khader, Mohammad Khammarnia, Muhammad S. Khan, Md Nuruzzaman Khan, Maseer Khan, Ejaz A. Khan,

Khaled Khatab, Gulam M. A. Kibria, Yun Jin Kim, Gyu Ri Kim, Ruth W. Kimokoti, Sezer Kisa, Adnan Kisa, Mika Kivimäki, Dhaval Kolte, Ali Koolivand, Sindhura Lakshmi Koulmane Laxminarayana, Ai Koyanagi, Kewal Krishan, Vijay Krishnamoorthy, Barthelemy Kuate Defo, Vaman Kulkarni, G Anil Kumar, Nithin Kumar, Om P. Kurmi, Dian Kusuma, Gene F. Kwan, Tea Lallukka, Qing Lan, Savita Lasrado, Zohra S. Lassi, Paolo Lauriola, Wayne R. Lawrence, Avula Laxmaiah, Kate E. LeGrand, Ming-Chieh Li, Bingyu Li, Shanshan Li, Lee-Ling Lim, Hualiang Lin, Ziqiang Lin, Ro-Ting Lin, Xuefeng Liu, Alan D. Lopez, Stefan Lorkowski, Nirmal K. M, Fabiana Madotto, Morteza Mahmoudi, Azeem Majeed, Reza Malekzadeh, Ahmad A. Malik, Abdullah A. Mamun, Navid Manafi, Mohammad Ali Mansournia, Lorenzo G. Mantovani, Santi Martini, Manu R. Mathur, Giampiero Mazzaglia, Suresh Mehata, Toni Meier, Ritesh G. Menezes, Atte Meretoja, Tomasz Miazgowski, Irmina Maria Michalek, Ted R. Miller, Erkin M. Mirrakhimov, Babak Moazen, Masoud Moghadaszadeh, Yousef Mohammad, Dara K. Mohammad, Shafiu Mohammed, Mohammed A. Mohammed, Yaser Mokhayeri, Mariam Molokhia, Ghobad Moradi, Rahmatollah Moradzadeh, Lidia Morawska, Jakub Morze, Sumaira Mubarik, Walter Muruet, Kamarul Imran Musa, Ahamarshan J. Nagarajan, Vinay Nangia, Atta Abbas Naqvi, Sreenivas Narasimha Swamy, Bruno R. Nascimento, Javad Nazari, Milad Nazarzadeh, Ruxandra I. Negoi, Sandhya Neupane Kandel, Huong L. T. Nguyen, Molly R. Nixon, Bo Norrving, Jean Jacques Noubiap, Christoph Nowak, Oluwakemi O. Odukoya, Felix A. Ogbo, Andrew T. Olagunju, Hans Orru, Alberto Ortiz, Samuel M. Ostroff, Jagadish Rao Padubidri, Raffaele Palladino, Adrian Pana, Songhomitra Panda-Jonas, Utsav Parekh, Eun-Cheol Park, Mojtaba Parvizi, Fatemeh Pashazadeh Kan, Urvish K. Patel, Mona Pathak, Rajan Paudel, Veincent Christian F. Pepito, Arokiasamy Perianayagam, Hai Q. Pham, Michael A. Piradov, Vivek Podder, Akram Pourshams, Dimas R. A. Pribadi, Navid Rabiee, Mohammad Rabiee, Amir Radfar, Alireza Rafiei, Fakher Rahim, Vafa Rahimi-Movaghar, Mohammad Hifz Ur Rahman, Muhammad Aziz Rahman, Amir Masoud Rahmani, Ivo Rakovac, Pradhum Ram, Juwel Rana, Priyanga Ranasinghe, Sowmya J Rao, Priya Rathi, Lal Rawal, Wasiq F. Rawasia, Reza Rawassizadeh, Andre M. N. Renzaho, Aziz Rezapour, Seyed Mohammad Riahi, Leonardo Roever, Gholamreza Roshandel, Godfrey M. Rwegerera, Maha M. Saber-Ayad, Siamak Sabour, Sahar Saeedi Moghaddam, Saeed Safari, Sana Salehi, Mehrnoosh Samaei, Abdallah M. Samy, Itamar S. Santos, Milena M. Santric-Milicevic, Thirunavukkarasu Sathish, Monika Sawhney, Maria I. Schmidt, Aletta E. Schutte, Subramanian Senthilkumaran, Sadaf G. Sepanlou, Feng Sha, Saeed Shahabi, Izza Shahid, Masood A. Shaikh, Mahdi Shamali, Md Shajedur Rahman Shawon, Aziz Sheikh, Mika Shigematsu, Min-Jeong Shin, Jae Il Shin, Rahman Shiri, Ivy Shiue, Kerem Shuval, Tariq J. Siddiqi, Jasvinder A. Singh, Ambrish Singh, Valentin Y. Skryabin, Anna A. Skryabina, Amin Soheili, Emma E. Spurlock, Leo Stockfelt, Stefan Stortecky, Saverio Stranges, Rizwan Suliankatchi Abdulkader, Hooman Tadbiri, Eyayou G. Tadesse, Degena B. Tadesse, Masih Tajdini, Md Tariqujjaman, Mohamad-Hani Temsah, Ayenew K. Tesema, Bhaskar Thakur, Rekha Thapar, Binod Timalsina, Marcello Tonelli, Mathilde Touvier, Marcos R. Tovani-Palone, Avnish Tripathi, Jaya P. Tripathy, Thomas C. Truelsen, Guesh M. Tsegay, Gebiyaw W. Tsegaye, Nikolaos Tsilimparis, Biruk S. Tusa, Stefanos Tyrovolas, Krishna Kishore Umapathi, Bhaskaran Unnikrishnan, Muhammad S. Usman, Muthiah Vaduganathan, Pascual R. Valdez, Diana Z. Velazquez, Narayanaswamy Venketasubramanian, Giang T. Vu, Yasir Waheed, Yanzhong Wang, Jingkai Wei, Abrha H. Weldemariam, Ronny Westerman, Andrea S. Winkler, Charles D. A. Wolfe, Befikadu Legesse Wubishet, Gelin Xu, Ali Yadollahpour, Kazumasa Yamagishi, Lijing L. Yan, Yuichiro Yano, Hiroshi Yatsuya, Christopher S. Yilgwan, Naohiro Yonemoto, Chuanhua Yu, Sojib Bin Zaman, Maryam Zamanian, Alireza Zandifar, Mikhail S. Zastrozhin, Anasthasia Zastrozhina, Yunquan Zhang, Wangjian Zhang, Zhiyong Zou, Yves Miel H. Zuniga, and Christopher J. L. Murray.

Drafting the work or revising it critically for important intellectual content

Gregory A. Roth, George A. Mensah, Catherine O. Johnson, Giovanni Addolorato, Enrico Ammirati, Larry M. Baddour, Noel C. Barengo, Andrea Beaton, Emelia J. Benjamin, Catherine P. Benziger, Aime Bonny, Michael Brauer, Marianne Brodmann, Thomas J. Cahill, Jonathan R. Carapetis, Alberico L. Catapano, Sumeet Chugh, Leslie T. Cooper, Josef Coresh, Michael H. Criqui, Nicole K. DeCleene, Kim A. Eagle, Sophia Emmons-Bell, Valery L. Feigin, Joaquim Fernández-Sola, F Gerry R. Fowkes, Emmanuela Gakidou, Scott M. Grundy, Feng J. He, George Howard, Frank Hu, Lesley Inker, Ganesan Karthikeyan, Nicholas J. Kassebaum, Walter J. Koroshetz, Carl Lavie, Donald Lloyd-Jones, Hong S. Lu, Antonio Mirijello, Awoke T. Misganaw, Ali H. Mokdad, Andrew E. Moran, Paul Muntner, Jagat Narula, Bruce Neal, Mpiko Ntsekhe, Gláucia M. M. Oliveira, Catherine M. Otto, Mayowa O. Owolabi, Michael Pratt, Sanjay Rajagopalan, Marissa B. Reitsma, Antonio Luiz P. Ribeiro, Nancy A. Rigotti, Anthony Rodgers, Craig A. Sable, Saate S. Shakil, Karen Sliwa, Benjamin A. Stark, Johan Sundström, Patrick Timpel, Imad I. Tleyjeh, Marco Valgimigli, Theo Vos, Paul K.. Whelton, Magdi Yacoub, Liesl J. Zuhlke, Mohsen Abbasi-Kangevari, Alireza Abdi, Aidin Abedi, Victor Aboyans, Eman Abu-Gharbieh, Abdelrahman I. Abushouk, Dilaram Acharya, Shailesh M. Advani, Gina Agarwal, Pradyumna Agasthi, Sohail Ahmad, Sepideh Ahmadi, Muktar B. Ahmed, Budi Aji, Yonas Akalu, Fares Alahdab, Khalid F. Alhabib, Amir Almasi-Hashiani, Adeladza K. Amegah, Saeed Amini, Arya Aminorroaya, Hubert Amu, Dickson A. Amugsi, Robert Ancuceanu, Deanna Anderlini, Zelalem A. Anteneh, Ippazio Cosimo Antonazzo, Benny Antony, Jalal Arabloo, Johan Ärnlöv, Zerihun Ataro, Marcel Ausloos, Asma T. Awan, Henok T. Ayele, Darshan B. B, Atif A. Baig, Maciej Banach, Emerson A. Baptista, Miguel A. Barboza, Lingkan Barua, Sanjay Basu, Neeraj Bedi, Yannick Béjot, Derrick A. Bennett, Adam E. Berman, Yihienew M. Bezabih, Akshaya S. Bhagavathula, Sonu Bhaskar, Krittika Bhattacharyya, Boris Bikbov, Luisa C. Brant, Hermann Brenner, Nikolay I. Briko, Florentino Luciano Caetano dos Santos, Leah E. Cahill, Lucero Cahuana-Hurtado, Ismael R. Campos-Nonato, Carlos Cantu-Brito, Josip Car, Juan J. Carrero, Felix Carvalho, Carlos A. Castañeda-Orjuela, Ferrán Catalá-López, Ester Cerin, Vijay Kumar Chattu, Simiao Chen, Jee-Young J. Choi, Sean Coffey, Sara Conti, Vera M. Costa, Baye Dagnew, Albertino A. M. Damasceno, Vanessa De la Cruz-Góngora, Fernando P. De la Hoz, Jan-Walter De Neve, Edgar Denova-Gutiérrez, Meseret Derbew Molla, Behailu T. Derseh, Rupak Desai, Günther Deuschl, Samath D. Dharmaratne, Meghnath Dhimal, Daniel Diaz, Klara Dokova, Abdel Douiri, Andre R. Duraes, Arielle W. Eagan, Aziz Eftekhari, Sahar Eftekharzadeh, Islam Y. Elgendy, Muhammed Elhadi, Shaimaa I. El-Jaafary, Oghenowede Eyawo, Ibtihal Fadhil, Medhat Farwati, Eduarda Fernandes, Carlota Fernandez Prendes, Pietro Ferrara, Irina Filip, Florian Fischer, David Flood, Takeshi Fukumoto, Mohamed M. Gad, Shilpa Gaidhane, Jalaj Garg, Birhan G. Gebregiorgis, Gebreamlak G. Gebremeskel, Lemma Getacher, Abera Getachew Obsa, Ahmad Ghashghaee, Nermin Ghith, Paramjit S. Gill, Richard F. Gillum, Ekaterina V. Glushkova, Elena V. Gnedovskaya, Alessandra C. Goulart, Jenny S. Guadamuz, Rajeev Gupta, Nima Hafezi-Nejad, Graeme J. Hankey, Maryam Hashemian, Abdiwahab Hashi, Shoaib Hassan, Hamid Y. Hassen, Rasmus J. Havmoeller, Simon I. Hay, Golnaz Heidari, Claudiu Herteliu, Ramesh Holla, Mostafa Hosseini, Mihaela Hostiuc, Sorin Hostiuc, Mowafa Househ, Junjie Huang, Ayesha Humayun, Ivo Iavicoli, Charles U. Ibeneme, Segun E. Ibitoye, Olayinka S. Ilesanmi, Irena M. Ilic, Milena D. Ilic, Seyed Sina N. Irvani, Sheikh Mohammed Shariful Islam, Rakibul M. Islam, Hiroyasu Iso, Vardhmaan Jain, Sathish Kumar Jayapal, Shubha Jayaram, Panniyammakal Jeemon, Ravi P. Jha, Jost B. Jonas, Jacek J. Jozwiak, Ali Kabir, Tanvir Kahlon, Rizwan Kalani, Himal Kandel, Amit Kandel, André Karch, Ayele Semachew Kasa, Patrick D.M.C. Katoto, Gbenga A. Kayode, Yousef S. Khader, Mohammad Khammarnia, Muhammad S. Khan, Md Nuruzzaman Khan, Maseer Khan, Ejaz A. Khan, Khaled Khatab, Gulam M. A. Kibria, Yun Jin Kim, Gyu Ri Kim, Sezer Kisa, Adnan Kisa, Mika Kivimäki, Dhaval Kolte, Vladimir A. Korshunov, Sindhura Lakshmi Koulmane Laxminarayana, Ai Koyanagi, Kewal Krishan, Vijay Krishnamoorthy, Barthelemy Kuate Defo,

Vaman Kulkarni, Om P. Kurmi, Dian Kusuma, Gene F. Kwan, Carlo La Vecchia, Ben Lacey, Tea Lallukka, Savita Lasrado, Kate E. LeGrand, Lee-Ling Lim, Alan D. Lopez, Stefan Lorkowski, Paulo A. Lotufo, Alessandra Lugo, Nirmal K. M, Azeem Majeed, Reza Malekzadeh, Ahmad A. Malik, Abdullah A. Mamun, Navid Manafi, Mohammad Ali Mansournia, Lorenzo G. Mantovani, Giampiero Mazzaglia, Toni Meier, Ritesh G. Menezes, Atte Meretoja, Tomislav Mestrovic, Irmina Maria Michalek, Ted R. Miller, Babak Moazen, Masoud Moghadaszadeh, Yousef Mohammad, Dara K. Mohammad, Shafiu Mohammed, Mariam Molokhia, Ahmed A. Montasir, Rahmatollah Moradzadeh, Paula Moraga, Ilais Moreno Velásquez, Jakub Morze, Sumaira Mubarik, Kamarul Imran Musa, Ahamarshan J. Nagarajan, Mahdi Nalini, Vinay Nangia, Sreenivas Narasimha Swamy, Bruno R. Nascimento, Vinod C. Nayak, Javad Nazari, Ruxandra I. Negoi, Sandhya Neupane Kandel, Huong L. T. Nguyen, Molly R. Nixon, Bo Norrving, Jean Jacques Noubiap, Brice E. Nouthe, Christoph Nowak, Oluwakemi O. Odukoya, Felix A. Ogbo, Andrew T. Olagunju, Hans Orru, Alberto Ortiz, Jagadish Rao Padubidri, Raffaele Palladino, Adrian Pana, Songhomitra Panda-Jonas, Utsav Parekh, Eun-Cheol Park, Fatemeh Pashazadeh Kan, Urvish K. Patel, Mona Pathak, Veincent Christian F. Pepito, Norberto Perico, Hai Q. Pham, Thomas Pilgrim, Michael A. Piradov, Farhad Pishgar, Vivek Podder, Roman V. Polibin, Amir Radfar, Alireza Rafiei, Fakher Rahim, Vafa Rahimi-Movaghar, Mohammad Hifz Ur Rahman, Ivo Rakovac, Pradhum Ram, Sudha Ramalingam, Juwel Rana, Priyanga Ranasinghe, Sowmya J Rao, Lal Rawal, Wasiq F. Rawasia, Giuseppe Remuzzi, Andre M. N. Renzaho, Seyed Mohammad Riahi, Ross L. Roberts-Thomson, Leonardo Roever, Peter Rohloff, Michele Romoli, Gholamreza Roshandel, Godfrey M. Rwegerera, Seyedmohammad Saadatagah, Maha M. Saber-Ayad, Siamak Sabour, Simona Sacco, Masoumeh Sadeghi, Amirhossein Sahebkar, Sana Salehi, Mehrnoosh Samaei, Abdallah M. Samy, Itamar S. Santos, Milena M. Santric-Milicevic, Nizal Sarrafzadegan, Arash Sarveazad, Thirunavukkarasu Sathish, Mete Saylan, Aletta E. Schutte, Sadaf G. Sepanlou, Saeed Shahabi, Izza Shahid, Masood A. Shaikh, Mahdi Shamali, Morteza Shamsizadeh, Md Shajedur Rahman Shawon, Aziz Sheikh, Mika Shigematsu, Jae Il Shin, Kerem Shuval, Tariq J. Siddiqi, Diego A. S. Silva, Jasvinder A. Singh, Anna A. Skryabina, Amin Soheili, Leo Stockfelt, Stefan Stortecky, Saverio Stranges, Eyayou G. Tadesse, Degena B. Tadesse, Mohamad-Hani Temsah, Bhaskar Thakur, Kavumpurathu R. Thankappan, Amanda G. Thrift, Marcello Tonelli, Mathilde Touvier, Marcos R. Tovani-Palone, Jaya P. Tripathy, Thomas C. Truelsen, Guesh M. Tsegay, Nikolaos Tsilimparis, Krishna Kishore Umapathi, Brigid Unim, Bhaskaran Unnikrishnan, Muhammad S. Usman, Muthiah Vaduganathan, Tommi J. Vasankari, Diana Z. Velazquez, Narayanaswamy Venketasubramanian, Giang T. Vu, Isidora S. Vujcic, Yasir Waheed, Yanzhong Wang, Fang Wang, Robert G. Weintraub, Ronny Westerman, Andrea S. Winkler, Charles S. Wiysonge, Befikadu Legesse Wubishet, Ali Yadollahpour, Kazumasa Yamagishi, Lijing L. Yan, Srikanth Yandrapalli, Tomas Y. Yeheyis, Yigizie Yeshaw, Christopher S. Yilgwan, Naohiro Yonemoto, Geevar Zachariah, Muhammed S. Zaman, Maryam Zamanian, Ramin Zand, Alireza Zandifar, Mikhail S. Zastrozhin, Anasthasia Zastrozhina, Zhi-Jiang Zhang, Chenwen Zhong, Christopher J. L. Murray, and Valentin Fuster.

Managing the overall research enterprise

Gregory A. Roth, George A. Mensah, Catherine O. Johnson, Michael Brauer, Ali H. Mokdad, Theo Vos, Ashkan Afshin, Lalit Dandona, Simon I. Hay, Kate E. LeGrand, Alan D. Lopez, and Christopher J. L. Murray.

Portions of this appendix have been reproduced or adapted from Vos et al. (1), Murray et al. (2), GBD 2017 Cause of Death Collaborators (3), GBD 2017 Disease and Injury Incidence and Prevalence Collaborators (4), Kyu et al. (5), and GBD 2017 Risk Factor Collaborators (6). References are provided for reproduced sections.

Contents

Diseases and Injuries Methods 26

- Overview 26
- Geographical units, age groups, time periods, and cause levels 26
- Data 26
- Data processing 27
- Modelling 28

Risk Factors Methods 29

- Overview 29
- Geographical units, age groups, and time periods 29
- GBD risk factor hierarchy 29
- Determining the inclusion of risk–outcome pairs in GBD 29
- Estimating relative risk as a function of exposure for each risk–outcome pair 30
- Estimation of the distribution of exposure for each risk by age-sex-location-year 30
- Determining the TMREL 31
- Estimation of the population attributable fraction and attributable burden 31
- Estimating the PAF and attributable burden for combinations of risk factors 32
- Risk-deleted death rates 32

CoD cause-specific modelling descriptions 32

- Cardiovascular Diseases 32
- Ischaemic Heart Disease 34
- Stroke 36
- Ischaemic Stroke 38
- Intracerebral haemorrhage 40
- Subarachnoid haemorrhage 42
- Hypertensive Heart Disease 43
- Congenital birth defects: neural tube defects, congenital heart anomalies, orofacial clefts, Down syndrome, Turner syndrome, Klinefelter syndrome, other chromosomal disorders, congenital musculoskeletal anomalies, urogenital congenital anomalies, digestive congenital anomalies, and other congenital birth defects 45

Rheumatic heart disease........52
Cardiomyopathy and Myocarditis........53
Myocarditis........55
Other cardiomyopathy........56
Alcoholic Cardiomyopathy........57
Atrial Fibrillation and Flutter........59
Aortic Aneurysm........63
Non-rheumatic valvular heart disease: Non-rheumatic calcific aortic valve disease, non-rheumatic degenerative mitral valve disease, and other non-rheumatic valvular heart diseases........65
Endocarditis........67
Peripheral artery disease........69
Non-fatal cause-specific modelling descriptions........71
Ischaemic heart disease........71
Ischaemic Stroke, Intracerebral Haemorrhage, and Subarachnoid Haemorrhage........81
Congenital birth defects........90
Rheumatic Heart Disease........106
Acute Myocarditis........112
Atrial Fibrillation and Flutter........115
Non-rheumatic valvular heart diseases: Calcific aortic valve disease, degenerative mitral valve disease, and other non-rheumatic valve disease........120
Acute Endocarditis........130
Peripheral arterial disease........132
Risk-specific modelling descriptions........136
High systolic blood pressure........136
High fasting plasma glucose........145
High LDL cholesterol........150
High body-mass index........156
Kidney dysfunction........164
Ambient particulate matter pollution........170
Household air pollution........211
Smoking........218
Secondhand smoke........223
Chewing tobacco........226
Dietary risks........229

Low physical activity 238
References 242

Diseases and Injuries Methods

Overview

The general approach to estimating causes of death and disease incidence and prevalence for GBD 2019 is the same as for GBD 2017 (3, 4). Here, we provide an overview of the methods, with an emphasis on the main methodology changes since GBD 2017.

For each iteration of GBD, the estimates for the whole time series are updated on the basis of addition of new data and change in methods where appropriate. Thus, the GBD 2019 results supersede those from previous rounds of GBD.

Geographical units, age groups, time periods, and cause levels

GBD 2019 estimated each epidemiological quantity of interest—incidence, prevalence, mortality, years lived with disability (YLDs), years of life lost (YLLs), and disability-adjusted life-years (DALYs)—for 23 age groups; males, females, and both sexes combined; and 204 countries and territories that were grouped into 21 regions and seven super-regions. For GBD 2019, nine countries and territories (Cook Islands, Monaco, San Marino, Nauru, Niue, Palau, Saint Kitts and Nevis, Tokelau, and Tuvalu) were added, such that the GBD location hierarchy now includes all WHO member states. GBD 2019 includes subnational analyses for Italy, Nigeria, Pakistan, the Philippines, and Poland, and 16 countries previously estimated at subnational levels (Brazil, China, Ethiopia, India, Indonesia, Iran, Japan, Kenya, Mexico, New Zealand, Norway, Russia, South Africa, Sweden, the UK, and the USA). All subnational analyses are at the first level of administrative organisation within each country except for New Zealand (by Māori ethnicity), Sweden (by Stockholm and non-Stockholm), the UK (by local government authorities), and the Philippines (by province). At the most detailed spatial resolution, we generated estimates for 990 locations. The GBD diseases and injuries analytical framework generated estimates for every year from 1990 to 2019.

Diseases and injuries were organised into a levelled cause hierarchy from the three broadest causes of death and disability at Level 1 to the most specific causes at Level 4. Within the three Level 1 causes—communicable, maternal, neonatal, and nutritional diseases; non-communicable diseases; and injuries—there are 22 Level 2 causes, 174 Level 3 causes, and 301 Level 4 causes (including 131 level 3 causes that are not further disaggregated at Level 4). 364 total causes are non-fatal and 286 are fatal.

Data

The GBD estimation process is based on identifying multiple relevant data sources for each disease or injury including censuses, household surveys, civil registration and vital statistics, disease registries, health service use, air pollution monitors, satellite imaging, disease notifications, and other sources. Each of these types of data are identified from systematic review of published studies, searches of government and international organisation websites, published reports, primary data sources such as the Demographic and Health Surveys, and contributions of datasets by GBD collaborators. 86,249 sources were used in this analysis, including 19,354 sources reporting deaths, 31,499 reporting

incidence, 19,773 reporting prevalence, and 26,631 reporting other metrics. Each newly identified and obtained data source is given a unique identifier by a team of librarians and included in the Global Health Data Exchange (GHDx; http://ghdx.healthdata.org/). The GHDx makes publicly available the metadata for each source included in GBD as well as the data, where allowed by the data provider. Readers can use the GHDx source (http://ghdx.healthdata.org/gbd-2019/data-input-sources) tool to identify which sources were used for estimating any disease or injury outcome in any given location.

Data processing

A crucial step in the GBD analytical process is correcting for known bias by redistributing deaths from unspecified codes to more specific disease categories, and by adjusting data with alternative case definitions or measurement methods to the reference method. We highlight several major changes in data processing that in some cases have affected GBD results.

Cause of death redistribution

Vital registration with medical certification of cause of death is a crucial resource for the GBD cause of death analysis in many countries. Cause of death data obtained using various revisions of the International Classification of Diseases and Injuries (ICD) (7) were mapped to the GBD cause list. Many deaths, however, are assigned to causes that cannot be the underlying cause of death (eg, cardiopulmonary failure) or are inadequately specified (eg, injury from undetermined intent). These deaths were reassigned to the most probable underlying causes of death as part of the data processing for GBD. Redistribution algorithms can be divided into three categories: proportionate redistribution, fixed proportion redistribution based on published studies or expert judgment, or statistical algorithms. For GBD 2019, data for 116 million deaths attributed to multiple causes were analysed to produce more empirical redistribution algorithms for sepsis (8), heart failure, pulmonary embolism, acute kidney injury, hepatic failure, acute respiratory failure, pneumonitis, and five intermediate causes (hydrocephalus, toxic encephalopathy, compression of brain, encephalopathy, and cerebral oedema) in the central nervous system. To redistribute unspecified injuries, we used a method similar to that of intermediate cause redistribution, using the pattern of the nature of injury codes in the causal chain where the ICD codes X59 ("exposure to unspecified factor") and Y34 ("unspecified event, undetermined intent") and GBD injury causes were the underlying cause of death. These new algorithms led to important changes in the causes to which these intermediate outcomes were redistributed. Additionally, data on deaths from diabetes and stroke lack the detail on subtype in many countries; we ran regressions on vital registration data with at least 50% of deaths coded specifically to type 1 or 2 diabetes and ischaemic, haemorrhagic, or subarachnoid stroke to predict deaths by these subtypes when these were coded to unspecified diabetes or stroke.

Correcting for non-reference case definitions or measurement methods

In previous cycles of GBD, data reported using alternative case definitions or measurement methods were corrected to the reference definition or measurement method primarily as part of the Bayesian meta-regression models. For example, in DisMod-MR, the population data were simultaneously modelled as a function of country covariates for variation in true rates and as a function of indicator variables capturing alternative measurement methods. To enhance transparency and to standardise and improve methods in GBD 2019, we estimated correction factors for alternative case definitions or measurement methods using network meta-regression, including only data where two methods were assessed in the same location–time period or in the exact same population. This included validation

studies where two methods had been compared in populations that were not necessarily random samples of the general population.

Clinical informatics

Clinical informatics data include inpatient admissions, outpatient (including general practitioner) visits, and health insurance claims. Several data processing steps were undertaken. Inpatient hospital data with a single diagnosis only were adjusted to account for non-primary diagnoses as well as outpatient care. For each GBD cause that used clinical data, ratios of non-primary to primary diagnosis rates were extracted from claims in the USA, Taiwan (province of China), New Zealand, and the Philippines, as well as USA Healthcare Cost and Utilization Project inpatient data. Ratios of outpatient to inpatient care for each cause were extracted from claims data from the USA and Taiwan (province of China). The log of the ratios for each cause were modelled by age and sex using MR-BRT (Meta-Regression-Bayesian Regularised Trimmed), the Bayesian meta-regression tool. To account for the incomplete health-care access in populations where not every person with a disease or injury would be accounted for in administrative clinical records, we transformed the adjusted admission rates using a scalar derived from the Healthcare Access and Quality Index (9). We used this approach to produce adjusted, standardised clinical data inputs.

Modelling

For most diseases and injuries, processed data are modelled using standardised tools to generate estimates of each quantity of interest by age, sex, location, and year. There are three main standardised tools: Cause of Death Ensemble model (CODEm), spatiotemporal Gaussian process regression (ST-GPR), and DisMod-MR. Previous publications (3, 4, 10) provide more details on these general GBD methods. Briefly, CODEm is a highly systematised tool to analyse cause of death data using an ensemble of different modelling methods for rates or cause fractions with varying choices of covariates that perform best with out-of-sample predictive validity testing. DisMod-MR is a Bayesian meta-regression tool that allows evaluation of all available data on incidence, prevalence, remission, and mortality for a disease, enforcing consistency between epidemiological parameters. ST-GPR is a set of regression methods that borrow strength between locations and over time for single metrics of interest, such as risk factor exposure or mortality rates. In addition, for select diseases, particularly for rarer outcomes, alternative modelling strategies have been developed.

In GBD 2019, we designated a set of standard locations that included all countries and territories as well as the subnational locations for Brazil, China, India, and the USA. Coefficients of covariates in the three main modelling tools were estimated for these standard locations only—ie, we ignored data from subnational locations other than for Brazil, China, India, and the USA. Using this set of standard locations will prevent changes in regression coefficients from one GBD cycle to the next that are solely due to the addition of new subnational units in the analysis that might have lower quality data or small populations. Changes to CODEm for GBD 2019 included the addition of count models to the model ensemble for rarer causes. We also modified DisMod-MR priors to effectively increase the out-of-sample coverage of uncertainty intervals (UIs) as assessed in simulation testing.

DisMod-MR was used to estimate deaths from three outcomes (dementia, Parkinson's, and atrial fibrillation), and to determine the proportions of deaths by underlying aetiologies of cirrhosis, liver cancer, and chronic kidney disease deaths.

Risk Factors Methods

Overview

The GBD 2019 estimation of attributable burden followed the general framework established for comparative risk assessment (CRA) (11, 12) used in GBD since 2002. Here, we provide a general overview and details on major innovations since GBD 2017. CRA can be divided into six key steps: inclusion of risk–outcome pairs in the analysis; estimation of relative risk as a function of exposure; estimation of exposure levels and distributions; determination of the counterfactual level of exposure, the level of exposure with minimum risk called the theoretical minimum risk exposure level (TMREL); computation of population attributable fractions and attributable burden; and estimation of mediation of different risk factors through other risk factors such as high body-mass index (BMI) and ischaemic heart disease, mediated through elevated systolic blood pressure (SBP), elevated fasting plasma glucose (FPG), and elevated LDL cholesterol, to compute the burden attributable to various combinations of risk factors (13).

Geographical units, age groups, and time periods

GBD 2019 estimated prevalence of exposure and attributable deaths, YLLs, YLDs, and DALYs for 23 age groups; males, females, and both sexes combined; and 204 countries and territories that were grouped into 21 regions and seven super-regions. GBD 2019 includes subnational analyses for Italy, Nigeria, Pakistan, the Philippines, and Poland, and 16 countries previously estimated at subnational levels (Brazil, China, Ethiopia, India, Indonesia, Iran, Japan, Kenya, Mexico, New Zealand, Norway, Russia, South Africa, Sweden, the UK, and the USA). All subnational analyses are at the first level of administrative organisation within each country except for New Zealand (by Māori ethnicity), Sweden (by Stockholm and non-Stockholm), the UK (by local government authorities), and the Philippines (by province). For this cycle, nine countries and territories (Cook Islands, Monaco, San Marino, Nauru, Niue, Palau, Saint Kitts and Nevis, Tokelau, and Tuvalu) were added, such that the GBD location hierarchy now includes all WHO member states. These new locations were previously included in regional totals by assuming that age-specific rates were equal to the regional rates. At the most detailed level, we generated estimates for 990 locations. The GBD diseases and injuries analytical framework generated estimates for every year from 1990 to 2019.

GBD risk factor hierarchy

Individual risk factors such as low birthweight or ambient ozone pollution are evaluated in the GBD CRA. In addition, there has been policy interest in groups of risk factors such as household air pollution combined with ambient particulate matter. To accommodate these diverse interests, the GBD CRA has a risk factor hierarchy. Level 1 risk factors are behavioural, environmental and occupational, and metabolic; Level 2 risk factors include 20 risks or clusters of risks; Level 3 includes 52 risk factors or clusters of risks; and Level 4 includes 69 specific risk factors. Counting all specific risk factors and aggregates computed in GBD 2019 yields 87 risks or clusters of risks.

Determining the inclusion of risk–outcome pairs in GBD

Since GBD 2010, we have used the World Cancer Research Fund criteria for convincing or probable evidence of risk–outcome pairs (14). For GBD 2019, we completely updated our systematic reviews for

81 risk–outcome pairs. Convincing evidence requires more than one study type, at least two cohorts, no substantial unexplained heterogeneity across studies, good-quality studies to exclude the risk of confounding and selection bias, and biologically plausible dose–response gradients. For GBD, for a newly proposed or evaluated risk–outcome pair, we additionally required that there was a significant association (p<0·05) after taking into account sources of potential bias. To avoid risk–outcome pairs repetitively entering and leaving the analysis with each cycle of GBD, the criteria for exclusion requires that with the available studies the association has a p value greater than 0·1. On the basis of these reviews and meta-regressions, 12 risk–outcome pairs included in GBD 2017 were excluded from GBD 2019: vitamin A deficiency and lower respiratory infections; zinc deficiency and lower respiratory infections; diet low in fruits and four outcomes: lip and oral cavity cancer, nasopharynx cancer, other pharynx cancer, and larynx cancer; diet low in whole grains and two outcomes: intracerebral haemorrhage and subarachnoid haemorrhage; intimate partner violence and maternal abortion and miscarriage; and high FPG and three outcomes: chronic kidney disease due to hypertension, chronic kidney disease due to glomerulonephritis, and chronic kidney disease due to other and unspecified causes. In addition, on the basis of multiple requests to begin capturing important dimensions of climate change into GBD, we evaluated the direct relationship between high and low non-optimal temperatures on all GBD disease and injury outcomes. Rather than rely on a heterogeneous literature with a small number of studies examining relationships with specific diseases and injuries, we analysed individual-level cause of death data for all locations with available information on daily temperature, location, and International Classification of Diseases-coded cause of death. These data totalled 58.9 million deaths covering eight countries. On the basis of this analysis, 27 GBD cause Level 3 outcomes met the inclusion criteria for each non-optimal risk factor and were included in this analysis. Other climate-related relationships, such as between precipitation or humidity and health outcomes, have not yet been evaluated.

Estimating relative risk as a function of exposure for each risk–outcome pair

In GBD, we use published systematic reviews and for GBD 2019, we updated these where necessary to include any new studies that became available before Dec 31, 2019. We did meta-analyses of relative risks from these studies as a function of exposure. For GBD 2019, 81 new systematic reviews were done, including for 44 diet risk–outcome pairs. To allow for risk functions that might not be log-linear, we relaxed the meta-regression assumptions to allow for monotonically increasing or decreasing but potentially non-linear functions for 147 risk–outcome pairs. 218 risk–outcome pairs were estimated assuming log-linear relationships. For 126 risk–outcome pairs, exposure was dichotomous or polytomous. For 37 risk–outcome pairs, the population attributable fractions were assumed by definition to be 100% (eg, 100% of diabetes is assumed to be, by definition, related to elevated FPG). For 32 risk–outcome pairs, other approaches were used that reflected the nature of the evidence that has been collected for those risks. For risks that affect cardiovascular outcomes, we adjusted relative risks by age such that they follow the empirical pattern of attenuation seen in published studies for elevated SBP, FPG, and LDL cholesterol.

Estimation of the distribution of exposure for each risk by age-sex-location-year

For each risk factor, we systematically searched for published studies, household surveys, censuses, administrative data, ground monitor data, or remote sensing data that could inform estimates of risk exposure. To estimate mean levels of exposure by age-sex-location-year, specific methods varied across risk factors. For many risk factors, exposure data were modelled using either spatiotemporal Gaussian

process regression or DisMod-MR 2.1 (4, 15), which are Bayesian statistical models developed over the past 12 years for GBD analyses. For most risk factors, the distribution of exposure across individuals was estimated by modelling a measure of dispersion, usually the SD, and fitting an ensemble of parametric distributions to the predicted mean and SD. Ensemble distributions for each risk were estimated based on individual-level data. Because of the strong dependency between birthweight and gestational age, exposure for these risks was modelled as a joint distribution using the copula method (16). In many cases, exposure data were available for the reference method of ascertainment and for alternative methods, such as tobacco surveys reporting daily smoking versus total smoking; in these cases, we estimated the statistical relationship between the reference and alternative methods of ascertainment using network meta-regression and corrected the alternative data using this relationship.

Determining the TMREL

For harmful risk factors with monotonically increasing risk functions, the theoretical minimum risk level was set to 0. For risk factors with J-shaped or U-shaped risk functions, such as for sodium and ischaemic heart disease or BMI and ischaemic heart disease, the TMREL was determined as the low point of the risk function. When the bottom of the risk function was flat or poorly determined, the TMREL uncertainty interval (UI) captured the range over which risks are indistinguishable. For protective risks with monotonically declining risk functions with exposure, namely risk factors where exposure lowers the risk of an outcome, the challenge is selecting the level of exposure with the lowest level of risk strongly supported by the available data. Projecting beyond the level of exposure supported by the available studies could exaggerate the attributable burden for a risk factor. In these cases, for each risk–outcome pair, we determined the exposure level at the 85th percentile of exposure in the cohorts or trials used in the risk meta-regression. We then generated the TMREL by weighting each risk–outcome pair by the relative global magnitude of each outcome.

Estimation of the population attributable fraction and attributable burden

For each risk factor j, we computed the population attributable fraction (PAF) by age-sex-location-year using the following general formula for a continuous risk:

$$\mathrm{PAF}_{joasgt} = \frac{\int_{x=l}^{u} \mathrm{RR}_{joasg}(x) P_{jasgt}(x) dx - \mathrm{RR}_{joasg}(\mathrm{TMREL}_{jas})}{\int_{x=l}^{u} \mathrm{RR}_{joasg}(x) P_{jasgt}(x) dx}$$

where PAF_{joasgt} is the PAF for cause o, for age group a, sex s, location g, and year t; $\mathrm{RR}_{joasg}(x)$ is the relative risk as a function of exposure level x for risk factor j, for cause o controlled for confounding, age group a, sex s, and location g with the lowest level of observed exposure as l and the highest as u; $P_{jasgt}(x)$ is the distribution of exposure at x for age group a, sex s, location g, and year t; and TMREL_{jas} is the TMREL for risk factor j, age group a, and sex s. Where risk exposure is dichotomous or polytomous, this formula simplifies to the discrete form of the equation.

Estimation of the PAF took into account the risk function and the distribution of exposure across individuals in each age-sex-location-year. By drawing 1000 samples from the risk function, 1000 distributions of exposure for each age-sex-location-year, and 1000 samples from the TMREL, we propagated all of these sources of uncertainty into the PAF distributions. PAFs were also applied at the draw level to the uncertainty distributions of each associated outcome for that age-sex-location-year.

Estimating the PAF and attributable burden for combinations of risk factors

For the estimation of each specific risk factor, the counterfactual distribution of exposure is the TMREL for that specific risk with no change in other risk factors. Thus, the sum of these risk-specific estimates of attributable burden can exceed 100% for some causes, such as cardiovascular diseases. It is also useful to assess the PAF and attributable burden for combinations of risk factors, such as all diet components together or household air and ambient particulate matter pollution. To estimate the combined effects of risk factors, we should take into account how one risk factor might be mediated through another (eg, the effect of fruit intake might be partly mediated through fibre intake). We used the mediation matrix as developed in GBD 2017 (6) to try to correct for overestimation of the PAF and the attributable burden for combinations of risks if we were to simply assume independence without any mediation.

Risk-deleted death rates

We computed risk-deleted death rates as the death rates that would be observed if all risk factors were set to their respective TMRELs. This was calculated as the death rate in each age-sex group multiplied by 1 minus the all-risk PAF for that age-sex group in each location.

CoD cause-specific modelling descriptions

Cardiovascular Diseases

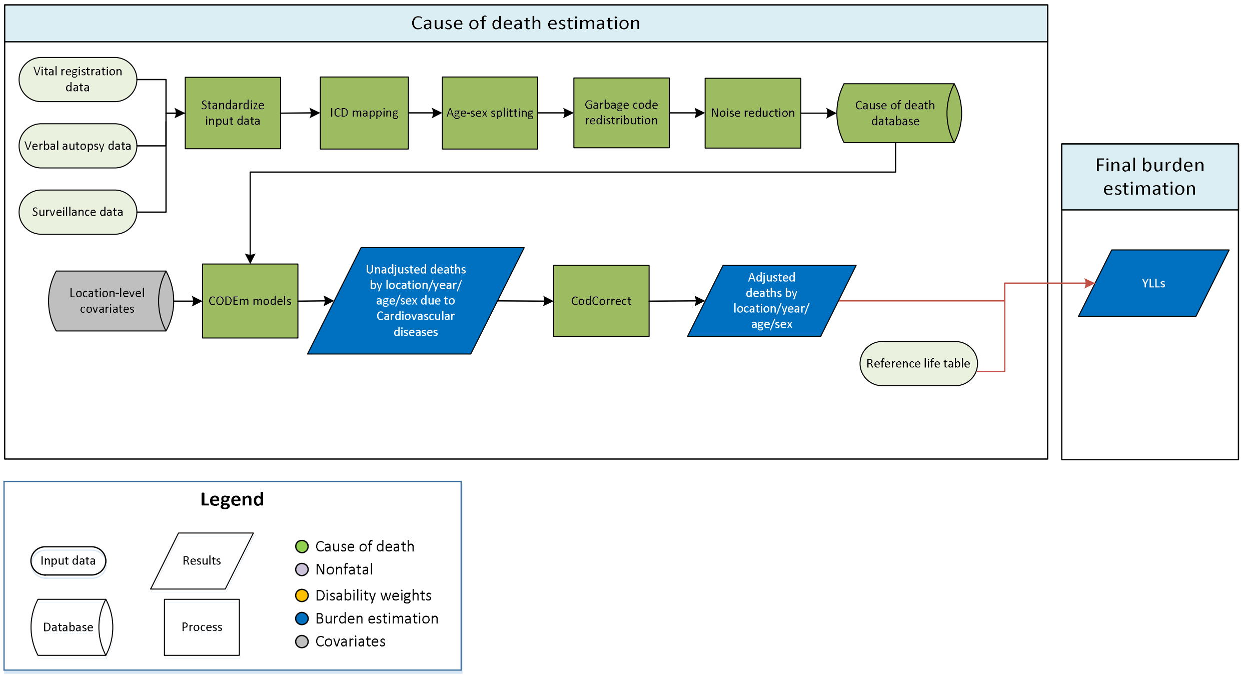

Input data

Vital registration and verbal autopsy data were used to model the parent cardiovascular envelope. We outliered non-representative subnational verbal autopsies from a number of Indian states and verbal autopsy data in Nepal and Papua New Guinea that were implausible in terms of time and age trends. We

also outliered verbal autopsy data sources that were implausibly low in all age groups and ICD8 and ICD9BTL data points that were inconsistent with the rest of the data and created implausible time trends.

Modelling strategy

We used a standard CODEm approach to model deaths from cardiovascular diseases. The covariates included in the ensemble modelling process are listed in the table below. For GBD 2019, adjusted dietary covariates for consumption of fruits, omega-3 fatty acids, vegetables, nuts and seeds, and polyunsaturated fatty acids were replaced with the summary exposure value scalars for diet low in each of these factors. The direction for each dietary covariate was changed from -1 to 1 to as our *a priori* assumption is that low levels of intake of these dietary factors are associated with increasing mortality risk from cardiovascular disease. In addition, the dietary covariate for whole grains (kcal/capita, adjusted) the covariate for socio-demographic index as exploratory analyses indicated that these covariates were not predictive of the outcome. The summary exposure value scalar for CVD was dropped as this covariate was not produced for Level 2 causes in GBD 2019. Apart from these changes to the covariates, there are no other substantive changes from the approach used in GBD 2017.

Table: Selected covariates for CODEm models, cardiovascular diseases

Covariate	Transformation	Level	Direction
Cholesterol (total, mean per capita)	None	1	1
Smoking prevalence	None	1	1
Systolic blood pressure (mmHg)	None	1	1
Mean BMI	None	2	1
Elevation over 1500m (proportion)	None	2	-1
Fasting plasma glucose (mmol/L)	None	2	1
Outdoor pollution ($PM_{2.5}$)	None	2	1
Indoor air pollution (all fuel types)	None	2	1
Healthcare access and quality index	None	2	-1
Lag distributed income per capita (I$)	Log	3	-1
Summary exposure value, omega-3 fatty acids	None	3	1
Summary exposure value, fruits	None	3	1
Summary exposure value, vegetables	None	3	1
Summary exposure value, Nuts and seeds	None	3	1
Pulses/legumes (kcal/capita, unadjusted)	None	3	-1
Summary exposure value, PUFA adjusted (percent)	None	3	1
Alcohol (litres per capita)	None	3	1
Trans fatty acid	None	3	1

Ischaemic Heart Disease

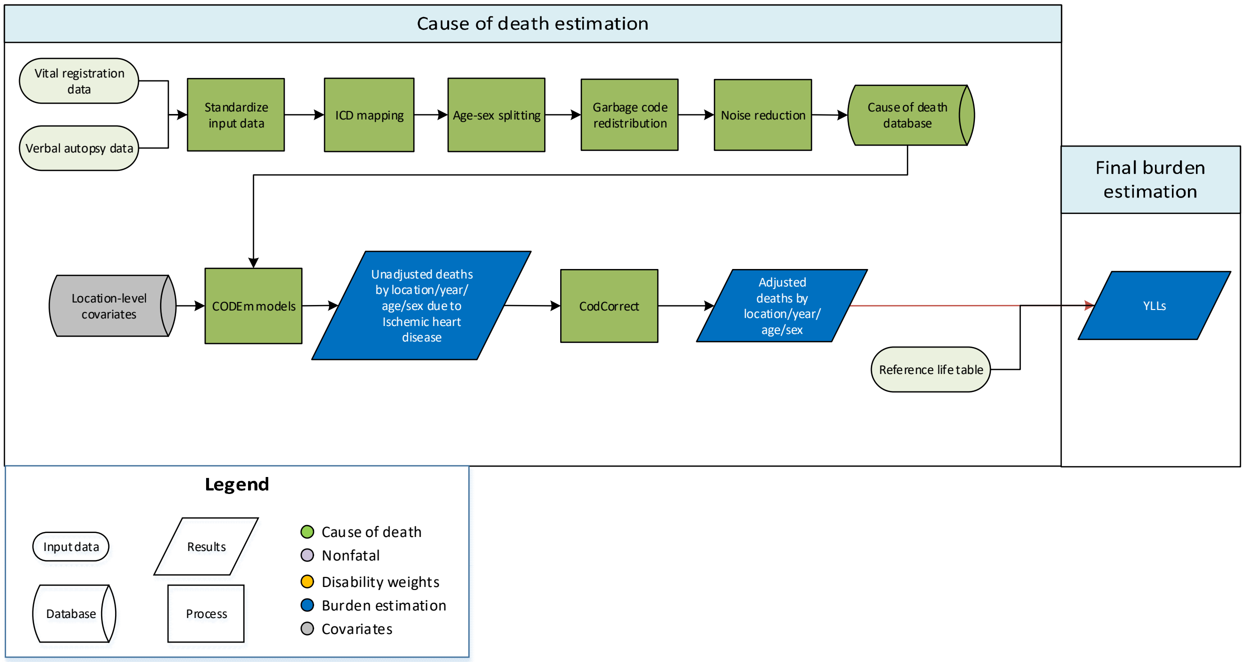

Input data

Vital registration and verbal autopsy data were used to model ischaemic heart disease. We outliered verbal autopsy data in countries and subnational locations where high-quality vital registration data were also available. We also outliered non-representative subnational verbal autopsy data points, ICD8 and ICD9BTL data points which were inconsistent with the rest of the data and created implausible time trends, and data in a number of Indian states identified by experts as poor-quality.

Modelling strategy

We used a standard CODEm approach to model deaths from ischemic heart disease. For GBD 2019, adjusted dietary covariates for consumption of fruits, omega-3 fatty acids, vegetables, nuts and seeds, and polyunsaturated fatty acids were replaced with the summary exposure value scalars for diet low in each of these factors. The direction for each dietary covariate was changed from -1 to 1 to as our *a priori* assumption is that low levels of intake of these dietary factors are associated with increasing mortality risk from ischaemic heart disease. We changed the direction of the alcohol variable from 0 to 1 to reflect our *a priori* hypothesis about the expected direction of the association between this risk factor and mortality risk of ischaemic heart disease. In addition, we changed the level of the covariate for trans fatty acid from 1 to 3. Besides these covariate changes, there are no other substantive changes from the approach used in GBD 2017.

Table: Selected covariates for CODEm models, ischaemic heart disease

Covariate	Transformation	Level	Direction
Summary exposure value, IHD	None	1	1
Cholesterol (total, mean per capita)	None	1	1
Smoking prevalence	None	1	1
Systolic blood pressure (mmHg)	None	1	1
Mean BMI	None	2	1
Elevation over 1500m (proportion)	None	2	-1
Fasting plasma glucose	None	2	1
Outdoor pollution ($PM_{2.5}$)	None	2	1
Indoor air pollution	None	2	1
Healthcare access and quality index	None	2	-1
Lag distributed income per capita (I$)	Log	3	-1
Summary exposure value, omega-3	None	3	1
Summary exposure value, fruits	None	3	1
Summary exposure value, vegetables	None	3	1
Summary exposure value, nuts and seeds	None	3	1
Pulses/legumes (kcal/capita, unadjusted)	None	3	-1
Summary exposure value, PUFA (percent, adjusted)	None	3	1
Alcohol (litres per capita)	None	3	1
Trans fatty acid	None	3	1

Stroke

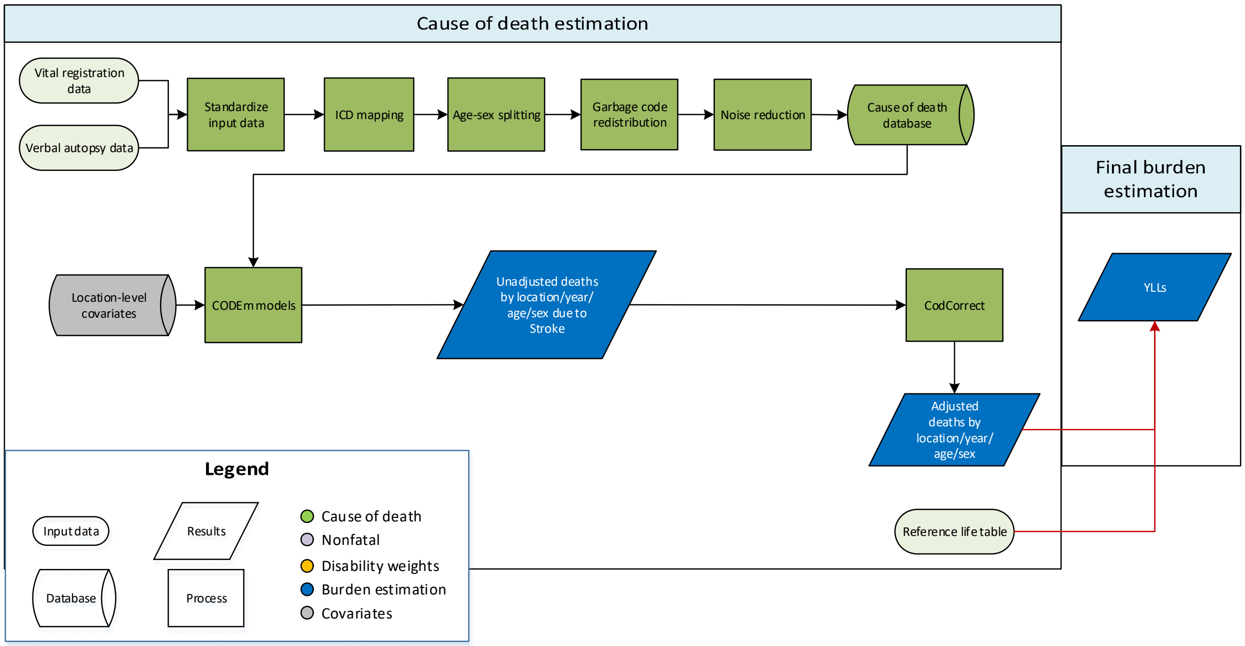

Input data

Verbal autopsy and vital registration data were used to model cerebrovascular disease (stroke). We reassigned deaths from verbal autopsy reports for cerebrovascular disease to the parent cardiovascular disease for both sexes for those under 20 years of age. We outliered non-representative subnational verbal autopsy datapoints. We also outliered ICD8, ICD9BTL, and tabulated ICD10 datapoints which were inconsistent with the rest of the data and created implausible time trends. Datapoints from sources which were implausibly low in all age groups and data points that were causing the regional estimates to be improbably high were outliered.

Modelling strategy

We used a standard CODEm approach to model deaths from stroke. The covariates included in the ensemble modelling process are listed in the table below. For GBD 2019, adjusted dietary covariates for consumption of fruits, omega-3 fatty acids, vegetables, nuts and seeds, and polyunsaturated fatty acids (PUFA) were replaced with the summary exposure value scalars for diet low in each of these factors. The direction for each dietary covariate was changed from -1 to 1 to as our a priori assumption is that low levels of intake of these dietary factors are associated with increasing mortality risk from stroke. We dropped the dietary covariate for whole grains (kcal/capita, adjusted) and the socio-demographic index covariate as exploratory analyses indicated that these variables were not predictive of stroke mortality. In addition, we changed the direction of the alcohol consumption covariate from 0 to 1 to reflect the expected direction of the association for this risk factor with stroke mortality. Apart from these covariate changes, there are no substantive changes from the approach used in GBD 2017.

Table: Selected covariates for CODEm models, stroke

Covariate	Transformation	Level	Direction
Summary exposure variable, stroke	None	1	1
Cholesterol (total, mean per capita)	None	1	1
Smoking prevalence	None	1	1
Systolic blood pressure (mmHg)	None	1	1
Mean BMI	None	2	1
Elevation over 1,500m (proportion)	None	2	-1
Fasting plasma glucose	None	2	1
Outdoor pollution ($PM_{2.5}$)	None	2	1
Indoor air pollution	None	2	1
Healthcare Access and Quality Index	None	2	-1
Lag distributed income per capita (I$)	Log	3	-1
Summary exposure value, omega-3	None	3	1
Summary exposure value, fruits	None	3	1
Summary exposure value, vegetables	None	3	1
Summary exposure value, nuts and seeds	None	3	1
Pulses/legumes (kcal/capita, unadjusted)	None	3	-1
Summary exposure value, PUFA adjusted (percent)	None	3	1
Alcohol (litres per capita)	None	3	1
Trans fatty acid	None	3	1

Ischaemic Stroke

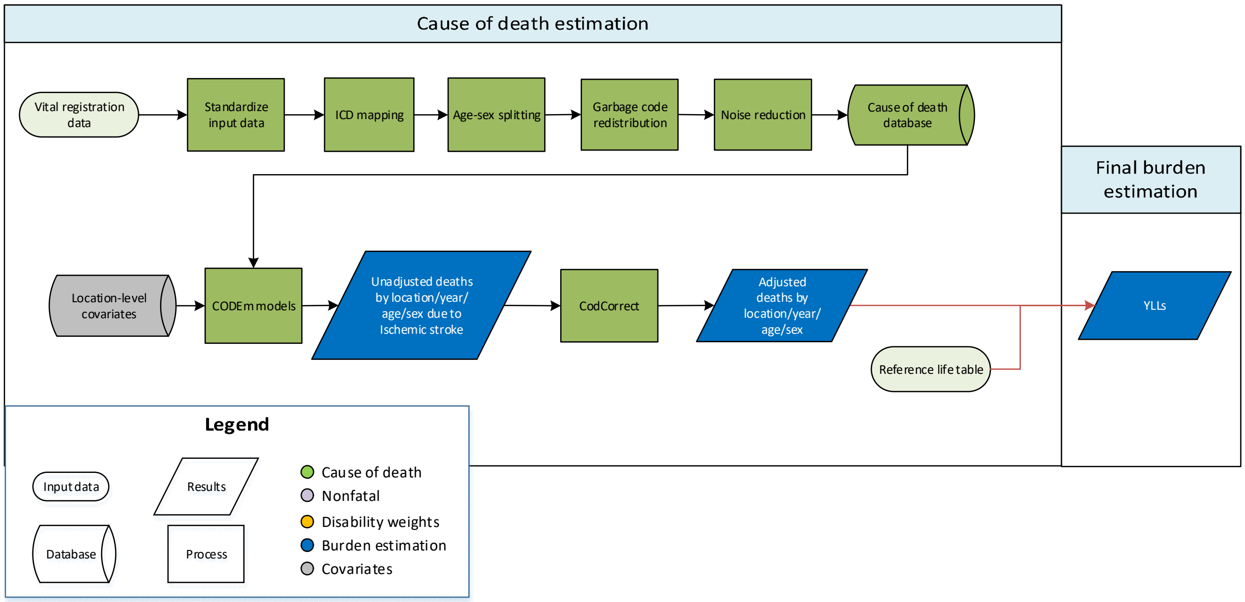

Input data

Vital registration data were used to model deaths from ischaemic stroke. We outliered ICD8 data points which were inconsistent with the rest of the data and created implausible time trends. We also outliered ICD10 data points in The Republic of Tajikistan due to unstable and implausible estimates in similar age groups.

Modelling strategy

We used a standard CODEm approach to model deaths from ischemic stroke. For GBD 2019, adjusted dietary covariates for consumption of fruits, omega-3 fatty acids, vegetables, nuts and seeds, and polyunsaturated fatty acids were replaced with the summary exposure value scalars for diet low in each of these factors. The direction for each dietary covariate was changed from -1 to 1 to as our *a priori* assumption is that low levels of intake of these dietary factors are associated with increasing mortality risk from ischaemic stroke. In addition, the dietary covariate for whole grains (kcal/capita, adjusted) and the socio-demographic index covariate were dropped as exploratory analyses indicated that the covariates were not predictive of the outcome. In addition, we changed the direction of the alcohol variable from 0 to 1 to reflect our *a priori* hypothesis about the expected direction of the association between this risk factor and mortality risk of ischaemic stroke. We also changed the level of the trans fatty acid covariate from 1 to 3. Besides these covariate changes, there are no other substantive changes from the approach used in GBD 2017.

Table: Selected covariates for CODEm models, ischaemic stroke

Covariate	Transformation	Level	Direction
Summary exposure value, ischaemic stroke	None	1	1
Cholesterol (total, mean per capita)	None	1	1
Smoking prevalence	None	1	1
Systolic blood pressure (mmHg)	None	1	1
Mean BMI	None	2	1
Elevation over 1500m (proportion)	None	2	-1
Fasting plasma glucose	None	2	1
Outdoor pollution ($PM_{2.5}$)	None	2	1
Indoor air pollution	None	2	1
Healthcare access and quality index	None	2	-1
Lag distributed income per capita (I$)	Log	3	-1
Summary exposure value, omega-3	None	3	1
Summary exposure value, fruits	None	3	1
Summary exposure value, vegetables	None	3	1
Summary exposure value, nuts and seeds	None	3	1
Pulses/legumes (kcal/capita, unadjusted)	None	3	-1
Summary exposure value PUFA adjusted	None	3	1
Alcohol (litres per capita)	None	3	1
Trans fatty acid	None	3	1

Intracerebral haemorrhage

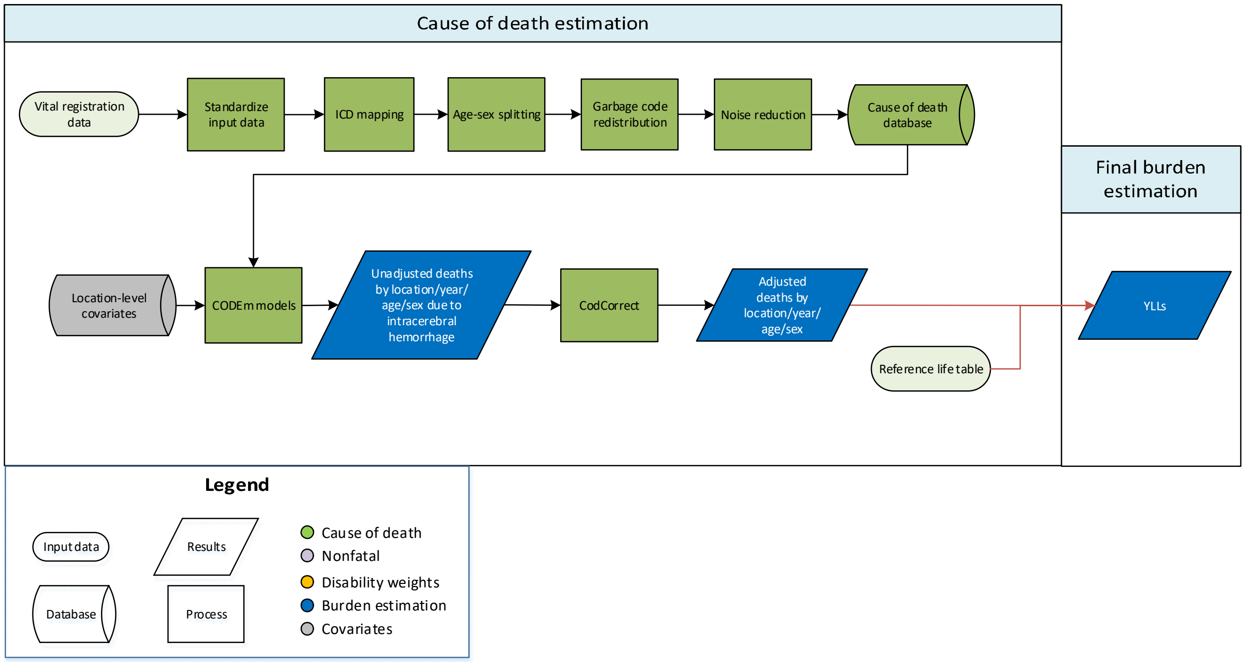

Input data

Vital registration data were used to model intracerebral haemorrhage. We outliered ICD8 data points which were inconsistent with the rest of the data and created implausible time trends. In addition, we outliered vital registration data points in certain countries in Latin American countries due to implausibly high values at the oldest age groups resulting in inconsistencies in time trends.

Modelling strategy

We used a standard CODEm approach to model deaths from intracerebral haemorrhage. For GBD 2019, adjusted dietary covariates for consumption of fruits, omega-3 fatty acids, vegetables, nuts and seeds, and polyunsaturated fatty acids were replaced with the summary exposure value scalars for diet low in each of these factors. The direction for each dietary covariate was changed from -1 to 1 to as our *a priori* assumption is that low levels of intake of these dietary factors are associated with increasing mortality risk from intracerebral haemorrhage. In addition, the dietary covariate for whole grains (kcal/capita, adjusted) and the social demographic index covariate were dropped as exploratory analyses indicated that these covariates were not predictive of the mortality risk from intracerebral haemorrhage. We changed the direction of the covariate for alcohol from 0 to 1 due to our *a priori* hypothesis about the direction of the association for this covariate. We also changed the level of the cholesterol covariate from 1 to 3 and the direction from 0 to -1 to reflect the mixed and inconclusive evidence regarding cholesterol levels and risk of intracerebral haemorrhage. In addition, we changed the level of the trans fatty acid from covariate from 1 to 3 in accordance with the expected importance of this risk factor on mortality from intracerebral haemorrhage. Besides these covariate changes, there are no other substantive changes from the approach used in GBD 2017.

Table: Selected covariates for CODEm models, intracerebral haemorrhage

Covariate	Transformation	Level	Direction
Summary exposure variable, ICH	None	1	1
Smoking prevalence	None	1	1
Systolic blood pressure (mmHg)	None	1	1
Mean BMI	None	2	1
Elevation over 1500m (proportion)	None	2	-1
Fasting plasma glucose	None	2	1
Outdoor pollution ($PM_{2.5}$)	None	2	1
Indoor air pollution	None	2	1
Healthcare access and quality index	None	2	-1
Lag distributed income per capita (I$)	Log	3	-1
Summary exposure value omega-3	None	3	1
Summary exposure value fruits	None	3	1
Summary exposure value vegetables	None	3	1
Summary exposure value nuts and seeds	None	3	1
Pulses/legumes (kcal/capita, un-adjusted)	None	3	-1
Summary exposure value PUFA	None	3	1
Cholesterol (total, mean per capita)	None	3	-1
Alcohol (litres per capita)	None	3	1
Trans fatty acid	None	3	1

Subarachnoid haemorrhage

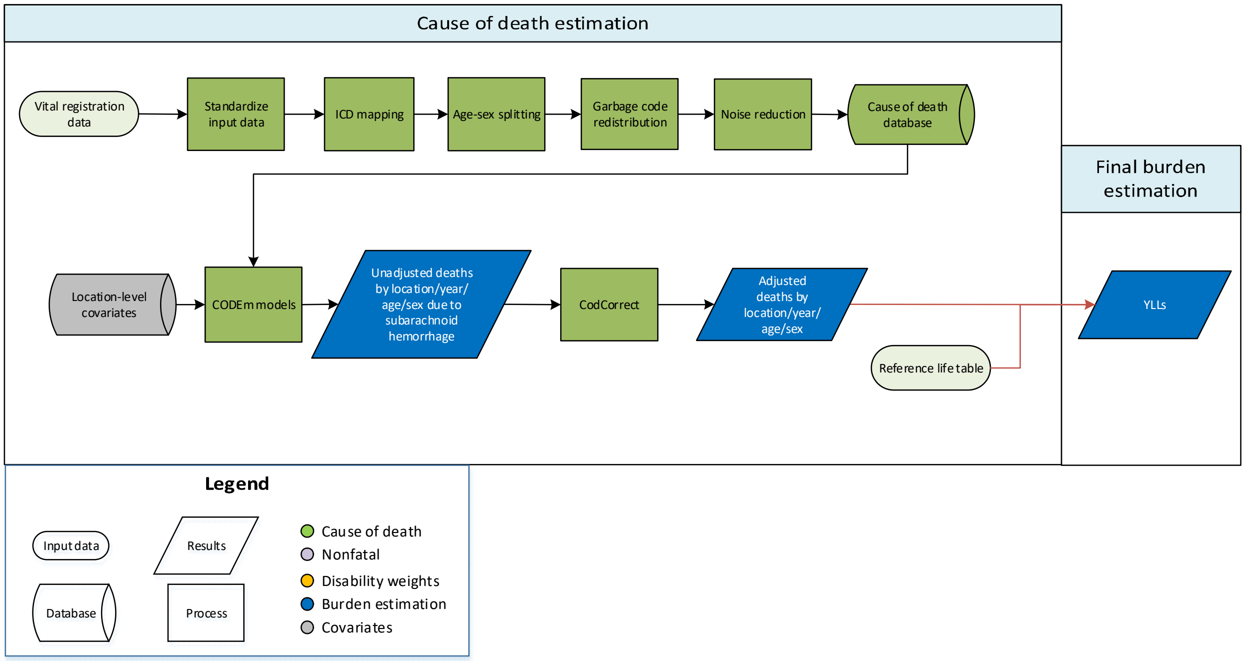

Input data

Vital registration data were used to model subarachnoid haemorrhage. We outliered ICD8 datapoints which were inconsistent with the rest of the data and created implausible time trends. In addition, we outliered vital registration data in Tibet that was implausibly high for all years and age groups.

Modelling strategy

We used a standard CODEm approach to model deaths from subarachnoid haemorrhage. The covariates chosen for inclusion in the ensemble modelling process are listed in the table below. For GBD 2019, we dropped the Socio-demographic Index covariate as exploratory analyses indicated that it was not predictive of the outcome. We also changed the direction of the alcohol covariate from 0 to 1 to reflect the expected direction of the association of this risk factor with mortality risk. Apart from these changes to the covariates, there are no substantive changes from the approach used in GBD 2017.

Table: Selected covariates for CODEm models, subarachnoid haemorrhage

Level	Covariate	Transformation	Direction
1	Smoking prevalence	None	1
1	Systolic blood pressure (mmHg)	None	1
2	Healthcare access and quality index	None	-1
3	Lag distributed income per capita (I$)	Log	-1
3	Alcohol (litres per capita)	None	1

Hypertensive Heart Disease

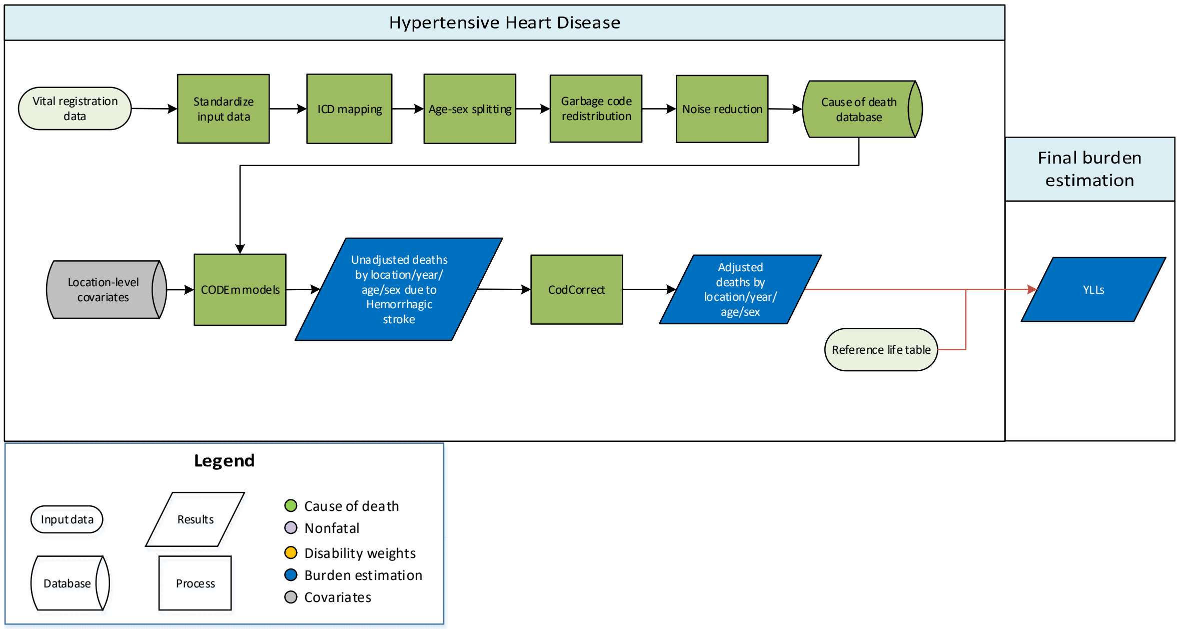

Input data

Vital registration data were used to model cause-specific mortalty for hypertensive heart disease. We outliered ICD9BTL data points, which were inconsistent with the rest of the data and created implausible

time trends. In addition, we outliered vital registration data from Grenada in 2017 for being implausibly low across all age groups.

Modelling strategy

We used a standard CODEm approach to model deaths from hypertensive heart disease. For GBD 2019, adjusted dietary covariates for consumption of fruits, omega-3 fatty acids, vegetables, nuts and seeds, and polyunsaturated fatty acids were replaced with the summary exposure value scalars for diet low in each of these factors. The direction for each dietary covariate was changed from -1 to 1 to as our *a priori* assumption is that low levels of intake of these dietary factors are associated with increasing mortality risk from hypertensive heart disease. We also changed the direction of the covariates for alcohol and socio-demographic index from 0 to 1 to reflect the expected direction of these covariates with mortality risk. Apart from these covariate updates, there are no other substantive changes from the approach used in GBD 2017.

Table: Selected covariates for CODEm models, hypertensive heart disease

Covariate	Transformation	Level	Direction
Systolic blood pressure (mmHg)	None	1	1
Cholesterol (total, mean per capita)	None	2	1
Smoking prevalence	None	2	1
Mean BMI	None	2	1
Healthcare access and quality index	None	2	-1
Lag distributed income per capita (I$)	Log	3	-1
Socio-demographic Index	None	3	1
Alcohol (litres per capita)	None	3	1
Summary exposure value, omega-3	None	3	1
Summary exposure value, fruits	None	3	1
Summary exposure value, nuts and seeds	None	3	1
Summary exposure value, PUFA	None	3	1
Summary exposure value, vegetables	None	3	1
Pulses/legumes (kcal/capita, unadjusted)	None	3	-1
Trans fatty acid (percent)	None	3	1

Congenital birth defects: neural tube defects, congenital heart anomalies, orofacial clefts, Down syndrome, Turner syndrome, Klinefelter syndrome, other chromosomal disorders, congenital musculoskeletal anomalies, urogenital congenital anomalies, digestive congenital anomalies, and other congenital birth defects

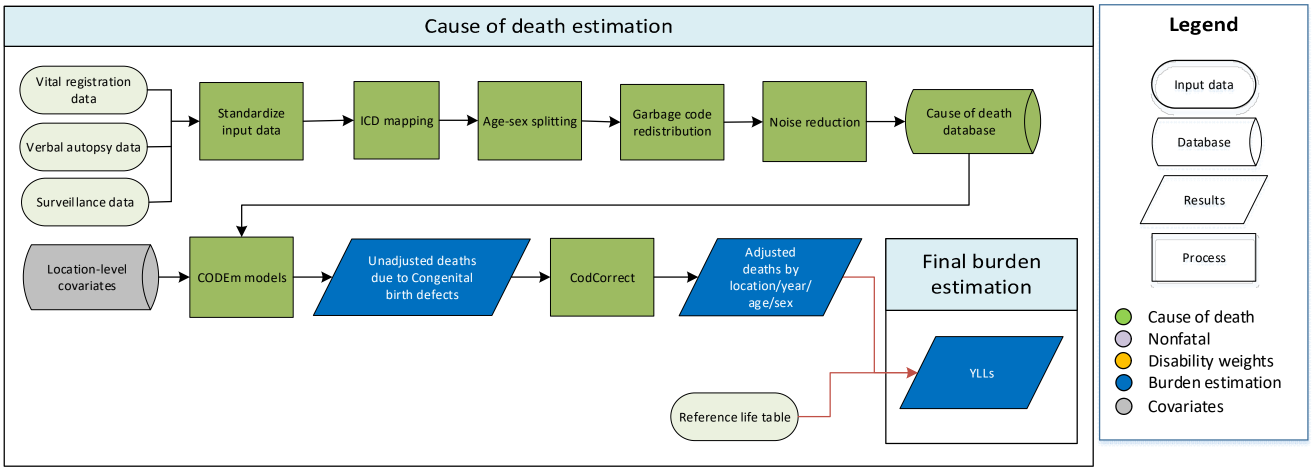

Input data

For GBD 2019, input data for estimating mortality due to congenital anomalies was centrally extracted, processed, and stored in cause of death (CoD) database. Vital registration (VR) was the dominant data type, followed by verbal autopsy (VA) and surveillance. Those CoD data sources that specified the

subcause of birth defect were included in estimation of both the parent congenital anomalies model as well as in subtype-specific models.

For GBD 2019, data exclusions were limited. The majority of VA data were outliered in those over 5 years old as the age patterns were unreliable and led to poor model performance in the under-5 age groups. We also excluded some data sources from the parent model where only a subset of subcauses were specified (e.g., congenital heart disease, neural tube defects, and other congenital anomalies) and the sum of the subcauses clearly represented systematic underreporting of one of the subcauses. Systematic underreporting was suspected when sex- and age-specific rates were more than an order of magnitude lower than neighbouring or comparable locations. Data sources for those locations were still included by default for subcause specific models because underreporting of the total was not assumed to necessarily be associated with underreporting of all of the component conditions.

Modelling strategy

All types of congenital anomalies were estimated using cause of death ensemble modelling (CODEm) for GBD 2019, as was done for previous iterations of the GBD study. Specific causes included neural tube defects, congenital heart anomalies, orofacial clefts, Down syndrome, other chromosomal anomalies, congenital musculoskeletal anomalies, urogenital congenital anomalies, digestive congenital anomalies, and other congenital birth defects. We assumed no mortality from either Klinefelter syndrome or Turner syndrome, for which we model nonfatal outcomes only. For GBD 2019, we modelled congenital anomalies as a cause of death for ages 0–69 years only, assuming that all mortality from congenital conditions occurs before age 70 years of age.

For GBD 2016, we added three new causes to the congenital anomalies: congenital musculoskeletal and limb anomalies; urogenital congenital anomalies; and digestive congenital anomalies. We made no additions to the causes of congenital anomalies for GBD 2017 or 2019.

Table 1: Covariates tested for CODEm model of overall congenital birth defects

Covariate	Transformation	Level	Direction
Maternal alcohol consumption during pregnancy (proportion)	None	1	+
In-facility delivery (proportion)	None	1	-
Live births 35+ (proportion)	None	1	+
Folic acid unadjusted (ug)	None	1	-
Folic acid fortification index	None	1	-
Birth prevalence of congenital heart disease	None	1	+
Birth prevalence of chromosomal anomalies	None	1	+
Legality of abortion	None	2	-
Antenatal care (1 visit) coverage (proportion)	None	2	-
Age-standardised summary exposure value (SEV) of smoking	None	2	+

Antenatal care (4 visits) coverage (proportion)	None	2	-
Healthcare Access and Quality Index	None	2	-
Maternal education (years per capita)	None	3	-
Alcohol (litres per capita)	None	3	+
Age-standardised SEV of low fruits	None	3	+
Outdoor air pollution (PM2.5)	None	3	+
Age-standardised SEV of household air pollution	None	3	+
Socio-demographic Index	None	3	-
Age-standardised SEV of low vegetables	None	3	+

Table 2: Covariates tested for CODEm model of neural tube defects

Covariate	Transformation	Level	Direction
In-facility delivery (proportion)	None	1	-
Folic acid unadjusted (ug)	None	1	-
Folic acid fortification index	None	1	-
Healthcare Access and Quality Index	None	2	-
Antenatal care (1 visit) coverage (proportion)	None	2	-
Antenatal care (4 visits) coverage (proportion)	None	2	-
Age-standardised SEV of smoking	None	2	+
Age-standardised SEV of low fruits	None	3	+
Age-standardised SEV of low vegetables	None	3	+
Maternal education (years per capita)	None	3	-
Socio-demographic Index	None	3	-
Legality of abortion	None	2	-
Maternal alcohol consumption during pregnancy (proportion)	None	3	+
Age-standardised SEV of household air pollution	None	3	+
Age-standardised SEV of fasting plasma glucose	None	3	+
Litres of alcohol consumed per capita	None	3	+

Table 3: Covariates selected for CODEm model of congenital heart anomalies

Covariate	Transformation	Level	Direction

Maternal alcohol consumption during pregnancy (proportion)	None	1	+
Birth prevalence of congenital heart disease	None	1	+
Socio-demographic Index	Log	2	-
Age-standardised SEV of smoking	None	2	+
Age-standardised SEV of diabetes	None	2	+
Healthcare Access and Quality Index	None	2	-
Legality of abortion	None	2	-
Antenatal care (1 visit) coverage (proportion)	None	2	-
In-facility delivery (proportion)	None	2	-
Maternal education (years per capita)	None	3	-
Alcohol (litres per capita)	None	3	+
Antenatal care (4 visits) coverage (proportion)	None	3	-
Skilled birth attendance (proportion)	None	3	-
Live births 35+ (proportion)	None	3	+

Table 4: Covariates selected for CODEm model of cleft lip and cleft palate

Covariate	Transformation	Level	Direction
Socio-demographic Index	None	1	-
Folic acid fortification index	None	1	-
Age-standardised SEV of diabetes	None	2	+
Maternal alcohol consumption during pregnancy (proportion)	None	2	+
Healthcare Access and Quality Index	None	2	-
Legality of abortion	None	2	-
Skilled birth attendance (proportion)	None	2	-
Age-standardised SEV of smoking	None	2	+
Age-standardised SEV of household air pollution	None	3	+
Age-standardised SEV of low vegetables	None	3	+
Alcohol (litres per capita)	None	3	+
Antenatal care (4 visits) coverage (proportion)	None	3	-
Maternal education (years per capita)	None	3	-

Age-standardised SEV of low fruits	None	3	+
Antenatal care (1 visit) coverage (proportion)	None	3	-

Table 5: Covariates selected for CODEm model of Down syndrome

Covariate	Transformation	Level	Direction
Live births 35+ (proportion)	None	1	+
Legality of abortion	None	1	-
Live births 40+ (proportion)	None	1	+
Birth prevalence of chromosomal anomalies	None	1	+
Socio-demographic Index	None	2	-
In-facility delivery (proportion)	None	2	-
Healthcare Access and Quality Index	None	2	-
Maternal alcohol consumption during pregnancy (proportion)	None	3	+
Antenatal care (1 visit) coverage (proportion)	None	3	-
Maternal education (years per capita)	None	3	-
Age-standardised SEV of household air pollution	None	3	+
Antenatal care (4 visits) coverage (proportion)	None	3	-
Age-standardised SEV of low vegetables	None	3	-
Age-standardised SEV of smoking	None	3	+
Litres of alcohol consumed per capita	None	3	+

Table 6: Covariates selected for CODEm model of other chromosomal abnormalities

Covariate	Transformation	Level	Direction
Live births 35+ (proportion)	None	1	+
Live births 40+ (proportion)	None	1	+
Legality of abortion	None	1	-
Lag distributed income (LDI) (I$ per capita)	Log	2	-
Healthcare Access and Quality Index	None	2	-
Antenatal care (4 visits) coverage (proportion)	None	2	-
Antenatal care (1 visit) coverage (proportion)	None	2	-
In-facility delivery (proportion)	None	2	-

Maternal alcohol consumption during pregnancy (proportion)	None	2	+
Socio-demographic Index	None	3	-
Alcohol (litres per capita)	None	3	+
Age-standardised SEV of smoking	None	3	+
Age-standardised SEV of household air pollution	None	3	+
Maternal education (years per capita)	None	3	-
Skilled birth attendance (proportion)	None	3	-

Table 7: Covariates selected for CODEm model of congenital musculoskeletal and limb anomalies

Covariate	Transformation	Level	Direction
Maternal alcohol consumption during pregnancy (proportion)	None	1	+
Legality of abortion	None	1	-
In-facility delivery (proportion)	None	2	-
Ag-standardised SEV of diabetes	None	2	+
Socio-demographic Index	None	2	-
Healthcare Access and Quality Index	None	2	-
Age-standardised SEV of household air pollution	None	2	+
Age-standardised SEV of smoking	None	2	+
Antenatal care (4 visits) coverage (proportion)	None	3	-
Alcohol (litres per capita)	None	3	+
Age-standardised SEV of low fruits	None	3	+
Age-standardised SEV of low vegetables	None	3	+
Maternal education (years per capita)	None	3	-
Antenatal care (1 visit) coverage (proportion)	None	3	-
LDI per capita	Log	3	-

Table 8: Covariates selected for CODEm model of urogenital congenital anomalies

Covariate	Transformation	Level	Direction
Age-standardised SEV of smoking	None	1	+
Maternal alcohol consumption during pregnancy (proportion)	None	1	+
Healthcare Access and Quality Index	None	2	-

Diabetes age-standardised prevalence (proportion)	None	2	+
Socio-demographic Index	None	2	-
Age-standardised SEV of outdoor air pollution	None	2	+
In-facility delivery (proportion)	None	2	-
Age-standardised SEV of household air pollution	None	2	+
Antenatal care (1 visit) coverage (proportion)	None	3	-
Alcohol (litres per capita)	None	3	+
Maternal education (years per capita)	None	3	-
LDI (I$ per capita)	Log	3	-
Antenatal care (4 visits) coverage (proportion)	None	3	-

Table 9: Covariates selected for CODEm model of digestive congenital anomalies

Covariate	Transformation	Level	Direction
Maternal alcohol consumption during pregnancy (proportion)	None	1	+
Age-standardised SEV of smoking	None	1	+
Age-standardised SEV of household air pollution	None	2	+
Diabetes age-standardised prevalence (proportion)	None	2	+
Age-standardised SEV of diabetes	None	2	+
Socio-demographic Index	None	2	-
Age-standardised SEV of obesity	None	2	+
In-facility delivery (proportion)	None	2	-
Healthcare Access and Quality Index	None	2	-
Alcohol (litres per capita)	None	3	+
Maternal education (years per capita)	None	3	-
Age-standardised SEV of low vegetables	None	3	+
Antenatal care (1 visit) coverage (proportion)	None	3	-
Antenatal care (4 visits) coverage (proportion)	None	3	-
Age-standardised SEV of low fruits	None	3	+
LDI (I$ per capita)	Log	3	-
MCI	None	3	-

Table 10: Covariates selected for CODEm model of other congenital birth defects

Covariate	Transformation	Level	Direction
Maternal alcohol consumption during pregnancy (proportion)	None	1	+
Live births 35+ (proportion)	None	1	+
Maternal education (years per capita)	None	2	-
Legality of abortion	None	2	-
In-facility delivery (proportion)	None	2	-
Age-standardised SEV of household air pollution	None	2	+
Healthcare Access and Quality Index	None	2	-
Antenatal care (1 visit) coverage (proportion)	None	3	-
Age-standardised SEV of diabetes	None	3	+
LDI (I$ per capita)	Log	3	-
Socio-demographic Index	None	3	-
Antenatal care (4 visits) coverage (proportion)	None	3	-
Alcohol (litres per capita)	None	3	+

Rheumatic heart disease

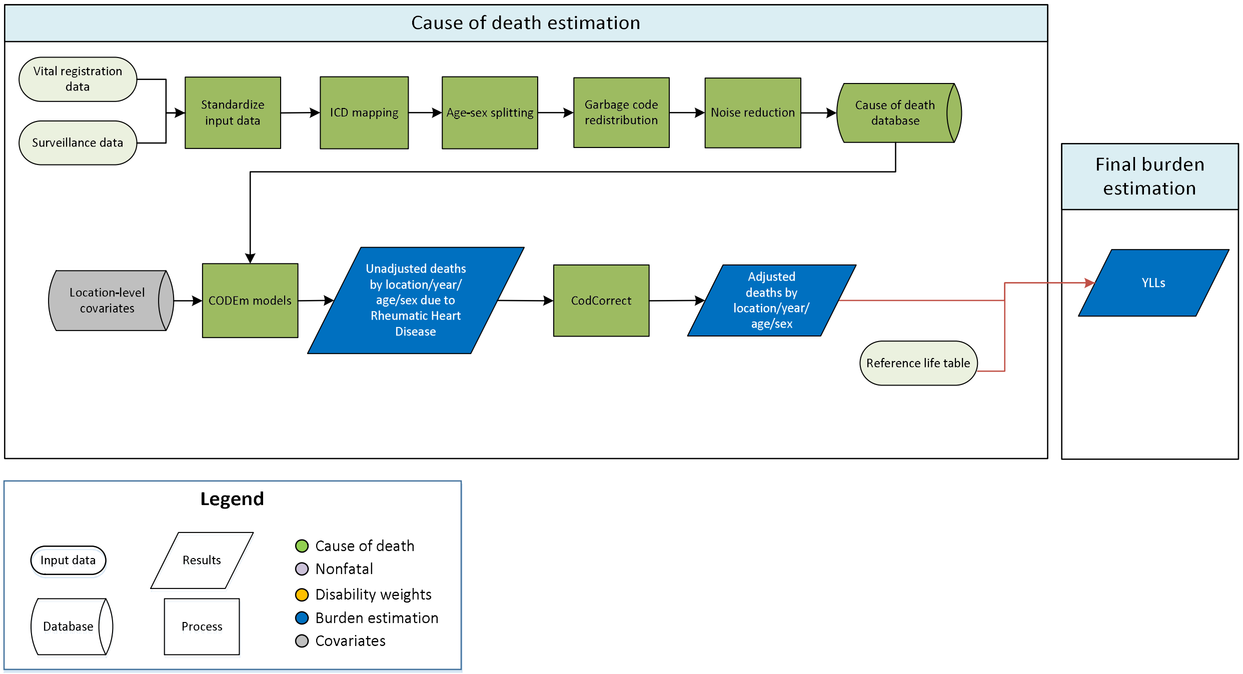

Input data

Vital registration and surveillance data were used to model rheumatic heart disease. We outliered ICD8 and ICD9 BTL datapoints which were inconsistent with the rest of the data and created implausible time trends. We also outliered datapoints which were too high after the redistribution process in a number of age groups. In addition, we outliered verbal autopsy datapoints in Nepal and Pakistan which created an implausibly low cause fraction.

Modelling strategy

We used a standard CODEm approach to model deaths from rheumatic heart disease. There have been no substantive changes from the approach used in GBD 2017, including any covariate changes.

Table 1: Selected covariates for CODEm models, rheumatic heart disease

Level	Covariate	Transformation	Direction
1	Rheumatic heat disease summary exposure value scalar	None	1
1	Improved water (proportion)	None	-1
1	Malnutrition	None	1
1	Sanitation (proportion with access)	None	-1
2	Healthcare access and quality index	None	-1
3	Lag distributed income per capita (I$)	Log	-1
3	Socio-demographic Index	None	-1
3	Education (years per capita)	None	-1

Cardiomyopathy and Myocarditis

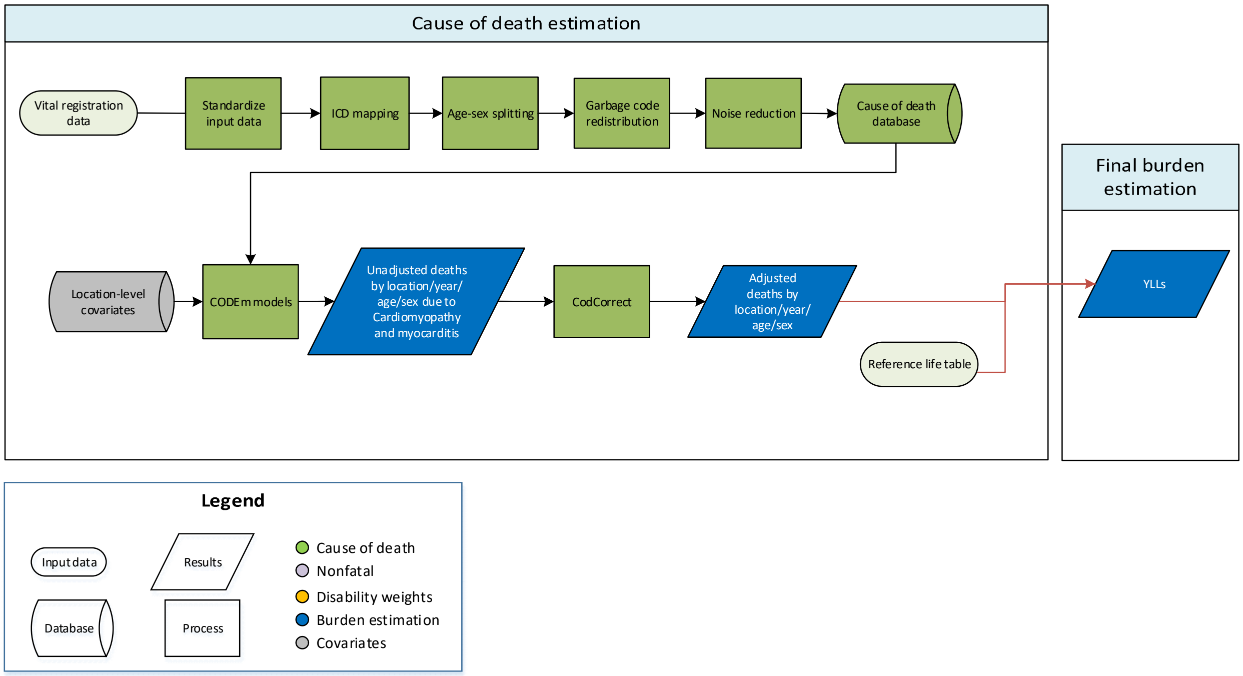

Input data

Vital registration data were used to model deaths due to cardiomyopathy and myocarditis. We outliered data points in Central Asia, Central Europe, and Eastern Europe due to implausibly high values which we attributed to variation in local coding practices. We also outliered ICD8 and ICD9BTL data points in countries where they were discontinuous with other data in the time series or were implausibly high or low. Additionally, we outliered ICD10 data points in Grenada that were improbably low and causing inconsistencies in the time pattern.

Modelling strategy

We used a standard CODEm approach to model deaths from cardiomyopathy and myocarditis. The covariates selected for inclusion in the CODEm modelling process can be found in the table below. A select few changes were made to the covariates as compared with GBD 2017. We dropped the alcohol (litres per capita) covariate as exploratory analyses indicated that it was not predictive of the outcome. We also changed the directions of the socio-demographic index covariate and lag distributed income (per capita) covariate from 0 to -1 to reflect our *a priori* hypotheses about the relationships of these covariates with mortality risk from cardiomyopathy and myocarditis. Aside from these covariate changes, there have been no substantive changes to the modelling strategy since GBD 2017.

Table: Selected covariates for CODEm models, cardiomyopathy and myocarditis

Covariate	Transformation	Level	Direction
Summary exposure value, CMP	none	1	1
Mean systolic blood pressure (mmHg)	none	1	1
Smoking prevalence	none	1	1
Mean BMI (kg/m^2)	None	2	1
Healthcare access and quality index	none	2	-1
Lag distributed income per capita (I$)	log	3	-1
Socio-demographic Index	none	3	-1

Myocarditis

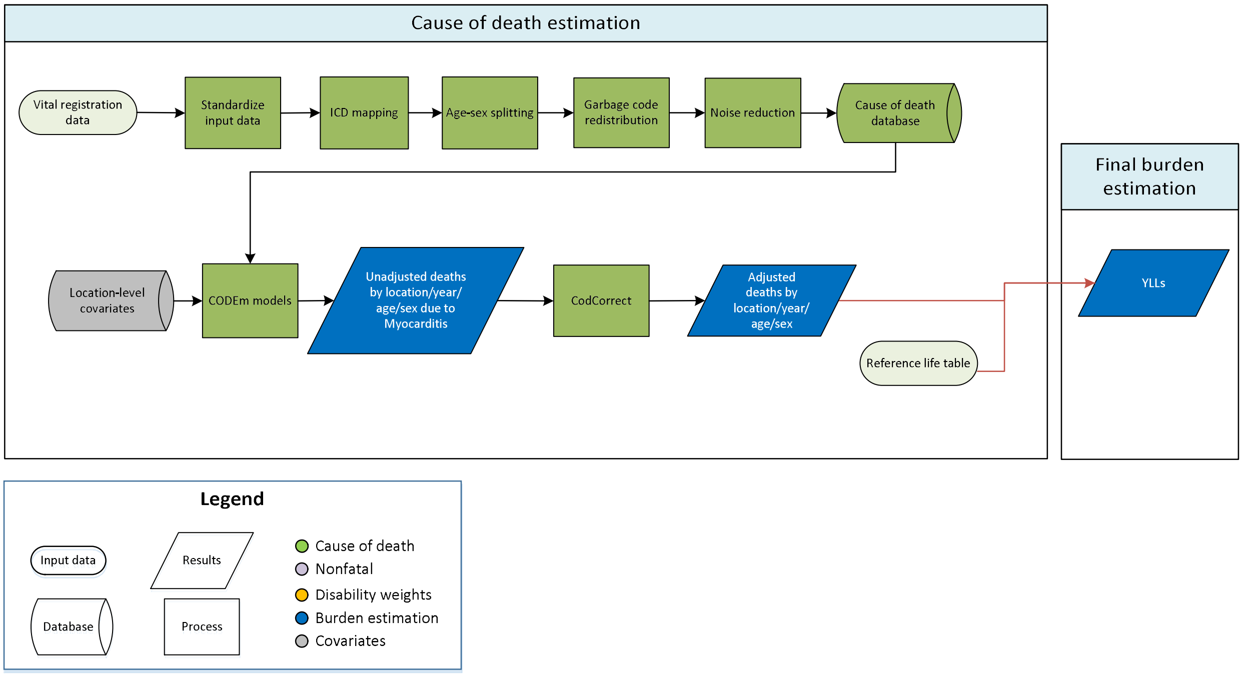

Input data

Vital registration data were used to model deaths due to myocarditis.

Modelling strategy

We used a standard CODEm approach to model deaths from myocarditis. The covariates selected for evaluation in the CODEm ensemble modelling process can be found in the table below. We changed the direction on the lag distributed income per capita and socio-demographic index covariates from 0 for both to -1 and 1, respectively, to reflect our *a priori* hypotheses regarding these associations. Aside from these changes, there have been no substantive changes to the modelling strategy since GBD 2017.

Table: Selected covariates for CODEm models, myocarditis

Covariate	Transformation	Level	Direction
Summary exposure variable, CMP	none	1	1

Systolic blood pressure (mm Hg)	none	1	1
Healthcare access and quality index	none	2	-1
Lag distributed income per capita (I$)	log	3	-1
Socio-demographic Index	none	3	1

Other cardiomyopathy

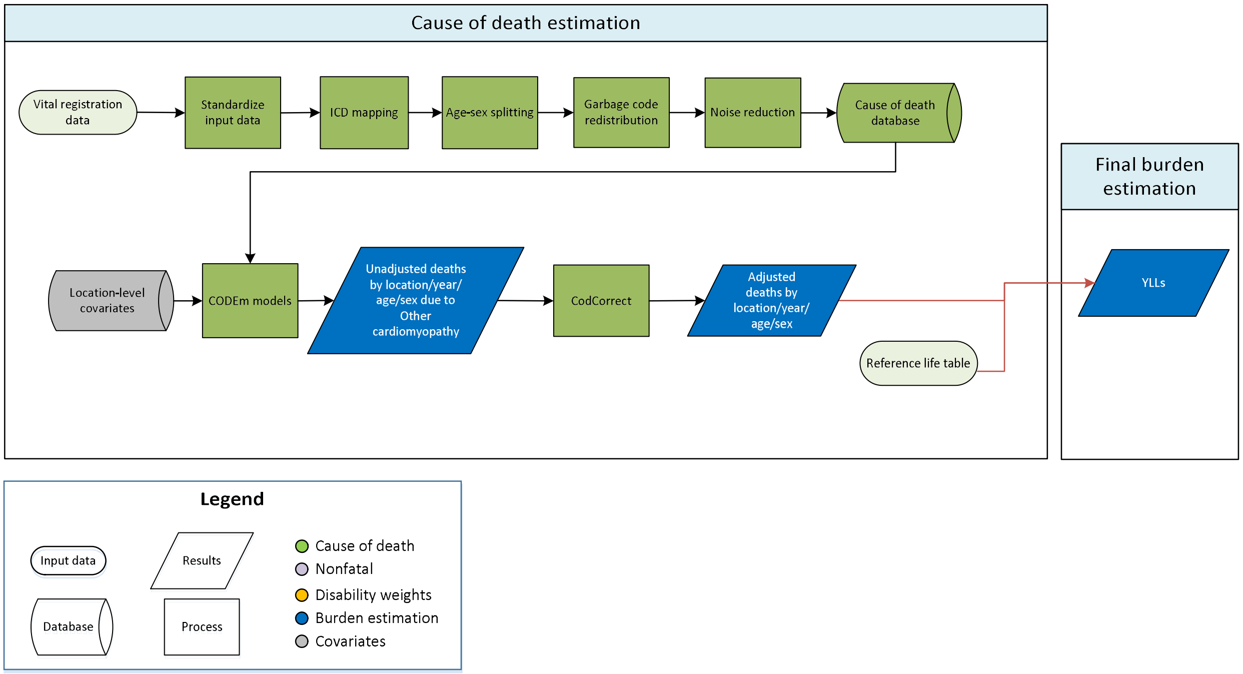

Input data

Vital registration data were used to model deaths due to other cardiomyopathy. We outliered datapoints in Central Asia and Central and Eastern Europe due to implausibly high values which we attributed to variation in local coding practices after review with experts.

Modelling strategy

We used a standard CODEm approach to model deaths from other cardiomyopathy. The covariates selected for inclusion in the CODEm modelling process can be found in the table below. We changed the directions of the Socio-demographic Index and lag distributed income per capita covariates from 0 for both to 1 and -1, respectively. Aside from these covariate changes, there have been no substantive changes to the modelling process since GBD 2017.

Table: Selected covariates for CODEm models, other cardiomyopathy

Level	Covariate	Transformation	Direction
1	Summary exposure variable, CMP	none	1
1	Systolic blood pressure (mmHg)	none	1
1	Smoking prevalence	none	1
2	Body mass index (kg/m^2)	none	1
2	Healthcare Access and Quality Index	none	-1

3	Lag distributed income per capita (I$)	log	-1
3	Socio-demographic Index	none	1

Alcoholic Cardiomyopathy

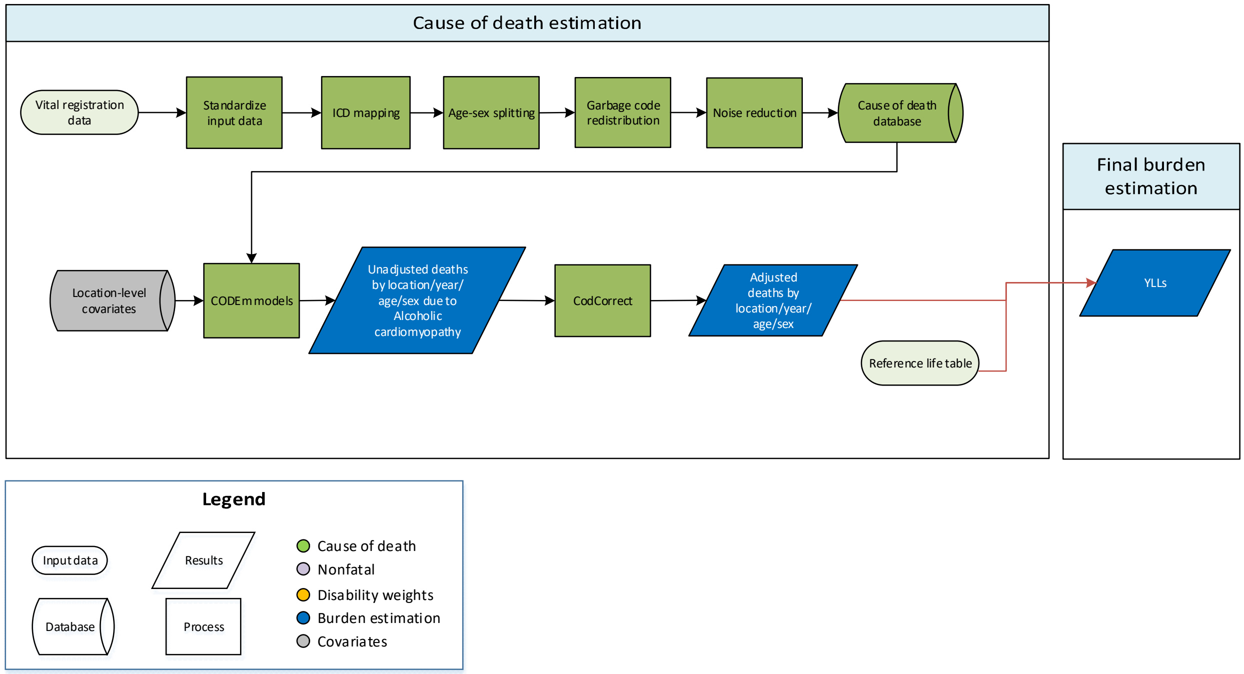

Input data
Vital registration data were used to model deaths due to alcoholic cardiomyopathy. We outliered ICD9 data points in Cyprus that were implausibly high and discontinuous with the rest of the time series. We also dropped ICD9BTL data points in locations in Central and Eastern Europe where we were unable to disaggregate them appropriately. Additionally, we outliered tabulated ICD10 data points in locations where unreliable estimates caused an abrupt inconsistency with detailed ICD10 data.

Modelling strategy
We used a standard CODEm approach to model deaths from alcoholic cardiomyopathy. The covariates selected for inclusion in the CODEm modelling process can be found in the table below. For GBD 2019, we dropped the covariate on socio-demographic index as exploratory analyses indicated that it was not predictive of the outcome. Additionally, we changed the direction of the lag distributed income per capita covariate from 0 to -1 to reflect our *a priori* hypothesis about the expected relationship between this covariate and deaths from alcoholic cardiomyopathy. Aside from these covariate changes, there have been no substantive changes from the approach used in GBD 2017.

Table: Selected covariates for CODEm models, alcoholic cardiomyopathy

Covariate	Transformation	Level	Direction
Summary exposure value, CMP	none	1	1
Smoking prevalence	none	1	1
Alcohol (litres per capita)	none	1	1

Healthcare access and quality index	none	2	-1
Lag distributed income per capita (I$)	log	3	-1

Atrial Fibrillation and Flutter

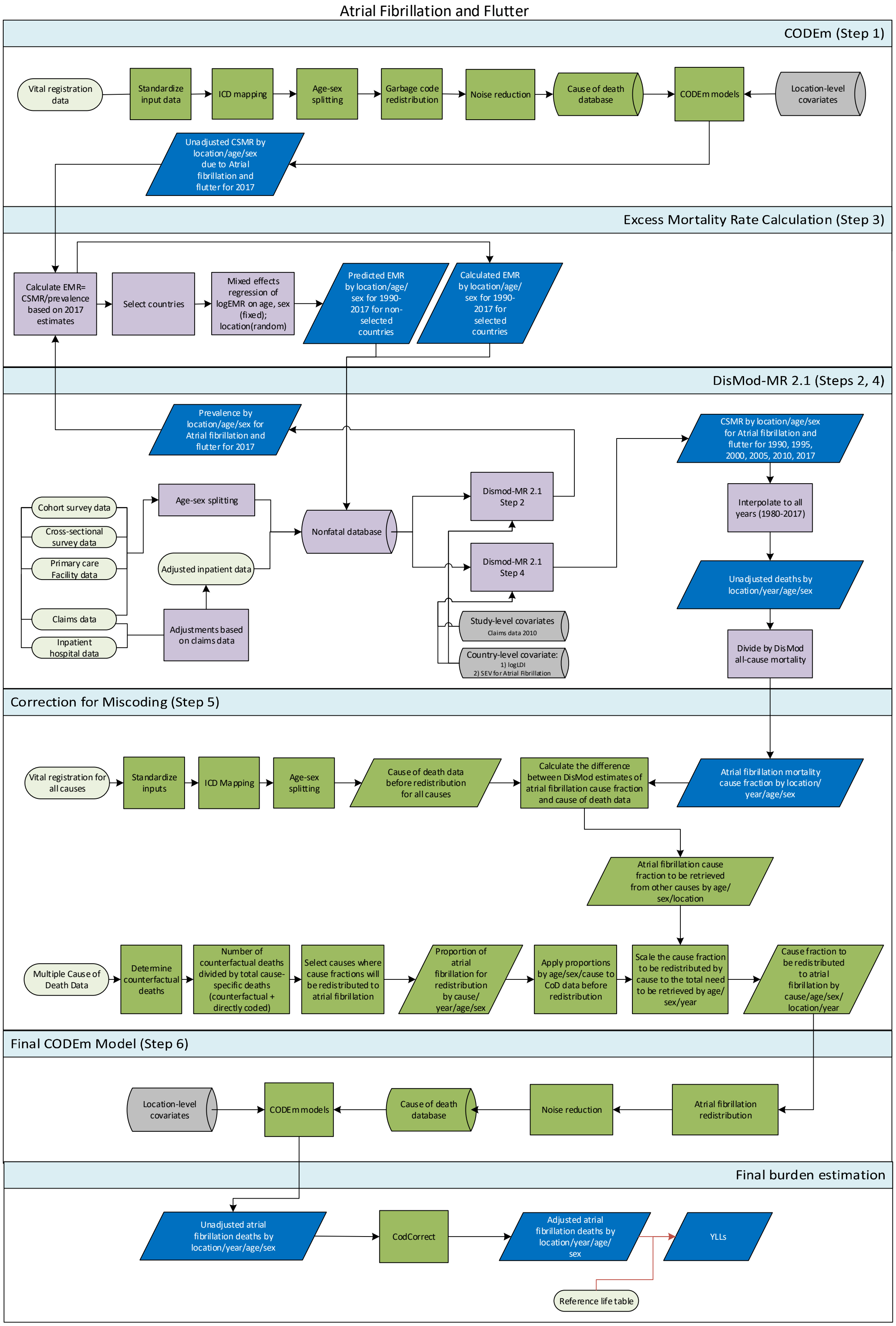

Input data

Vital registration (VR) data: We outliered ICD8 and ICD9 data points that were discontinuous from other data in the time series and created an unlikely time trend. We also outliered data points that were implausibly low in multiple age groups.

Modelling strategy

In order to address changes in coding practices for atrial fibrillation, we used an integrated approach that combined DisMod-MR 2.1 and CODEm models to estimate deaths from atrial fibrillation and flutter. This approach allowed us to adjust estimates to more accurately reflect the number of deaths for which atrial fibrillation was the true underlying cause of death. Due to the restrictions of the decomposition analysis implemented for GBD 2019, we utilized the CSMR from the final GBD 2017 DisMod-MR 2.1 model to inform the misdiagnosis correction described below.

The modelling steps are illustrated in the above flowchart. Covariates included in both the DisMod-MR 2.1 and CODEm models can be found in the table below. In Step 1, we estimated deaths for atrial fibrillation using a standard CODEm approach. In Step 2, we estimated prevalence rates in DisMod-MR 2.1 using data from published reports of cross-sectional and cohort surveys, as well as primary care facility data. We also used claims data covering inpatient and outpatient visits for the United States along with inpatient hospital data from 163 locations in 15 countries. Inpatient hospital data were adjusted using age- and sex-specific information for: 1) readmission within one year; 2) primary diagnosis code to secondary codes; and, 3) the ratio of inpatient to outpatient visits. We set priors of no remission and no excess mortality prior to age 30.

In Step 3, we calculated the excess mortality rate (EMR) for 2017 (defined as the cause-specific mortality rate (CSMR) estimated from CODEm divided by the prevalence rate from DisMod-MR 2.1). We then selected 17 countries based on four conditions: 1) ranking of 4 or 5 stars on the newly developed system for assessing the quality of VR data; 2) prevalence data available from the literature were included in the DisMod-MR 2.1 estimation; 3) prevalence rate ≥ 0.005; and, 4) CSMR ≥ 0.00002. Using information from these countries as input data, we ran a linear mixed-effects regression of logEMR on sex, age, and location. Sex and age were treated as fixed effects for the regression, while location was considered a random effect. We then predicted age- and sex-specific EMR using the results of this regression for all non-selected countries. Countries included in the regression were assigned their directly calculated values. These EMR data points were assigned to the time period 1990–2017 and uploaded into the nonfatal database in order to be used in modelling.

In Step 4, we reran DisMod-MR 2.1 including the EMR estimated in Step 3 as input data using the same priors as in Step 2 to obtain CSMR estimates from DisMod-MR 2.1 that are consistent with the available data for incidence and prevalence. As DisMod-MR 2.1 only generates estimates for six years (1990, 1995, 2000, 2005, 2010, 2017), we interpolated using a log-linear approach for 1990–2017. Estimates for 1980–1990 were generated via regression on the entire time series, using sociodemographic index as a predictor.

In Step 5, the CSMR estimates were divided by the all-cause mortality estimates used in DisMod-MR 2.1 to calculate the cause fraction for atrial fibrillation and flutter. We then calculated the difference between the cause fraction estimated by DisMod-MR 2.1 and the cause fraction in the VR data generated by the Cause of Death data preparation process. This yielded the cause fraction that would need to be retrieved from other causes via the process described in Section 2.6: Correction for miscoding of Alzheimer's and other dementias and Parkinson's disease. After this correction process, the cause fraction data are processed through the standard redistribution and noise reduction processes.

In Step 6, these adjusted cause fraction data are then used as inputs for a final CODEm model, using the covariates described below. The results from the CODEm model are processed through CoDCorrect; these post-CoDCorrected results are the final estimates for cause-specific mortality for atrial fibrillation and flutter.

Modelling strategy

We used a standard CODEm approach to model deaths from ischemic heart disease. For GBD 2019, adjusted dietary covariates for consumption of fruits, omega-3 fatty acids, vegetables, nuts and seeds, and polyunsaturated fatty acids were replaced with the summary exposure value scalars for diet low in each of these factors. The direction for each dietary covariate was changed from -1 to 1 to as our *a priori* assumption is that low levels of intake of these dietary factors are associated with increasing mortality risk from ischaemic heart disease. We changed the direction of the alcohol variable from 0 to 1 to reflect our *a priori* hypothesis about the expected direction of the association between this risk factor and mortality risk of ischaemic heart disease. In addition, we changed the level of the covariate for trans fatty acid from 1 to 3. Besides these covariate changes, there are no other substantive changes from the approach used in GBD 2017.

For GBD 2019, adjusted dietary covariates for consumption of fruits, omega-3 fatty acids, vegetables, nuts and seeds, and polyunsaturated fatty acids were replaced with the summary exposure value scalars for diet low in each of these factors. The direction for each dietary covariate was changed from -1 to 1 to as our *a priori* assumption is that low levels of intake of these dietary factors are associated with increasing mortality risk from atrial fibrillation. In addition, the dietary covariate for whole grains (kcal/capita, adjusted) was dropped as exploratory analyses indicated that it was not associated with mortality risk. The direction for the alcohol and socio-demographic index covariates was changed from 0 to 1 to reflect our *a priori* hypotheses about the expected directions of the associations between these covariates and mortality risk of atrial fibrillation. Besides these covariate changes, there are no other substantive changes from the approach used in GBD 2017.

CODEm Covariates, atrial fibrillation and flutter

Covariate	Transformation	Level	Direction
Summary exposure variable, atrial fibrillation	None	1	1
Smoking prevalence	None	1	1
Systolic blood pressure (mmHg)	None	1	1
Mean BMI	None	2	1
Fasting plasma glucose	None	2	1
Healthcare Access and Quality Index	None	2	-1
Cholesterol (total, mean per capita)	None	2	1
Lag distributed income per capita (I$)	Log	3	-1
Socio-demographic Index	None	3	1
Summary exposure value, omega-3	None	3	1
Summary exposure value, fruits	None	3	1
Summary exposure value, vegetables	None	3	1
Summary exposure value, nuts and seeds	None	3	1
Pulses/legumes (kcal/capita, unadjusted)	None	3	-1
Summary exposure value, PUFA	None	3	1
Alcohol (litres per capita)	None	3	1
Trans fatty acid	None	3	1

DisMod-MR 2.1 Covariates – Step 2

Covariate	Parameter	Beta	Exponentiated beta
All MarketScan, year 2010	Prevalence	-0.077 (-0.099 to -0.051)	0.93 (0.91 to 0.95)
SEV scalar: Atrial fibrillation	Prevalence	0.75 (0.75 to 0.75)	2.12 (2.12 to 2.12)
Healthcare access and quality index	Excess mortality rate	-0.11 (-0.13 to -0.088)	0.90 (0.88 to 0.92)

DisMod-MR 2.1 Covariates – Step 4

Covariate	Parameter	Beta	Exponentiated beta
All MarketScan, year 2010	Prevalence	0.017 (-0.013 to 0.040)	1.02 (0.99 to 1.04)
SEV scalar: Atrial fibrillation	Prevalence	0.75 (0.75 to 0.75)	2.12 (2.12 to 2.12)
LDI (I$ per capita)	Excess mortality rate	-0.1 (-0.1 to -0.1)	0.90 (0.90 to 0.90)

Aortic Aneurysm

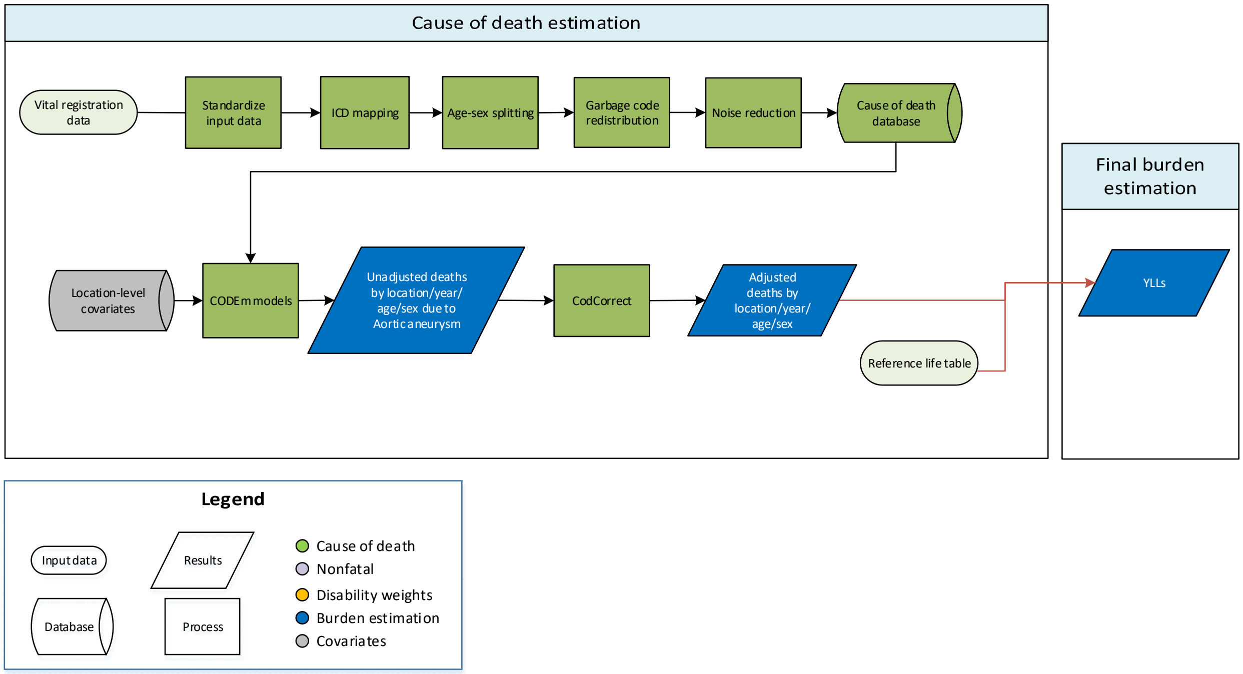

Input data

Vital registration data were used to model cause-specific mortality for aortic aneurysm. We outliered data in Oman as they were improbably high in comparison with the rest of the region. We also outliered ICD8 data that were discontinuous with the rest of the time series and created implausible time trends. In addition, we outliered a subset of vital registration data points in Latin America due to implausibly high values at the oldest age groups that resulted in inconsistencies in time trends.

Modelling strategy

We used a standard CODEm approach to model deaths from aortic aneurysm. The covariates selected for inclusion in the CODEm modelling process can be found in the table below. For GBD 2019, adjusted

dietary covariates for consumption of fruits, omega-3 fatty acids, vegetables, nuts and seeds, and polyunsaturated fatty acids were replaced with the summary exposure value scalars for diet low in each of these factors. The direction for each dietary covariate was changed from -1 to 1 to as our *a priori* assumption is that low levels of intake of these dietary factors are associated with increasing mortality risk from aortic aneurysm. We also changed the direction of the covariates for alcohol consumption and the socio-demographic index from 0 to 1. Besides these covariate changes, there are no other substantive changes from the approach used in GBD 2017.

Table: Selected covariates for CODEm models, aortic aneurysm

Covariate	Transformation	Level	Direction
Summary exposure variable, aortic aneurysm	None	1	1
Cholesterol (total, mean per capita)	None	1	1
Cumulative cigarettes (10 yrs)	None	1	1
Systolic blood pressure (mmHg)	None	1	1
Mean BMI	None	2	1
Healthcare access and quality index	None	2	-1
Lag distributed income per capita (I$)	Log	3	-1
Socio-demographic Index	None	3	1
Summary exposure value omega-3	None	3	1
Summary exposure value fruits	None	3	1
Summary exposure value vegetables	None	3	1
Summary exposure value nuts and seeds	None	3	1
Pulses/legumes (kcal/capita, un-adjusted)	None	3	-1
Summary exposure value PUFA	None	3	1
Alcohol (litres per capita)	None	3	1

Non-rheumatic valvular heart disease: Non-rheumatic calcific aortic valve disease, non-rheumatic degenerative mitral valve disease, and other non-rheumatic valvular heart diseases

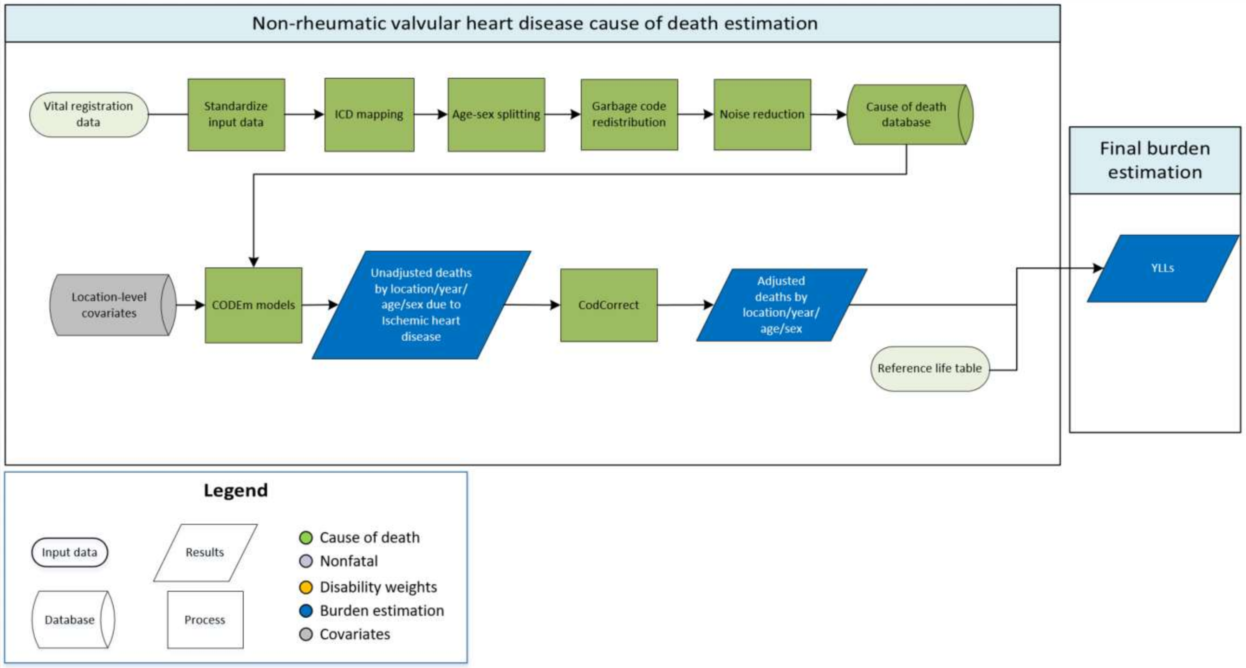

Input data

Vital registration data were used to model non-rheumatic valvular heart disease, non-rheumatic calcific valve disease, non-rheumatic degenerative mitral valve disease, and other non-rheumatic valve

diseases. We outliered ICD8, ICD9BTL, and tabulated ICD10 datapoints which were inconsistent with the rest of the data and created implausible time trends. Datapoints from sources which were implausibly low in all age groups and datapoints that were causing the regional estimates to be improbably high were outliered.

Modelling strategy

We used a standard CODEm approach to model deaths from non-rheumatic valvular heart disease, non-rheumatic calcific valve disease, non-rheumatic degenerative mitral valve disease, and other non-rheumatic valvular diseases. The covariates used in the GBD 2019 models, along with their transformations, importance levels, and imposed directions are reported by cause in the tables below. For non-rheumatic valvular heart disease and non-rheumatic calcific aortic valve disease, we added the appropriate summary exposure value, setting both the direction and level to 1. We changed the direction of the Socio-demographic Index covariate from 0 to 1; this change affected the non-rheumatic valve disease, non-rheumatic calcific aortic valve disease, and non-rheumatic degenerative mitral valve disease models. We also changed the direction of the alcohol consumption variable from 0 to 1; this update affected the non-rheumatic valvular heart disease and calcific aortic valve disease models. All covariates for the other non-rheumatic valvular heart disease model were changed. In GBD 2017, we had included only the summary exposure value for cardiovascular diseases in the model. For GBD 2019, we updated the model to include the summary exposure value for non-rheumatic valvular heart disease (level 1, direction 1), Healthcare Access and Quality Index (level 1, direction -1), and Socio-demographic Index (level 2, direction -1).

Table 1: Selected covariates for CODEm models, non-rheumatic valvular heart disease

Level	Covariate	Transformation	Direction
1	Smoking prevalence	None	1
1	Summary exposure value, non-rheumatic valve disease	None	1
1	Systolic blood pressure (mmHg)	None	1
2	Cholesterol (total, mean per capita)	None	1
2	Mean BMI	None	1
2	Healthcare Access and Quality Index	None	-1
3	Lag distributed income per capita (I$)	Log	-1
3	Socio-demographic Index	None	1
3	Alcohol (litres per capita)	None	1

Table 2: Selected covariates for CODEm models, non-rheumatic calcific aortic valve disease

Level	Covariate	Transformation	Direction
1	Smoking prevalence	None	1
1	Summary exposure value, non-rheumatic calcific aortic valve disease	None	1
1	Systolic blood pressure (mmHg)	None	1
2	Cholesterol (total, mean per capita)	None	1
2	Mean BMI	None	1
2	Fasting plasma glucose	None	1
2	Healthcare Access and Quality Index	None	-1
3	Lag distributed income per capita (I$)	Log	-1

3	Socio-demographic Index	None	1
3	Alcohol (litres per capita)	None	1

Table 3: Selected covariates for CODEm models, non-rheumatic degenerative mitral valve disease

Level	Covariate	Transformation	Direction
1	Healthcare Access and Quality Index	None	-1
1	Lag distributed income per capita (I$)	Log	1
1	Socio-demographic Index	None	1

Table 4: Selected covariates for CODEm models, other non-rheumatic valvular heart diseases

Level	Covariate	Transformation	Direction
1	Summary exposure value, non-rheumatic valve disease	None	1
1	Healthcare Access and Quality Index	None	-1
2	Socio-demographic Index	None	-1

Endocarditis

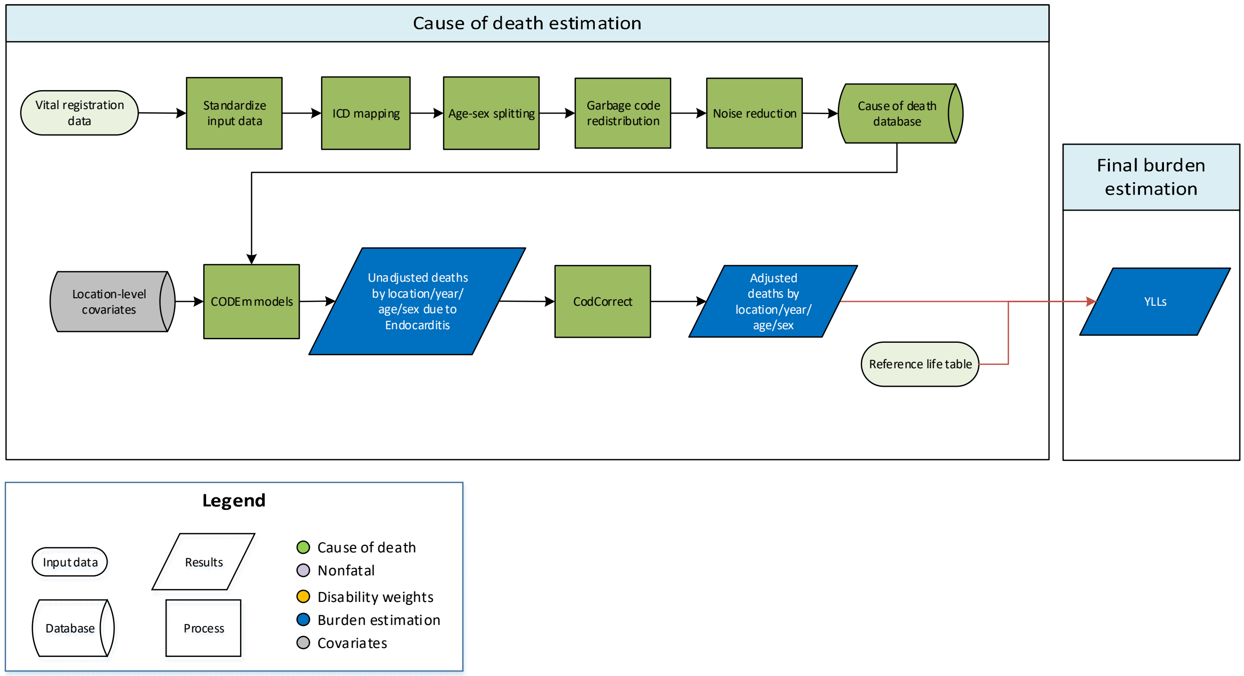

Input data

Vital registration data were used to model endocarditis. We outliered data in Mozambique as these were non-representative for sub-Saharan Africa and were causing regional estimates to be implausibly low. We also outliered ICD8 data that were discontinuous from the rest of the data series and created an implausible time trend.

Modelling strategy

We used a standard CODEm approach to model deaths from endocarditis. Covariates selected for inclusion in the CODEm ensemble modelling process are listed in the table below. For GBD 2019, the same covariates as GBD 2017 were used. We changed the level of the healthcare access and quality index covariate from 1 to 2 for consistency with our *a priori* hypothesis about the relative impact of the covariate on mortality from endocarditis. We also changed the direction of the socio-demographic index covariate from 0 to -1. Apart from these updates to the covariates, there have been no substantive changes from the approach used in GBD 2016.

Table: Selected covariates for CODEm models, endocarditis

Covariate	Transformation	Level	Direction
Summary exposure value, endocarditis	None	1	1
Improved water (proportion)	None	1	-1
Sanitation (proportion with access)	None	1	-1
Healthcare access and quality index	None	2	-1
Lag distributed income per capita (I$)	Log	3	-1
Socio-demographic Index	None	3	-1

Peripheral artery disease

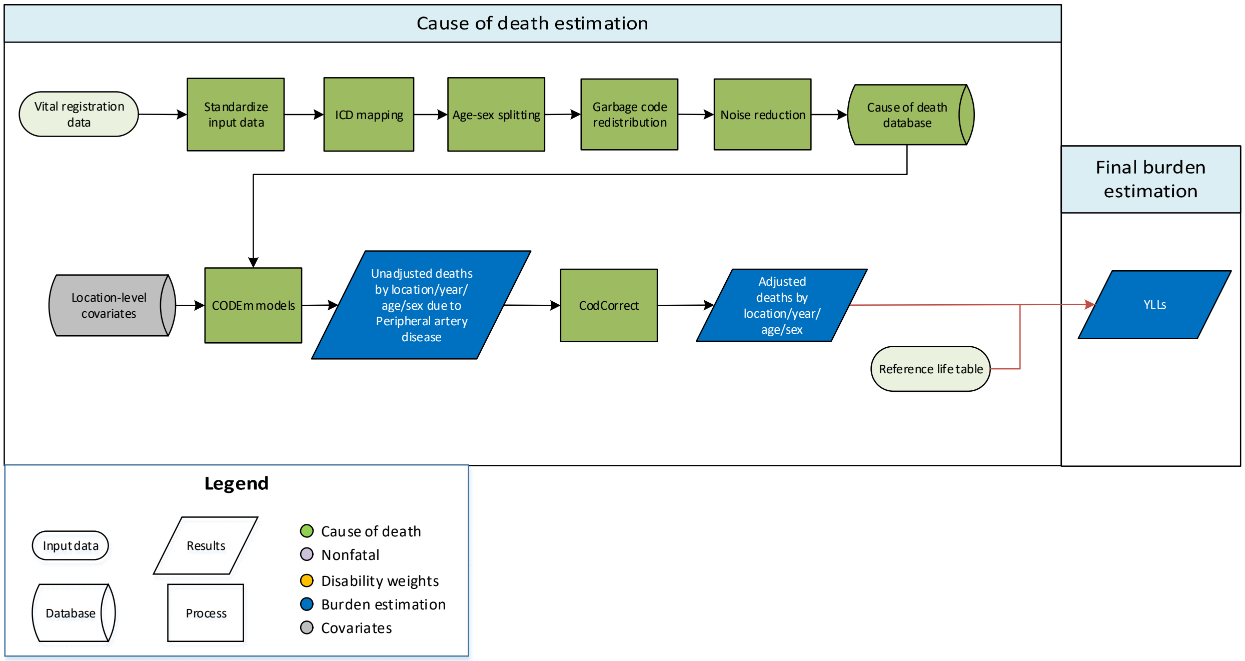

Input data

Vital registration data were used to model peripheral artery disease. We outliered all datapoints with less than 1 death in Egypt per expert review.

Modelling strategy

We used a standard CODEm approach to model deaths from peripheral artery disease. For GBD 2019, adjusted dietary covariates for consumption of fruits, omega-3 fatty acids, vegetables, nuts and seeds, and polyunsaturated fatty acids were replaced with the summary exposure value scalars for diet low in each of these factors. The direction for each dietary covariate was changed from -1 to 1 to as our a priori assumption is that low levels of intake of these dietary factors are associated with increasing mortality risk from peripheral arterial disease. In addition, we dropped the dietary covariates for whole grains (kcal/capita, adjusted) and trans fatty acid (percent). We changed the direction of the alcohol and the Socio-demographic Index covariates from 0 to 1 to reflect the expected direction of the association for these risk factors with mortality risk. Apart from these changes, there are no substantive changes from the approach used in GBD 2017.

Table: Selected covariates for CODEm models, peripheral artery disease

Level	Covariate	Transformation	Direction
1	Summary exposure variable, PAD	None	1
1	Systolic blood pressure (mmHg)	None	1
1	Cholesterol (total, mean per capita)	None	1
1	Smoking prevalence	None	1
2	Mean body mass index (kg/m^2)	None	1
2	Healthcare Access and Quality Index	None	-1
2	Diabetes fasting plasma glucose (mmol/L)	None	1
3	Lag distributed income per capita (I$)	Log	-1
3	Socio-demographic Index	None	1
3	Summary exposure value, omega-3	None	1
3	Summary exposure value, fruits	None	1
3	Summary exposure value, vegetables	None	1
3	Summary exposure value, nuts and seeds	None	1
3	Pulses/legumes (kcal/capita, unadjusted)	None	-1
3	Summary exposure value, polyunsaturated fatty acids	None	1
3	Alcohol (litres per capita)	None	1

Non-fatal cause-specific modelling descriptions

Ischaemic heart disease

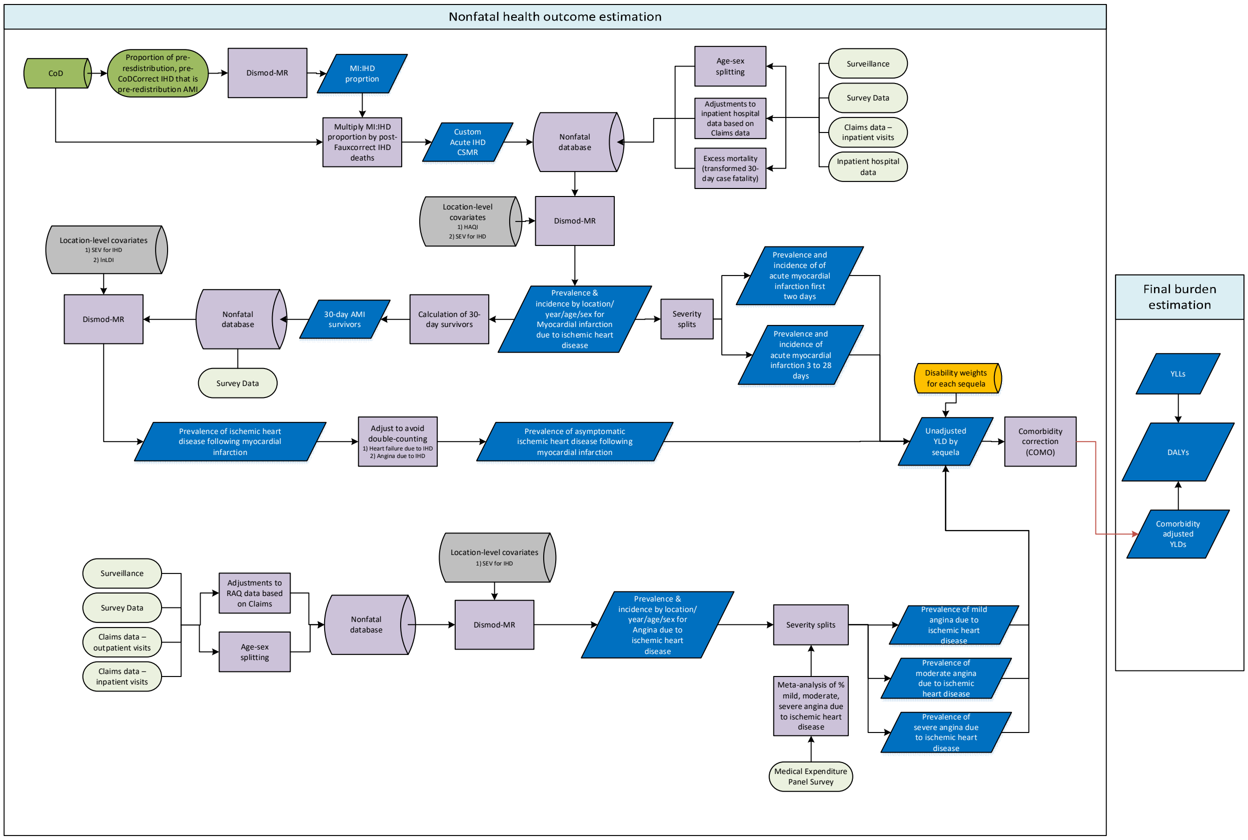

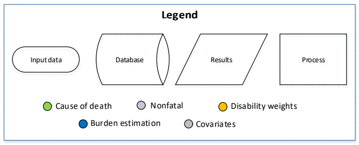

Input data and methodological summary

Case definition

<u>Case definitions:</u>

1) Acute myocardial infarction (MI): Definite and possible MI according to the third universal definition of myocardial infarction:
    a. When there is clinical evidence of myocardial necrosis in a clinical setting consistent with myocardial ischaemia or
    b. Detection of a rise and/or fall of cardiac biomarker values and with at least one of the following: i) symptoms of ischaemia, ii) new or presumed new ST-segment-T wave changes or new left bundle branch block, iii) development of pathological Q waves in the ECG, iv) imaging evidence of new loss of viable myocardium or new regional wall motion abnormality, or v) identification of an intracoronary thrombus by angiography or autopsy.
    c. Sudden (abrupt) unexplained cardiac death, involving cardiac arrest or no evidence of a non-coronary cause of death
    d. Prevalent MI is considered to last from the onset of the event to 28 days after the event and is divided into an acute phase (0–2 days) and subacute (3–28 days).

2) Chronic IHD
    a. Angina; clinically diagnosed stable exertional angina pectoris or definite angina pectoris according to the Rose Angina Questionnaire, physician diagnosis, or taking nitrate medication for the relief of chest pain.
    b. Asymptomatic ischaemic heart disease following myocardial infarction; survival to 28 days following incident MI. The GBD study does not use estimates based on ECG evidence for prior MI, due to its limited specificity and sensitivity (1).

ICD codes used for inclusion of hospital and claims data for MI and angina can be found elsewhere in the appendix.

Input data

The total source counts for non-fatal ischaemic heart disease are shown in the table below by measure.

Table 1: Source counts for all non-fatal ischaemic heart disease models.

Measure	Total sources	Countries with data
All measures	442	84
Prevalence	88	61
Incidence	296	44
Excess mortality rate	90	21
Relative risk	1	1

Standardized mortality ratio	1	1
With-condition mortality rate	4	4
Proportion	16	1

Myocardial infarction

A systematic review was done for myocardial infarction for GBD 2019 in order to update our current database. The search strings used were ((“myocardial infarction”[tiab] AND (incidence OR “case fatality” OR “excess mortality”)) OR (“acute coronary syndrome”[tiab] AND (incidence OR “case fatality” OR “excess mortality”)) OR (angina[tiab] AND (incidence OR prevalence OR “case fatality” OR “excess mortality”))) AND ("2019/01/01"[PDAT] : "2019/12/31"[PDAT]) NOT rat[tiab] NOT mice[tiab] NOT monkey[tiab] NOT pig[tiab] NOT animals[tiab].

The dates of the search were 1/1/2019 – 12/31/2019. 28957 studies were returned, 80 were extracted. The PRISMA diagram for the systematic review is given below. In the diagram, screening refers to reviewing of the title and abstract of an article for relevant information, not screening of the entire article.

PRISMA Diagram

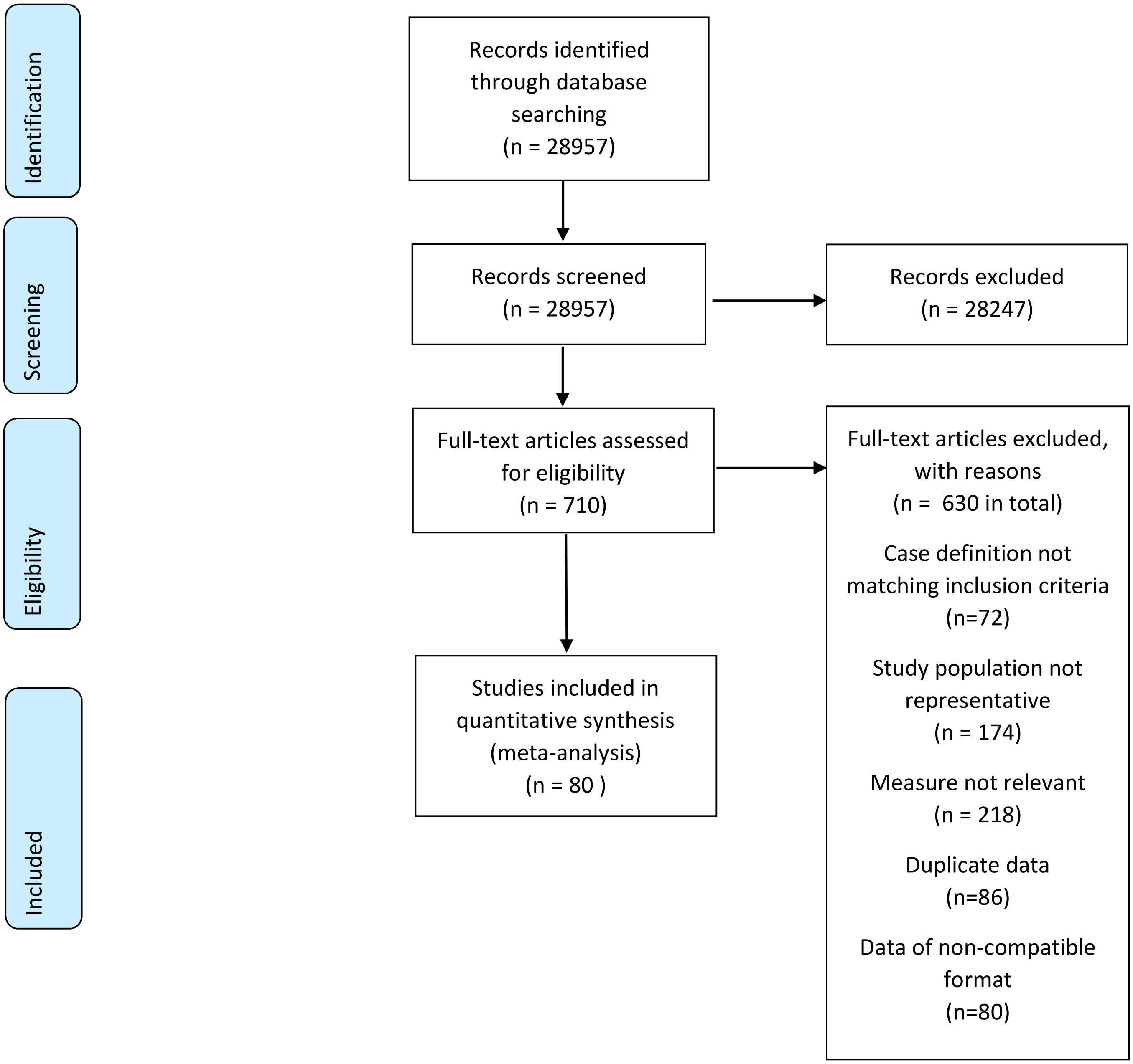

The last systematic review for myocardial infarction was done for GBD 2015. The dates of the search were 1/1/2009 – 2/3/2015. 38,522 studies were returned; 194 were extracted (this number includes extractions that were done for STEMI/NSTEMI models and revascularisation models that are not currently part of the MI modelling process but may be in the future).

A systematic review for myocardial infarction was also done for GBD 2013. The extensive search terms for that review will be provided on request.

Apart from inpatient hospital and inpatient claims data, we did not include any data from sources other than the literature for myocardial infarction. We also split excess mortality data points where the age range was greater than 25 years. Age splitting was based on the global sex-specific age pattern from a Dismod model that only used excess mortality input data from scientific literature with less than a 25-year age range. We excluded incidence data with broad age ranges where it was impossible to obtain more granular data, as these data caused the known age pattern for increased risk of myocardial infarction to be masked in the estimates generated from DisMod.

We crosswalked incidence measurements for myocardial infarction literature data with alternative definitions to agree with our case reference definition using MR-BRT (Meta Regression – Bayesian, Regularized, Trimmed) modeling tool. MR-BRT and the process of data adjustment are discussed elsewhere in the appendix. For myocardial infarction we crosswalked using multiple different covariates: a covariate to capture only first-ever MI, using studies where all events were included as the reference; a covariate to adjust estimates from studies that only included non-fatal cases, using sources that included fatal and non-fatal cases as reference; and a covariate to adjust for studies that did not use troponin measurements in their case diagnosis, using sources that did include troponin measurements in their diagnostic method. The coefficients in Table 2 below can be used to calculate adjustment factors for alternative definitions. The formula for computing adjustment factors is given in equation 1 below. We also included a standardized age variable (age scaled) and a sex variable to the regression to adjust for the possibly of bias.

Equation 1: Calculation of adjustment factors:

$$Estimated\ Reference\ Def = invlogit(logit(Alternative\ Def) - Beta_{Alternative\ Def} - Beta_{Sex} * Sex - Beta_{Age_{scaled}} * Age\ Scaled)$$

Table 2a: MR-BRT Crosswalk Adjustment Factors for Myocardial Infarction

Data input	Measure	Reference or alternative case definition	Gamma	Beta Coefficient, Logit (95% CI)
Any event, fatal and nonfatal events, used troponin	Incidence	Ref	0.27	---
Troponin not used as part of definition	Incidence	Alt		-0.55 (-1.08 - -0.01)
First-ever	Incidence	Alt		-0.59 (-1.21 – 0.03)
Non-fatal	Incidence	Alt		-0.35 (-0.98 – 0.29)
Age scaled	Incidence	Alt		-0.05 (-0.59 – 0.49)
Sex (male)	Incidence	Alt		-0.001 (-0.54 – 0.54)

<u>Asymptomatic ischaemic heart disease following myocardial infarction</u>

No systematic review was performed for Asymptomatic ischaemic heart disease following myocardial infarction in GBD 2019. The primary input for this model are 28-day survivors calculated from the excess mortality estimates for the myocardial infarction model. We included data for excess mortality and standardised mortality ratio to inform the estimates of survival after myocardial infarction.

Angina

A systematic review was not performed for GBD 2019. Updates to systematic reviews are performed on an ongoing schedule across all GBD causes; an update for angina will be performed in the next one to two iterations.

A systematic review for angina was last performed for GBD 2013. The search terms for that are: (Angina Pectoris/epidemiology[Mesh] OR Angina Pectoris/mortality[Mesh]) AND (prevalence[Title/Abstract] OR incidence[Title/Abstract]) AND ("2010"[Date - Publication] : "3000"[Date - Publication])

We included survey data (including NHANES and World Health Study questionnaires) which included the RAQ items. Prevalence of angina was calculated using the standard algorithm to determine whether the RAQ was positive or negative.

We excluded data with broad age ranges where it was impossible to obtain more granular data, as these data caused the known age pattern for increased risk of angina to be masked in the estimates generated from DisMod.

We also included US claims data, but did not include inpatient hospital data from any locations. Stable angina (unstable angina is modeled as part of MI) is expected to be rare in inpatient but common in outpatient data as it is a condition usually managed on an outpatient basis, except for specific surgical interventions. This discrepancy leads to implausible correction factors based on inpatient/outpatient information from claims data (~150X); thus adjusted data cannot be used. Including uncorrected data in the model is likely to lead to incorrect estimates as hospitalisation and procedure rates are likely to vary between geographies based on access to and patterns of care. All outpatient data were excluded as they were implausibly low for all locations when compared with literature and claims data.

We crosswalked prevalence data obtained from survey data using the RAQ using claims data as a reference since the RAQ has been shown to be neither sensitive nor specific. Specifics on the crosswalking process are discussed elsewhere in the appendix. Table 2b shows the coefficients adjustments made to the alternative definition.

Table 2b: MR-BRT Crosswalk Adjustment Factors for Angina

Data input	Measure	Reference or alternative case definition	Gamma	Beta Coefficient, Logit (95% CI)
United States Claims Data	Prevalence	Ref	0.11	---
Rose Angina Questionnaire	Prevalence	Alt		2.21 (1.97 to 2.44)
Age (scaled)	Prevalence	Alt		-0.97 (-1.20 to -0.74)
Sex (male)	Prevalence	Alt		-0.62 (-0.86 to -0.38)

Severity split inputs

Acute myocardial infarction was split into two severity levels by length of time since the event – days 1 and 2 versus days 3 through 28. Disability weights were established for these two severities using the standard approach for GBD 2019.

Asymptomatic ischaemic heart disease following myocardial infarction was all assigned to the asymptomatic severity level. No disability weight is assigned to this level.

Angina was split into asymptomatic, mild, moderate, and severe groups using information from MEPS. Disability weights were established for these severities using the standard approach for GBD 2019.

<u>Acute myocardial infarction</u>

Table 3a. Severity distribution, details on the severity levels for Myocardial Infarction in GBD 2019 and the associated disability weight (DW) with that severity.

Severity level	Lay description	DW (95% CI)
Acute myocardial infarction, days 1-2	Has severe chest pain that becomes worse with any physical activity. The person feels nauseated, short of breath, and very anxious.	0.432 (0.288–0.579)
Acute myocardial infarction, days 3-28	Gets short of breath after heavy physical activity, and tires easily, but has no problems when at rest. The person has to take medication every day and has some anxiety.	0.074 (0.049–0.105)

<u>Asymptomatic ischaemic heart disease following myocardial infarction</u>

Table 3b. Severity distribution, details on the severity levels for <u>Asymptomatic ischaemic heart disease following myocardial infarction</u> in GBD 2019 and the associated disability weight (DW) with that severity.

Severity level	Lay description	DW (95% CI)

Asymptomatic ischaemic heart disease		N/A

Angina pectoris

Table 3c. Severity distribution, details on the severity levels for Angina pectoris in GBD 2019 and the associated disability weight (DW) with that severity.

Severity level	Lay description	DW (95% CI)
Asymptomatic angina		N/A
Mild angina	Has chest pain that occurs with strenuous physical activity, such as running or lifting heavy objects. After a brief rest, the pain goes away.	0.033 (0.02–0.052)
Moderate angina	Has chest pain that occurs with moderate physical activity, such as walking uphill or more than half a kilometer (around a quarter-mile) on level ground. After a brief rest, the pain goes away.	0.08 (0.052–0.113)
Severe angina	Has chest pain that occurs with minimal physical activity, such as walking only a short distance. After a brief rest, the pain goes away. The person avoids most physical activities because of the pain.	0.167 (0.11–0.24)

Modelling strategy

Myocardial infarction

- We first calculated custom cause-specific mortality estimates using cause of death data prior to garbage code redistribution, generating age-sex-country-specific proportions of IHD deaths that were due to MI (acute IHD) versus those due to other causes of IHD (chronic IHD). Estimates of this proportion for all locations were then generated using a DisMod proportion-only model. Due to a high degree of variability in pre-redistribution coding practices by location, we used the global age-, sex-, and year-specific proportions of acute deaths in subsequent calculations. The global proportions were multiplied by post-Fauxcorrect (final GBD 2019 CoD estimates with GBD 2017 scalers) IHD deaths by location to generate CSMR estimates for MI. These data, along with incidence and excess mortality data, informed a DisMod model to estimate the prevalence and incidence of myocardial infarction due to ischaemic heart disease.
- These estimates were split into estimates for days 1-2 and days 3-28 post-event. Disability weights were assigned to each of these two groupings.
- We set a value prior of one month for remission (11/13) from the MI model. We also set a value prior for the maximum excess mortality rate of 10 for all ages. We included the Healthcare Access and Quality (HAQ) Index as a fixed-effect country-level covariate on excess mortality, forcing an inverse relationship.

Table 4a. Covariates. Summary of covariates used in the Myocardial Infarction DisMod-MR meta-regression model

Covariate	Parameter	Beta	Exponentiated beta
Healthcare Access and Quality (HAQ) Index	Excess mortality rate	-0.01 (-0.01 to -0.01)	0.99 (0.99 to 0.99)
Log-transformed age-standardised SEV scalar: IHD	Incidence	0.75 (0.75 to 0.76)	2.12 (2.12 to 2.13)

Asymptomatic ischaemic heart disease

- Excess mortality estimates from the myocardial infarction model were used to generate data of the incidence of surviving 28 days post-event.
- We used these data, along with the estimates of CSMR due to chronic IHD (the other part of the proportion described in step 1) and excess mortality data in a DisMod model to estimate the prevalence of persons with IHD following myocardial infarction. This estimate included subjects with angina and heart failure; a proportion of this prevalence was removed in order to avoid double-counting based on evidence from the literature (2). The result of this step generates estimates of asymptomatic ischaemic heart disease following myocardial infarction.
- We set a value prior of 0 for remission for all ages.
- We also included the log-transformed, age-standardised SEV scalar for IHD as a fixed effect, country-level covariate on prevalence and LDI (I$ per capita) as a fixed-effect country-level covariate on excess mortality, forcing an inverse relationship for LDI.

Table 4b. Covariates. Summary of covariates used in Asymptomatic Ischaemic Heart Disease DisMod-MR meta-regression model

Covariate	Parameter	Beta	Exponentiated beta
LDI (I$ per capita)	Excess mortality rate	-0.28 (-0.45 to -0.13)	0.76 (0.63 to 0.88)
Log-transformed age-standardised SEV scalar: IHD	Incidence	1.00 (0.77 to 1.24)	2.72 (2.15 to 3.47)

Angina

- We used prevalence data from the literature and USA claims databases, along with data on mortality risk to estimate the prevalence and incidence of angina for all locations. Data which used the Rose Angina Questionnaire to determine prevalence of angina was adjusted using MR-BRT as described above.
- The proportion of mild, moderate, and severe angina was determined by the standard approach for severity splitting for GBD 2019.
- We included a value prior of 0 for remission for all ages. We also included a value prior of 1 for excess mortality for all ages.
- We also included the log-transformed, age-standardised SEV scalar for IHD as a fixed effect, country-level covariate on prevalence and LDI (I$ per capita) as a fixed effect, country-level covariate on excess mortality, forcing an inverse relationship LDI.

Table 4c. Covariates. Summary of covariates used in the Angina DisMod-MR meta-regression model

Covariate	Parameter	Beta	Exponentiated beta

Log-transformed age-standardised SEV scalar: IHD	Prevalence	1.09 (1.01 to 1.18)	2.99 (2.74 to 3.27)
LDI (I$ per capita)	Excess mortality rate	-0.54 (-0.99 to -.10)	0.58 (0.37 to 0.90)

There have been no substantive changes in the modelling strategy for myocardial infarction, asymptomatic ischaemic heart disease following myocardial infarction, and angina from GBD 2017.

Ischaemic Stroke, Intracerebral Haemorrhage, and Subarachnoid Haemorrhage

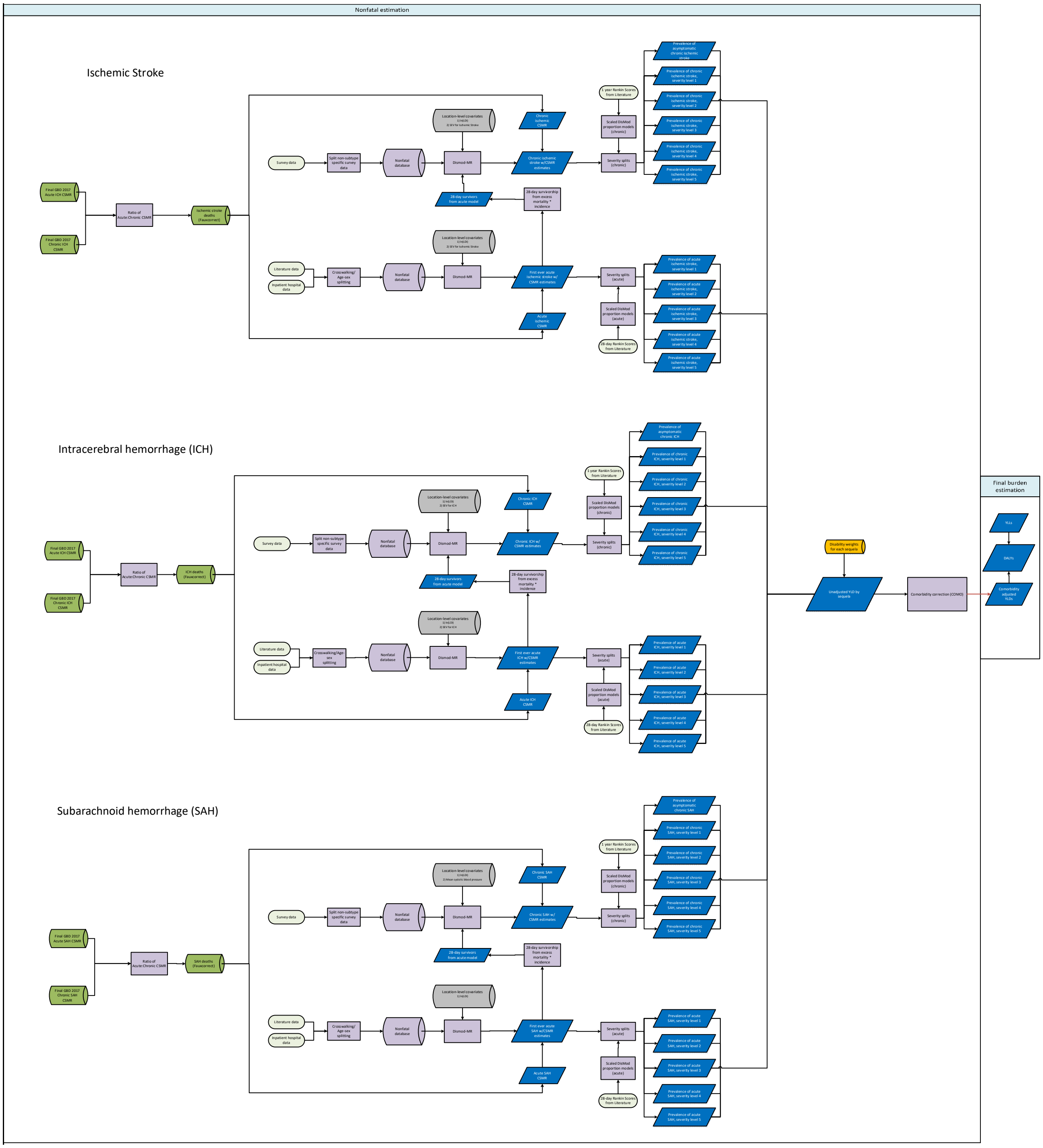

Input data and methodological summary

Case definition

Stroke was defined according to WHO criteria – rapidly developing clinical signs of focal (at times global) disturbance of cerebral function lasting more than 24 hours or leading to death with no apparent cause other than that of vascular origin (1). Data on transient ischaemic attack (TIA) were not included.

Acute stroke: Stroke cases are considered acute from the day of incidence of a first-ever stroke through day 28 following the event.

Chronic stroke: Stroke cases are considered chronic beginning 28 days following the occurrence of an event. Chronic stroke includes the sequelae of an acute stroke AND all recurrent stroke events. GBD 2015 adopts this broader definition of chronic stroke than was used in prior iterations in order to model acute strokes using only first-ever incident events.

Ischaemic stroke: an episode of neurological dysfunction caused by focal cerebral, spinal, or retinal infarction

Intracerebral haemorrhage: a focal collection of blood within the brain parenchyma or ventricular system that is not caused by trauma

Subarachnoid haemorrhage: bleeding into the subarachnoid space (the space between the arachnoid membrane and the pia mater of the brain or spinal cord)

ICD codes used for inclusion of hospital and claims data can be found elsewhere in the appendix.

Input data

Tables 1a, 1b, and 1c display source count information for non-fatal ischaemic stroke, intracerebral haemorrhage, and subarachnoid haemorrhage respectively.

Table 1a: Source counts for ischaemic stroke models.

Measure	Total sources	Countries with data
All measures	523	76
Prevalence	117	24
Incidence	332	62
Excess mortality rate	141	47
Case fatality rate	50	22

Table 1b: Source counts for intracerebral haemorrhage models.

Measure	Total sources	Countries with data
All measures	502	74
Prevalence	117	24
Incidence	322	61
Excess mortality rate	125	41
Case fatality rate	40	18

Table 1c: Source counts for subarachnoid haemorrhage models.

Measure	Total sources	Countries with data
All measures	435	63
Prevalence	117	24
Incidence	260	47
Excess mortality rate	88	28

A systematic review was not performed for GBD 2019. However, a systematic review was performed for GBD 2017. Search terms, dates of search, and databases queried follow:

1) Ischaemic stroke
    a. Google scholar: ("ischemic stroke" OR "cerebral infarction" OR "ischaemic stroke") AND (incidence OR prevalence OR mortality OR epidemiology). Reviewed first 1000 hits, sorted by relevance
    b. Global Index Medicus search: (tw:("ischemic stroke") OR tw:("cerebral infarction" OR tw:("ischaemic stroke")) AND (tw:(incidence) OR tw:(prevalence) OR tw:(mortality) OR tw:(epidemiology)) AND NOT (tw:(rats) OR tw:(mice) OR tw:(dogs) OR tw:(apes) OR tw:(monkeys)). Dates of search: 01Jan2010 – 31Aug2017
2) Intracerebral haemorrhage
    a. Google scholar: ("hemorrhagic stroke" OR "intracerebral hemorrhage" OR "haemorrhagic stroke" OR "intracerebral haemorrhage") AND (incidence OR prevalence OR mortality OR epidemiology). Reviewed first 1000 hits, sorted by relevance
    b. GIM search: (tw:("intracerebral hemorrhage") OR tw:("intracerebral haemorrhage") OR tw:("hemorrhagic stroke") OR tw:("haemorrhagic stroke")) AND (tw:(incidence) OR tw:(prevalence) OR tw:(mortality) OR tw:(epidemiology)) AND NOT (tw:(rats) OR tw:(mice) OR tw:(dogs) OR tw:(apes) OR tw:(monkeys)). Dates of search: 01Jan2010 – 31Aug2017
3) Subarachnoid haemorrhage
    a. Google scholar search: ("subarachnoid hemorrhage" OR "subarachnoid haemorrhage") AND (incidence OR prevalence OR mortality OR epidemiology). Reviewed first 1000 hits, sorted by relevance.
    b. GIM search: (tw:("subarachnoid hemorrhage") OR tw:("subarachnoid haemorrhage")) AND (tw:(incidence) OR tw:(prevalence) OR tw:(mortality) OR tw:(epidemiology)) AND

NOT (tw:(rats) OR tw:(mice) OR tw:(dogs) OR tw:(apes) OR tw:(monkeys)). Dates of search: 01Jan2010 – 31Aug2017

We included inpatient hospital data, adjusted for readmission and primary to any diagnosis using correction factors estimated from US claims data. We excluded data for locations where the data points were implausibly low (Vietnam, Philippines, India). In addition, we included unpublished stroke registry data for acute ischaemic stroke, acute intracerebral haemorrhage, and acute subarachnoid haemorrhage. We also included survey data for chronic stroke. These surveys were identified based on expert opinion and review of major survey series focused on world health that included questions regarding self-reported history of stroke. For GBD 2019, we split unspecified strokes (ICD-10 I64) into ischaemic stroke, intracerebral haemorrhage, and subarachnoid haemorrhage according to the proportions of subtype-specific coded strokes in the original data. We also split ICD-10 I62 into intracerebral haemorrhage, and subarachnoid haemorrhage using the same approach.

As with many models in GBD, the diversity of data sources available means that we needed to adjust available data to our reference case definition. We thus crosswalked incidence and excess mortality data that did not meet our reference case definitions using MR- BRT, a Bayesian meta-regression tool develop for the GBD. More information on MR-BRT can be found elsewhere in the appendix.

We adjusted data points for first and recurrent strokes combined, using data for first strokes only as reference. For ischaemic stroke and intracerebral haemorrhage, we also adjusted data points that reported all stroke subtypes combined, using as reference studies with subtype-specific information. We also adjusted data which included only persons who survived to hospital admission, using as reference data on both fatal and nonfatal strokes. In addition, we adjusted subtype-specific, inpatient clinical informatics data using subtype-specific literature estimates as a reference. These adjustments can be examined more closely in Table 2. The coefficients in Tables 2a, 2b, and 2c below can be used to calculate adjustment factors for alternative definitions. The formula for computing adjustment factors is given in equation 1 below. We also included a standardized age variable (age scaled) and a sex variable to the crosswalking procedure to adjust for the possibly of bias.

Equation 1: Calculation of adjustment factors:

$$Estimated\ Reference\ Def = invlogit(logit(Alternative\ Def) - Beta_{Alternative\ Def} - Beta_{Sex} * Sex - Beta_{Ag\ \ scaled} * Age\ Scaled)$$

No data adjustments were necessary for the chronic stroke models.

Table 2a: MR-BRT Crosswalk Adjustment Factors for Ischaemic stroke

	Data input	Measure	Reference or alternative case definition	Gamma	Beta Coefficient, Logit (95% CI)
Ischaemic stroke	First-ever, subtype-specific, fatal and nonfatal events	Incidence	Ref	---	---
Ischaemic stroke	Hospital data	Incidence	Alt	0.97	-0.26

					(-2.22 to 1.70)
Ischaemic stroke	Any stroke	Incidence	Alt		0.02 (-1.94 to 1.98)
Ischaemic stroke	Acute first-ever stroke	Incidence	Alt		0.22 (-1.67 to 2.12)
Ischaemic stroke	Inpatient clinical informatics	Incidence	Alt		0.70 (-1.26 to 2.66)
Ischaemic stroke	Sex (male)	Incidence	Alt		0.07 (-1.82 to 1.96)
Ischaemic stroke	Age scaled	Incidence	Alt		0.28 (-1.61 to 2.17)

Table 2b: MR-BRT Crosswalk Adjustment Factors for Intracerebral Haemorrhage

	Data input	Measure	Reference or alternative case definition	Gamma	Beta Coefficient, Logit (95% CI)
Intracerebral Haemorrhage	First-ever, subtype-specific, fatal and nonfatal events	Incidence	Ref	---	---
Intracerebral Haemorrhage	Hospital data	Incidence	Alt	0.50	0.04 (-0.93 to 1.02)
Intracerebral Haemorrhage	Any stroke	Incidence	Alt		1.78 (0.80 to 2.76)
Intracerebral Haemorrhage	Acute first-ever stroke	Incidence	Alt		0.15 (-0.83 to 1.13)
Intracerebral Haemorrhage	Inpatient clinical informatics	Incidence	Alt		1.40 (0.41 to 2.38)
Intracerebral Haemorrhage	Age scaled	Incidence	Alt		0.09 (-0.88 to 1.07)
Intracerebral Haemorrhage	Sex (male)	Incidence	Alt		0.10 (-0.88 to 1.06)

Table 2c: MR-BRT Crosswalk Adjustment Factors for Subarachnoid Haemorrhage

	Data input	Measure	Reference or alternative case definition	Gamma	Beta Coefficient, Logit (95% CI)
Subarachnoid Haemorrhage	First-ever, subtype-specific, fatal and nonfatal events	Incidence	Ref	---	---
Subarachnoid Haemorrhage	Aneurysmal subarachnoid haemorrhage only	Incidence	Alt	0.76	-0.79 (-2.28 to 0.70)
Subarachnoid Haemorrhage	Age scaled	Incidence	Alt		-0.11 (-1.59 to 1.38)
Subarachnoid Haemorrhage	Sex (male)	Incidence	Alt		-0.07 (-1.56 to 1.42)

Severity split inputs

The table below illustrates the severity level, lay description, and disability weights for GBD 2019. In previous iterations of GBD, severity splits for stroke were based on the standard approach described elsewhere (3). For GBD 2016, we undertook a review to identify epidemiologic literature which reported the degree of disability at 28 days (for acute stroke) or one year (for chronic stroke) using the modified Rankin scale (mRS) and the Mini-Mental State Examination (MMSE) or the Montreal Cognitive Assessment (MoCA). The mRS assesses functional capabilities, while the MMSE and MoCA tests provide evaluations of cognitive functioning. We then mapped these measures to the existing GBD categories as indicated below. This approach allowed us to include location-specific information and can be updated as more data on functional or cognitive status become available.

Acute stroke severity splits

Table 3a. Severity distribution, details on the severity levels for Acute Stroke in GBD 2019 and the associated disability weight (DW) with that severity.

Severity level	Lay description	Modified Rankin score	Cognitive status	DW (95% CI)
Stroke, mild	Has some difficulty in moving around and some weakness in one hand, but is able to walk without help.	1	N/A	0.019 (0.01–0.032)

Stroke, moderate	Has some difficulty in moving around, and in using the hands for lifting and holding things, dressing, and grooming.	2, 3	MoCA>=24 or MMSE>=26	0.07 (0.046–0.099)
Stroke, moderate plus cognition problems	Has some difficulty in moving around, in using the hands for lifting and holding things, dressing and grooming, and in speaking. The person is often forgetful and confused.	2, 3	MoCA<24 or MMSE<26	0.316 (0.206–0.437)
Stroke, severe	Is confined to bed or a wheelchair, has difficulty speaking, and depends on others for feeding, toileting, and dressing.	4, 5	MoCA>=24 or MMSE>=26	0.552 (0.377–0.707)
Stroke, severe plus cognition problems	Is confined to bed or a wheelchair, depends on others for feeding, toileting, and dressing, and has difficulty speaking, thinking clearly, and remembering things.		MoCA<24 or MMSE<26	0.588 (0.411–0.744)

Chronic stroke severity splits

Table 3b. Severity distribution, details on the severity levels for Chronic Stroke in GBD 2019 and the associated disability weight (DW) with that severity.

Severity level	Lay description	Modified Rankin score	Cognitive status	DW (95% CI)
Stroke, asymptomatic		0	N/A	N/A
Stroke, long-term consequences, mild	Has some difficulty in moving around and some weakness in one hand, but is able to walk without help.	1	N/A	0.019 (0.01–0.032)
Stroke, long-term consequences, moderate	Has some difficulty in moving around, and in using the hands for lifting and holding things, dressing, and grooming.	2, 3	MoCA>=24 or MMSE>=26	0.07 (0.046–0.099)

Stroke, long-term consequences, moderate plus cognition problems	Has some difficulty in moving around, in using the hands for lifting and holding things, dressing and grooming, and in speaking. The person is often forgetful and confused.	2, 3	MoCA<24 or MMSE<26	0.316 (0.206–0.437)
Stroke, long-term consequences, severe	Is confined to bed or a wheelchair, has difficulty speaking, and depends on others for feeding, toileting, and dressing.	4, 5	MoCA>=24 or MMSE>=26	0.552 (0.377–0.707)
Stroke, long-term consequences, severe plus cognition problems	Is confined to bed or a wheelchair, depends on others for feeding, toileting, and dressing, and has difficulty speaking, thinking clearly, and remembering things.	4, 5	MoCA<24 or MMSE<26	0.588 (0.411–0.744)

Table 4: Data input counts for the estimation process for the custom severity splits.

	**Acute proportion**	**Chronic proportion**
**Site-years (total)**	9	16
**Number of countries with data**	6	13
**Number of GBD regions with data (out of 21 regions)**	6	7
**Number of GBD super-regions with data (out of 7 super-regions)**	4	5

We used DisMod-MR, a Bayesian meta-regression tool, to model the six severity levels, with an independent proportion model for each. Reports which grouped mRS scores differently than our mapping (eg, 0-2) were adjusted in DisMod by estimating the association between these alternate groupings and our preferred mappings. These statistical associations were used to adjust data points to the referent category as necessary. The six models were scaled such that the sum of the proportions for all levels equaled 1.

Modelling strategy
The general approach employed for all of the components of the stroke modelling process is detailed in the table below.

- Data points were adjusted from alternative to reference case definitions using estimates from statistical models generated by MR-BRT (discussed elsewhere in the appendix) for the acute models. Coefficients for these crosswalks can be found in Table 2a, 2b, and 2c.

- The GBD summary exposure values (SEV), which are the relative risk-weighted prevalence of exposure, were included as covariates for the ischaemic stroke or intracerebral haemorrhage models as appropriate, and a covariate for country income was used as a country-level covariate for both models (4). Subarachnoid haemorrhage did not included an SEV covariate, but did include a covariate for country income for excess mortality. Coefficients for these covariates can be found in Table 5a, 5b, 5c for fixed effects located below.

- We used the ratio of acute:chronic cause-specific mortality estimated by the final GBD 2017 dismod model estimates to divide GBD 2019 stroke deaths into acute and chronic stroke deaths, using the global average for the proportion of acute:chronic stroke mortality. The acute and chronic models were then run using the same incidence, prevalence, and case fatality data as well as the custom cause-specific mortality rates as input data.

- We ran the first-ever acute subtype-specific models with CSMR as derived from FauxCorrect and epidemiological data as described above using Dismod-MR.

- We then calculated the rate of surviving until 28 days after an acute event for all three subtypes using the modelled estimates of excess mortality and incidence from the acute stroke models.

- Twenty-eight-day survivorship data was uploaded into the chronic subtype-specific with CSMR models. These chronic models also use CSMR as derived from FauxCorrect and epidemiological data as described above. Models were evaluated based on expert opinion, comparison with previous iterations, and model fit.

Table 5a, 5b, 5c below indicate the covariates used by cause in the estimation process, as well as the beta and exponentiated beta values.

Table 5a: Coefficients for covariates used in the acute and chronic ischemic stroke DisMod-MR models

Model	Variable name	Measure	beta	Exponentiated beta
First-ever acute ischaemic stroke with CSMR	Log-transformed age-standardised SEV scalar: Ischaemic stroke	Incidence	0.90<br>(0.85 to 0.95)	2.46<br>(2.34 to 2.58)
First-ever acute ischaemic stroke with CSMR	Healthcare access and quality index	Excess mortality rate	-0.035<br>(-0.035 to -0.035)	0.97<br>(0.97 to 0.97)

Chronic ischaemic stroke with CSMR	Log-transformed SEV scalar: Ischaemic stroke	Prevalence	0.85 (0.78 to 0.92)	2.34 (2.18 to 2.51)
Chronic ischaemic stroke with CSMR	LDI (I$ per capita)	Excess mortality rate	-0.41 (-0.46 to -0.36)	0.67 (0.63 to 0.70)

Table 5b: Coefficients for covariates used in the acute and chronic intracerebral haemorrhage DisMod-MR models

Model	Variable name	Measure	beta	Exponentiated beta
First-ever acute intracerebral haemorrhage with CSMR	Log-transformed SEV scalar: Intracerebral Haemorrhage	Incidence	0.76 (0.75 to 0.77)	2.13 (2.12 to 2.15)
First-ever acute intracerebral haemorrhage with CSMR	Healthcare access and quality index	Excess mortality rate	-0.07 (-0.07 to -0.069)	0.93 (0.93 to 0.93)
Chronic intracerebral haemorrhage with CSMR	Log-transformed SEV scalar: Intracerebral haemorrhage	Prevalence	0.75 (0.75 to 0.76)	2.12 (2.12 to 2.14)
Chronic intracerebral haemorrhage with CSMR	LDI (I$ per capita)	Excess mortality rate	-0.5 (-0.5 to -0.5)	0.61 (0.61 to 0.61)

Table 5c: Coefficients for covariates used in the acute and chronic subarachnoid DisMod-MR models

Model	Variable name	Measure	beta	Exponentiated beta
First-ever acute subarachnoid haemorrhage with CSMR	LDI (I$ per capita)	Excess mortality rate	-0.3 (-0.49 to -0.11)	0.74 (0.61 to 0.90)

Congenital birth defects

Overview and Cause List

We have estimated the prevalence and associated disability of the following categories of congenital birth defects (those in bold are GBD causes):

1. **Neural tube defects**
    a. Anencephaly
    b. Encephalocele
    c. Spina bifida
2. **Congenital heart defects**
    a. Single ventricle and single ventricle pathway defects

    b. Complex congenital heart defects excluding single ventricle and single ventricle pathway defects
    c. Malformations of great vessels, congenital valvular heart disease and patent ductus arteriosis
    d. Ventricular septal defect and atrial septal defect
    e. Other congenital cardiovascular anomalies
3. **Orofacial clefts: Cleft lip and cleft palate**
4. **Total chromosomal congenital birth defects**
    a. **Down Syndrome**
    b. **Turner Syndrome**
    c. **Klinefelter Syndrome**
    d. **Other chromosomal abnormalities, genetic syndromes, and micro-deletions**
        i. Edwards Syndrome and Patau Syndrome
        ii. Other chromosomal abnormalities, genetic syndromes, and micro-deletions
5. **Congenital anomalies of the urogenital system**
    a. Congenital urinary anomalies
    b. Congenital genital anomalies
6. **Congenital anomalies of the digestive system**
    a. Congenital diaphragmatic hernia
    b. Congenital malformations of the abdominal wall
    c. Congenital atresia and/or stenosis of the gastrointestinal tract
    d. Other congenital malformations of the gastrointestinal tract
7. **Musculoskeletal congenital anomalies**
    a. Polydactyly and syndactyly
    b. Limb reduction defects
    c. Other musculoskeletal congenital anomalies
8. **Other congenital anomalies: all birth defects (excluding minor anomalies) not contained in the other categories.**

This appendix will first describe the input data sources and aspects of the modelling strategy that are common to all sub-types of congenital anomalies. We will then provide a description of the case definitions, ICD-10 codes, and health states associated with each of the component congenital causes, as well as the specific modelling strategies employed in each congenital cause, including the model settings, study-level and country-level covariates, and other modelling decisions made.

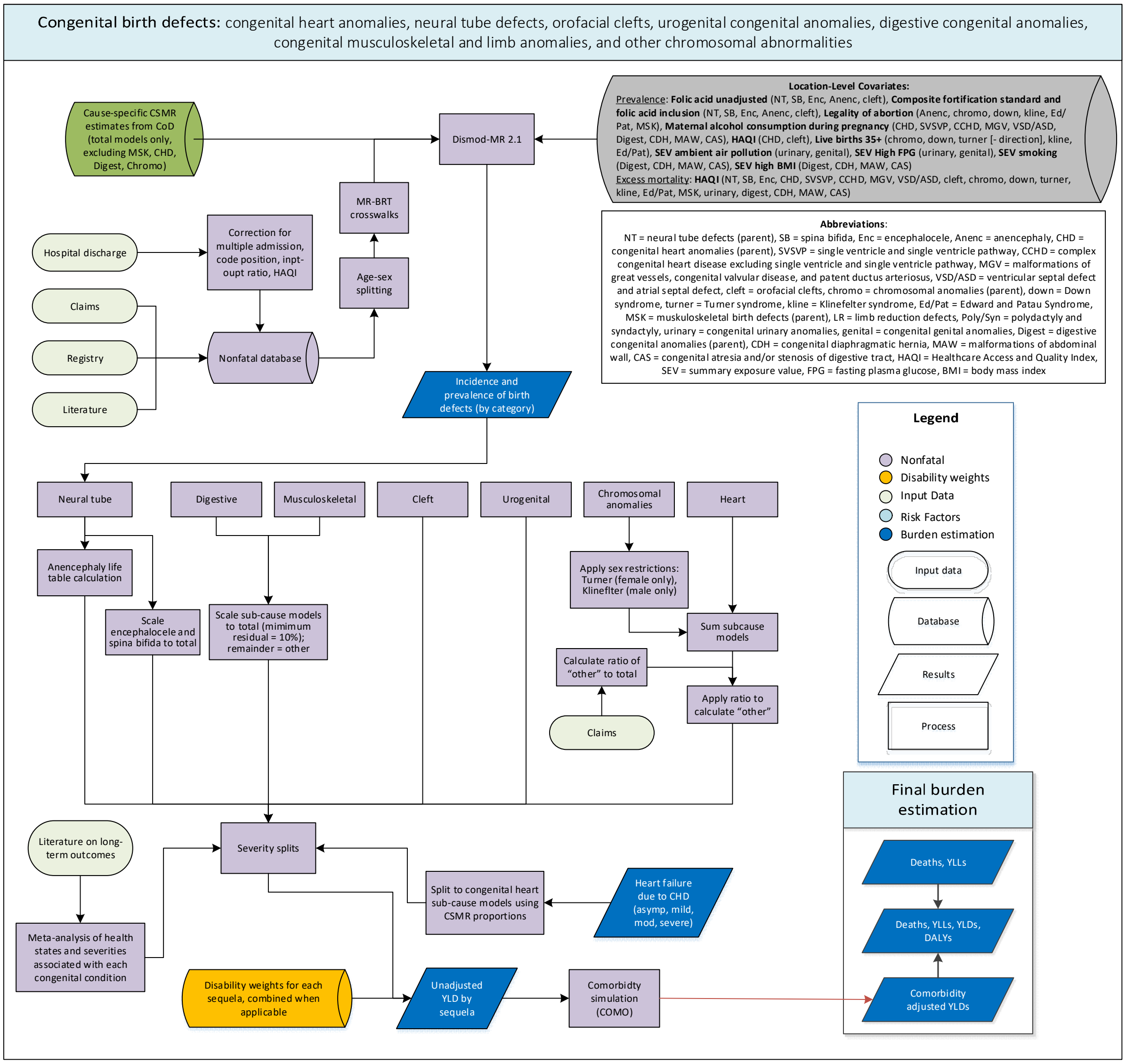

Case Definition

The GBD case definition of congenital anomalies includes any condition present at birth that is a result of abnormalities of embryonic development, excluding those that are directly the result of infections or substance abuse (e.g. fetal alcohol syndrome, congenital syphilis) modeled elsewhere in GBD and excludes minor anomalies as they are defined by EUROCAT.

Input Data

Several types of data sources are used in the estimation of congenital anomalies: literature prevalence, with-condition mortality and excess mortality data, birth prevalence and neonatal with-condition mortality data from a number of international birth defects registries and surveillance systems, inpatient hospital and Marketscan claims data prepared internally by the GBD research team, and cause-specific mortality estimates produced by the causes of death analysis.

First, We extracted data from a number of international birth defects registries. The International Clearinghouse for Birth Defects Surveillance and Research (ICBDSR) reports birth prevalence from a number of international member registries. The World Atlas Report also published birth prevalence estimates from these international registries prior to the publication of ICBDSR reports. The European Surveillance of Congenital Anomalies (EUROCAT) reports the birth prevalence of anomalies for a variety of locations in Western Europe as reported by participating member registries. China's Maternal and Child Health Surveillance survey (MCHS) reports birth prevalence and early neonatal mortality data for all subnational locations of China. The National Birth Defects Prevention Network (NBDPN) reports birth prevalence estimates as compiled by a number of subnational registries within the United States. The Birth Defects Registry of India (BDRI) reports congenital anomalies from participating hospitals within India.

Second, we used ionpatient hospital and claims data (from USA, Taiwan, and Singapore) for all congenital anomalies causes and sub-cause models. These data were prepared centrally by the clinical informatics research team and is described in detail in the Clinical Informatics section of this appendix. Four rounds of data bias correction were employed in the processing of clinical data. This included 1) adjustment for readmission, 2) correction of primary diagnoses to all diagnoses, 3) adjustment for inpatient-to-outpatient ratio, and 4) adjustment based on Healthcare Access and Quality Index (HAQI). Of note, in GBD 2017 we used congenital birth defects data only using the first two corrections, but changed in GBD 2019 to use clinical data that had all four corrections applied. This change was factilitated by improvements in analysis of corrections by the clinical informatics team and was a change made across GBD. Of note, we also changed the mapping of club foot and hip dysplasia in GBD 2019. Previously they were mapped to "limb reduction defects," but in preparatioin for disaggregated models (which is planned for the next time they are estimated in GBD), they are now included only in the total for musculoskeletal birth defects.

Third, we included data from a systematic review of the available literature for all types of congenital birth defects that was completed in GBD 2015 by constructing search strings designed to capture information on the prevalence, associated mortality and long-term health outcomes associated with each sub-category of congenital anomalies. All results were screened – first abstracts, then full-text screenings – to ensure the availability of required information and the representativeness of the reported population, and the exclusion of duplicate data also reported as part of the birth registry data inputs.

Table 1: Data inputs for modeling prevalence of congenital causes

Cause	Total Sources	Countries with Data
Congenital birth defects (all measures)	2065	104
Prevalence	1875	97
With condition mortality rate	160	41
Proportion	52	27

Other	7	5
Neural tube defects (all measures)	1677	88
Prevalence	1663	88
With condition mortality rate	10	6
Proportion	8	3
Congenital heart anomalies (all measures)	1717	93
Prevalence	1623	88
With condition mortality rate	98	28
Other	7	5
Orofacial clefts (all measures)	1619	87
Prevalence	1616	87
With condition mortality rate	5	2
Down syndrome (all measures)	1661	75
Prevalence	1626	73
With condition mortality rate	23	17
Proportion	21	21
Turner syndrome (all measures)	777	46
Prevalence	773	46
With condition mortality rate	2	2
Proportion	3	1
Klinefelter syndrome (all measures)	769	43
Prevalence	766	43
Proportion	3	1
Other chromosomal abnormalities (all measures)	1327	67
Prevalence	1304	65
Proportion	23	22
Congenital musculoskeletal and limb anomalies (all measures)	1639	87
Prevalence	1635	87
With condition mortality rate	2	1

Proportion	2	1
Urogenital congenital anomalies (all measures)	1709	93
Prevalence	1697	92
With condition mortality rate	7	4
Proportion	7	5
Digestive congenital anomalies (all measures)	1758	80
Prevalence	1716	76
With condition mortality rate	45	16
Proportion	7	6

Data processing

Age-sex splitting

Any data that was not sex-specific or did not fit entirely within GBD age-groups were age- and sex-split to fit these groups prior to modeling using empirical age- and sex-patterns derived from previous DisMod-MR 2.1 models of the same condition. This is a change from GBD 2017 when age- and sex-splitting of data was not completed prior to modeling and had a substantial effect on the magnitude of estimates in those causes for which cause-specific mortality rate (CSMR) data was used in modeling. This is described further below.

Crosswalks in MR-BRT

A number of the input data sources used for the estimation of congenital birth defects are known to have biases leading to under-reporting or over-reporting relative to the true prevalence of congenital anomalies among live births and all subsequent age groups. We used Meta Regression – Bayesian, Regularised Trimmed (MR-BRT) to develop statistical models that were used to adjust non-reference data. The alternate definitions that were crosswalked are described below. The specifics of each MR-BRT crosswalk are shown in the corresponding cause-specific sections.

Live/Stillbirths: Where necessary, we used a crosswalk to adjust for the inclusion of stillbirths in the reported birth prevalence estimates in literature and registry data sources, as stillbirths are not included in our case definition of prevalence among live births. Each of these crosswalks used a spline on log-transformed neonatal mortality rate.

Exclusion of chromosomal conditions: Some sources report birth defects on in isolation (i.e. excluding any persons who have a coexisting genetic or chromosomal disorder). Our reference definition is the inclusion of chromosomal diagnoses. No splines were used in these crosswalks.

Registry to total: For a subset of congenital causes, particularly the congenital heart defects, we noted substantial differences in the lists of case definitions being reported to the various congenital registries. Across all types of congenital heart defects, the National Birth Defects Prevention Network (NBDPN) had the most complete list of reported case definitions – i.e. the highest case ascertainment – and was considered the gold standard among all birth registry data sources. We used registry-specific crosswalks

to adjust all other birth defects registries to match the case ascertainment seen in the NBDPN. No splines were used in these crosswalks.

Determining outliers and data thresholds

Underreporting of congenital birth defects is common and can vary by source, location, year, sex, and age. In order to have an empirical, systematic approach to outliering of data, we adapted the non-zero floor approach used by the GBD cause-specific mortatlity analysis. After all age-sex splitting and crosswalking was complete, the first step was to calculate median absolute deviation (MAD) for the age group of birth, where registry and literature data were combined with all clinical data for the early neonatal age group (0 to 6 days). The thresholds chosen were -0.5 MAD and +3 MAD with any data outside of these bounds being identified as outliers. This was determined based on the right skewed distribution observed in most of the congenital data and the expert prior that underreporting is far more prevalent than overreporting – and therefore the bias is asymmetric. In any case where the lower MAD bound was negative, we used a threshold of 0.

For most models, we calculated the MADs using only the EUROCAT data, which we found to be the most reliable source for prevalence of congenital disorders. Exceptions were neural tube defects (all data sources), Urinary birth defects (EUROCAT and USA claims data), musculoskeletal defects (only USA claims data), and chromosomal anomalies, which differed by condition given the high volume of zeroes in the data. For Down Syndrome, we used all data. For Edward Syndrome and Patau Syndrome, we used all non-zero EUROCAT data. For Turner and Klinefelter syndrome, we used EUROCAT data and logged mean absolute deviation and exponentiated this to determine bounds for these data.

To evaluate data for older age groups, we employed two approaches. First, we outliered data from any location-year-source that was outliered for the first stage MAD algorithm. Second, using all clinical and literature data, we developed a model with fixed effects by age to estimated implied MAD bounds for each non-zero age group and again applied the same thresholds of -0.5 MAD and +3 MAD.

Modelling Strategy

Overview

All available input data was utilized in a series DisMod-MR 2.1 models in order to estimate the prevalence of each category of congenital anomalies across the full life course for each location/age/sex combination. Incidence was set to 0 for all congenital models, as congenital conditions occur at the time of birth and by GBD case definition, congenital cases do not occur after birth. Remission was allowed only in the models of a select subset of causes for which surgical intervention or spontaneous remission can completely eliminate the disability due to that congenital condition. Cause-specific priors and slope priors were used to guide biologically plausible DisMod-MR 2.1 estimates of excess mortality and remission where applicable.

For most of the congenital birth defects causes, we ran DisMod-MR 2.1 models of all defects combined (termed “parent” models). This allowed us to use data on all anomalies within each cause as well as to leverage cause-specific mortality rate (CSMR) results from the GBD cause of death (COD) analysis. When CSMR data is used as an input, DisMod-MR 2.1 pairs each CSMR datum with a mathing prevalence data point by age, sex, location, and year. After matching, CSMR is divided by prevalence to calculate an implied excess-mortality rate (EMR) datum. All EMR data is then used in driving the model. Of note, EMR

data is not calculated when prevalence data is of broader than GBD age groups or is for both sexes combined.

We used CSMR as input to all of the models except congenital heart disease, chromosomal anomalies, digestive anomalies, musculoskeletal birth defects, and urogenital congenital anomalies. For congenital heart defects, the reason is that excess mortalilty would be underestimated in older ages if CSMR results are used because despite continuing higher rates of mortality through adolescence and adulthood, many of these deaths are not coded as being due to congenital heart disease. Similarly, musculoskeletal and gastrointestinal anomalies estimates for CSMR in older children, adolescents, and adults are much lower than would be suggested by cohort and cross-sectional studies of survival as few of these deaths are coded as being due to the congenital birth defect present. Finally, for urogenital congenital anomalies, in addition to our modeling urinary and genital anomalies separately, the mechanism of death in older ages will typically be via development of chronic kidney disease and these deaths are classified in GBD as being due to chronic kidney disease due to other conditions. Details are in each cause-specific section below.

Location-level Covariates

Location-level covariates were used in each of the congenital DisMod-MR 2.1 models based on published information about the risk factors for these birth defects. Folic acid availability was used as a covariate on prevalence for all neural tube defects models and a subset of the congenital musculoskeletal anomalies models. A folic acid fortification covariate was used in the neural tube defects and cleft models, which was modelled based on data from the Global Fortification Data Exchange. The legality of abortion was used as a covariate on prevalence for conditions in which prenatal diagnosis is commonly available and the prognosis is severe enough to cause high rate of termination of pregnancy following prenatal diagnosis: these include all chromosomal conditions and a subset of the congenital heart defects. Maternal consumption of alcohol during pregnancy, as a proportion of all pregnancies, was used as a covariate on prevalence for all congenital heart defects. The proportion of live births by mothers age 35+ was used as a covariate on all chromosomal models. Across many of the congenital models, the Health Access and Quality Index (HAQI) covariate was used to guide the global pattern of with-condition mortality and excess mortality, as was the natural log of the lag-distributed income per capita (LN-LDI). For most of the severe congenital conditions, the mortality associated with the condition is highly dependent on access to adequate surgical interventions and other medical care during the first hours, weeks, and years of life.

Post-model processing

For those causes with a parent model (neural tube defects, we then squeezed the sum of the specific sub-cause prevalence estimates to these total prevalence estimates in order to ensure internal consistency of our cause-level and sub-cause estimates. *The prevalence of other* heart, musculoskeletal, and gastrointestinal anomalies *was derived by reducing the total envelope model for each cause by its sub-causes to derive the difference that was attributable to other anomalies in that category.*

Assigning health states and sequelae for long-term outcomes

To determine the distribution of health outcomes associated with the congenital causes, we performed a review of available literature on the long-term health outcomes of survivors in cohorts born with each type of congenital malformation. For conditions requiring surgical intervention shortly after birth to

ensure survival, the health states included in the disability weight calculations correspond to the post-surgery outcomes reported in cohorts of individuals born with these life-threatening congenital conditions. Where data was available from multiple cohorts, we pooled these cohorts together to calculate the proportion of individuals with each health state. Where data on the joint distribution of the long-term health outcomes was not available, we assumed independence of each long-term health outcome. Combined disability weights were calculated for all necessary combinations of existing disability weights.

Congenital heart anomalies

Summary and associated health states

There are many distinct types of congenital heart anomalies with a range of anatomical patterns, severities, and requirements for medical treatment. For the purpose of estimating nonfatal outcomes, in GBD 2017 congenital heart anomalies were split into five-sub categories based on both the anatomical characteristics and the treatment requirements of each condition.

1. Single ventricle and single ventricle pathway defects
2. Complex congenital heart defects excluding single ventricle and single ventricle pathway defects
3. Malformations of great vessels, congenital valvular heart disease and patent ductus arteriosis
4. Ventricular septal defect and atrial septal defect
5. Other congenital cardiovascular anomalies

We also began development of a model of total congenital heart anomalies, but this was not used in scaling the subcauses for GBD 2019. Instead, we used claims data to calculate a ratio of other-to-total and this was applied to the sum of the other four subcauses for each location, age group, sex, and year.

Every case of congenital heart defects was associated with a health state of congenital heart disease, except for a proportion of ventricular and atrial septal defects which are considered asymptomatic. All congenital heart defects cases were split into a proportion without intellectual disability and a proportion with every severity from borderline to profound intellectual disability. The proportion of congenital heart anomalies cases experiencing each severity of intellectual disability were calculated using available literature sources on the prevalence and severity of intellectual disability in congenital heart defect populations[1,2,3]. The proportion of VSD/ASD cases attributed to the asymptomatic category was derived from literature sources on the long-term outcomes of patients diagnosed with septal defects at birth[4,5,6]. GBD estimates of congenital heart failure were assigned to the congenital heart defect categories according to the proportion of total congenital heart cause-specific mortality assigned to each category of congenital heart defects.

Total congenital heart anomalies

Crosswalks

The MR-BRT crosswalk results are shown below.

Table 1: MR-BRT crosswalk betas for alternate definitions (reference = livebirths including those with chromosomal anomalies)

Crosswalk	Beta	Standard Error
Excluding chromosomal diagnoses adjustment	-0.096	0.006

Figure 1: MR-BRT crosswalk of alternate definition (livebirths and stillbirths included) with spline on log-transformed neonatal mortality rate.

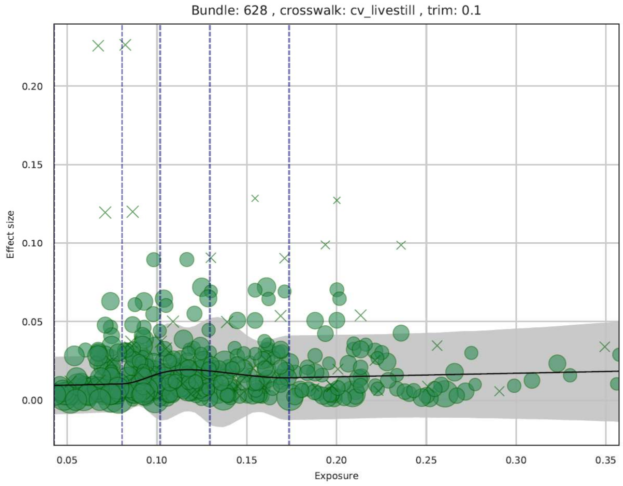

Modelling strategy

In the DisMod-MR 2.1 model of total congenital heart anomalies, random effects on prevalence were limited to +- 0.5 in order to limit geographic variation in the estimates of birth prevalence. The minimum excess mortality rate for the neonatal age range was set to 5.0. The smoothness on excess mortality rate was increased to Xi=5.0 in order to allow high excess mortality in the neonatal age groups and lower excess mortality rates in older ages.

Table 2. Location-level covariate effects

Covariate Name	Measure	Beta value	Exponentiated value
Maternal alcohol consumption during pregnancy (proportion)	Prevalence	0.17046 (0.01367 - 0.37530)	1.19 (1.01 - 1.46)
Healthcare access and quality index	Prevalence	0.00087 (0.00007 - 0.00202)	1.00 (1.00 - 1.00)
Healthcare access and quality index	EMR	-0.15320 (-0.29760 - -0.00718)	0.86 (0.74 - 0.99)

Single ventricle and single ventricle pathway defects

Case definition

Single ventricle and single ventricle pathway defects include tricuspid atresia, hypoplastic left heart syndrome, mitral valve atresia, single left ventricle, double outlet right ventricle, and pulmonary atresia; the corresponding ICD-10 codes are Q20.1, Q20.2, Q20.4, Q22.4, Q22.6 and Q23.4. Each of the single ventricle and single ventricle pathway conditions requires surgical intervention shortly after birth to ensure infant survival.

Crosswalks

The MR-BRT crosswalk results are shown below.

Table 1: MR-BRT crosswalk betas for alternate definitions (reference = livebirths including those with chromosomal anomalies)

Crosswalk	Beta	Standard Error
Excluding chromosomal diagnoses adjustment	-0.066	0.023
Adjustment for registry specific case definitions (World Atlas)	-0.752	0.035
Adjustment for registry specific case definitions (ICBDMS)	-0.751	0.036
Adjustment for registry specific case definitions (Congenital Malformations Worldwide)	-0.754	0.036

Figure 1: MR-BRT crosswalk of alternate definition (livebirths and stillbirths included) with spline on log-transformed neonatal mortality rate.

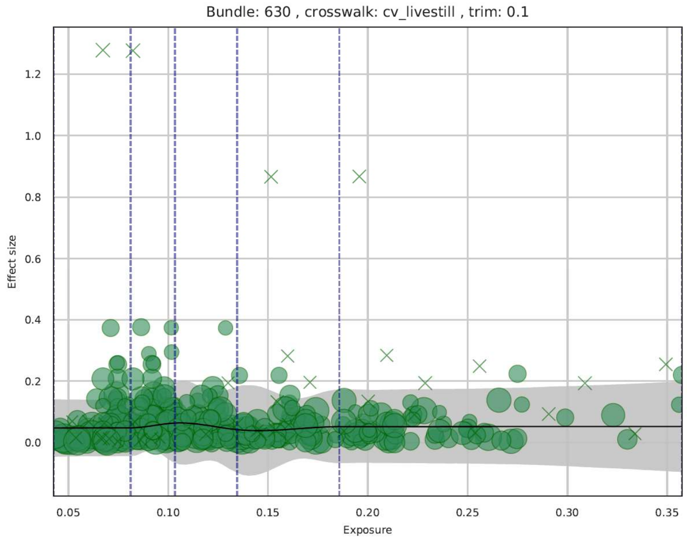

Modelling strategy

In the DisMod-MR 2.1 model of single ventricle and single ventricle pathway heart defects, random effects on prevalence were limited to +- 0.5 in order to limit the estimated geographic variation in birth prevalence. A minimum excess mortality rate of 8 was set for the early neonatal period in order to capture the high mortality risk, based on expert priors and a review available literature on the mortality risk among infants born with single ventricle and single ventricle pathway heart defects. The smoothness on excess mortality rate was set to 5.0 in order to fit steep changes in the excess mortality rate during the first weeks of life, as the risk of death due to these congenital heart anomalies is greatest shortly after birth and diminishes over the life course.

Table 2. Location-level covariate effects

Covariate Name	Measure	Beta value	Exponentiated value

Maternal alcohol consumption during pregnancy (proportion)	Prevalence	0.23369 (0.02821 - 0.45690)	1.26 (1.03 - 1.58)
HAQI	EMR	-0.04909 (-0.09541 - -0.00156)	0.95 (0.91 - 1.00)

Complex congenital heart defects excluding single ventricle and single ventricle pathway defects

Case definition

Complex congenital heart defects excluding single ventricle and single ventricle pathway defects include common arterial trunk, common truncus, discordant ventriculoaterial connection, transposition of great vessels, atrioventricular septal defect, endocardial cushion defect, Tetralogy of fallot, aortopulmonary septal defect, pulmonary valve atresia, congenital stenosis of aortic valve, and total anomalous pulmonary venous connection. This category of severe congenital heart defects includes ICD-10 codes Q20.0; Q20.3; Q21.2; Q21.3; Q21.4; Q22.0; Q23.0 and Q26.2.

Crosswalks

The MR-BRT crosswalk results are shown below.

Table 1: MR-BRT crosswalk betas for alternate definitions (reference = livebirths including those with chromosomal anomalies)

Crosswalk	Beta	Standard Error
Excluding chromosomal diagnoses adjustment	-0.223	0.014
Adjustment for registry specific case definitions (World Atlas)	-0.626	0.015
Adjustment for registry specific case definitions (ICBDMS)	-0.625	0.016

Figure 1: MR-BRT crosswalk of alternate definition (livebirths and stillbirths included) with spline on log-transformed neonatal mortality rate.

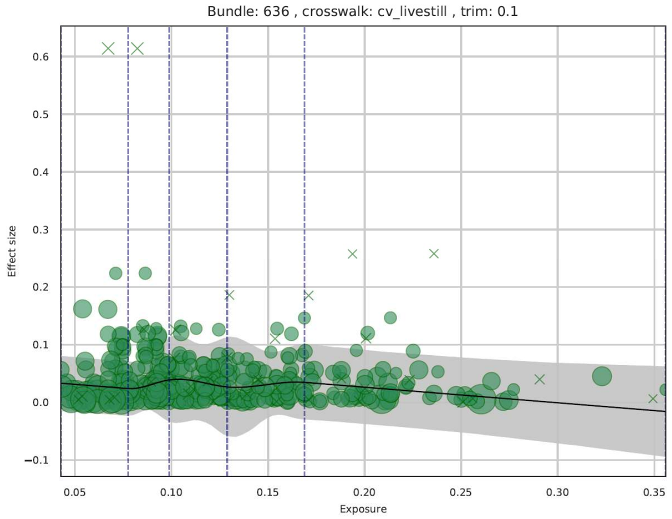

Modelling strategy

In the DisMod-MR 2.1 model of congenital heart defects excluding single ventricle and single ventricle pathway defects, random effects on prevalence were limited to +- 0.5. A minimum excess mortality rate of 1.0 for the early neonatal period was enforced in order to capture the high risk of mortality associated with these conditions, and a decreasing slope prior on excess mortality rate was applied for all ages. The smoothness on excess mortality rate was set to Xi = 3.0 in order to allow the model to fit steep changes in the mortality rate of these conditions in the neonatal age period.

Table 2. Location-level covariate effects

Covariate Name	Measure	Beta value	Exponentiated value
Maternal alcohol consumption during pregnancy (proportion)	Prevalence	0.18871 (0.01810 - 0.43850)	1.21 (1.02 - 1.55)
HAQI	EMR	-0.05045 (-0.09804 - -0.00408)	0.95 (0.91 - 1.00)

Malformations of great vessels, congenital valvular heart disease and patent ductus arteriosis

Case definition

Malformations of great vessels, congenital valvular heart disease and patent ductus arteriosis. The malformations of vessels and valves in this sub-cause category include Ebstein's anomaly, congenital pulmonary valve stenosis, pulmonary valve insufficiency, other malformations of the pulmonary valve, malformations of the tricuspid valve, tricuspid atresia or stenosis, insufficiency of the aortic valve, mitral stenosis or insufficiency, and other malformations of aortic and mitral valves. Patent ductus arteriosis cases are only included among infants of >37 weeks gestational age, as premature infants often have minor patent ductus arteriosis that closes shortly after birth. The ICD-10 codes corresponding to the critical malformations of great vessels category include Q22.1, Q22.2, Q22.3, Q22.5, Q22.8, Q22.9, Q23.1, Q23.2, Q23.3, Q23.8, Q23, Q25.1, Q25.2, Q25.3, Q25.4, Q25.5, and Q25.0. The majority of these conditions require medical attention shortly within the first few weeks of life.

Crosswalks

The MR-BRT crosswalk results are shown below.

Table 1: MR-BRT crosswalk betas for alternate definitions (reference = livebirths including those with chromosomal anomalies)

Crosswalk	Beta	Standard Error
Excluding chromosomal diagnoses adjustment	-0.094	0.01
Adjustment for registry specific case definitions (World Atlas)	-1.079	0.021
Adjustment for registry specific case definitions (ICBDMS)	-1.08	0.021

Figure 1: MR-BRT crosswalk of alternate definition (livebirths and stilbirths included) with spline on log-transformed neonatal mortality rate.

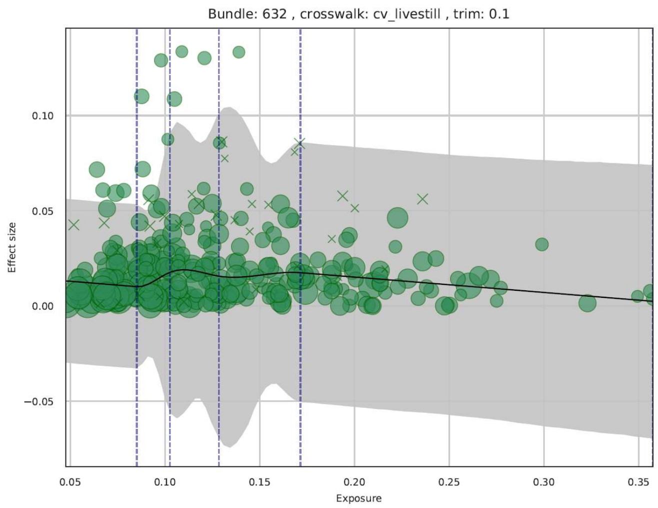

Modelling strategy

In the DisMod-MR 2.1 model of critical malformations of great vessels, congenital valvular heart disease and patent ductus arteriosis, random effects on prevalence were limited to +- 0.5. A minimum excess mortality rate of 1.0 was set for the early neonatal period in order to capture the high mortality risk associated with these conditions. The smoothness on excess mortality was increased to Xi = 3.0 in order to fit steep changes in the mortality associated with these conditions during and after the neonatal period, as the risk of death due to congenital heart anomalies is highest shortly after birth.

Table 2. Location-level covariate effects

Covariate Name	Measure	Beta value	Exponentiated value
Maternal alcohol consumption during pregnancy (proportion)	Prevalence	0.23645 (0.03553 - 0.45853)	1.27 (1.04 - 1.58)
HAQI	EMR	-0.04919 (-0.09692 - 0.00000)	0.95 (0.91 - 1.00)

Ventricular septal defects and atrial septal defects

Case definition

Ventricular septal defects and atrial septal defects, includes holes in the walls separating the chambers of the heart. Many of these septal defects close spontaneously, while other require surgical care. The ICD-10 codes corresponding to ventricular septal defect and atrial septal defect are Q21.0 and Q21.1, respectively.

Crosswalks

The MR-BRT crosswalk results are shown below.

Table 1: MR-BRT crosswalk betas for alternate definitions (reference = livebirths including those with chromosomal anomalies)

Crosswalk	Beta	Standard Error
Excluding chromosomal diagnoses adjustment	-0.082	0.006

Figure 1: MR-BRT crosswalk of alternate definition (livebirths and stillbirths included) with spline on log-transformed neonatal mortality rate.

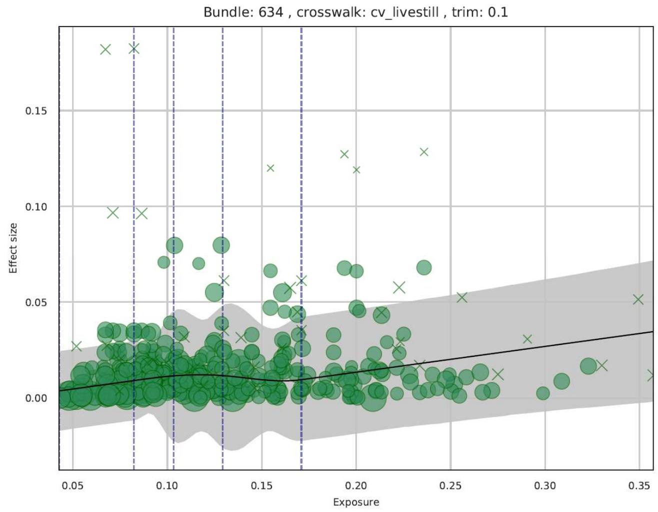

Modelling strategy

In the DisMod-MR 2.1 model of ventricular septal defects and atrial septal defects (VSD/ASD), remission was set to zero for all ages. Cases of septal defects that spontaneously close over time were considered as part of the asymptomatic proportion of VSD/ASD rather than remitted cases. Random effects on prevalence were limited to +- 0.3 in order to limit the random geographic variation in the estimated birth prevalence. No minimum excess mortality rate was set in this model, as VSD/ASD cases are not associated with excess mortality rates as high as the other subtypes of congenital heart defects. The smoothness on excess mortality rate was set to Xi=3.0, and a decreasing slope prior was set on remission for all ages, with remission set to 0 past age 10.

Table 2. Location-level covariate effects

Covariate Name	Measure	Beta value	Exponentiated value
Maternal alcohol consumption during pregnancy (proportion)	Prevalence	0.06761 (0.00336 - 0.17970)	1.07 (1.00 - 1.2)
HAQI	EMR	-0.14973 (-0.29700 - -0.00485)	0.86 (0.74 - 1.0)

Other congenital cardiovascular birth defects

Case definition

The fifth and final sub-cause category of congenital heart defects is other congenital cardiovascular anomalies, which correspond to ICD-10 codes Q27, Q27.1, Q27.2, Q27.3, Q27.30, Q27.31, Q27.32, Q27.33, Q27.34, Q27.39, Q27.4, Q27.8, Q27.9, Q28, Q28.0, Q28.1, Q28.2, Q28.3, Q28.8 and Q28.9.

Modeling strategy

Other congenital cardiovascular anomalies are modeled by applying the ratio of other congenital heart anomalies to total congenital heart anomalies as it is reflected in Marketscan data (a trusted data source), to the sum of the sub-causes of congenital cardiovascular anomalies. The result is prevalence of other congenital cardiovascular anomalies by age/year/sex/location. Specifically, we use claims data to calculate the proportion of cases that are due to the other causes. To do that, we sum the cases for the specified congenital subcauses and the other category subcauses. We divide the number of other subcause cases by the total number of cases to obtain the proportion. In order to have a valid proportion, we only use datapoints for which we have the combination of age, sex, location and year for all subcauses. We then calculate the prevalence of other: p_other = (p_sum_subcauses / 1-prop_other) - p_sub_subcauses.

References

1. Riehle-Colarusso T, Autry A, Razzaghi H, Boyle CA, Mahle WT, Van Naarden Braun K, Correa A. Congenital Heart Defects and Receipt of Special Education Services. Pediatrics. 2015; 136(3): 496– 504.

2. Menting ME, Cuypers JAAE, Opić P, Utens EMWJ, Witsenburg M, van den Bosch AE, van Domburg RT, Meijboom FJ, Boersma E, Bogers AJJC, Roos-Hesselink JW. The unnatural history of the ventricular septal defect: outcome up to 40 years after surgical closure. J Am Coll Cardiol. 2015; 65(18): 1941– 51.

3. Gaynor JW, Stopp C, Wypij D, Andropoulos DB, Atallah J, Atz AM, Beca J, Donofrio MT, Duncan K, Ghanayem NS, Goldberg CS, Hövels-Gürich H, Ichida F, Jacobs JP, Justo R, Latal B, Li JS, Mahle WT, McQuillen PS, Menon SC, Pemberton VL, Pike NA, Pizarro C, Shekerdemian LS, Synnes A, Williams I, Bellinger DC, Newburger JW, International Cardiac Collaborative on Neurodevelopment (ICCON) Investigators. Neurodevelopmental outcomes after cardiac surgery in infancy. Pediatrics. 2015; 135(5): 816–25.

4. Wren C, O'Sullivan JJ. Survival with congenital heart disease and need for follow up in adult life. Heart. 2001; 85(4): 438–43.

5. Gabriel HM, Heger M, Innerhofer P, Zehetgruber M, Mundigler G, Wimmer M, Maurer G, Baumgartner H. Long-term outcome of patients with ventricular septal defect considered not to require surgical closure during childhood. J Am Coll Cardiol. 2002; 39(6): 1066–71.

6. Neumayer U, Stone S, Somerville J. Small ventricular septal defects in adults. Eur Heart J. 1998; 19(10): 1573–82.

Rheumatic Heart Disease

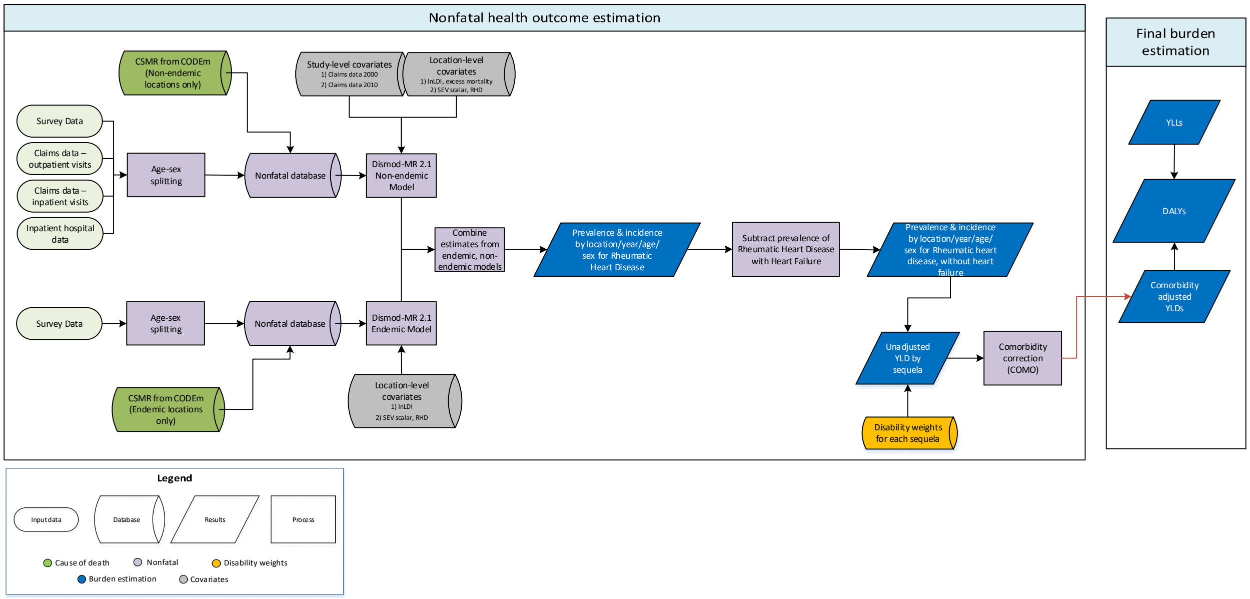

Input data and methodological appendix

Case definition

Rheumatic heart disease (RHD) was defined as a clinical diagnosis by a physician with or without confirmation using echocardiography. This case definition for echocardiographic confirmation of RHD follows the World Heart Federation criteria for echocardiographic diagnosis of rheumatic heart disease (1).

**Criterion**	**Definition**
1. Echocardiography	Prevalent rheumatic heart disease based on echocardiographic assessment and clinical confirmation
2. Clinical diagnosis	Prevalent rheumatic heart disease based on physician diagnosis

ICD codes for data included from hospital records can be found elsewhere in the appendix.

Input data

Model inputs

Table 1: Source counts for rheumatic heart disease

Measure	Total sources	Countries with data
All measures	198	58

Prevalence	198	58

Table 1 shows the source counts for rheumatic heart disease. We did not perform a systematic review for GBD 2017. A systematic review was performed for GBD 2013 and updated for GBD 2015. The GBD 2015 search information encompassed the following:

- Search terms: ('rheumatic heart disease' AND epidemiology[MeSH Subheading]) OR ('acute rheumatic fever' AND epidemiology[MeSH Subheading]) OR ('rheumatic fever' AND epidemiology[MeSH Subheading]) OR (RHD AND epidemiology[MeSH Subheading]) OR ('valvular heart disease' AND epidemiology[MeSH Subheading]) OR (((streptococcus OR streptococci) AND heart) AND epidemiology[MeSH Subheading]) OR (heart AND valve AND disease AND epidemiology[MeSH Subheading]) OR ('mitral valve stenosis' AND epidemiology[MeSH Subheading]) OR (('rheumatic heart disease' OR 'rheumatic fever') AND prevalence) OR (('rheumatic heart disease' OR 'rheumatic fever') AND incidence) OR (('rheumatic heart disease' OR 'rheumatic fever') AND ('standardized mortality ratio' OR SMR)) OR ('rheumatic heart disease' OR 'rheumatic fever' AND 'case fatality')
- Dates included in search: 1/1/2013 – 3/16/2015
- Number of initial hits: 2,045
- Number of sources included: 17

These differed from the GBD 2013 search terms:

- (hasabstract[text] AND Humans[Mesh] AND middle age[MeSH])) OR 21) AND ((rheumatic heart disease/epidemiology[Mesh] OR rheumatic heart disease/mortality[Mesh]) AND (prevalence[Title/Abstract] OR incidence[Title/Abstract]) AND ("2010"[Date - Publication] : "3000"[Date - Publication]) AND (hasabstract[text] AND Humans[Mesh] AND middle age[MeSH]))

We did not include any non-literature-based data types other than the hospital and claims data described elsewhere. Prevalence from hospital and claims data sources were included only for the non-endemic country model. Inpatient data were adjusted for multiple visits, non-primary diagnoses, and inpatient to outpatient utilisation ratios. This methodology is detailed elsewhere in the appendix.

Severity splits and disability weights

**Severity level**	**Lay description**	**DW (95% CI)**
Rheumatic heart disease, not including heart failure	Has a chronic disease that requires medication every day and causes some worry but minimal interference with daily activities.	0.049 (0.031–0.072)

Modelling strategy

For GBD 2019 estimation, we ran two models using DisMod-MR – one for non-endemic countries and one for endemic countries. For GBD 2016, we identified locations as endemic if the estimated death rate due to RHD was greater than 0.15 per 100,000 in the 5 to 9 age group, or if that location had an SDI less

than 0.6. Beginning in GBD 2017, we identified locations as endemic if the estimated death rate due to RHD was greater than 0.15 per 100,000 in the 10 to 14 age group, or if that location had an SDI less than 0.6. This change in age group was made based on feedback from RHD expert reviewers due to concerns that the death rate in 5 to 9 age group would not capture endemicity in locations where RHD is common only in later age groups. Each location estimated as part of GBD 2019 is listed below as either "Endemic" or "Non-endemic".

Remission

In GBD 2016, we assumed that there was no remission from RHD. Beginning in GBD 2017, we estimated remission in both the endemic and non-endemic DisMod models. This decision was based on two studies[2,3] that observed remission among confirmed RHD cases. We used the equation below to convert reported proportion of remitted individuals in each study to a remission rate, defined as the number of remitted cases divided by the total person-years of disease:

$$remission\ rate = \frac{\log(1 - proportion\ remitted)}{years\ of\ followup}$$

Where *proportion remitted* is the reported proportion of all individuals with RHD at baseline who ended up remitting, and *years of followup* is the mean follow-up time in the study. The relevant values for the two papers and the calculated remission rates are listed in the table below.

Study	Remitted proportion	Mean follow-up time	Calculated remission rate
Beaton et al[2]	0.3	2.4 years	0.14 cases per person-year
Engelman et al[3]	0.1	7.5 years	0.014 cases per person-year

In order to acknowledge the uncertainty in these calculated remission rates and to allow DisMod flexibility in estimating remission, we input 0.2 as the upper bound for remission the remission prior and 0.00 as the lower bound for remission the remission prior. Because the two studies used to estimate remission were done only in children, we applied these remission priors to only those younger than age 20, and setting a remission prior of zero for adults older than age 20.

DisMod models

Non-endemic model: We included hospital data, claims data, and limited literature data on prevalence. We also included CSMR from our mortality estimates of RHD for non-endemic locations only. A prior of no remission was set, and excess mortality was capped at 0.1 for all ages. Coefficients for selected covariates are listed in the table below.

Endemic model: We included prevalence data from surveys published in the literature. As with the high-income model, we included CSMR from our mortality estimates of RHD for endemic locations only. A prior of no remission was set for all ages, and excess mortality was capped at 0.07, the highest observed mean excess mortality rate data point observed in this model. We also set priors of 0 on incidence for ages 0 to 1 and 50 to 100 to account for patterns of incidence in endemic countries. We used lnLDI as a fixed-effect country-level covariate on prevalence and excess mortality, enforcing an inverse relationship for both. The log-transformed, age-standardised SEV scalar was also used as a fixed-effect country-level covariate on prevalence.

We combined estimates from the endemic and non-endemic models, selecting estimates for the locations identified as non-endemic from the non-endemic model and estimates for the locations identified as endemic from the endemic model. Estimates of heart failure due to RHD were then

subtracted from the estimates for RHD, giving the overall prevalence of RHD without heart failure. A description of the modelling strategy for heart failure due to RHD can be found in the heart failure appendix. We evaluated models based on comparing estimates with input data as well as estimates from previous rounds of GBD.

The table below shows the country covariates, parameters, betas, and exponentiated betas:

Covariate	Parameter	Beta	Exponentiated beta
Endemic model			
Log-transformed age-standardised SEV scalar: RHD	Prevalence	0.95 (0.76 to 1.17)	2.57 (2.15 to 3.23)
LDI (I$ per capita)	Excess mortality rate	-0.3 (-0.49 to -0.11)	0.74 (0.61 to 0.90)
Non-endemic model			
Log-transformed age-standardised SEV scalar: RHD	Prevalence	0.76 (0.75 to 0.78)	2.14 (2.12 to 2.18)
LDI (I$ per capita)	Excess mortality rate	-0.94 (-0.96 to -0.93)	0.39 (0.38 to 0.40)

Endemic locations: Aceh, Acre, Addis Ababa, Afar, Afghanistan, Alagoas, Albania, Alborz, Algeria, Amapá, Amazonas, American Samoa, Amhara, Andean Latin America, Andhra Pradesh, Andhra Pradesh, Rural, Andhra Pradesh, Urban, Angola, Anhui, Antigua and Barbuda, Ardebil, Argentina, Armenia, Arunachal Pradesh, Arunachal Pradesh, Rural, Assam, Assam, Rural, Assam, Urban, Azerbaijan, Bahia, Bangladesh, Barbados, Baringo, Belize, Bengkulu, Benin, Benishangul-Gumuz, Bhutan, Bihar, Bihar, Rural, Bihar, Urban, Bolivia, Bomet, Botswana, Brazil, Bungoma, Burkina Faso, Burundi, Busia, Cambodia, Cameroon, Cape Verde, Caribbean, Ceará, Central African Republic, Central Asia, Central Europe, Eastern Europe, and Central Asia, Central Kalimantan, Central Sub-Saharan Africa, Chad, Chahar Mahaal and Bakhtiari, Chhattisgarh, Chhattisgarh, Rural, Chhattisgarh, Urban, Chiapas, China, Chongqing, Comoros, Congo, Costa Rica, Cote d'Ivoire, Cuba, Delhi, Delhi, Rural, Delhi, Urban, Democratic Republic of the Congo, Dire Dawa, Distrito Federal, Djibouti, Dominica, Dominican Republic, East Asia, East Azarbayejan, East Nusa Tenggara, Eastern Cape, Eastern Sub-Saharan Africa, Ecuador, Egypt, El Salvador, Elgeyo-Marakwet, Embu, Equatorial Guinea, Eritrea, Espírito Santo, Ethiopia, Fars, Federated States of Micronesia, Fiji, Free State, Gabon, Gambella, Gansu, Garissa, Gauteng, Georgia, Ghana, Gilan, Global, Goa, Goa, Rural, Goa, Urban, Goiás, Golestan, Gorontalo, Grenada, Guam, Guangxi, Guatemala, Guerrero, Guinea, Guinea-Bissau, Guizhou, Gujarat, Gujarat, Rural, Gujarat, Urban, Guyana, Hainan, Haiti, Hamadan, Harari, Haryana, Haryana, Rural, Haryana, Urban, Hebei, Heilongjiang, Henan, Hidalgo, Himachal Pradesh, Himachal Pradesh, Rural, Himachal Pradesh, Urban, HomaBay, Honduras, Hormozgan, Hubei, Hunan, Ilam, India, Inner Mongolia, Iran, Iraq, Isfahan, Isiolo, Jamaica, Jammu and Kashmir, Jammu and Kashmir, Rural, Jammu and Kashmir, Urban, Jharkhand, Jharkhand, Rural, Jharkhand, Urban, Jiangxi, Jilin, Kajiado, Kakamega, Karnataka, Karnataka, Rural, Karnataka, Urban, Kenya, Kerala, Kerala, Rural, Kerala, Urban, Kericho, Kerman, Kermanshah, Khorasan-e-Razavi, Khuzestan, Kiambu, Kilifi, Kiribati, Kirinyaga, Kisii, Kisumu, Kitui, Kohgiluyeh and Boyer-Ahmad, Kurdistan, Kwale, KwaZulu-Natal, Kyrgyzstan, Laikipia, Lamu, Laos, Latin America and Caribbean, Lesotho, Liaoning, Liberia, Libya, Limpopo, Lorestan, Machakos, Madagascar, Madhya Pradesh, Madhya

Pradesh, Rural, Madhya Pradesh, Urban, Maharashtra, Maharashtra, Rural, Maharashtra, Urban, Makueni, Malawi, Malaysia, Maldives, Mali, Maluku, Mandera, Manipur, Manipur, Rural, Manipur, Urban, Maranhão, Markazi, Marsabit, Marshall Islands, Mato Grosso, Mato Grosso do Sul, Mauritania, Mauritius, Mazandaran, Meghalaya, Meghalaya, Rural, Meghalaya, Urban, Meru, Mexico City, Michoacán de Ocampo, Migori, Minas Gerais, Mizoram, Rural, Mombasa, Mongolia, Morocco, Mozambique, Mpumalanga, Murang'a, Myanmar, Nagaland, Nagaland, Rural, Nairobi, Nakuru, Namibia, Nandi, Narok, Nepal, Nicaragua, Niger, Nigeria, Ningxia, North Africa and Middle East, North Africa and Middle East, North Khorasan, North Korea, North Maluku, North-West, Northern Cape, Northern Mariana Islands, Nyamira, Nyandarua, Nyeri, Oaxaca, Oceania, Odisha, Odisha, Rural, Odisha, Urban, Oromia, Pakistan, Palestine, Panama, Papua, Papua New Guinea, Pará, Paraguay, Paraíba, Paraná, Pernambuco, Peru, Philippines, Piaui, Puebla, Punjab, Punjab, Rural, Punjab, Urban, Qazvin, Qinghai, Rajasthan, Rajasthan, Rural, Rajasthan, Urban, Republic of Tuva, Riau Islands, Rio de Janeiro, Rio Grande do Norte, Rio Grande do Sul, Rondônia, Roraima, Rwanda, Saint Lucia, Saint Vincent and the Grenadines, Samburu, Samoa, Santa Catarina, São Paulo, Sao Tome and Principe, Semnan, Senegal, Sergipe, Seychelles, Shaanxi, Shandong, Shanxi, Siaya, Sichuan, Sierra Leone, Sikkim, Sikkim, Rural, Sikkim, Urban, Sistan and Baluchistan, Solomon Islands, Somali, Somalia, South Africa, South Asia, South Asia, South Kalimantan, South Khorasan, South Sudan, Southeast Asia, Southeast Asia, East Asia, and Oceania, Southeast Sulawesi, Southern Nations, Nationalities, and Peoples, Southern Sub-Saharan Africa, Sub-Saharan Africa, Sudan, Suriname, Swaziland, Syria, TaitaTaveta, Tajikistan, Tamil Nadu, Tamil Nadu, Rural, Tamil Nadu, Urban, TanaRiver, Tanzania, Tehran, Telangana, Telangana, Rural, Telangana, Urban, Thailand, TharakaNithi, The Bahamas, The Gambia, Tianjin, Tibet, Tigray, Timor-Leste, Tocantins, Togo, Tonga, TransNzoia, Trinidad and Tobago, Tripura, Tripura, Rural, Tripura, Urban, Tropical Latin America, Turkana, Turkmenistan, Tyumen oblast without autonomous areas, UasinGishu, Uganda, Union Territories other than Delhi, Union Territories other than Delhi, Rural, Union Territories other than Delhi, Urban, United Arab Emirates, Uttar Pradesh, Uttar Pradesh, Rural, Uttar Pradesh, Urban, Uttarakhand, Uttarakhand, Rural, Uttarakhand, Urban, Uzbekistan, Vanuatu, Veracruz de Ignacio de la Llave, Vihiga, Wajir, West Azarbayejan, West Bengal, West Bengal, Rural, West Bengal, Urban, West Kalimantan, West Nusa Tenggara, West Papua, West Sulawesi, West Sumatra, Western Cape, Western Sub-Saharan Africa, WestPokot, Xinjiang, Yemen, Yunnan, Zambia, Zanjan, Zimbabwe

Non-endemic locations: Aguascalientes, Aichi, Akershus, Akita, Alabama, Alaska, Altai kray, Amur oblast, Andorra, Aomori, Arizona, Arkansas, Arkhangelsk oblast without Nenets autonomous district, Arunachal Pradesh, Urban, Astrakhan oblast, Aust-Agder, Australasia, Australia, Austria, Bahrain, Baja California, Baja California Sur, Bali, Bangka-Belitung Islands, Banten, Barking and Dagenham, Barnet, Barnsley, Bath and North East Somerset, Bedford, Beijing, Belarus, Belgium, Belgorod oblast, Bermuda, Bexley, Birmingham, Blackburn with Darwen, Blackpool, Bolton, Bosnia and Herzegovina, Bournemouth, Bracknell Forest, Bradford, Brent, Brighton and Hove, Bristol, City of, Bromley, Brunei, Bryansk oblast, Buckinghamshire, Bulgaria, Bury, Bushehr, Buskerud, Calderdale, California, Cambridgeshire, Camden, Campeche, Canada, Central Bedfordshire, Central Europe, Central Java, Central Latin America, Central Sulawesi, Chechen Republic, Chelyabinsk oblast, Cheshire East, Cheshire West and Chester, Chiba, Chihuahua, Chile, Chukchi autonomous area, Chuvash Republic, Coahuila, Colima, Colombia, Colorado, Connecticut, Cornwall, County Durham, Coventry, Croatia, Croydon, Cumbria, Cyprus, Czech Republic, Darlington, Delaware, Denmark, Derby, Derbyshire, Devon, District of Columbia, Doncaster, Dorset, Dudley, Durango, Ealing, East Java, East Kalimantan, East Midlands, East of England, East Riding of

Yorkshire, East Sussex, Eastern Europe, Ehime, Enfield, England, Essex, Estonia, Finland, Finnmark, Florida, France, Fujian, Fukui, Fukuoka, Fukushima, Gateshead, Georgia, Germany, Gifu, Gloucestershire, Greater London, Greece, Greenland, Greenwich, Guanajuato, Guangdong, Gunma, Hackney, Halton, Hammersmith and Fulham, Hampshire, Haringey, Harrow, Hartlepool, Havering, Hawaii, Hedmark, Herefordshire, County of, Hertfordshire, High-income, High-income Asia Pacific, High-income North America, Hillingdon, Hiroshima, Hokkaidō, Hong Kong Special Administrative Region of China, Hordaland, Hounslow, Hungary, Hyōgo, Ibaraki, Iceland, Idaho, Illinois, Indiana, Indonesia, Iowa, Ireland, Irkutsk oblast, Ishikawa, Isle of Wight, Islington, Israel, Italy, Ivanovo oblast, Iwate, Jakarta, Jalisco, Jambi, Japan, Jewish autonomous oblast, Jiangsu, Jordan, Kabardian-Balkar Republic, Kagawa, Kagoshima, Kaliningrad oblast, Kaluga oblast, Kamchatka kray, Kanagawa, Kansas, Karachaev-Chercassian Republic, Kazakhstan, Kemerovo oblast, Kensington and Chelsea, Kent, Kentucky, Khabarovsk kray, Khanty-Mansi autonomous area, Kingston upon Hull, City of, Kingston upon Thames, Kirklees, Kirov oblast, Knowsley, Kōchi, Komi Republic, Kostroma oblast, Krasnodar kray, Krasnoyarsk kray, Kumamoto, Kurgan oblast, Kursk oblast, Kuwait, Kyōto, Lambeth, Lampung, Lancashire, Latvia, Lebanon, Leeds, Leicester, Leicestershire, Leningrad oblast, Lewisham, Lincolnshire, Lipetzk oblast, Lithuania, Liverpool, Louisiana, Luton, Luxembourg, Macao Special Administrative Region of China, Macedonia, Magadan oblast, Maine, Malta, Manchester, Maryland, Massachusetts, Medway, Merton, Mexico, México, Michigan, Middlesbrough, Mie, Milton Keynes, Minnesota, Mississippi, Missouri, Miyagi, Miyazaki, Mizoram, Mizoram, Urban, Moldova, Montana, Montenegro, Møre og Romsdal, Morelos, Moscow City, Moscow oblast, Murmansk oblast, Nagaland, Urban, Nagano, Nagasaki, Nara, Nayarit, Nebraska, Nenets autonomous district, Netherlands, Nevada, New Hampshire, New Jersey, New Mexico, New York, New Zealand, New Zealand Maori population, New Zealand non-Maori population, Newcastle upon Tyne, Newham, Niigata, Nizhny Novgorod oblast, Nordland, Norfolk, North Carolina, North Dakota, North East England, North East Lincolnshire, North Kalimantan, North Lincolnshire, North Somerset, North Sulawesi, North Sumatra, North Tyneside, North West England, North Yorkshire, Northamptonshire, Northern Ireland, Northumberland, Norway, Nottingham, Nottinghamshire, Novgorod oblast, Novosibirsk oblast, Nuevo León, Ohio, Ōita, Okayama, Okinawa, Oklahoma, Oldham, Oman, Omsk oblast, Oppland, Oregon, Orenburg oblast, Oryol oblast, Ōsaka, Oslo, Østfold, Oxfordshire, Pennsylvania, Penza oblast, Perm kray, Peterborough, Plymouth, Poland, Poole, Portsmouth, Portugal, Primorsky kray, Pskov oblast, Puerto Rico, Qatar, Qom, Querétaro, Quintana Roo, Reading, Redbridge, Redcar and Cleveland, Republic of Adygeya, Republic of Altai, Republic of Bashkortostan, Republic of Buryatia, Republic of Crimea, Republic of Dagestan, Republic of Ingushetia, Republic of Kalmykia, Republic of Karelia, Republic of Khakasia, Republic of Mariy El, Republic of Mordovia, Republic of North Ossetia-Alania, Republic of Sakha (Yakutia), Republic of Tatarstan, Rhode Island, Riau, Richmond upon Thames, Rochdale, Rogaland, Romania, Rostov oblast, Rotherham, Russian Federation, Rutland, Ryazan oblast, Saga, Saitama, Sakhalin oblast, Salford, Samara oblast, San Luis Potosí, Sandwell, Sankt-Petersburg, Saratov oblast, Saudi Arabia, Scotland, Sefton, Serbia, Sevastopol, Shanghai, Sheffield, Shiga, Shimane, Shizuoka, Shropshire, Sinaloa, Singapore, Slough, Slovakia, Slovenia, Smolensk oblast, Sogn og Fjordane, Solihull, Somerset, Sonora, South Carolina, South Dakota, South East England, South Gloucestershire, South Korea, South Sulawesi, South Sumatra, South Tyneside, South West England, Southampton, Southend-on-Sea, Southern Latin America, Southwark, Spain, Sri Lanka, St Helens, Staffordshire, Stavropol kray, Stockholm, Stockport, Stockton-on-Tees, Stoke-on-Trent, Suffolk, Sunderland, Surrey, Sutton, Sverdlovsk oblast, Sweden, Sweden except Stockholm, Swindon, Switzerland, Tabasco, Taiwan, Tamaulipas, Tambov oblast, Tameside, Telemark, Telford and Wrekin,

Tennessee, Texas, Thurrock, Tlaxcala, Tochigi, Tokushima, Tōkyō, Tomsk oblast, Torbay, Tottori, Tower Hamlets, Toyama, Trafford, Troms, Trøndelag, Tula oblast, Tunisia, Turkey, Tver oblast, Udmurt Republic, Ukraine, Ukraine (without Crimea & Sevastopol), Ulyanovsk oblast, United Kingdom, United States, Uruguay, Utah, Venezuela, Vermont, Vest-Agder, Vestfold, Vietnam, Virgin Islands, U.S., Virginia, Vladimir oblast, Volgograd oblast, Vologda oblast, Voronezh oblast, Wakayama, Wakefield, Wales, Walsall, Waltham Forest, Wandsworth, Warrington, Warwickshire, Washington, West Berkshire, West Java, West Midlands, West Sussex, West Virginia, Western Europe, Westminster, Wigan, Wiltshire, Windsor and Maidenhead, Wirral, Wisconsin, Wokingham, Wolverhampton, Worcestershire, Wyoming, Yamagata, Yamaguchi, Yamalo-Nenets autonomous area, Yamanashi, Yaroslavl oblast, Yazd, Yogyakarta, York, Yorkshire and the Humber, Yucatán, Zabaikalsk kray, Zacatecas, Zhejiang

References

1. Reményi, B. et al. Nat. Rev. Cardiol. 9, 297–309 (2012); published online 28 February 2012
2. Beaton A, Aliku T, Dewyer A, et al. Latent Rheumatic Heart Disease: Identifying the Children at Highest Risk of Unfavorable Outcome. Circulation. 2017;136(23):2233-2244.
3. Engelman D, Wheaton GR, Mataika RL, et al. Screening-detected rheumatic heart disease can progress to severe disease. Heart Asia. 2016;8(2):67-73.

Acute Myocarditis

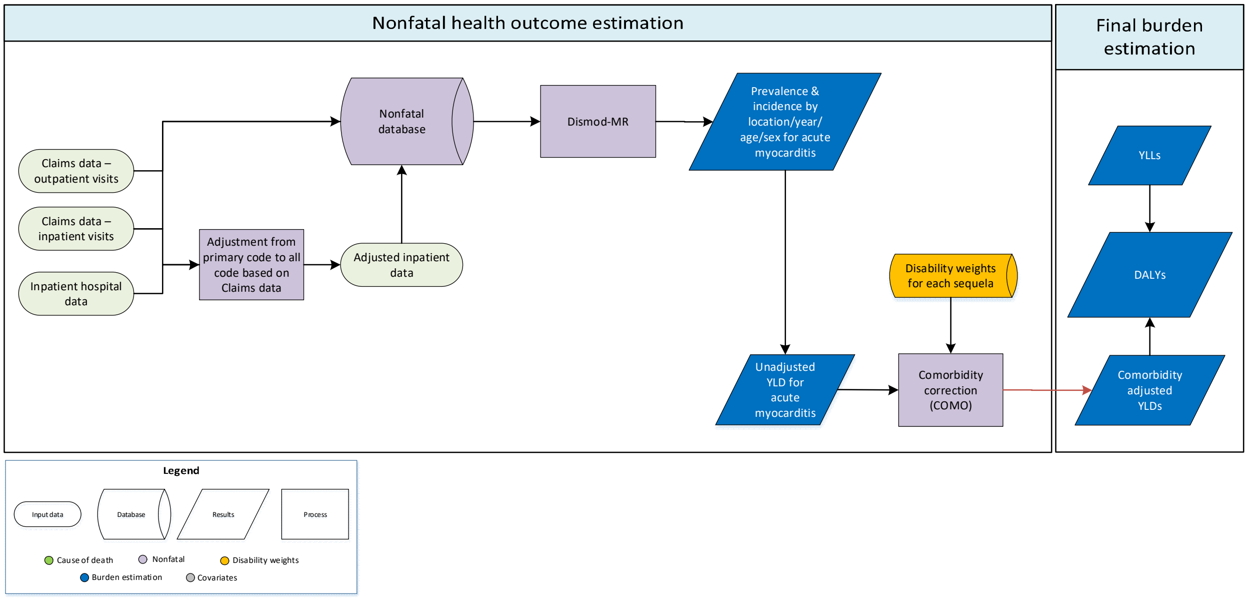

Input data and methodological summary

Case definition

Myocarditis refers to a heterogenous group of diseases with variable clinical and pathological features. Acute myocarditis was defined for GBD as the acute and time-limited symptoms of myocarditis separate from its chronic heart failure-related sequelae. Heart failure due to myocarditis is estimated separately in GBD (see methods for heart failure). Symptoms of acute myocarditis are nonspecific and include a flu-

like or gastrointestinal syndrome, followed by anginal-type chest pain, arrhythmias, syncope, or heart failure.

A list of the ICD codes included can be found in elsewhere in the appendix.

Input data

Model inputs

The preferred data sources for acute myocarditis were hospital admission data and other health facility data identifying cases of acute myocarditis. Table 1 shows the source counts for acute myocarditis.

Table 1: Source counts for acute myocarditis

Measure	Total sources	Countries with data
All measures	250	39
Incidence	250	39

A systematic review was performed for GBD 2013 and updated for GBD 2015. A systematic review was not performed for GBD 2019.

The GBD 2015 search terms included: (cardiomyopathy AND epidemiology [MeSH Subheading]) OR (myocarditis AND epidemiology [MeSH Subheading]) OR (cardiomyopathy AND (incidence OR prevalence OR “case fatality”)) OR (myocarditis AND (incidence OR prevalence OR “case fatality”))

- Dates included in search: 1/1/2013 – 3/16/2015
- Number of initial hits: 3,598
- Number of sources included: 0

The GBD 2013 search terms included: (hasabstract[text] AND Humans[Mesh] AND middle age[MeSH])) OR 21) AND ((cardiomyopathy/epidemiology[Mesh] OR cardiomyopathy/mortality[Mesh]) AND (prevalence[Title/Abstract] OR incidence[Title/Abstract]) AND ("2010"[Date - Publication] : "3000"[Date - Publication]) AND (hasabstract[text] AND Humans[Mesh] AND middle age[MeSH]))

We did not include any non-literature-based data, apart from the hospital and claims data described elsewhere. We used inpatient hospital data adjusted for readmission, primary to any diagnosis, and inpatient to outpatient utilisation based on correction factors generated using USA claims data. We excluded all outpatient data, as they were implausibly low when compared with inpatient data from the same locations and with claims data. Inpatient hospital data points that were more than two-fold higher or 0.5-fold lower than the median absolute deviation value for high-income North America, Central Europe, and Western Europe for that age-sex group were excluded.

Severity splits and disability weights

Table 2. Severity distribution, details on the severity levels for Acute Myocarditis in GBD 2019 and the associated disability weight (DW) with that severity.

Severity level	Lay description	DW (95% CI)
Acute myocarditis	Has a fever and aches, and feels weak, which causes some difficulty with daily activities.	0.051 (0.032–0.074)

Modelling strategy

For GBD 2019, we estimated acute myocarditis using a DisMod-MR Bayesian meta-regression model, setting a minimum of 3 and maximum of 5 as value priors on remission to establish an average duration of three months. We set a value prior of 0 for all ages on excess mortality. In GBD 2017, the country-level covariates used included the cardiomyopathy and myocarditis summary exposure variable (SEV) on incidence and the Healthcare Access and Quality index (HAQ Index) on excess mortality. For GBD 2019, The only country level covariate used was Healthcare Access and Quality Index (HAQ Index) on excess mortality.

Table 3 below gives the parameters, betas, and exponentiated betas for study-level and country-level covariates used in the model

Table 3. Covariates. Summary of covariates used in the Acute Myocarditis DisMod-MR meta-regression model

Study covariate	Parameter	beta	Exponentiated beta
Healthcare Access and Quality index	Excess mortality rate	-0.55 (-0.99 to -0.1)	0.58 (0.37 to 0.90)

Aside from the minor covariate change, no other substantive changes were made to the modelling approach for GBD 2017.

Atrial Fibrillation and Flutter

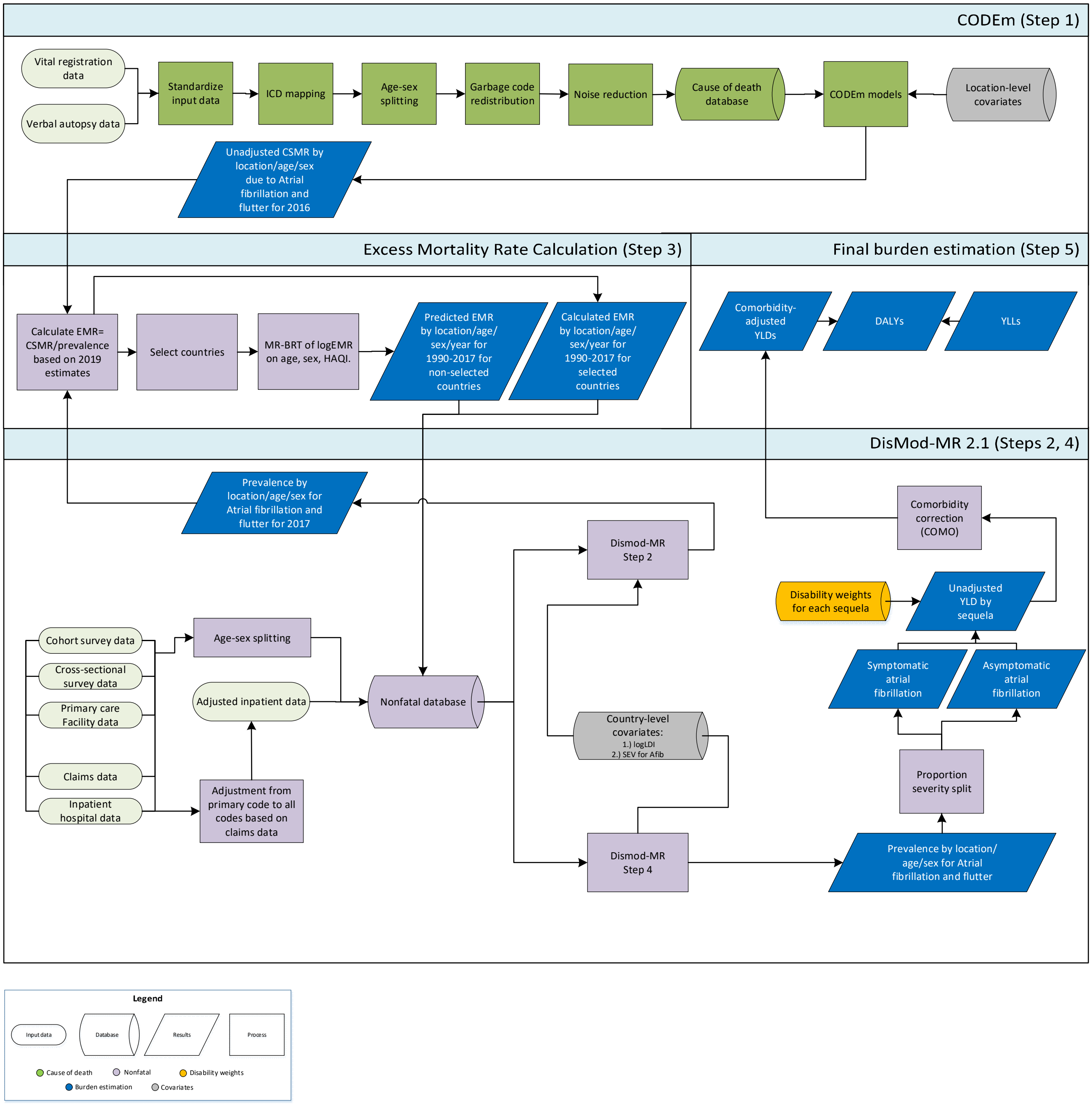

Input data and methodological summary

Case definition

Atrial fibrillation is a supraventricular arrhythmia due to disorganised depolarisation of the atrium. Atrial flutter is a macro-reentrant supraventricular arrhythmia, usually involving the cavo-tricuspid isthmus. Diagnosis requires an ECG demonstrating: 1) irregularly irregular RR intervals (in the absence of

complete AV block); 2) no distinct P waves on the surface ECG, and; 3) an atrial cycle length (when visible) that is usually variable and less than 200 milliseconds.

ICD codes used for inclusion of hospital and claims data can be found elsewhere in the appendix.

Input data

Model inputs

Table 1 shows the source counts for atrial fibrillation and flutter in GBD 2019.

Measure	Total sources	Countries with data
All measures	347	51
Prevalence	335	51
Incidence	11	8
Excess mortality rate	4	4
With-condition mortality rate	6	6

We did not perform a systematic review for GBD 2019. A systematic review was performed for GBD 2015 with the following search terms: ("atrial fibrillation" AND epidemiology[MeSH Subheading]) OR ("atrial flutter" AND epidemiology[MeSH Subheading]) OR ("atrial fibrillation" AND (prevalence OR incidence OR "case fatality")) OR ("atrial flutter" AND (prevalence OR incidence OR "case fatality")) OR ("heart atrium fibrillation" AND epidemiology[MeSH Subheading]) OR ("heart atrium fibrillation" AND (prevalence OR incidence OR "case fatality"))

The dates of the search were 1/1/2013 – 3/15/2016. There were 5,630 studies returned and, of those, 27 were extracted.

A systematic review was also performed for GBD 2013, with the search terms: (hasabstract[text] AND Humans[Mesh] AND middle age[MeSH])) OR 21) AND ((atrial fibrillation/epidemiology[Mesh] OR atrial fibrillation/mortality[Mesh]) AND (prevalence[Title/Abstract] OR incidence[Title/Abstract]) AND ("2010"[Date - Publication]: "3000"[Date - Publication]) AND (hasabstract[text] AND Humans[Mesh] AND middle age[MeSH]))

Apart from hospital and claims data points on prevalence, no non-literature-based data were included. We included hospital data corrected for readmission, primary to any diagnosis, and inpatient to outpatient utilisation ratios using adjustment factors calculated from US claims data. We excluded hospital data in certain geographies (eg, Philippines, China, India, Mexico, Botswana) where the data were implausibly low. We also excluded all outpatient administrative data as the values for all locations were implausibly low.

We adjusted claims and inpatient hospital data using literature data in which an ECG reading was used as a reference using MR-BRT crosswalking procedures. These procedures are discussed in detail

elsewhere in the appendix. Table 2 shows the adjustment factors produced by the crosswalking procedure. The crosswalking coefficients in Table 2 below can be used to calculate adjustment factors for alternative definitions. The formula for computing adjustment factors is given in equation 1 below. We also included a standardized age variable (age scaled) and a sex variable to the crosswalking procedure to adjust for the possibly of bias.

Equation 1: Calculation of adjustment factors:

$$Estimated\ Reference\ Def = invlogit(logit(Alternative\ Def) - Beta_{Alternative\ Def} - Beta_{Sex} * Sex - Beta_{Age_{scaled}} * Age\ Scaled)$$

Table 2: MR-BRT Crosswalk Adjustment Factors for Atrial Fibrillation and Flutter

Data input	Reference or alternative case definition	Gamma	Beta Coefficient, Logit (95% CI)
Literature using ECG reading	Ref	0.99	---
Claims and hospital inpatient data	Alt		-0.29(-2.33 to 1.75)
Age scaled	Alt		-0.04 (-1.98 to 1.89)
Sex (male)	Alt		-0.07 (-2.00 to 1.87)

Severity splits & disability weights

Atrial fibrillation is split into symptomatic and asymptomatic based on standard GBD proportion information. The table below includes lay descriptions and disability weights for the severity levels of atrial fibrillation:

Table 3. Severity distribution, details on the severity levels for Atrial Fibrillation and Flutter in GBD 2019 and the associated disability weight (DW) with that severity.

Severity level	Lay description	DW (95% CI)
Asymptomatic	No symptoms	N/A
Symptomatic	Has periods of rapid and irregular heartbeats and occasional fainting	0.224 (0.151–0.312)

Modelling strategy

In order to address changes in coding practices for atrial fibrillation that resulted in an implausible trend of increasing death-certificate-based mortality rates, we used a prevalence-based modelling approach that combined DisMod-MR and CODEm models to generate estimates for atrial fibrillation and flutter. This approach, first used in GBD 2015, allowed us to generate more accurate estimates, using observed prevalence and incidence rates along with modelled excess mortality rates generated from prevalence and cause-specific mortality estimates.

The modelling steps are illustrated in the above flowchart. Effect sizes for covariates included in both the DisMod-MR 2.1 and CODEm models can be found in the table below.

- In Step 1, we estimated deaths for atrial fibrillation using a standard CODEm approach.

- In Step 2, we estimated prevalence rates in DisMod-MR using data from published reports of cross-sectional and cohort surveys, as well as primary care facility data. We also used claims data covering inpatient and outpatient visits for the United States along with inpatient hospital data from 247 locations in 15 countries. For GBD 2019, inpatient hospital data were adjusted using age- and sex-specific information for: 1) readmission within one year; 2) primary diagnosis code to secondary codes; and, 3) the ratio of inpatient to outpatient visits. These clinical informatics data were then further adjusted using MR-BRT to account for misclassification compared with reference data. We set priors of no remission and capped excess mortality at 0.4 for all ages. We included the Healthcare Access and Quality (HAQ) index as a country-level, fixed-effect covariate on excess mortality and the log-transformed, age-standardised SEV scalar for atrial fibrillation and flutter as a country-level, fixed-effect covariate on prevalence.

- In Step 3, we calculated the excess mortality rate (EMR) for 2019 (defined as the cause-specific mortality rate [CSMR] estimated from CODEm divided by the prevalence rate from DisMod-MR). We then selected 17 countries based on four conditions: 1) ranking of 4 or 5 stars on the system for assessing the quality of VR data; 2) prevalence data available from the literature were included in the DisMod-MR estimation; 3) prevalence rate ≥ 0.005; and, 4) CSMR ≥ 0.00002. Using information from these countries as input data, we ran a MR-BRT model of logEMR on sex, a cubic spline of age, and HAQI. Specifics on the MR-BRT framework can be found elsewhere in the appendix. We then predicted year-, age- and sex-specific EMR using the results of this regression for all non-selected countries. Countries included in the regression were assigned their directly calculated values. These EMR data points were assigned to the time period 1990–2017 and uploaded into the non-fatal database in order to be used in modelling.

- In Step 4, we re-ran DisMod-MR using the input data described in Step 2 along with the EMR estimated in Step 3. We included Healthcare access and quality index (HAQI) as a fixed-effect, country-level covariate on excess mortality and the log-transformed, age-standardised SEV scalar for atrial fibrillation and flutter as a fixed-effect, country-level covariate on prevalence. We included a value prior of 0 for remission for all ages and set a value prior of 0 for excess mortality for ages 0-30.

The prevalence from the DisMod-MR model in Step 4 was used as the finalised output for upload to COMO and further processing into YLDs and DALYs.

Models were evaluated based on expert opinion, comparison with results from previous rounds of GBD, and model fit.

The tables below include the study covariates, parameters, betas, and exponentiated betas.

Table 4a. Covariates. Summary of covariates used in the Atrial Fibrillation and Flutter step 2 DisMod-MR meta-regression model

Covariate	Parameter	Beta	Exponentiated beta
Log-transformed age-standardised SEV scalar: A Fib	Prevalence	0.75 (0.75 to 0.76)	2.12 (2.12 to 2.13)

Healthcare Access and Quality Index	Excess mortality rate	-0.11 (-0.13 to -0.099)	0.89 (0.88 to 0.91)

Table 4b. Covariates. Summary of covariates used in the Atrial Fibrillation and Flutter step 4 DisMod-MR meta-regression model

Covariate	Parameter	Beta	Exponentiated beta
Log-transformed age-standardised SEV scalar: A Fib	Prevalence	1.15 (1.10 to 1.21)	3.17 (3.01 to 3.34)
Healthcare Access and Quality Index	Excess mortality rate	-0.017 (-0.017 to -0.017)	0.98 (0.98 to 0.98)

No substantive changes were made to the modelling strategy for GBD 2019.

Non-rheumatic valvular heart diseases: Calcific aortic valve disease, degenerative mitral valve disease, and other non-rheumatic valve disease

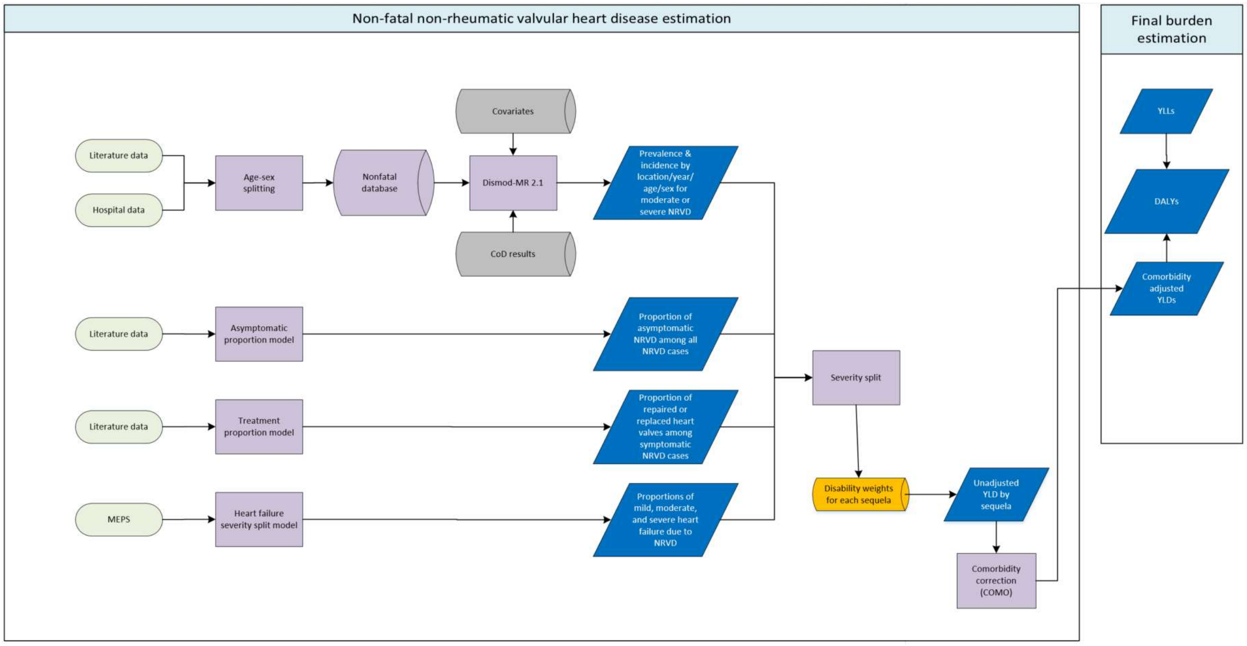

Case definitions

Calcific aortic valve disease

Calcific aortic valve disease was defined as clinical diagnosis of aortic valve stenosis or regurgitation due to progressive calcification of the aortic valve or annulus leading to haemodynamically moderate or severe aortic stenosis or regurgitation. Cases were determined by echocardiography. Calcific aortic valve disease in the GBD did not include aortic valve disease with an aetiology that was congenital, rheumatic, or infectious. Disease due to these aetiologies are modelled in other causes in the GBD. Information on unicuspid or bicuspid valves was generally not available and is often unknown in advanced calcific disease. Therefore, we included cases of unicuspid or bicuspid valves in our case definition if they developed clinically significant aortic stenosis. The criteria for aortic stenosis follow the American Heart Association/American College of Cardiology definition of haemodynamically moderate or severe aortic stenosis and are listed in Table 1. The criteria for aortic regurgitation follow the American Heart Association/American College of Cardiology definition of haemodynamically moderate or severe aortic regurgitation and are listed in Table 2. Mild haemodynamic aortic stenosis or regurgitation was not included in our case definition because mildly abnormal haemodynamic parameters are difficult to differentiate from non-pathological stenosis and/or regurgitation, and are generally not reported in population-based studies.

Table 1: AHA/ACC definitions of aortic stenosis

Maximum jet velocity ≥ 3 m/s
Mean pressure gradient ≥ 20 mmHg

Table 2: AHA/ACC definitions of aortic regurgitation

Central jet mitral regurgitation ≥ 25% of the left ventricular outflow tract
Vena contracta ≥ 0.3 cm
Regurgitant volume ≥ 30 mL/beat
Regurgitant fraction ≥ 30%
Angiography grade ≥ 2+

Degenerative mitral valve disease

Degenerative mitral valve disease was defined as myxomatous degeneration of the mitral valve leading to regurgitation or prolapse. Cases were determined by echocardiography by a physician. Degenerative mitral valve disease did not include mitral valve disease with an aetiology that was congenital, rheumatic, infectious, traumatic, carcinoid, or functional (ie, secondary to left ventricular remodeling due to heart failure from another cause). Mitral valve stenosis was always considered to have a rheumatic aetiology and therefore was not included in the definition of degenerative mitral valve disease. Degenerative mitral valve disease was restricted to persons at or above the age of 15 in order to exclude congenital mitral valve disorders. This age restriction is consistent with other progressive

cardiovascular diseases modelled in the GBD. The criteria for mitral regurgitation follow the American Heart Association/American College of Cardiology definition of haemodynamically progressive or severe mitral regurgitation and are listed in Table 3. Mild haemodynamic mitral regurgitation was not included in our case definition because mild mitral valve disease cannot be differentiated from nonpathological regurgitation and is generally not reported in population-based studies.

Table 3: AHA/ACC definitions of mitral regurgitation

Central jet mitral regurgitation > 20% of the left atrium
Vena contracta ≥ 0.7 cm
Regurgitant volume ≥ 60 mL/beat
Regurgitant fraction ≥ 50%
Effective regurgitant orifice ≥ 0.4 cm^2
Angiography grade ≥ 2+

Other non-rheumatic valve disease

Other non-rheumatic valve disease is a residual category that captures non-rheumatic, non-congenital valve disorders of the tricuspid and pulmonary valves. This includes tricuspid regurgitation, tricuspid stenosis, pulmonary regurgitation, and pulmonary stenosis. Other non-rheumatic valve disease did not include tricuspid or pulmonary valve disease with an aetiology that was congenital, rheumatic, infectious, traumatic, carcinoid, or functional (ie, secondary to heart failure due to another cause).

Input data

Data on the prevalence, incidence, treatment, haemodynamic severity, and asymptomatic status were collected from PubMed using the following search strings on 8/21/2017:

Calcific aortic valve disease

("aortic stenosis"[Title/Abstract] OR "aortic regurgitation"[Title/Abstract]) NOT ("Transcatheter Aortic Valve Replacement"[MeSH] OR "Transcatheter aortic valve implantation"[KEYWORD]) AND (epidemiology[MeSH Major Topic] OR epidemiology[Subheading] OR epidemiology[MeSH Terms] OR prevalence[Title/Abstract] OR mortality[Title/Abstract]) NOT (animals[MeSH] NOT humans[MeSH]) AND ("1980/1/01"[PDAT] : "2017/12/31"[PDAT]) NOT Comment[ptyp] NOT Case Reports[ptyp]

Degenerative mitral valve disease

("mitral stenosis"[Title/Abstract] OR "mitral regurgitation"[Title/Abstract]) AND ("epidemiology"[MeSH Major Topic] OR "epidemiology"[Subheading] OR "epidemiology"[MeSH Terms] OR prevalence[Title/Abstract] OR mortality[Title/Abstract]) NOT (animals[MeSH] NOT humans[MeSH]) AND ("1980/1/01"[PDAT] : "2017/12/31"[PDAT]) NOT Comment[ptyp] NOT Case Reports[ptyp]

Other non-rheumatic valve disease

We did not run a literature review for "other non-rheumatic valve diseases" because we did not directly model non-fatal burden due to this cause.

We excluded literature that was not representative, included rheumatic, endocarditic, or congenital heart disease in its case definition, or included haemodynamically mild valve disease in its case definition.

Data on the prevalence of calcific aortic valve and degenerative mitral valve disease were also obtained from inpatient hospital data. These data were adjusted for multiple visits, non-primary diagnoses, and inpatient to outpatient utilisation ratios. Hospital data were excluded below age 30 or if the age-series for a given hospital data source was implausible. Prevalence data from both inpatient and outpatient hospital claims were used in the United States.

For GBD 2019, we used the modeling software Meta-Regression, Baysian Regularized Trimming (MR-BRT) to correct for biases in data types, replacing the in-DisMod crosswalks used in GBD 2017. We used a network meta-analysis to adjust inpatient data, MarketScan data from 2010-2016, and MarketScan data from 2000, which used a different sampling methodology than other years, to literature and inpatient data. Tables 4 and 5 show MR-BRT crosswalk adjustment factors.

MR-BRT was used to split both-sex data points into sex-specific estimates. This methodology is detailed elsewhere in the appendix. We also split data points where the age range was greater than 25 years. Age splitting was based on the global sex-specific age pattern from a Dismod model that only used input data from scientific literature with less than a 25-year age range.

Source counts

	Measure	Total sources	Countries with data
Calcific aortic valve disease	Prevalence	221	35
Calcific aortic valve disease	Case fatality rate	1	1
Degenerative mitral valve disease	Prevalence	198	30
Degenerative mitral valve disease	With-condition mortality rate	1	1
Degenerative mitral valve disease	Case fatality rate	1	1

Table 4: MR-BRT adjustment factors for calcific aortic valve disease

$$Estimated\ Reference\ Def = invlogit(logit(Alternative\ Def) - Beta_{Alternative\ Def} - Beta_{Sex} * Sex - Beta_{Ag\ \ scaled} * Age\ Scaled)$$

Data input	Reference or alternative case definition	Gamma	Beta Coefficient, Logit (95% CI)	Beta Coefficient, real-space
Literature	Reference	0.07	---	---

Inpatient	Alternate		-1.08 (-1.27, -0.89)	0.25
Marketscan, 2000	Alternate		-0.78 (-0.98, -0.58)	0.31
Marketscan, 2010-2016	Alternate		-0.04 (-0.23, 0.15)	0.49
Age, scaled			0.45 (0.32, 0.59)	0.61
Male			0.06 (-0.08, 0.19)	0.51

Table 5: MR-BRT adjustment factors for degenerative mitral valve disease

$$Estimated\ Reference\ Def = invlogit(logit(Alternative\ Def) - Beta_{Alternative\ Def} - Beta_{Sex} * Sex - Beta_{Age_{scaled}} * Age\ Scaled)$$

Data input	Reference or alternative case definition	Gamma	Beta Coefficient, Logit (95% CI)	Beta Coefficient, real-space
Literature	Reference	0.07	---	---
Inpatient	Alternate		-1.88 (-2.34, -1.43)	0.13
Marketscan, 2000	Alternate		-1.53 (-1.99, -1.06)	0.18
Marketscan, 2010-2016	Alternate		-0.82 (-1.28, -0.37)	0.31
Age, scaled			0.41 (0.03, 0.80)	0.60
Male			0.01 (-0.38, 0.39)	0.50

Modelling strategy

For other non-rheumatic valve diseases, we estimated nonfatal burden using the cause of death heart failure approach. This method is used for most cardiovascular diseases that cause heart failure and is described in detail in the appendix section on heart failure.

In order to estimate non-fatal burden for calcific aortic valve disease and degenerative mitral valve disease, we first determined the sequelae and corresponding health states that result from these conditions. This information, along with the disability weights applied to each health state, are displayed in Table 6.

Table 6: Sequelae, health state lay descriptions, and disability weights

Sequela	Health state name	Health state lay description	Disability weight
Asymptomatic non-rheumatic valve disease	Asymptomatic	--	0

Non-rheumatic valve disease after treatment	Generic uncomplicated disease: worry and daily medication	Has a chronic disease that requires medication every day and causes some worry but minimal interference with daily activities.	0.049 (0.031–0.072)
Mild heart failure due to non-rheumatic valve disease	Heart failure, mild	Is short of breath and easily tires with moderate physical activity, such as walking uphill or more than a quarter-mile on level ground. The person feels comfortable at rest or during activities requiring less effort.	0.041 (0.026–0.062)
Moderate heart failure due to non-rheumatic valve disease	Heart failure, moderate	Is short of breath and easily tires with minimal physical activity, such as walking only a short distance. The person feels comfortable at rest but avoids moderate activity.	0.072 (0.047–0.103)
Severe heart failure due to non-rheumatic valve disease	Heart failure, severe	Is short of breath and feels tired when at rest. The person avoids any physical activity, for fear of worsening the breathing problems.	0.179 (0.122–0.251)

To model the burden due to each of the sequela above, we first modelled the overall prevalence of combined haemodynamically moderate and severe calcific aortic valve disease and degenerative mitral valve disease. We then estimated the proportion of those with prevalent disease who were haemodynamically moderate, assuming that this would approximate the proportion who were asymptomatic. We next estimated the proportion of those with symptomatic disease (ie, those with haemodynamically severe disease) who were treated. The remaining proportion – those with untreated symptomatic disease – was split into four proportions: 1) controlled, medically managed; 2) mild; 3) moderate; and 4) severe heart failure. All proportions were calculated and converted to population prevalence at the draw level, thus propagating uncertainty from each step through to all subsequent steps. Population prevalence for each severity level are necessary in order to accurately calculate the burden for these diseases. Figure 1 visualises this framework. Each of these modelling steps is outlined in greater detail below.

Figure 1: Modelling framework for calcific aortic valve disease and degenerative mitral valve disease

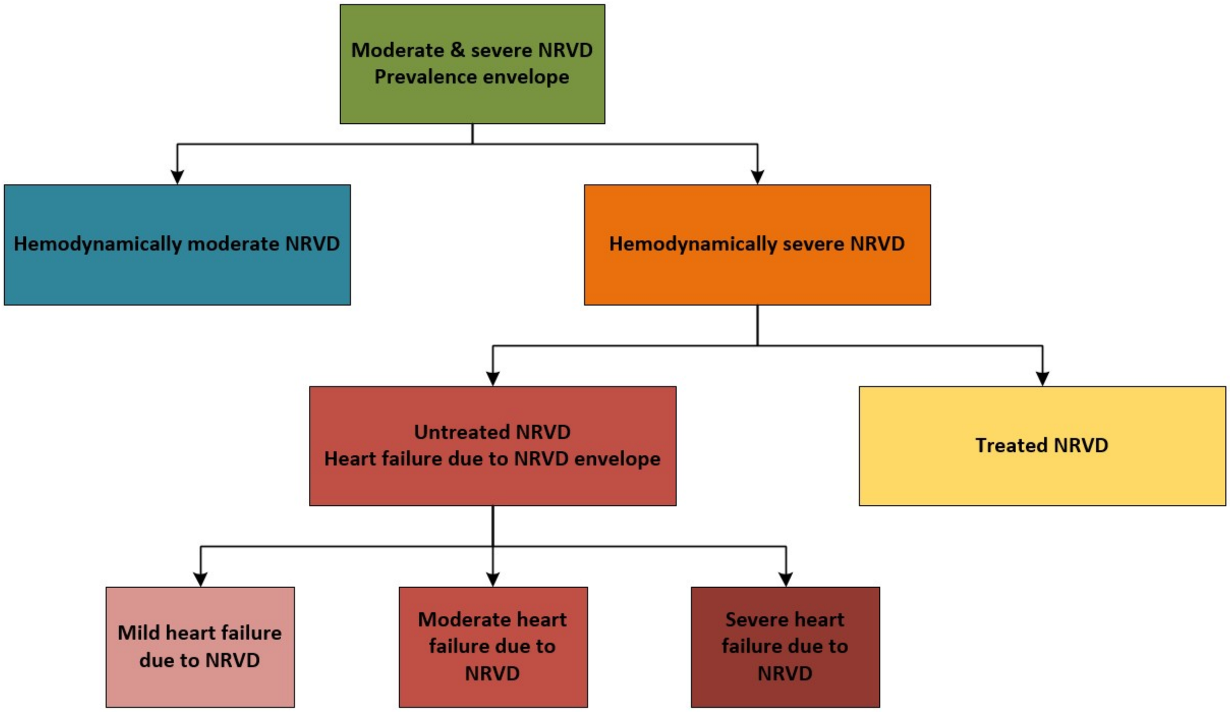

Prevalence envelope

We separately modelled the overall prevalence of calcific aortic valve disease and degenerative mitral valve disease in DisMod-MR 2.1. We used cause-specific mortality rates from the fatal modelling process as inputs. These two models estimate the prevalence of these two valve diseases for each age, sex, location, and year. Covariates included in the DisMod models for prevalence of calcific aortic valve and degenerative mitral valve disease are presented in tables 9 and 10.

Table 9: Covariates and resulting coefficients for calcific aortic valve disease DisMod model

Covariate	Integrand	Coefficients	Exponentiated coefficients
Mean BMI	Prevalence	1.76 (1.74-1.77)	5.79 (5.72-5.88)
Smoking Prevalence	Prevalence	0.0026 (0.000086 to 0.0095)	1.00 (1.00 to 1.01)
HAQ index	Excess mortality rate	-0.079 (-0.082 to -0.077)	0.92 (0.92 to 0.93)

Table 10: Covariates and resulting coefficients for degenerative mitral valve disease DisMod model

Covariate	Integrand	Coefficients	Exponentiated coefficients
HAQ index	Excess mortality rate	-0.073<br>(-0.18 to –0.005)	0.93<br>(0.84 to 1.00)

Haemodynamically moderate proportion

We estimated the proportion of individuals with haemodynamically moderate or severe valve disease who were haemodynamically moderate. As mentioned above, we assumed that individuals with haemodynamically moderate disease were asymptomatic. There were a total of five data sources that reported the proportion of individuals who were haemodynamically moderate. Because of the sparsity of data, we modelled the haemodynamically moderate proportion together for both calcific aortic valve disease and degenerative mitral valve disease. We modelled a proportion with uncertainty that varied by age with the following regression:

$$logit(y) = \beta_0 + \beta_1 age + \gamma$$

Where y is the proportion of haemodynamically moderate disease, *age* is the midpoint age for each data point, and γ is a random effect for each data source. The regression coefficients are reported in Table 11.

Table 11: Moderate NRVD regression coefficients

Covariate	Coefficients	Transformed coefficients
Intercept (β_0)	6.6 (4.9 to 8.4)	0.998 (0.992 to 0.999)
Age (β_1)	-0.07 (-0.093 to -0.047)	0.932 (0.911 to 0.954)

The prevalence of those with haemodynamically moderate valve disease and the prevalence of those with haemodynamically severe disease were calculated using the prevalence envelope and the proportion of those with haemodynamically moderate disease for each five-year age group, sex, location, and year.

Treated proportion

We estimated the proportion of individuals who had haemodynamically severe disease who had been treated. Treatment was defined as valve replacement or repair. We assumed that treatment was not performed on any individuals with only haemodynamically moderate disease. The number of data points are reported in Table 10.

Table 12: Data on treated calcific aortic and degenerative mitral valve disease

Input data	Number of data points
**Unique sources**	**23**
**Geography-years**	**35**

These data were all from relatively high-income geographies, yet it is important that we capture the difference in treatment between high- and low-income locations. Because of this challenge, we ran a regression using the Healthcare Access and Quality (HAQ) index predicting the level of treatment and set a prior that the proportion of individuals with a valve replacement or repair was zero where HAQ index was equal to zero. This assumption allowed us to estimate an increasing relationship between HAQ index and proportion treated, where the estimated proportion treated was based on data where HAQ index was high. We used the regression equation:

$$logit(y) = \alpha + \beta_1 * haqi + \beta_2 * age + \beta_3 * severity$$

where y is the proportion of individuals with haemodynamically severe disease who had a valve replacement or repair, $haqi$ is the Healthcare Access and Quality index, age is the midpoint of the age range for a given data point, and $severity$ is an indicator variable to adjust for data points where the denominator of the proportion treated included both haemodynamically moderate and haemodynamically severe individuals. The prevalence of those with treated valve disease and the prevalence of those with untreated haemodynamically severe disease were calculated using the prevalence of haemodynamically severe disease and the proportion of those with treated valve disease. The results of this regression are reported in Table 13 and plotted for three ages in Figure 2.

Table 13: Treated calcific aortic valve and degenerative mitral valve disease regression coefficients

Covariate	Coefficients	Transformed coefficients
Intercept (β_0)	-4.69 (-5.90 to -3.43)	0.009 (0.003 to 0.032)
HAQI (β_1)	0.080 (0.070 to 0.089)	1.083 (1.073 to 1.093)
Age (β_2)	-0.029 (-0.04 to -0.015)	0.971 (0.957 to 0.985)
Severity (β_3)	-0.947 (-1.40 to -0.54)	0.377 (0.246 to 0.578)

Figure 2: Results of treatment model for three ages

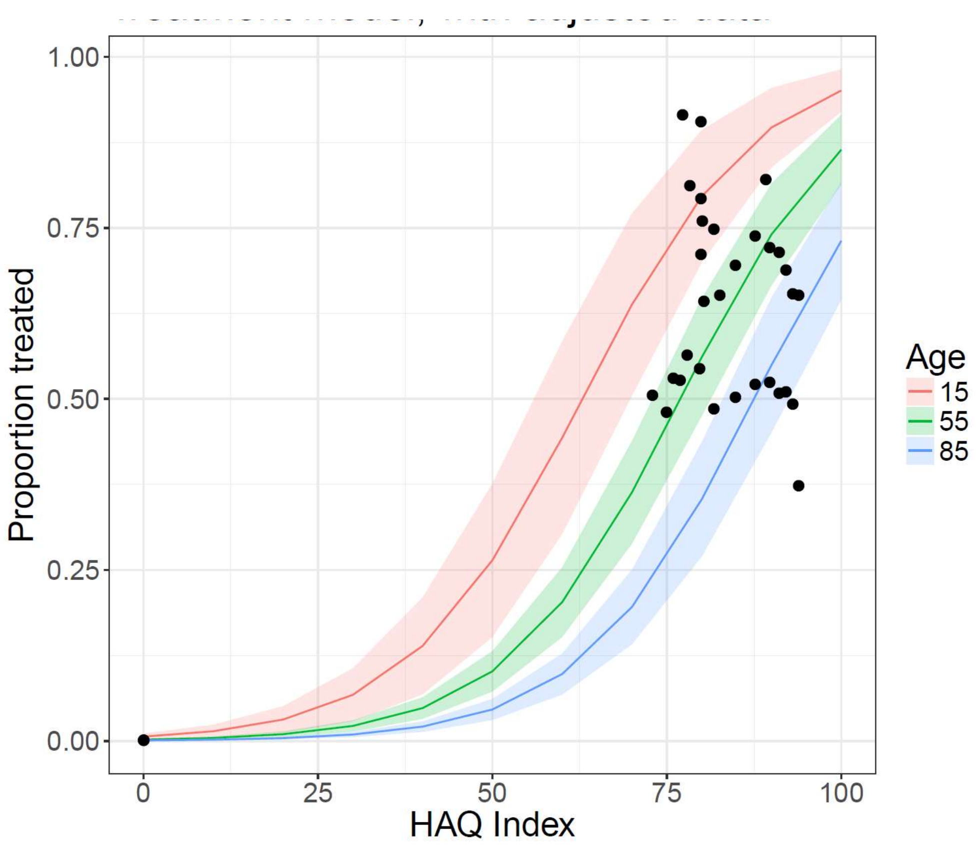

Final burden estimation

The proportions of 1) controlled, medically managed, 2) mild, 3) moderate and 4) severe heart failure due to valve disease were estimated using the approach described in the heart failure section of the appendix. Prevalence for each of these health states was estimated using the prevalence of haemodynamically severe disease and the corresponding proportion for each severity of heart failure. Burden due to each severity of valve disease was estimated by multiplying the prevalence of each severity by the corresponding disability weight.

Acute Endocarditis

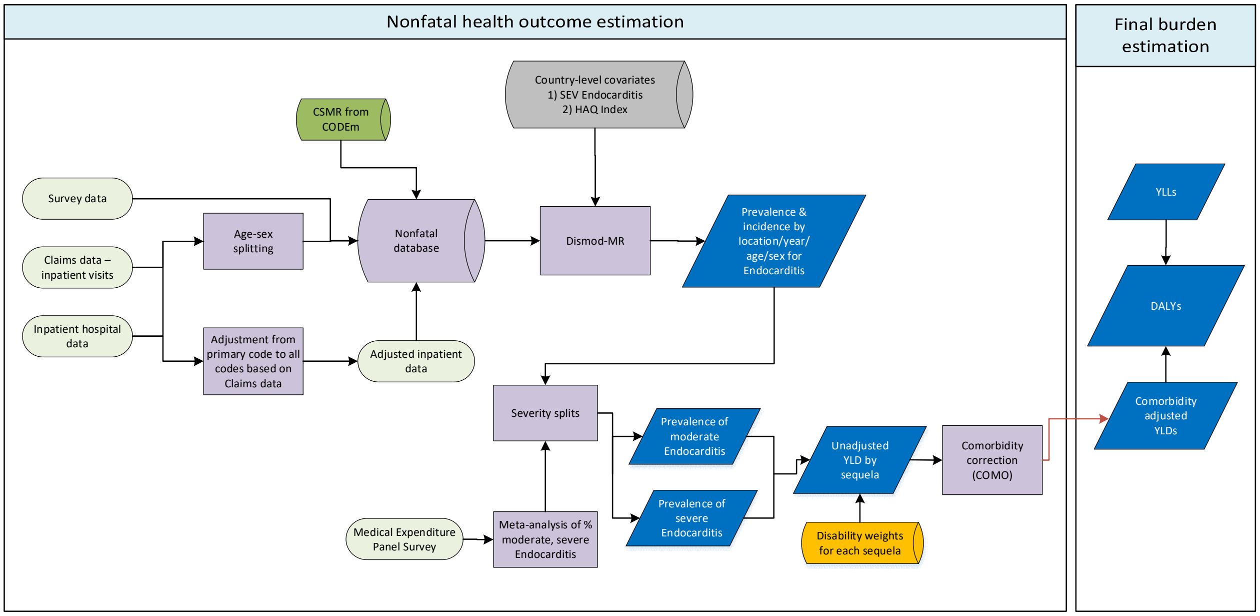

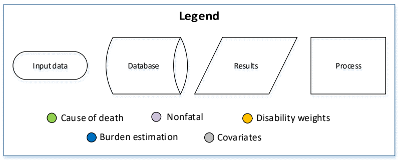

Input data and methodological appendix

Case definition

Our case definition for acute endocarditis was a clinical diagnosis of infective endocarditis. The ICD codes included can be found elsewhere in the appendix.

Input data

Model inputs

Table 1: Source counts for acute endocarditis

Measure	Total sources	Countries with data
All measures	303	41
Incidence	303	41

Table 1 displays the source counts for the non-fatal acute endocarditis model. We did not perform a systematic review for GBD 2019. A systematic review was performed for GBD 2013 and updated for GBD 2015. . The following search terms were used: ((‘endocarditis’[MeSH Terms] OR ‘endocarditis’[All Fields]) AND ‘epidemiology’[Subheading]) OR ((‘endocarditis’[MeSH Terms] OR ‘endocarditis’[All Fields]) AND ((‘epidemiology’[Subheading] OR ‘epidemiology’[All Fields] OR ‘incidence’[All Fields] OR ‘incidence’[MeSH Terms]) OR (‘epidemiology’[Subheading] OR ‘epidemiology’[All Fields] OR

‘prevalence’[All Fields] OR ‘prevalence’[MeSH Terms]) OR ‘case fatality’[All Fields])) OR ((‘endocardium’[MeSH Terms] OR ‘endocardium’[All Fields]) AND inflammation[TIAB] AND ‘epidemiology’[Subheading]) OR ((‘endocardium’[MeSH Terms] OR ‘endocardium’[All Fields]) AND inflammation[TIAB] AND ((‘epidemiology’[Subheading] OR ‘epidemiology’[All Fields] OR ‘incidence’[All Fields] OR ‘incidence’[MeSH Terms]) OR (‘epidemiology’[Subheading] OR ‘epidemiology’[All Fields] OR ‘prevalence’[All Fields] OR ‘prevalence’[MeSH Terms]) OR ‘case fatality’[All Fields]))

- Dates included in search: 1/1/2013 – 3/16/2015
- Number of initial hits: 1,246
- Number of sources included: 6

We did not include any non-literature-based data types, apart from the hospital and claims data described elsewhere. We excluded all outpatient data, as they were implausibly low when compared with inpatient data from the same locations and claims data. We used hospital data corrected for readmission and primary to any diagnosis based on the correction factors generated by the clinical informatics team. We excluded any inpatient hospital data points which were more than two-fold higher or 0.5-fold lower than the median absolute deviation value for high-income North America, Central Europe, and Western Europe for that age-sex group. No data adjustments was done for acute endocarditis in GBD 2019.

Severity split inputs

We used the standard GBD approach, which utilises MEPS data to split overall estimates of endocarditis into moderate and severe categories. The table below includes the severity level, lay descriptions, and DWs associated with acute endocarditis.

Table 2. Severity distribution, details on the severity levels for Acute Endocarditis in GBD 2019 and the associated disability weight (DW) with that severity.

Severity level	Lay description	DW (95% CI)
Moderate	Has a fever and aches, and feels weak, which causes some difficulty with daily activities.	0.051 (0.032–0.074)
Severe	Has a high fever and pain, and feels very weak, which causes great difficulty with daily activities.	0.133 (0.088–0.19)

Modelling strategy

For GBD 2019, we estimated endocarditis using a DisMod-MR Bayesian meta-regression model, setting a minimum of 11 and maximum of 13 as value priors on remission to establish an average duration of one month. For GBD 2019, we outliered cause specific mortality rate data from Mali due to implausibly high estimates. Country-level covariates used included the endocarditis summary exposure variable (SEV) on incidence and Health Access and Quality Index on excess mortality.

We evaluated models by comparing model fits with the data and with results from previous GBD estimation cycles.

The table below gives the parameters, betas, and exponentiated betas for study-level and country-level covariates used in the model.

Table 3. Covariates. Summary of covariates used in the Acute Endocarditis DisMod-MR meta-regression model

Covariate	Parameter	Beta	Exponentiated beta (95% Uncertainty Interval)
Health Access and Quality Index	Excess mortality rate	-0.1 (-0.1 to -0.1)	0.90 (0.90 to 0.90)
Log-transformed age-standardised SEV scalar: endocarditis	Incidence	0.78 (0.75 to 0.83)	2.19 (2.12 to 2.30)

No significant changes were made to the modelling strategy from GBD 2017.

Peripheral arterial disease

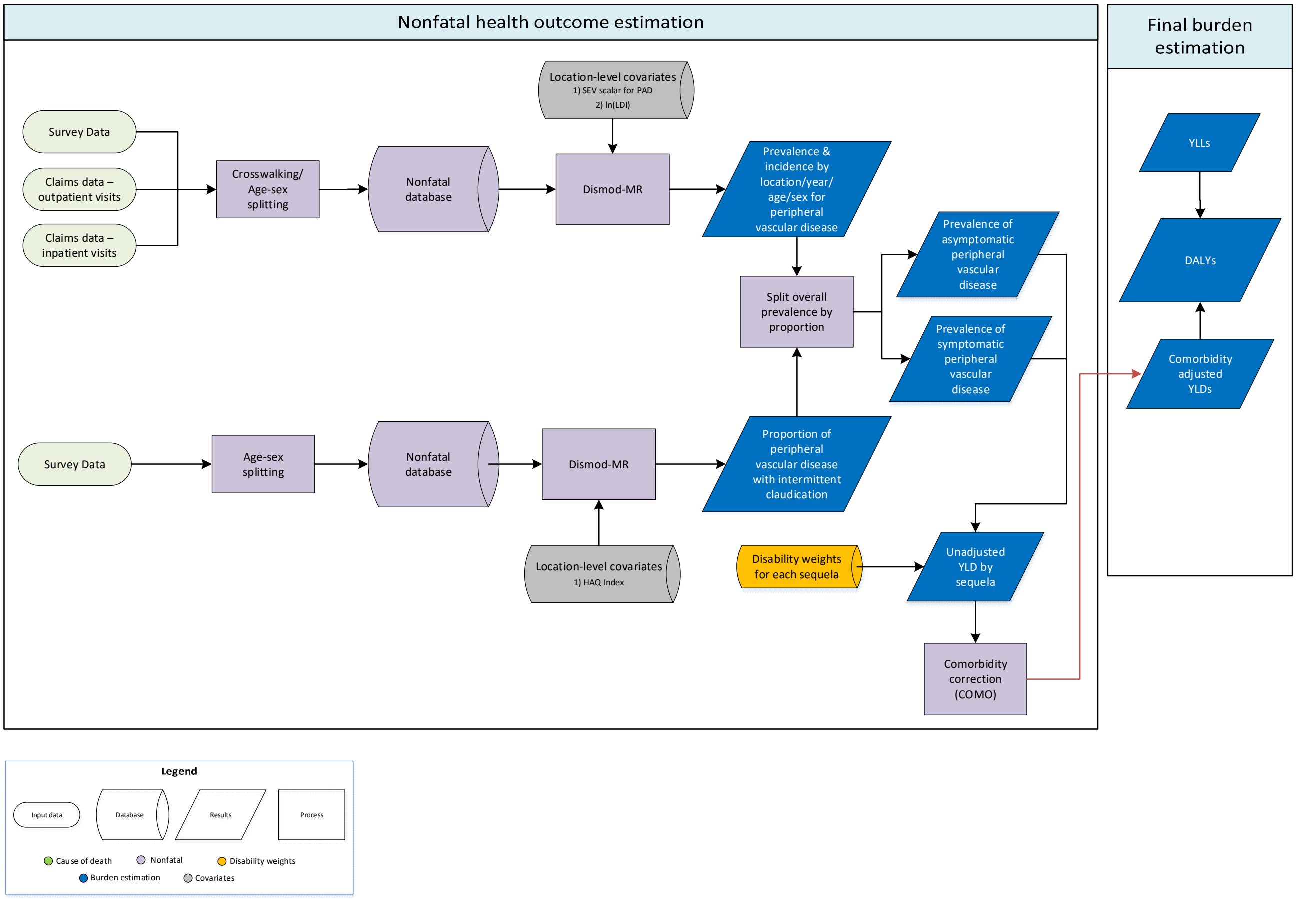

Input data and methodological appendix

Case definition

For GBD 2019, peripheral arterial disease was defined as having an ankle-brachial index (ABI) < 0.9. Intermittent claudication was defined clinically.

Specific ICD codes for claims data included can be found elsewhere in the appendix.

Input data

Model inputs

Table 1: Source counts for peripheral arterial disease

Measure	Total sources	Countries with data
All measures	45	15
Prevalence	37	14
Proportion	11	4

Table 1 shows the source counts for peripheral arterial disease modeling. We did not perform a systematic review for GBD 2019. A systematic review was performed for peripheral arterial disease and intermittent claudication for GBD 2015. The search terms were: ('peripheral vascular disease'[TIAB] AND 'epidemiology'[Subheading]) OR ('peripheral arterial disease'[TIAB] AND 'epidemiology'[Subheading]) OR ('peripheral artery disease'[TIAB] AND 'epidemiology'[Subheading]) OR ('intermittent claudication'[TIAB] AND 'epidemiology'[Subheading]) OR ('ankle-brachial index'[TIAB] AND 'epidemiology'[Subheading]) OR ('ankle brachial index'[TIAB] AND 'epidemiology'[Subheading]) OR ('peripheral artery occlusive disease'[TIAB] AND 'epidemiology'[Subheading]) OR ('peripheral obliterative arteriopathy'[TIAB] AND 'epidemiology'[Subheading]) OR ('peripheral vascular disease'[TIAB] AND 'prevalence'[MeSH Terms]) OR ('peripheral vascular disease'[TIAB] AND 'incidence'[MeSH Terms]) OR ('peripheral vascular disease'[TIAB] AND 'case fatality'[All Fields]) OR ('symptomatic claudication'[TIAB] AND (proportion[All Fields] OR percent[All Fields]))

The search was conducted from 1/1/2013 to 3/16/2015. 1,658 results were returned, of which six were extracted.

A systematic review was also performed for peripheral arterial disease and intermittent claudication for GBD 2013. Search terms can be provided upon request.

Apart from the claims data from the United States, we did not include any non-literature-based data types. We did not use inpatient hospital data, as peripheral arterial disease is expected to be rare in inpatient data but common in outpatient data as it is a condition usually managed on an outpatient basis, except for specific surgical interventions. This discrepancy leads to implausible correction factors based on inpatient/outpatient information from claims data (~150X); thus, adjusted data cannot be used. Including uncorrected data in the model is likely to lead to incorrect estimates as hospitalisation and procedure rates are likely to vary between geographies based on access to and patterns of care.

For GBD 2019 we adjusted prevalence data from claims using the MR-BRT data adjustment procedure described elsewhere the appendix. Our reference data was from literature in which the prevalence of PAD was based on directly-measured ABI values. The coefficients in Table 2 below can be used to

calculate adjustment factors for alternative definitions. The formula for computing adjustment factors is given in equation 1 below. We also included a standardized age variable (age scaled) and a sex variable to the crosswalking procedure to adjust for the possibly of bias.

Equation 1: Calculation of adjustment factors:

$$Estimated\ Reference\ Def = invlogit(logit(Alternative\ Def) - Beta_{Alternative\ Def} - Beta_{Sex} * Sex - Beta_{Age_{scaled}} * Age\ Scaled)$$

Table 2: MR-BRT Crosswalk Adjustment Factors for Peripheral Arterial Disease

Data input	Measure	Reference or alternative case definition	Gamma	Beta Coefficient,Logit (95% CI)
Measured ABI less than or equal to 0.90	Prevalence	Ref	0	---
Claims data	Prevalence	Alt		-1.87 (-1.92 to -1.82)
Age scaled	Prevalence	Alt		0.27 (0.23 to 0.31)
Sex (male)	Prevalence	Alt		0.29 (0.22 to 0.36)

Severity splits and disability weights

We used the proportion of intermittent claudication to split the overall prevalence of peripheral arterial disease into symptomatic and asymptomatic peripheral vascular disease. The table below illustrates these values:

Table 3. Severity distribution, details on the severity levels for Peripheral Arterial Disease in GBD 2019 and the associated disability weight (DW) with that severity.

Severity level	Lay description	DW (95% CI)
Asymptomatic	No symptoms	No DW assigned
Symptomatic	Has cramping pains in the legs after walking a medium distance. The pain goes away after a short rest.	0.014 (0.007–0.025)

Modelling strategy

For GBD 2019, we used DisMod MR 2.1 to model the overall prevalence of peripheral arterial disease using prevalence data from literature studies and and crosswalked claims data.

We included the log-transformed, age-standardised SEV scalar for PAD and log-transformed LDI as fixed-effect, country-level covariates. We set value priors of 0 for incidence from ages 0 to 30. We also set a value prior of 0 for remission for all ages. Additionally, we set a value prior of 0 for excess mortality inbetween ages 0 and 30 as well as a value prior between 0 and 0.05 for excess mortality inbetween ages 30 and 100.

The table below illustrate the beta values and and exponentiated beta values for the covariates chosedn for the overall peripheral vascular disease model.

Table 4a. Covariates. Summary of covariates used in the Peripheral Arterial Disease DisMod-MR meta-regression model

Covariate	Parameter	Beta	Exponentiated beta
Log-transformed age-standardised SEV scalar: PAD	Prevalence	1.24 (1.22 to 1.25)	3.46 (3.39 to 3.49)
LDI (I$ per capita)	Excess mortality rate	-0.3 (-0.5 to -0.1)	0.74 (0.61 to 0.90)

We used DisMod MR to model the proportion of peripheral vascular disease with intermittent claudication. We set a value prior of 0 for proportion for ages 0 to 40. We included the Health Access and Quality Index score as a country-level covariate for excess mortality.

The table below illustrate the study covariates, parameters, beta, and exponentiated beta values for the proportion model for intermittent claudication.

Table 4b. Covariates. Summary of covariates used in the Intermittent Claudication DisMod-MR meta-regression model

Covariate	Parameter	Beta	Exponentiated beta
Healthcare Access and Quality index	Proportion	-.0064 (-.014 to -.00066)	0.99 (.99 to 1.00)

To obtain final estimates for the sequelae of interest, we multiplied the prevalence model by the proportion model at the draw level to generate the prevalence of symptomatic and asymptomatic peripheral vascular disease.

Models were evaluated based on expert review, comparisons with estimates from prior rounds of GBD, and assessing model fit.

There have been no substantive changes from GBD 2017 in terms of modelling strategy for peripheral arterial disease.

Risk-specific modelling descriptions

High systolic blood pressure

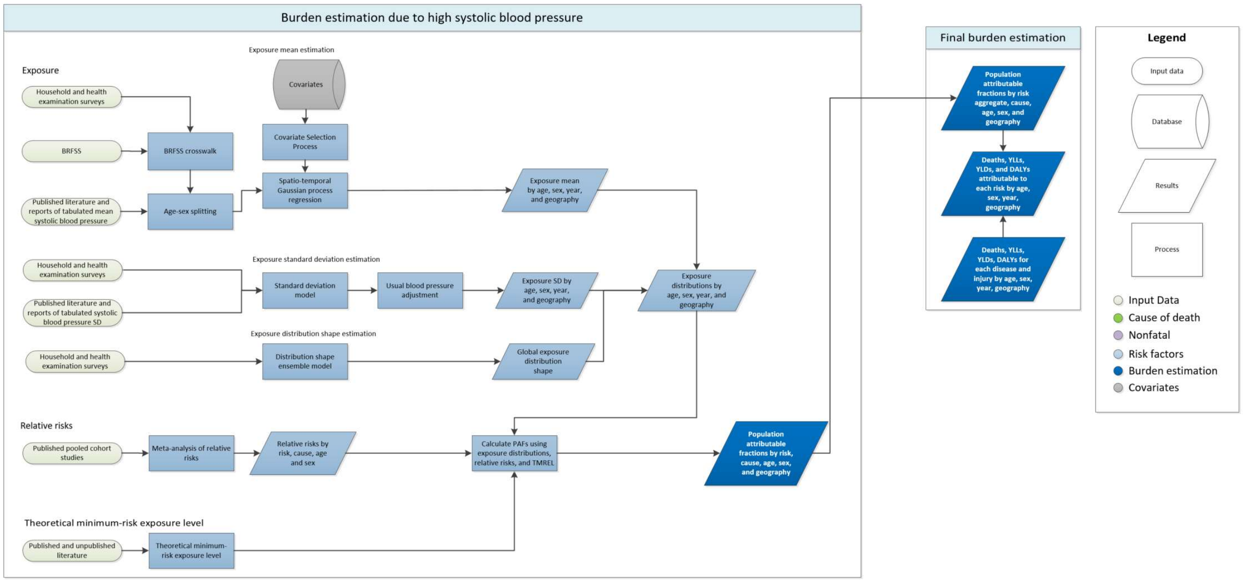

Input data and methodological summary

Exposure

Case definition

Brachial systolic blood pressure in mmHg.

Input data

We utilised data on mean systolic blood pressure from literature and from household survey microdata and reports (e.g. STEPS, NHANES). For GBD 2019, we did not carry out a systematic review of the literature for new data. Counts of the data inputs used for GBD 2019 are show in Tables 1 and 2 below. Details of inclusion and exclusion criteria and data processing steps follow.

Table 1: Data inputs for exposure for systolic blood pressure.

Input data	Exposure
Total sources	1112
Number of countries with data	166

Table 2: Data inputs for relative risks for systolic blood pressure.

Input data	Relative risk
Source count (total)	3

Inclusion criteria

Studies were included if they were population-based and directly measured systolic blood pressure using a sphygmomanometer. We assumed the data were representative if the geography or the population were not selected because it was related to hypertension or hypertensive outcomes.

Outliers
Data were utilised in the modelling process unless an assessment strongly suggested that the source was biased. A candidate source was excluded if the quality of study did not warrant a valid estimate because of selection (non-representative populations) or if the study did not provide methodological details for evaluation. In a small number of cases, a data point was considered to be an outlier candidate if the level was implausibly low or high based on expert judgement and data from other country data.

Data extraction
Where possible, individual-level data on blood pressure estimates were extracted from survey microdata. These data points were collapsed across demographic groupings to produce mean estimates in the standard GBD five-year age-sex groups. If microdata were unavailable, information from survey reports or from literature were extracted along with any available measure of uncertainty including standard error, uncertainty interval, and sample size. Standard deviations were also extracted. Where mean systolic blood pressure was reported split out by groups other than age, sex, location, and year (e.g. by hypertensive status), a weighted mean was calculated.

Incorporating United States prevalence data
Survey reports and literature often report information only about the prevalence, but not the level, of hypertension in the population studied. These sources were not used to model systolic blood pressure, with the exception of data from the Behavioral Risk Factors Surveillance System (BRFSS) because of the availability of a similarly structured exam survey that is representative of the same population (NHANES). BRFSS is a telephone survey conducted in the United States for all US counties. It collects self-reported diagnosis of hypertension. These self-reported values of prevalence of raised blood pressure were adjusted for self-report bias and tabulated by age group, sex, US state, and year. These prevalence values were used to predict a mean systolic blood pressure for the same strata with a regression using data from the National Health and Nutrition Examination Survey, a nationally representative health examination survey of the US adult population. The regression was run separately by sex, and was specified as:

$$\mathrm{SBP}_{l,a,t,s} = \beta_0 + \beta_1 \mathrm{prev}_{l,a,t,s}$$

where $\mathrm{SBP}_{l,a,t,s}$ is the location, age, time, and sex specific mean systolic blood pressure and $\mathrm{prev}_{l,a,t,s}$ is the location, age, time, and sex specific prevalence of raised blood pressure. The coefficients for both models are reported in Table 3.

Table 3. Coefficients in the sex-specific US states blood pressure prediction models

Term	Male model	Female model
Intercept (β_0)	114.65	108.28
Prevalence (β_1)	51.86	68.87

Out of sample RMSE was used to quantify the predictive validity of the model. The regression was repeated 10 times for each sex, each time randomly holding out 20% of the data. The RMSEs from each holdout analysis were averaged to get the average out of sample RMSE. The results of this holdout analysis are reported in Table 4.

Table 4. Out of sample RMSEs of the sex-specific US states blood pressure prediction models

	Male model	Female model
Out of sample RMSE	2.37 mmHg	3.27 mmHg

Age and sex splitting
Prior to modelling, data provided in age groups wider than the GBD five-year age groups were processed using the approach outlined in Ng and colleagues.[2] Briefly, age-sex patterns was identified using 115 sources of microdata with multiple age-sex groups, and these patterns were applied to estimate age-sex-specific levels of mean systolic blood pressure from aggregated results reported in published literature or survey reports. In order to incorporate uncertainty into this process and borrow strength across age groups when constructing the age-sex pattern, we used a model with auto-regression on the change in mean SBP over age groups:

$$\mu_a = \mu_{a-1} + \omega_a$$
$$\omega_a \sim N(\omega_{a-1}, \tau)$$

Where μ_a is the mean predicted value for age group a, μ_{a-1} is the mean predicted value for the age group previous to age group a, ω_a is the difference in mean between age group *a* and age group *a-1*, ω_{a-1} is the difference between age group *a-1* and age group *a-2*, and τ is a user-input prior on how quickly the mean SBP changes for each unit increase in age. We used a τ of 1.5 mmHg for this model. Draws of the age-sex pattern were combined with draws of the input data needing to be split in order to calculate the new variance of age-sex split data points.

Modelling
Exposure estimates were produced from 1980 to 2019 for each national and subnational location, sex, and for each five-year age group starting from 25+. As in GBD 2017, we used a spatiotemporal Gaussian process regression (ST-GPR) framework to model the mean systolic blood pressure at the location-, year-, age-, sex- level. Details of the ST-GPR method used in GBD 2019 can be found elsewhere in the appendix.

Covariate selection
The first step of the ST-GPR framework requires the creation of a linear model for predicting SBP at the location-, year-, age-, sex- level. Covariates for this model were selected in two stages. First a list of variables with an expected causal relationship with SBP was created based on significant association found within high-quality prospective cohort studies reported in the published scientific literature. The second stage in covariate selection was to test the predictive validity of every possible combination of covariates in the linear model, given the covariates selected above. This was done separately for each sex. Predictive validity was measured with out of sample root-mean-squared error.

In GBD 2016, the linear model with the lowest root-mean-squared error for each sex was then used in the ST-GPR model. Beginning in GBD 2017, we used an ensemble model of the 50 models with the lowest root-mean-squared error for each sex. This allows us to utilise covariate information from many plausible linear mixed-effects models. The 50 models were each used to predict the mean SBP for every age, sex, location, and year, and the inverse-RMSE-weighted average of this set of 50 predictions was used as the linear prior. The relative weight contributed by each covariate is plotted by sex in Figure 1.

Figure 1. Results of the ensemble linear model covariate selection

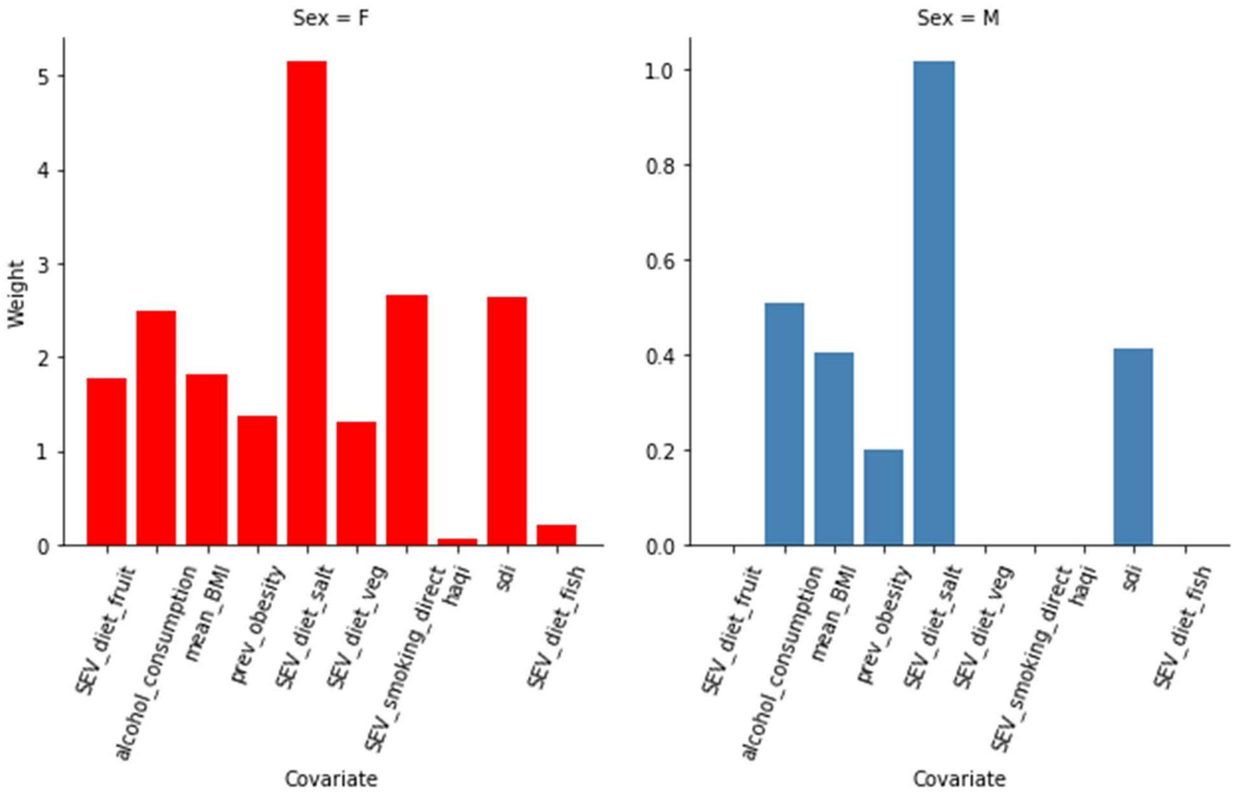

The results of the ensemble linear model were used for the first stage in an ST-GPR model. The result of the ST-GPR model are estimates of the mean SBP for each age, sex, location, and year.

Estimate of standard deviation

Currently, the ST-GPR model only produces an estimate of mean exposure level without standard deviation. Therefore, the standard deviation of systolic blood pressure within a population was estimated for each national and subnational location, sex, and five-year age group starting from age 25 using the standard deviation from person-level and some tabulated data sources. Person-level microdata accounted for 10 375 of the total 12 570 rows of data on standard deviation. The remaining 2195 rows came from tabulated data. Tabulated data were only used to model standard deviation if it was sex-specific and five-year-age-group-specific and reported a population standard deviation of systolic blood pressure. The systolic blood pressure standard deviation function was estimated using a linear regression:

$$\log(SD_{l,a,t,s}) = \beta_0 + \beta_1 \log(mean\_SBP_{l,a,t,s}) + \beta_4 sex + \sum_{k=2}^{16} \beta_k I_A$$

where $mean\_SBP_{l,a,t,s}$ is the location-, age-, time-, and sex-specific mean SBP estimate from ST-GPR, and I_A is a dummy variable for a fixed effect on a given five-year age group.

Adjustment for usual levels of blood pressure

To account for in-person variation in systolic blood pressure, a "usual blood pressure" adjustment was done. The need for this adjustment has been described elsewhere.[5] Briefly, measurements of a risk

factor taken at a single time point may not accurately capture an individual's true long-term exposure to that risk. Blood pressure readings are highly variable over time due to measurement error as well as diurnal, seasonal, or biological variation. These sources of variation result in an overestimation of the variation in cross-sectional studies of the distribution of SBP.

To adjust for this overestimation, we applied a correction factor to each location-, age-, time-, and sex-specific standard deviation. These correction factors were age-specific and represented the proportion of the variation in blood pressure within a population that would be observed if there were no within-person variation across time. Four longitudinal surveys were used to estimate these factors: the China Health and Retirement Longitudinal Survey (CHRLS), the Indonesia Family Life Survey (IFLS), the National Health and Nutrition Examination Survey I Epidemiological Follow-up Study (NHANES I/EFS), and the South Africa National Income Dynamics Survey (NIDS). The sample size and number of blood pressure measurements at each measurement period for each survey is reported in Table 5.

Table 4. Characteristics of longitudinal surveys used for the usual blood pressure adjustment

Source	Measurement periods	Number of measurements	Sample size
CHRLS	2008	3	1967
	2012	3	1419
IFLS	1997	1	19 418
	2000	1	16 626
	2007	3	14 136
NIDS	1997	2	14 084
	2000	2	9612
	2007	2	9098
NHANES I/EFS	1971–1976	2	20 716
	1982–1984	3	9932

For each survey, the following regression was created for each age group:

$$\mathrm{SBP}_{i,a} = \beta_0 + \beta_1 \mathrm{sex} + \beta_3 \mathrm{age} + +\upsilon_i$$

where $\mathrm{SBP}_{i,a}$ is the systolic blood pressure of an individual i at age a, sex is a dummy variable for the sex of an individual, age is a continuous variable for the age of an individual, and υ_i is a random intercept for each individual. Then, a blood pressure value $\widehat{\mathrm{SBP}}_{i,b}$ was predicted for each individual i for his/her age at baseline b. The correction factor cf for each age group within each survey was calculated as variation in these predicted blood pressures was divided by the variation in the observed blood pressures at baseline, $\mathrm{SBP}_{i,b}$:

$$\mathrm{cf} = \sqrt{\frac{\mathrm{var}(\widehat{\mathrm{SBP}}_b)}{\mathrm{var}(\mathrm{SBP}_b)}}$$

The average of the correction factors was taken over the three surveys to get one set of age-specific correction factors, which were then multiplied by the square of the modelled standard deviations to estimate standard deviation of the "usual blood pressure" of each age, sex, location, and year. Because of low sample sizes, the correction factors for the 75–79 age group was used for all terminal age groups. The final correction factors for each age group are reported in Table 6. Figure 2 shows the correction factors by survey and age group ID.

Table 5. Age-specific usual blood pressure correction factors

Age group	Correction factor
25–29	0.665
30–34	0.713
35–39	0.737
40–44	0.733
45–49	0.798
50–54	0.771
55–59	0.764
60–64	0.753
65–69	0.719
70–74	0.689
75+	0.678

Figure 2: Correction factor by survey and age group id. The correction factor is equal to the variance of the predictions divided by the variance of the raw dataset. In pink is the average correction factor for each age group, summarised in Table 6.

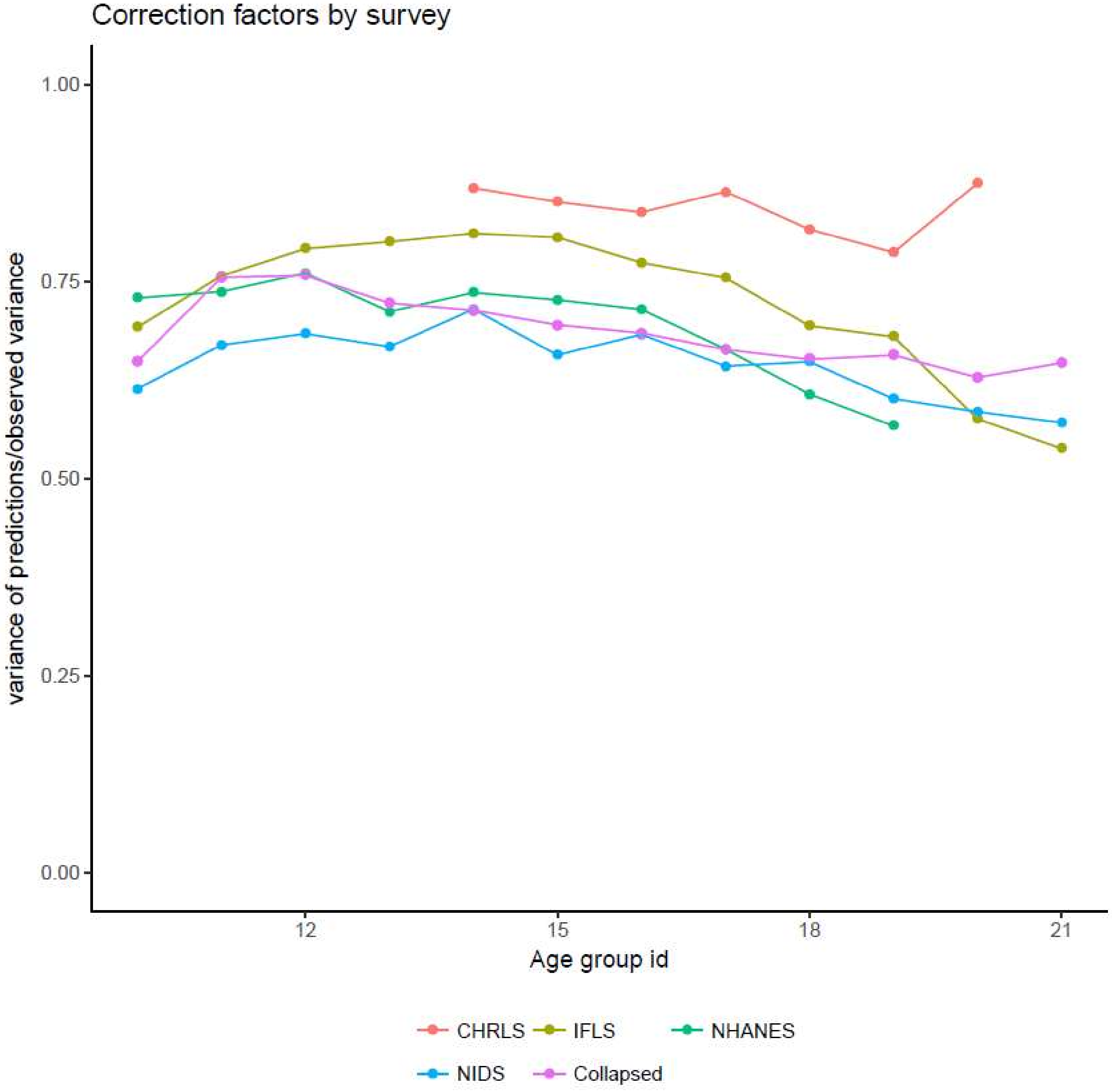

A visualisation of how the uncorrected blood pressure measurements overestimate the "usual" blood pressure variation is shown in Figure 3. This image shows the density of the distribution of the observed blood pressure values $\mathrm{SBP}_{i,b}$ in participants in the Indonesian Family Life Study survey in red, and the density of the predicted blood pressure values $\widehat{\mathrm{SBP}}_{i,b}$ in blue. The ratio of the variance of the blue distribution to the variance of the red distribution is an example of the scalar adjustment factor being applied to the modelled standard deviations.

Figure 3: Raw and predicted distributions of blood pressure in the Indonesia Family Life Survey

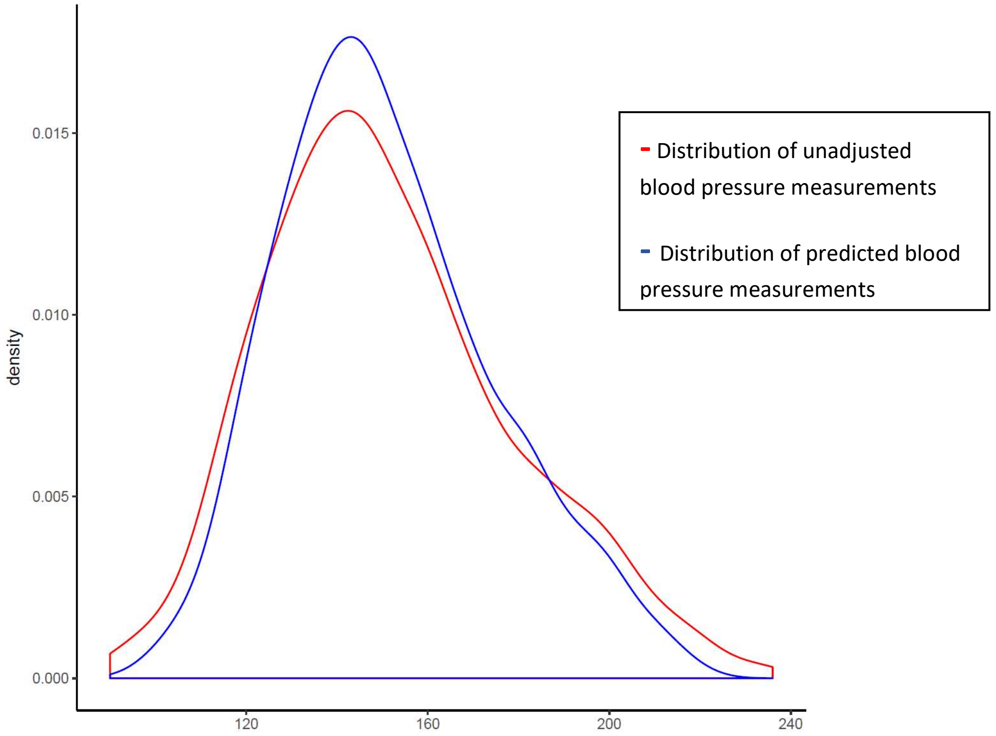

Estimating the exposure distribution shape

The shape of the distribution of systolic blood pressure was estimated using all available person-level microdata sources, which was a subset of the input data into the modelling process. The distribution shape modelling framework for GBD 2019 is detailed in the elsewhere in the appendix. Briefly, an ensemble distribution created from a weighted average of distribution families was fit for each individual microdata source, separately by sex. The weights for the distribution families for each individual source were then averaged and weighted to create a global ensemble distribution for each sex.

Theoretical minimum-risk exposure level

No changes have been made to the TMREL used for systolic blood pressure since GBD 2015. We estimated that the TMREL of SBP ranges from 110 to 115 mmHg based on pooled prospective cohort studies that show risk of mortality increases for SBP above that level.[3,4] Our selection of a TMREL of 110–115 mmHg is consistent with the GBD study approach of estimating all attributable health loss that could be prevented even if current interventions do not exist that can achieve such a change in exposure level, for example a tobacco smoking prevalence of zero percent. To include the uncertainty in the TMREL, we took a random draw from the uniform distribution of the interval between 110 mmHg and 115 mmHg each time the population attributable burden was calculated.

Relative risks

No changes have been made to the relative risk estimates for blood pressure outcomes used since GBD 2016. RRs for chronic kidney disease are from the Renal Risk Collaboration meta-analysis of 2.7 million individuals in 106 cohorts. For other outcomes, we used data from two pooled epidemiological studies:

the Asia Pacific Cohort Studies Collaboration (APCSC) and the Prospective Studies Collaboration (PSC).[4,5] Additional estimates of RR for cardiovascular outcomes were used from the CALIBER study, a health-record linkage cohort study from the UK.[6]

For cardiovascular disease, epidemiological studies have shown that the RR associated with SBP declines with age, with the log (RR) having an approximately linear relationship with age and reaching a value of 1 between the ages of 100 and 120. RRs were reported per 10 mmHg increase in SBP above the TMREL value (115 mmHg), calculated as in the equation below:

$$RR(x) = RR_0^{\frac{(x-TMRE\ \ )}{10\ mmHg}}$$

Where $RR(x)$ is the RR at exposure level x and RR_0 is the increase in RR for each 10 mmHg above the TMREL. We used DisMod-MR 2.1 to pool effect sizes from included studies and generate a dose-response curve for each of the outcomes associated with high SBP. The tool enabled us to incorporate random effects across studies and include data with different age ranges. RRs were used universally for all countries and the meta-regression only helped to pool the three major sources and produce RRs with uncertainty and covariance across ages taking into account the uncertainty of the data points.

References

1. Bangalore S, Gong Y, Cooper-DeHoff RM, Pepine CJ, Messerli FH. 2014 Eighth Joint National Committee panel recommendation for blood pressure targets revisited: results from the INVEST study. *J Am Coll Cardiol* 2014; 64: 784–93.
2. Ng M, Fleming T, Robinson M, *et al.* Global, regional, and national prevalence of overweight and obesity in children and adults during 1980–2013: a systematic analysis for the Global Burden of Disease Study 2013. *The Lancet* 2014; 384: 766–81.
3. Singh GM, Danaei G, Farzadfar F, *et al.* The age-specific quantitative effects of metabolic risk factors on cardiovascular diseases and diabetes: a pooled analysis. *PloS One* 2013; 8: e65174.
4. Collaboration APCS, others. Blood pressure and cardiovascular disease in the Asia Pacific region. *J Hypertens* 2003; 21: 707–16.
5. Prospective Studies Collaboration. Age-specific relevance of usual blood pressure to vascular mortality: a meta-analysis of individual data for one million adults in 61 prospective studies. *The Lancet* 2002; 360: 1903–13.
6. Rapsomaniki E, Timmis A, George J, *et al.* Blood pressure and incidence of twelve cardiovascular diseases: lifetime risks, healthy life-years lost, and age-specific associations in 1·25 million people. *Lancet Lond Engl* 2014; 383: 1899–911.

High fasting plasma glucose

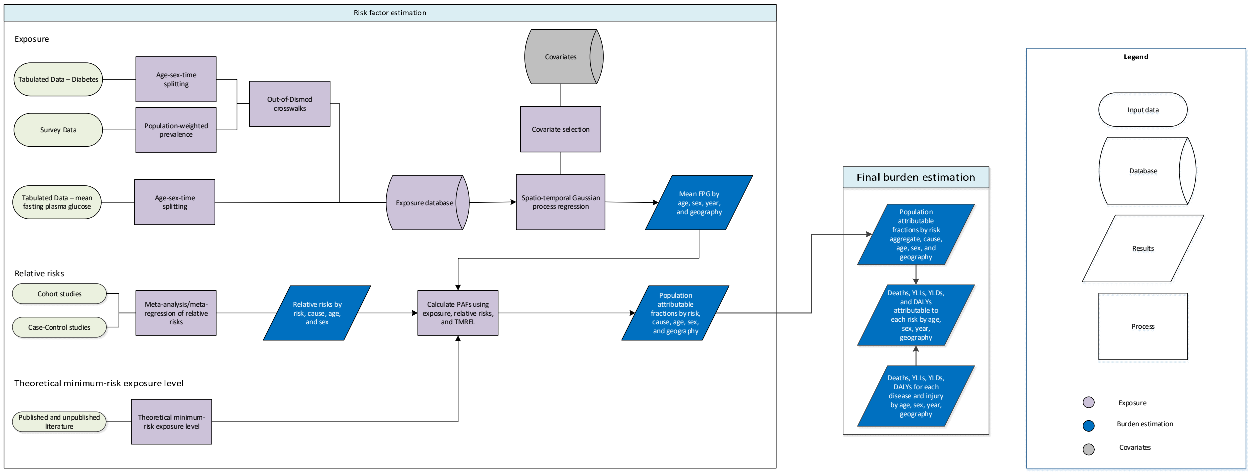

Case definition

High fasting plasma glucose (FPG) is measured as the mean FPG in a population, where FPG is a continuous exposure in units of mmol/L. Since FPG is along a continuum, we define high FPG as any level above the TMREL, which is 4.8-5.4 mmol/L.

Data seeking

Exposure

We conducted a systematic review for FPG and diabetes in GBD 2019. We use all available sources on FPG and prevalence of diabetes in the FPG model.

1. Search terms:

Diabetes Mellitus search string: (diabetes[TI] AND (prevalence[TIAB] OR incidence[TIAB])) OR ('Diabetes Mellitus'[MeSH Terms] AND 'epidemiology'[MeSH Terms]) OR (diabetes[TI] AND 'epidemiology'[MeSH Terms]) NOT gestational[All Fields] NOT ('neoplasms'[MeSH Terms] OR 'neoplasms'[All Fields] OR 'cancer'[All Fields]) NOT ('mice'[MeSH Terms] OR 'mice'[All Fields]) NOT ('schizophrenia'[MeSH Terms] OR 'schizophrenia'[All Fields]) NOT ('emigrants and immigrants'[MeSH Terms] OR ('emigrants'[All Fields] AND 'immigrants'[All Fields]) OR 'emigrants and immigrants'[All Fields] OR 'immigrants'[All Fields]) NOT ('pregnancy'[MeSH Terms] OR 'pregnancy'[All Fields] OR 'gestation'[All Fields]) NOT ('rats'[MeSH Terms] OR 'rats'[All Fields] OR 'rat'[All Fields]) NOT ('kidney'[MeSH Terms] OR 'kidney'[All Fields]) NOT renal[All Fields] NOT ('vitamins'[Pharmacological Action] OR 'vitamins'[MeSH Terms] OR 'vitamins'[All Fields] OR 'vitamin'[All Fields])

And

FPG search string: ((“glucose”[Mesh] OR “hyperglycemia”[Mesh] OR “prediabetic state”[Mesh]) AND "Geographic Locations"[Mesh] NOT "United States"[Mesh]) AND ("humans"[Mesh] AND "adult"[MeSH]) AND ("Data Collection"[Mesh] OR "Health Services Research"[Mesh] OR "Population Surveillance"[Mesh] OR "Vital statistics"[Mesh] OR "Population"[Mesh] OR "Epidemiology"[Mesh] OR surve*[TiAb]) NOT Comment[ptyp] NOT Case Reports[ptyp]) NOT "hospital"[TiAb]

Search date: October 17, 2018. The search took place for the following dates: 10/15/2017-10/16/2018. The number of studies returned was 717, and the number of studies extracted was 36.

Figure 1: PRISMA diagram of data sources used in GBD 2019 high fasting plasma glucose model

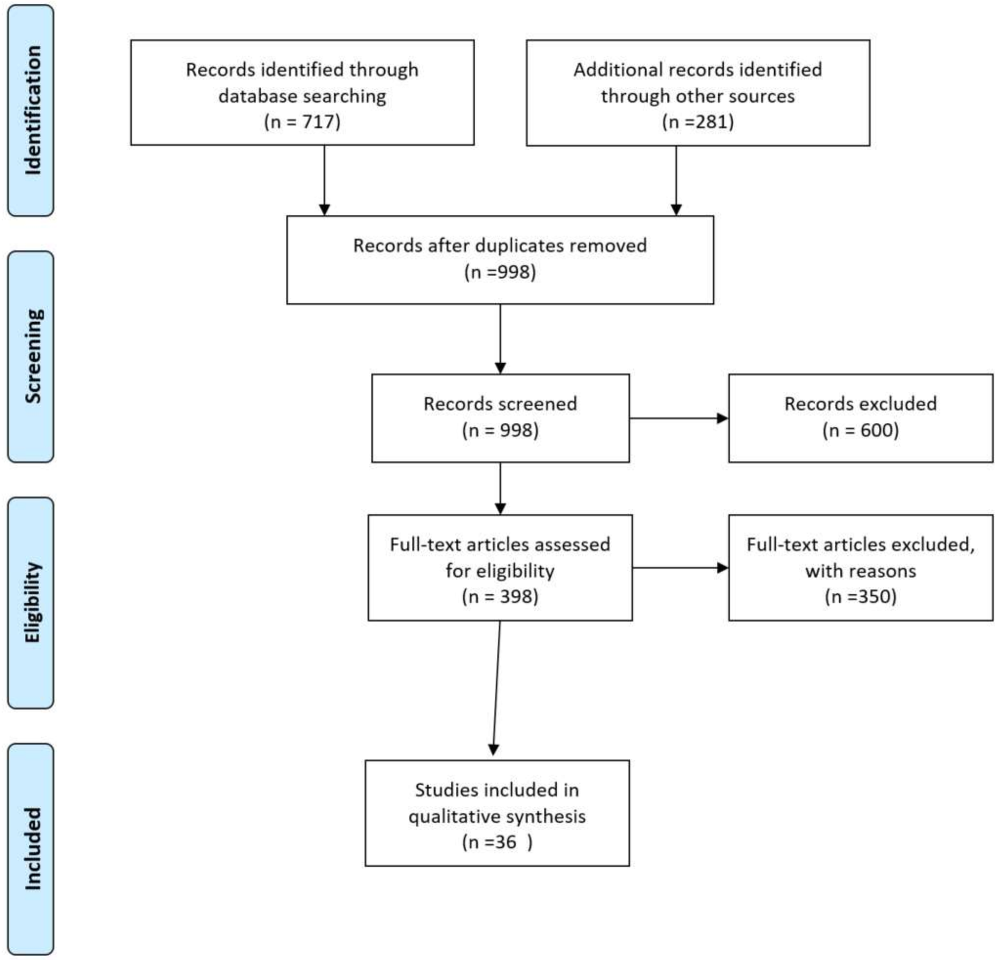

Data inputs

Data inputs come from 3 sources:

- Estimates of mean FPG in a representative population
- Individual-level data of fasting plasma glucose measured from surveys
- Estimates of diabetes prevalence in a representative population

Data sources that did not report mean FPG or prevalence of diabetes are excluded from analysis. When a study reported both mean fasting plasma glucose (FPG) and prevalence of diabetes, we use the mean FPG for exposure estimates. Where possible, individual-level data supersede any data described in a

study. Individual-level data are aggregated to produce estimates for each 5-year age group, sex, location, and year of a survey.

Table 1: Number of sources used in exposure and relative risk models in GBD 2019

Measure	Total sources	Countries with data
Total	549	127
Relative risk	20	-
Exposure	529	127

Data processing

We perform several processing steps to the data in order to address sampling and measurement inconsistencies that will ensure the data are comparable.

1. *Small sample size*
   Estimates in a sex and age group with a sample size <30 persons is considered a small sample size. In order to avoid small sample size problems that may bias estimates, data are collapsed into the next age group in the same study till the sample size reach at least 30 persons. The intent of collapsing the data is to preserve as much granularity between age groups as possible. If the entire study sample consists of <30 persons and did not include a population-weight, the study is excluded from the modelling process.

2. *Crosswalks*
   We predicted mean FPG from diabetes prevalence using an ensemble distribution. We characterized the distribution of FPG using individual-level data. Details on the ensemble distribution can be found elsewhere in the Appendix. Before predicting mean FPG from prevalence of diabetes, we ensured that the prevalence of diabetes was based on the reference case definition: fasting plasma glucose (FPG) >126 mg/dL (7 mmol/L) or on treatment. For more details on how the case-definition crosswalk is conducted, please see the diabetes mellitus appendix in *Global, regional, and national incidence, prevalence, and years lived with disability for 354 diseases and injuries for 195 countries, 1990–2019: a systematic analysis for the Global Burden of Disease Study 2019*.

Exposure modelling

Exposure estimates are produced for every year between 1980 to 2019 for each national and subnational location, sex, and for each 5-year age group starting from 25 years. As in previous rounds of GBD, we used a Spatio-Temporal Gaussian Process Regression (ST-GPR) framework to model the mean fasting plasma glucose at the location-, year-, age-, and sex- level. Updates to the ST-GR modelling framework for GBD 2019 are detailed elsewhere in the Appendix.

Fasting plasma glucose is frequently tested or reported in surveys aiming at assessing the prevalence of diabetes mellitus. In these surveys, the case definition of diabetes may include both a glucose test and questions about treatment for diabetes. People with positive history of diabetes treatment may be excluded from the FPG test. Thus, the mean FPG in these surveys would not represent the mean FPG in the entire population. In this event, we estimated the prevalence of diabetes assuming a definition of

FPG>126 mg/dL (7mmol/L), then crosswalked it to our reference case definition, and then predicted mean FPG.

To inform our estimates in data-sparse countries, we systematically tested a range of covariates and selected age specific prevalence of obesity as a covariate based on direction of the coefficient and significance level.

Mean FPG is estimated using a mixed-effects linear regression, run separately by sex:

$$\text{logit}(\text{FPG}_{c,a,t}) = \beta_0 + \beta_1 p_{\text{overweight}_{c,a,t}} + \sum_{k=2}^{16} \beta_k I_{A[a]} + \alpha_s + \alpha_r + \alpha_c + \epsilon_{c,a,t}$$

where $p_{\text{overweight}_{c,a,t}}$ is the prevalence of overweight, $I_{A[a]}$ is an indicator variable for a fixed effect on a given 5-year age group, and α_s α_r α_c are random effects at the super-region, region, and country level, respectively. The estimates were then propagated through the ST-GPR framework to obtain 1000 draws for each location, year, age, and sex.

Theoretical minimum-risk exposure level

The theoretical minimum-risk exposure level (TMREL) for FPG is 4.8-5.4 mmol/L. This was calculated by taking the person-year weighted average of the levels of FPG that were associated with the lowest risk of mortality in the pooled analyses of prospective cohort studies.[1]

Relative risks

We estimate 15 outcomes due to high fasting plasma glucose (continuous risk) or diabetes (categorical risk).

**Risk**	**Outcome**
Fasting plasma glucose	Ischemic heart disease
Fasting plasma glucose	Ischemic stroke
Fasting plasma glucose	Subarachnoid hemorrhage
Fasting plasma glucose	Intracerebral hemorrhage
Fasting plasma glucose	Peripheral vascular disease
Fasting plasma glucose	Type 1 diabetes
Fasting plasma glucose	Type 2 diabetes
Fasting plasma glucose	Chronic kidney disease due to Type 1 diabetes
Fasting plasma glucose	Chronic kidney disease due to Type 2 diabetes
Diabetes mellitus	Drug-resistant tuberculosis
Diabetes mellitus	Drug-susceptible tuberculosis
Diabetes mellitus	Multidrug-resistant tuberculosis without extensive drug resistance

Diabetes mellitus	Extensively drug-resistant tuberculosis
Diabetes mellitus	Liver cancer due to NASH
Diabetes mellitus	Liver cancer due to other causes
Diabetes mellitus	Pancreatic cancer
Diabetes mellitus	Ovarian cancer
Diabetes mellitus	Colorectal cancer
Diabetes mellitus	Bladder cancer
Diabetes mellitus	Lung cancer
Diabetes mellitus	Breast cancer
Diabetes mellitus	Glaucoma
Diabetes mellitus	Cataracts
Diabetes mellitus	Dementia

Relative risks for High Fasting Plasma Glucose (continuous risk)

After a review of the chronic kidney disease literature, we determined that there is only an attributable risk of chronic kidney disease due to diabetes type 1 and chronic kidney disease due to diabetes type 2 to FPG. Thus, in GBD 2019 we removed chronic kidney disease due to glomerulonephritis, chronic kidney disease due to hypertension, chronic kidney disease due to other causes as an outcome.

Relative risks (RR) were obtained from dose-response meta-analysis of prospective cohort studies. Please see the citation list for a full list of studies that are utilized. For cardiovascular outcomes, we estimated age-specific RRs using DisMod-MR 2.1 with log (RR) as the dependent variable and median age at event as the independent variable with an intercept at age 110. Morbidity and mortality directly caused by diabetes type 1 and diabetes type 2 is considered directly attributable to FPG.

Relative risks for Diabetes mellitus (Categorical risk)

Relative risks were obtained from meta-analysis of cohort studies. Please see the citation list for a full list of studies that are utilized.

References

1. Singh GM, Danaei G, Farzadfar F, *et al.* The age-specific quantitative effects of metabolic risk factors on cardiovascular diseases and diabetes: a pooled analysis. *PloS One* 2013; **8**: e65174.

High LDL cholesterol

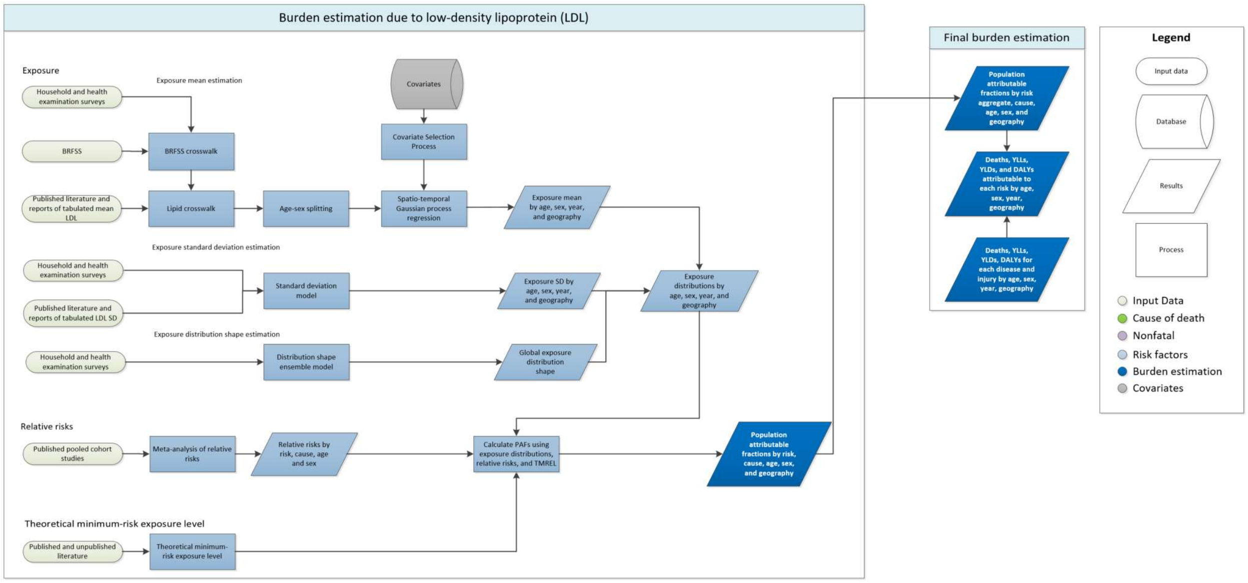

Input data and methodological summary

Exposure

Case definition

In earlier iterations of the GBD study, we estimated burden attributable to total cholesterol. Beginning in GBD 2017, we modelled blood concentration of low-density lipoprotein (LDL) in units of mmol/L.

Input data

We used data on blood levels for low-density lipoprotein, total cholesterol, triglyceride, and high-density lipoprotein from literature and from household survey microdata and reports. We adjusted data for total cholesterol, triglycerides, and high-density lipoprotein using the correction approach described in the Lipid Crosswalk section below. Counts of the data inputs used for GBD 2019 are show in Tables 1 and 2 below. Details of inclusion and exclusion criteria and data processing steps follow.

Table 1: Data inputs for exposure for low-density lipoprotein

Input data	Exposure
Total sources	711
Number of countries with data	145

Table 2: Data inputs for relative risks for low-density lipoprotein

Input data	Relative risk
Source count (total)	1

Inclusion criteria

Studies were included if they were population-based and measured total LDL, total cholesterol (TC), high-density lipoprotein (HDL), and/or triglycerides (TG) were available from blood tests or if LDL was calculated using the Friedewald equation. We assumed the data were representative of the location if the geography or population chosen were not related to the diseases and if it was not an outlier compared to other data in the country or region.

Outliers

Data were utilised in the modelling process unless an assessment of data strongly suggested that the data were biased. A candidate source was excluded if the quality of study did not warrant a valid estimate because of selection (non-representative populations) or if the study did not provide methodological details for evaluation. In a small number of cases, a data point was considered to be an outlier candidate if the level was implausibly low or high based on expert judgement and other country data.

Data extraction

Where possible, individual-level data on LDL estimates were extracted from survey microdata and these were collapsed across demographic groupings to produce mean estimates in the standard GBD five-year age-sex groups. If microdata were unavailable, information from survey reports or from literature were extracted along with any available measure of uncertainty including standard error, uncertainty intervals, and sample size. Standard deviations were also extracted. Where LDL was reported split out by groups other than age, sex, location, and year (eg, by diabetes status), a weighted mean was calculated.

Lipid crosswalk

Total cholesterol consists of three major components: LDL, HDL, and TG. LDL is often calculated for an individual using the Friedewald equation, shown below:

$$LDL = TC - \left(HDL + \frac{TGL}{2.2}\right)$$

We utilised this relationship at the individual level to impute the mean LDL for a study population when only data on TC, HDL, and TGL were available. Because studies report different combinations of TC, HDL, and TGL, we constructed a single regression to utilise all available data to evaluate the relationship between each lipid and LDL at the population level. We used the following regression:

$$LDL = ind_{tc}\beta_1 TC - \left(ind_{hdl}\beta_2 HDL + ind_{tgl}\beta_3 TGL\right) + \sum \alpha_l I_l$$

Where ind_{tc}, ind_{hdl}, and ind_{tgl} are indicator variables for whether data are available for a given lipid, I_l is an indicator variable a given set of available lipids l. α_l is a unique intercept for each set of available lipid combinations. For example, for sources that only reported TC and HDL, $\alpha_{l=TC,HDL}$ should account for the missing lipid data, ie, TGL. The form of this regression allows us to estimate the betas for each lipid using all available data. As a sensitivity analysis, we also ran separate regressions for each set of available lipids and found that the single regression method had much lower root-mean-squared error. A comparison of the observed versus predicted LDL for each set of available lipids is shown in Figure 1. We found almost no relationship between LDL and HDL or TGL when TC was not available, so only studies that reported TC were adjusted to LDL.

Figure 6. Results of the lipid crosswalk using a single regression method

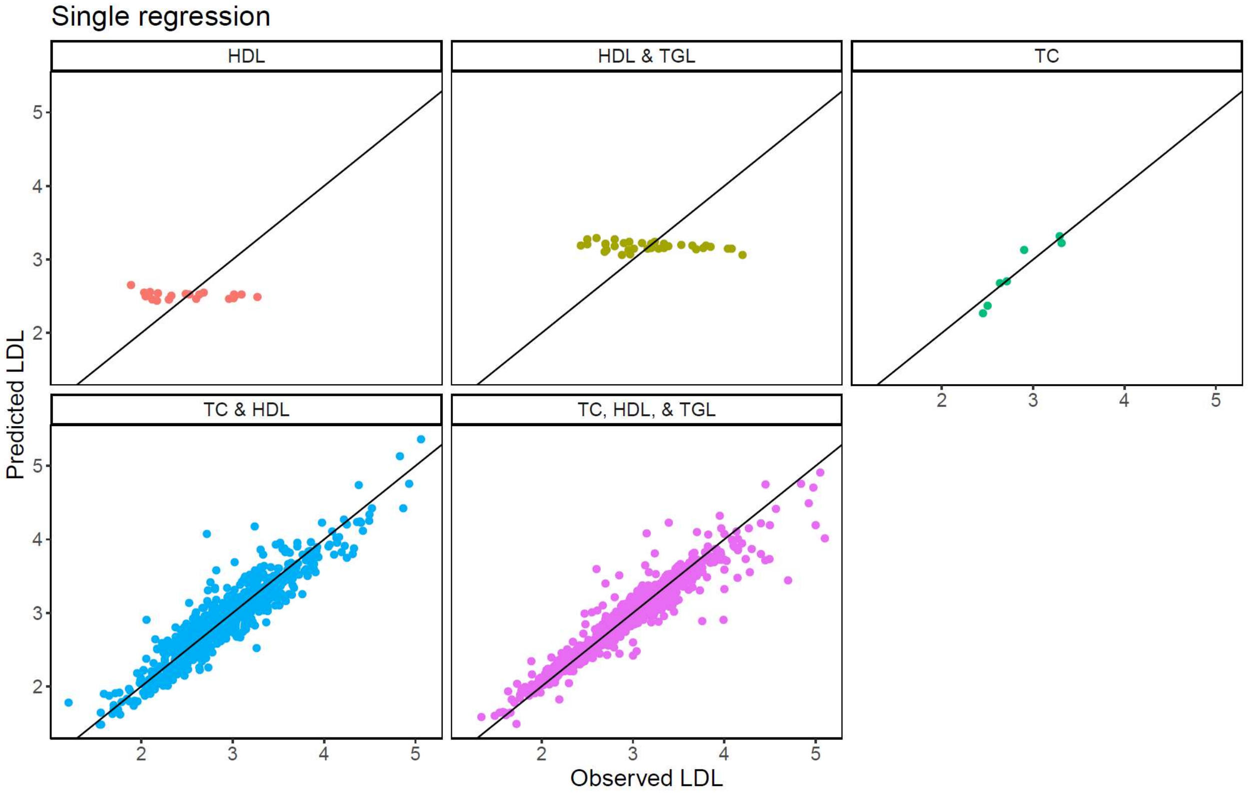

Incorporating United States prevalence data

Survey reports and literature often report information only about the prevalence, but not the level, of hypercholesterolemia in the population studied. These sources were not used to model LDL, with the exception of data from the Behavioral Risk Factors Surveillance System (BRFSS) because of the availability of a similarly structured exam survey covering the identical population (NHANES). BRFSS is a telephone survey conducted in the United States for all counties. It collects self-reported diagnosis of hypercholesterolemia. These self-reported values of prevalence of raised total cholesterol in each age group, sex, US state, and year were used to predict a mean total cholesterol for the same strata with a regression using data from the National Health and Nutrition Examination Survey, a nationally representative health examination survey of the US adult population. The regression was:

$$\mathrm{TC}_{l,a,t,s} = \beta_0 + \beta_1 \mathrm{prev}_{l,a,t,s}$$

where $\mathrm{TC}_{l,a,t,s}$ *is the location, age, time, and sex specific mean total cholesterol and* $\mathrm{prev}_{l,a,t,s}$ is the location, age, time, and sex specific prevalence of raised total cholesterol. The coefficients for both models are reported in Table 1.

Table 3. Coefficients in the sex-specific US states TC prediction models

Term	Male model	Female model
Intercept	4.23	4.36
Prevalence	6.25	5.22

Out of sample RMSE was used to quantify the predictive validity of the model. The regression was repeated 10 times for each sex, each time randomly holding out 20% of the data. The RMSEs from each holdout analysis were averaged to get the average out of sample RMSE. The results of this holdout analysis are reported in Table 2. Total cholesterol estimates were crosswalked to LDL using the lipid crosswalk reported above.

Table 4. Out of sample RMSEs of the sex-specific US states TC prediction models

	Male model	Female model
Out of sample RMSE	0.21 mmol/L	0.20 mmol/L

Age and sex splitting

Prior to modelling, data provided in age groups wider than the GBD five-year age groups were processed using the approach outlined in Ng and colleagues.[2] Briefly, age-sex patterns were identified using person-level microdata (58 sources), and estimate age-sex-specific levels of total cholesterol from aggregated results reported in published literature or survey reports. In order to incorporate uncertainty into this process and borrow strength across age groups when constructing the age-sex pattern, we used a model with auto-regression on the change in mean LDL over age groups:

$$\mu_a = \mu_{a-1} + \omega_a$$
$$\omega_a \sim N(\omega_{a-1}, \tau)$$

Where μ_a is the mean predicted value for age group a, μ_{a-1} is the mean predicted value for the age group previous to age group a, ω_a is the difference in mean between age group *a* and age group *a-1*, ω_{a-1} is the difference between age group *a-1* and age group *a-2*, and τ is a user-input prior on how quickly the mean LDL changes for each unit increase in age. We used a τ of 0.05 mmol/L for this model. Draws of the age-sex pattern were combined with draws of the input data needing to be split in order to calculate the new variance of age-sex-split data points.

Modelling strategy

Exposure estimates were produced from 1980 to 2019 for each national and subnational location, sex, and for each five-year age group starting from 25. As in GBD 2017, we used a spatiotemporal Gaussian process regression (ST-GPR) framework to model the mean LDL at the location-, year-, age-, and sex-level. Details of the ST-GPR method used in GBD 2019 can be found elsewhere in the appendix.

Covariate selection
The first step of the ST-GPR framework requires the creation of a linear model for predicting LDL at the location-, year-, age-, sex- level. Covariates for this model were selected in two stages. First a list of variables with an expected causal relationship with LDL was created based on significant association found within high-quality prospective cohort studies reported in the published scientific literature. The second stage in covariate selection was to test the predictive validity of every possible combination of covariates in the linear model, given the covariates selected above. This was done separately for each sex. Predictive validity was measured with out of sample root-mean-squared error.

In GBD 2016, the linear model with the lowest root-mean-squared error for each sex was then used in the ST-GPR model. Beginning in GBD 2017, we used an ensemble model of the 50 models with the lowest root-mean-squared error for each sex. This allows us to utilise covariate information from many plausible linear mixed-effects models. The 50 models were each used to predict the mean LDL for every age, sex, location, and year, and the inverse-RMSE-weighted average of this set of 50 predictions was used as the linear prior. The relative weight contributed by each covariate is plotted by sex in Figure 2.

Figure 2. Results of the ensemble linear model covariate selection

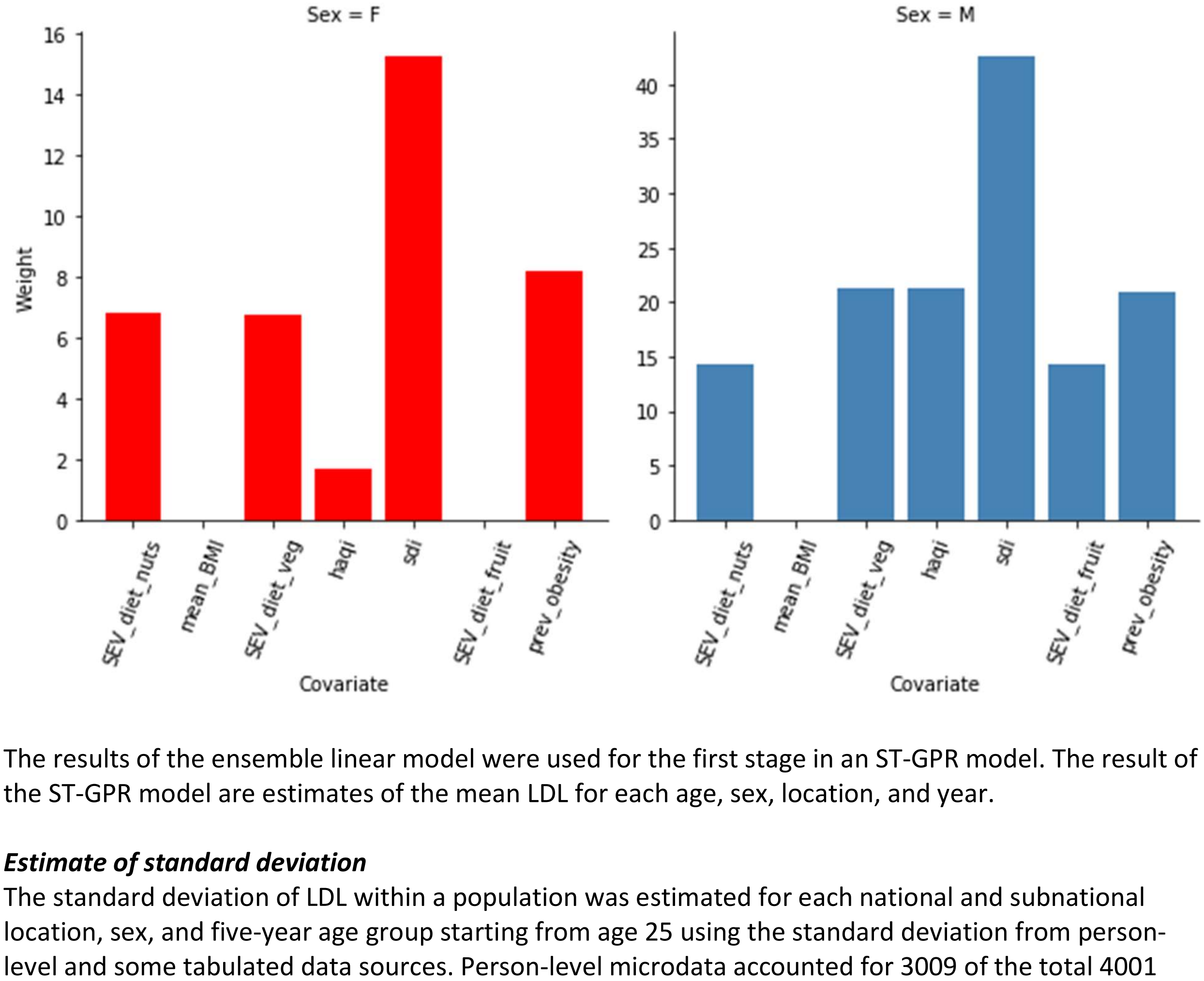

The results of the ensemble linear model were used for the first stage in an ST-GPR model. The result of the ST-GPR model are estimates of the mean LDL for each age, sex, location, and year.

Estimate of standard deviation
The standard deviation of LDL within a population was estimated for each national and subnational location, sex, and five-year age group starting from age 25 using the standard deviation from person-level and some tabulated data sources. Person-level microdata accounted for 3009 of the total 4001 rows of data on standard deviation. The remaining 992 rows came from tabulated data. Tabulated data

were only used to model standard deviation if they were sex-specific and five-year-age-group-specific and reported a population standard deviation LDL. The LDL standard deviation function was estimated using a linear regression:

$$\log\left(\mathrm{SD}_{c,a,t,s}\right) = \beta_0 + \beta_1 \log(\mathrm{mean\_LDL}_{c,a,t,s}) + \beta_4 \mathrm{sex} + \sum_{k=2}^{16} \beta_k \mathrm{I}_{A[a]}$$

where $\mathrm{mean\_LDL}_{c,a,t,s}$ is the country-, age-, time-, and sex-specific mean LDL estimate from ST-GPR, and $\mathrm{I}_{A[a]}$ is a dummy variable for a fixed effect on a given five-year age group.

Distribution shape modelling

The shape of the distribution of LDL was estimated using all available person-level microdata sources, which was a subset of the input data into the modelling process. The distribution shape modelling framework for GBD 2019 is detailed elsewhere in the appendix. Briefly, an ensemble distribution created from a weighted average of distribution families was fit for each individual microdata source, separately by sex. The weights for the distribution families for each individual source were then averaged and weighted to create a global ensemble distribution for each sex.

Theoretical minimum-risk exposure level

For GBD 2017, we reviewed the literature to select a TMREL for LDL. A meta-analysis of randomised trials has shown that outcomes can be improved even at low levels of LDL-cholesterol, below 1.3 mmol/L.[3] Recent studies of PCSK-9 inhibitors support these results.[4] We therefore used a TMREL with a uniform distribution between 0.7 and 1.3 mmol/L; this value remained unchanged for GBD 2019.

Relative risks

After a systematic search, we were unable to find relative risks for LDL that were reported by age and level of LDL. Given this evidence that the relative risks for LDL and TC are very similar[5] and the strong linear correlation between TC and LDL at the individual level, we used relative risks reported for TC to approximate the relative risks for LDL. We used DisMod-MR 2.1 to pool effect sizes from included studies and generate a dose-response curve for each of the outcomes associated with LDL. The tool enabled us to incorporate random effects across studies and include data with different age ranges. RRs were used universally for all countries and produce RRs with uncertainty and covariance across ages, considering the uncertainty of the data points.

As in GBD 2017, RRs for IHD and ischaemic stroke are obtained from meta-regressions of pooled epidemiological studies: the Asia Pacific Cohort Studies Collaboration (APCSC) and the Prospective Studies Collaboration (PSC).[6] RRs for IHD were modelled with log (RR) as the dependent variable and median age at event as the independent variable with an age intercept (RR equals 1) at age 110. For LDL and ischaemic stroke, a similar approach was used, except that there was no age intercept at age 110, due to the fact that there was no statistically significant relationship between LDL and stroke after age 70 with a mean RR less than one. We assumed that there is not a protective effect of LDL and therefore did not include an RR for ages 80+.

References

1. Roth GA, Fihn SD, Mokdad AH, Aekplakorn W, Hasegawa T, Lim SS. High total serum cholesterol, medication coverage and therapeutic control: an analysis of national health examination survey data from eight countries. *Bull World Health Organ* 2011; **89**: 92–101.

2. Ng M, Fleming T, Robinson M, *et al.* Global, regional, and national prevalence of overweight and obesity in children and adults during 1980–2013: a systematic analysis for the Global Burden of Disease Study 2013. *The Lancet* 2014; **384**: 766–81.
3. Boekholdt SM, Hovingh GK, Mora S, *et al.* Very Low Levels of Atherogenic Lipoproteins and the Risk for Cardiovascular EventsA Meta-Analysis of Statin Trials. *J Am Coll Cardiol* 2014; **64**: 485–94.
4. Sabatine MS, Giugliano RP, Keech AC, et al. Evolocumab and Clinical Outcomes in Patients with Cardiovascular Disease. *N Engl J Med*. 2017; **376**:1713-1722.
5. Wilson PF, D'Agostino RB, Levy D, Belanger AM, Silbershatz H, Kannel WB. Prediction of Coronary Heart Disease Using Risk Factor Categories. *Circulation*. 1998; **97**:1837-1847.
6. Singh GM, Danaei G, Farzadfar F, *et al.* The age-specific quantitative effects of metabolic risk factors on cardiovascular diseases and diabetes: a pooled analysis. *PloS One* 2013; **8**: e65174.

High body-mass index

Adult (Ages 20+) High Body-Mass Index: Data and Model Flow Chart

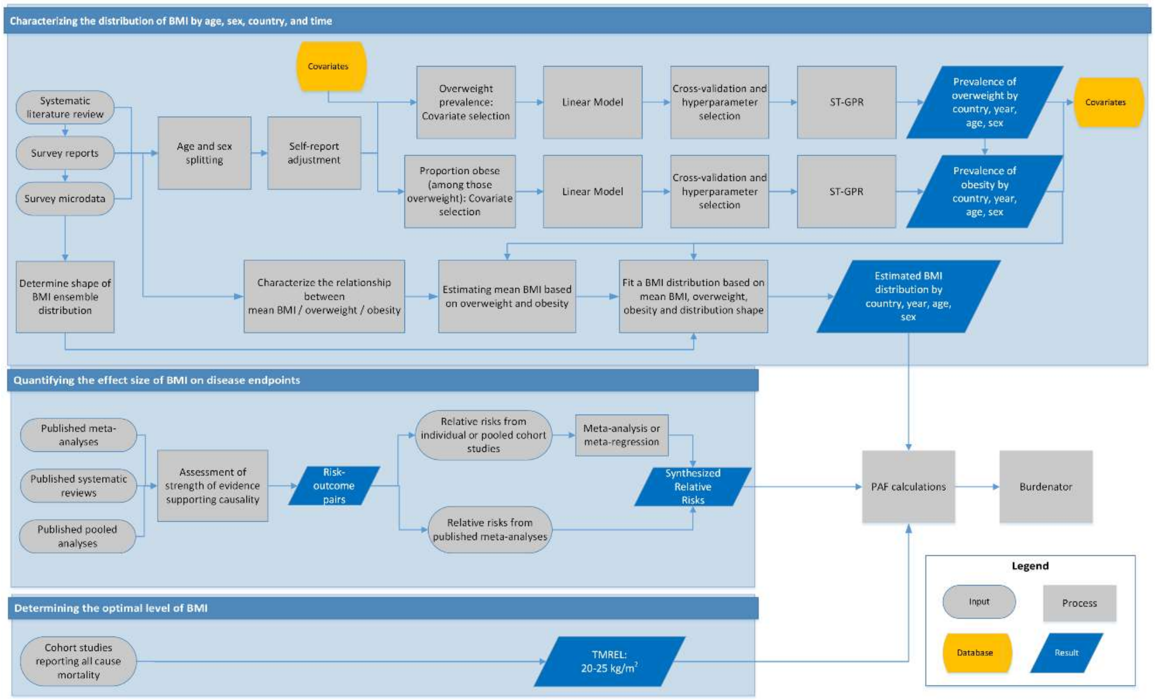

Childhood (Ages 2-19) High Body-Mass Index: Data and Model Flow Chart

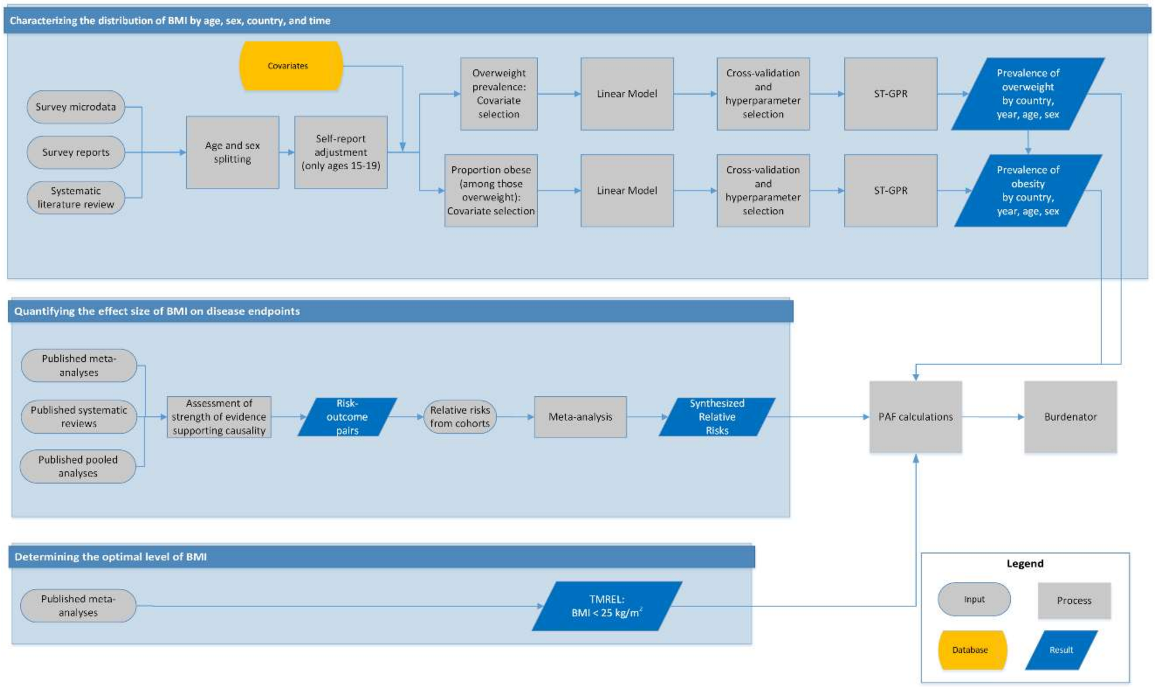

Input data and methodological summary

Case definitions

High body-mass index (BMI) for adults (ages 20+) is defined as BMI greater than 20 to 25 kg/m^2. High BMI for children (ages 1–19) is defined as being overweight or obese based on International Obesity Task Force standards.

Data sources

In GBD 2019, new data were added from sources included in the annual GHDx update of known survey series. We conducted a systematic review in GBD 2017 to identify studies providing nationally or subnationally representative estimates of overweight prevalence, obesity prevalence, or mean body-mass index (BMI). We limited the search to literature published between January 1, 2016, and December 31, 2016, to update the systematic literature search previously performed as part of GBD 2015.

The search for adults was conducted on 4 January 2017, using the following terms:

((("Body Mass Index"[Mesh] OR "Overweight"[Mesh] OR "Obesity"[Mesh]) AND ("Geographic Locations"[Mesh] NOT “United States”[Mesh]) AND ("humans"[Mesh] AND "adult"[MeSH]) AND ("Data Collection"[Mesh] OR "Health Services Research"[Mesh] OR "Population Surveillance"[Mesh] OR "Vital statistics"[Mesh] OR "Population"[Mesh] OR "Epidemiology"[Mesh] OR "surve*"[TiAb]) NOT (Comment[ptyp] OR Case Reports[ptyp] OR "hospital"[TiAb])) AND ("2016/01/01"[Date - Publication] : "2016/12/31"[Date - Publication]))

The search for children was conducted on 4 August 2016, using the following terms:

((("Body Mass Index"[Mesh] OR "Overweight"[Mesh] OR "Obesity"[Mesh]) AND ("Geographic Locations"[Mesh] NOT “United States”[Mesh]) AND ("humans"[Mesh] AND "child"[MeSH]) AND ("Data Collection"[Mesh] OR "Health Services Research"[Mesh] OR "Population Surveillance"[Mesh] OR "Vital statistics"[Mesh] OR "Population"[Mesh] OR "Epidemiology"[Mesh] OR "surve*"[TiAb]) NOT (Comment[ptyp] OR Case Reports[ptyp] OR "hospital"[TiAb])) AND ("2016/01/01"[Date - Publication] : "2016/12/31"[Date - Publication]))

Table 1: Data inputs for exposure for high body-mass index.

Input data	Exposure
Source count (total)	2022
Number of countries with data	190

Table 2: Data inputs for relative risks for high body-mass index.

Input data	Relative risk
Source count (total)	267
Number of countries with data	32

Eligibility criteria

We included representative studies providing data on mean BMI or prevalence of overweight or obesity among adults or children. For adults, studies were included if they defined overweight as BMI≥25 kg/m^2 and obesity as BMI≥30 kg/m^2, or if estimates using those cutoffs could be back-calculated from reported categories. For children (children ages 2–19), studies were included if they used International Obesity Task Force (IOTF) standards to define overweight and obesity thresholds. We only included studies reporting data collected after January 1, 1980. Studies were excluded if they used non-random samples (eg, case-control studies or convenience samples), conducted among specific subpopulations (eg, pregnant women, racial or ethnic minorities, immigrants, or individuals with specific diseases), used alternative methods to assess adiposity (eg, waist-circumference, skin-fold thickness, or hydrodensitometry), had sample sizes of less than 20 per age-sex group, or provided inadequate information on any of the inclusion criteria. We also excluded review articles and non-English-language articles.

Data collection process

Where individual-level survey data were available, we computed mean BMI using weight and height. We then used BMI to determine the prevalence of overweight and obesity. For individuals aged over 19 years, we considered them to be overweight if their BMI was greater than or equal to 25 kg/m^2, and obese if their BMI was greater than or equal to 30 kg/m^2. For individuals aged 2 to 19 years, we used monthly IOTF cutoffs[2] to determine overweight and obese status when age in months was available. When only age in years was available, we used the cutoff for the midpoint of that year. Obese individuals were also considered to be overweight. We excluded studies using the World Health Organization (WHO) standards or country-specific cutoffs to define childhood overweight and obesity. At the individual level, we considered BMI<10 kg/m^2 and BMI>70 kg/m^2 to be biologically implausible and excluded those observations.

The rationale for choosing to use the IOTF cutoffs over the WHO standards has been described elsewhere.[1] Briefly, the IOTF cutoffs provide consistent child-specific standards for ages 2–18 derived from surveys covering multiple countries. By contrast, the WHO growth standards apply to children under age 5, and the WHO growth reference applies to children ages 5–19. The WHO growth reference for children ages 5–19 was derived from United States data, which are less representative than the multinational data used by IOTF. Additionally, the switch between references at age 5 can produce artificial discontinuities. Given that we estimate global childhood overweight and obesity for ages 2–19 (with ages 19 using standard adult cutoffs), the IOTF cutoffs were preferable. Additionally, we found that IOTF cutoffs were more commonly used in scientific literature covering childhood obesity.

From report and literature data, we extracted data on mean BMI, prevalence of overweight, and prevalence of obesity, measures of uncertainty for each, and sample size, by the most granular age and sex groups available. Additionally, we extracted the same study-level covariates as were extracted from microdata (measurement, urbanicity, and representativeness), as well as location and year.

In addition to the primary indicators described above, we extracted relevant survey-design variables, including primary sampling unit, strata, and survey weights, which were used to tabulate individual-level microdata and produce accurate measures of uncertainty. We extracted three study-level covariates: 1) whether height and weight data were measured or self-reported; 2) whether the study was predominantly conducted in an urban area, rural area, or both; and 3) the level of representativeness of the study (national or subnational).

Finally, we extracted relevant demographic indicators, including location, year, age, and sex. We estimated the standard error of the mean from individual-level data, where available, and used the reported standard error of the mean for published data. When multiple data sources were available for the same country, we included all of them in our analysis. If data from the same data source were available in multiple formats such as individual-level data and tabulated data, we used individual-level data.

Modelling strategy

Age and sex splitting

Any report or literature data provided in age groups wider than the standard five-year age groups or as both sexes combined were split using the approach used by Ng and colleagues.[2] Briefly, age-sex patterns were identified using sources with data on multiple age-sex groups and these patterns were applied to split aggregated report and literature data. Uncertainty in the age-sex split was propagated by multiplying the standard error of the data by the square root of the number of splits performed. We did not propagate the uncertainty in the age pattern and sex pattern used to split the data as they seemed to have small effect.

Self-report bias adjustment

We included both measured and self-reported data. We tested for bias in self-report data compared to measured data, which is considered to be the gold-standard. There was no clear direction of bias for children ages 2–14, so for these age groups we only included measured data. For individuals ages 15 and above, we *adjusted self-reported data for overweight prevalence and obesity prevalence. In GBD 2017, the self-report bias adjustment used a nested hierarchical mixed-effects regression model. This approach was updated in GBD 2019 to utilise the power of MR-BRT. For both overweight and obesity, we fit sex-*

specific MR-BRT models on the logit difference between measured and self-reported with a fixed effect on super-region. The bias coefficients derived from these two models are in Table 1 and 2.

Table 1: MR-BRT self-report crosswalk adjustment factors for overweight prevalence

Model	Data input	Reference or alternative case definition	Gamma	Beta coefficient, logit (95% CI)
Females	Measured data	Ref	0.26	---
	Self-reported data (southeast Asia, east Asia, and Oceania)	Alt		-0.53 (-1.03, -0.04)
	Self-reported data (central Europe, eastern Europe, and central Asia)	Alt		-0.20 (-0.69, 0.30)
	Self-reported data (high-income)	Alt		-0.25 (-0.75, 0.24)
	Self-reported data (Latin America and Caribbean)	Alt		-0.19 (-0.69, 0.31)
	Self-report data (north Africa and Middle East)	Alt		-0.38 (-0.89,0.11)
	Self-report data (south Asia)	Alt		0.36 (-0.14, 0.85)
	Self-report data (sub-Saharan Africa)	Alt		-0.26 (-0.76, 0.24)
Males	Measured data	Ref	0.43	---
	Self-reported data (southeast Asia, east Asia, and Oceania)	Alt		-0.36 (-1.17, 0.50)
	Self-reported data (central Europe, eastern Europe, and central Asia)	Alt		-0.03 (-0.84, 0.82)
	Self-reported data (high-income)	Alt		0.05 (-0.77, 0.87)
	Self-reported data (Latin America and Caribbean)	Alt		-0.02 (-0.84, 0.81)
	Self-report data (north Africa and Middle East)	Alt		-0.21 (-1.04, 0.61)
	Self-report data (south Asia)	Alt		0.53 (-0.28, 1.37)
	Self-report data (sub-Saharan Africa)	Alt		-0.27 (-1.09, 0.55)

Table 2: MR-BRT self-report crosswalk adjustment factors for obesity prevalence

Model	Data input	Reference or alternative case definition	Gamma	Beta coefficient, logit (95% CI)
Females	Measured data	Ref	0.38	---
	Self-reported data (southeast Asia, east Asia, and Oceania)	Alt		-0.11 (-0.86, 0.64)

	Self-reported data (central Europe, eastern Europe, and central Asia)	Alt		-0.95 (-1.70, -0.19)
	Self-reported data (high-income)	Alt		-0.42 (-1.16, 0.34)
	Self-reported data (Latin America and Caribbean)	Alt		-0.41 (-1.16, 0.34)
	Self-report data (north Africa and Middle East)	Alt		-0.48 (-1.23, 0.27)
	Self-report data (south Asia)	Alt		0.50 (-0.25, 1.26)
	Self-report data (sub-Saharan Africa)	Alt		-0.41 (-1.16, 0.34)
Males	Measured data	Ref	0.74	
	Self-reported data (southeast Asia, east Asia, and Oceania)	Alt		0.04 (-1.41, 1.53)
	Self-reported data (central Europe, eastern Europe, and central Asia)	Alt		-0.79 (-2.25, 0.71)
	Self-reported data (high-income)	Alt		-0.13 (-1.58, 1.40)
	Self-reported data (Latin America and Caribbean)	Alt		-0.26 (-1.70, 1.21)
	Self-report data (north Africa and Middle East)	Alt		-0.33 (-1.77, 1.16)
	Self-report data (south Asia)	Alt		0.66 (-0.78, 2.15)
	Self-report data (sub-Saharan Africa)	Alt		-0.41 (-1.86, 1.08)

Prevalence estimation for overweight and obesity

After adjusting for self-report bias and splitting aggregated data into five-year age-sex groups, we used spatiotemporal Gaussian process regression (ST-GPR) to estimate the prevalence of overweight and obesity. This modelling approach has been described in detail elsewhere.

The linear model, which when added to the smoothed residuals forms the mean prior for GPR is as follows:

$$\mathrm{logit(overweight)}_{c,a,t} = \beta_0 + \beta_1 \mathrm{energy}_{c,t} + \beta_2 \mathrm{SDI}_{c,t} + \beta_3 \mathrm{vehicles}_{c,t} + \beta_4 \mathrm{agriculture}_{c,t} + \sum_{k=5}^{21} \beta_k I_{A[a]} + \alpha_s + \alpha_r + \alpha_c$$

$$\mathrm{logit(obesity/overweight)}_{c,a,t} = \beta_0 + \beta_1 \mathrm{energy}_{c,t} + \beta_2 \mathrm{SDI}_{c,t} + \beta_3 \mathrm{vehicles}_{c,t} + \sum_{k=4}^{21} \beta_k I_{A[a]} + \alpha_s + \alpha_r + \alpha_c$$

where energy is ten-year lag-distributed energy consumption per capita, SDI is a composite index of development including lag-distributed income per capita, education, and fertility, vehicles is is the number of two- or four-wheel vehicles per capita, and agriculture is the proportion of the population working in agriculture. $I_{A[a]}$ is a dummy variable indicating specific age group A that the prevalence point captures, and α_s, α_r, and α_c are super-region, region, and country random intercepts, respectively. Random effects were used in model fitting but were not used in prediction.

We tested all combinations of the following covariates to see which performed best in terms of in-sample AIC for the overweight linear model and the obesity as a proportion of overweight linear model: ten-year lag-distributed energy per capita, proportion of the population living in urban areas, SDI, lag-distributed income per capita, educational attainment (years) per capita, proportion of the population

working in agriculture, grams of sugar adjusted for energy per capita, grams of sugar not adjusted for energy per capita, and the number of two- or four-wheeled vehicles per capita. We selected these candidate covariates based on theory as well as reviewing covariates used in other publications. The final linear model was selected based on 1) if the direction of covariates matched what is expected from theory, 2) all the included covariates were significant, and 3) minimising in-sample AIC. The covariate selection process was performed using the dredge package in R.

Estimating mean BMI

To estimate the mean BMI for adults in each country, age, sex, and time period 1980–2019, we first used the following nested hierarchical mixed-effects model, fit using restricted maximum likelihood on data from sources containing estimates of all three indicators (prevalence of overweight, prevalence of obesity, and mean BMI), in order to characterise the relationship between overweight, obesity, and mean BMI:

$$\log(\mathrm{BMI}_{c,a,s,t}) = \beta_0 + \beta_1 \mathrm{ow}_{c,a,s,t} + \beta_2 \mathrm{ob}_{c,a,s,t} + \beta_3 \mathrm{sex} + \sum_{k=4}^{20} \beta_k I_{A[a]} + \alpha_s(1 + \mathrm{ow}_{c,a,s,t} + \mathrm{ob}_{c,a,s,t}) + \alpha_r(1 + \mathrm{ow}_{c,a,s,t} + \mathrm{ob}_{c,a,s,t}) + \alpha_c(1 + \mathrm{ow}_{c,a,s,t} + \mathrm{ob}_{c,a,s,t}) + \epsilon_{c,a,s,t}$$

where $\mathrm{ow}_{c,a,s,t}$ *is the prevalence of overweight in country c, age a, sex s, and year t,* $\mathrm{ob}_{c,a,s,t}$ *is the prevalence of obesity in country c, age a, sex s, and year t,* sex *is a fixed effect on sex,* $I_{A[a]}$ *is an indicator variable for age, and* α_s, α_r, *and* α_c *are random effects at the super-region, region, and country, respectively. The model was run in Stata 13.*

We applied 1000 draws of the regression coefficients to the 1000 draws of overweight prevalence and obesity prevalence produced through ST-GPR to estimate 1000 draws of mean BMI for each country, year, age, and sex. This approach ensured that overweight prevalence, obesity prevalence, and mean BMI were correlated at the draw level and uncertainty was propagated.

Estimating BMI distribution

We used the ensemble distribution approach described in the manuscript. We fit ensemble weights by source and sex, with source- and sex-specific weights averaged across all sources included to produce the final global weights. The ensemble weights were fit on measured microdata. The final ensemble weights were exponential = 0.002, gamma = 0.028, inverse gamma = 0.085, log-logistic = 0.187, Gumbel = 0.220, Weibull = 0.011, log-normal = 0.058, normal = 0.012, beta = 0.136, mirror gamma = 0.008, and mirror Gumbel = 0.113.

One thousand draws of BMI distributions for each location, year, age group, and sex estimated were produced by fitting an ensemble distribution using 1000 draws of estimated mean BMI, 1000 draws of estimated standard deviation, and the ensemble weights. Estimated standard deviation was produced by optimising a standard deviation to fit estimated overweight prevalence draws and estimated obesity prevalence draws.

Assessment of risk-outcome pairs

Risk-outcome pairs were defined based on strength of available evidence supporting a causal effect. We performed a systematic review of published meta-analyses, pooled analyses, and systematic reviews available through PubMed using the following search string: ("Body Mass Index"[Mesh] OR

"Overweight"[Mesh] OR "Obesity"[Mesh]) AND (Meta-Analysis[ptyp] OR "systematic review"[tiab] OR "pooled analysis"[tiab]). Inclusion criteria are 1) the health outcome is included in GBD, 2) at least one prospective cohort is included, and 3) that the summary effect size is statistically significant. For outcomes meeting inclusion criteria we completed causal criteria tables to evaluate the strength of evidence supporting a causal relationship (see Appendix Table 4). Gallbladder disease, cataract, multiple myeloma, gout, non-Hodgkin lymphoma, asthma, Alzheimer's disease, and atrial fibrillation were added as new outcomes in GBD 2016, resulting in a total of 38 outcomes.

Theoretical minimum risk exposure level

For adults (ages 20+), the theoretical minimum risk exposure level (TMREL) of BMI (20–25 kg/m^2) was determined based on the BMI level that was associated with the lowest risk of all-cause mortality in prospective cohort studies.[3]

For children (ages 2–19), the TMREL is "normal weight," that is, not overweight or obese, based on IOTF cutoffs.

Relative risk

The relative risk per five-unit change in BMI for each disease endpoint was obtained from meta-analyses, and where available, pooled analyses of prospective observational studies. In cases where a relative risk per five-unit change in BMI was not available we computed our own dose-response meta-analysis using two-step generalised least squares for time trends estimation methods.

For childhood outcomes (ages 2–19), we computed categorical relative risks for overweight and obesity using a random effects meta-analysis.

References

1. Cole, TJ, and T Lobstein. "Extended International (IOTF) Body Mass Index Cut-Offs for Thinness, Overweight and Obesity." Pediatric Obesity 2012; 7(4): 284–94.
2. Ng M, Fleming T, Robinson M, et al. Global, regional, and national prevalence of overweight and obesity in children and adults during 1980–2013: a systematic analysis for the Global Burden of Disease Study 2013. The Lancet 2014; 384: 766–81.
3. Angelantonio ED, Bhupathiraju SN, Wormser D, et al. Body-mass index and all-cause mortality: individual-participant-data meta-analysis of 239 prospective studies in four continents. The Lancet 2016; 388: 776–86.

Kidney dysfunction

Kidney dysfunction

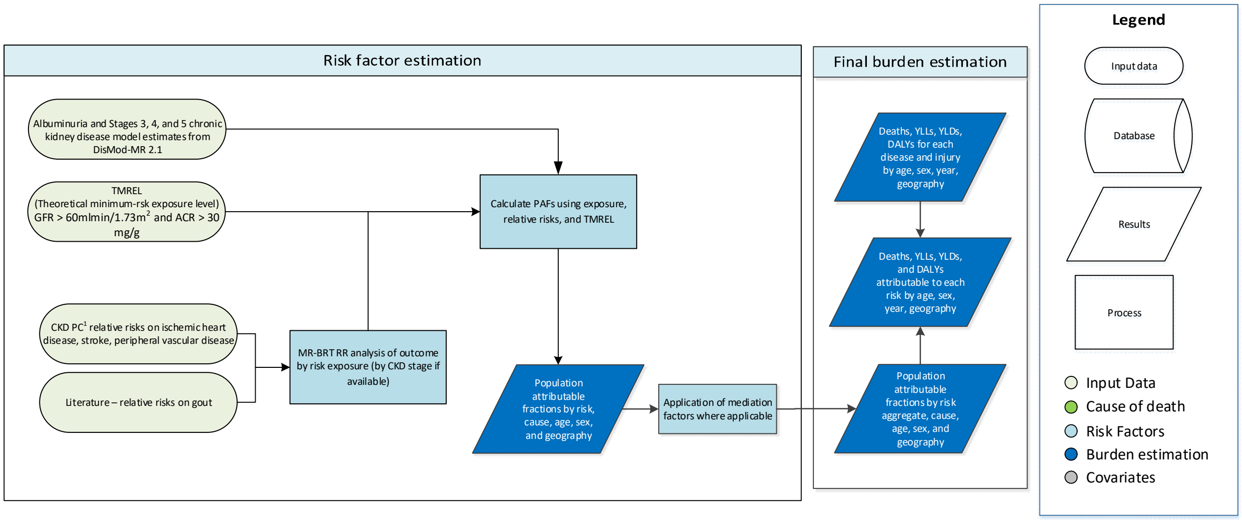

1. The Chronic Kidney Disease Prognosis Consortium is a research group composed of investigators representing cohorts from around the world. Investigators share data for the purpose of collaborative meta-analyses to study prognosis in CKD.

Input data and methodological summary

Exposure

Case definition

The kidney dysfunction risk factor exposure is divided into four categories of renal function defined by urinary albumin to creatinine ratio (ACR) and estimated glomerular filtration rate (eGFR):

- Albuminuria with preserved eGFR (ACR >30 mg/g & eGFR >=60 ml/min/1.73m^2); this corresponds to stages 1 and 2 chronic kidney disease (CKD) in the Kidney Disease Improving Global Outcomes (KDIGO) classification
- CKD stage 3 (eGFR of 30-59 ml/min/1.73m^2);
- CKD stage 4 (eGFR of 15-29 ml/min/1.73m^2); and
- CKD stage 5 (eGFR <15ml/min/1.73m^2, not (yet) on renal replacement therapy).

The modelling of renal function prevalence estimates is described in detail in the CKD section of the appendix to the GBD 2019 disease and injury paper.

Theoretical minimum-risk exposure level

The theoretical minimum-risk exposure level is ACR 30 mg/g or less and eGFR greater than 60ml/min/1.73m^2. An ACR above 30 mg/g and eGFR below 60ml/min/1.73m^2 have been demonstrated in the literature to be the thresholds at which increased cardiovascular and gout events occur secondary to kidney dysfunction.(1-10)

Input data

The last systematic review of prevalence of low glomerular filtration rate was conducted for GBD 2016, updating searches done in GBD 2015, GBD 2013, and GBD 2010. Exclusion criteria included surveys that were not population-representative and studies not reporting on CKD by stage.

Data sources for kidney dysfunction:

Input data	Exposure
Source count (total)	98
Number of countries with data	35

Input data	Relative risk
Source count (total)	9

Modelling strategy

We model the proportion of cardiovascular and musculoskeletal diseases attributable to kidney dysfunction. This is performed by 1) running DisMod-MR 2.1 models to estimate the prevalence of albuminuria, stage 3 CKD, stage 4 CKD, and stage 5 CKD; 2) estimate relative risks from available data on cardiovascular outcomes and gout; 3) calculate the population attributable fraction of those outcomes to IKF.

The prevalence of exposure to albuminuria and CKD were obtained from the GBD 2019 non-fatal burden of disease analysis.

Data on relative risks were contributed by the Chronic Kidney Disease Prognosis Consortium (CKD-PC). The Chronic Kidney Disease Prognosis Consortium is a research group composed of investigators representing cohorts from around the world. Investigators share data for the purpose of collaborative meta-analyses to study prognosis in CKD.

Relative risks

We estimate burden attributable to kidney dysfunction for cardiovascular diseases, chronic kidney diseases, and gout.

In GBD 2017, we relied on a pooled cohort analysis of six cohort studies from the CKD-PC. For GBD 2019, in collaboration with CKD-PC, we got data on 38 new cohorts and continued to use the original from the previous analysis. We ran these new data through MR-BRT meta-regression to determine the relationship between age and outcomes based on exposure to IKF. Estimates were nested within cohorts. A three-degree spline was placed on age with decreasing monotonicity. All relative risk estimates for stroke and ischaemic heart disease above age 85 were set equal to the risk at age 85 to control for lack of data in older age groups. Gout currently uses GBD 2017 estimations of relative risk.

We ran some sensitivity analyses with and without controlling for blood pressure. This is because IKF increases the risk of cardiovascular diseases directly, as well as through blood pressure. We wanted to understand how estimates of risk would differ. Generally, the relative risk of cardiovascular disease was lower when controlling for blood pressure. We decided to go with this lower risk that controlled for hypertension for a more conservative estimate.

Relative risk plots

The following plot shows the relative risks for heart disease and stroke by each stage of CKD. As expected, stage 5 and stage 4 CKD have higher risks overall. Risks is also higher at younger ages and lower at the oldest age, likely reflecting competing risk factors. While the risks themselves dip below

zero at the oldest age, we believe this is merely a function of lack of data above age 85. Because of this, our estimates for relative risk above age 85 take the estimate at age 85.

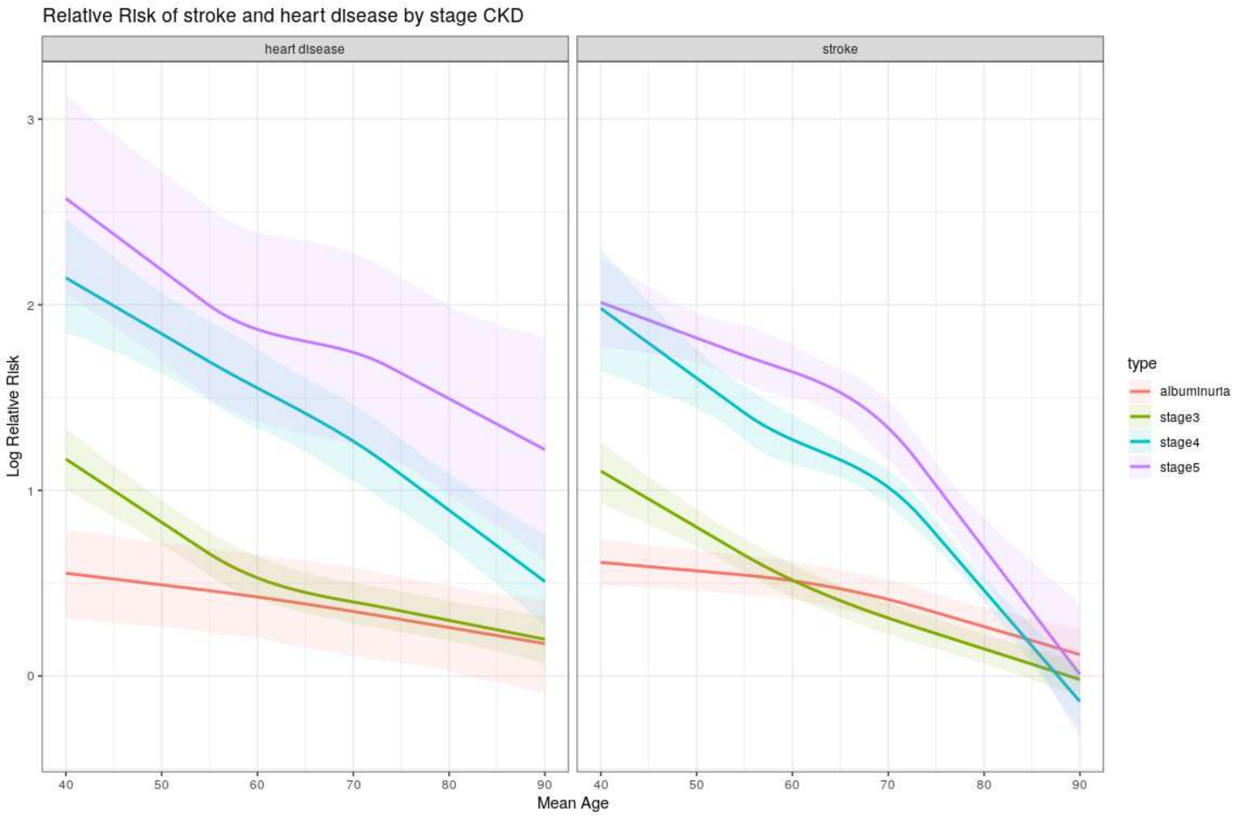

We also include two forest plots to show the distribution of risk estimates for heart disease and stroke across our studies. In general, we see an expected pattern, with earlier stages of CKD with lower risks.

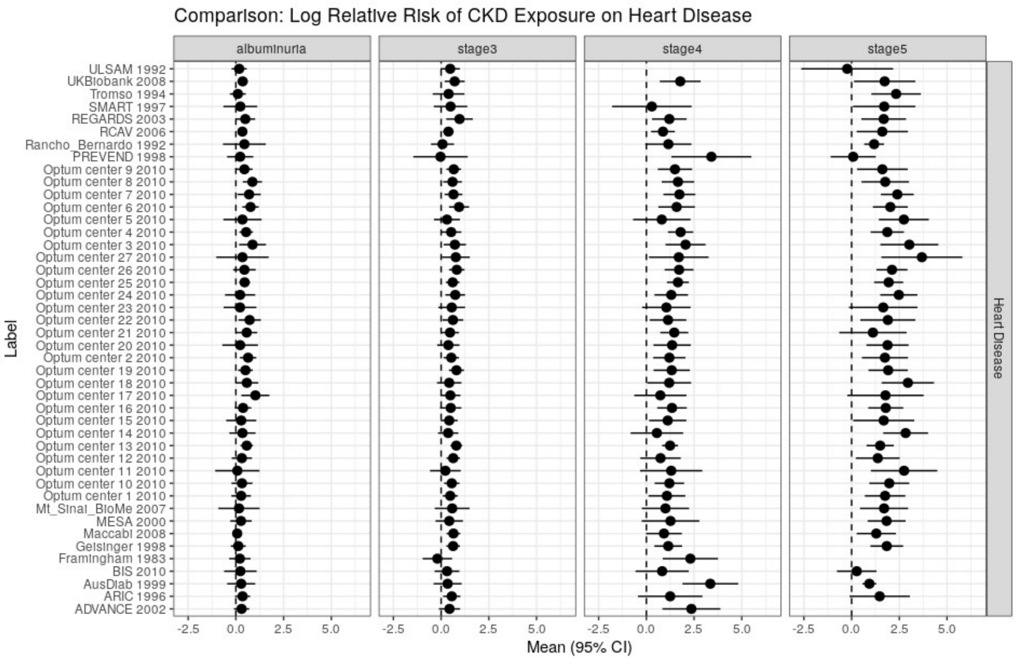

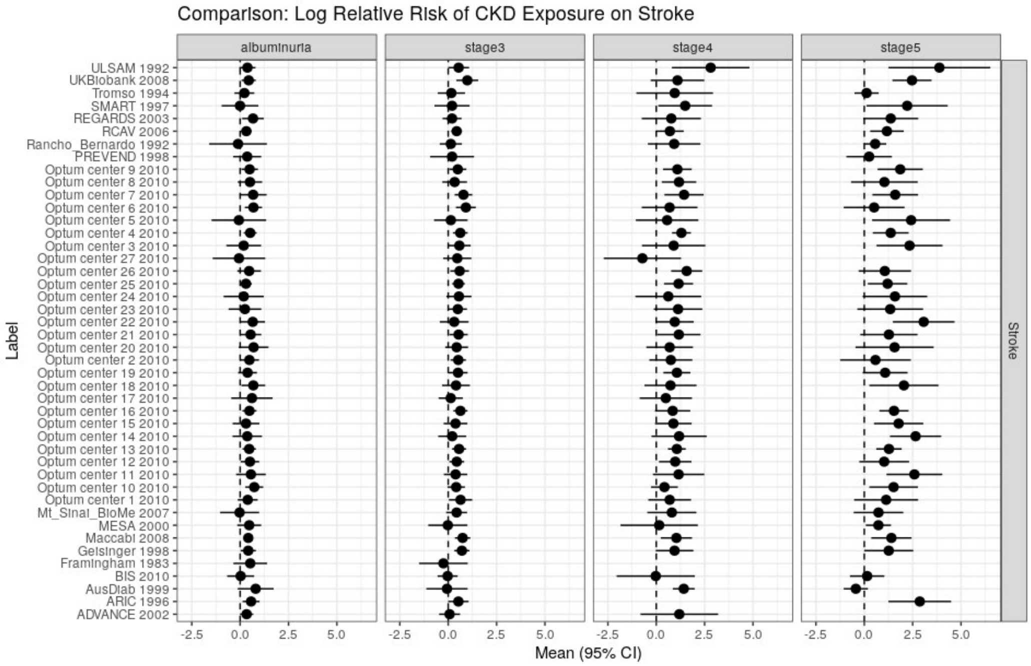

Population attributable fraction

We calculated the cardiovascular and gout fatal and non-fatal burden attributable to the categorical exposure to kidney dysfunction using the following equation:

$$PAF = \frac{\sum_{i=1}^{n} P_i(RR_i - 1)}{\sum_{i=1}^{n} P_i(RR_i - 1) + 1}$$

Equation 1. PAF based on categorical exposure

where RR_i is the relative risk for exposure level *i*, P_i is the proportion of the population in that exposure category, and *n* is the number of exposure categories.(11)

Primary changes between GBD 2017 and GBD 2019

The following are the main changes in the GBD 2019 modelling strategy compared to GBD 2017:

1. In GBD 2019, we used MR-BRT to run a nested meta-regression analysis on the within-study sex ratios to estimate a pooled sex ratio with 95% confidence intervals. In GBD 2017, this was estimated in DisMod-MR 2.1.
2. In GBD 2019, we used MR-BRT to make bias adjustments for data with alternative case definitions. CKD uses CKD-Epi as the reference definition. Alternative equations include the Cockcroft-Gualt and Modification of Diet in Renal Disease equations. MR-BRT models have larger confidence intervals due to taking into account study variance across all input data. In GBD 2017, these adjustments were made in DisMod-MR 2.1. The values of these adjustments are in the table below:

MR-BRT bias adjustment factors

Data input	Status	*Gamma*	*Beta coefficient, logit (95% CI)*	*Adjustment factor**
CKD-EPI	Ref	---	---	---
Stage III CG	Alt	*0.25*	0.24 (-0.28 to 0.76)	0.56 (0.43–0.68)
Stage III MDRD	Alt	*0.03*	0.49 (0.34–0.64)	0.62 (0.58–0.66)
Stage IV CG	Alt	*0*	0.09 (-0.05 to 0.24)	0.52 (0.49–0.56)
Stage IV MDRD	Alt	*0*	-0.07 (-0.19 to 0.04)	0.48 (0.45–0.51)
Stage V CG	Alt	*0*	-0.18 (-0.45 to 0.09)	0.45 (0.39–0.52)
Stage V MDRD	Alt	*0*	-0.06 (-0.28 to 0.18)	0.49 (0.43–0.54)
Stage III-V CG	Alt	*0.26*	0.23 (-0.29 to 0.75)	0.56 (0.43–0.68)
Stage III-V MDRD	Alt	*0.03*	0.47 (0.32–0.62)	0.62 (0.58–0.65)

3. In GBD 2017, the RRs were estimated via a pooled cohort meta-regression conducted in R using the metafor package. In GBD 2019, we made use of MR-BRT to run a nested meta-regression analysis that allowed more flexibility in the estimation process.

References

1. Go AS, Chertow GM, Fan D, McCulloch CE, Hsu CY. Chronic kidney disease and the risks of death, cardiovascular events, and hospitalization. N Engl J Med. 2004;351(13):1296-305.

2. Ninomiya T, Kiyohara Y, Kubo M, Tanizaki Y, Doi Y, Okubo K, et al. Chronic kidney disease and cardiovascular disease in a general Japanese population: the Hisayama Study. Kidney international. 2005;68(1):228-36.
3. Shara NM, Wang H, Mete M, Al-Balha YR, Azalddin N, Lee ET, et al. Estimated GFR and incident cardiovascular disease events in American Indians: the Strong Heart Study. American journal of kidney diseases : the official journal of the National Kidney Foundation. 2012;60(5):795-803.
4. Mann JF, Gerstein HC, Pogue J, Bosch J, Yusuf S. Renal insufficiency as a predictor of cardiovascular outcomes and the impact of ramipril: the HOPE randomized trial. Annals of internal medicine. 2001;134(8):629-36.
5. Chronic Kidney Disease Prognosis C, Matsushita K, van der Velde M, Astor BC, Woodward M, Levey AS, et al. Association of estimated glomerular filtration rate and albuminuria with all-cause and cardiovascular mortality in general population cohorts: a collaborative meta-analysis. Lancet. 2010;375(9731):2073-81.
6. De Graauw J, Chonchol M, Poppert H, Etgen T, Sander D. Relationship between kidney function and risk of asymptomatic peripheral arterial disease in elderly subjects. Nephrology, dialysis, transplantation : official publication of the European Dialysis and Transplant Association - European Renal Association. 2011;26(3):927-32.
7. Wattanakit K, Folsom AR, Selvin E, Coresh J, Hirsch AT, Weatherley BD. Kidney function and risk of peripheral arterial disease: results from the Atherosclerosis Risk in Communities (ARIC) Study. Journal of the American Society of Nephrology : JASN. 2007;18(2):629-36.
8. O'Hare AM, Vittinghoff E, Hsia J, Shlipak MG. Renal insufficiency and the risk of lower extremity peripheral arterial disease: results from the Heart and Estrogen/Progestin Replacement Study (HERS). Journal of the American Society of Nephrology : JASN. 2004;15(4):1046-51.
9. Manjunath G, Tighiouart H, Coresh J, Macleod B, Salem DN, Griffith JL, et al. Level of kidney function as a risk factor for cardiovascular outcomes in the elderly. Kidney international. 2003;63(3):1121-9.
10. Manjunath G, Tighiouart H, Ibrahim H, MacLeod B, Salem DN, Griffith JL, et al. Level of kidney function as a risk factor for atherosclerotic cardiovascular outcomes in the community. Journal of the American College of Cardiology. 2003;41(1):47-55.
11. Miettinen OS. Proportion of disease caused or prevented by a given exposure, trait or intervention. American journal of epidemiology. 1974;99(5):325-32.

Ambient particulate matter pollution

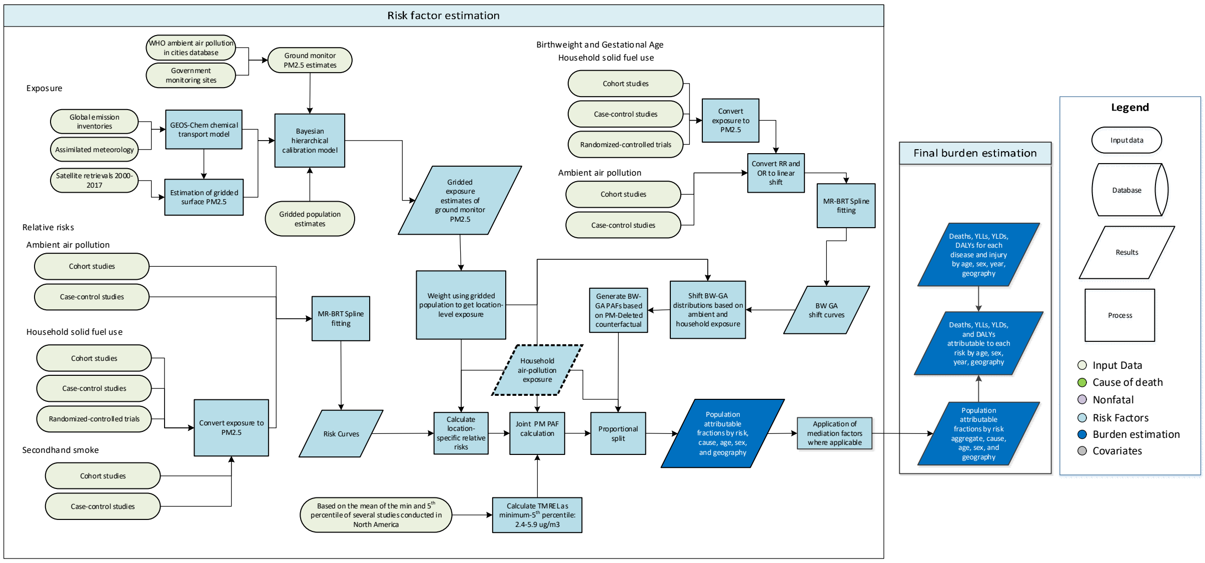

Input data and modelling strategy

Exposure

Definition

Exposure to ambient particulate matter pollution is defined as the population-weighted annual average mass concentration of particles with an aerodynamic diameter less than 2.5 micrometers ($PM_{2.5}$) in a cubic meter of air. This measurement is reported in µg/m^3.

Input data

The data used to estimate exposure to ambient particulate matter pollution comes from multiple sources, including satellite observations of aerosols in the atmosphere, ground measurements, chemical transport model simulations, population estimates, and land-use data. Table 1 summarizes exposure input data.

Table 1: Exposure Input Data

Input data	Exposure
Source count (total)	663
Number of countries with data	114

The following details the updates in methodology and input data used in GBD 2019.

$PM_{2.5}$ ground measurement database

Ground measurements used for GBD 2019 include updated measurements from sites included in 2017 and additional measurements from new locations. New and up-to-date data (mainly from the USA,

Canada, EU, Bangladesh, China and USA embassies and consulates), were added to the data from the 2018 update of the WHO Global Ambient Air Quality Database used in GBD 2017. The updated data included measurements of concentrations of PM_{10} and $PM_{2.5}$ from 10,408 ground monitors from 116 countries from 2010 to 2017. The majority of measurements were recorded in 2016 and 2017 (as there is a lag in reporting measurements, few data from 2018 or newer were available). Annual averages were excluded if they were based on less than 75% coverage within a year. If information on coverage was not available, then data were included unless there were already sufficient data within the same country (monitor density greater than 0.1).

For locations measuring only PM_{10}, $PM_{2.5}$ measurements were estimated from PM_{10}. This was performed using a hierarchy of conversion factors ($PM_{2.5}/PM_{10}$ ratios): (i) for any location a 'local' conversation factor was used, constructed as the ratio of the average measurements (of $PM_{2.5}$ and PM_{10}) from within 50km of the location of the PM_{10} measurement, and within the same country, if such measurements were available; (ii) if there was not sufficient local information to construct a conversion factor then a country-wide conversion factor was used; and (iii) if there was no appropriate information within a country, then a regional factor was used. In each case, to avoid the possible effects of outliers in the measured data (both $PM_{2.5}$ and PM_{10}), extreme values of the ratios were excluded (defined as being greater/lesser than the 95% and 5% quantiles of the empirical distributions of conversion factors). As with GBD 2013, 2015, 2016, and 2017 databases, in addition to values of $PM_{2.5}$ and whether they were direct measurement or converted from PM_{10}, the database also included additional information, where available, related to the ground measurements such as monitor geo-coordinates and monitor site type.

Satellite-based estimates

The global geophysical $PM_{2.5}$ estimates for the years 2000–2017 are from Hammer and colleagues Version V4.GL.03.NoGWR used at 0.1°x0.1° resolution (~11 x 11 km resolution at the equator).[1] The method is based on the algorithms of van Donkelaar and colleagues (2016) as used in GBD 2017,[2] with updated satellite retrievals, chemical transport modelling, and ground-based monitoring. The algorithm uses aerosol optical depth (AOD) from several updated satellite products (MAIAC, MODIS C6.1, and MISR v23), including finer resolution, increased global coverage, and improved long-term stability. Ground-based observations from a global sunphotometer network (AERONET version 3) are used to combine different AOD information sources. This is the first time that data from MAIAC at 1 km resolution was used to estimate $PM_{2.5}$ at the global scale. The GEOS-Chem chemical transport model with updated algorithms was used for geophysical relationships between surface $PM_{2.5}$ and AOD. Updates to the GEOS-Chem simulation included improved representation of mineral dust and secondary organic aerosol, as well as updated emission inventories. The resultant geophysical $PM_{2.5}$ estimates are highly consistent with ground monitors worldwide (R^2=0.81, slope = 1.03, n = 2541).

Population data

A comprehensive set of population data, adjusted to match UN2015 Population Prospectus, on a high-resolution grid was obtained from the Gridded Population of the World (GPW) database. Estimates for 2000, 2005, 2010, 2015, and 2020 were available from GPW version 4, with estimates for 1990 and 1995 obtained from the GPW version 3. These data are provided on a 0.0083°× 0.0083° resolution. Aggregation to each 0.1°×0.1° grid cell was accomplished by summing the central 12 × 12 population

cells. Populations estimates for 2001–2004, 2006–2009, 2011–2014 and 2016–2019 were obtained by interpolation using natural splines with knots placed at 2000, 2005, 2010, 2015, and 2020. This was performed for each grid cell.

Chemical transport model simulations
Estimates of the sum of particulate sulfate, nitrate, ammonium, and organic carbon and the compositional concentrations of mineral dust simulated using the GEOS Chem chemical transport model, and a measure combining elevation and the distance to the nearest urban land surface (as described in van Donkelaar and colleagues 2016[2] and Hammer and colleagues (submitted))[1] were available for 2000–2017 for each 0.1°×0.1° grid cell.

Modelling strategy
The following is a summary of the modelling approach, known as the Data Integration Model for Air Quality (DIMAQ) used in GBD 2015, 2016, 2017, and now in GBD 2019.[3,4]

Before the implementation of DIMAQ (ie, in GBD 2010 and GBD 2013), exposure estimates were obtained using a single global function to calibrate available ground measurements to a "fused" estimate of $PM_{2.5}$; the mean of satellite-based estimates and those from the TM5 chemical transport model, calculated for each 0.1°×0.1° grid cell. This was recognised to represent a tradeoff between accuracy and computational efficiency when utilising all the available data sources. In particular, the GBD 2013 exposure estimates were known to underestimate ground measurements in specific locations (see discussion in Brauer and colleagues, 2015).[5] This underestimation was largely due to the use of a single, global calibration function, whereas in reality the relationship between ground measurements and other variables will vary spatially.

In GBD 2015 and GBD 2016, coefficients in the calibration model were estimated for each country. Where data were insufficient within a country, information can be "borrowed" from a higher aggregation (region) and, if enough information is still not available, from an even higher level (super-region). Individual country-level estimates were therefore based on a combination of information from the country, its region, and its super-region. This was implemented within a Bayesian hierarchical modelling (BHM) framework. BHMs provide an extremely useful and flexible framework in which to model complex relationships and dependencies in data. Uncertainty can also be propagated through the model, allowing uncertainty arising from different components, both data sources and models, to be incorporated within estimates of uncertainty associated with the final estimates. The results of the modelling comprise a posterior distribution for each grid cell, rather than just a single point estimate, allowing a variety of summaries to be calculated. The primary outputs here are the median and 95% credible intervals for each grid cell. Based on the availability of ground measurement data, modelling and evaluation were focused on the year 2016.

The model used in GBD 2017 and GBD 2019 also included within-country calibration variation.[6] The model used for GBD 2019, henceforth referred to as DIMAQ2, provides a number of substantial improvements over the initial formulation of DIMAQ. In DIMAQ, ground measurements from different years were all assumed to have been made in the primary year of interest and then regressed against values from other inputs (eg, satellites, etc.) made in that year. In the presence of changes over time, therefore, and particularly in areas where no recent measurements were available, there was the possibility of mismatches between the ground measurements and other variables. In DIMAQ2, ground

measurements were matched with other inputs (over time), and the (global-level) coefficients were allowed to vary over time, subject to smoothing that is induced by a first-order random walk process. In addition, the manner in which spatial variation can be incorporated within the model has developed: where there are sufficient data, the calibration equations can now vary (smoothly) both within and between countries, achieved by allowing the coefficients to follow (smooth) Gaussian processes. Where there are insufficient data within a country, to produce accurate equations, as before, information is borrowed from lower down the hierarchy and it is supplemented with information from the wider region.

DIMAQ2 as described above is used for all regions except for the north Africa and Middle East and sub-Saharan Africa super-regions, where there are insufficient data across years to allow the extra complexities of the new model to be implemented. In these super-regions, a simplified version of DIMAQ2 is used in which the temporal component is dropped.

Model evaluation

Model development and comparison was performed using within- and out-of-sample assessment. In the evaluation, cross-validation was performed using 25 combinations of training (80%) and validation (20%) datasets. Validation sets were obtained by taking a stratified random sample, using sampling probabilities based on the cross-tabulation of $PM_{2.5}$ categories (0-24.9, 25-49.9, 50-74.9, 75-99.9, 100+ $\mu g/m^3$) and super-regions, resulting in them having the same distribution of $PM_{2.5}$ concentrations and super-regions as the overall set of sites. The following metrics were calculated for each training/evaluation set combination: for model fit – R^2 and deviance information criteria (DIC, a measure of model fit for Bayesian models); for predictive accuracy – root mean squared error (RMSE) and population weighted root mean squared error (PwRMSE). The median R^2 was 0.9, and the median PwRMSE was 10.1 $\mu g/m^3$.

All modelling was performed on the log-scale. The choice of which variables were included in the model was made based on their contribution to model fit and predictive ability. The following is a list of variables and model structures that were included in DIMAQ.

Continuous explanatory variables:

- (SAT) Estimate of $PM_{2.5}$ (in $\mu g/m^3$) from satellite remote sensing on the log-scale.
- (POP) Estimate of population for the same year as SAT on the log-scale.
- (SNAOC) Estimate of the sum of sulfate, nitrate, ammonium, and organic carbon simulated using the GEOS Chem chemical transport model.
- (DST) Estimate of compositional concentrations of mineral dust simulated using the GEOS-Chem chemical transport model.
- (EDxDU) The log of the elevation difference between the elevation at the ground measurement location and the mean elevation within the GEOS Chem simulation grid cell multiplied by the inverse distance to the nearest urban land surface.

Discrete explanatory variables:

- (LOC) Binary variable indicating whether exact location of ground measurement is known.
- (TYPE) Binary variable indicating whether exact type of ground monitor is known.

- (CONV) Binary variable indicating whether ground measurement is $PM_{2.5}$ or converted from PM_{10}.

Interactions*:*

- Interactions between the binary variables and the effects of SAT.

Random effects:

- Regional temporal (random walk) hierarchical random-effects on the intercept
- Regional hierarchical random-effects for the coefficient associated with SAT
- Regional hierarchical random-effects for the coefficient associated with POP
- Smoothed, spatially varying random-effects for the intercept
- Smoothed, spatially varying random-effects for the coefficient associated with SAT

Inference and prediction

Due to both the complexity of the models and the size of the data, notably the number of spatial predictions that are required, recently developed techniques that perform "approximate" Bayesian inference based on integrated nested Laplace approximations (INLA) were used.[7] Computation was performed using the R interface to the INLA computational engine (R-INLA). GBD 2019 also makes use of an innovation in the way that samples from the (Bayesian) model are used to represent distributions of estimated concentrations in each grid-cell. Here estimates, and distributions representing uncertainty, of concentrations for each grid are obtained by taking repeated (joint) samples from the posterior distributions of the parameters and calculating estimates based on a linear combination of those samples and the input variables.[8]

DIMAQ2 was used to produce estimates of ambient $PM_{2.5}$ for 1990, 1995, and 2010–2019 by matching the gridded estimates with the corresponding coefficients from the calibration. As there is a lag in reporting ambient air pollution based quantities, the input variables were extrapolated (as in GBD 2017), allowing estimates for 2018 and 2019 to be produced in the same way as other years and, crucially, allowing measures of uncertainty to be produced within the BHM framework rather than by using post-hoc approximations.

Estimates from the satellites and the GEOS-Chem chemical transport model in 2018 and 2019 were produced by extrapolating estimates from 2000–2017 using generalised additive models,[9] on a cell-by-cell basis, except in those grid cells that saw a >100% increase between 2016 and 2017, in which case only the 2000–2016 estimates were used for extrapolating, in order to avoid unrealistic and/or unjustified extrapolation of trends. Population estimates for 2018 and 2019 were obtained by interpolation as described above.

Theoretical minimum-risk exposure level

The TMREL was assigned a uniform distribution with lower/upper bounds given by the average of the minimum and fifth percentiles of outdoor air pollution cohort studies exposure distributions conducted in North America, with the assumption that current evidence was insufficient to precisely characterise the shape of the concentration-response function below the fifth percentile of the exposure distributions. The TMREL was defined as a uniform distribution rather than a fixed value in order to represent the uncertainty regarding the level at which the scientific evidence was consistent with adverse effects of exposure. The specific outdoor air pollution cohort studies selected for this averaging

were based on the criteria that their fifth percentiles were less than that of the American Cancer Society Cancer Prevention II (CPSII) cohort's fifth percentile of 8.2 based on Turner and colleagues (2016).[10] This criterion was selected since GBD 2010 used the minimum, 5.8, and fifth percentile solely from the CPS II cohort. The resulting lower/upper bounds of the distribution for GBD 2019 were 2.4 and 5.9. This has not changed since GBD 2015.

Relative risks and population attributable fractions

We create one set of cause-specific risk curves for both household air pollution and ambient air pollution as two different sources of $PM_{2.5}$. In GBD 2017, we estimated the particulate matter-attributable burden of disease based on the relation of long-term exposure to PM2.5 with Ischemic Heart Disease, stroke (ischemic and hemorrhagic), COPD, lung cancer, acute lower respiratory infection, and Type II Diabetes. In GBD 2019, we added adverse birth outcomes including low birthweight and short gestation. Because these are already risk factors (and not outcomes) in the GBD, we performed a mediation analysis, in which a proportion of the burden attributable to low birthweight and short gestation was attributed to $PM_{2.5}$ pollution.

For the six non-mediated outcomes, we used results from cohort and case-control studies of ambient $PM_{2.5}$ pollution, cohort studies, case-control studies, and randomised-controlled trials of household use of solid fuel for cooking, and cohort and case-control studies of secondhand smoke. For the first time in GBD 2019, we no longer use active smoking data in the risk curves

For GBD 2019, we made several important changes to the risk functions. Previously, we have used relative risk estimates for active smoking, converting cigarettes-per-day to $PM_{2.5}$ exposure in order to estimate the $PM_{2.5}$ relative risk at the highest end of the $PM_{2.5}$ exposure-response curve. We took this approach because the vast majority of the air pollution epidemiological studies have been performed in low-pollution settings in high-income countries, preventing us from extrapolating the steep relationship at the beginning of the exposure range to locations with high exposure but no relative risk estimates, such as India and China. However, with the recent publication of studies in China and other higher-exposure settings and additional studies of HAP, we have been able to include more estimates at high $PM_{2.5}$ levels in the model.[11,12,13,14,15] Furthermore, in contrast to previous cycles of the GBD where the power function used to develop the IER required the inclusion of active smoking data to anchor the risk function, with the current use of splines and their flexibility, it is easier to fit functions to the (ambient, household, and SHS) data without active smoking data. Beginning in GBD 2019, we excluded active smoking studies from the risk curves. Removal of active smoking information removes an important source of uncertainty in our earlier estimates related to differences in dose rates and other aspects of exposure between active smoking and the other $PM_{2.5}$ sources, including differences in voluntary (active smoking) and involuntary (ambient and household $PM_{2.5}$, secondhand smoke) exposure.[16,17]

Additionally, in the past, we have built the curves for ischaemic heart disease and stroke based on studies of mortality and used evidence from three studies of both mortality and incidence to scale down the mortality curves to generate estimates of incidence risk. This year we extracted incidence and mortality from all available studies and included this as a covariate in the model. There was no significant difference between estimates of incidence risk and mortality risk, so we included both types of risk estimates in the curve fitting and used the same curve for both incidence and mortality. This is what was done for all other outcomes in the past and in GBD 2019.

For cardiovascular diseases, evidence suggests that the relative risk decreases with age.[18] To account for this in our model, we generate unique risk curves for every five-year age group from 25–29 to 95 and older for both ischaemic heart disease and stroke. Because we do not have risk data for every unique age group, we adjust each study based on the median age during follow-up to generate a full adjusted dataset for every curve. We calculate the median age of follow-up by taking the median (or mean) age at enrollment and adding one-half of median or mean follow-up time. If follow-up time is not available, we take 70% of total study period based on the observed ratio of follow-up time to total study period for other studies.

Once we have a median age during follow-up (*a*), we extrapolate each study to the full set of ages where the estimated datapoint for age, a_j, is calculated with the following equation and accompanying explanatory figure:

$$\log(RR)_{a_j} = \frac{\log(RR)_a - 0}{a - 110} * (a_j - 110)$$

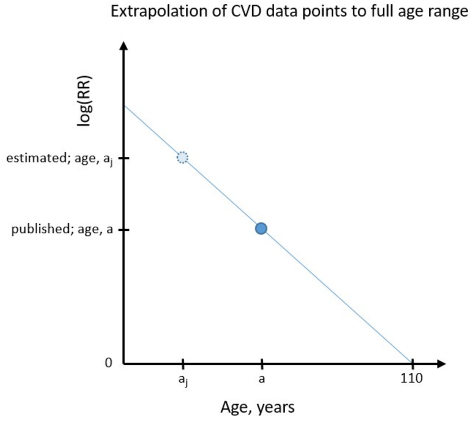

Previously we have used a fixed functional form to fit the risk curves.[16] In GBD 2019, we used MR-BRT (described in detail elsewhere) splines to fit the risk data with a more flexible shape. While previously we built in the TMREL estimates into the model fitting, this year we have fit the curve beginning at zero exposure and incorporate the TMREL into the relative risk calculation process. This allows others to use our risk curves with whatever counterfactual level is of interest to them. Relative risk curves are available upon request.

When fitting the risk curves, we consider the published relative risk over a range of exposure data. For OAP studies, the relative risk informs the curve from the fifth to the 95th percentile of observed exposure. When this is not available in the published study, we estimate the distribution from the provided information (mean and standard deviation, mean and IQR, etc.). We scale the RR to this range.

For HAP studies, we allow each study to inform the curve from the Exp_{OAP} to Exp_{OAP}+Exp_{HAP}, where Exp_{OAP} is the GBD 2017 estimate of the ambient exposure level in the study location and year, and Exp_{HAP} is the GBD 2017 estimate of the excess exposure for those who use solid fuel for cooking in the study location and year.

For SHS studies, we updated our strategy of exposure estimation in GBD 2019. For the first time, we are also accounting for outdoor exposure. Similar to the approach used for HAP, we allow each study to

inform the curve from the Exp_{OAP} to Exp_{OAP}+Exp_{SHS}, where Exp_{OAP} is the GBD 2017 estimate of the ambient exposure level in the study location and year, and Exp_{SHS} is an estimate of the excess exposure for those who experience secondhand smoke. This is estimated from the number of cigarettes smoked per smoker per day in a given location and year, estimated by the smoking team of GBD, and from a study in Sweden, which measured the $PM_{2.5}$ exposure in homes of smokers.[19] We divided the household $PM_{2.5}$ exposure level by the average number of cigarettes smoked per smoker per day in Sweden over the study duration to estimate the SHS $PM_{2.5}$ exposure per cigarette (2.31 μg/m^3 [95% UI 1.53–3.39]). To calculate Exp_{SHS} we multiplied the estimated number of cigarettes per smoker per day by the average $PM_{2.5}$ exposures per cigarette to generate a predicted $PM_{2.5}$ exposure level.

MR-BRT risk splines

We fit splines on the datasets including studies of OAP, HAP, and SHS using the following functional form, where X and X_{CF} represent the range of exposure characterised by the effect size:

$$log\left(\frac{MRBRT(X)}{MRBRT(X_{CF})}\right) \sim \log(Published\ Effect\ Size)$$

For each of the risk-outcome pairs, we tested various model settings and priors in fitting the MR-BRT splines. The final models used third-order splines with two interior knots and a constraint on the right-most segment, forcing the fit to be linear rather than cubic. We used an ensemble approach to knot placement, wherein 100 different models were run with randomly placed knots and then combined by weighting based on a measure of fit that penalises excessive changes in the third derivative of the curve. Knots were free to be placed anywhere within the fifth and 95th percentile of the data, as long as a minimum width of 10% of that domain exists between them. We included shape constraints so that the risk curves were concave down and monotonically increasing, the most biologically plausible shape for the $PM_{2.5}$ risk curve. On the non-linear segments, we included a Gaussian prior on the third derivative of mean 0 and variance 0.01 to prevent over-fitting; on the linear segment, a stronger prior of mean 0 and variance 1e-6 was used to ensure that the risk curves do not continue to increase beyond the range of the data.

For chronic obstructive pulmonary disease, we used a looser Gaussian prior of mean 0 and variance 1e-4 on the linear segment of the risk function. For this outcome, we have epidemiological evidence from household air pollution that the risk continues to increase at higher levels of $PM_{2.5}$.

Table 2 summarizes relative risk input data for ambient particulate matter pollution and household air pollution.

Table 2: Relative Risk Input Data

Input data	Relative risk
Source count (total)	200
Number of countries with data	40

The following table includes all ambient and household sources used in generating risk curves.

Source	Citation
1	Abusalah A, Gavana M, Haidich AB, Smyrnakis E, Papadakis N, Papanikolaou A, Benos A. Low birth weight and prenatal exposure to indoor pollution from tobacco smoke and wood fuel smoke: a matched case-control study in Gaza Strip. Matern Child Health J. 2012; 16(8): 1718-27.
2	Akhtar T, Ullah Z, Khan MH, Nazli R. Chronic bronchitis in women using solid biomass fuel in rural Peshawar, Pakistan. Chest. 2007; 132(5): 1472–5.
3	Alam DS, Chowdhury MAH, Siddiquee AT, Ahmed S, Hossain MD, Pervin S, Streatfield K, Cravioto A, Niessen LW. Adult Cardiopulmonary Mortality and Indoor Air Pollution: A 10-Year Retrospective Cohort Study in a Low-Income Rural Setting. Glob Heart. 2012; 7(3): 215–21.
4	Alexander DA, Northcross A, Karrison T, Morhasson-Bello O, Wilson N, Atalabi OM, Dutta A, Adu D, Ibigbami T, Olamijulo J, Adepoju D, Ojengbede O, Olopade CO. Pregnancy outcomes and ethanol cook stove intervention: A randomized-controlled trial in Ibadan, Nigeria. Environ Int. 2018; 111: 152-163.
5	Al-Sonboli N, Hart CA, Al-Aghbari N, Al-Ansi A, Ashoor O, Cuevas LE. Human metapneumovirus and respiratory syncytial virus disease in children, Yemen. Emerg Infect Dis. 2006; 12(9): 1437–9.
6	Atkinson RW, Carey IM, Kent AJ, van Staa TP, Anderson HR, Cook DG. Long-term exposure to outdoor air pollution and the incidence of chronic obstructive pulmonary disease in a national English cohort. Occup Environ Med. 2015; 72(1): 42–8.
7	Azizi BH, Zulkifli HI, Kasim MS. Protective and risk factors for acute respiratory infections in hospitalized urban Malaysian children: a case control study. Southeast Asian J Trop Med Public Health. 1995; 26(2): 280–5.
8	Balakrishnan K, Ghosh S, Thangavel G, Sambandam S, Mukhopadhyay K, Puttaswamy N, Sadasivam A, Ramaswamy P, Johnson P, Kuppuswamy R, Natesan D, Maheshwari U, Natarajan A, Rajendran G, Ramasami R, Madhav S, Manivannan S, Nargunanadan S, Natarajan S, Saidam S, Chakraborty M, Balakrishnan L, Thanasekaraan V. Exposures to fine particulate matter (PM2.5) and birthweight in a rural-urban, mother-child cohort in Tamil Nadu, India. Environ Res. 2018; 161: 524–31.
9	Basu R, Harris M, Sie L, Malig B, Broadwin R, Green R. Effects of fine particulate matter and its constituents on low birth weight among full-term infants in California. Environ Res. 2014; 128: 42–51.
10	Beelen R, Hoek G, van den Brandt PA, Goldbohm RA, Fischer P, Schouten LJ, Jerrett M, Hughes E, Armstrong B, Brunekreef B. Long-Term Effects of Traffic-Related Air Pollution on Mortality in a Dutch Cohort (NLCS-AIR Study) [Unpublished data]. Environ Health Perspect. 2008; 116(2): 196–202.
11	Beelen R, Hoek G, van den Brandt PA, Goldbohm RA, Fischer P, Schouten LJ, Jerrett M, Hughes E, Armstrong B, Brunekreef B. Long-Term Effects of Traffic-Related Air Pollution on Mortality in a Dutch Cohort (NLCS-AIR Study). Environ Health Perspect. 2008; 116(2): 196–202.
12	Beelen R, Stafoggia M, Raaschou-Nielsen O, Andersen ZJ, Xun WW, Katsouyanni K, Dimakopoulou K, Brunekreef B, Weinmayr G, Hoffmann B, Wolf K, Samoli E, Houthuijs D, Nieuwenhuijsen M, Oudin A, Forsberg B, Olsson D, Salomaa V, Lanki T, Yli-Tuomi T, Oftedal B, Aamodt G, Nafstad P, De Faire U, Pedersen NL, Östenson CG, Fratiglioni L, Penell J, Korek M, Pyko A, Eriksen KT, Tjønneland A, Becker T, Eeftens M, Bots M, Meliefste K, Wang M, Bueno-de-Mesquita B, Sugiri D, Krämer U, Heinrich J, de Hoogh K, Key T, Peters A, Cyrys J, Concin H, Nagel G, Ineichen A, Schaffner E, Probst-Hensch N, Dratva J, Ducret-Stich R, Vilier A, Clavel-Chapelon F, Stempfelet M, Grioni S, Krogh V, Tsai MY, Marcon A, Ricceri F, Sacerdote C, Galassi C, Migliore E, Ranzi A, Cesaroni G, Badaloni C, Forastiere F, Tamayo I, Amiano P, Dorronsoro M, Katsoulis M, Trichopoulou A, Vineis P, Hoek G. Long-term exposure to air pollution and cardiovascular mortality: an analysis of 22 European cohorts. Epidemiology. 2014; 25(3): 368–378.

Source	Citation
13	Bell ML, Belanger K, Ebisu K, Gent JF, Lee HJ, Koutrakis P, Leaderer BP. Prenatal Exposure to Fine Particulate Matter and Birth Weight: Variations by Particulate Constituents and Sources. Epidemiology. 2010; 21(6): 884–91.
14	Bell ML, Ebisu K, Belanger K. Ambient Air Pollution and Low Birth Weight in Connecticut and Massachusetts. Environ Health Perspect. 2007; 115(7): 1118–24.
15	Benmarhnia T, Huang J, Basu R, Wu J, Bruckner TA. Decomposition Analysis of Black-White Disparities in Birth Outcomes: The Relative Contribution of Air Pollution and Social Factors in California. Environ Health Perspect. 2017; 125(10): 107003.
16	Bowe B, Xie Y, Li T, Yan Y, Xian H, Al-Aly Z. The 2016 global and national burden of diabetes mellitus attributable to PM2·5 air pollution. Lancet Planet Health. 2018; 2(7): e301–12.
17	Boy E, Bruce N, Delgado H. Birth weight and exposure to kitchen wood smoke during pregnancy in rural Guatemala. Environ Health Perspect. 2002; 110(1): 109-14.
18	Brauer M, Lencar C, Tamburic L, Koehoorn M, Demers P, Karr C. A cohort study of traffic-related air pollution impacts on birth outcomes. Environ Health Perspect. 2008; 116(5): 680-6.
19	Broor S, Pandey RM, Ghosh M, Maitreyi RS, Lodha R, Singhal T, Kabra SK. Risk factors for severe acute lower respiratory tract infection in under-five children. Indian Pediatr. 2001; 1361-9.
20	Burnett RT. Cox Proportional Survival Model Hazard Ratios from Census Year to 2011 for Adults Aged 25 to 89 in CanCHEC Cohort.
21	Carey IM, Atkinson RW, Kent AJ, van Staa T, Cook DG, Anderson HR. Mortality associations with long-term exposure to outdoor air pollution in a national English cohort. Am J Respir Crit Care Med. 2013; 187(11): 1226-33.
22	Cesaroni G, Badaloni C, Gariazzo C, Stafoggia M, Sozzi R, Davoli M, Forastiere F. Long-term exposure to urban air pollution and mortality in a cohort of more than a million adults in Rome. Environ Health Perspect. 2013; 121(3): 324–31.
23	Chang HH, Reich BJ, Miranda ML. A spatial time-to-event approach for estimating associations between air pollution and preterm birth. J R Stat Soc Ser C Appl Stat. 2013; 62(2).
24	Chen H, Burnett RT, Kwong JC, Villeneuve PJ, Goldberg MS, Brook RD, van Donkelaar A, Jerrett M, Martin RV, Brook JR, Copes R. Risk of incident diabetes in relation to long-term exposure to fine particulate matter in Ontario, Canada. Environ Health Perspect. 2013; 121(7): 804–10.
25	Chen LH, Knutsen SF, Shavlik D, Beeson WL, Petersen F, Ghamsary M, Abbey D. The association between fatal coronary heart disease and ambient particulate air pollution: Are females at greater risk?. Environ Health Perspect. 2005; 113(12): 1723-9.
26	Clark C, Sbihi H, Tamburic L, Brauer M, Frank LD, Davies HW. Association of Long-Term Exposure to Transportation Noise and Traffic-Related Air Pollution with the Incidence of Diabetes: A Prospective Cohort Study. Environ Health Perspect. 2017; 125(8): 087025.
27	Clemens T, Turner S, Dibben C. Maternal exposure to ambient air pollution and fetal growth in North-East Scotland: A population-based study using routine ultrasound scans. Environ Int. 2017; 107: 216–26.
28	Coker E, Ghosh J, Jerrett M, Gomez-Rubio V, Beckerman B, Cockburn M, Liverani S, Su J, Li A, Kile ML, Ritz B, Molitor J. Modeling spatial effects of PM(2.5) on term low birth weight in Los Angeles County. Environ Res. 2015; 142: 354-64.

Source	Citation
29	Collings DA, Sithole SD, Martin KS. Indoor woodsmoke pollution causing lower respiratory disease in children. Trop Doct. 1990; 20(4): 151–5.
30	Coogan PF, White LF, Yu J, Burnett RT, Seto E, Brook RD, Palmer JR, Rosenberg L, Jerrett M. PM2.5 and Diabetes and Hypertension Incidence in the Black Women's Health Study. Epidemiology. 2016; 27(2): 202–10.
31	Dadvand P, Ostro B, Figueras F, Foraster M, Basagaña X, Valentín A, Martinez D, Beelen R, Cirach M, Hoek G, Jerrett M, Brunekreef B, Nieuwenhuijsen MJ. Residential proximity to major roads and term low birth weight: the roles of air pollution, heat, noise, and road-adjacent trees. Epidemiology. 2014; 25(4): 518-25.
32	Darrow LA, Klein M, Strickland MJ, Mulholland JA, Tolbert PE. Ambient Air Pollution and Birth Weight in Full-Term Infants in Atlanta, 1994–2004. Environ Health Perspect. 2011; 119(5): 731–7.
33	Dennis RJ, Maldonado D, Norman S, Baena E, Martinez G. Woodsmoke exposure and risk for obstructive airways disease among women. Chest. 1996; 109(1): 115–9.
34	Dherani M, Pope D, Mascarenhas M, Smith KR, Weber M, Bruce N. Indoor air pollution from unprocessed solid fuel use and pneumonia risk in children aged under five years: a systematic review and meta-analysis. Bull World Health Organ. 2008; 86(5): 390-398C and Kossove D. and Jeena PM, Ayannusi OE, Annamalai K, Naidoo P, Coovadia HM, Guldner P. Risk factors for admission and the role of respiratory syncytial virus-specific cytotoxic T-lymphocyte responses in children with acute bronchiolitis. S Afr Med J. 2003; 93(4): 291–4.
35	Ebisu K, Bell ML. Airborne PM2.5 chemical components and low birth weight in the northeastern and mid-Atlantic regions of the United States. Environ Health Perspect. 2012; 120(12): 1746-52.
36	Ebisu K, Berman JD, Bell ML. Exposure to coarse particulate matter during gestation and birth weight in the U.S. Environ Int. 2016; 94: 519–24.
37	Erickson AC, Ostry A, Chan LH, Arbour L. The reduction of birth weight by fine particulate matter and its modification by maternal and neighbourhood-level factors: a multilevel analysis in British Columbia, Canada. Environ Health. 2016; 15: 51.
38	Ezzati M, Kammen DM. Indoor air pollution from biomass combustion and acute respiratory infections in Kenya: an exposure-response study. Lancet. 2001; 358(9282): 619–24.
39	Fleischer NL, Merialdi M, van Donkelaar A, Vadillo-Ortega F, Martin RV, Betran AP, Souza JP. Outdoor air pollution, preterm birth, and low birth weight: analysis of the world health organization global survey on maternal and perinatal health. Environ Health Perspect. 2014; 122(4): 425-30.
40	Fonseca W, Kirkwood BR, Victora CG, Fuchs SR, Flores JA, Misago C. Risk factors for childhood pneumonia among the urban poor in Fortaleza, Brazil: a case--control study. Bull World Health Organ. 1996; 74(2): 199–208.
41	Galeone C, Pelucchi C, La Vecchia C, Negri E, Bosetti C, Hu J. Indoor air pollution from solid fuel use, chronic lung diseases and lung cancer in Harbin, Northeast China. Eur J Cancer Prev. 2008; 17(5): 473–8.
42	Gan WQ, FitzGerald JM, Carlsten C, Sadatsafavi M, Brauer M. Associations of ambient air pollution with chronic obstructive pulmonary disease hospitalization and mortality. Am J Respir Crit Care Med. 2013; 187(7): 721–7.
43	Gan WQ, Koehoorn M, Davies HW, Demers PA, Tamburic L, Brauer M. Long-Term Exposure to Traffic-Related Air Pollution and the Risk of Coronary Heart Disease Hospitalization and Mortality. Environ Health Perspect. 2011; 119(4): 501–7.

Source	Citation
44	Geer LA, Weedon J, Bell ML. Ambient air pollution and term birth weight in Texas from 1998 to 2004. J Air Waste Manag Assoc. 2012; 62(11): 1285–95.
45	Gehring U, Tamburic L, Sbihi H, Davies HW, Brauer M. Impact of Noise and Air Pollution on Pregnancy Outcomes. Epidemiology. 2014; 25(3): 351–8.
46	Gehring U, Wijga AH, Fischer P, de Jongste JC, Kerkhof M, Koppelman GH, Smit HA, Brunekreef B. Traffic-related air pollution, preterm birth and term birth weight in the PIAMA birth cohort study. Environ Res. 2011; 111(1): 125–35.
47	Ger LP, Hsu WL, Chen KT, Chen CJ. Risk Factors of Lung Cancer by Histological Category in Taiwan. Anticancer Res. 1993; 13(5A): 1491–500.
48	Gray SC, Edwards SE, Schultz BD, Miranda ML. Assessing the impact of race, social factors and air pollution on birth outcomes: a population-based study. Environ Health. 2014; 13(1): 4.
49	Gray SC, Gelfand AE, Miranda ML. Hierarchical spatial modeling of uncertainty in air pollution and birth weight study. Stat Med. 2011; 30(17): 2187-98.
50	Gupta D, Boffetta P, Gaborieau V, Jindal SK. Risk factors of lung cancer in Chandigarh, India. Indian J Med Res. 2001; 113: 142–50.
51	Ha S, Hu H, Roussos-Ross D, Haidong K, Roth J, Xu X. The effects of air pollution on adverse birth outcomes. Environ Res. 2014; 134: 198-204.
52	Hansen AB, Ravnskjær L, Loft S, Andersen KK, Bräuner EV, Baastrup R, Yao C, Ketzel M, Becker T, Brandt J, Hertel O, Andersen ZJ. Long-term exposure to fine particulate matter and incidence of diabetes in the Danish Nurse Cohort. Environ Int. 2016; 91: 243–50.
53	Hao H, Chang HH, Holmes HA, Mulholland JA, Klein M, Darrow LA, Strickland MJ. Air Pollution and Preterm Birth in the U.S. State of Georgia (2002-2006): Associations with Concentrations of 11 Ambient Air Pollutants Estimated by Combining Community Multiscale Air Quality Model (CMAQ) Simulations with Stationary Monitor Measurements. Environ Health Perspect. 2016; 124(6): 875-80.
54	Hao Y, Strosnider H, Balluz L, Qualters JR. Geographic Variation in the Association between Ambient Fine Particulate Matter (PM2.5) and Term Low Birth Weight in the United States. Environ Health Perspect. 2016; 124(2): 250-5.
55	Harris G, Thompson WD, Fitzgerald E, Wartenberg D. The association of PM(2.5) with full term low birth weight at different spatial scales. Environ Res. 2014; 134: 427-34.
56	Hart J, Garshick E, Dockery D, Smith T, Ryan L, Laden F. Long-Term Ambient Multipollutant Exposures and Mortality. Am J Respir Crit Care Med. 2011; 183: 75–8.
57	Hart JE, Puett RC, Rexrode KM, Albert CM, Laden F. Effect Modification of Long-Term Air Pollution Exposures and the Risk of Incident Cardiovascular Disease in US Women. J Am Heart Assoc. 2015; 4(12).
58	Heft-Neal S, Burney J, Bendavid E, Burke M. Robust relationship between air quality and infant mortality in Africa. Nature. 2018; 559(7713): 2548.
59	Hertz-Picciotto I, Baker RJ, Yap P-S, Dostál M, Joad JP, Lipsett M, Greenfield T, Herr CEW, Benes I, Shumway RH, Pinkerton KE, Srám R. Early childhood lower respiratory illness and air pollution. Environ Health Perspect. 2007; 115(10): 1510-8.
60	Huang C, Zhang X, Qiao Z, Guan L, Peng S, Liu J, Xie R, Zheng L. A case-control study of dietary factors in patients with lung cancer. Biomed Environ Sci. 1992; 5(3): 257–65.

Source	Citation
61	Huynh M, Woodruff TJ, Parker JD, Schoendorf KC. Relationships between air pollution and preterm birth in California. Paediatr Perinat Epidemiol. 2006; 20(6): 454-61.
62	Hyder A, Lee HJ, Ebisu K, Koutrakis P, Belanger K, Bell ML. PM2.5 Exposure and Birth Outcomes: Use of Satellite- and Monitor-Based Data. Epidemiology. 2014; 25(1): 58–67.
63	Hystad P, Demers PA, Johnson KC, Carpiano RM, Brauer M. Long-term residential exposure to air pollution and lung cancer risk. Epidemiology. 2013; 24(5): 762-72.
64	Hystad P, Duong M, Brauer M, Larkin A, Arku R, Kurmi OP, Fan WQ, Avezum A, Azam I, Chifamba J, Dans A, du Plessis JL, Gupta R, Kumar R, Lanas F, Liu Z, Lu Y, Lopez-Jaramillo P, Mony P, Mohan V, Mohan D, Nair S, Puoane T, Rahman O, Lap AT, Wang Y, Wei L, Yeates K, Rangarajan S, Teo K, Yusuf S, on behalf of Prospective Urban and Rural Epidemiological (PURE) Study investigators. Health Effects of Household Solid Fuel Use: Findings from 11 Countries within the Prospective Urban and Rural Epidemiology Study. Environ Health Perspect. 2019; 127(5): 57003.
65	Hystad P, Duong M, Brauer M, Larkin A, Arku R, Kurmi OP, Fan WQ, Avezum A, Azam I, Chifamba J, Dans A, du Plessis JL, Gupta R, Kumar R, Lanas F, Liu Z, Lu Y, Lopez-Jaramillo P, Mony P, Mohan V, Mohan D, Nair S, Puoane T, Rahman O, Lap AT, Wang Y, Wei L, Yeates K, Rangarajan S, Teo K, Yusuf S, on behalf of Prospective Urban and Rural Epidemiological (PURE) Study investigators. Health Effects of Household Solid Fuel Use: Findings from 11 Countries within the Prospective Urban and Rural Epidemiology Study [Unpublished]. Environ Health Perspect. 2019; 127(5): 57003.
66	Hystad P, Larkin A, Rangarajan S, PURE country investigators, Yusuf S, Brauer M. Outdoor fine particulate matter air pollution and cardiovascular disease: Results from 747 communities across 21 countries in the PURE Study [Unpublished].
67	Jedrychowski W, Perera F, Mrozek-Budzyn D, Mroz E, Flak E, Spengler JD, Edwards S, Jacek R, Kaim I, Skolicki Z. Gender differences in fetal growth of newborns exposed prenatally to airborne fine particulate matter. Environ Res. 2009; 109(4): 447-56.
68	Jin C, Rossignol AM. Effects of passive smoking on respiratory illness from birth to age eighteen months, in Shanghai, People's Republic of China. J Pediatr. 1993; 123(4): 553–8.
69	Johnson AW, Aderele WI. The association of household pollutants and socio-economic risk factors with the short-term outcome of acute lower respiratory infections in hospitalized pre-school Nigerian children. Ann Trop Paediatr. 1992; 12(4): 421–32.
70	Karr C, Lumley T, Schreuder A, Davis R, Larson T, Ritz B, Kaufman J. Effects of subchronic and chronic exposure to ambient air pollutants on infant bronchiolitis. Am J Epidemiol. 2007; 165(5): 553-60.
71	Karr CJ, Rudra CB, Miller KA, Gould TR, Larson T, Sathyanarayana S, Koenig JQ. Infant exposure to fine particulate matter and traffic and risk of hospitalization for RSV bronchiolitis in a region with lower ambient air pollution. Environ Res. 2009; 109(3): 321-7.
72	Katanoda K, Sobue T, Satoh H, Tajima K, Suzuki T, Nakatsuka H, Takezaki T, Nakayama T, Nitta H, Tanabe K, Tominaga S. An association between long-term exposure to ambient air pollution and mortality from lung cancer and respiratory diseases in Japan. J Epidemiol. 2011; 21(2): 132-43.
73	Kim C, Seow WJ, Shu X-O, Bassig BA, Rothman N, Chen BE, Xiang Y-B, Hosgood HD, Ji B-T, Hu W, Wen C, Chow W-H, Cai Q, Yang G, Gao Y-T, Zheng W, Lan Q. Cooking Coal Use and All-Cause and Cause-Specific Mortality in a Prospective Cohort Study of Women in Shanghai, China. Environ Health Perspect. 2016; 124(9): 1384–9.
74	Kleinerman RA, Wang Z, Wang L, Metayer C, Zhang S, Brenner AV, Zhang S, Xia Y, Shang B, Lubin JH. Lung cancer and indoor exposure to coal and biomass in rural China. J Occup Environ Med. 2002; 44(4): 338–44.

Source	Citation
75	Kloog I, Melly SJ, Ridgway WL, Coull BA, Schwartz J. Using new satellite based exposure methods to study the association between pregnancy pm2.5 exposure, premature birth and birth weight in Massachusetts. Environ Health. 2012; 11(1).
76	Ko YC, Lee CH, Chen MJ, Huang CC, Chang WY, Lin HJ, Wang HZ, Chang PY. Risk factors for primary lung cancer among non-smoking women in Taiwan. Int J Epidemiol. 1997; 26(1): 24-31.
77	Kumar N. Uncertainty in the relationship between criteria pollutants and low birth weight in Chicago. Atmos Environ. 2012; 49: 171–9.
78	Kumar S, Awasthi S, Jain A, Srivastava RC. Blood zinc levels in children hospitalized with severe pneumonia: a case control study. Indian Pediatr. 2004; 41(5): 486–91.
79	Lan Q, He X, Shen M, Tian L, Liu LZ, Lai H, Chen W, Berndt SI, Hosgood HD, Lee K-M, Zheng T, Blair A, Chapman RS. Variation in lung cancer risk by smoky coal subtype in Xuanwei, China. Int J Cancer. 2008; 123(9): 2164–9.
80	Laurent O, Hu J, Li L, Cockburn M, Escobedo L, Kleeman MJ, Wu J. Sources and contents of air pollution affecting term low birth weight in Los Angeles County, California, 2001-2008. Environ Res. 2014; 134: 488-95.
81	Laurent O, Hu J, Li L, Kleeman MJ, Bartell SM, Cockburn M, Escobedo L, Wu J. A Statewide Nested Case-Control Study of Preterm Birth and Air Pollution by Source and Composition: California, 2001-2008. Environ Health Perspect. 2016; 124(9): 1479-86.
82	Laurent O, Hu J, Li L, Kleeman MJ, Bartell SM, Cockburn M, Escobedo L, Wu J. Low birth weight and air pollution in California: Which sources and components drive the risk?. Environ Int. 2016; 92-93: 471-7.
83	Laurent O, Wu J, Li L, Chung J, Bartell S. Investigating the association between birth weight and complementary air pollution metrics: a cohort study. Environ Health. 2013; 12(1).
84	Lavigne E, Yasseen AS 3rd, Stieb DM, Hystad P, van Donkelaar A, Martin RV, Brook JR, Crouse DL, Burnett RT, Chen H, Weichenthal S, Johnson M, Villeneuve PJ, Walker M. Ambient air pollution and adverse birth outcomes: Differences by maternal comorbidities. Environ Res. 2016; 148: 457-466.
85	Le CH, Ko YC, Cheng LS, Lin YC, Lin HJ, Huang MS, Huang JJ, Kao EL, Wang HZ. The heterogeneity in risk factors of lung cancer and the difference of histologic distribution between genders in Taiwan. Cancer Causes Control. 2001; 12(4): 289–300.
86	Lepeule J, Laden F, Dockery D, Schwartz J. Chronic exposure to fine particles and mortality: an extended follow-up of the Harvard Six Cities study from 1974 to 2009 - Unpublished data. Environ Health Perspect. 2012; 120(7): 965-70.
87	Lepeule J, Laden F, Dockery D, Schwartz J. Chronic exposure to fine particles and mortality: an extended follow-up of the Harvard Six Cities study from 1974 to 2009. Environ Health Perspect. 2012; 120(7): 965-70.
88	Lim CC, Hayes RB, Ahn J, Shao Y, Silverman DT, Jones RR, Garcia C, Thurston GD. Association between long-term exposure to ambient air pollution and diabetes mortality in the US. Environ Res. 2018; 165: 330-36
89	Lipsett MJ, Ostro BD, Reynolds P, Goldberg D, Hertz A, Jerrett M, Smith DF, Garcia C, Chang ET, Bernstein L. Long-term exposure to air pollution and cardiorespiratory disease in the California teachers study cohort [Unpublished data]. Am J Respir Crit Care Med. 2011; 184(7): 828-35.
90	Lipsett MJ, Ostro BD, Reynolds P, Goldberg D, Hertz A, Jerrett M, Smith DF, Garcia C, Chang ET, Bernstein L. Long-term exposure to air pollution and cardiorespiratory disease in the California teachers study cohort. Am J Respir Crit Care Med. 2011; 184(7): 828-35.
91	Lissowska J, Bardin-Mikolajczak A, Fletcher T, Zaridze D, Szeszenia-Dabrowska N, Rudnai P, Fabianova E, Cassidy A, Mates D, Holcatova I, Vitova V, Janout V, Mannetje A, Brennan P, Boffetta P. Lung cancer and

Source	Citation
	indoor pollution from heating and cooking with solid fuels: the IARC international multicentre case-control study in Eastern/Central Europe and the United Kingdom. Am J Epidemiol. 2005; 162(4): 326–33.
92	Luo RX, Wu B, Yi YN, Huang ZW, Lin RT. Indoor burning coal air pollution and lung cancer--a case-control study in Fuzhou, China. Lung Cancer. 1996; 14 Suppl 1: S113-119.
93	MacIntyre EA, Gehring U, Mölter A, Fuertes E, Klümper C, Krämer U, Quass U, Hoffmann B, Gascon M, Brunekreef B, Koppelman GH, Beelen R, Hoek G, Birk M, de Jongste JC, Smit HA, Cyrys J, Gruzieva O, Korek M, Bergström A, Agius RM, de Vocht F, Simpson A, Porta D, Forastiere F, Badaloni C, Cesaroni G, Esplugues A, Fernández-Somoano A, Lerxundi A, Sunyer J, Cirach M, Nieuwenhuijsen MJ, Pershagen G, Heinrich J. Air Pollution and Respiratory Infections during Early Childhood: An Analysis of 10 European Birth Cohorts within the ESCAPE Project. Environ Health Perspect. 2014; 122(1): 107–13.
94	Mahalanabis D, Gupta S, Paul D, Gupta A, Lahiri M, Khaled MA. Risk factors for pneumonia in infants and young children and the role of solid fuel for cooking: a case-control study. Epidemiol Infect. 2002; 129(1): 65–71.
95	Miller KA, Siscovick DS, Sheppard L, Shepherd K, Sullivan JH, Anderson GL, Kaufman JD. Long-term exposure to air pollution and incidence of cardiovascular events in women. N Engl J Med. 2007; 356(5): 447-58.
96	Morello-Frosch R, Jesdale BM, Sadd JL, Pastor M. Ambient air pollution exposure and full-term birth weight in California. Environ Health. 2010; 9(1).
97	Naess Ø, Nafstad P, Aamodt G, Claussen B, Rosland P. Relation between concentration of air pollution and cause-specific mortality: four-year exposures to nitrogen dioxide and particulate matter pollutants in 470 neighborhoods in Oslo, Norway. Am J Epidemiol. 2007; 165(4): 435-43.
98	Park SK, Adar SD, O'Neill MS, Auchincloss AH, Szpiro A, Bertoni AG, Navas-Acien A, Kaufman JD, Diez-Roux AV. Long-term exposure to air pollution and type 2 diabetes mellitus in a multiethnic cohort. Am J Epidemiol. 2015; 181(5): 327–36.
99	Parker JD, Kravets N, Vaidyanathan A. Particulate Matter Air Pollution Exposure and Heart Disease Mortality Risks by Race and Ethnicity in the United States. Circulation. 2018; 137(16): 1688–97.
100	Parker JD, Woodruff TJ, Basu R, Schoendorf KC. Air Pollution and Birth Weight Among Term Infants in California. Pediatrics. 2005; 115(1): 121–8.
101	Parker JD, Woodruff TJ. Influences of study design and location on the relationship between particulate matter air pollution and birthweight. Paediatr Perinat Epidemiol. 2008; 22(3): 214–27.
102	Pereira G, Belanger K, Ebisu K, Bell ML. Fine particulate matter and risk of preterm birth in Connecticut in 2000-2006: a longitudinal study. Am J Epidemiol. 2014; 179(1): 67-74.
103	Pereira G, Bell ML, Belanger K, de Klerk N. Fine particulate matter and risk of preterm birth and pre-labor rupture of membranes in Perth, Western Australia 1997-2007: a longitudinal study. Environ Int. 2014; 73: 143-9.
104	Pinault L, Tjepkema M, Crouse DL, Weichenthal S, van Donkelaar A, Martin RV, Brauer M, Chen H, Burnett RT. Risk estimates of mortality attributed to low concentrations of ambient fine particulate matter in the Canadian community health survey cohort [Unpublished]. Environ Health. 2016; 15: 18.
105	Pinault L, Tjepkema M, Crouse DL, Weichenthal S, van Donkelaar A, Martin RV, Brauer M, Chen H, Burnett RT. Risk estimates of mortality attributed to low concentrations of ambient fine particulate matter in the Canadian community health survey cohort. Environ Health. 2016; 15(1): 18.

Source	Citation
106	Puett RC, Hart JE, Schwartz J, Hu FB, Liese AD, Laden F. Are particulate matter exposures associated with risk of type 2 diabetes? Environ Health Perspect. 2011; 119(3): 384–9.
107	Puett RC, Hart JE, Suh H, Mittleman M, Laden F. Particulate matter exposures, mortality, and cardiovascular disease in the health professionals follow-up study. Environ Health Perspect. 2011; 119(8): 1130-5.
108	Puett RC, Hart JE, Yanosky JD, Paciorek C, Schwartz J, Suh H, Speizer FE, Laden F. Chronic fine and coarse particulate exposure, mortality, and coronary heart disease in the Nurses' Health Study. Environ Health Perspect. 2009; 117(11): 1697-701.
109	Qian Z, Liang S, Yang S, Trevathan E, Huang Z, Yang R, Wang J, Hu K, Zhang Y, Vaughn M, Shen L, Liu W, Li P, Ward P, Yang L, Zhang W, Chen W, Dong G, Zheng T, Xu S, Zhang B. Ambient air pollution and preterm birth: A prospective birth cohort study in Wuhan, China. Int J Hyg Environ Health. 2016; 219(2): 195-203.
110	Qiu H, Schooling CM, Sun S, Tsang H, Yang Y, Lee RS, Wong CM, Tian L. Long-term exposure to fine particulate matter air pollution and type 2 diabetes mellitus in elderly: A cohort study in Hong Kong. Environ Int. 2018; 113: 350-56.
111	Raaschou-Nielsen O, Andersen ZJ, Beelen R, Samoli E, Stafoggia M, Weinmayr G, Hoffmann B, Fischer P, Nieuwenhuijsen MJ, Brunekreef B, Xun WW, Katsouyanni K, Dimakopoulou K, Sommar J, Forsberg B, Modig L, Oudin A, Oftedal B, Schwarze PE, Nafstad P, De Faire U, Pedersen NL, Ostenson C-G, Fratiglioni L, Penell J, Korek M, Pershagen G, Eriksen KT, Sørensen M, Tjønneland A, Ellermann T, Eeftens M, Peeters PH, Meliefste K, Wang M, Bueno-de-Mesquita B, Key TJ, de Hoogh K, Concin H, Nagel G, Vilier A, Grioni S, Krogh V, Tsai M-Y, Ricceri F, Sacerdote C, Galassi C, Migliore E, Ranzi A, Cesaroni G, Badaloni C, Forastiere F, Tamayo I, Amiano P, Dorronsoro M, Trichopoulou A, Bamia C, Vineis P, Hoek G. Air pollution and lung cancer incidence in 17 European cohorts: prospective analyses from the European Study of Cohorts for Air Pollution Effects (ESCAPE). Lancet Oncol. 2013; 14(9): 813–22.
112	Robin LF, Less PS, Winget M, Steinhoff M, Moulton LH, Santosham M, Correa A. Wood-burning stoves and lower respiratory illnesses in Navajo children. Pediatr Infect Dis J. 1996; 15(10): 859–65.
113	Sapkota A, Gajalakshmi V, Jetly DH, Roychowdhury S, Dikshit RP, Brennan P, Hashibe M, Boffetta P. Indoor air pollution from solid fuels and risk of hypopharyngeal/laryngeal and lung cancers: a multicentric case-control study from India. Int J Epidemiol. 2008; 37(2): 321–8.
114	Sasco AJ, Merrill RM, Dari I, Benhaïm-Luzon V, Carriot F, Cann CI, Bartal M. A case-control study of lung cancer in Casablanca, Morocco. Cancer Causes Control. 2002; 13(7): 609–16.
115	Savitha MR, Nandeeshwara SB, Pradeep Kumar MJ, ul-Haque F, Raju CK. Modifiable risk factors for acute lower respiratory tract infections. Indian J Pediatr. 2007; 74(5): 477–82.
116	Savitz DA, Bobb JF, Carr JL, Clougherty JE, Dominici F, Elston B, Ito K, Ross Z, Yee M, Matte TD. Ambient Fine Particulate Matter, Nitrogen Dioxide, and Term Birth Weight in New York, New York. Am J Epidemiol. 2014; 179(4): 457–66.
117	Sezer H, Akkurt I, Guler N, Marako?lu K, Berk S. A case-control study on the effect of exposure to different substances on the development of COPD. Ann Epidemiol. 2006; 16(1): 59–62.
118	Shah N, Ramankutty V, Premila PG, Sathy N. Risk factors for severe pneumonia in children in south Kerala: a hospital-based case-control study. J Trop Pediatr. 1994; 40(4): 201–6.
119	Shen M, Chapman RS, Vermeulen R, Tian L, Zheng T, Chen BE, Engels EA, He X, Blair A, Lan Q. Coal use, stove improvement, and adult pneumonia mortality in Xuanwei, China: a retrospective cohort study. Environ Health Perspect. 2009; 117(2): 261–6.

Source	Citation
120	Siddiqui AR, Gold EB, Yang X, Lee K, Brown KH, Bhutta ZA. Prenatal exposure to wood fuel smoke and low birth weight. Environ Health Perspect. 2008; 116(4): 543-9.
121	Smith KR, McCracken JP, Weber MW, Hubbard A, Jenny A, Thompson LM, Balmes J, Diaz A, Arana B, Bruce N. Effect of reduction in household air pollution on childhood pneumonia in Guatemala (RESPIRE): a randomised controlled trial. Lancet. 2011; 378(9804): 1717-26.
122	Smith RB, Fecht D, Gulliver J, Beevers SD, Dajnak D, Blangiardo M, Ghosh RE, Hansell AL, Kelly FJ, Anderson HR, Toledano MB. Impact of London's road traffic air and noise pollution on birth weight: retrospective population based cohort study. BMJ. 2017; 359: j5299.
123	Stafoggia M, Cesaroni G, Peters A, Andersen ZJ, Badaloni C, Beelen R, Caracciolo B, Cyrys J, de Faire U, de Hoogh K, Eriksen KT, Fratiglioni L, Galassi C, Gigante B, Havulinna AS, Hennig F, Hilding A, Hoek G, Hoffmann B, Houthuijs D, Korek M, Lanki T, Leander K, Magnusson PK, Meisinger C, Migliore E, Overvad K, Ostenson C-G, Pedersen NL, Pekkanen J, Penell J, Pershagen G, Pundt N, Pyko A, Raaschou-Nielsen O, Ranzi A, Ricceri F, Sacerdote C, Swart WJR, Turunen AW, Vineis P, Weimar C, Weinmayr G, Wolf K, Brunekreef B, Forastiere F. Long-term exposure to ambient air pollution and incidence of cerebrovascular events: results from 11 European cohorts within the ESCAPE project. Environ Health Perspect. 2014; 122(9): 919–25.
124	Stieb DM, Chen L, Beckerman BS, Jerrett M, Crouse DL, Omariba DW, Peters PA, van Donkelaar A, Martin RV, Burnett RT, Gilbert NL, Tjepkema M, Liu S, Dugandzic RM. Associations of Pregnancy Outcomes and PM2.5 in a National Canadian Study. Environ Health Perspect. 2016; 124(2): 243-9.
125	Thompson LM, Bruce N, Eskenazi B, Diaz A, Pope D, Smith KR. Impact of reduced maternal exposures to wood smoke from an introduced chimney stove on newborn birth weight in rural Guatemala. Environ Health Perspect. 2011; 119(10): 1489-94.
126	Thurston GD, Ahn J, Cromar KR, Shao Y, Reynolds HR, Jerrett M, Lim CC, Shanley R, Park Y, Hayes RB. Ambient Particulate Matter Air Pollution Exposure and Mortality in the NIH-AARP Diet and Health Cohort [Unpublished]. Environ Health Perspect. 2016; 124(4): 484-90.
127	Tielsch JM, Katz J, Thulasiraj RD, Coles CL, Sheeladevi S, Yanik EL, Rahmathullah L. Exposure to indoor biomass fuel and tobacco smoke and risk of adverse reproductive outcomes, mortality, respiratory morbidity and growth among newborn infants in south India. Int J Epidemiol. 2009; 38(5): 1351-63.
128	To T, Zhu J, Villeneuve PJ, Simatovic J, Feldman L, Gao C, Williams D, Chen H, Weichenthal S, Wall C, Miller AB. Chronic disease prevalence in women and air pollution--A 30-year longitudinal cohort study. Environ Int. 2015; 80: 26–32.
129	Tseng E, Ho W-C, Lin M-H, Cheng T-J, Chen P-C, Lin H-H. Chronic exposure to particulate matter and risk of cardiovascular mortality: cohort study from Taiwan. BMC Public Health. 2015; 15: 936.
130	Turner MC,Jerrett M, Pope CA 3rd, Krewski D, Gapstur SM, Diver WR, Beckerman BS, Marshall JD, Su J, Crouse DL, Burnett RT. Long-term ozone exposure and mortality in a large prospective study . Am J Respir Crit Care Med. 2016; 193(10): 1134-42.
131	Victora CG, Fuchs SC, Flores JA, Fonseca W, Kirkwood B. Risk factors for pneumonia among children in a Brazilian metropolitan area. Pediatrics. 1994; 977-85.
132	Villeneuve PJ, Weichenthal SA, Crouse D, Miller AB, To T, Martin RV, van Donkelaar A, Wall C, Burnett RT. Long-term exposure to fine particulate matter air pollution and mortality among Canadian women. Epidemiology. 2015; 26(4): 536-45.
133	Wayse V, Yousafzai A, Mogale K, Filteau S. Association of subclinical vitamin D deficiency with severe acute lower respiratory infection in Indian children under 5 y. Eur J Clin Nutr. 2004; 58(4): 563–7.

Source	Citation
134	Weichenthal S, Villeneuve PJ, Burnett RT, van Donkelaar A, Martin RV, Jones RR, DellaValle CT, Sandler DP, Ward MH, Hoppin JA. Long-term exposure to fine particulate matter: association with nonaccidental and cardiovascular mortality in the agricultural health study cohort. Environ Health Perspect. 2014; 122(6): 609-15.
135	Weinmayr G, Hennig F, Fuks K, Nonnemacher M, Jakobs H, Möhlenkamp S, Erbel R, Jöckel K-H, Hoffmann B, Moebus S, Heinz Nixdorf Recall Investigator Group. Long-term exposure to fine particulate matter and incidence of type 2 diabetes mellitus in a cohort study: effects of total and traffic-specific air pollution. Environ Health. 2015; 14: 53.
136	Wesley AG, Loening WE. Assessment and 2-year follow-up of some factors associated with severity of respiratory infections in early childhood. S Afr Med J. 1996; 86(4): 365–8.
137	Wilhelm M, Ghosh JK, Su J, Cockburn M, Jerrett M, Ritz B. Traffic-related air toxics and preterm birth: a population-based case-control study in Los Angeles County, California. Environ Health. 2011; 10: 89.
138	Wong CM, Lai HK, Tsang H, Thach TQ, Thomas GN, Lam KBH, Chan KP, Yang L, Lau AKH, Ayres JG, Lee SY, Man Chan W, Hedley AJ, Lam TH. Satellite-Based Estimates of Long-Term Exposure to Fine Particles and Association with Mortality in Elderly Hong Kong Residents. Environ Health Perspect. 2015; 123(11): 1167-72.
139	Wu AH, Henderson BE, Pike MC, Yu MC. Smoking and other risk factors for lung cancer in women. J Natl Cancer Inst. 1985; 74(4): 747-51.
140	Wu J, Wilhelm M, Chung J, Ritz B. Comparing exposure assessment methods for traffic-related air pollution in an adverse pregnancy outcome study. Environ Res. 2011; 111(5): 685-92.
141	Wylie BJ, Coull BA, Hamer DH, Singh MP, Jack D, Yeboah-Antwi K, Sabin L, Singh N, MacLeod WB. Impact of biomass fuels on pregnancy outcomes in central East India. Environ Health. 2014; 13(1): 1.
142	Wylie BJ, Kishashu Y, Matechi E, Zhou Z, Coull B, Abioye AI, Dionisio KL, Mugusi F, Premji Z, Fawzi W, Hauser R, Ezzati M. Maternal exposure to carbon monoxide and fine particulate matter during pregnancy in an urban Tanzanian cohort. Indoor Air. 2017; 27(1): 136-146.
143	Yin P, Brauer M, Cohen A, Burnett RT, Liu J, Liu Y, Liang R, Wang W, Qi J, Wang L, Zhou M. Long-term Fine Particulate Matter Exposure and Nonaccidental and Cause-specific Mortality in a Large National Cohort of Chinese Men [Unpublished]. Environ Health Perspect. 2017; 125(11): 117002.
144	Yin P, Brauer M, Cohen A, Burnett RT, Liu J, Liu Y, Liang R, Wang W, Qi J, Wang L, Zhou M. Long-term Fine Particulate Matter Exposure and Nonaccidental and Cause-specific Mortality in a Large National Cohort of Chinese Men. Environ Health Perspect. 2017; 125(11): 117002.
145	Yu K, Qiu G, Chan K-H, Lam K-BH, Kurmi OP, Bennett DA, Yu C, Pan A, Lv J, Guo Y, Bian Z, Yang L, Chen Y, Hu FB, Chen Z, Li L, Wu T. Association of Solid Fuel Use With Risk of Cardiovascular and All-Cause Mortality in Rural China. JAMA. 2018; 319(13): 1351–61.
146	Yucra S, Tapia V, Steenland K, Naeher LP, Gonzales GF. Association between biofuel exposure and adverse birth outcomes at high altitudes in Peru: a matched case-control study. Int J Occup Environ Health. 2011; 17(4): 307-13.

The following figures display risk curves for each outcome. The dashed line depicts the GBD 2017 IER including active smoking data, the dotted line depicts the GBD 2019 IER including active smoking data and updates to the AS and SHS exposure incorporation, and the solid line depicts the GBD 2019 MR-BRT curve without the inclusion of active smoking data. The grey shaded areas represent the 95% CI. The red

box represents the TMREL area of the curve. On each page, the first figure depicts the typical range of outdoor exposure, whereas the second plot includes higher levels typical of household air pollution exposure.

Each point or number represents one study effect size. Each is plotted at the 95th percentile of the exposure distribution (OAP), the expected level of exposure for individual using solid fuel (HAP), or the expected level of exposure for individuals experiencing SHS. The relative risk is plotted relative to the predicted relative risk at the fifth percentile of exposure distribution (OAP), the expected (ambient only) level of exposure for individuals not using solid fuel (HAP), or the expected (ambient only) level of exposure for individuals not exposed to SHS. For example, a study predicting a relative risk of 1.5 for an exposure range of 10 to 20 would be plotted at (20, MRBRT(10)*1.5). Arrows represent studies that would have been outside the range of the plot but have been moved to include on the figure.

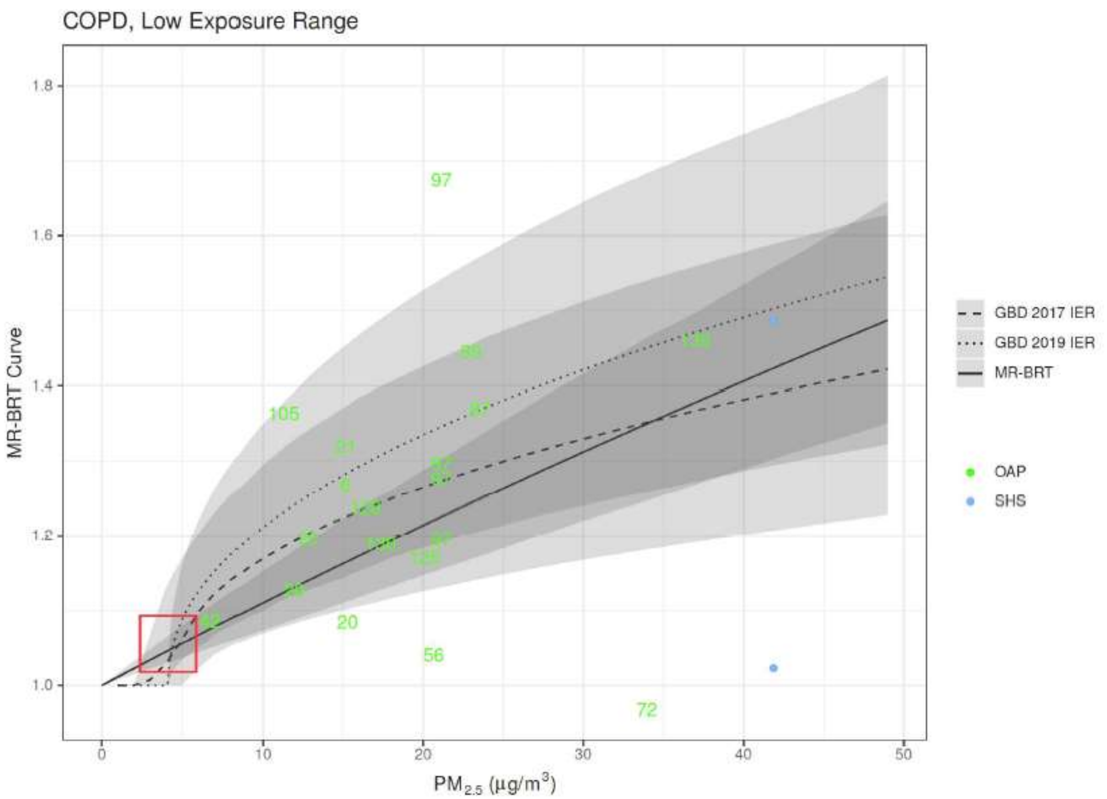
COPD, Low Exposure Range
MR-BRT Curve
PM2.5 (µg/m3)
GBD 2017 IER
GBD 2019 IER
MR-BRT
OAP
SHS
97
105
20
56
72

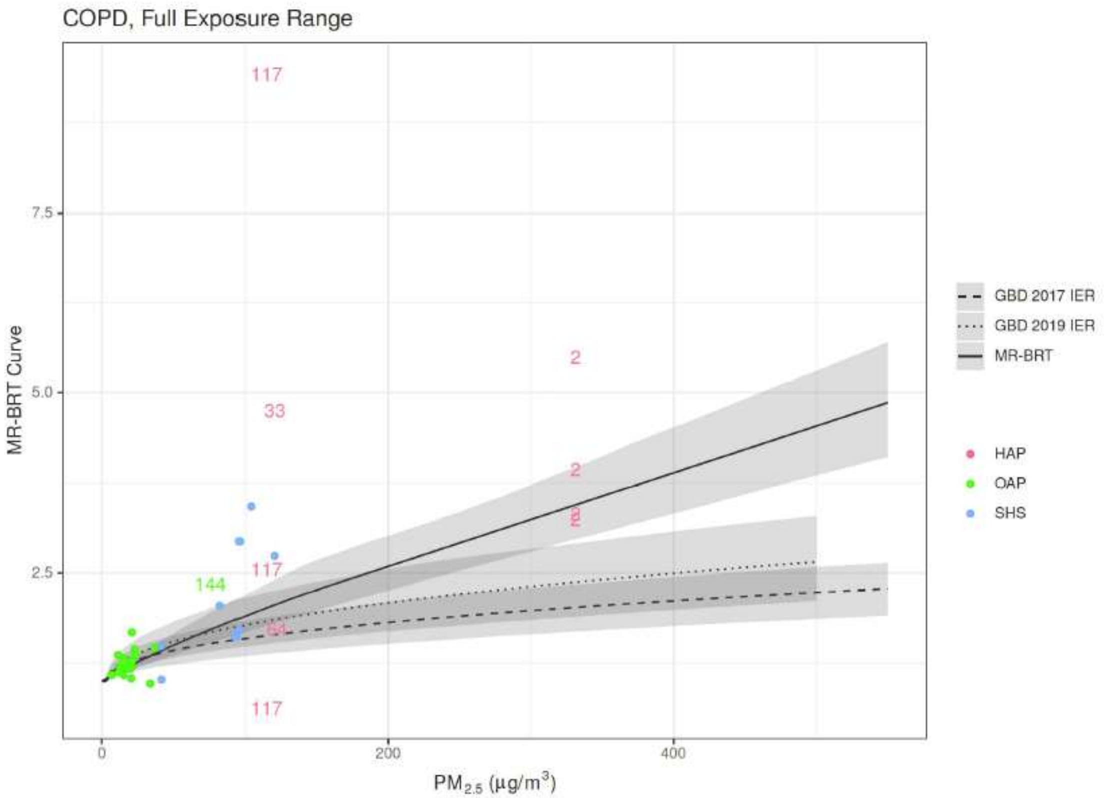
COPD, Full Exposure Range
MR-BRT Curve
PM2.5 (µg/m3)
GBD 2017 IER
GBD 2019 IER
MR-BRT
HAP
OAP
SHS
117
33
2
144
117

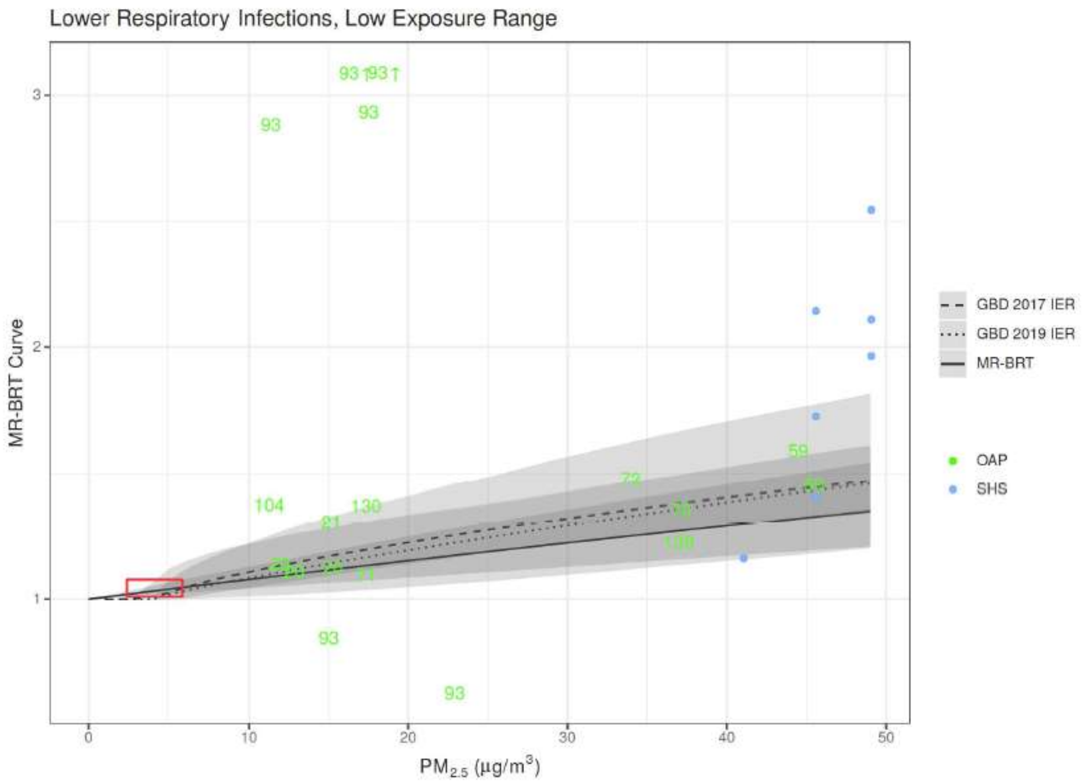
Lower Respiratory Infections, Low Exposure Range
MR-BRT Curve
PM$_{2.5}$ (µg/m^3)
GBD 2017 IER
GBD 2019 IER
MR-BRT
OAP
SHS

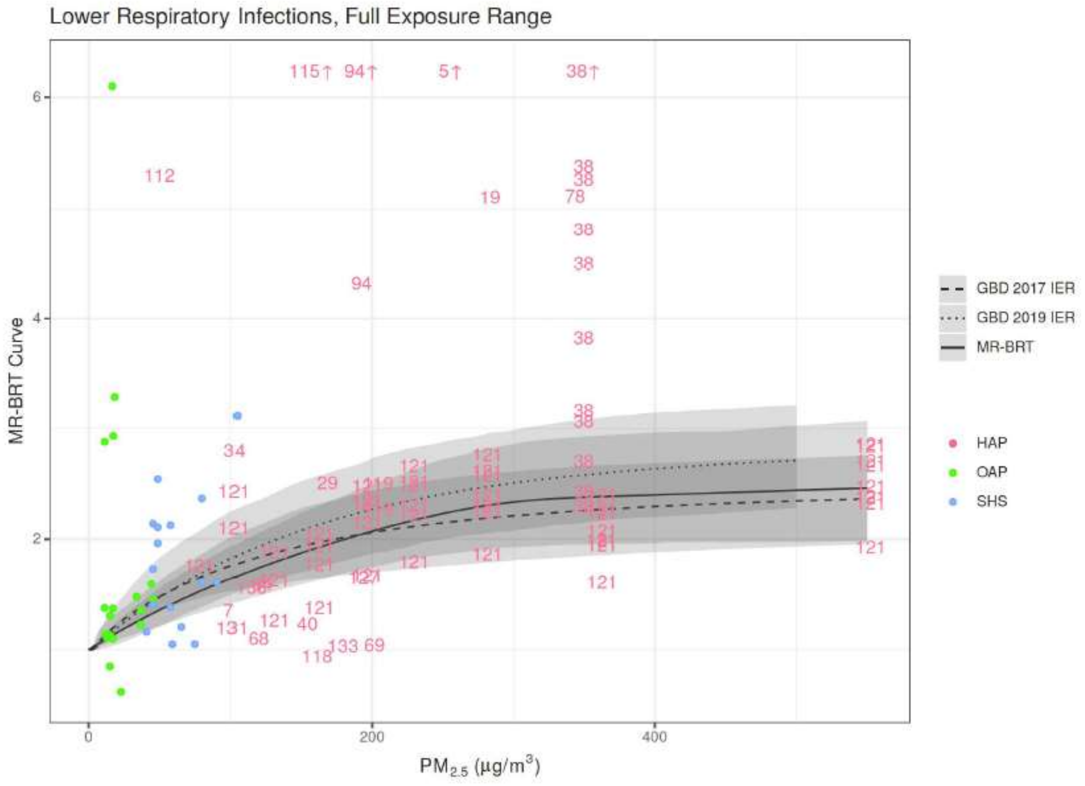
Lower Respiratory Infections, Full Exposure Range
MR-BRT Curve
PM$_{2.5}$ (µg/m^3)
GBD 2017 IER
GBD 2019 IER
MR-BRT
HAP
OAP
SHS

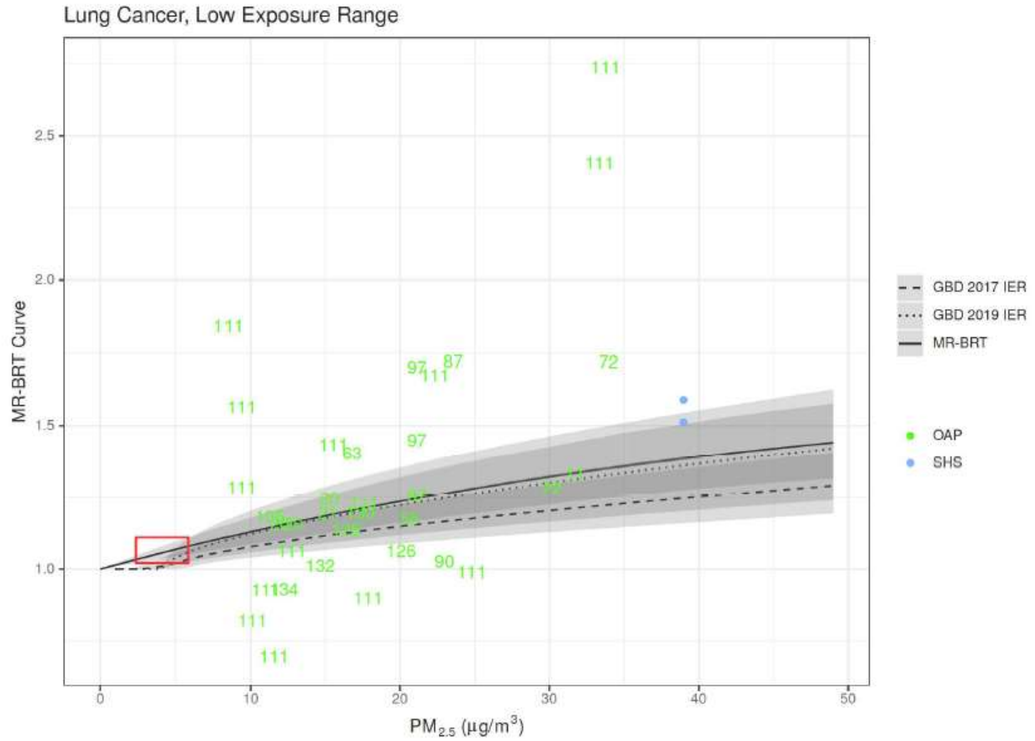
Lung Cancer, Low Exposure Range
MR-BRT Curve
PM2.5 (µg/m³)
GBD 2017 IER
GBD 2019 IER
MR-BRT
OAP
SHS

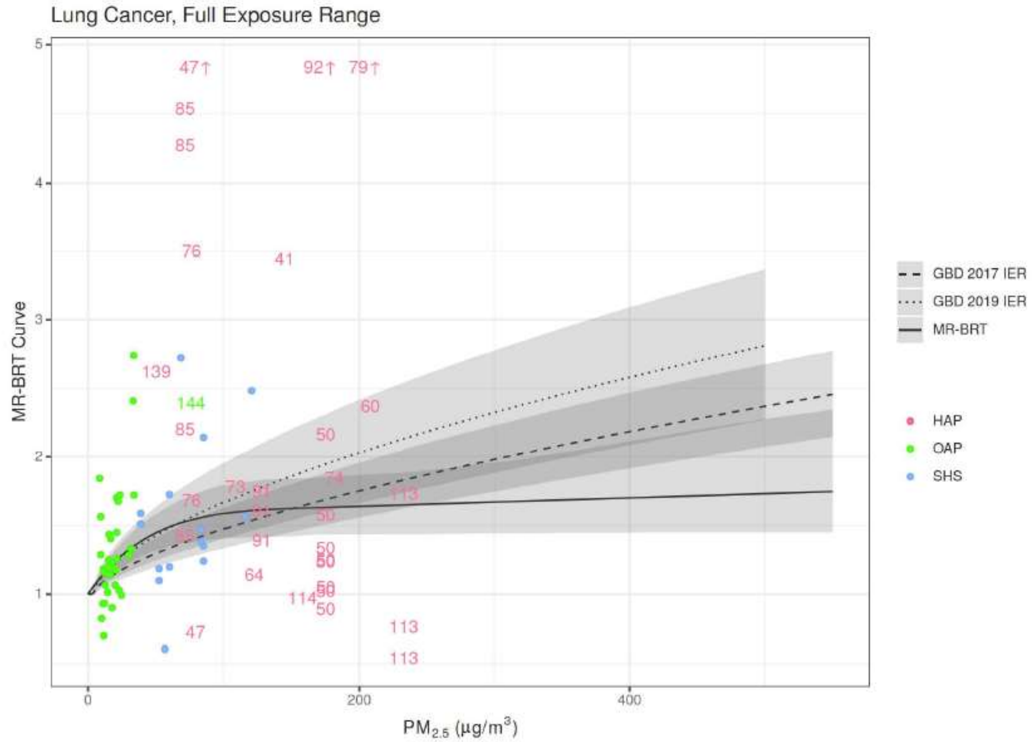
Lung Cancer, Full Exposure Range
MR-BRT Curve
PM2.5 (µg/m³)
GBD 2017 IER
GBD 2019 IER
MR-BRT
HAP
OAP
SHS

Type 2 Diabetes, Low Exposure Range

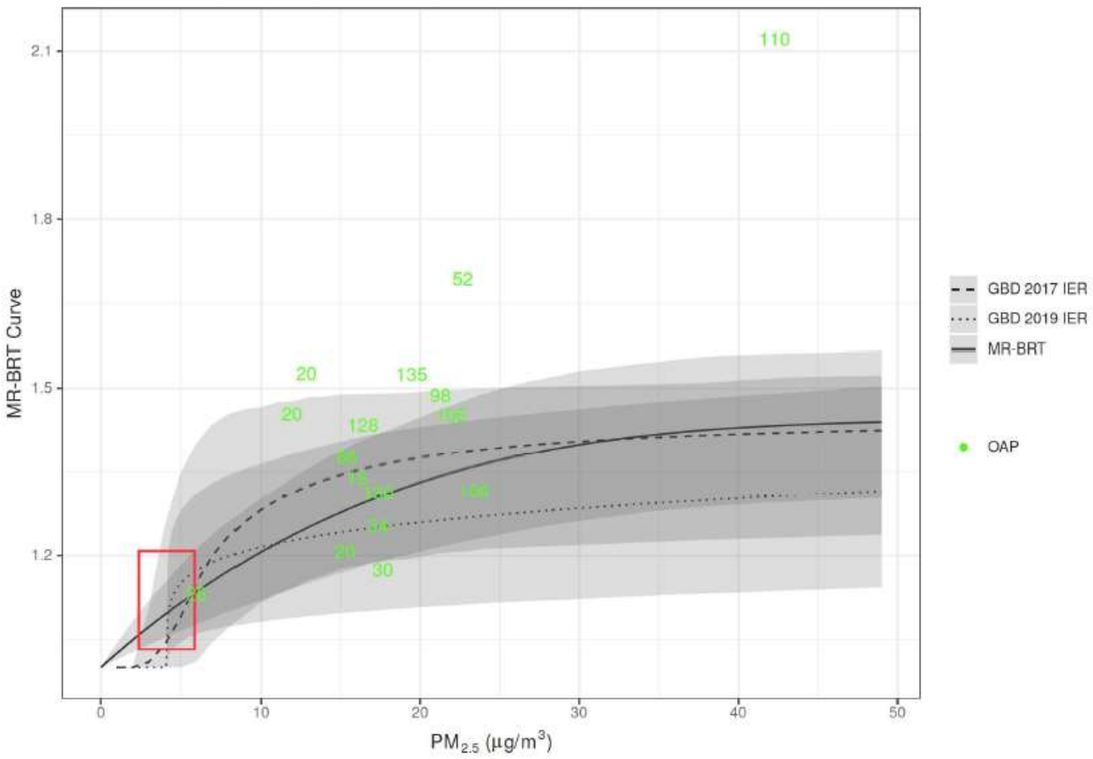

Type 2 Diabetes, Full Exposure Range

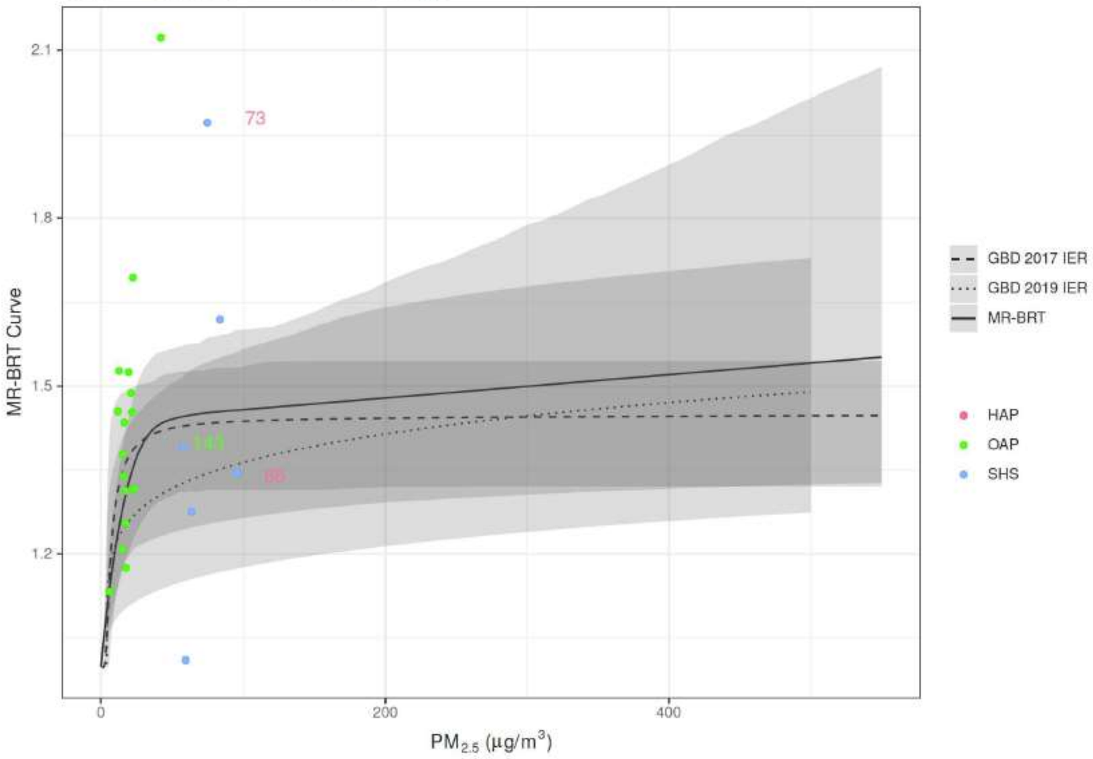

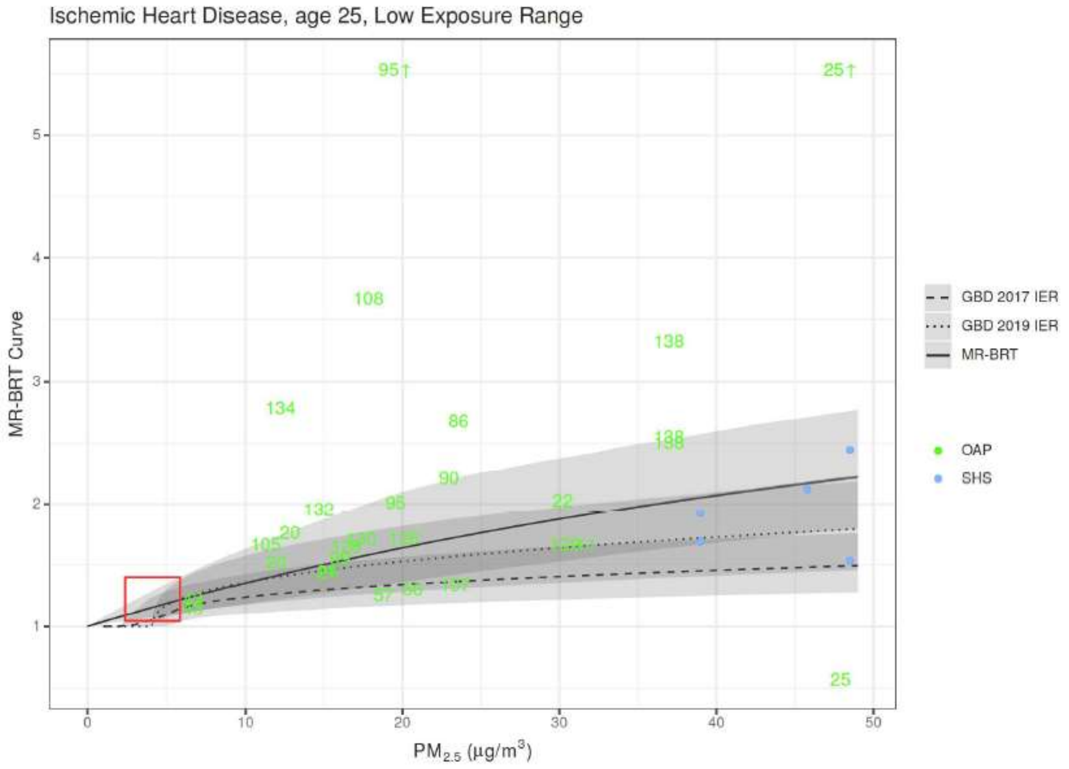
Ischemic Heart Disease, age 25, Low Exposure Range
MR-BRT Curve
PM2.5 (μg/m3)
GBD 2017 IER
GBD 2019 IER
MR-BRT
OAP
SHS

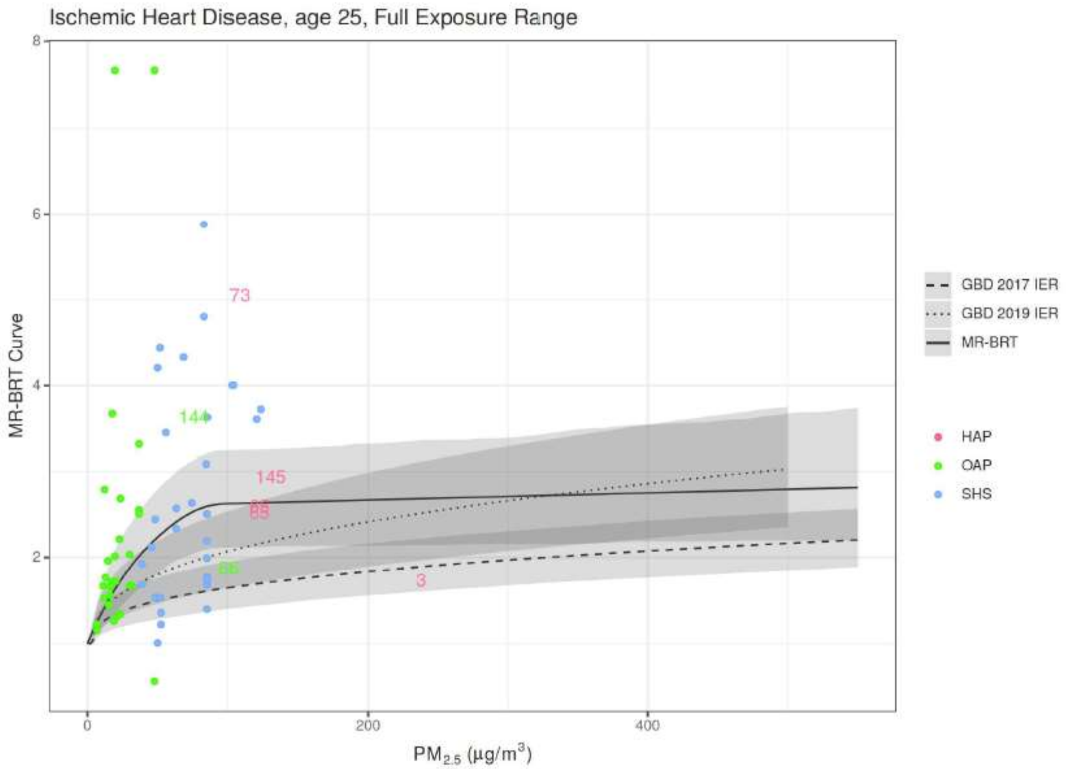
Ischemic Heart Disease, age 25, Full Exposure Range
MR-BRT Curve
PM2.5 (μg/m3)
GBD 2017 IER
GBD 2019 IER
MR-BRT
HAP
OAP
SHS

Ischemic Heart Disease, age 50, Low Exposure Range

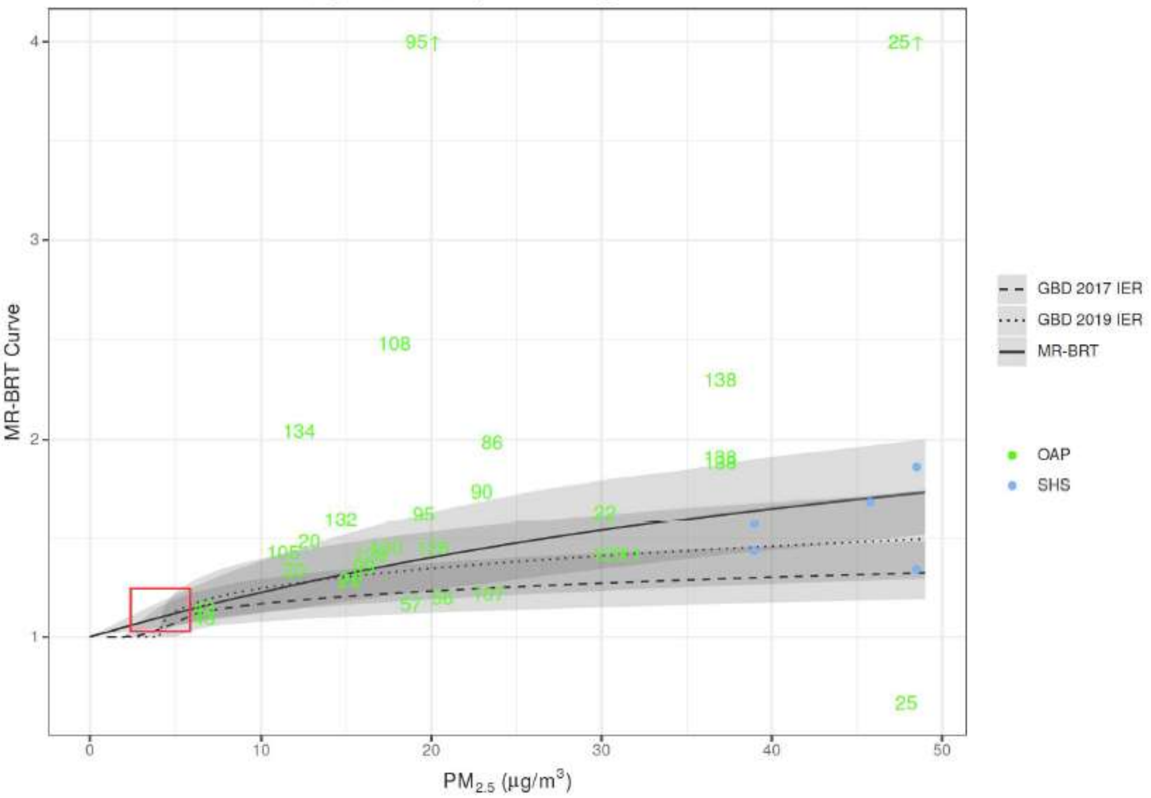

Ischemic Heart Disease, age 50, Full Exposure Range

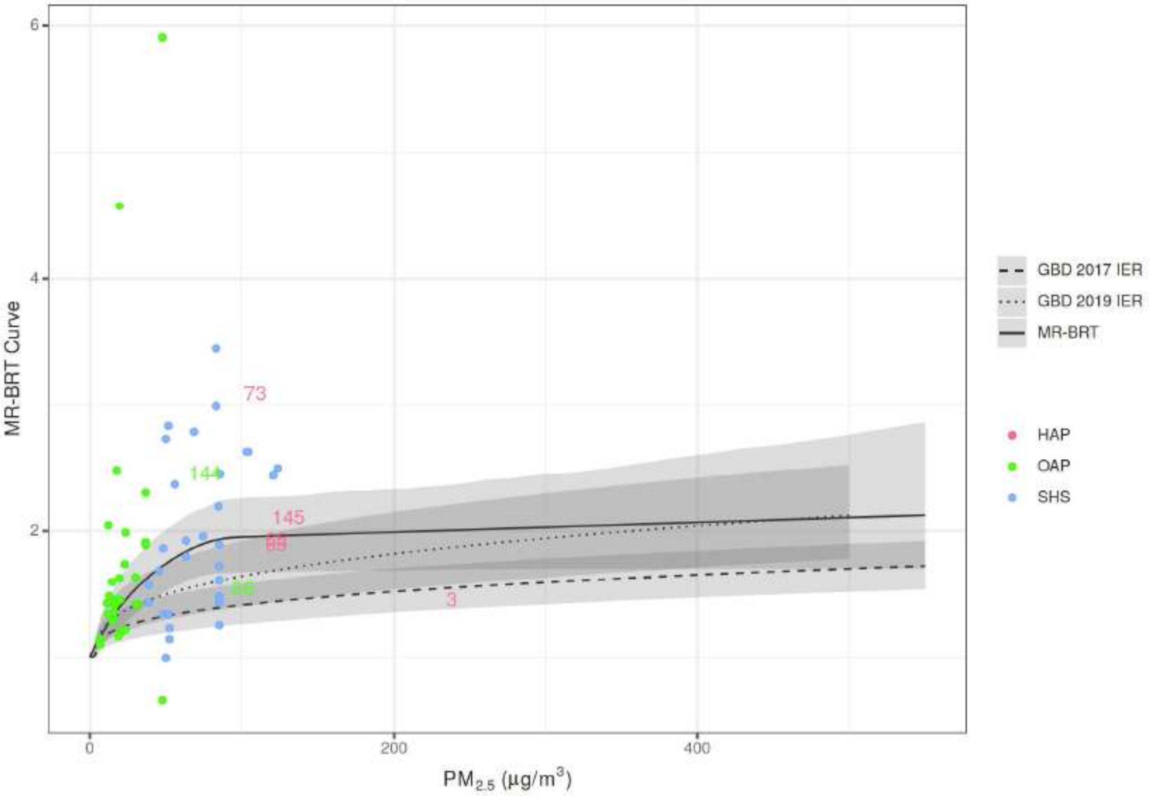

Ischemic Heart Disease, age 80, Low Exposure Range

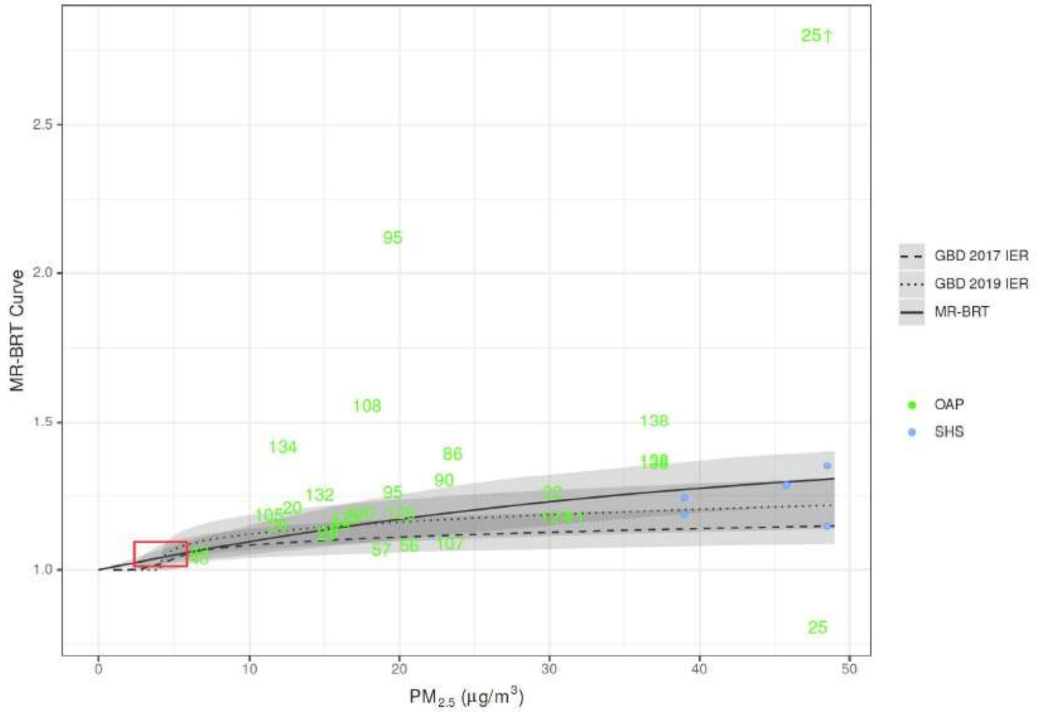

Ischemic Heart Disease, age 80, Full Exposure Range

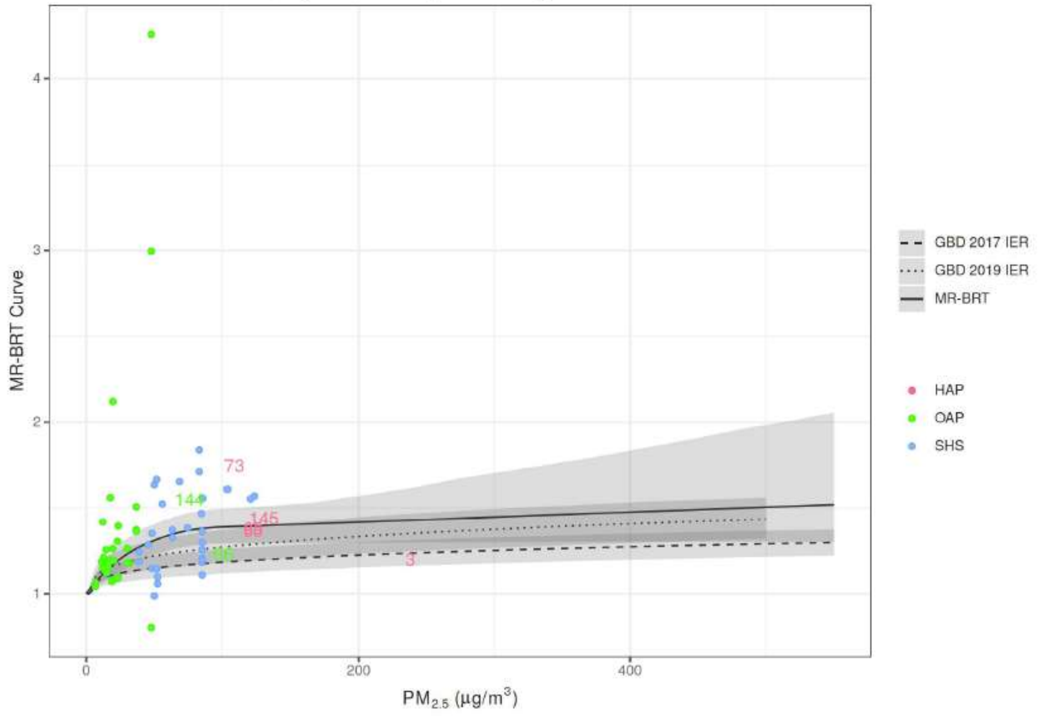

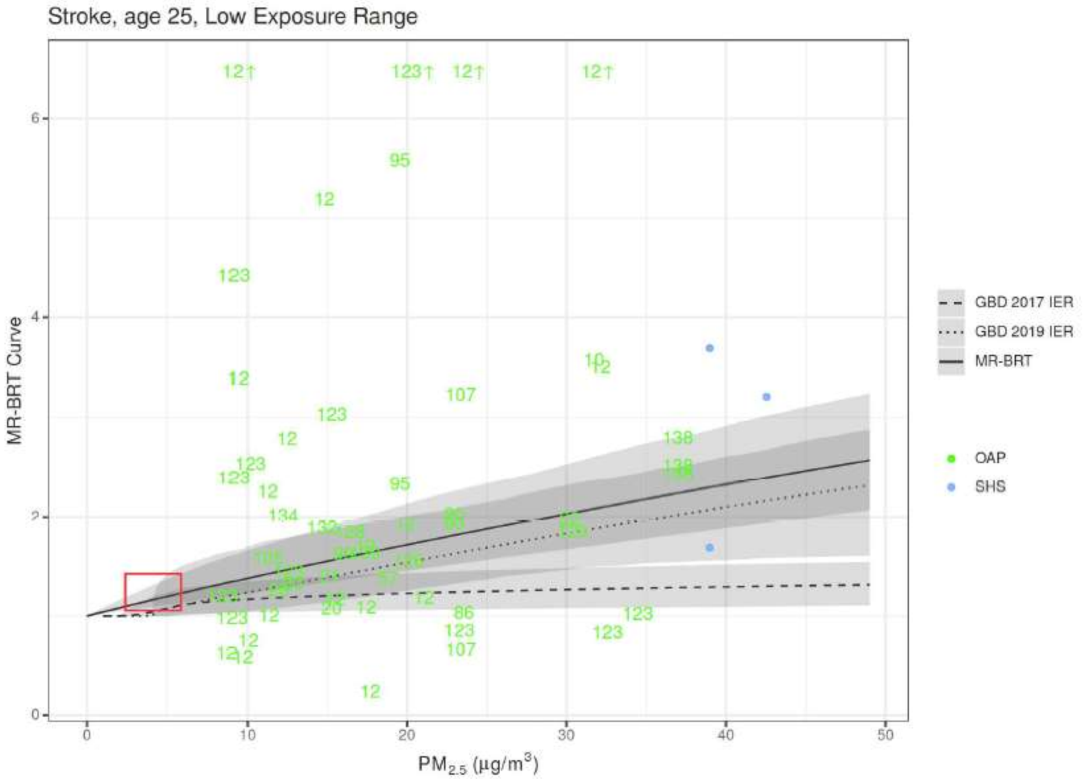
Stroke, age 25, Low Exposure Range
MR-BRT Curve
PM2.5 (µg/m³)
GBD 2017 IER
GBD 2019 IER
MR-BRT
OAP
SHS

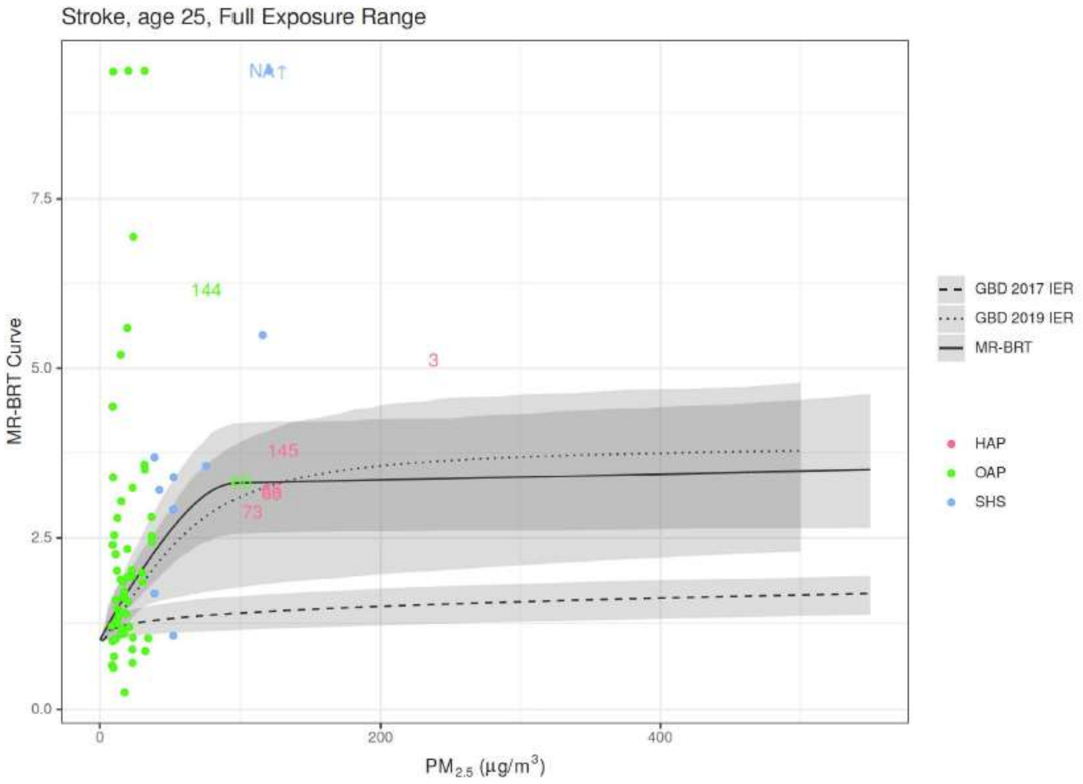
Stroke, age 25, Full Exposure Range
MR-BRT Curve
PM2.5 (µg/m³)
GBD 2017 IER
GBD 2019 IER
MR-BRT
HAP
OAP
SHS

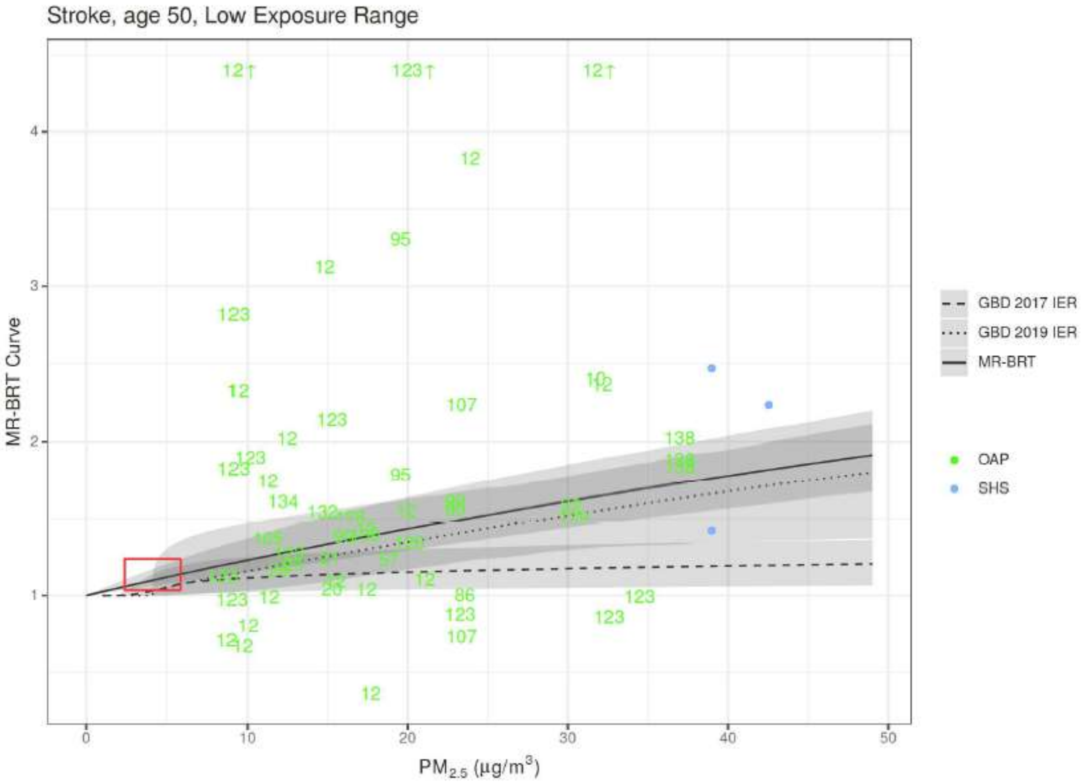

Stroke, age 50, Low Exposure Range
MR-BRT Curve
PM2.5 (μg/m3)
GBD 2017 IER
GBD 2019 IER
MR-BRT
OAP
SHS

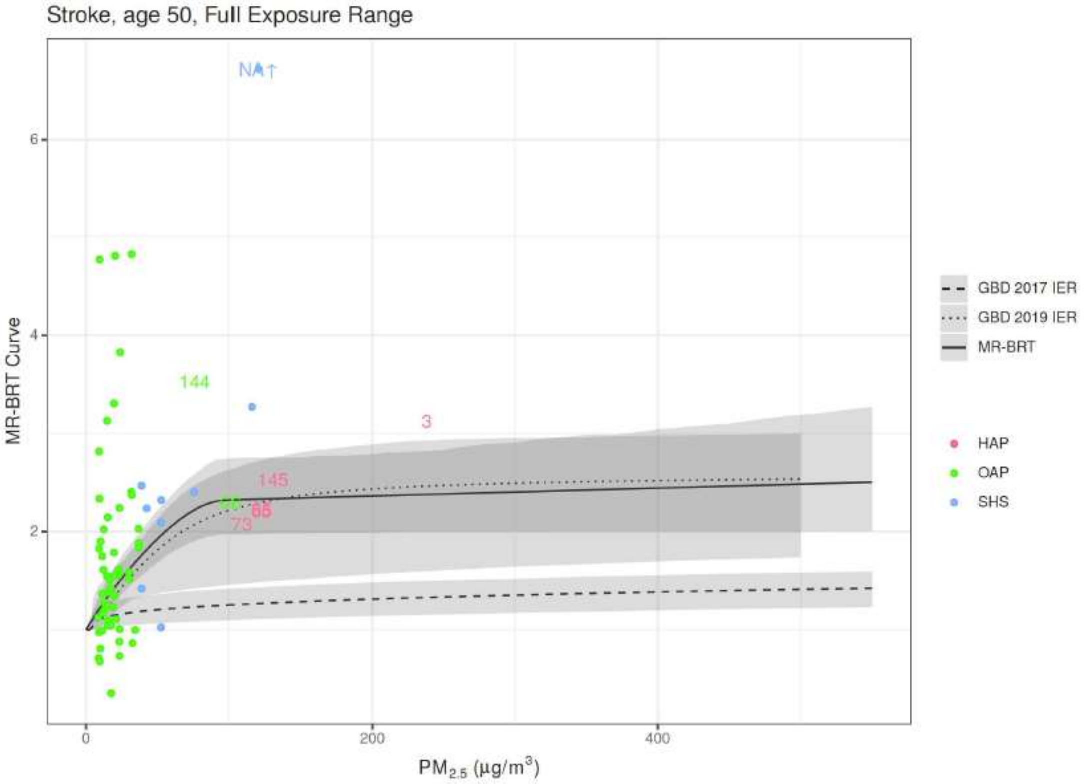

Stroke, age 50, Full Exposure Range
MR-BRT Curve
PM2.5 (μg/m3)
GBD 2017 IER
GBD 2019 IER
MR-BRT
HAP
OAP
SHS

Stroke, age 80, Low Exposure Range

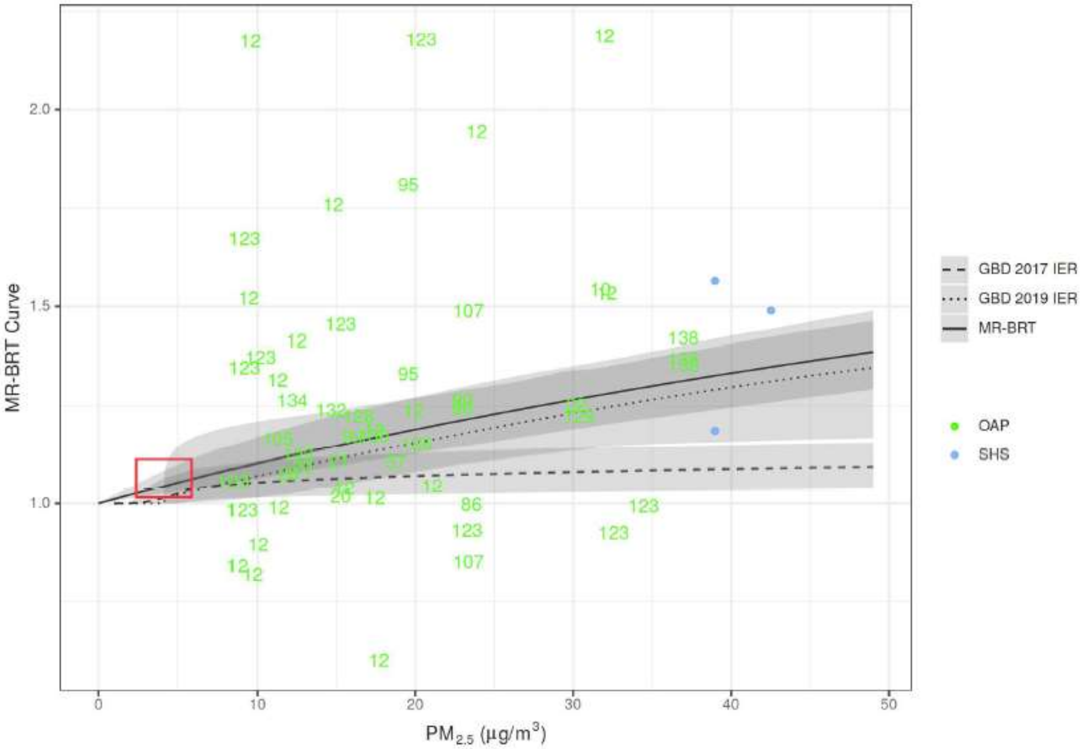

Stroke, age 80, Full Exposure Range

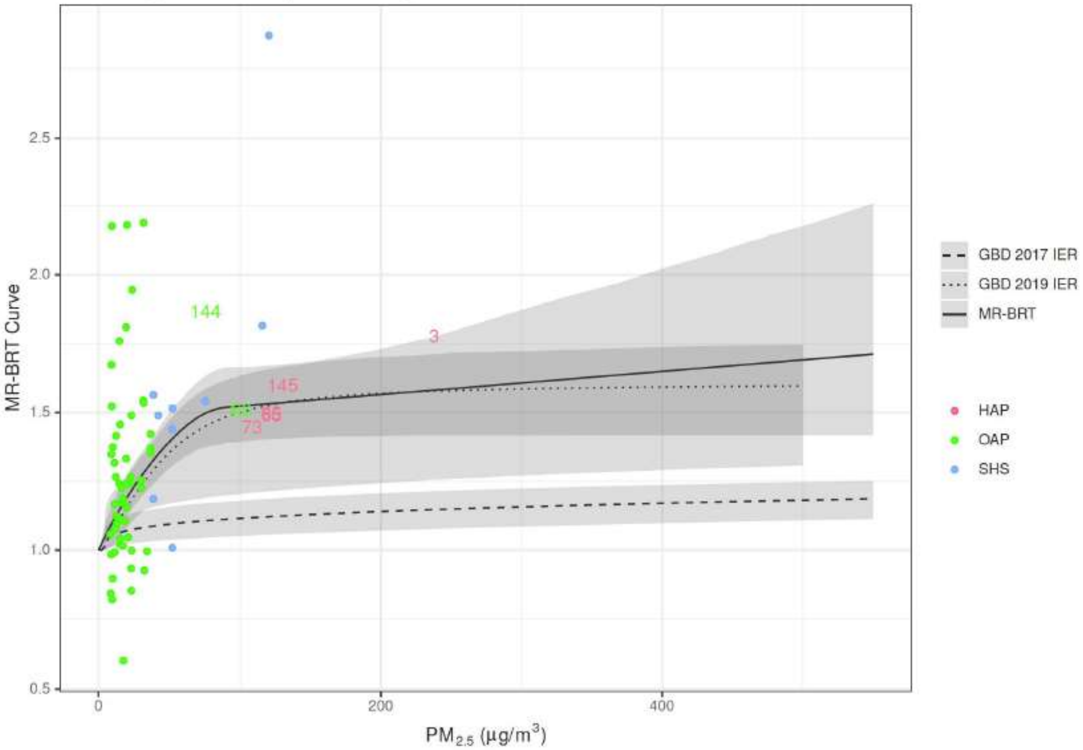

Low birthweight and short gestation mediation analysis

The outcomes of low birthweight and short gestation include mortality due to diarrhoeal diseases, lower respiratory infections, upper respiratory infections, otitis media, meningitis, encephalitis, neonatal preterm birth, neonatal encephalopathy due to birth asphyxia and trauma, neonatal sepsis and other neonatal infections, haemolytic disease and other neonatal jaundice, and other neonatal disorders. We also calculate attributable YLDs for neonatal preterm birth. These are specific to ages 0-6 days and 7-27 days.

In partnership with Dr. Rakesh Ghosh at the University of California, San Francisco, we conducted a systematic review of all cohort, case-control, or randomised-controlled trial studies of ambient $PM_{2.5}$ pollution or household air pollution and birthweight or gestational age outcomes. Outcomes measured included continuous birthweight (bw), continuous gestational age (ga), low birthweight (LBW) (<2500 g), preterm birth (PTB) (<37 weeks), and very preterm birth (VPTB) (<32 weeks). We included any papers published until March 31, 2018. Systematic review PRISMA diagrams are below.

Ambient particulate matter pollution, low birth weight

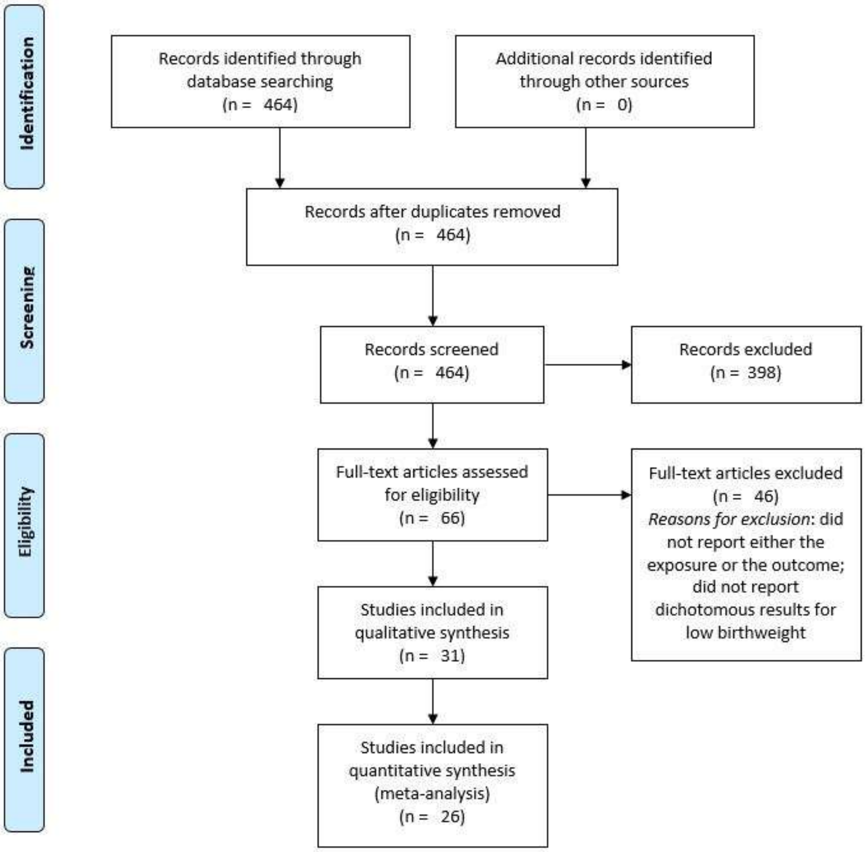

Ambient particulate matter pollution, preterm birth

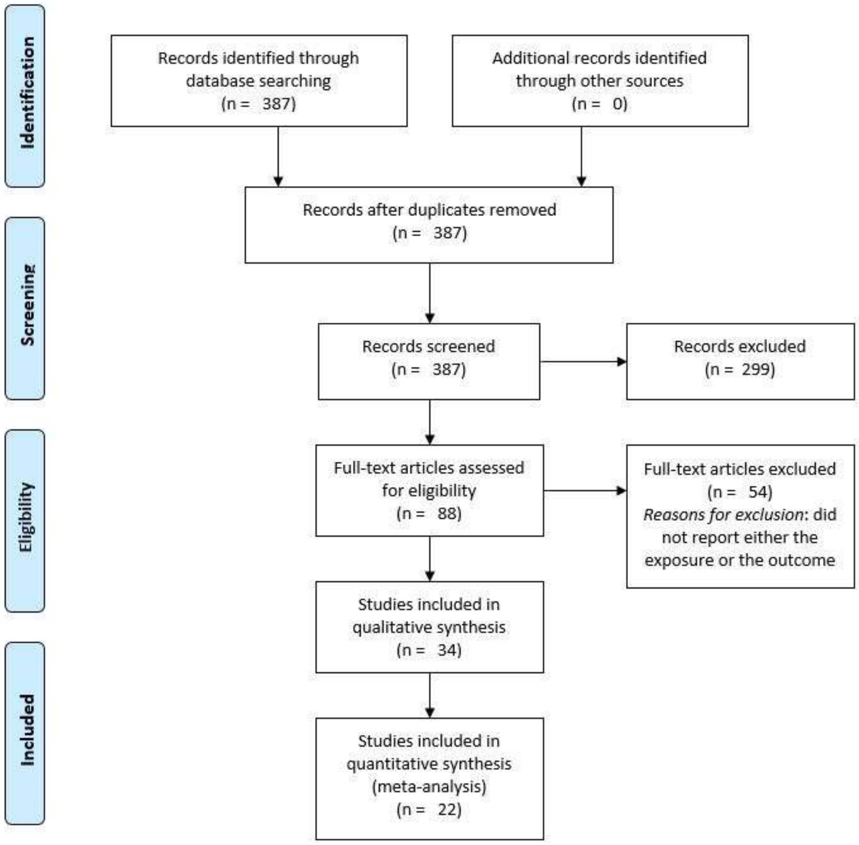

Household air pollution, all outcomes

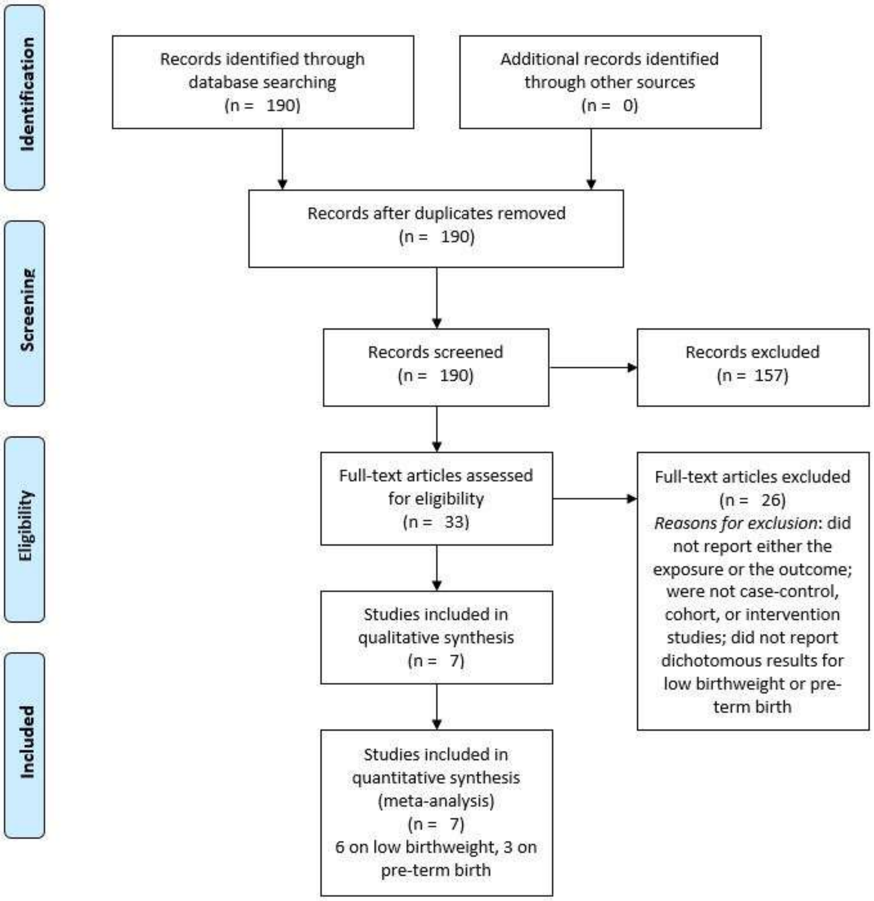

The following plots depict forest and funnel plots for studies of OAP and birthweight, low birthweight, and preterm birth. Note that these plots do not capture the exposure level of these studies but the linear risk or difference in birthweight per 10-unit increase in $PM_{2.5}$ exposure.

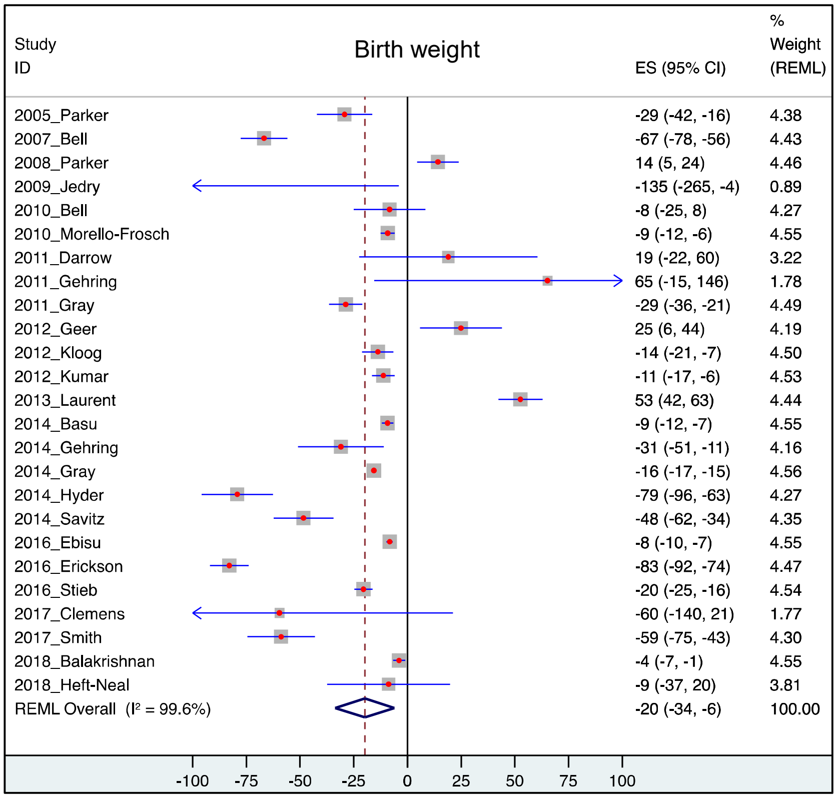
Birth weight
Study ID
ES (95% CI)
% Weight (REML)
2005_Parker -29 (-42, -16) 4.38
2007_Bell -67 (-78, -56) 4.43
2008_Parker 14 (5, 24) 4.46
2009_Jedry -135 (-265, -4) 0.89
2010_Bell -8 (-25, 8) 4.27
2010_Morello-Frosch -9 (-12, -6) 4.55
2011_Darrow 19 (-22, 60) 3.22
2011_Gehring 65 (-15, 146) 1.78
2011_Gray -29 (-36, -21) 4.49
2012_Geer 25 (6, 44) 4.19
2012_Kloog -14 (-21, -7) 4.50
2012_Kumar -11 (-17, -6) 4.53
2013_Laurent 53 (42, 63) 4.44
2014_Basu -9 (-12, -7) 4.55
2014_Gehring -31 (-51, -11) 4.16
2014_Gray -16 (-17, -15) 4.56
2014_Hyder -79 (-96, -63) 4.27
2014_Savitz -48 (-62, -34) 4.35
2016_Ebisu -8 (-10, -7) 4.55
2016_Erickson -83 (-92, -74) 4.47
2016_Stieb -20 (-25, -16) 4.54
2017_Clemens -60 (-140, 21) 1.77
2017_Smith -59 (-75, -43) 4.30
2018_Balakrishnan -4 (-7, -1) 4.55
2018_Heft-Neal -9 (-37, 20) 3.81
REML Overall (I² = 99.6%) -20 (-34, -6) 100.00
-100 -75 -50 -25 0 25 50 75 100

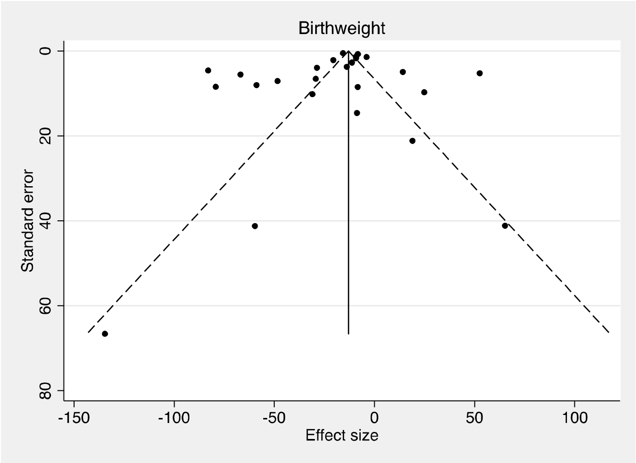
Birthweight
Standard error
0 20 40 60 80
-150 -100 -50 0 50 100
Effect size

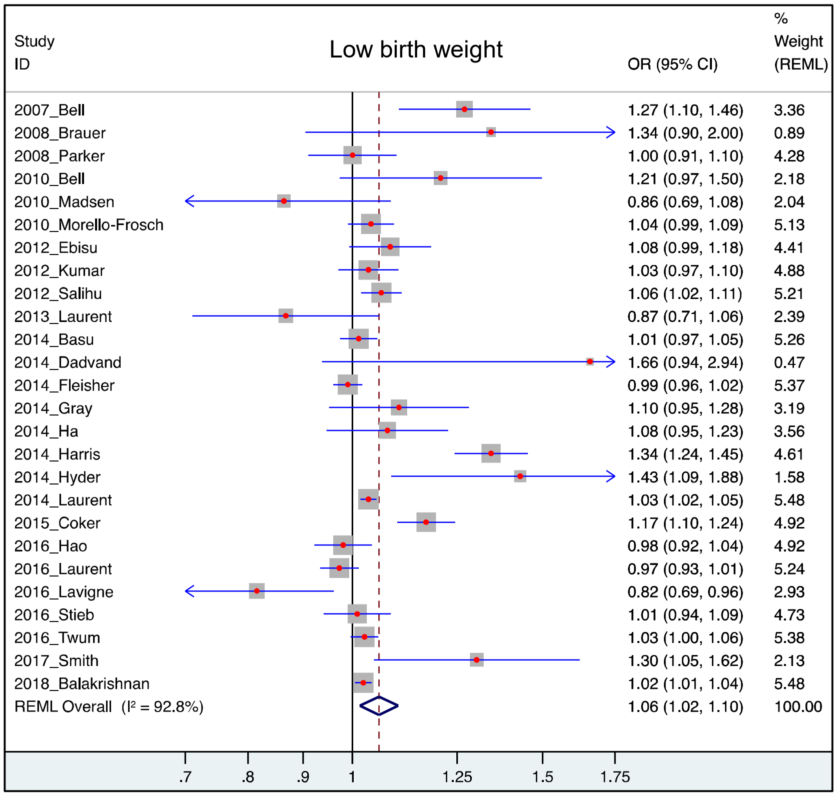

Low birth weight
Study ID
OR (95% CI)
% Weight (REML)
2007_Bell 1.27 (1.10, 1.46) 3.36
2008_Brauer 1.34 (0.90, 2.00) 0.89
2008_Parker 1.00 (0.91, 1.10) 4.28
2010_Bell 1.21 (0.97, 1.50) 2.18
2010_Madsen 0.86 (0.69, 1.08) 2.04
2010_Morello-Frosch 1.04 (0.99, 1.09) 5.13
2012_Ebisu 1.08 (0.99, 1.18) 4.41
2012_Kumar 1.03 (0.97, 1.10) 4.88
2012_Salihu 1.06 (1.02, 1.11) 5.21
2013_Laurent 0.87 (0.71, 1.06) 2.39
2014_Basu 1.01 (0.97, 1.05) 5.26
2014_Dadvand 1.66 (0.94, 2.94) 0.47
2014_Fleisher 0.99 (0.96, 1.02) 5.37
2014_Gray 1.10 (0.95, 1.28) 3.19
2014_Ha 1.08 (0.95, 1.23) 3.56
2014_Harris 1.34 (1.24, 1.45) 4.61
2014_Hyder 1.43 (1.09, 1.88) 1.58
2014_Laurent 1.03 (1.02, 1.05) 5.48
2015_Coker 1.17 (1.10, 1.24) 4.92
2016_Hao 0.98 (0.92, 1.04) 4.92
2016_Laurent 0.97 (0.93, 1.01) 5.24
2016_Lavigne 0.82 (0.69, 0.96) 2.93
2016_Stieb 1.01 (0.94, 1.09) 4.73
2016_Twum 1.03 (1.00, 1.06) 5.38
2017_Smith 1.30 (1.05, 1.62) 2.13
2018_Balakrishnan 1.02 (1.01, 1.04) 5.48
REML Overall (I² = 92.8%) 1.06 (1.02, 1.10) 100.00
.7
.8
.9
1
1.25
1.5
1.75

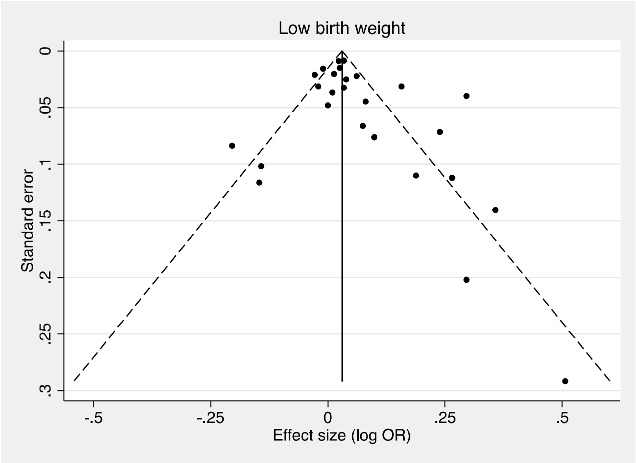

Low birth weight
Standard error
0
.05
.1
.15
.2
.25
.3
-.5
-.25
0
.25
.5
Effect size (log OR)

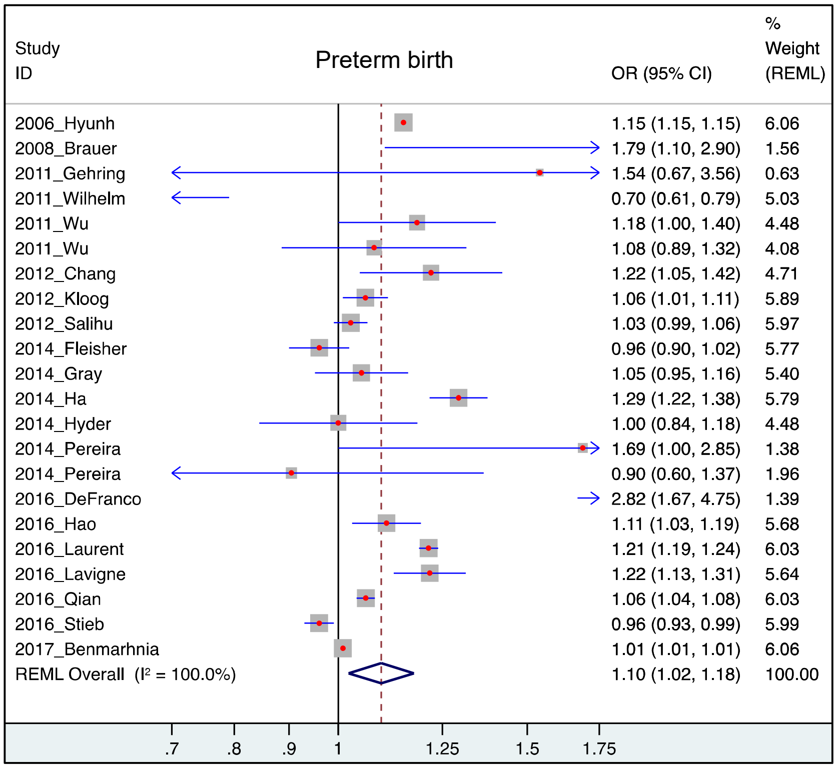

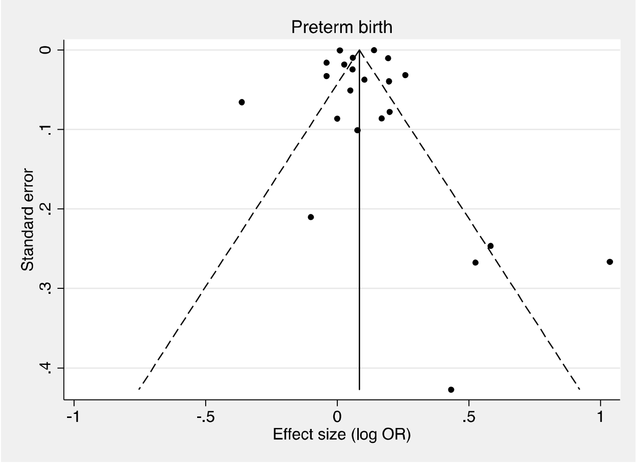

For studies of household air pollution, we used the same strategy described above to map them to $PM_{2.5}$ exposure values.

Because birthweight and gestational age are modelled using a continuous joint distribution for the GBD, we were interested in how those distributions changed under the influence of $PM_{2.5}$ pollution. We therefore estimated the continuous shift in birthweight (bw, in grams) and gestational age (ga, in weeks) at a given $PM_{2.5}$ exposure level.

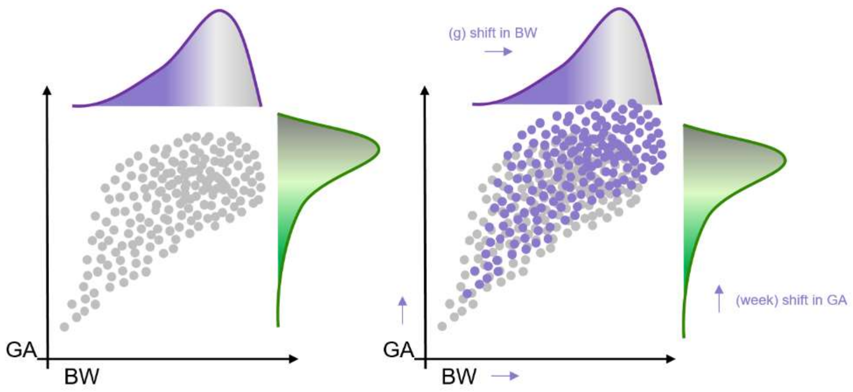

When available, we used estimates of continuous shift in bw or ga directly from each study. When that was not available, we used the published OR/RR/HR for LBW, PTB, or VPTB and the following strategy:

1. Extract the OR/RR/HR from the study.
2. Select the GBD 2017 estimated bw-ga joint distribution for the study location and year.
3. Calculate the number of grams or weeks required to shift the distribution such that the proportion of births under the specified threshold (P) is reduced by the study effect size to a counterfactual level (P_{cf}).
4. Save the resulting shift and 95% CI as the continuous effect.

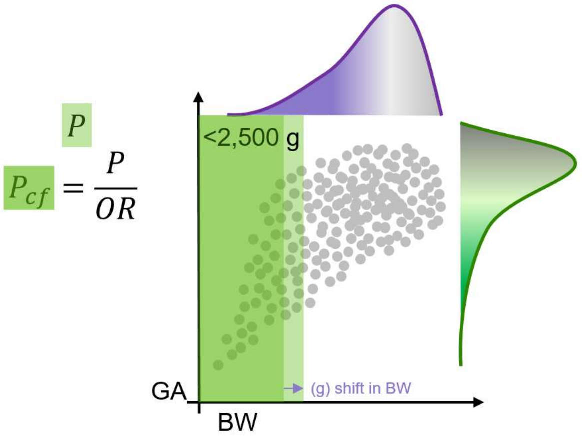

We then fit a MR-BRT spline to these studies, where the difference in the value of the model at the upper concentration (X) and the value of the model at the counterfactual concentration (X_{CF}) is equal to the published or calculated shift in bw or ga. We fit the same model and priors as the non-mediated outcomes (with the exception of COPD), except, because the change in birthweight and gestational age was expected to be negative, the shape constraints were monotonically decreasing and concave up.

$$MRBRT(X) - MRBRT(X_{CF}) \sim Shift$$

The following figures depict the MR-BRT curves for shift in grams (bw) and weeks (ga).

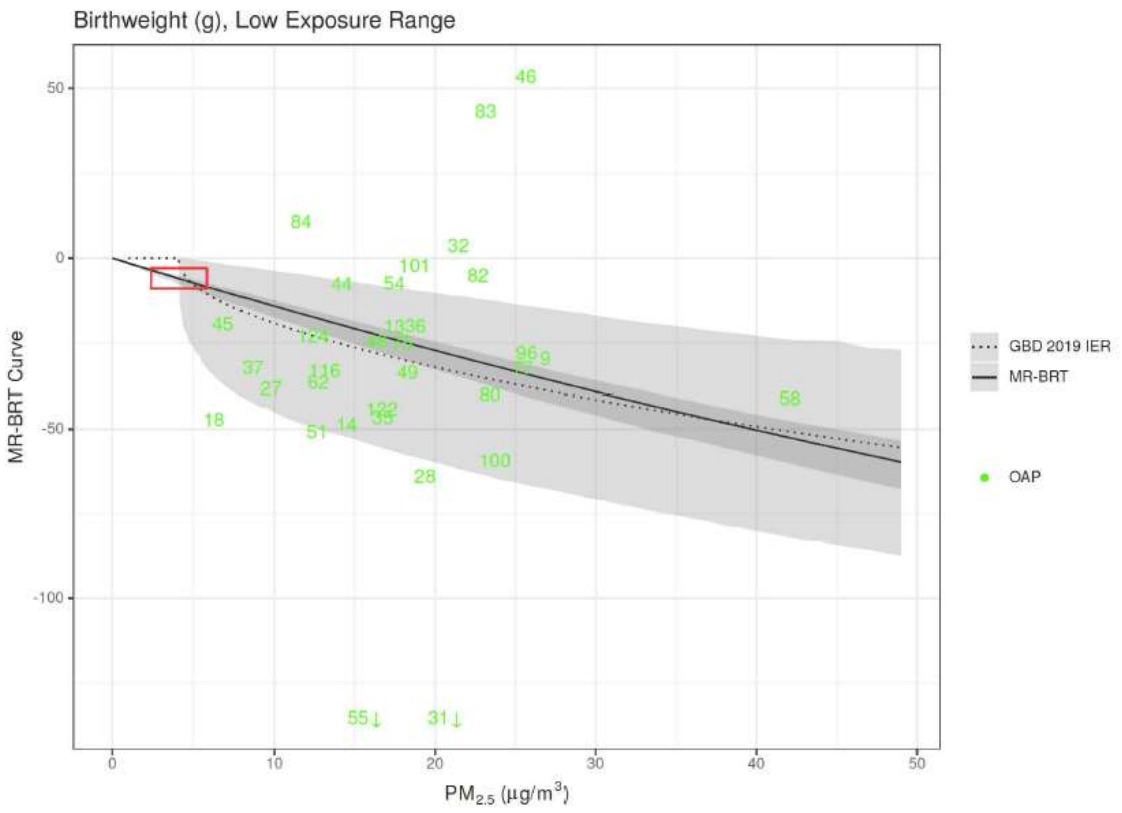
Birthweight (g), Low Exposure Range
MR-BRT Curve
PM$_{2.5}$ (µg/m^3)
GBD 2019 IER
MR-BRT
OAP

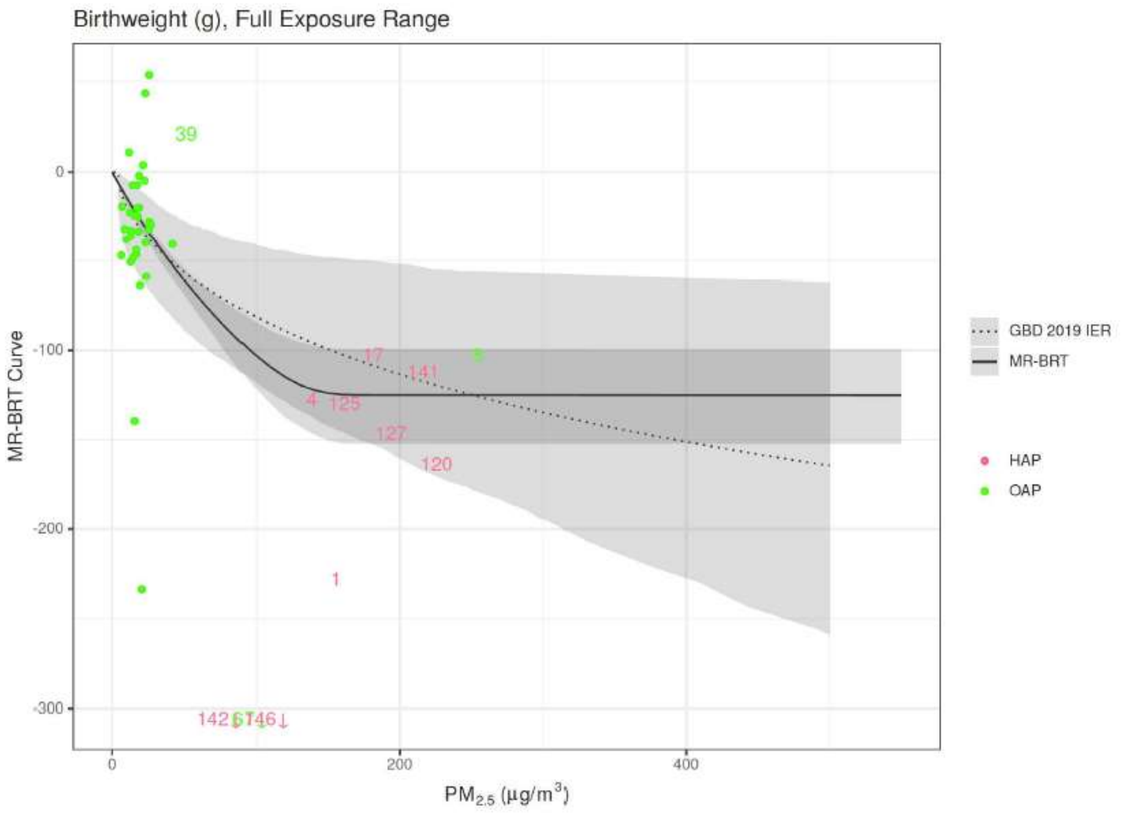
Birthweight (g), Full Exposure Range
MR-BRT Curve
PM$_{2.5}$ (µg/m^3)
GBD 2019 IER
MR-BRT
HAP
OAP

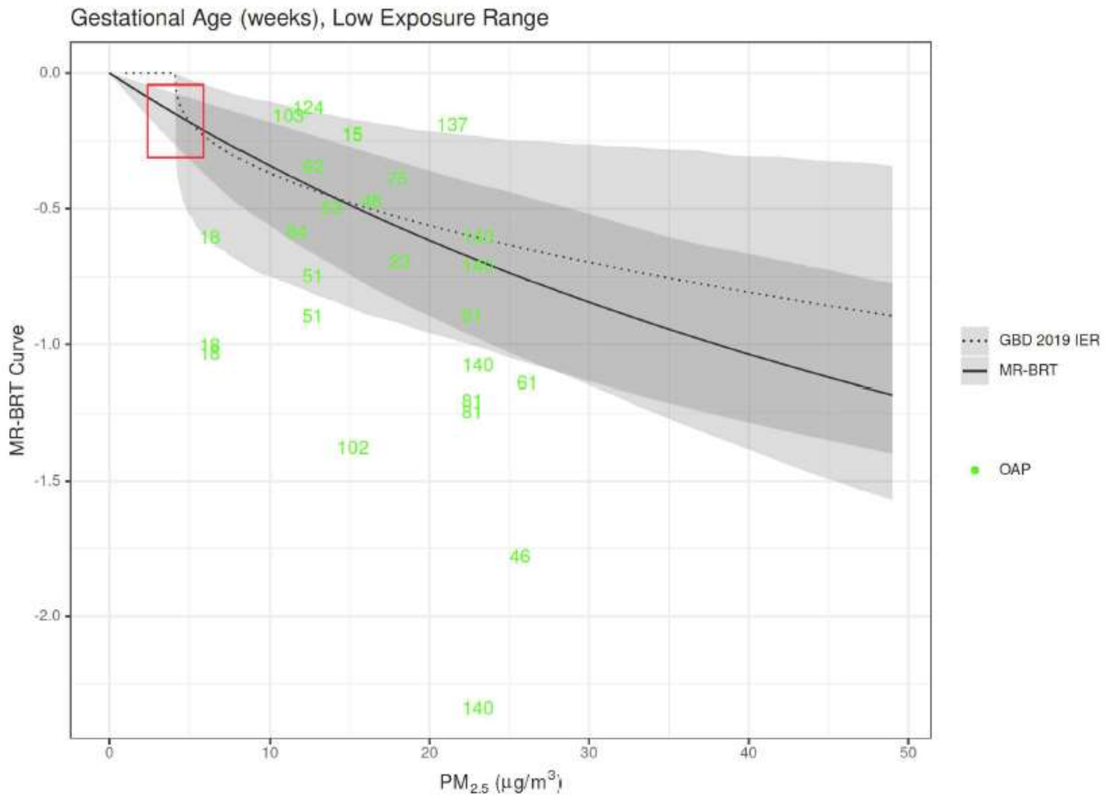
Gestational Age (weeks), Low Exposure Range
MR-BRT Curve
0.0
-0.5
-1.0
-1.5
-2.0
0
10
20
30
40
50
PM2.5 (µg/m3)
GBD 2019 IER
MR-BRT
OAP

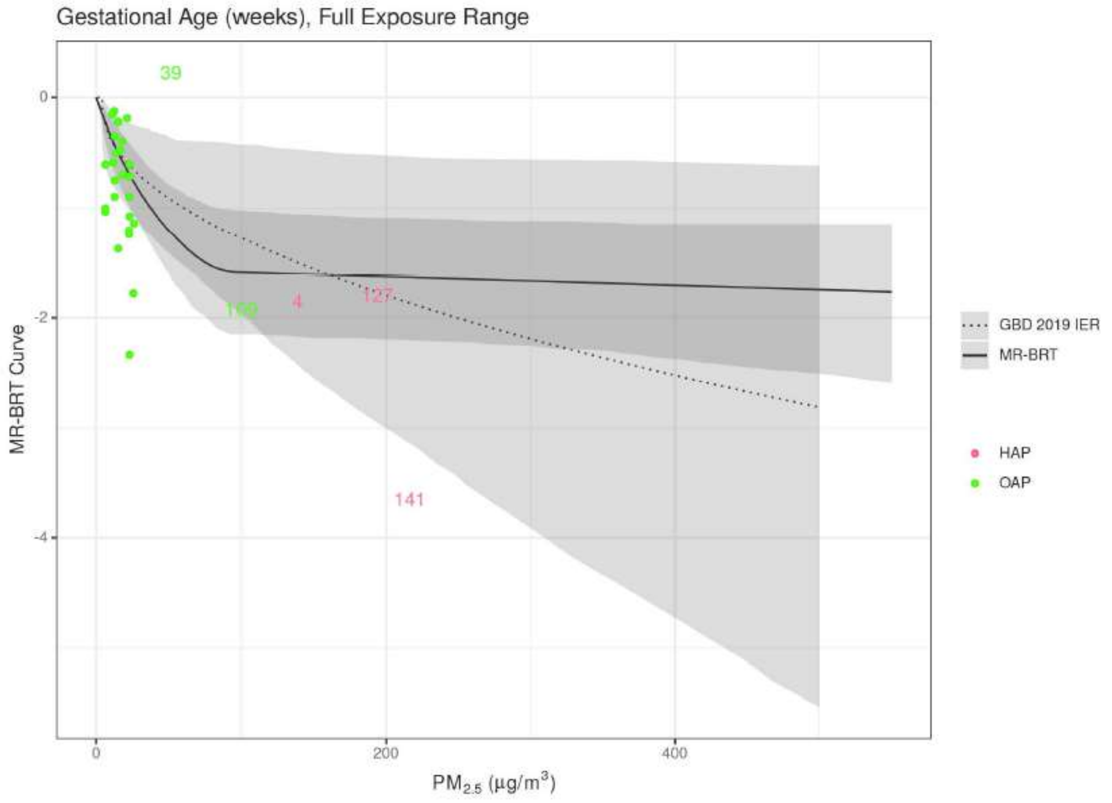
Gestational Age (weeks), Full Exposure Range
MR-BRT Curve
0
-2
-4
0
200
400
PM2.5 (µg/m3)
GBD 2019 IER
MR-BRT
HAP
OAP

Once we had curves of estimated shifts across the exposure range, we predicted the shift in both birthweight and gestational age for total female particulate matter pollution exposure in each location and year. Because the epidemiological studies mutually controlled for birthweight and gestational age, we assumed these shifts are independent. We then shifted the observed distributions to reflect the expected bwga distribution in the absence of particulate matter pollution. These shifted distributions were used as the counterfactual in the PAF calculation equation to calculate the burden attributable to $PM_{2.5}$ pollution.

To calculate PAFs, the distribution is divided into 56 bw-ga categories, each with a unique RR. Let p_i be the observed proportion of babies in category, *i* and p_i' be the counterfactual proportion of babies in category, *i* if there were no particulate matter pollution.

$$PAF_{PM} = \frac{\sum_{i \in bwga\ category} RR_i p_i - \sum_{i \in bwga\ category} RR_i p_i'}{\sum_{i \in bwga} RR_i p_i}$$

We proportionately split this PAF to ambient and HAP based on exposure as is described below. One important assumption to note is that we are assuming the shift in bw and ga is linear across the bwga distribution.

For lower respiratory infections, we have directly estimated PAFs attributable to $PM_{2.5}$ in addition to those mediated through birthweight and gestational age. We would expect that some of the directly estimated PAFs are mediated through bw and ga. Additionally, the directly estimated PAF is based on a summary of relative risks for all children under 5 years, so there is a chance that the mediated PAF, which is more finely resolved, could be greater. To avoid double-counting for these two age groups (0-6 days and 0-27 days), we take the max of the two PAF estimates. If the directly estimated PAF is greater than the bw-ga-mediated PAF, we take the direct estimate, and if the mediated PAF is greater, we take the mediated.

PTB incidence and mortality are both outcomes measured in the GBD. 100% of the burden for this cause is attributable to short gestation. To calculate the percentage attributable to particulate matter pollution, we estimated the percentage of babies born at less than 37 weeks (p_{ptb}) and the percentage of babies that would have been born at less than 37 weeks in the counterfactual scenario of no particulate matter pollution (p_{ptb}').

$$PAF_{ptb,pm} = 1 - \frac{p_{ptb}'}{p_{ptb}}$$

Limitations

Although in GBD 2019 we have not used active smoking data to estimate the risk curves, we are still using an integrated exposure response approach because we are integrating relative risk estimates across various exposure sources: ambient, SHS, and HAP. The use of various sources to construct a risk curve with $PM_{2.5}$ as the exposure indicator assumes equitoxicity of particles, despite some evidence suggesting differences in health impact by PM source, size, and chemical composition. However, in the absence of consistent and robust evidence of differential toxicity by source and sufficient estimates of source or composition-specific exposure-response relationships, integrating across OAP, SHS, and HAP studies is the approach most consistent with the current evidence, as reviewed by US EPA and WHO.[20,21] Use of a common risk function may affect the magnitude of risk estimates for HAP and OAP compared to

separate risk functions. As more data from higher OAP concentration locations and from HAP studies for non-respiratory outcomes becomes available it may be possible to evaluate the strength of evidence for each and to develop separate risk functions.

Proportional PAF approach

Prior to GBD 2017, relative risks for both exposures were obtained from the IER as a function of exposure and relative to the same TMREL. In reality, were a country to reduce only one of these risk factors, the other would remain. We did not consider the joint effects of particulate matter from outdoor exposure and burning solid fuels for cooking. For GBD 2017 we developed a new approach to use the IER for obtaining PAFs for both OAP and HAP:

Let Exp_{OAP} be the ambient $PM_{2.5}$ exposure level and Exp_{HAP} be the excess exposure for those who use solid fuel for cooking. Let P_{HAP} be the proportion of the population using solid fuel for cooking. We calculated PAFs at each 0.1°×0.1° grid cell. We assumed that the distribution of those using solid fuel for cooking (HAP) was equivalent across all grid cells of the GBD location.

For the proportion of the population not exposed to HAP the relative risk was:

$$RR_{OAP} = MRBRT(z = Exp_{OAP})/MRBRT(z = TMREL),$$

And for those exposed to HAP, the relative risk was

$$RR_{HAP} = MRBRT(z = Exp_{OAP} + Exp_{HAP})/MRBRT(z = TMREL).$$

We then calculate a population level RR and PAF for all particulate matter exposure.

$$RR_{PM} = RR_{OAP}(1 - P_{HAP}) + RR_{HAP}P_{HAP}$$

$$PAF_{PM} = \frac{RR_{PM} - 1}{RR_{PM}}$$

We population weight the grid-cell level particulate matter PAFs to get a country level PAF, and finally, we split this PAF based on the average exposure to each OAP and HAP.

$PAF_{OAP} = \frac{Exp_{OAP}}{Ex_{OAP} + P_{HAP} * Exp_{HAP}} PAF_{PM}$, and $PAF_{HAP} = \frac{P_{HAP} * Ex_{HAP}}{Exp_{OAP} + P_{HAP} * Exp_{HAP}} PAF_{PM}$.

With this strategy, $PAF_{PM} = PAF_{HAP} + PAF_{OAP}$, and no burden is counted twice.

References

1. Hammer, M. S., A. van Donkelaar, R. V. Martin, C. Li, A. Lyapustin, A. M. Sayer, C. N. Hsu, R. C. Levy, M. J. Garay, O. V. Kalashnikova, R. A. Kahn, M. Brauer, J. S. Apte, D. K. Henze, L. Zhang, and Q. Zhang (submitted), Improved Global Estimates of Fine Particulate Matter Concentrations and Trends Derived from Updated Satellite Retrievals, Modeling Advances, and Additional Ground-Based Monitors, *Environ. Sci. Technol*.
2. van Donkelaar, A.; Martin, R. V; Brauer, M.; Hsu, N. C.; Kahn, R. A.; Levy, R. C.; Lyapustin, A.; Sayer, A. M.; Winker, D. M. Global Estimates of Fine Particulate Matter using a Combined Geophysical-Statistical Method with Information from Satellites, Models, and Monitors. Environ. Sci. Technol. 2016, 50 (7), 3762–3772
3. Shaddick, G., Thomas, M.L., Jobling, A., Brauer, M., van Donkelaar, A., Burnett, R., Chang, H., Cohen, A., Van Dingenen, R., Dora, C. and Gumy, S., 2016. Data Integration Model for Air

Quality: A Hierarchical Approach to the Global Estimation of Exposures to Ambient Air Pollution. Journal of Royal Statistical Society Series C (Applied Statistics). 2017. DOI: 10.1111/rssc.12227

4. Shaddick, G., Thomas, M. L., Mudu, P., Ruggeri, G. and Gumy, S. Half the world's population are exposed to increasing air pollution. Accepted by Nature Climate and Atmospheric Science.
5. Brauer, M.; Freedman, G.; Frostad, J.; van Donkelaar, A.; Martin, R. V; Dentener, F.; Van Dingenen, R.; Estep, K.; Amini, H.; Apte, J. S.; et al. Ambient Air Pollution Exposure Estimation for the Global Burden of Disease 2013. Environ. Sci. Technol. 2015, 50 (1), 79–88.
6. Shaddick G, Thomas M, Amini H, Broday DM, Cohen A, Frostad J, Green A, Gumy S, Liu Y, Martin RV, Prüss-Üstün A, Simpson D, van Donkelaar A, Brauer M. Data integration for the assessment of population exposure to ambient air pollution for global burden of disease assessment. Environ Sci Technol. 2018 Jun 29. doi: 10.1021/acs.est.8b02864
7. Rue, H.; Martino, S.; Chopin, N.; Approximate Bayesian inference for latent Gaussian models by using integrated nested Laplace approximations. Journal of the royal statistical society: Series b (statistical methodology). 2009;71(2):319-92.
8. Thomas, M. L., Shaddick, G., Simpson, D., de Hoogh, K. and Zidek, J. V. Spatio-temporal downscaling for continental–scale estimation of air pollution concentrations. arXiv preprint arXiv:1907.00093 (also been Submitted to the Journal of the Royal Statistical Society: Series C (Applied Statistics)).
9. Wood, S. N. (2017). Generalized additive models: an introduction with R. Chapman and Hall/CRC.
10. Turner MC,Jerrett M, Pope CA 3rd, Krewski D, Gapstur SM, Diver WR, Beckerman BS, Marshall JD, Su J, Crouse DL, Burnett RT. Long-term ozone exposure and mortality in a large prospective study . Am J Respir Crit Care Med. 2016; 193(10): 1134-42.
11. Yin P, Brauer M, Cohen A, et al. Long-term Fine Particulate Matter Exposure and Nonaccidental and Cause-specific Mortality in a Large National Cohort of Chinese Men. Environ Health Perspect 2017; 125: 117002.
12. Li T, Zhang Y, Wang J, et al. All-cause mortality risk associated with long-term exposure to ambient PM2·5 in China: a cohort study. Lancet Public Health 2018; 3: e470–7.
13. Yang Y, Tang R, Qiu H, et al. Long term exposure to air pollution and mortality in an elderly cohort in Hong Kong. Environ Int 2018; 117: 99–106.
14. Hystad P, Larkin A, Rangarajan S, AlHabib KF, Avezum A, Tumerdem Calik KB; Chifamba J, Dans A, Diaz R, du Plessis JL, Gupta R, Iqbal R, Khatib R, Kelishadi R, Lanas F, Liu Z, Lopez-Jaramillo P, Nair S, Poirier P, Rahman O, Rosengren A, Swidan H, Tse L-A, Wei L, Wielgosz A, Yeates K, Yusoff K, Zatoński T, Yusuf S, Brauer M. Outdoor fine particulate matter air pollution and cardiovascular disease: Results from 747 communities across 21 countries in the PURE Study. (Submitted to Lancet Global Health)
15. Joseph P, Rangarajan S, Islam S, Mente A, Hystad P, Brauer M, Raman Kutty V, Gupta R, Wielgosz A, AlHabib KF, Dans A, Lopez-Jaramillo P, Avezum A, Lanas F, Oguz A, Kruger IM, Diaz R, Yusoff K, Mony P, Chifamba J, Yeates K, Kelishadi R, Yusufali A, Khatib R, Rahman O, Zatonska K, Iqbal R, Wei L, Bo H, Rosengren A, Kaur M, Mohan V, Lear SA, Teo KK, O'Donnell M, McKee M, Dagenais G, Yusuf S. Modifiable risk factors, cardiovascular disease and mortality in 155,722 individuals from 21 high-, middle-, and low-income countries (PURE): a prospective cohort study. The Lancet. 2019. doi:10.1016/S0140-6736(19)32008-2
16. Burnett RT, Pope CA 3rd, Ezzati M, Olives C, Lim SS, Mehta S, Shin HH, Singh G, Hubbell B, Brauer M, Anderson HR, Smith KR, Balmes JR, Bruce NG, Kan H, Laden F, Prüss-Ustün A, Turner MC, Gapstur SM, Diver WR, Cohen A. An integrated risk function for estimating the global burden of

disease attributable to ambient fine particulate matter exposure. *Environ Health Perspect*. 2014; 122(4): 397-403.

17. Pope CA III, Cohen AJ, Burnett RT. Cardiovascular Disease and Fine Particulate Matter: Lessons and Limitations of an Integrated Exposure Response Approach. Circulation Research. 2018;122:1645-1647.
18. Lind L, Sundström J, Ärnlöv J, Lampa E. Impact of Aging on the Strength of Cardiovascular Risk Factors: A Longitudinal Study Over 40 Years. *J Am Heart Assoc.* 2018;7(1):e007061. Published 2018 Jan 6. doi:10.1161/JAHA.117.007061
19. Semple S, Apsley A, Ibrahim TA, Turner SW, Cherrie JW. Fine particulate matter concentrations in smoking households: just how much secondhand smoke do you breathe in if you live with a smoker who smokes indoors? *Tob Control* 2015; 24: e205–11.
20. US Environmental Protection Agency. Integrated science assessment (ISA) for particulate matter (Final Report, Dec 2009). EPA/600/R-08/139F, 2009. Washington, DC: US Environmental Protection Agency; 2009. Available at: http://cfpub.epa.gov/ncea/risk/recordisplay.cfm?deid=216546
21. World Health Organization. Review of evidence on health aspects of air pollution – REVIHAAP Project technical report. Copenhagen: WHO Regional Office for Europe; 2013. Available at: http://www.euro.who.int/__data/assets/pdf_file/0004/193108/REVIHAAP-Final-technical-report-final-version.pdf?ua=1

Household air pollution

Household Air Pollution from Solid Fuels

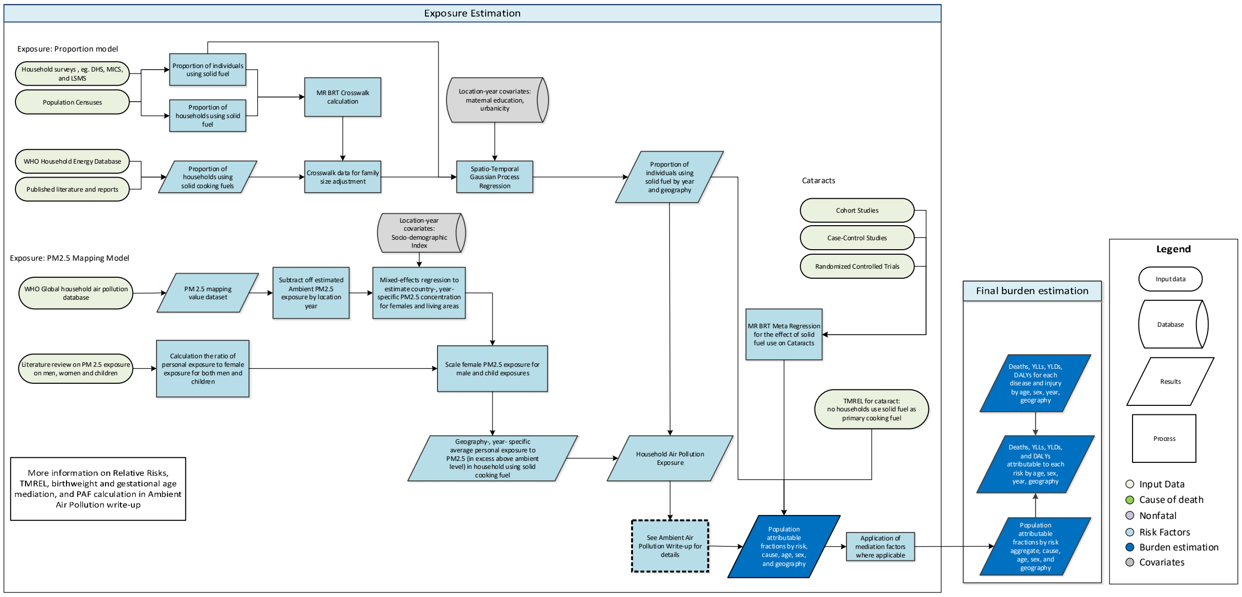

Input data and methodological summary

Exposure

Case definition

Exposure to household air pollution from solid fuels (HAP) is estimated from both the proportion of individuals using solid cooking fuels and the level of $PM_{2.5}$ air pollution exposure for these individuals. Solid fuels in our analysis include coal, wood, charcoal, dung, and agricultural residues.

Input data

We extracted information on use of solid fuels from the standard multi-country survey series such as Demographic and Health Surveys (DHS), Living Standards Measurement Surveys (LSMS), Multiple Indicator Cluster Surveys (MICS), and World Health Surveys (WHS), as well as censuses and country-specific survey series such as Kenya Welfare Monitoring Survey and South Africa General Household Survey. To fill the gaps of data in surveys and censuses, we also downloaded and updated estimates from WHO Energy Database and extracted from literature through systematic review. Each nationally or subnationally representative datapoint provided an estimate for the percentage of households using solid cooking fuels. We used studies from 1980 to 2019 to inform the time series.

We also excluded sources that did not distinguish specific primary fuel types, estimated fuel used for purposes other than cooking (eg, lighting or heating), failed to report standard error or sample size, had over 15% of households with missing responses, reported fuel use in physical units, or were secondary sources referencing primary analyses. Table 1 summarizes exposure input data.

Table 1: Exposure Input Data

Input data	Exposure
Source count (total)	1680
Number of countries with data	195

Family size crosswalk

Many estimates in the WHO Energy Database and other reports quantify the proportion of households using solid fuel for cooking; however, we are interested in the proportion of individuals using solid fuel for cooking. To crosswalk these estimates, whenever we had the available information, we extracted fuel use at both the individual and household levels. We included 3676 source-specific pairs in the MR-BRT crosswalk model.

MR-BRT crosswalk adjustment factors for household air pollution exposure

Data input	Reference or alternative case definition	Gamma	Beta coefficient, logit (95% CI)
Proportion of individuals	Ref	0.097	---
Proportion of Households	Alt		-0.095 (-0.100, -0.090)

We then apply this coefficient to household-only reports with the following formula:

$prop_{individ}$ = the proportion of individuals using solid fuel for cooking, and

$prop_{hh}$ = the proportion of households using solid fuel for cooking.

$$\log\left(\frac{prop_{individ}}{1 - prop_{individ}}\right) = \log\left(\frac{prop_{hh}}{1 - prop_{hh}}\right) - \beta$$

or

$$prop_{individ} = \frac{prop_{hh} * e^{-\beta}}{1 - prop_{hh} + prop_{hh} * e^{-\beta}}$$

The effect is that the household studies are inflated to account for bias. Larger households are more likely to use solid fuel for cooking. The following figure depicts the 3676 data points that informed the crosswalk model. There the red points indicate the 10% of studies that were trimmed as outliers.

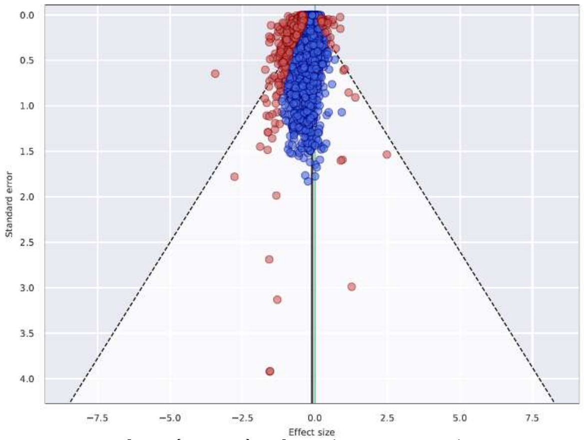

$logit(prop_{hh}) - logit(prop_{individual})$

Modelling strategy

Household air pollution was modelled at individual level using a three-step modelling strategy that uses linear regression, spatiotemporal regression, and Gaussian process regression (GPR). The first step is a mixed-effect linear regression of logit-transformed proportion of individuals using solid cooking fuels. The linear model contains maternal education and the proportion of population living in urban areas as covariates and has nested random effects by GBD region and GBD super-region. The full ST-GPR process is specified elsewhere this appendix. No substantial modelling changes were made in this round compared to GBD 2017.

First-stage linear model and coefficients

$$logit(proportion) \sim maternal\ education + urbanicity + (1|region) + (1|super - region)$$

Variable	Beta (95% CI)
Intercept	3.16 (1.59, 4.74)
Maternal education (years per capita)	-0.45 (-0.76, -0.15)

Urbanicity (proportion of population living in urban areas)	-1.42 (-2.67, -0.17)

Theoretical minimum-risk exposure level

For cataract, the TMREL is defined as no households using solid cooking fuel. For outcomes related to both ambient and household air pollution, the PAFs are estimated jointly and the TMREL is defined as uniform distribution between 2.4 and 5.9 ug/m^3 $PM_{2.5}$.

Relative risks

In addition to the previously included outcomes of lower respiratory infections (LRI), stroke, ischaemic heart disease (IHD), chronic obstructive pulmonary disease (COPD), lung cancer, type 2 diabetes, and cataract, in GBD 2019 we added low birthweight and short gestation as new outcomes of household air pollution through a mediation analyses. With the exception of cataract, all causes share risk curves and are jointly calculated with ambient $PM_{2.5}$ air pollution. Table 2 summarizes relative risk input data for ambient particulate matter pollution and household air pollution.

Table 2: Relative Risk Input Data

Input data	Relative risk
Source count (total)	200
Number of countries with data	40

Prior to GBD 2019, we utilised the results of an external meta-analysis with a summary relative of 2.47 with 95% CI (1.63, 3.73).[1] While this effect estimate was for both sexes, in the past we estimated burden for women only because women are known to have higher HAP exposure than men. In GBD 2019, we performed our own meta-regression analysis of household air pollution and cataracts. We extracted all of the components studies of the above meta-analysis paper but excluded one cross-sectional study. GBD risk factor analyses typically do not include cross-sectional analyses. In additional literature search, we found one additional paper describing different fuel types and cataracts.[4] We excluded this study because there was no comparison group without solid fuel use. Our resulting dataset contained eight estimates from six sources in India and Nepal.

On these eight estimates, we ran a MR BRT meta-regression to generate a summary effect size of 2.51 (1.58, 3.96). We included a study-level bias covariate of whether or not the study participants were blind to the exposure-outcome pair of interest. The prior on this covariate was a Gaussian distribution with mean 0 and variance 0.1. The prior on gamma was a Gaussian distribution with mean 0.04 and 0.1. The table and figure below provide the model coefficients and a visual representation.

MR-BRT relative risk meta-analysis for household air pollution and cataract

Covariate	Gamma	Beta coefficient, logit (95% CI)	Beta coefficient, adjusted (95% CI)
Intercept	0.40	0.918 (0.460, 1.377)	2.51 (1.58, 3.96)

Outcome unblinded		0.031 (-0.450, 0.512)	1.03 (0.64, 1.67)

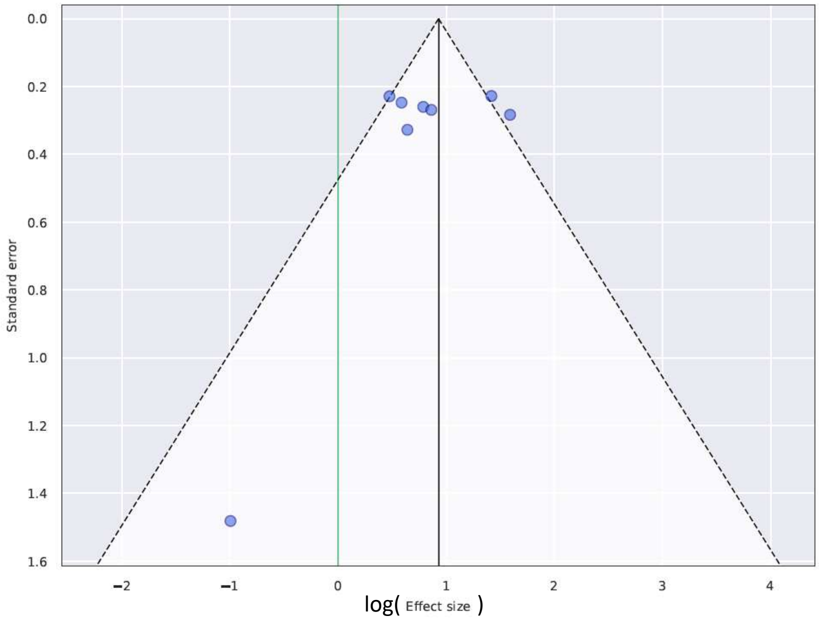

Studies reported effect sizes for males, females, and/or both sexes. In a sensitivity analysis we included a covariate for sex and found no significant difference in effect size by sex. Therefore, we now estimate cataract as an outcome of household air pollution in both males and females.

In GBD 2019, we also made substantial changes to our particulate matter risk curves. These risk curves, utilising splines in MR-BRT, the new mediation analysis with birthweight and gestational age, and the joint-estimation PAF approach are described in the ambient particulate matter appendix.

$PM_{2.5}$ mapping value

In order to use the particulate matter risk curves, we must estimate the level of exposure to particulate matter with diameter of less than 2.5 micrometers ($PM_{2.5}$) for individuals using solid fuels for cooking. The Global Household Air Pollution (HAP) Measurements database from WHO contains 196 studies with measurements from 43 countries of various pollution metrics in households using solid fuel for cooking.[2] From this database, we take all measurements of $PM_{2.5}$ using indoor or personal monitors. In addition to the WHO database, we included eight additional studies from a systematic review conducted in 2015 for GBD.

The final dataset included 336 estimates from 75 studies in 43 unique locations. We included 260, 64, nine, and three measurements indoors, on personal monitors for females, children (under 5), and males, respectively. 274 estimates were in households using solid fuels, 47 in households only using clean (gas or electricity) fuels, and 15 in households using a mixture of solid and clean fuels.

We use the following model:

$$\log(excess\ PM) \sim solid + measure\ group + 24\ hr\ measurement + SDI + (1|study)$$

Where,

- 24-hour measurement: binary variable equal to 1 if the measurement occurred over at least a 24-hour period and not only during mealtimes
- Measure group: categorical variable indicating indoor, female, male, or children
- Solid: indicator variable equal to 1 if the measurements were among households using solid fuel only, 0.5 if the measurements represented a mix of clean and solid fuels, and 0 if the households only used clean fuels.

We also included the Socio-demographic Index (SDI) as a variable to predict a unique value of HAP for each location and year based on development. We also included a random effect on study. We weighted each study by its sample size.

Before modelling, we calculated the excess particulate matter in households using solid fuel by subtracting off the predicted ambient $PM_{2.5}$ value in the study location and year based on the GBD 2017 $PM_{2.5}$ exposure model. The final model coefficients are included below:

HAP mapping model and coefficients

Variable	Beta, log (95% CI)	Beta, adjusted (95% CI)
Intercept	6.23 (4.58, 7.88)	506 (97, 2635)
Solid	2.60 (2.06, 3.13)	13.4 (7.8, 23.0)
Measure group<br>• Indoor (ref)<br>• Female<br>• Male<br>• Child	 <br>-0.56 (-1.15, 0.04)<br>-1.56 (-3.81, 0.70)<br>-1.13 (-2.06, -0.20)	 <br>0.57 (0.32, 1.04)<br>0.21 (0.02, 2.02)<br>0.32 (0.13, 0.82)
24-hour measurement	-0.29 (-1.04, 0.46)	0.75 (0.35, 1.59)
SDI	-6.42 (-9.30, -3.54)	1.6 e -3 (9.1 e -5, 2.9 e -2)

Therefore, for females in households using solid fuel, we would expect their long-term mean excess $PM_{2.5}$ exposure due to the use of solid fuels to be 1522, 117, and 9 µg/m^3 in SDI of 0.1, 0.5, and 0.9, respectively.

Because there are so few studies of personal monitoring in men and children, rather than directly using the results of the model, we generated ratios using studies that measured at least two of the population groups for any size particulate matter. For $PM_{2.5}$ we used the predicted ambient $PM_{2.5}$ value in the study location and year based on the GBD 2017 $PM_{2.5}$ exposure model as the "outdoor" measurement, and for PM_4 and PM_{10} we used published values in the studies themselves. We first subtracted off this outdoor value from each PM measurement, and then calculated the ratio of male to female and child to female exposure, weighted by sample size.

Study	Location	Year	Pollutant	Female N	Female PM	Group	N	PM	Outdoor
Balakrishnan et al., 2004	Andhra Pradesh, Rural	2004	PM_4	591	352	male	503	187	94
Gao X et al., 2009.	Tibet	2009	$PM_{2.5}$	52	127	male	85	111	27
Dasgupta et al., 2006	Bangladesh	2006	PM_{10}	944	209	male	944	166	50

Devkumar et al., 2014	Nepal	2014	$PM_{2.5}$	405	169	male	429	167	90
Balakrishnan et al., 2004	Andhra Pradesh, Rural	2004	PM_4	591	352	child	56	262	94
Dionisio et al., 2008.	The Gambia	2008	$PM_{2.5}$	13	275	child	13	219	31
Dasgupta et al., 2006	Bangladesh	2006	PM_{10}	944	209	child	944	199	50

The final ratios were 0.64 95% CI (0.45, 0.91) for males and 0.85 95% CI (0.56, 1.31) for children. We used these results to scale the $PM_{2.5}$ mapping model for these age and sex groups to input into the $PM_{2.5}$ risk curves.

References

1. Smith KR, Bruce N, Balakrishnan K, Adair-Rohani H, Balmes J, Chafe Z, *et al*. Millions Dead: How Do We Know and What Does It Mean? Methods Used in the Comparative Risk Assessment of Household Air Pollution. Annu Rev Public Health. 2014; **35**(1):185–206.
2. Shupler M, Balakrishnan K, Ghosh S, *et al*. Global household air pollution database: Kitchen concentrations and personal exposures of particulate matter and carbon monoxide. Data in Brief 2018; **21**: 1292–5.
3. Shupler M, Godwin W, Frostad J, Gustafson P, Arku RE, Brauer M. Global estimation of exposure to fine particulate matter (PM2.5) from household air pollution. Environment International 2018; **120**: 354–63.
4. Tanchangya J, Geater AF. Use of traditional cooking fuels and the risk of young adult cataract in rural Bangladesh: a hospital-based case-control study. *BMC Ophthalmology* 2011; **11**.

Smoking

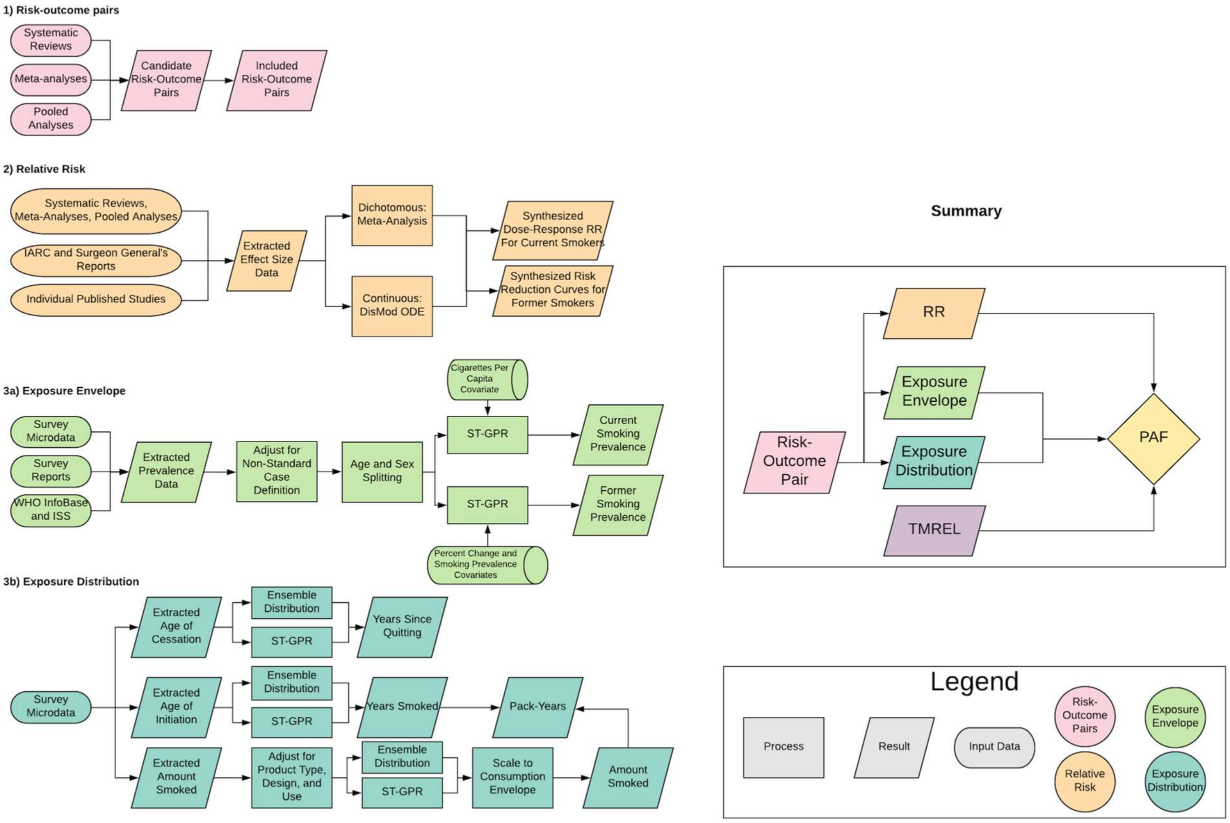

Input data and methodological summary

Definition

Exposure

As in GBD 2017, we estimated the prevalence of current smoking and the prevalence of former smoking using data from cross-sectional nationally representative household surveys. We defined current smokers as individuals who currently use any smoked tobacco product on a daily or occasional basis. We defined former smokers as individuals who quit using all smoked tobacco products for at least six months, where possible, or according to the definition used by the survey.

Input data

Our extraction method has not changed from GBD 2017. We extracted primary data from individual-level microdata and survey report tabulations. We extracted data on current, former, and/or ever smoked tobacco use reported as any combination of frequency of use (daily, occasional, and unspecified, which includes both daily and occasional smokers) and type of smoked tobacco used (all smoked tobacco, cigarettes, hookah, and other smoked tobacco products such as cigars or pipes), resulting in 36 possible combinations. Other variants of tobacco products, for example hand-rolled cigarettes, were grouped into the four type categories listed above based on product similarities.

For microdata, we extracted relevant demographic information, including age, sex, location, and year, as

well as survey metadata, including survey weights, primary sampling units, and strata. This information allowed us to tabulate individual-level data in the standard GBD five-year age-sex groups and produce accurate estimates of uncertainty. For survey report tabulations, we extracted data at the most granular age-sex group provided.

Table 1: Data inputs for exposure for smoking.

Input data	Exposure
Source count (total)	3439
Number of countries with data	201

Table 2: Data inputs for relative risks for smoking.

Input data	Relative risk
Source count (total)	673
Number of countries with data	16

Crosswalk

Our GBD smoking case definitions were current smoking of any tobacco product and former smoking of any tobacco product. All other data points were adjusted to be consistent with either of these definitions. Some sources contained information on more than one case definition and these sources were used to develop the adjustment coefficient to transform alternative case definitions to the GBD case definition. The adjustment coefficient was the beta value derived from a linear model with one predictor and no intercept. We used the same crosswalk adjustment coefficients as in GBD 2017, and thus we have not included a methods explanation in this appendix, as it has been detailed previously.

Age and sex splitting

As in GBD 2017, we split data reported in broader age groups than the GBD 5-year age groups or as both sexes combined by adapting the method reported in Ng et al[1] to split using a sex- geography- time-specific reference age pattern. We separated the data into two sets: a training dataset, with data already falling into GBD sex-specific 5-year age groups, and a split dataset, which reported data in aggregated age or sex groups. We then used spatiotemporal Gaussian process regression (ST-GPR) to estimate sex-geography-time-specific age patterns using data in the training dataset. The estimated age patterns were used to split each source in the split dataset.

The ST-GPR model used to estimate the age patterns for age-sex splitting used an age weight parameter value that minimises the effect of any age smoothing. This parameter choice allowed the estimated age pattern to be driven by data, rather than being enforced by any smoothing parameters of the model. Because these age-sex split data points were to be incorporated in the final ST-GPR exposure model, we did not want to doubly enforce a modelled age pattern for a given sex-location-year on a given aggregate data point.

Modelling strategy

Smoking prevalence modelling

We used ST-GPR to model current and former smoking prevalence. The model is nearly identical to that in GBD 2017. Full details on the ST-GPR method are reported elsewhere in the appendix. Briefly, the mean function input to GPR is a complete time series of estimates generated from a mixed effects hierarchical linear model plus weighted residuals smoothed across time, space, and age. The linear model formula for current smoking, fit separately by sex using restricted maximum likelihood in R, is:

$$logit(p_{g,a,t}) = \beta_0 + \beta_1 CPC_{g,t} + \sum_{k=2}^{19} \beta_k I_{A[a]} + \alpha_s + \alpha_r + \alpha_g + \epsilon_{g,a,t}$$

Where $CPC_{g,t}$ is the tobacco consumption covariate by geography g and time t, described above, $I_{A[a]}$ is a dummy variable indicating specific age group A that the prevalence point $p_{g,a,t}$ captures, and α_s, α_r, and α_g are super-region, region, and geography random intercepts, respectively. Random effects were used in model fitting but not in prediction.

The linear model formula for former smoking is:

$$logit(p_{g,a,t}) = \beta_0 + \beta_1 PctChange_{A[a],g,t} + \beta_3 CSP_{A[a],g,t} + \sum_{k=3}^{20} \beta_k I_{A[a]} + \alpha_s + \alpha_r + \alpha_g + \epsilon_{g,a,t}$$

Where $PctChange_{A[a],g,t}$ is the percentage change in current smoking prevalence from the previous year, and $CSP_{A[a],g,t}$ is the current smoking prevalence by specific age group A, geography g, and time t that point $p_{g,a,t}$ captures, both derived from the current smoking ST-GPR model defined above.

Supply-side estimation

The methods for modelling supply-side-level data were changed substantially from those used in GBD 2017. The raw data were domestic supply (USDA Global Surveillance Database and UN FAO) and retail supply (Euromonitor) of tobacco. Domestic supply was calculated as production + imports - exports. The data went through three rounds of outliering. First, they were age-sex split using daily smoking prevalence to generate number of cigarettes per smoker per day for a given location-age-sex-year. If more than 12 points for a particular source-location-year (equal to over 1/3 of the split points) were above the given thresholds, that source-location-year was outliered. A point would not be outliered if it was (in cigarettes per smoker): under five (10–14 year olds); under 20 (males, 15–19 year olds); under 18 (females, 15–19 year olds); under 38/35 and over three (males/females, 20+ year olds). These thresholds were chosen by visualising histograms of the data for each age-sex, as well as with expert knowledge about reasonable consumption levels. In the second round of outliering, the mean tobacco per capita value over a 10-year window was calculated. If a point was over 70% of that mean value away from the mean value, it was outliered. The 70% limit was chosen using histograms of these distances. Additionally, some manual outliering was performed to account for edge cases. Finally, data smoothing was performed by taking a three-year rolling mean over each location-year.

Next, a simple imputation to fill in missing years was performed for all series to remove compositional bias from our final estimates. Since the data from our main sources covered different time periods, by imputing a complete time series for each data series, we reduced the probability that compositional bias of the sources was leading to biased final estimates. To impute the missing years for each series, we modelled the log ratio of each pair of sources as a function of an intercept and nested random effects on super-region, region, and location. The appropriate predicted ratio was multiplied by each source that we did have, and then the predictions were averaged to get the final imputed value. For example, if source A was missing for a particular location-year, but sources B and C were present, then we predicted A twice: once from the modelled ratio of A to B, and again from the modelled ratio of A to C. These two predictions were then averaged. For some locations where there was limited overlap between series, the predicted ratio did not make sense, and a regional ratio was used.

Finally, variance was calculated both across series (within a location-year) as well as across years (within a location-source). Additionally, if a location-year had one imputed point was, the variance was multiplied by 2. If a location-year had two imputed points, the variance was multiplied by 4. The average estimates in each location-year were the input to an ST-GPR model. For this, we used a simple mixed effects model, which was modelled in log space with nested location random effects. Subnational estimates were then further modelled by splitting the country-level estimates using current smoking prevalence.

Theoretical minimum-risk exposure level

The theoretical minimum-risk exposure level is 0.

Exposure among current and former smokers

Identical to GBD 2017, we estimated exposure among current smokers for two continuous indicators: cigarettes per smoker per day and pack-years. Pack-years incorporates aspects of both duration and amount. One pack-year represents the equivalent of smoking one pack of cigarettes (assuming a 20-cigarette pack) per day for one year. Since the pack-years indicator collapses duration and intensity into a single dimension, one pack-year of exposure can reflect smoking 40 cigarettes per day for six months or smoking 10 cigarettes per day for two years.

To produce these indicators, we simulated individual smoking histories based on distributions of age of initiation and amount smoked. We informed the simulation with cross-sectional survey data capturing these indicators, modelled at the mean level for all locations, years, ages, and sexes using ST-GPR. We rescaled estimates of cigarettes per smoker per day to an envelope of cigarette consumption based on supply-side data. We estimated pack-years of exposure by summing samples from age- and time-specific distributions of cigarettes per smoker for a birth cohort in order to capture both age trends and time trends and avoid the common assumption that the amount someone currently smokes is the amount they have smoked since they began smoking. All distributions were age-, sex-, and region- specific ensemble distributions, which were found to outperform any single distribution.

We estimated exposure among former smokers using years since cessation. We utilised ST-GPR to model mean age of cessation using cross-sectional survey data capturing age of cessation. Using these estimates, we generated ensemble distributions of years since cessation for every location, year, age group, and sex.

Relative risk

The same risk-outcome pairs from GBD 2017 were used: tuberculosis, lower respiratory tract infections, oesophageal cancer, stomach cancer, bladder cancer, liver cancer, laryngeal cancer, lung cancer, breast cancer, cervical cancer, colorectal cancer, lip and oral cancer, nasopharyngeal cancer, other pharyngeal cancer, pancreatic cancer, kidney cancer, leukaemia, ischaemic heart disease, ischaemic stroke, haemorrhagic stroke, subarachnoid haemorrhage, atrial fibrillation and flutter, aortic aneurysm, peripheral arterial disease, chronic obstructive pulmonary disease, other chronic respiratory diseases, asthma, peptic ulcer disease, gallbladder and biliary tract diseases, Alzheimer disease and other dementias, Parkinson disease (protective), multiple sclerosis, type-II diabetes, rheumatoid arthritis, low back pain, cataracts, macular degeneration, and fracture.

Dose-response risk curves

Input data for relative risks were nearly the same as in GBD 2017. The only addition was for chronic obstructive pulmonary disease, for which a few additional studies were included. We synthesised effect sizes by cigarettes per smoker per day, pack-years, and years since quitting from cohort and case-control studies to produce nonlinear dose-response curves using a Bayesian meta-regression model. For outcomes with significant differences in effect size by sex or age, we produced sex- or age-specific risk curves.

We estimated risk curves of former smokers compared to never smokers taking into account the rate of risk reduction among former smokers seen in the cohort and case-control studies, and the cumulative exposure among former smokers within each age, sex, location, and year group.

Population attributable fraction (PAF)

As in GBD 2017, we estimated PAFs based on the following equation:

$$PAF = \frac{p(n) + p(f)\int \exp(x) * rr(x) + p(c)\int \exp(y) * rr(y) - 1}{p(n) + p(f)\int \exp(x) * rr(x) + p(c)\int \exp(y) * rr(y)}$$

where $p(n)$ is the prevalence of never smokers, $p(f)$ is the prevalence of former smokers, $p(c)$ is the prevalence of current smokers, $\exp(x)$ is a distribution of years since quitting among former smokers, $rr(x)$ is the relative risk for years since quitting, $\exp(y)$ is a distribution of cigarettes per smoker per day or pack-years, and $rr(y)$ is the relative risk for cigarettes per smoker per day or pack-years.

We used pack-years as the exposure definition for cancers and chronic respiratory diseases, and cigarettes per smoker per day for cardiovascular diseases and all other health outcomes.

References

1. Ng M, Freeman MK, Fleming TD, Robinson M, Dwyer-Lindgren L, Thomson B, et al. Smoking Prevalence and Cigarette Consumption in 187 Countries, 1980–2012. *JAMA*. 2014 Jan 8;311(2):183–92.

Secondhand smoke

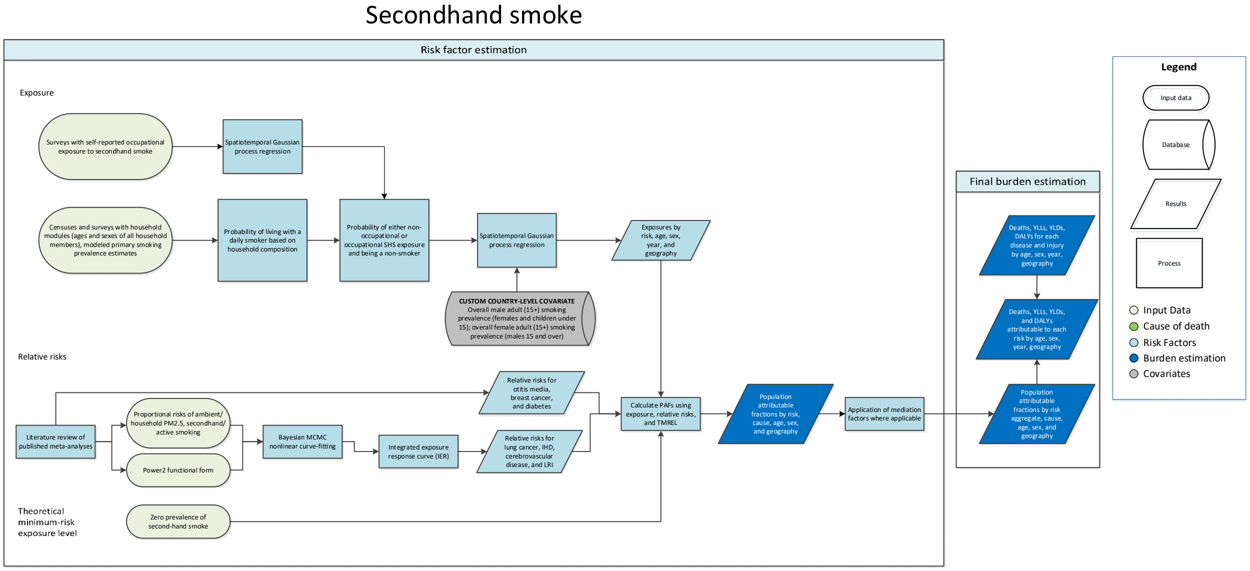

Exposure

Case definition

We define secondhand smoke exposure as current exposure to secondhand tobacco smoke at home, at work, or in other public places. We use household composition as a proxy for non-occupational secondhand smoke exposure and make the assumption that all persons living with a daily smoker are exposed to tobacco smoke. We use surveys to estimate the proportion of individuals exposed to secondhand smoke at work. We only consider non-smokers to be exposed to secondhand smoke. Non-smokers are defined as all persons who are not daily smokers. Ex-smokers and occasional smokers are considered non-smokers in this analysis. Exposure is evaluated for both children and adults.

Input data

To calculate the proportion of non-smokers who live with at least one smoker, we used unit record data on household composition, which included the ages and sexes of all persons living in the same household. Our sources included representative major survey series with a household composition module, including the Demographic Health Surveys (DHS), the Multiple Indicator Cluster Surveys (MICS), and the Living Standards Measurement Surveys (LSMS); and national and subnational censuses, which included those captured in the IPUMS project and identified using the Global Health Data Exchange catalog (GHDx).

To calculate the proportion of individuals exposed to secondhand smoke at work, by age and sex, we used cross-sectional surveys that ask respondents about self-reported occupational secondhand smoke exposure. Sources include the Global Adult Tobacco Surveys, Eurobarometer Surveys, and WHO STEPS Surveys. We identified sources using the GHDx.

No major changes have been introduced to data inputs since 2016. A new systematic review is planned for the next GBD round. Table 1 summarizes exposure input data.

Input data	Exposure
Source count (total)	721
Number of countries with data	153

Given the nature of the data used in our models (microdata), no crosswalk for case definition adjustment or age- and sex-splitting processes were required. Estimates of daily smoking prevalence in each location were also used in our calculations, as described in the modelling strategy section below.

Modelling strategy

Identical to GBD 2017, we estimated the probability that each person is living with a smoker and is also a non-smoker themselves using set theory. First, household composition data were used at the individual level to capture the ages and sexes of each person in the household. Second, we analysed surveys with both household composition data and tobacco use questions and determined that the distribution of household size, mean age of the household members, and the age distribution were not significantly different between households with and without a self-reported smoker. Since we did not find that household composition varied between smokers and non-smokers, we then used the GBD 2019 primary daily smoking prevalence model to calculate the probability that each household member is a daily smoker. Next, we used the probability of the union of sets on each individual household member to calculate the overall probability that at least one of the other household members was a daily smoker. As in GBD 2017, we incorporated occupational exposure by modelling prevalence of current exposure to secondhand smoke at work, by age, sex, location, and year, using ST-GPR. In order to avoid double counting we calculated the probability that an individual is exposed through either non-occupational exposure or occupational exposure, given their age, sex, and household composition. Finally, we multiplied this probability of exposure by the probability that the individual is not a smoker themselves (ie, 1 minus primary daily smoking prevalence for that person's location, year, age, and sex). We then collapsed these individual-level probabilities to produce average probabilities of exposure by location, year, age, and sex.

These probabilities were modelled in the GBD ST-GPR framework, which generates exposure estimates from a mixed effects hierarchical linear model plus weighted residuals smoothed across time, space, and age. The linear model formula was fit separately by sex using restricted maximum likelihood in R.

We used the sex-specific overall daily smoking prevalence for adults (age 15 and older) as a country-level covariate in the model. The overall male adult daily smoking prevalence was used as the covariate for females of all ages and for males under age 15. The overall female adult daily smoking prevalence was used as the covariate for males age 15 and older.

All input datapoints from the probability calculation had a measure of uncertainty (variance and sample size) coming from the uncertainty of the primary smoking prevalence model and the sample size from the unit record data going into the modelling process. Geographical random effects were used in model fitting but were not used in prediction.

Theoretical minimum-risk exposure level

The theoretical minimum-risk exposure level for secondhand smoke is zero exposure among non-smokers, meaning that non-smokers would not live with any primary smokers.

Relative risks

The same risk-outcome pairs from GBD 2017 were used. For children ages 0-14, we estimated the burden of otitis media attributable to secondhand smoke exposure. For all ages we estimated the burden of lower respiratory infections (LRI), and for adults greater than or equal to 25 years of age we estimated the burden of lung cancer, chronic obstructive pulmonary disease (COPD), ischaemic heart disease, and cerebrovascular disease attributable to secondhand smoke exposure, breast cancer, and type 2 diabetes.

For lung cancer, ischaemic heart disease, cerebrovascular disease, and LRI, we used country-specific relative risks created using integrated exposure response curves (IER) for $PM_{2.5}$ air pollution. IER curve calculation was updated with the GBD 2019 cigarettes per smoker estimates. The relative risks for otitis media[1], breast cancer[2], and diabetes[3] are derived from published meta-analyses and are the same as the ones used in the previous GBD cycle. Table 2 summarizes relative risk input data.

Input data	Exposure
Source count (total)	232
Number of countries with data	34

We used the standard GBD population attributable fraction (PAF) equation to estimate burden based on exposure and relative risks.

References

1. Jones LL, Hassanien A, Cook DG, Britton J, Leonardi-Bee J. Parental smoking and the risk of middle ear disease in children. Arch Pediatr Adolesc Med. 2012; 166: 18–27.
2. Macacu A, Autier P, Boniol M, Boyle P. Active and passive smoking and risk of breast cancer: a meta-analysis. Breast Cancer Res Treat 2015; 154:213–224.
3. Zhu B, Wu X, Wang X, Zheng Q, Sun G. The association between passive smoking and type 2 diabetes: a meta-analysis. Asia-Pacific Journal of Public Health 2014; 26:226-237.

Chewing tobacco

Chewing Tobacco

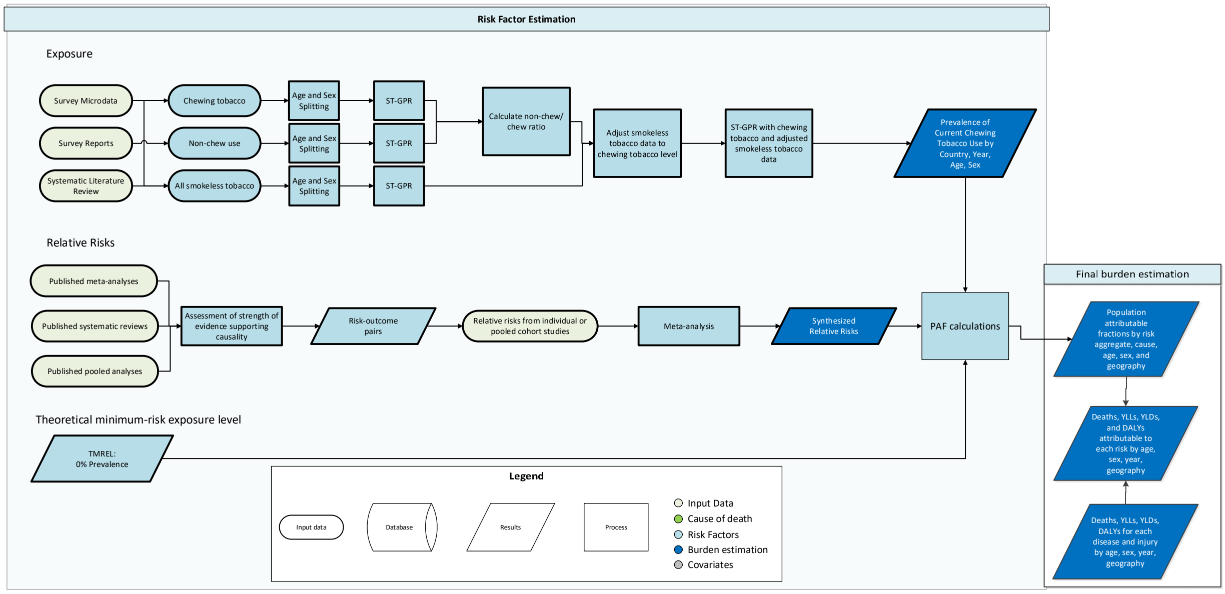

Input data and methodological summary

Definition

Exposure

Current chewing tobacco use is defined as current use (use within the last 30 days where possible, or according to the closest definition available from the survey) of any frequency (any, daily, or less than daily). Chewing tobacco includes local products, such as betel quid with tobacco.

Input data

As in GBD 2017, we included sources that reported primary chewing tobacco, non-chew smokeless tobacco, and all smokeless tobacco use among respondents over age 10. To be eligible for inclusion, sources had to be representative for their level of estimation (ie, national sources needed to be nationally representative, subnational sources subnationally representative). We included only self-reported use data and excluded data from questions asking about others' tobacco use behaviours.

We extracted primary data from individual-level microdata and survey report tabulations on chewing tobacco, non-chew smokeless tobacco, and all smokeless tobacco use. We extracted data on current, former, and/or ever use as well as frequency of use (daily, occasional, and unspecified, which includes both daily and occasional smokers). Products that do not include tobacco, such as betel quid without tobacco, were excluded or estimated separately as part of the drug use risk factor, if applicable.

For microdata, we extracted relevant demographic information, including age, sex, location, and year, as well as survey metadata, including survey weights, primary sampling units, and strata. This information allowed us to tabulate individual-level data in the standard GBD five-year age-sex groups and produce accurate estimates of uncertainty. For survey report tabulations, we extracted data at the most granular age-sex group provided.

Table 1: Data inputs for exposure for chewing tobacco.

Input data	Exposure
Source count (total)	5030
Number of countries with data	203

Table 2: Data inputs for relative risks for chewing tobacco.

Input data	Relative risk
Source count (total)	827
Number of countries with data	38

Age and sex splitting

We split data reported in broader age groups than the GBD five-year age groups or as both sexes combined by adapting the method reported in Ng and colleagues (http://jamanetwork.com/journals/jama/fullarticle/1812960) to split using a sex-geography-time-specific reference age pattern. We separated the data into two sets: a training dataset, with data already falling into GBD sex-specific five-year age groups, and a split dataset, which reported data in aggregated age or sex groups. We then used spatiotemporal Gaussian process regression (ST-GPR) to estimate sex-geography-time-specific age patterns using data in the training dataset. The estimated age patterns were then used to split each source in the split dataset.

The ST-GPR model used to estimate the age patterns for age-sex splitting used an age weight parameter value that minimises the effect of any age smoothing. This parameter choice allows the estimated age pattern to be driven by data, rather than being enforced by any smoothing parameters of the model. Because these age-sex-split datapoints will be incorporated in the final ST-GPR exposure model, we do not want to doubly enforce a modelled age pattern for a given sex-location-year on a given aggregate datapoint. We run three separate ST-GPR models for age-sex splitting – one for each smokeless tobacco category (chew, non-chew, and all smokeless).

Modelling strategy

Prevalence modelling

We used a ST-GPR to model chewing tobacco prevalence. Full details on the ST-GPR method are reported elsewhere in the Appendix. Briefly, the mean function input to GPR is a complete time series of estimates generated from a mixed effects hierarchical linear model plus weighted residuals smoothed across time, space, and age. The linear model formula for chewing tobacco, fit separately by sex using restricted maximum likelihood in R, is:

$$logit(p_{g,a,t}) = \beta_0 + \sum_{k=1}^{18} \beta_k I_{A[a]} + \alpha_s + \alpha_r + \alpha_g + \epsilon_{g,a,t}$$

Where $I_{A[a]}$ is a dummy variable indicating specific age group A that the prevalence point $p_{g,a,t}$ captures, and α_s, α_r, and α_g are super-region, region, and geography random intercepts, respectively. The hyperparameters are the same as in GBD 2017.

We run three ST-GPR models for each prevalence category – one for each smokeless tobacco category (chew, non-chew, and all smokeless).

All smokeless tobacco prevalence adjustment
Using the 1000 draws from each of the prevalence ST-GPR models, we calculated 1000 draws of chewing tobacco prevalence divided by the sum of chewing tobacco and non-chewing tobacco prevalence for each location, age group, sex, and year. The draws were unordered, as we did not want to enforce an assumption about the relationship between the levels of chewing tobacco and non-chewing tobacco prevalence.

The draws of the ratio of chewing to non-chewing tobacco were then multiplied by the draws from the all smokeless tobacco prevalence model to adjust the estimates to chewing tobacco prevalence. These were then averaged to get the mean estimate. The variance across the ratios was calculated for each location, year, age, and sex, and was added to the variance from the original all smokeless tobacco draws.

Final chewing tobacco prevalence model
To calculate the final chewing tobacco prevalence, we ran an additional ST-GPR model with both the original chewing tobacco data (post-age-sex splitting), as well as the adjusted data. These adjusted data add more information to the model – as surveys will often only ask about all smokeless tobacco consumption – while taking into consideration the uncertainty from the ratio calculation.

Theoretical minimum-risk exposure level
The theoretical minimum risk exposure level is that everyone in the population has been a lifelong non-user of chewing tobacco.

Relative risk
As in GBD 2017, we included outcomes based on the strength of available evidence supporting a causal relationship. There was sufficient evidence to include oral cancer and oesophageal cancer as health outcomes caused by chewing tobacco use.

Relative risk estimates were derived from prospective cohort studies and population-based case-control studies. We used the same underlying effect size estimates from prospective cohort studies and population-based case-control studies as in GBD 2017. Briefly, we did not include hospital-based case control studies due to concerns over representativeness. We only included sources that adequately adjusted for major confounders, especially smoking status. Summary effect size estimates were calculated in R, using the 'metafor' package. We performed a random effects meta-analysis using the DerSimonian and Laird method, which does not assume a true effect size but considers each input study as selected from a random sample of all possible sets of studies for the outcome of interest. The random-effects method allows for more variation between the studies, and incorporates this variance into the estimation process. We used an inverse-variance weighting method to determine component

study weights. We found significantly different relative risks for oral cancer for males and females, and estimated relative risks separately by sex for oral cancer alone.

Dietary risks

Dietary risks

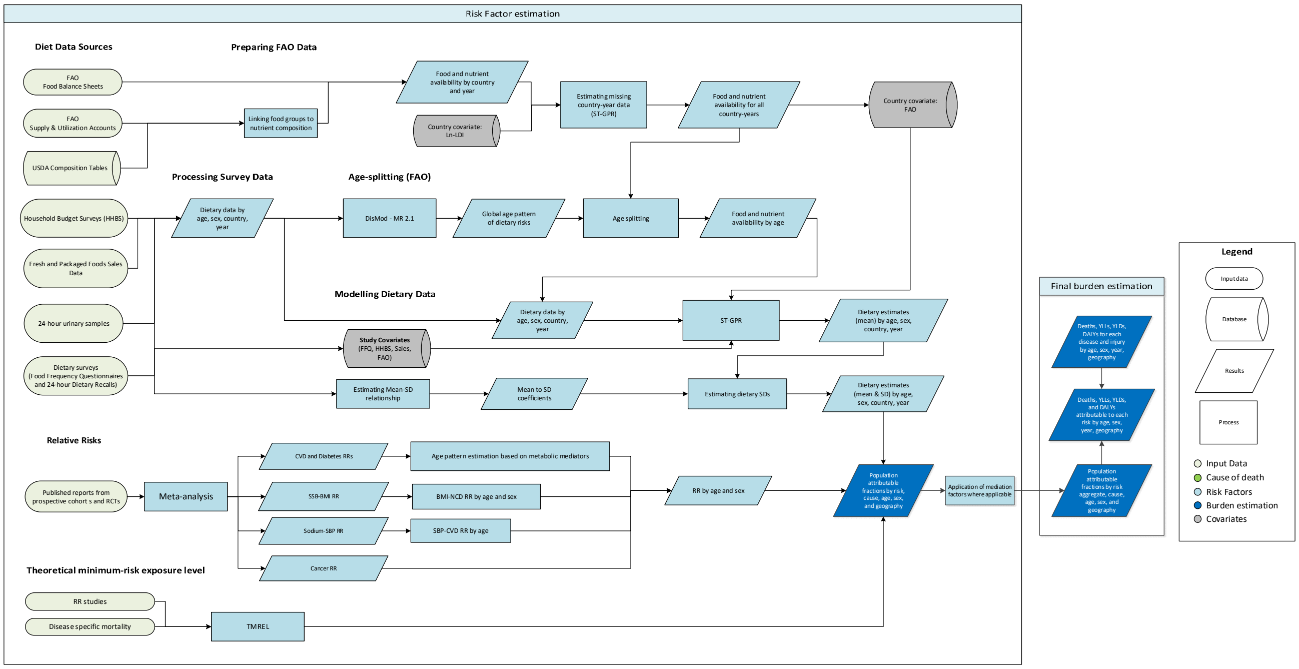

Input data and methodological summary

Definition

Exposure

Risk	Definition
**Diet low in fruit**	Average daily consumption (in grams per day) of less than 310-340 grams of fruit including fresh, frozen, cooked, canned, or dried fruit, excluding fruit juices and salted or pickled fruits
**Diet low in vegetables**	Average daily consumption (in grams per day) of less than 280-320 grams of vegetables, including fresh, frozen, cooked, canned, or dried vegetables and excluding legumes and salted or pickled vegetables, juices, nuts and seeds, and starchy vegetables such as potatoes or corn
**Diet low in whole grains**	Average daily consumption (in grams per day) of less than 140-160 grams of whole grains (bran, germ, and endosperm in their natural proportion) from breakfast cereals, bread, rice, pasta, biscuits, muffins, tortillas, pancakes, and other sources
**Diet low in nuts and seeds**	Average daily consumption (in grams per day) of less than 10-19 grams of nuts and seeds, including tree nuts and seeds and peanuts

**Diet low in fibre**	Average daily consumption (in grams per day) of less than 21-22 grams of fibre from all sources including fruits, vegetables, grains, legumes, and pulses
**Diet low in omega-3 fatty acids**	Average daily consumption (in milligrams per day) of less than 430-470 milligrams of eicosapentaenoic acid (EPA) and docosahexaenoic acid (DHA)
**Diet low in polyunsaturated fatty acids (PUFA)**	Average daily consumption (in % daily energy) of less than 7-9% total energy intake from polyunsaturated fatty acids
**Diet low in calcium**	Average daily consumption (in grams per day) of less than 1.06-1.1 grams of calcium from all sources, including milk, yogurt, and cheese
**Diet low in milk**	Average daily consumption (in grams per day) of less than 360-500 grams of milk including non-fat, low-fat, and full-fat milk, excluding soy milk and other plant derivatives
**Diet low in legumes**	Average daily consumption (in grams per day) of less than of 90-100 grams of legumes and pulses, including fresh, frozen, cooked, canned, or dried legumes
**Diet high in red meat**	Any intake (in grams per day) of red meat including beef, pork, lamb, and goat but excluding poultry, fish, eggs, and all processed meats
**Diet high in processed meat**	Any intake (in grams per day) of meat preserved by smoking, curing, salting, or addition of chemical preservatives
**Diet high in sugar-sweetened beverages (SSBs)**	Any intake (in grams per day) of beverages with ≥50 kcal per 226.8 gram serving, including carbonated beverages, sodas, energy drinks, fruit drinks, but excluding 100% fruit and vegetable juices
**Diet high in trans fatty acids**	Any intake (in percent daily energy) of trans fat from all sources, mainly partially hydrogenated vegetable oils and ruminant products
**Diet high in sodium**	Average 24-hour urinary sodium excretion (in grams per day) greater than 1-5 grams

Input data

In GBD 2019, we included new dietary recall sources from a literature search of PubMed and new sources from the IHME GHDx yearly known survey series updates in our models. We also conducted a new systematic review for sodium (Figure 1). As in GBD 2017, the dietary data that we use in the models comes from multiple sources, including nationally and subnationally representative nutrition surveys, household budget surveys, accounts of national sales from the Euromonitor, and availability data from the United Nations FAO Supply and Utilization Accounts (SUA). Table 1 below provides a summary of data inputs used for dietary risk modeling in GBD 2019.

Figure 1: PRISMA diagram for sodium intake data systematic review

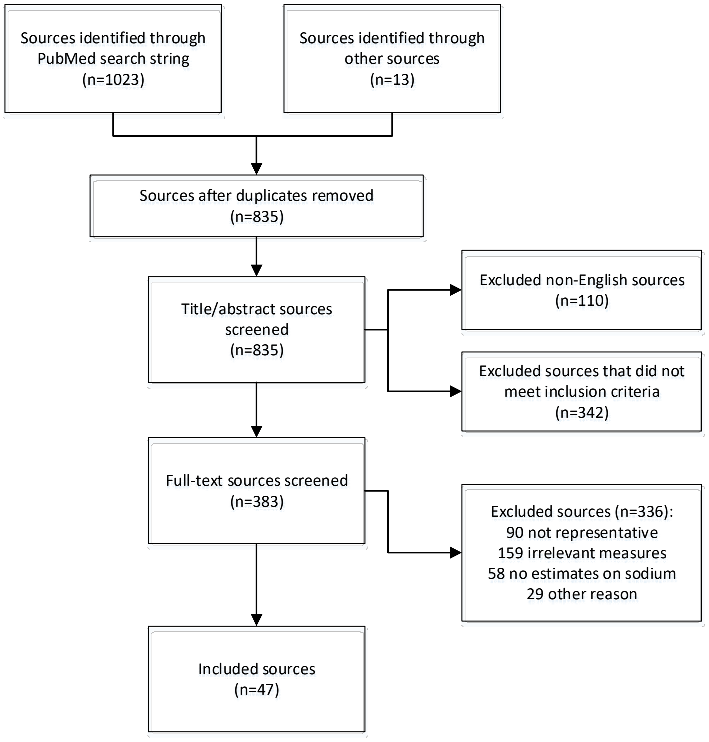

Table 1a: Data inputs for exposure for dietary risk factors.

Dietary risk factor	Total exposure sources	Countries with data
All dietary risks	1461	195
Calcium	160	178
Fiber	155	180
Fruit	869	180
Legumes	683	169
Milk	1148	177
Nuts and seeds	100	158
Omega 3	20	178
Processed meat	737	66
PUFA	70	180
Red meat	760	178
Sodium	92	53
SSBs	720	66
Trans fat	924	72
Vegetables	871	180
Whole grains	52	188

Table 1b: Data inputs for risk analysis for dietary risk factors.

Dietary risk factor	Total relative risk sources	Countries with data
Calcium	37	9
Fiber	64	16
Fruit	116	23
Legumes	10	5
Milk	12	8
Nuts and seeds	23	9
Omega 3	50	16
Processed meat	41	11
PUFA	18	8
Red meat	92	20
Sodium	21	6
SSBs	15	5
Transfat	10	5
Vegetable	39	11
Whole grains	37	9

The availability data for food groups in GBD were previously based on the FAO Food Balance Sheets (FBS), which provide tabulated and processed data of national food supply. In GBD 2019, to more accurately characterise the national availability of various food groups, we used more disaggregated data on food commodities that were included in FAO SUA and recreated the national availability of each food group based on the GBD definition of the food group. We modelled missing country-year data from FAO using a spatiotemporal Gaussian process regression and lag-distributed country income as the covariate. For nutrient availability, we continued to use data from Global Nutrient Database.[1]

For each dietary factor, we estimated the global age pattern of consumption based on nutrition surveys (ie, 24-hour diet recall) and applied that age pattern to the all-age data (availability, sales and household budget surveys) before the data source bias adjustment.

Our gold-standard data source for all dietary risks (except sodium) is 24-hour dietary recall surveys where food and nutrient intake are reported or convertible to grams per person per day; the gold-standard data source for sodium is 24-hour urinary sodium. The other data sources we use – household budget surveys, food frequency questionnaires, sales, and availability – are treated as alternate definitions for dietary intake and crosswalked to the gold-standard definition. In GBD 2016 and GBD 2017, we determined the bias adjustment factors from a mixed effects linear regression. In GBD 2019, we used MR-BRT (a network meta-regression) to determine the adjustment factors for non-gold-standard datapoints. Coefficients for these models can be found in Table 3.

Table 2. Types of data sources (other than 24-hour dietary recall) and covariates used in modelling of each dietary factor.

	Data sources				Country-level covariate
	Sales	FFQ[1]	HBS[2]	FAO	
Diet low in fruits	●	●	●	●	Lag distributed income
Diet low in vegetables	●	●	●	●	Energy availability (kcal)
Diet low in whole grains	-	●	-	●	Energy availability (kcal)
Diet low in nuts and seeds	-	-	●	●	Energy availability (kcal)
Diet low in milk	●	●	●	●	Energy availability (kcal)
Diet high in red meat	●	●	●	●	Energy availability (kcal)
Diet high in processed meat	●	●	●	-	Energy availability (kcal), pigs per capita
Diet low in legumes	●	●	-	●	Energy availability (kcal)
Diet high in sugar-sweetened beverages	●	●	●	-	Energy availability (kcal), availability of sugar
Diet low in fibre	-	●	-	●	Energy availability (kcal)
Diet suboptimal in calcium	-	●	-	●	Energy availability (kcal)
Diet low in seafood omega-3 fatty acids	-	-	-	●	Lag distributed income, proportion landlocked area
Diet low in polyunsaturated fatty acids	-	●	-	●	Lag distributed income
Diet high in trans fatty acids	●	●	-	-	
Diet high in sodium[3]	-	-	-	-	

[1] Food Frequency Questionnaire
[2] Household Budge Survey
[3] For sodium, we used data from the 24-hour urinary sodium and 24-hour dietary recall.

Table 3: MR-BRT crosswalk adjustment factors for all dietary risks

Dietary risk	Sex	Data input	Reference or alternative case definition	Gamma	Beta coefficient, log (95% CI)	Adjustment factor*
Calcium	---	DR	Ref	0.24	---	---
Calcium	Female	FAO	Alt		0.04 (0.04, 0.5)	0.96 (0.64, 1.65)
Calcium	Female	FFQ	Alt		-0.04 (-0.04, 0.43)	1.04 (0.59, 1.53)
Calcium	Male	FAO	Alt		0.17 (0.17, 0.63)	0.84 (0.73, 1.88)
Calcium	Male	FFQ	Alt		0.09 (0.09, 0.55)	0.91 (0.67, 1.74)
Fibre	---	DR	Ref	0.33	---	---
Fibre	Female	FAO	Alt		0.56 (0.56, 1.17)	0.57 (0.93, 3.23)
Fibre	Female	FFQ	Alt		0.27 (0.27, 0.88)	0.76 (0.69, 2.41)
Fibre	Male	FAO	Alt		0.55 (0.55, 1.17)	0.57 (0.92, 3.22)
Fibre	Male	FFQ	Alt		0.26 (0.26, 0.88)	0.77 (0.69, 2.4)
Fruit	---	DR	Ref	0.76	---	---
Fruit	Female	FAO	Alt		0.36 (0.36, 1.83)	0.7 (0.31, 6.21)
Fruit	Female	Sales	Alt		0.73 (0.73, 2.19)	0.48 (0.45, 8.98)

Fruit	Female	FFQ	Alt		-0.15 (-0.15, 1.32)	1.17 (0.19, 3.73)
Fruit	Female	HHBS	Alt		0.23 (0.23, 1.71)	0.79 (0.27, 5.5)
Fruit	Male	FAO	Alt		0.32 (0.32, 1.79)	0.73 (0.3, 5.97)
Fruit	Male	Sales	Alt		0.69 (0.69, 2.16)	0.5 (0.43, 8.64)
Fruit	Male	FFQ	Alt		-0.19 (-0.19, 1.28)	1.21 (0.18, 3.58)
Fruit	Male	HHBS	Alt		0.19 (0.19, 1.66)	0.83 (0.26, 5.27)
Legumes	---	DR	Ref		---	---
Legumes	Female	FAO	Alt		-0.08 (-1.49,1.39)	1.08 (0.22,4)
Legumes	Female	Sales	Alt		-0.9 (-2.31,0.56)	2.47 (0.1,1.75)
Legumes	Female	FFQ	Alt	0.74	-0.53 (-1.94,0.95)	1.7 (0.14,2.58)
Legumes	Male	FAO	Alt		0.06 (-1.35,1.53)	0.94 (0.26,4.61)
Legumes	Male	Sales	Alt		-0.76 (-2.16,0.7)	2.14 (0.12,2.01)
Legumes	Male	FFQ	Alt		-0.39 (-1.79,1.09)	1.47 (0.17,2.98)
Milk	---	DR	Ref		---	---
Milk	Female	FAO	Alt		0.27 (0.27, 2.57)	0.76 (0.16, 13.01)
Milk	Female	Sales	Alt		0.01 (0.01, 2.31)	0.99 (0.13, 10.11)
Milk	Female	FFQ	Alt		0.46 (0.46, 2.78)	0.63 (0.18, 16.2)
Milk	Female	HHBS	Alt	1.06	-0.61 (-0.61, 1.69)	1.84 (0.07, 5.4)
Milk	Male	FAO	Alt		0.28 (0.28, 2.58)	0.75 (0.17, 13.17)
Milk	Male	Sales	Alt		0.03 (0.03, 2.33)	0.97 (0.13, 10.23)
Milk	Male	FFQ	Alt		0.48 (0.48, 2.8)	0.62 (0.18, 16.43)
Milk	Male	HHBS	Alt		-0.59 (-0.59, 1.7)	1.81 (0.07, 5.48)
Nuts	---	DR	Ref		---	---
Nuts	Female	FAO	Alt		0.49 (0.49, 3.63)	0.62 (0.06, 37.68)
Nuts	Female	FFQ	Alt		-0.34 (-0.34, 2.76)	1.41 (0.02, 15.75)
Nuts	Female	HHBS	Alt	1.58	-0.72 (-0.72, 2.42)	2.06 (0.02, 11.27)
Nuts	Male	FAO	Alt		0.6 (0.6, 3.73)	0.55 (0.07, 41.65)
Nuts	Male	FFQ	Alt		-0.23 (-0.23, 2.87)	1.26 (0.03, 17.58)
Nuts	Male	HHBS	Alt		-0.62 (-0.62, 2.54)	1.85 (0.02, 12.66)
Omega-3	---	DR	Ref		---	---
Omega-3	Male	FAO	Alt	0.12	-1.15 (-1.15, -0.92)	3.16 (0.25, 0.4)
Omega-3	Female	FAO	Alt		-1.01 (-1.01, -0.78)	2.75 (0.29, 0.46)
Proc. meat	---	DR	Ref		---	---
Proc. meat	Female	Sales	Alt		0.79 (0.79, 3.14)	0.46 (0.19, 23.07)
Proc. meat	Female	FFQ	Alt		-0.3 (-0.3, 2.25)	1.35 (0.05, 9.49)
Proc. meat	Female	HHBS	Alt	1.21	-0.46 (-0.46, 1.89)	1.59 (0.05, 6.63)
Proc. meat	Male	Sales	Alt		0.95 (0.95, 3.3)	0.39 (0.22, 27.03)
Proc. meat	Male	FFQ	Alt		-0.13 (-0.13, 2.42)	1.14 (0.06, 11.2)
Proc. meat	Male	HHBS	Alt		-0.3 (-0.3, 2.06)	1.35 (0.06, 7.82)
PUFA	---	DR	Ref		---	---
PUFA	Female	FAO	Alt		-0.14 (-0.14, 0.14)	1.15 (0.65, 1.15)
PUFA	Female	FFQ	Alt	0.14	1.05 (1.05, 1.43)	0.35 (1.96, 4.18)
PUFA	Male	FAO	Alt		-0.18 (-0.18, 0.1)	1.2 (0.62, 1.1)

PUFA	Male	FFQ	Alt		1 (1, 1.38)	0.37 (1.87, 3.98)
Red meat	---	DR	Ref	0.83	---	---
Red meat	Female	FAO	Alt		0.89 (0.89, 2.54)	0.41 (0.45, 12.69)
Red meat	Female	Sales	Alt		1.09 (1.09, 2.74)	0.34 (0.54, 15.49)
Red meat	Female	FFQ	Alt		-0.34 (-0.34, 1.6)	1.4 (0.11, 4.95)
Red meat	Female	HHBS	Alt		0.45 (0.45, 2.1)	0.64 (0.29, 8.18)
Red meat	Male	FAO	Alt		0.89 (0.89, 2.54)	0.41 (0.45, 12.66)
Red meat	Male	Sales	Alt		1.09 (1.09, 2.74)	0.34 (0.54, 15.43)
Red meat	Male	FFQ	Alt		-0.34 (-0.34, 1.6)	1.4 (0.11, 4.94)
Red meat	Male	HHBS	Alt		0.45 (0.45, 2.1)	0.64 (0.29, 8.15)
Sodium	---	Urinary sodium	Ref	0.39	---	---
Sodium	Female	DR	Alt		-0.02 (-0.02, 0.85)	1.02 (0.38, 2.34)
Sodium	Female	FFQ	Alt		0.47 (0.47, 1.29)	0.63 (0.69, 3.64)
Sodium	Male	DR	Alt		-0.06 (-0.06, 0.8)	1.06 (0.38, 2.23)
Sodium	Male	FFQ	Alt		0.43 (0.43, 1.26)	0.65 (0.67, 3.52)
SSBs	---	DR	Ref	0.61	---	---
SSBs	Female	Sales	Alt		0.15 (0.15, 1.43)	0.86 (0.37, 4.17)
SSBs	Female	FFQ	Alt		-0.01 (-0.01, 1.32)	1.01 (0.3, 3.75)
SSBs	Female	HHBS	Alt		-0.59 (-0.59, 0.68)	1.8 (0.18, 1.98)
SSBs	Male	Sales	Alt		0.35 (0.35, 1.63)	0.7 (0.45, 5.1)
SSBs	Male	FFQ	Alt		0.19 (0.19, 1.53)	0.83 (0.37, 4.6)
SSBs	Male	HHBS	Alt		-0.39 (-0.39, 0.89)	1.48 (0.22, 2.43)
Trans fat	---	DR	Ref	0.22	---	---
Trans fat	Male	Sales	Alt		-0.23 (-1.27,0.94)	1.25 (0.28, 2.55)
Trans fat	Female	Sales	Alt		-0.23 (-1.27,0.94)	1.25 (0.28, 2.55)
Trans fat	Male	FFQ	Alt		0.59 (-2.72,4.23)	0.56 (0.07,68.72)
Trans fat	Female	FFQ	Alt		0.86 (-2.63,4.9)	0.42 (0.07,134.0)
Vegetables	---	DR	Ref	0.64	---	---
Vegetables	Female	FAO	Alt		0.12 (0.12, 1.33)	0.89 (0.31, 3.78)
Vegetables	Female	Sales	Alt		0.62 (0.62, 1.83)	0.54 (0.51, 6.21)
Vegetables	Female	FFQ	Alt		-0.05 (-0.05, 1.16)	1.05 (0.26, 3.18)
Vegetables	Female	HHBS	Alt		0.1 (0.1, 1.31)	0.91 (0.3, 3.69)
Vegetables	Male	FAO	Alt		0.16 (0.16, 1.37)	0.85 (0.32, 3.94)
Vegetables	Male	Sales	Alt		0.66 (0.66, 1.87)	0.52 (0.53, 6.49)
Vegetables	Male	FFQ	Alt		-0.01 (-0.01, 1.2)	1.01 (0.27, 3.32)
Vegetables	Male	HHBS	Alt		0.14 (0.14, 1.35)	0.87 (0.32, 3.85)
Whole grains	---	DR	Ref	0.69	---	---
Whole grains	Female	FAO	Alt		1.94 (1.94, 3.37)	0.14 (1.82, 29.05)
Whole grains	Female	FFQ	Alt		-0.35 (-0.35, 1.37)	1.42 (0.13, 3.94)
Whole grains	Male	FAO	Alt		2.09 (2.09, 3.52)	0.12 (2.12, 33.76)
Whole grains	Male	FFQ	Alt		-0.2 (-0.2, 1.52)	1.22 (0.15, 4.58)

**Adjustment factor is the transformed beta coefficient in normal space and can be interpreted as the factor by which the alternative case definition is adjusted to reflect what it would have been if measured as the reference.*

Modelling strategy

Exposure model

We use a spatiotemporal Gaussian process regression (ST-GPR) framework to estimate the mean intake of each dietary factor by age, sex, country, and year. In GBD 2019, we removed lag-distributed income as a covariate from most of our models and added country-level energy availability (Table 2).

To characterise the distribution of each dietary factor at the population level, we use an ensemble approach that separately fit 12 distributions for individual-level microdata to specific to each data source's sampled population. The respective goodness of fit of each family was assessed, and a weighting scheme was determined to optimise overall fit to the unique distribution of each risk factor. A global mean of the weights for each risk factor's data sources was created. We then determined the standard deviation of each population's consumption through a linear regression that captured the relationship between the standard deviation and mean of intake in nationally representative nutrition surveys using 24-hour diet recalls:

$$ln\ (Standard\ deviation) = \beta_0 + \beta_1 \times ln\ (Mean_i)$$

Then we applied the coefficients of this regression to the outputs of our ST-GPR model to calculate the standard deviation of intake by age, sex, year, and country. We also quantified the within-person variation in consumption of each dietary component and adjusted the standard deviations accordingly.

Theoretical minimum-risk exposure level

The dietary TRMELs were updated for GBD 2019. For harmful dietary risks other than sodium, TMREL was set to zero. For protective dietary risk factors, we first calculated the level of intake associated with the lowest risk of mortality from each disease endpoint based on the 85th percentile of intake across all epidemiological studies included in the meta-analysis of the risk-outcome pair. Then we calculated the TMREL as the weighted average of these numbers using the global number of deaths from each outcome as the weight.

Table 4**. Theoretical minimum-risk exposure level for dietary factors, GBD 2017 and GBD 2019**

Dietary factor	GBD 2017	GBD 2019
Fruits	200-300 g/day	310-340 g/day
Vegetables	290-430 g/day	280-320 g/day
Whole grains	100-150 g/day	140-160 g/day
Nuts	16-25 g/day	10-19 g/day
Red meats	18-27 g/day	0 g/day
Processed meats	0-4 g/day	0 g/day

Milk	350-520 g/day	360-500 g/day
Legumes	50-70 g/day	90-100 g/day
Sugar sweetened beverages	0-5 g/day	0 g/day
Polyunsaturated fatty acids	9-13% of total daily energy	7-9% of total daily energy
Seafood omega-3 fatty acids	200-300 mg/day	430-470 mg/day
Trans fatty acids	0-1% of total daily energy	0% of total daily energy
Dietary fibre	19-28 g/day	21-22 g/day
Dietary calcium	1.0-1.3 g/day	1.06 – 1.1 g/day
Dietary sodium	1-5 g/day	1-5 g/day

Relative risks

For GBD 2019, we performed systematic reviews for each dietary risk and its related outcomes. Using the sources identified during these searches, we incorporated the most recent epidemiological evidence assessing the relationship between each GBD dietary risk factor and related outcomes in our relative risk analysis. After evaluating all available evidence, we found sufficient evidence on the casual relationship for 8 new R-O pairs and insufficient evidence for 5 old R-O pairs. Based on these results, we updated the R-O pairs used the GBD dietary risk factor analysis in the following ways:

Removed:

Diet low in fruit and nasopharynx cancer
Diet low in fruit and other pharynx cancer
Diet low in fruit and oesophageal cancer
Diet low in fruit and larynx cancer
Diet low in whole grains and haemorrhagic stroke

Added:

Diet low in whole grains and colon and rectum cancer
Diet high in red meat and breast cancer
Diet high in red meat and ischaemic heart disease
Diet high in red meat and haemorrhagic stroke
Diet high in red meat and ischaemic stroke
Diet low in fibre and ischaemic stroke
Diet low in fibre and haemorrhagic stroke
Diet low in fibre and diabetes mellitus

Additionally, based on the most recent epidemiological evidence and GBD 2019 newly developed methods for characterising the risk curve, we updated the dose-response curve of relative risks for all dietary risks. For sodium, we continued to estimate its effect on cardiovascular disease based on the effect of sodium on systolic blood pressure.

There is a well-documented attenuation of the risk for cardiovascular disease due to metabolic risks factors throughout one's life. To incorporate this age trend in the relative risks, we first identified the

median age-at-event across all cohorts and considered that as the reference age group. We then assigned our newly estimated risk curves to this reference age group. Then, we derived the percentage change in relative risks between each age group and the reference age group by averaging percentage changes in relative risks of all metabolic mediators. The three cardiovascular disease outcomes for dietary risks are haemorrhagic stroke (including intracerebral hemmorhage and subarachnoid hemmorhage), ischaemic stroke, and ischaemic heart disease, and the effects of dietary risks on them are mediated through high systolic blood pressure, cholesterol (not included for haemorrhagic stroke), and fasting plasma glucose. Since the effect of diet is estimated independently of body-mass index (BMI) in the GBD, BMI was not included as a mediator in the RR age trend analysis.

References

1. Schmidhuber, Josef, et al. The Global Nutrient Database: Availability of Macronutrients and Micronutrients in 195 Countries from 1980 to 2013. *The Lancet Planetary Health*, vol. 2, no. 8, 2018, doi:10.1016/s2542-5196(18)30170-0.

Low physical activity

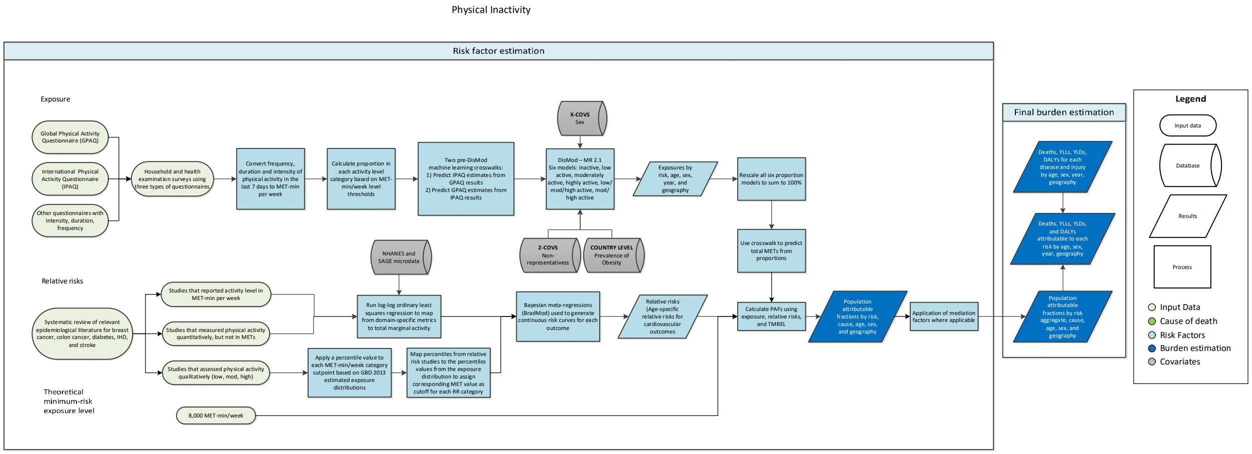

Input data and methodological summary

Exposure

Case definition

We measure physical activity performed by adults older than 25 years of age, for duration of at least ten minutes at a time, across all domains of life (leisure/recreation, work/household and transport). We use frequency, duration and intensity of activity to calculate total metabolic equivalent-minutes per week. MET (Metabolic Equivalent) is the ratio of the working metabolic rate to the resting metabolic rate. One MET is equivalent to 1 kcal/kg/hour and is equal to the energy cost of sitting quietly. A MET is also defined as the oxygen uptake in ml/kg/min with one MET equal to the oxygen cost of sitting quietly, around 3.5 ml/kg/min.

Input data

We included surveys of the general adult population that captured self-reported physical activity in all domains of life (leisure/recreation, work/household and transport), where random sampling was used.

Data were primarily derived from two standardised questionnaires: The Global Physical Activity Questionnaire (GPAQ) and the International Physical Activity Questionnaire (IPAQ), although we included other survey instruments that asked about intensity, frequency and duration of physical activities performed across all activity domains.

Due to a lack of a consistent relationship on the individual level between activity performed in each domain and total activity, we were not able to use studies that included only recreational/leisure activities.

Physical activity level is categorised by total MET-minutes per week using four categories based on rounded values closest to the quartiles of the global distribution of total MET-minutes/week. The lower limit for the Level 1 category (600 MET-min/week) is the recommended minimum amount of physical activity to get any health benefit. We used four categories with higher thresholds rather than the GPAQ and IPAQ recommended 3 categories to better capture any additional protective effects from higher activity levels.

- Level 0: < 600 MET-min/week (inactive)
- Level 1: 600-3999 MET-min/week (low-active)
- Level 2: 4000-7,999 MET-min/week (moderately-active)
- Level 3: ≥ 8,000 MET-min/week (highly active)

The GHDx was used to locate all surveys that use the GPAQ or IPAQ questionnaire. Although there were many other surveys that focused specifically on leisure activity, we were unable to use these sources because they did not comprise all three domains (work, transport and leisure). In addition, we excluded any surveys that did not report frequency, duration, and intensity of activity.

Table 1: Data inputs for exposure for low physical activity.

Input data	Exposure
Site-years (total)	255
Number of countries with data	128

Table 2: Data inputs for relative risks for low physical activity.

Input data	Exposure
Site-years (total)	121
Number of countries with data	38

Modelling strategy

DisMod modelling

For this round of the GBD, we have chosen to use a machine learning crosswalk to predict IPAQ estimates for GPAQ results and GPAQ estimates for IPAQ results, with original and estimated results then being combined to get one comprehensive IPAQ dataset and one comprehensive GPAQ dataset. We then estimated the proportion of each country/year/age/sex subpopulation in each of the above four activity levels using 12 separate Dismod models (one set of six for IPAQ and one for GPAQ). We use six categories of physical activity prevalence rather than four to accommodate the different MET-minute/week cutoffs presented in tabulated data sources where individual unit record data was not available. Since the accepted threshold/definition for inactivity is consistently <600 MET-minutes/week, the vast majority of tabulated data was broken down into proportion inactive (model A) and proportion low, moderate or highly active (model B).

	Label	MET-min/week	Name of sequelae in online visualisation tool
A	inactive	<600	Physical inactivity and low physical activity, inactive
B	low/moderately/highly active	≥600	Physical inactivity and low physical activity, low/moderately/highly active
C	low active	600-3999	Physical inactivity and low physical activity, low active
D	moderately/highly active	>4000	Physical inactivity and low physical activity, moderately/highly active
E	moderately active	4000-7999	Physical inactivity and low physical activity, moderately active
F	highly active	≥8,000	Physical inactivity and low physical activity, highly active

These models have mesh points at 0 15 25 35 45 55 65 75 85 100, and a study-level fixed effect on integrand variance (Z-cov) for whether a study was nationally representative or not, to account for the heterogeneity introduced by studies that are not generalizable to the entire population. They also have national level fixed effects on prevalence of obesity.

After DisMod, we rescale each of the 6 models specific to each data source so that the proportions sum to one. Since we have the most data for models A and B, we rescale the sum of the proportion in each category to be equal to one. Next we rescale the sum of model C and D to be equal to the rescaled value from model B. Then we rescale the sum of models E and F to be equal to the rescaled value from model D. After these three rescales we are left with a proportion for each of the four categories that all sum to 1. Scaled results for each data source are then hybridised to produce only one set of results for the prevalence of the four categories of physical activity.

Similar to the previous round, we have not directly estimated total MET-minutes per week globally. Although, this year we made use of two specific machine learning algorithms (Random Forest and XGBoost) that were trained using data that could characterise the relationship between total MET-mins/week and each of the categorical prevalences of physical activity. This resulted in country-year-age-sex specific estimates of total physical activity in the form of MET-minutes per week.

Utilising microdata on total MET-mins per week from individual-level surveys, we characterised the distribution of activity level at the population level. We then used an ensemble approach to distribution fitting, borrowing characteristics from individual distributions to tailor a unique distribution to fit the data using a weighting scheme. We characterised the standard deviation of each population's activity through a linear regression that captured the relationship between standard deviation and mean activity levels in nationally representative IPAQ surveys:

$$ln\,(Standard\ deviation) = \beta_0 + \beta_1 \times ln\,(Mean_i) + \beta_2 \times Age_i + \beta_3 \times SR_i + \beta_4 \times Fem_i$$

Age_i is the youngest age in population *i*'s age group, SR_i is the super region in which the population lives, and Fem_i is a Boolean value depicting whether the population is female. We then applied the coefficients of this regression to the outputs of our estimate of total MET-minutes per week regression outputs to calculate the standard deviation by country, year, age, and sex.

Theoretical minimum-risk exposure level

The theoretical minimum-risk exposure level for physical inactivity is 3000-4500 MET-min per week, which was calculated as the exposure at which minimal deaths across outcomes occurred.[3]

Relative risk

We used a dose-response meta-analysis of prospective cohort studies to estimate the effect size of the change in physical activity level on breast cancer, colon cancer, diabetes, ischemic heart disease and ischemic stroke.[3]

There is a well-documented attenuation of the risk for cardiovascular disease and diabetes due to metabolic risks factors throughout one's life. To incorporate this age trend in the relative risks, we first identified the median age-at-event across all cohorts and considered that as the reference age-group. We then assigned our risk curves to this reference age group. Then, we derived the percent change in relative risks between each age group and the reference age group by averaging percentage changes in relative risks of all metabolic mediators.

References

1. IPAQ Research Committee. Guidelines for data processing and analysis of the International Physical Activity Questionnaire (IPAQ)–short and long forms. Retrieved September. 2005;17:2008.

2. World Health Organization. Global Physical Activity Questionnaire (GPAQ) Analysis Guide. 2011. Geneva, Switzerland: WHO Google Scholar. 2013

3. Kyu HH, Bachman VF, Alexander LT, Mumford JE, Afshin A, Estep K, Veerman JL, Delwiche K, Iannarone ML, Moyer ML, Cercy K. Physical activity and risk of breast cancer, colon cancer, diabetes, ischemic heart disease, and ischemic stroke events: systematic review and dose-response meta-analysis for the Global Burden of Disease Study 2013. bmj. 2016 Aug 9;354:i3857.

References

References are for the main appendix text. References for cause-specific and risk-specific write-ups are found at the end of each of those sections.

1. Vos T, Lim SS, Abbafati C, et al. Global burden of 369 diseases and injuries in 204 countries and territories, 1990–2019: a systematic analysis for the Global Burden of Disease Study 2019. The Lancet 2020;396:1204–1222.

2. Murray CJL, Aravkin AY, Zheng P, et al. Global burden of 87 risk factors in 204 countries and territories, 1990–2019: a systematic analysis for the Global Burden of Disease Study 2019. The Lancet 2020;396:1223–1249.

3. GBD 2017 Causes of Death Collaborators. Global, regional, and national age-sex-specific mortality for 282 causes of death in 195 countries and territories, 1980-2017: a systematic analysis for the Global Burden of Disease Study 2017. Lancet 2018;392:1736–1788.

4. GBD 2017 Disease and Injury Incidence and Prevalence Collaborators. Global, regional, and national incidence, prevalence, and years lived with disability for 354 diseases and injuries for 195 countries and territories, 1990-2017: a systematic analysis for the Global Burden of Disease Study 2017. Lancet 2018;392:1789–1858.

5. Kyu HH, Abate D, Abate KH, et al. Global, regional, and national disability-adjusted life-years (DALYs) for 359 diseases and injuries and healthy life expectancy (HALE) for 195 countries and territories, 1990–2017: a systematic analysis for the Global Burden of Disease Study 2017. The Lancet 2018;392:1859–1922.

6. GBD 2017 Risk Factor Collaborators. Global, regional, and national comparative risk assessment of 84 behavioural, environmental and occupational, and metabolic risks or clusters of risks for 195 countries and territories, 1990–2017: a systematic analysis for the Global Burden of Disease Study 2017. Lancet 2018;392:1923–1994.

7. WHO I. International Classification of Diseases (ICD). WHO Geneva, Switzerland; 2007.

8. Rudd KE, Johnson SC, Agesa KM, et al. Global, regional, and national sepsis incidence and mortality, 1990-2017: analysis for the Global Burden of Disease Study. Lancet 2020;395:200–211.

9. GBD 2016 Healthcare Access and Quality Collaborators. Measuring performance on the Healthcare Access and Quality Index for 195 countries and territories and selected subnational locations: a systematic analysis from the Global Burden of Disease Study 2016. Lancet 2018;391:2236–2271.

10. GBD 2017 Diet Collaborators. Health effects of dietary risks in 195 countries, 1990-2017: a systematic analysis for the Global Burden of Disease Study 2017. Lancet 2019;393:1958–1972.

11. Murray CJ, Lopez AD. Global mortality, disability, and the contribution of risk factors: Global Burden of Disease Study. Lancet 1997;349:1436–1442.

12. Murray CJ, Ezzati M, Lopez AD, Rodgers A, Vander Hoorn S. Comparative quantification of health risks conceptual framework and methodological issues. Popul Health Metr 2003;1:1.

13. GBD 2015 Risk Factors Collaborators. Global, regional, and national comparative risk assessment of 79 behavioural, environmental and occupational, and metabolic risks or clusters of risks, 1990-2015: a systematic analysis for the Global Burden of Disease Study 2015. Lancet 2016;388:1659–1724.

14. Fund WCR, Research AI for C. Food, nutrition, physical activity, and the prevention of cancer: a global perspective. Amer Inst for Cancer Research; 2007.

15. GBD 2016 Alcohol Collaborators. Alcohol use and burden for 195 countries and territories, 1990-2016: a systematic analysis for the Global Burden of Disease Study 2016. Lancet 2018;392:1015–1035.

16. Kurowicka D, Cooke RM. Sampling algorithms for generating joint uniform distributions using the vine-copula method. Computational Statistics & Data Analysis 2007;51:2889–2906.
