# Supplementary material for: Global Burden of Cardiovascular Diseases and Risk Factors, 1990–2019: Update From the GBD 2019 Study
Source: J Am Coll Cardiol. 2020 Dec 22;76(25):2982–3021. doi: 10.1016/j.jacc.2020.11.010 (PMC7755038; doi:10.1016/j.jacc.2020.11.010)
Supplement: Supplemental Data 2 [file mmc2.pdf]

Supplementary Tables and Figures for “Global Burden of Cardiovascular Diseases and Risk Factors, 1990-2019: Update from the Global Burden of Disease 2019 Study”

Contents

Supplementary Figure 1. Global Burden of Disease (GBD) Regions.. 4
Supplementary Figure 2. DALYs due to Cardiovascular Diseases in 2019 by Age.................................... 5
Supplementary Figure 3. Age-Standardized DALYs due to Cardiovascular Diseases in 2019 by Region .. 6
Supplementary Figure 4. Map of Age-Standardized DALYs due to Cardiovascular Diseases in 2019....... 7
Supplementary Figure 5. DALYs due to Ischemic Heart Disease in 2019 by Age 8
Supplementary Figure 6. Age-Standardized DALYs due to Ischemic Heart Disease in 2019 by Region.... 9
Supplementary Figure 7. Map of Age-Standardized DALYs due to Ischemic Heart Disease in 2019 10
Supplementary Figure 8. Total Numbers and Rates of Stroke... 11
Supplementary Figure 9. Prevalence of Stroke Survivors 13
Supplementary Figure 10. Total Numbers and Rates of Ischemic Stroke 14
Supplementary Figure 11. Total Numbers and Rates of Intracerebral Hemorrhage................................. 16
Supplementary Figure 12. Total Numbers and Rates of Subarachnoid Hemorrhage 18
Supplementary Figure 13. Map of Age-Standardized DALYs due to Stroke in 2019............................... 20
Supplementary Figure 14. Total Numbers and Rates of Hypertensive Heart Disease............................... 21
Supplementary Figure 15. DALYs due to Hypertensive Heart Disease in 2019 by Age 23
Supplementary Figure 16. Age-Standardized DALYs due to Hypertensive Heart Disease in 2019 by Region 24
Supplementary Figure 17. Total Numbers and Rates of Rheumatic Heart Disease 25
Supplementary Figure 18. Age-Standardized DALYs due to Rheumatic Heart Disease in 2019 by Region 27
Supplementary Figure 19. Total Numbers and Rates of Cardiomyopathy and Myocarditis 28
Supplementary Figure 20. Age-Standardized DALYs due to Other Cardiomyopathy in 2019 by Region 30
Supplementary Figure 21. Total Numbers and Rates of Alcoholic Cardiomyopathy 31
Supplementary Figure 22. DALYs due to Alcoholic Cardiomyopathy in 2019 by Age 33
Supplementary Figure 23. Total Numbers and Rates of Atrial Fibrillation and Flutter 34
Supplementary Figure 24. DALYs due to Atrial Fibrillation and Flutter in 2019 by Age 36
Supplementary Figure 25. YLLs due to Aortic Aneurysm in 2019 by Age 37
Supplementary Figure 26. Map of Age-Standardized YLLs due to Aortic Aneurysm in 2019................. 38
Supplementary Figure 27. Total Numbers and Rates of Non-Rheumatic Calcific Aortic Valve Disease.. 39

Supplementary Figure 28. Total Numbers and Rates of Non-Rheumatic Degenerative Mitral Valve Disease 41

Supplementary Figure 29. Map of Age-Standardized DALYs due to Non-Rheumatic Degenerative Mitral Valve Disease in 2019 43

Supplementary Figure 30. Age-Standardized DALYs due to Non-Rheumatic Degenerative Mitral Valve Disease in 2019 by Region 44

Supplementary Figure 31. DALYs due to Endocarditis in 2019 by Age 45

Supplementary Figure 32. Age-Standardized DALYs due to Endocarditis in 2019 by Region 46

Supplementary Figure 33. Map of Age-Standardized DALYs due to Endocarditis in 2019 47

Supplementary Figure 34. Total Numbers and Rates of Peripheral Artery Disease 48

Supplementary Figure 35. Age-Standardized DALYs due to Peripheral Artery Disease in 2019 by Region 50

Supplementary Figure 36. Total Numbers and Rates of High Systolic Blood Pressure 51

Supplementary Figure 37. Age-Standardized DALYs due to High Systolic Blood Pressure in 2019 by Region 53

Supplementary Figure 38. Map of Age-Standardized DALYs due to High Systolic Blood Pressure in 2019 54

Supplementary Figure 39. DALYs due to High Fasting Plasma Glucose in 2019 by Age 55

Supplementary Figure 40. Age-Standardized DALYs due to High Fasting Plasma Glucose in 2019 by Region 56

Supplementary Figure 41. Map of Age-Standardized DALYs due to High Fasting Plasma Glucose in 2019 57

Supplementary Figure 42. Total Numbers and Rates of High LDL Cholesterol 58

Supplementary Figure 43. DALYs due to High LDL Cholesterol in 2019 by Age 60

Supplementary Figure 44. Age-Standardized DALYs due to High LDL Cholesterol in 2019 by Region . 61

Supplementary Figure 45. DALYs due to High Body-mass Index in 2019 by Age 62

Supplementary Figure 46. Age-Standardized DALYs due to High Body-mass Index in 2019 by Region 63

Supplementary Figure 47. Map of Age-Standardized DALYs due to High Body-mass Index in 2019 64

Supplementary Figure 48. Total Numbers and Rates of Impaired Kidney Function 65

Supplementary Figure 49. Total Numbers and Rates of Chronic Kidney Disease 67

Supplementary Figure 50. DALYs due to Chronic Kidney Disease in 2019 by Age 69

Supplementary Figure 51. DALYs due to Impaired Kidney Function in 2019 by Age 70

Supplementary Figure 52. Age-Standardized DALYs due to Impaired Kidney Function in 2019 by Region 71

Supplementary Figure 53. Total Numbers and Rates of Ambient Particulate Matter Pollution 72

Supplementary Figure 54. Total Numbers and Rates of Household Air Pollution From Solid Fuels 74

Supplementary Figure 55. Map of Age-Standardized DALYs due to Household Air Pollution From Solid Fuels in 2019 76
Supplementary Figure 56. DALYs due to Ambient Particulate Matter Pollution in 2019 by Age 77
Supplementary Figure 57. DALYs due to Household Air Pollution From Solid Fuels in 2019 by Age 78
Supplementary Figure 58. DALYs due to Tobacco in 2019 by Age 79
Supplementary Figure 59. Age-Standardized DALYs due to Tobacco in 2019 by Region 80
Supplementary Figure 60. DALYs due to Dietary Risks in 2019 by Age 81
Supplementary Figure 61. Age-Standardized DALYs due to Dietary Risks in 2019 by Region 82
Supplementary Figure 62. Map of Age-Standardized DALYs due to Dietary Risks in 2019 83
Supplementary Figure 63. Total Numbers and Rates of Low Physical Activity 84
Supplementary Figure 64. DALYs due to Low Physical Activity in 2019 by Age 86
Supplementary Figure 65. Age-Standardized DALYs due to Low Physical Activity in 2019 by Region 87
Supplementary Table 1. Prevalence of High SBP in 1990 and 2019 88

Supplementary Figure 1. Global Burden of Disease (GBD) Regions

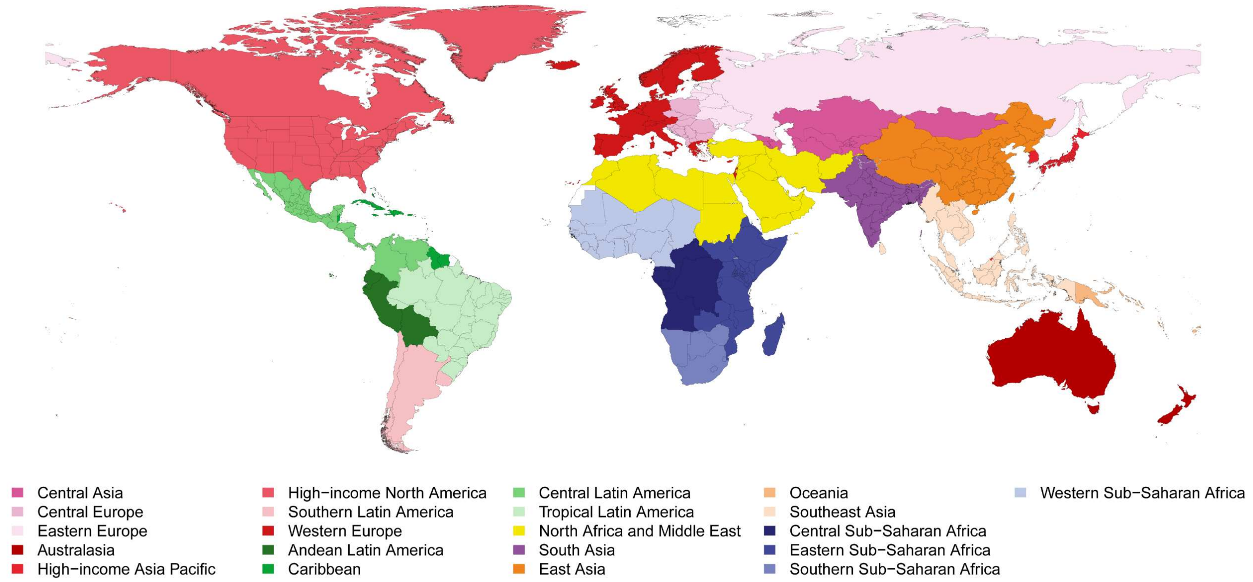

Map of 21 Global Burden of Disease (GBD) regions.

Supplementary Figure 2. DALYs due to Cardiovascular Diseases in 2019 by Age

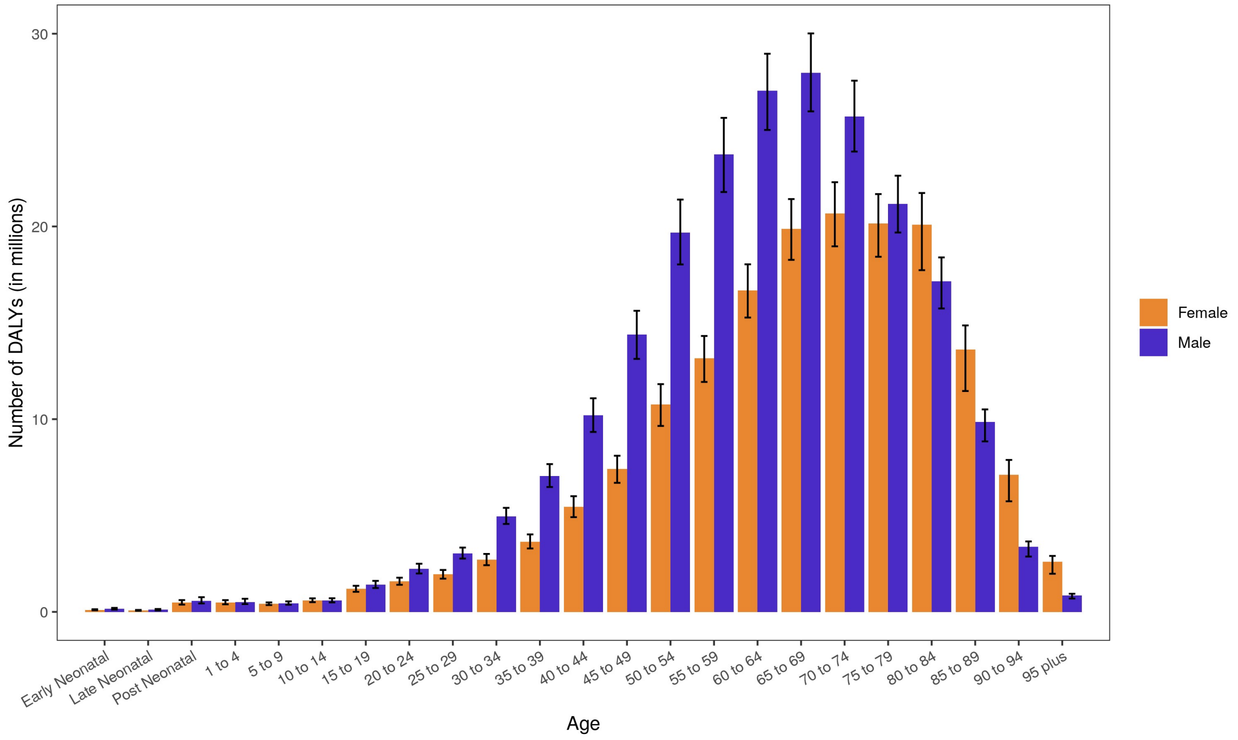

Number of disability-adjusted life years (DALYs) due to cardiovascular diseases by age and sex with 95% uncertainty intervals, 2019.

Supplementary Figure 3. Age-Standardized DALYs due to Cardiovascular Diseases in 2019 by Region

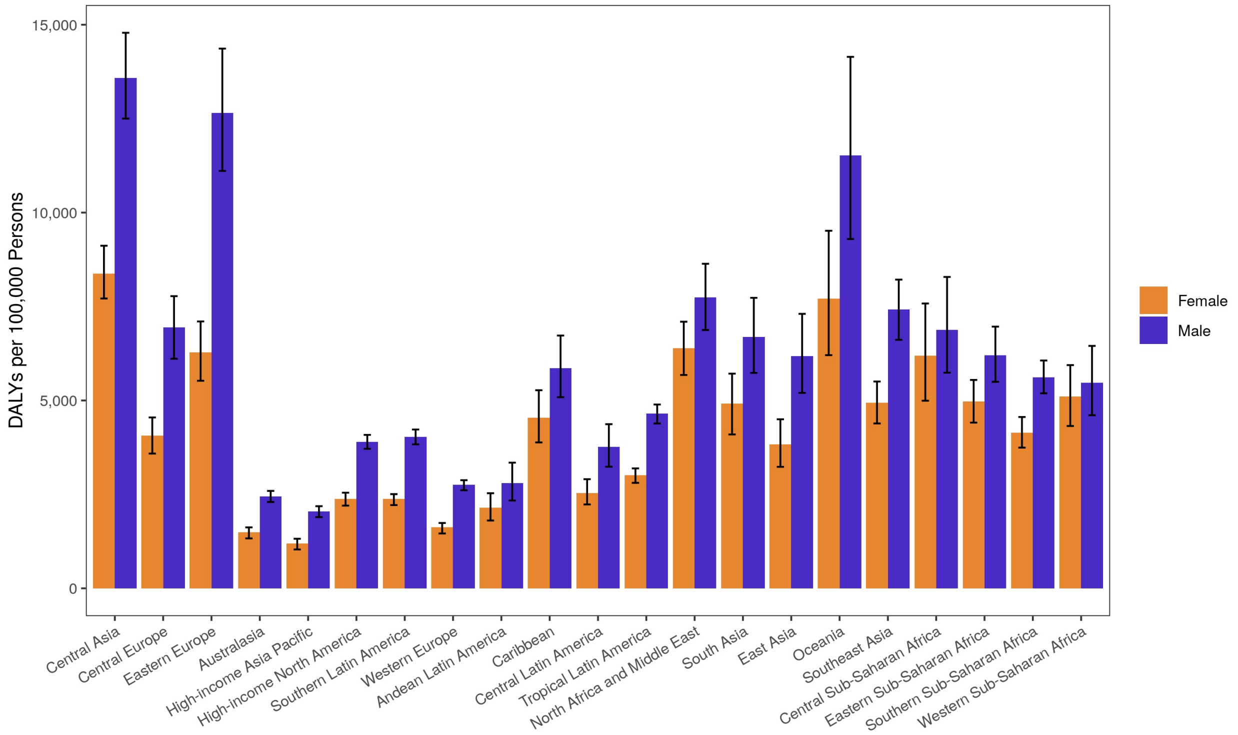

Age-standardized disability-adjusted life years (DALYs) rate of cardiovascular diseases by region and sex with 95% uncertainty intervals, 2019.

Supplementary Figure 4. Map of Age-Standardized DALYs due to Cardiovascular Diseases in 2019

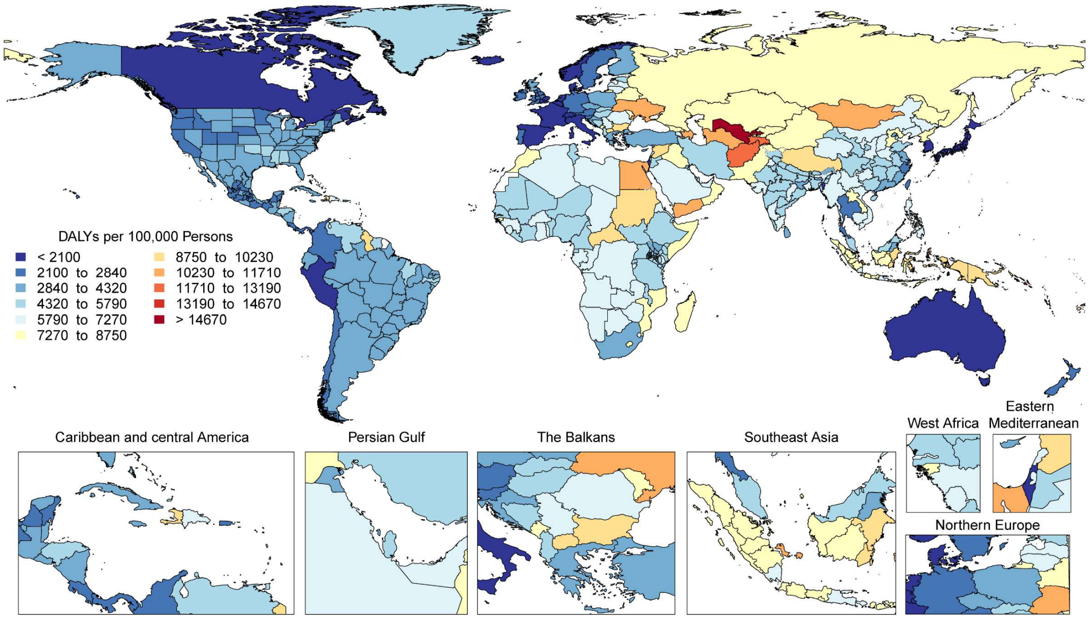

Map of age-standardized disability-adjusted life years (DALYs) rate of cardiovascular diseases, 2019.

Supplementary Figure 5. DALYs due to Ischemic Heart Disease in 2019 by Age

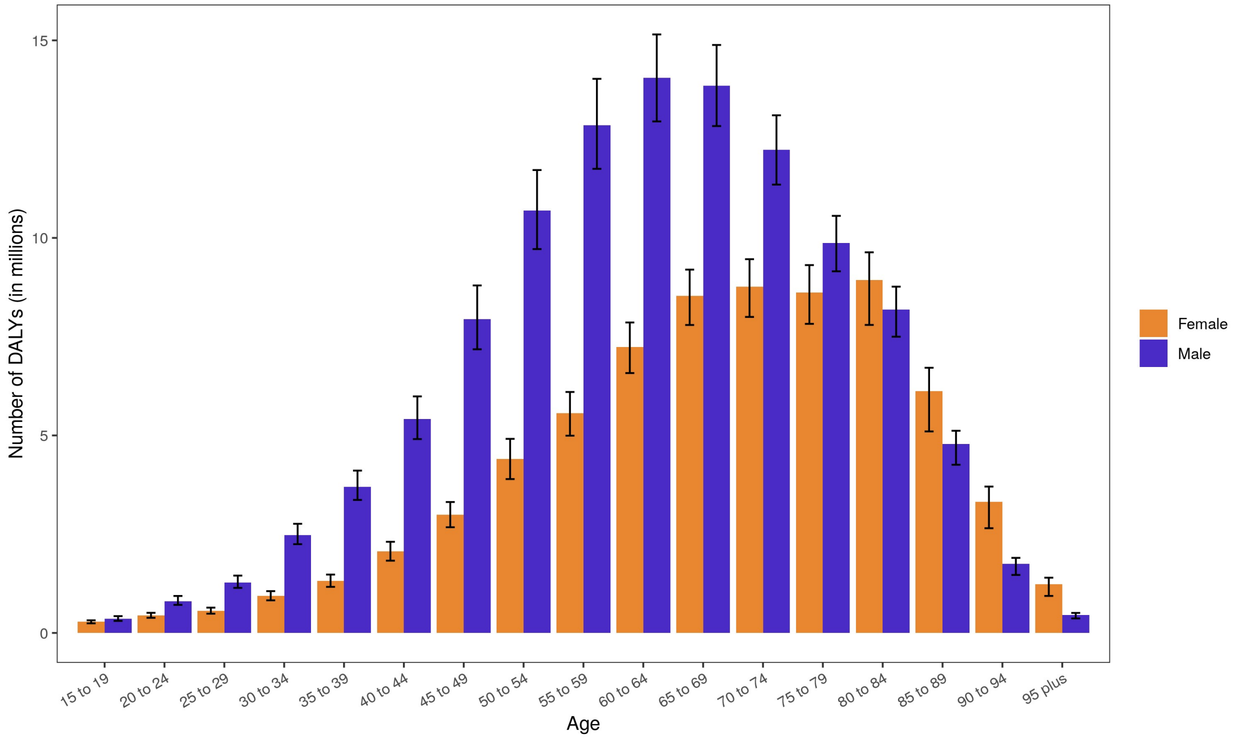

Number of disability-adjusted life years (DALYs) due to ischemic heart disease by age and sex with 95% uncertainty intervals, 2019. Ages below 15 were removed from the figure as they are not modeled for this cause.

Supplementary Figure 6. Age-Standardized DALYs due to Ischemic Heart Disease in 2019 by Region

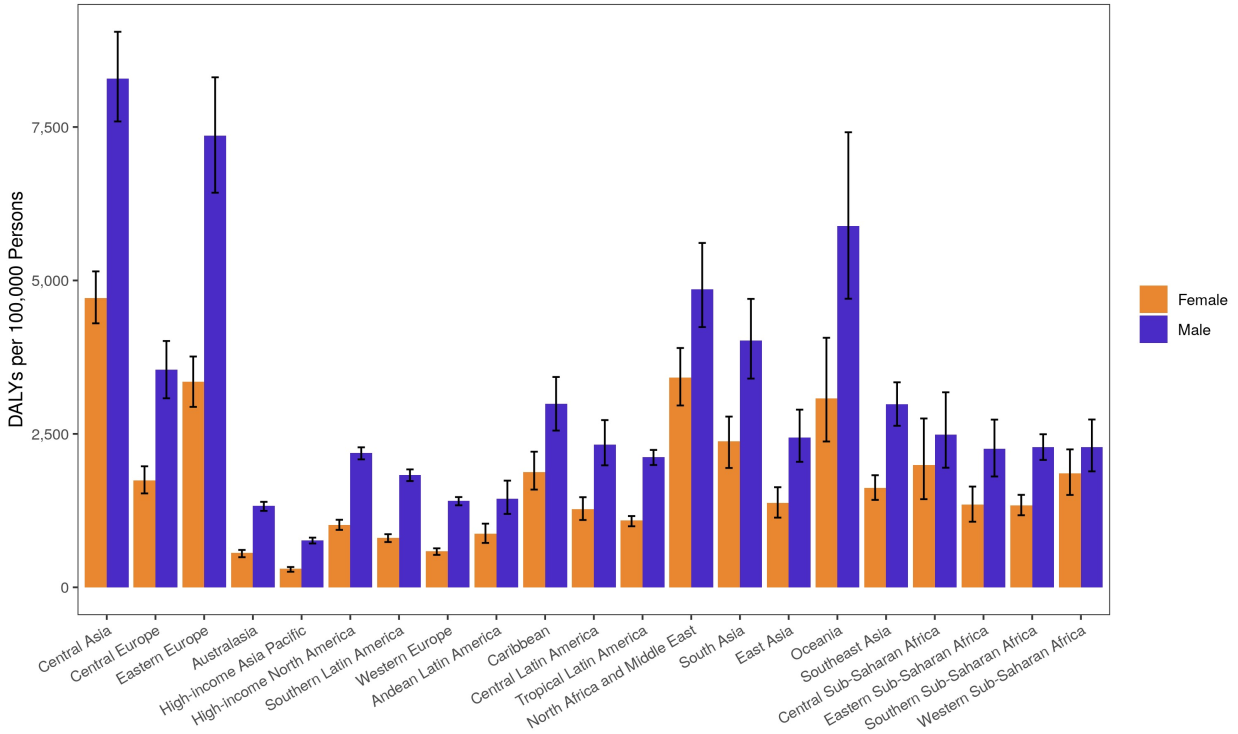

Age-standardized disability-adjusted life years (DALYs) rate of ischemic heart disease by region and sex with 95% uncertainty intervals, 2019.

Supplementary Figure 7. Map of Age-Standardized DALYs due to Ischemic Heart Disease in 2019

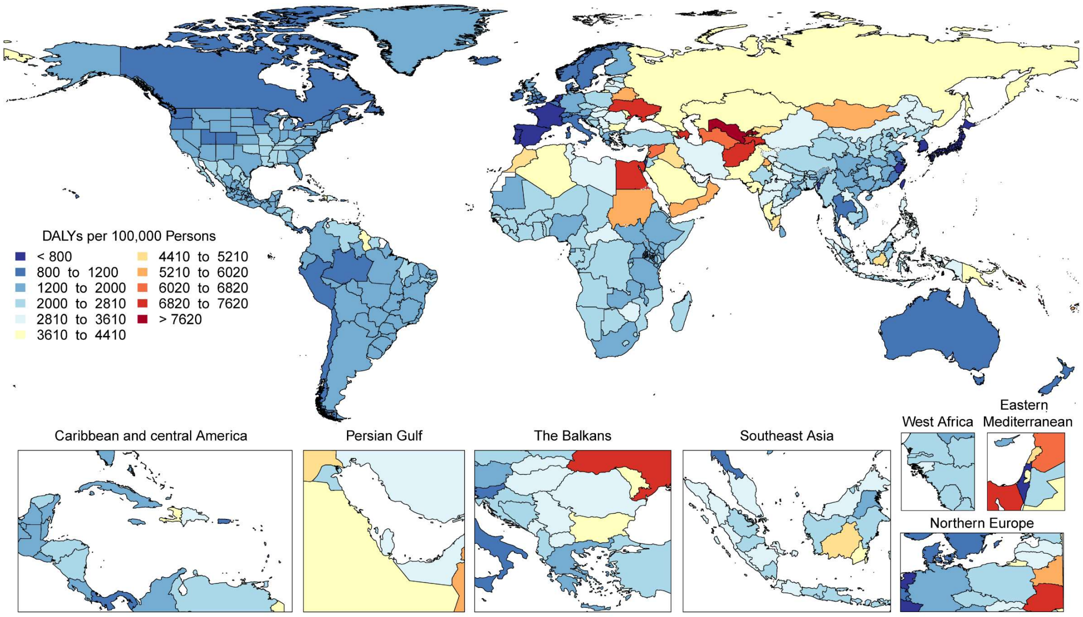

Map of age-standardized disability-adjusted life years (DALYs) rate of ischemic heart disease, 2019.

Supplementary Figure 8. Total Numbers and Rates of Stroke

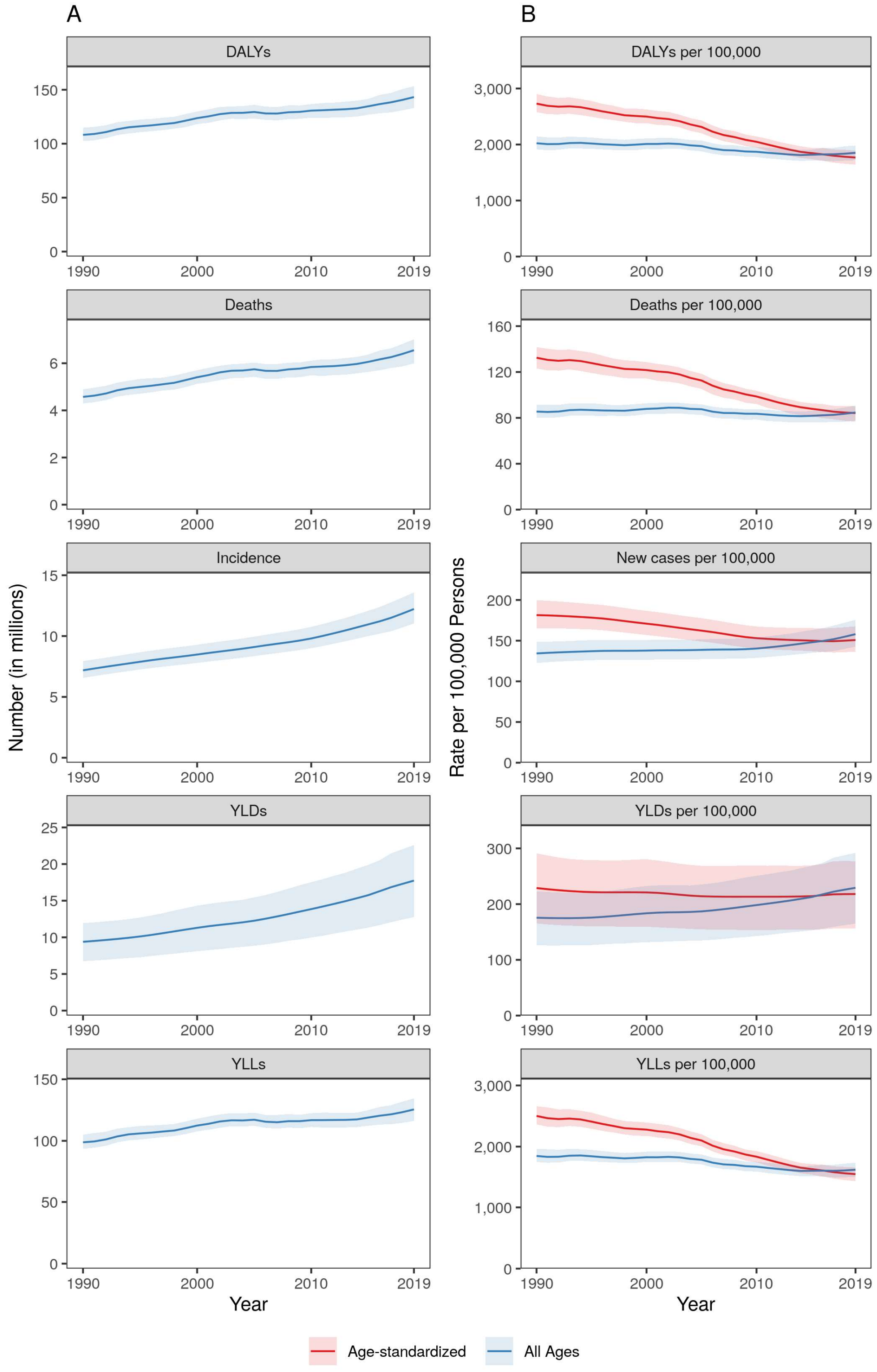

A. Total number of disability-adjusted life years (DALYs), deaths, incident cases, years lived with disability (YLDs), and years of life lost (YLLs) due to stroke, 1990 to 2019. Shaded regions

represent 95% uncertainty intervals. B. Age-standardized and all ages disability-adjusted life years (DALYs), death, incidence, years lived with disability (YLDs), and years of life lost (YLLs) rates of stroke, 1990 to 2019. Shaded regions represent 95% uncertainty intervals.

Supplementary Figure 9. Prevalence of Stroke Survivors

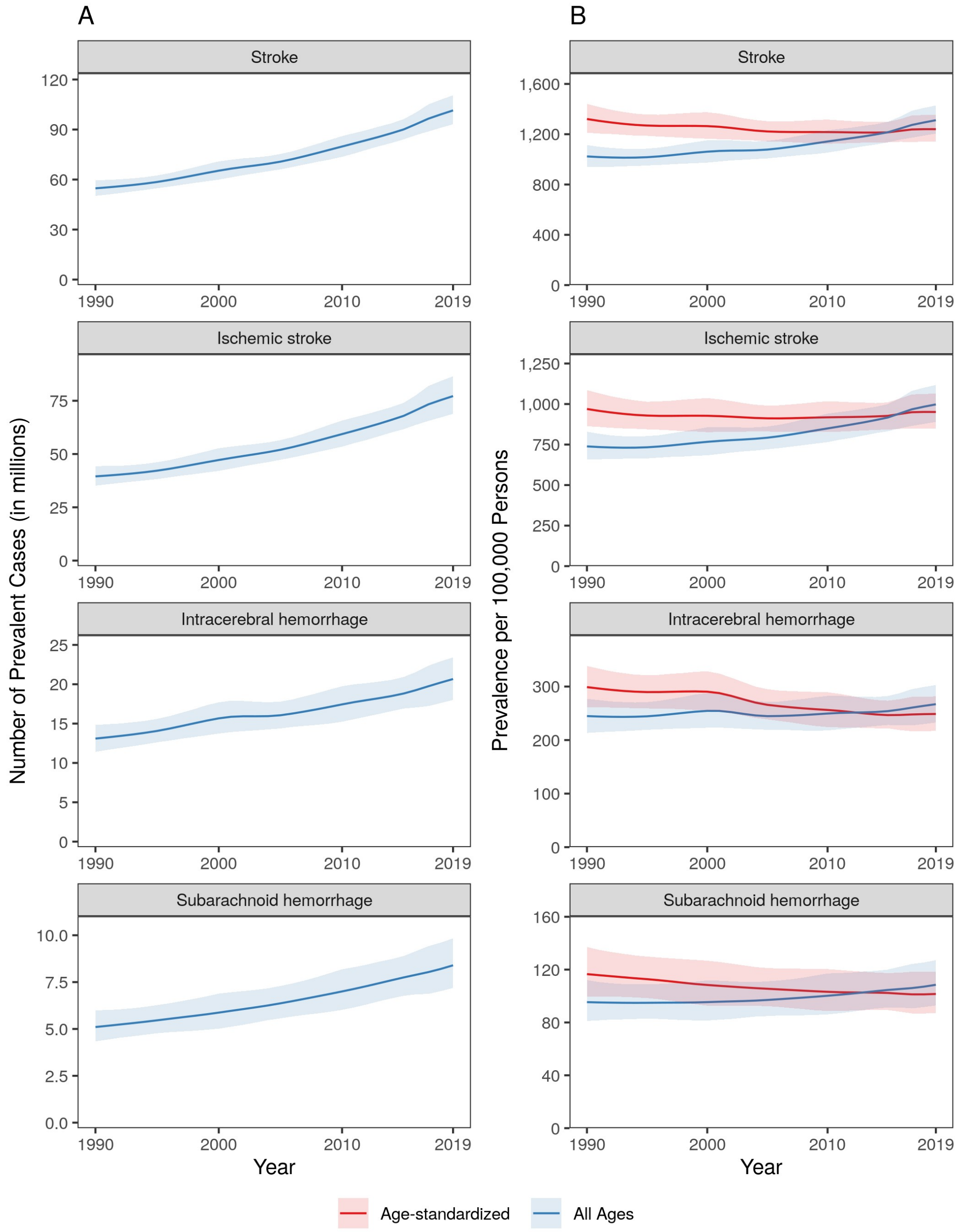

A. Total number of prevalent cases of stroke, ischemic stroke, intracerebral hemorrhage, and subarachnoid hemorrhage, 1990 to 2019. Shaded regions represent 95% uncertainty intervals. B. Age-standardized and all ages prevalence of stroke, ischemic stroke, intracerebral hemorrhage, and subarachnoid hemorrhage, 1990 to 2019. Shaded regions represent 95% uncertainty intervals.

Supplementary Figure 10. Total Numbers and Rates of Ischemic Stroke

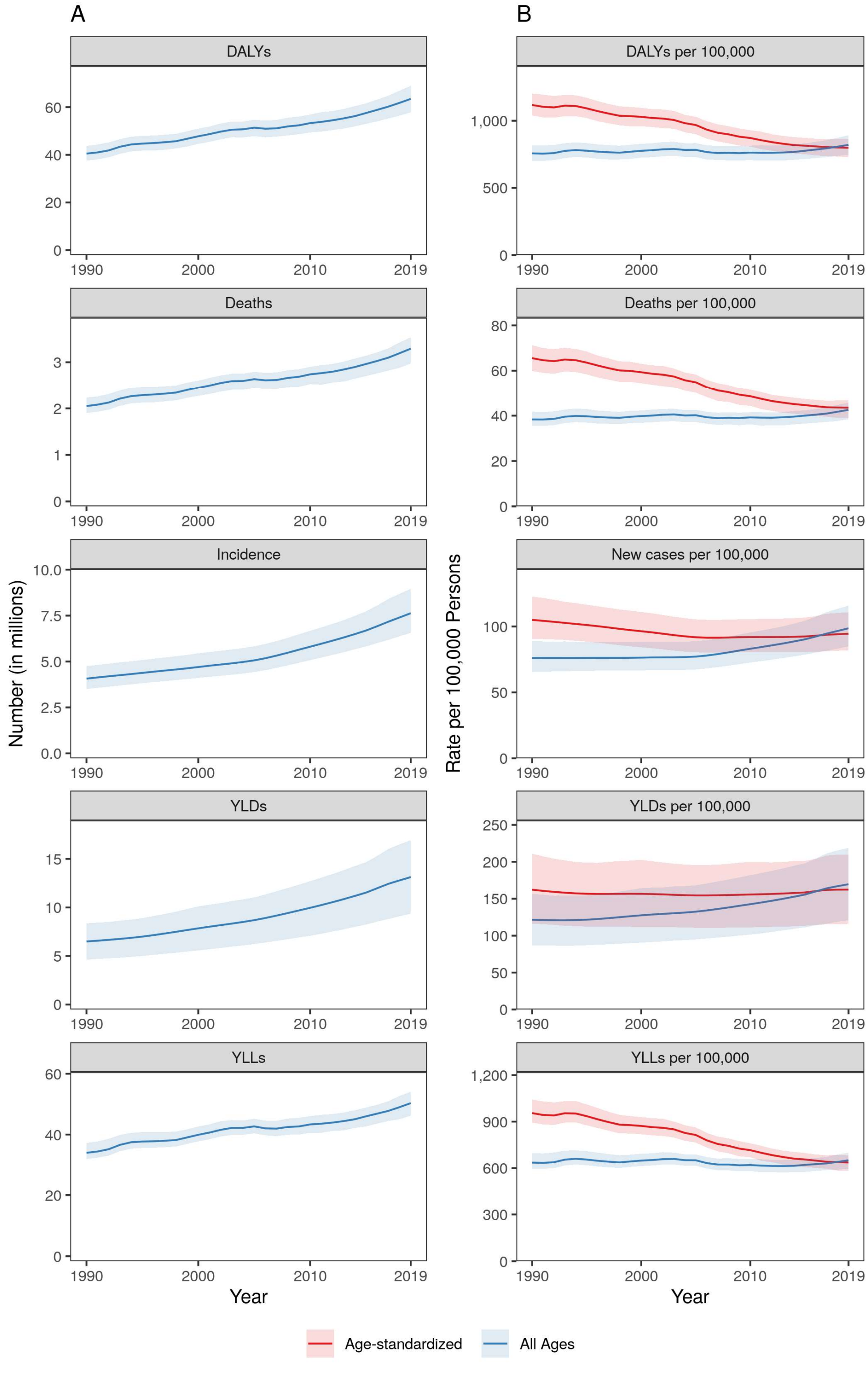

A. Total number of disability-adjusted life years (DALYs), deaths, incident cases, years lived with disability (YLDs), and years of life lost (YLLs) due to ischemic stroke, 1990 to 2019.

Shaded regions represent 95% uncertainty intervals. B. Age-standardized and all ages disability-adjusted life years (DALYs), death, incidence, years lived with disability (YLDs), and years of life lost (YLLs) rates of ischemic stroke, 1990 to 2019. Shaded regions represent 95% uncertainty intervals.

Supplementary Figure 11. Total Numbers and Rates of Intracerebral Hemorrhage

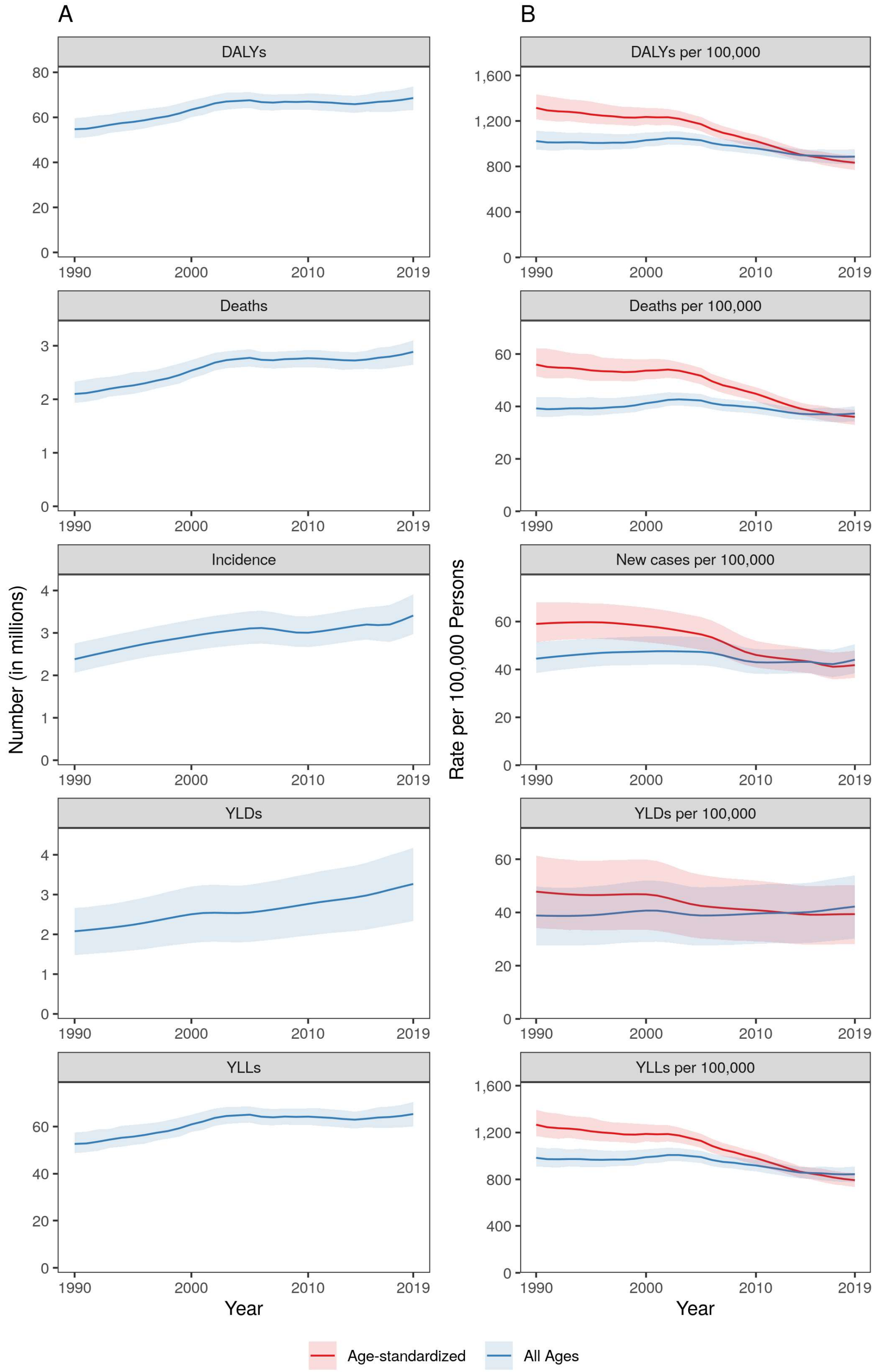

A. Total number of disability-adjusted life years (DALYs), deaths, incident cases, years lived with disability (YLDs), and years of life lost (YLLs) due to intracerebral hemorrhage, 1990 to

2019. Shaded regions represent 95% uncertainty intervals. B. Age-standardized and all ages disability-adjusted life years (DALYs), death, incidence, years lived with disability (YLDs), and years of life lost (YLLs) rates of intracerebral hemorrhage, 1990 to 2019. Shaded regions represent 95% uncertainty intervals.

Supplementary Figure 12. Total Numbers and Rates of Subarachnoid Hemorrhage

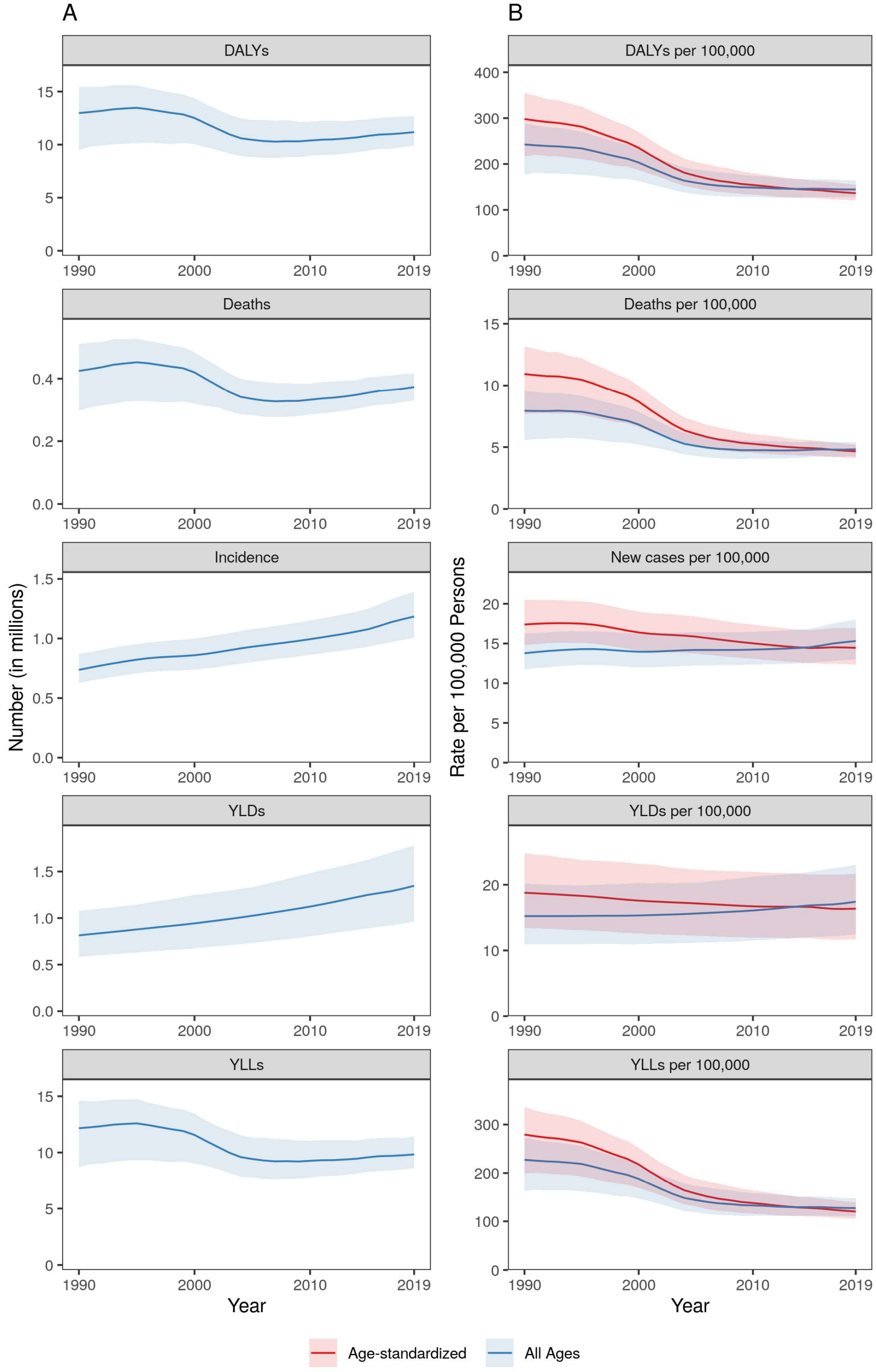

A. Total number of disability-adjusted life years (DALYs), deaths, incident cases, years lived with disability (YLDs), and years of life lost (YLLs) due to subarachnoid hemorrhage, 1990 to

2019. Shaded regions represent 95% uncertainty intervals. B. Age-standardized and all ages disability-adjusted life years (DALYs), death, incidence, years lived with disability (YLDs), and years of life lost (YLLs) rates of subarachnoid hemorrhage, 1990 to 2019. Shaded regions represent 95% uncertainty intervals.

Supplementary Figure 13. Map of Age-Standardized DALYs due to Stroke in 2019

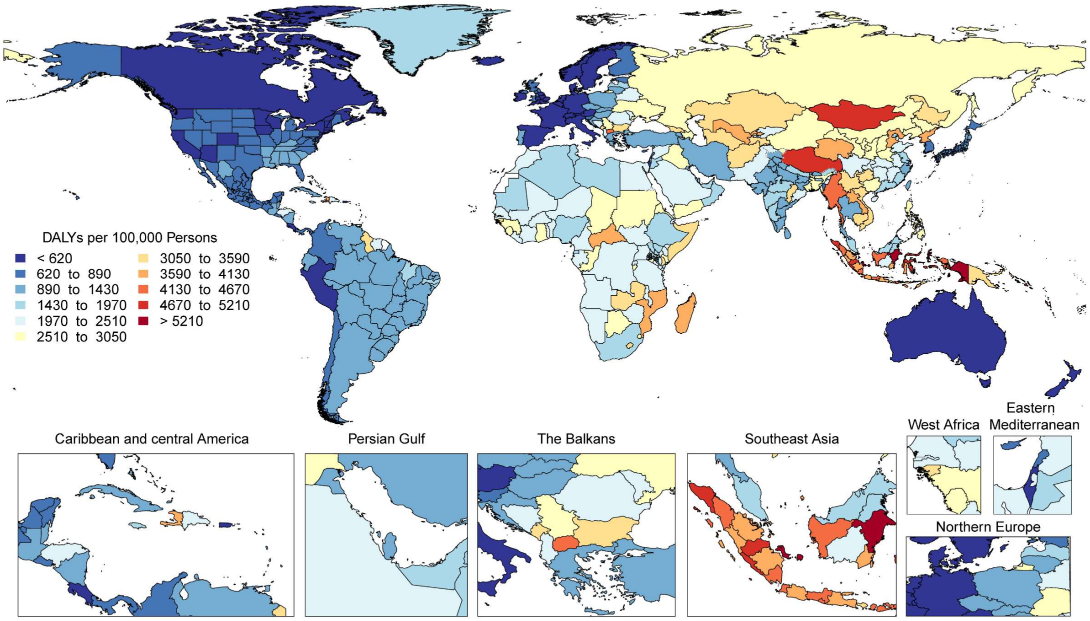

Map of age-standardized disability-adjusted life years (DALYs) rate of stroke, 2019.

Supplementary Figure 14. Total Numbers and Rates of Hypertensive Heart Disease

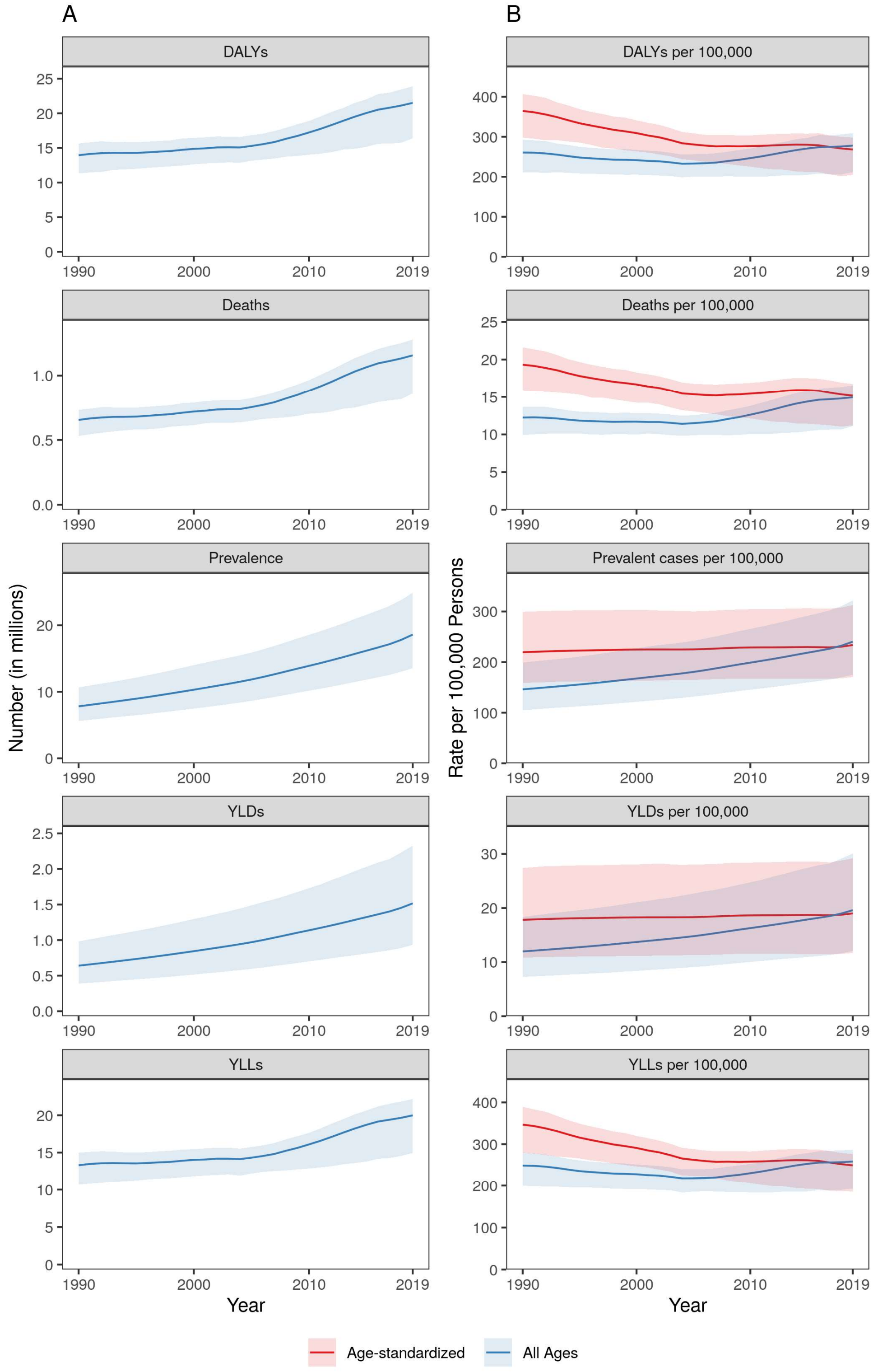

A. Total number of disability-adjusted life years (DALYs), deaths, prevalent cases, years lived with disability (YLDs), and years of life lost (YLLs) due to hypertensive heart disease, 1990 to

2019. Shaded regions represent 95% uncertainty intervals. B. Age-standardized and all ages disability-adjusted life years (DALYs), death, prevalence, years lived with disability (YLDs), and years of life lost (YLLs) rates of hypertensive heart disease, 1990 to 2019. Shaded regions represent 95% uncertainty intervals.

Supplementary Figure 15. DALYs due to Hypertensive Heart Disease in 2019 by Age

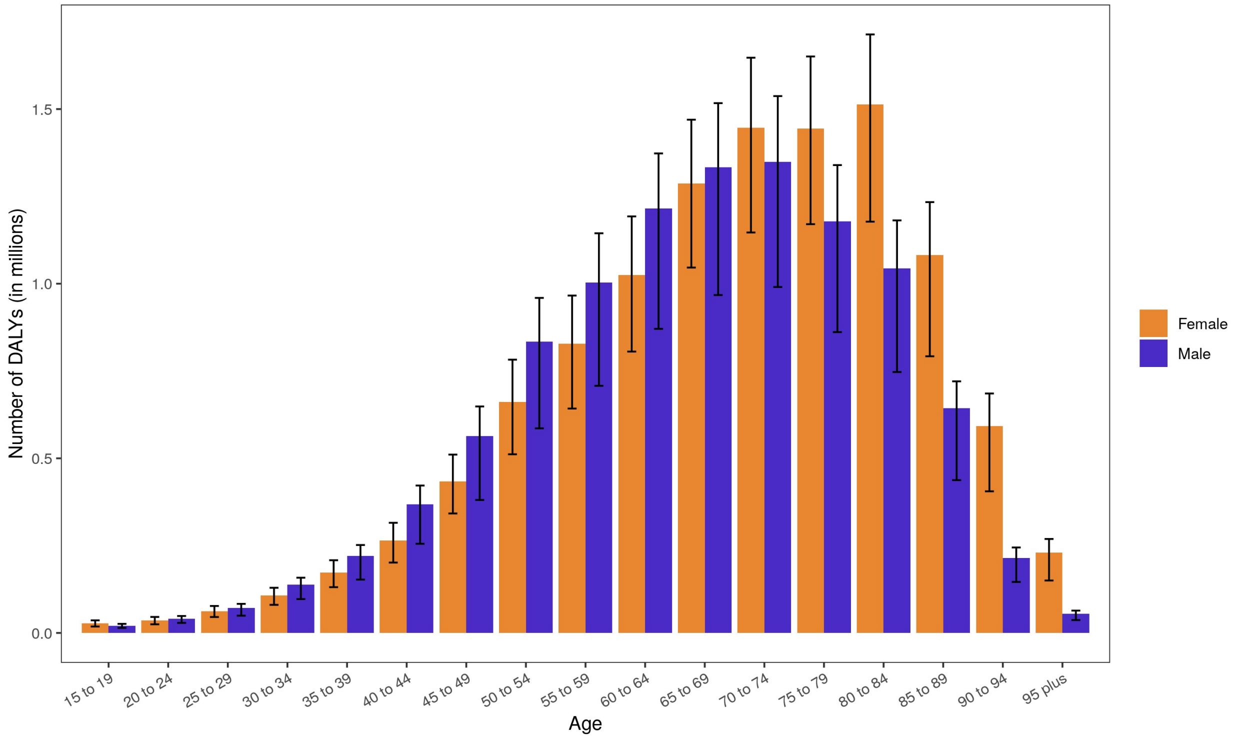

Number of disability-adjusted life years (DALYs) due to hypertensive heart disease by age and sex with 95% uncertainty intervals, 2019. Ages below 15 were removed from the figure as they are not modeled for this cause.

Supplementary Figure 16. Age-Standardized DALYs due to Hypertensive Heart Disease in 2019 by Region

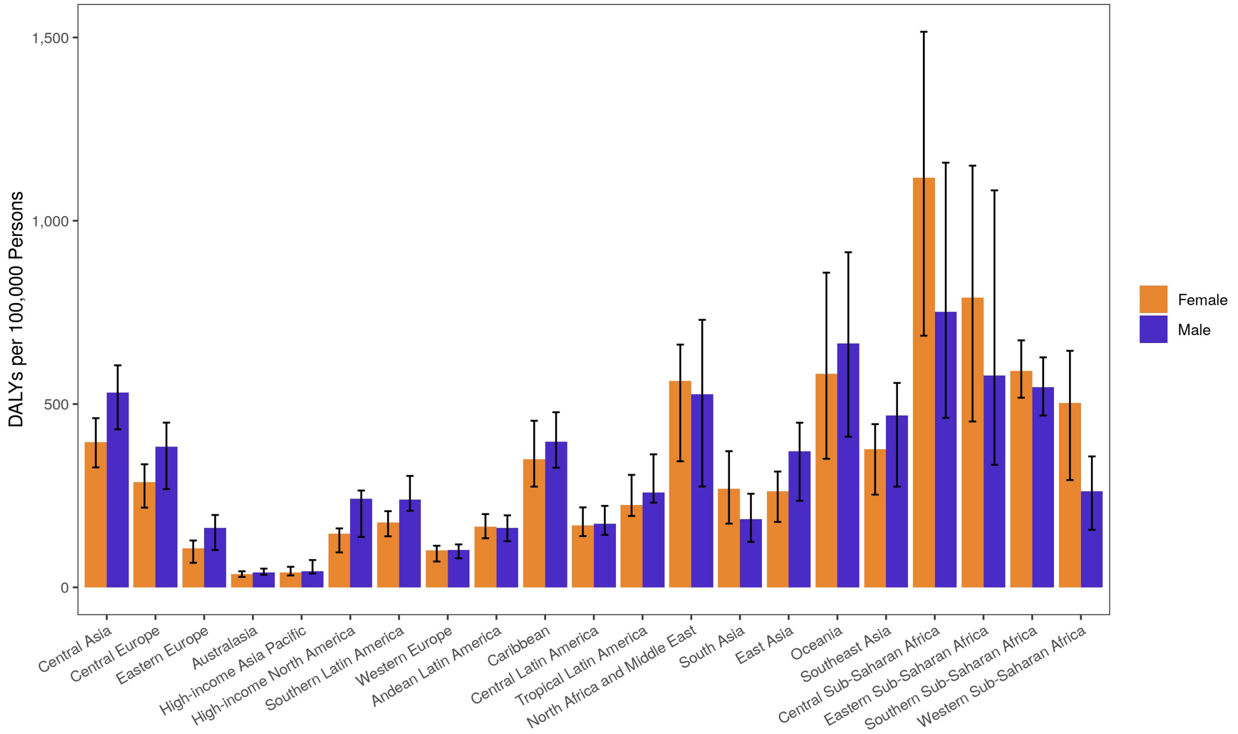

Age-standardized disability-adjusted life years (DALYs) rate of hypertensive heart disease by region and sex with 95% uncertainty intervals, 2019.

Supplementary Figure 17. Total Numbers and Rates of Rheumatic Heart Disease

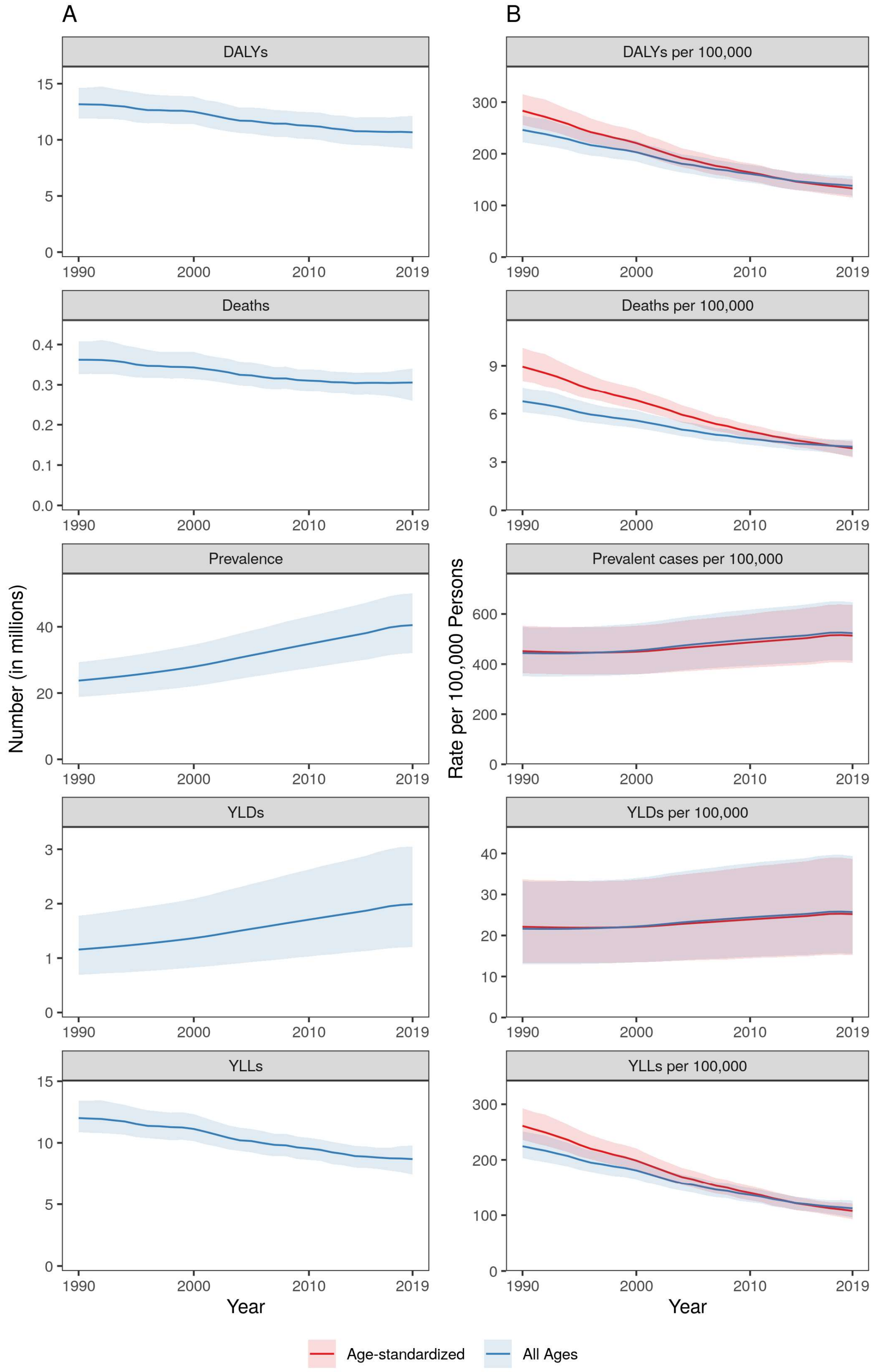

A. Total number of disability-adjusted life years (DALYs), deaths, prevalent cases, years lived with disability (YLDs), and years of life lost (YLLs) due to rheumatic heart disease, 1990 to

2019. Shaded regions represent 95% uncertainty intervals. B. Age-standardized and all ages disability-adjusted life years (DALYs), death, prevalence, years lived with disability (YLDs), and years of life lost (YLLs) rates of rheumatic heart disease, 1990 to 2019. Shaded regions represent 95% uncertainty intervals.

Supplementary Figure 18. Age-Standardized DALYs due to Rheumatic Heart Disease in 2019 by Region

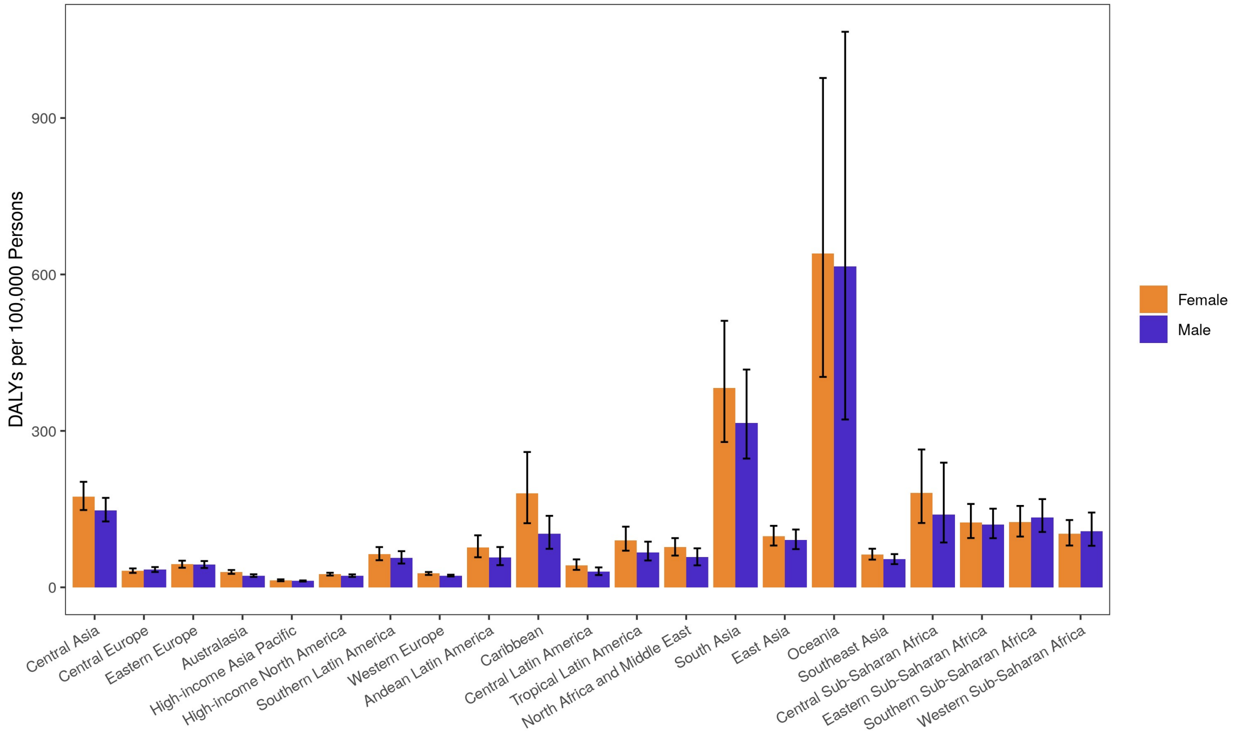

Age-standardized disability-adjusted life years (DALYs) rate of rheumatic heart disease by region and sex with 95% uncertainty intervals, 2019.

Supplementary Figure 19. Total Numbers and Rates of Cardiomyopathy and Myocarditis

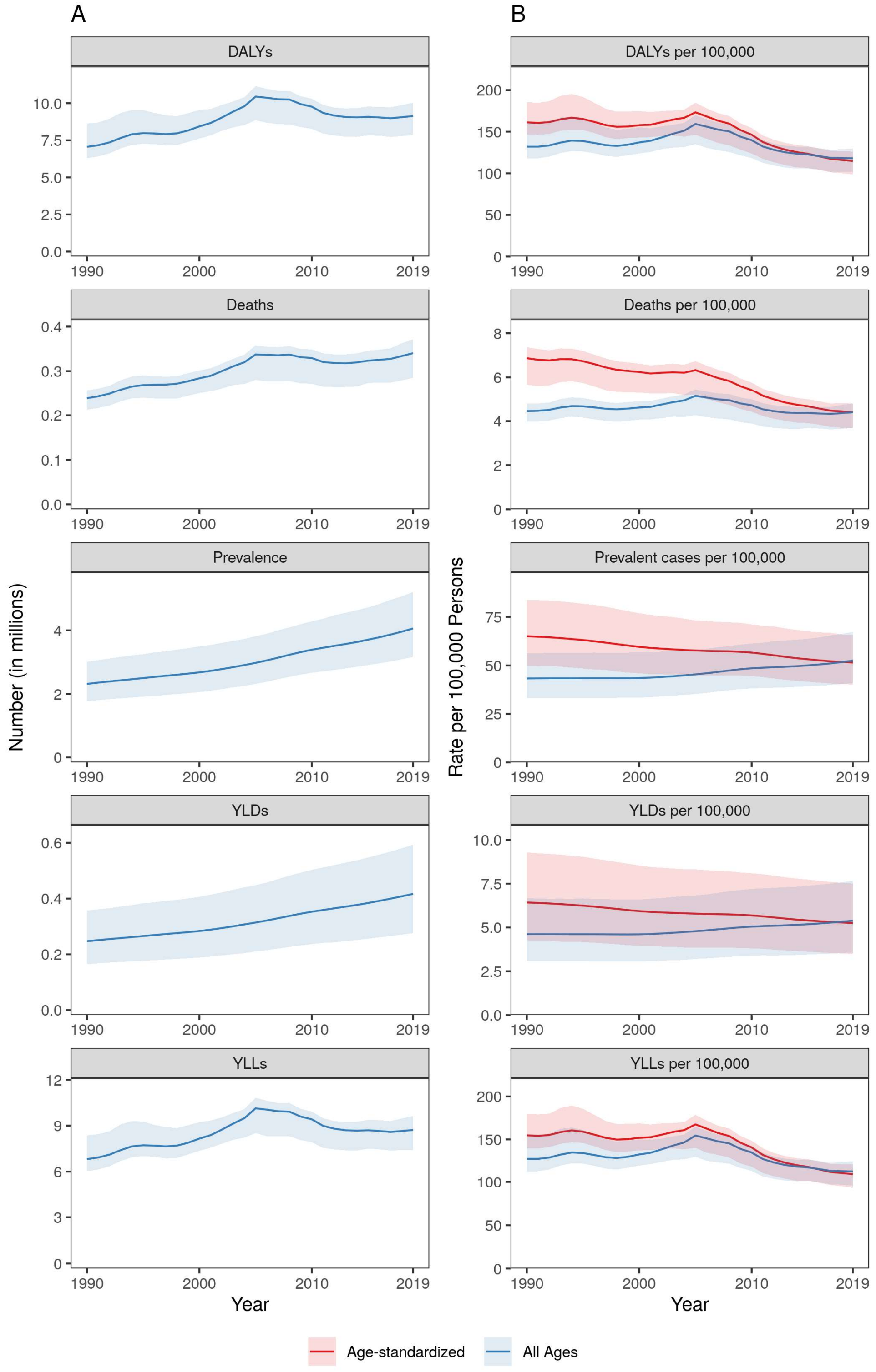

A. Total number of disability-adjusted life years (DALYs), deaths, prevalent cases, years lived with disability (YLDs), and years of life lost (YLLs) due to cardiomyopathy and myocarditis,

1990 to 2019. Shaded regions represent 95% uncertainty intervals. B. Age-standardized and all ages disability-adjusted life years (DALYs), death, prevalence, years lived with disability (YLDs), and years of life lost (YLLs) rates of cardiomyopathy and myocarditis, 1990 to 2019. Shaded regions represent 95% uncertainty intervals.

Supplementary Figure 20. Age-Standardized DALYs due to Other Cardiomyopathy in 2019 by Region

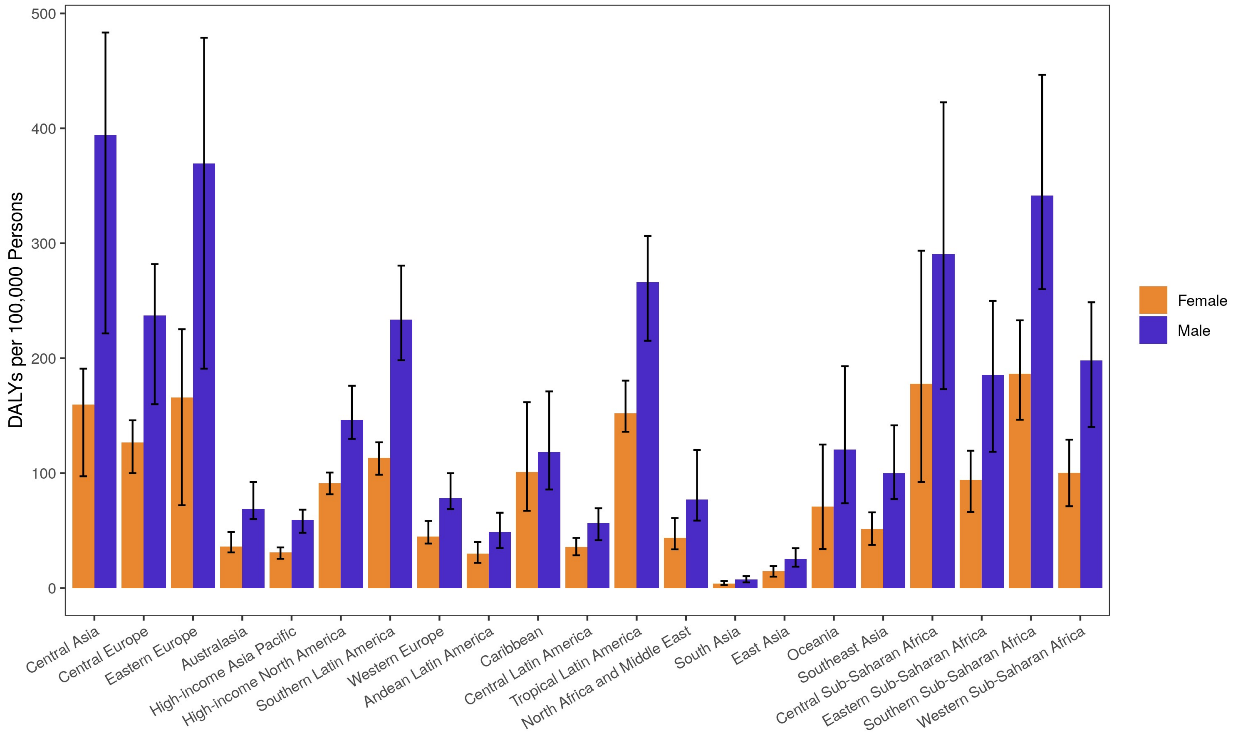

Age-standardized disability-adjusted life years (DALYs) rate of other cardiomyopathy by region and sex with 95% uncertainty intervals, 2019.

Supplementary Figure 21. Total Numbers and Rates of Alcoholic Cardiomyopathy

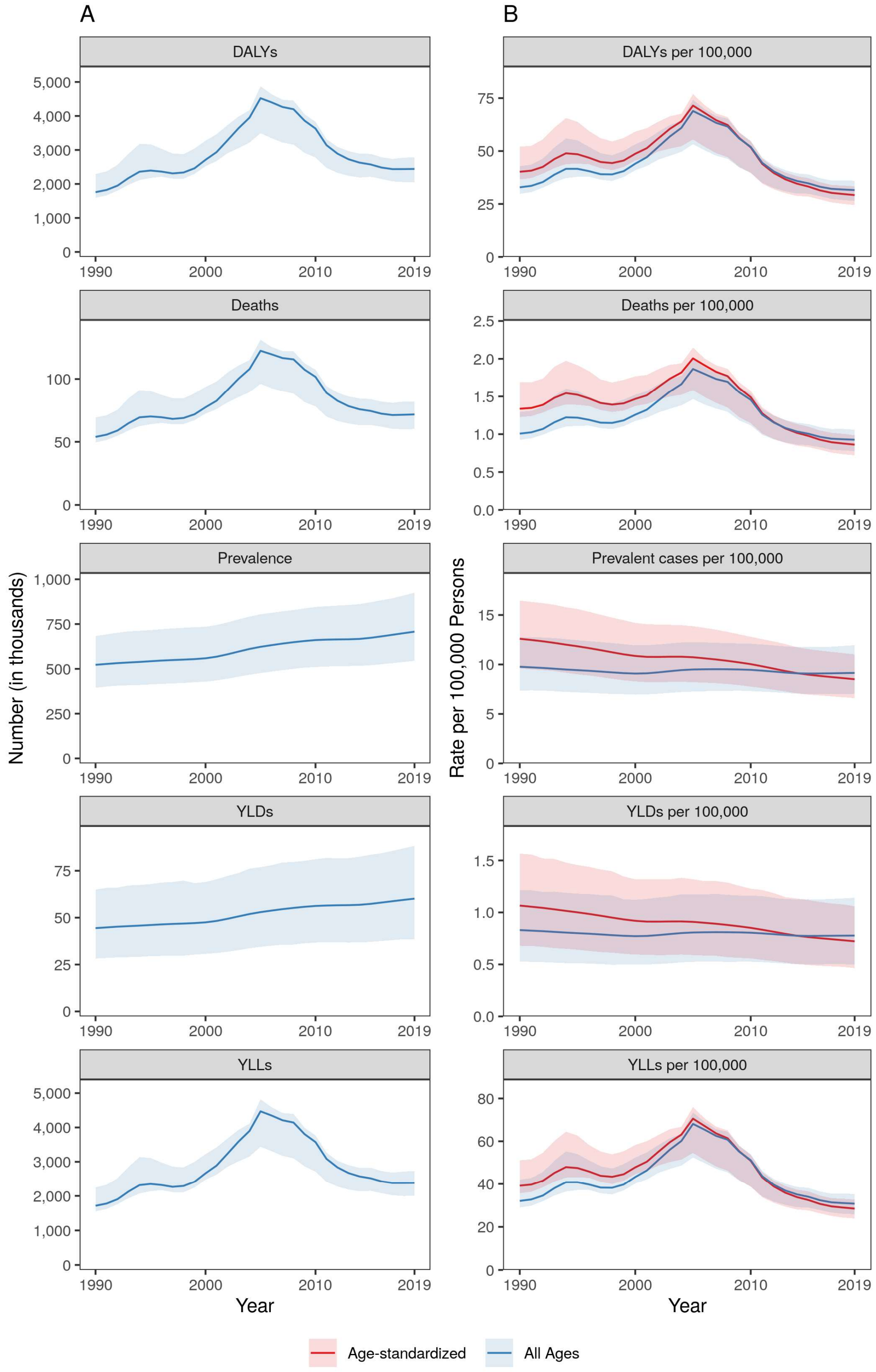

A. Total number of disability-adjusted life years (DALYs), deaths, prevalent cases, years lived with disability (YLDs), and years of life lost (YLLs) due to alcoholic cardiomyopathy, 1990 to

2019. Shaded regions represent 95% uncertainty intervals. B. Age-standardized and all ages disability-adjusted life years (DALYs), death, prevalence, years lived with disability (YLDs), and years of life lost (YLLs) rates of alcoholic cardiomyopathy, 1990 to 2019. Shaded regions represent 95% uncertainty intervals.

Supplementary Figure 22. DALYs due to Alcoholic Cardiomyopathy in 2019 by Age

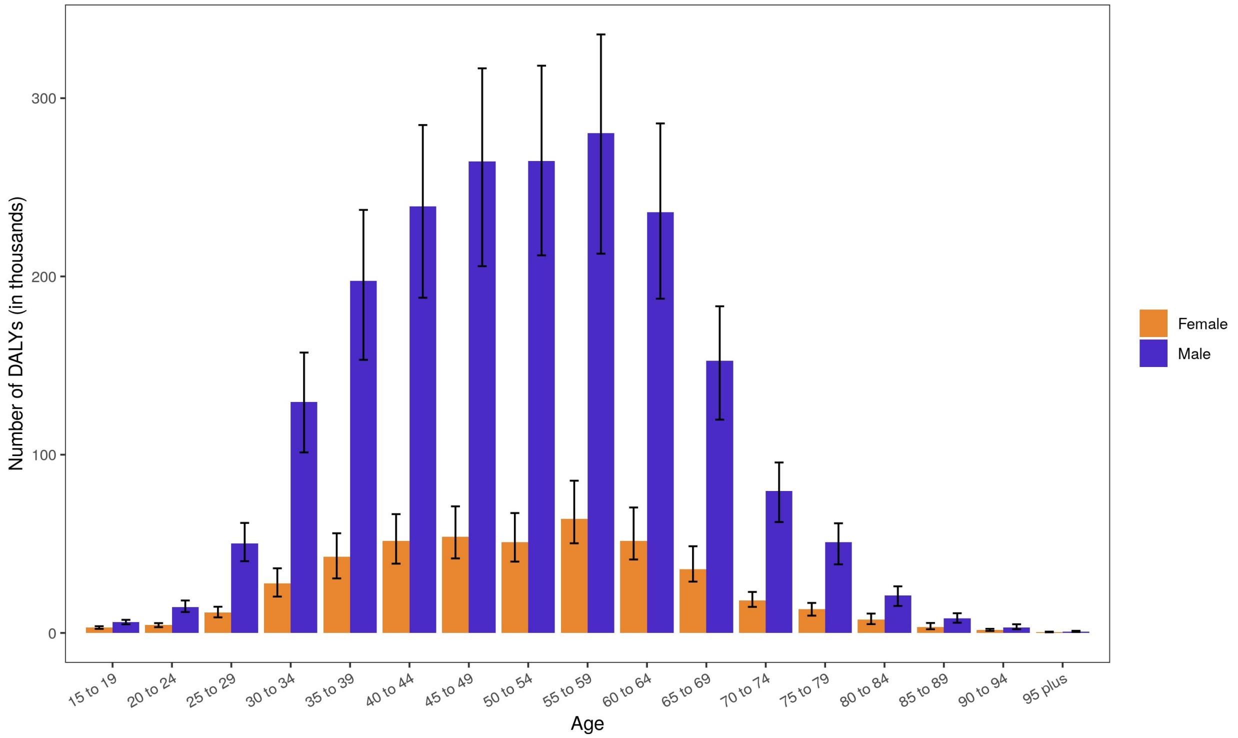

Number of disability-adjusted life years (DALYs) due to alcoholic cardiomyopathy by age and sex with 95% uncertainty intervals, 2019. Ages below 15 were removed from the figure as they are not modeled for this cause.

Supplementary Figure 23. Total Numbers and Rates of Atrial Fibrillation and Flutter

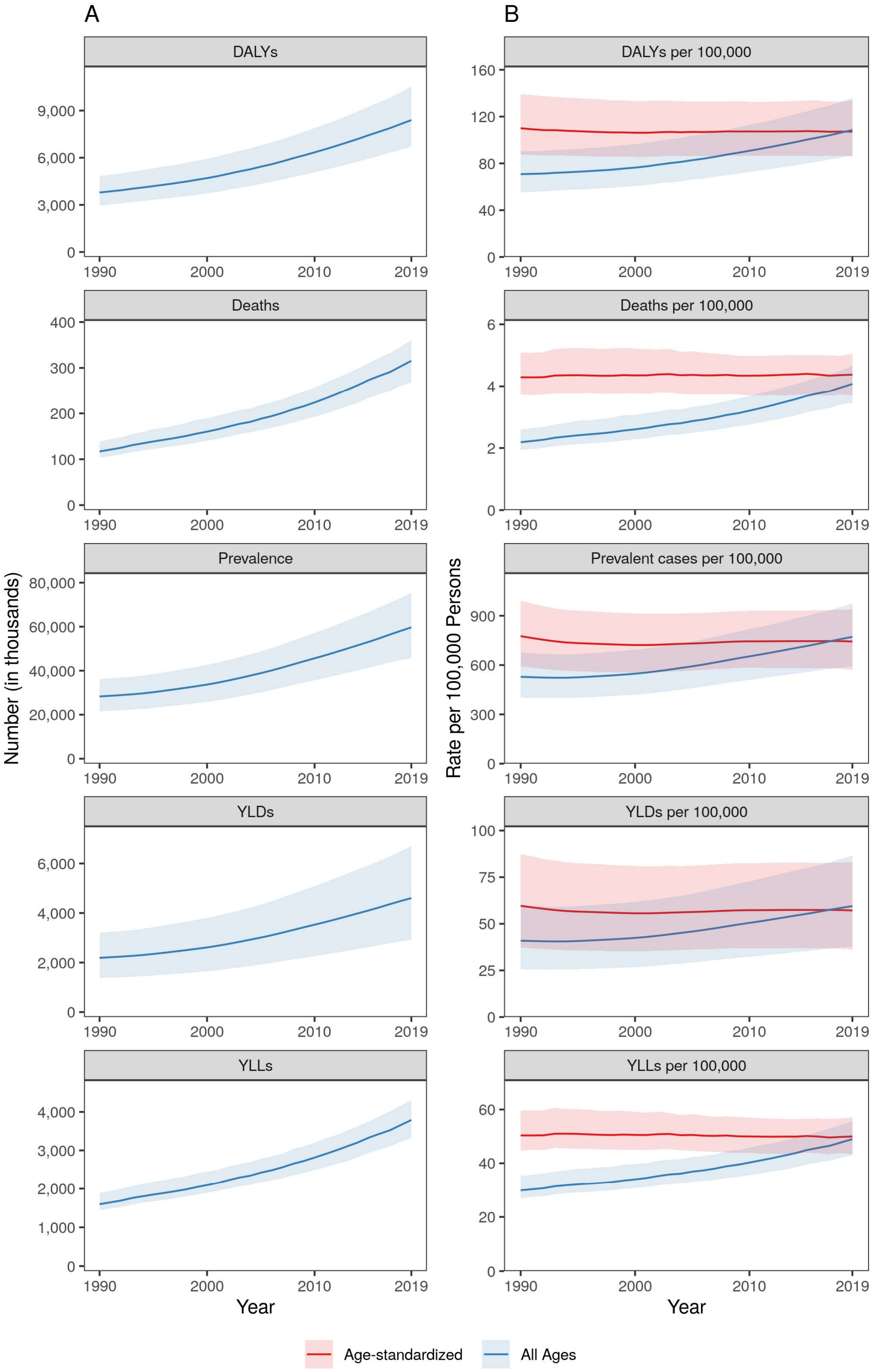

A. Total number of disability-adjusted life years (DALYs), deaths, prevalent cases, years lived with disability (YLDs), and years of life lost (YLLs) due to atrial fibrillation and flutter, 1990 to

2019. Shaded regions represent 95% uncertainty intervals. B. Age-standardized and all ages disability-adjusted life years (DALYs), death, prevalence, years lived with disability (YLDs), and years of life lost (YLLs) rates of atrial fibrillation and flutter, 1990 to 2019. Shaded regions represent 95% uncertainty intervals.

Supplementary Figure 24. DALYs due to Atrial Fibrillation and Flutter in 2019 by Age

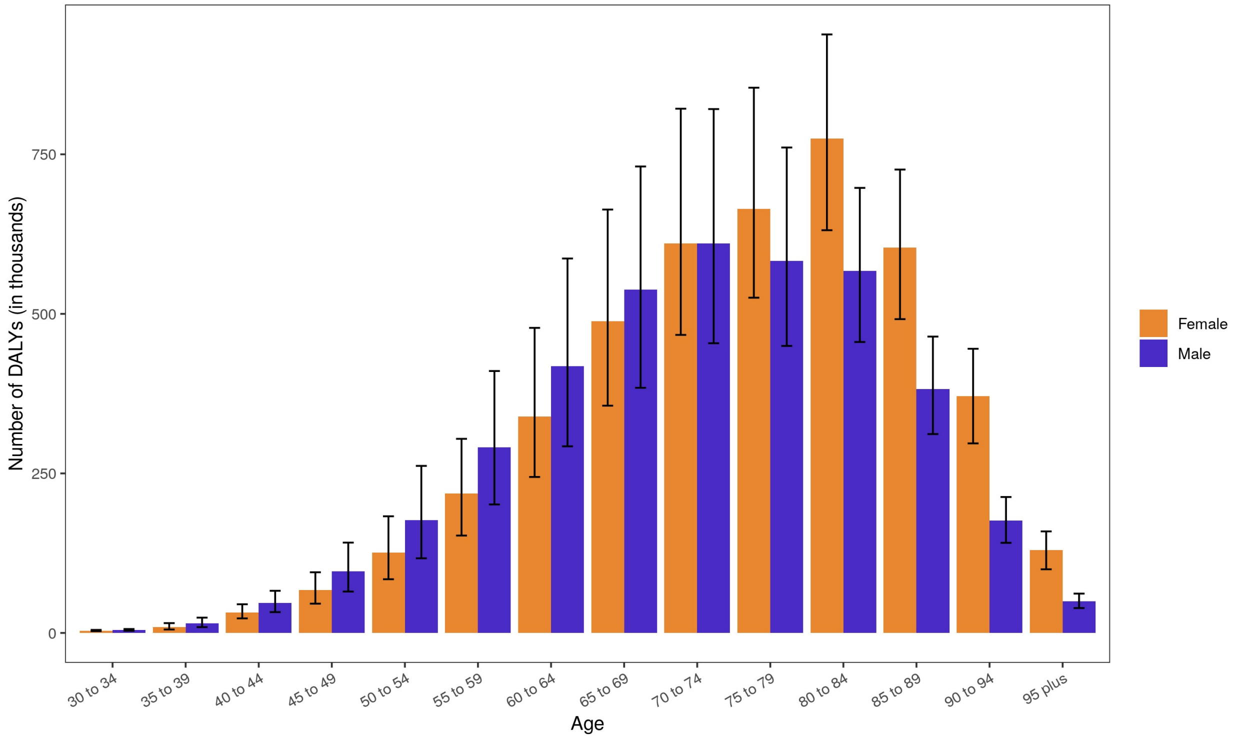

Number of disability-adjusted life years (DALYs) due to atrial fibrillation and flutter by age and sex with 95% uncertainty intervals, 2019. Ages below 30 were removed from the figure as they are not modeled for this cause.

Supplementary Figure 25. YLLs due to Aortic Aneurysm in 2019 by Age

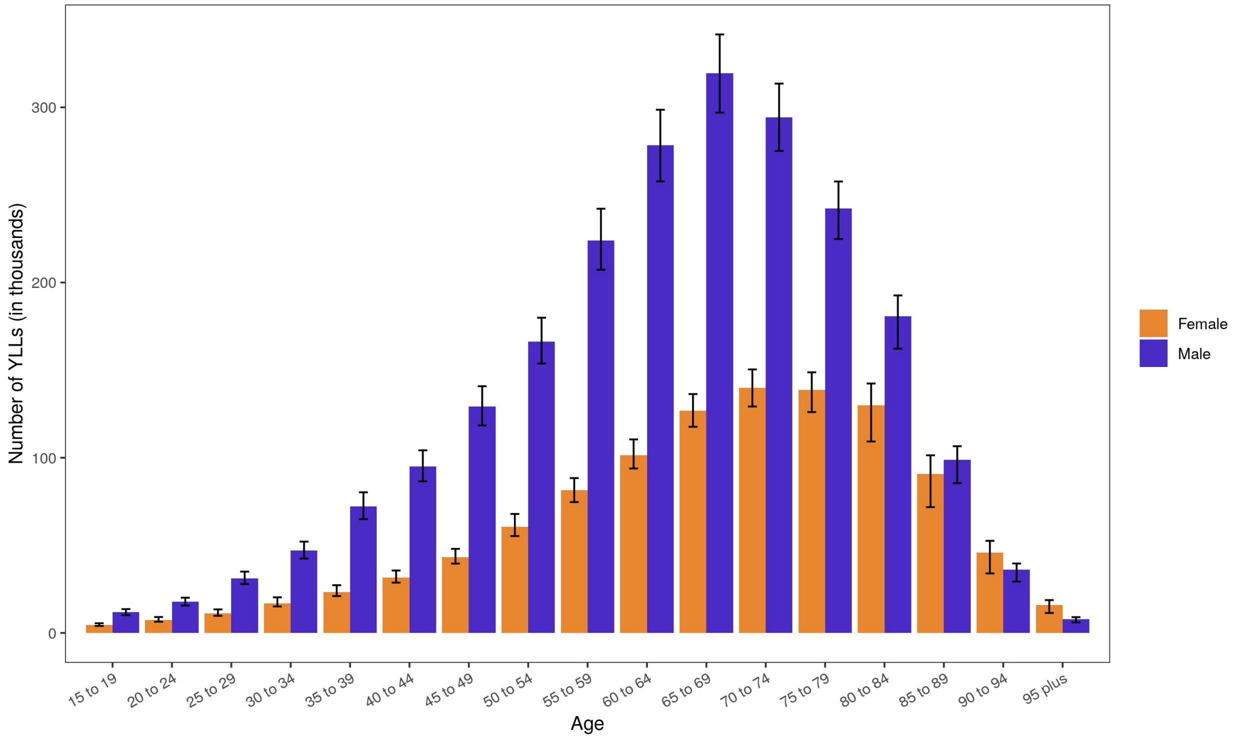

Number of years of life lost (YLLs) due to aortic aneurysm by age and sex with 95% uncertainty intervals, 2019. Ages below 15 were removed from the figure as they are not modeled for this cause.

Supplementary Figure 26. Map of Age-Standardized YLLs due to Aortic Aneurysm in 2019

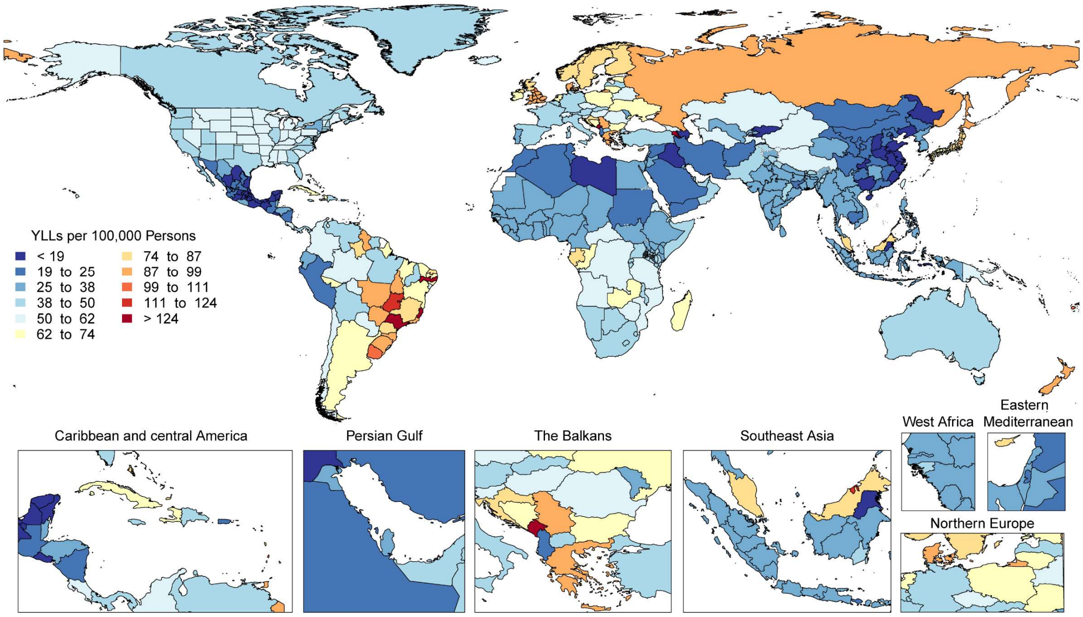

Map of age-standardized years of life lost (YLLs) rate of aortic aneurysm, 2019.

Supplementary Figure 27. Total Numbers and Rates of Non-Rheumatic Calcific Aortic Valve Disease

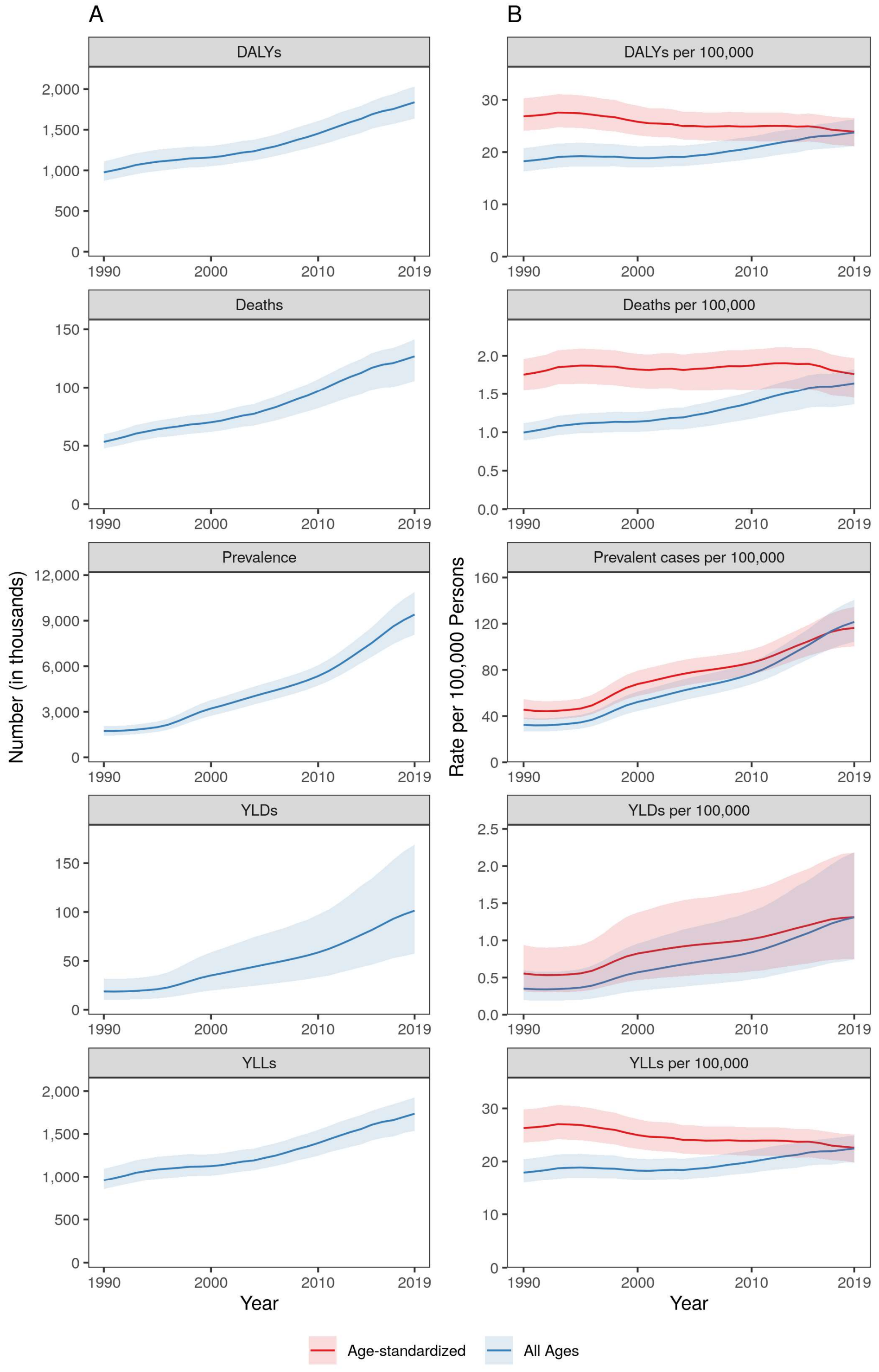

A. Total number of disability-adjusted life years (DALYs), deaths, prevalent cases, years lived with disability (YLDs), and years of life lost (YLLs) due to non-rheumatic calcific aortic valve

disease, 1990 to 2019. Shaded regions represent 95% uncertainty intervals. B. Age-standardized and all ages disability-adjusted life years (DALYs), death, prevalence, years lived with disability (YLDs), and years of life lost (YLLs) rates of non-rheumatic calcific aortic valve disease, 1990 to 2019. Shaded regions represent 95% uncertainty intervals.

Supplementary Figure 28. Total Numbers and Rates of Non-Rheumatic Degenerative Mitral Valve Disease

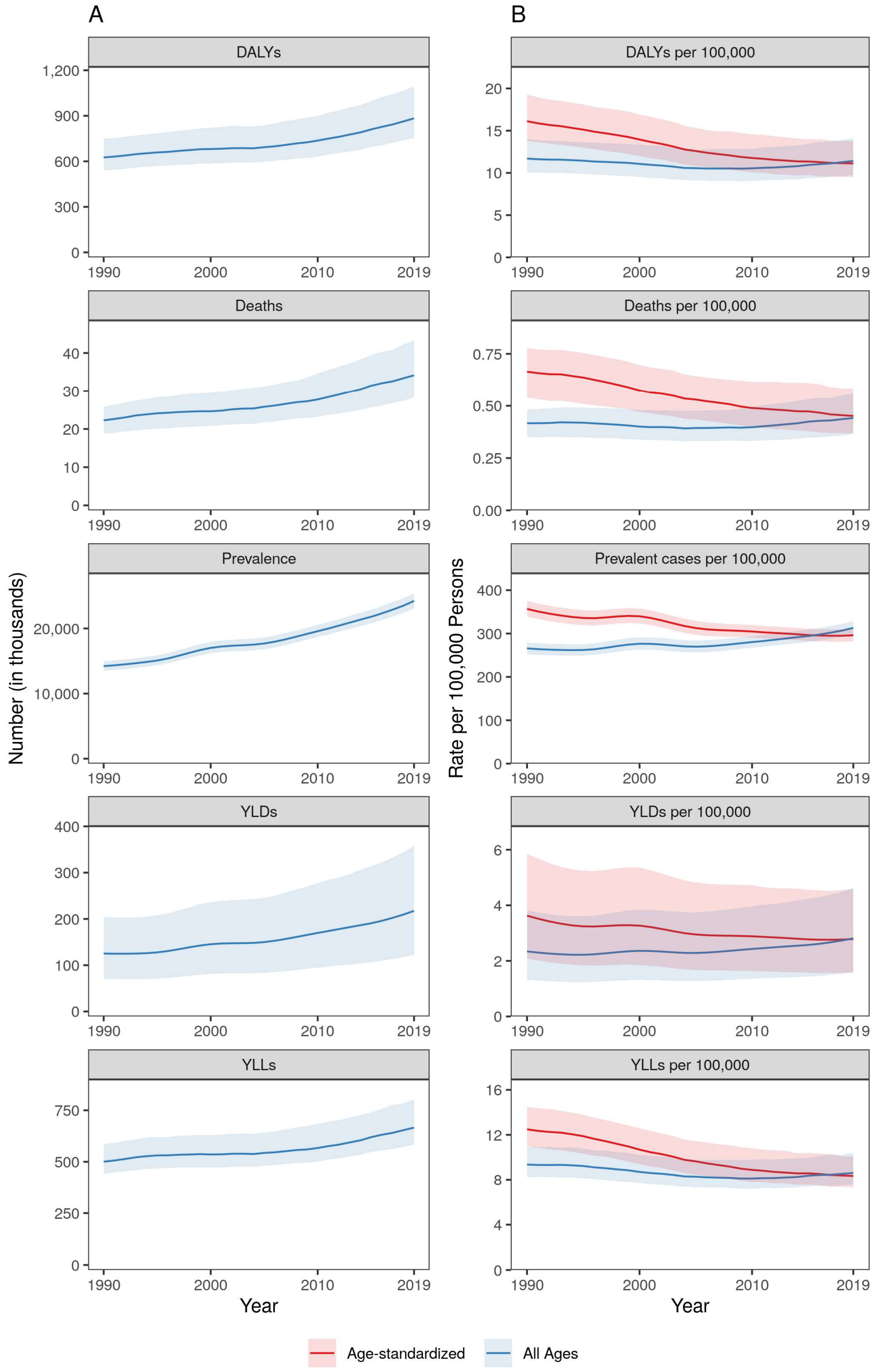

A. Total number of disability-adjusted life years (DALYs), deaths, prevalent cases, years lived with disability (YLDs), and years of life lost (YLLs) due to non-rheumatic degenerative mitral

valve disease, 1990 to 2019. Shaded regions represent 95% uncertainty intervals. B. Age-standardized and all ages disability-adjusted life years (DALYs), death, prevalence, years lived with disability (YLDs), and years of life lost (YLLs) rates of non-rheumatic degenerative mitral valve disease, 1990 to 2019. Shaded regions represent 95% uncertainty intervals.

Supplementary Figure 29. Map of Age-Standardized DALYs due to Non-Rheumatic Degenerative Mitral Valve Disease in 2019

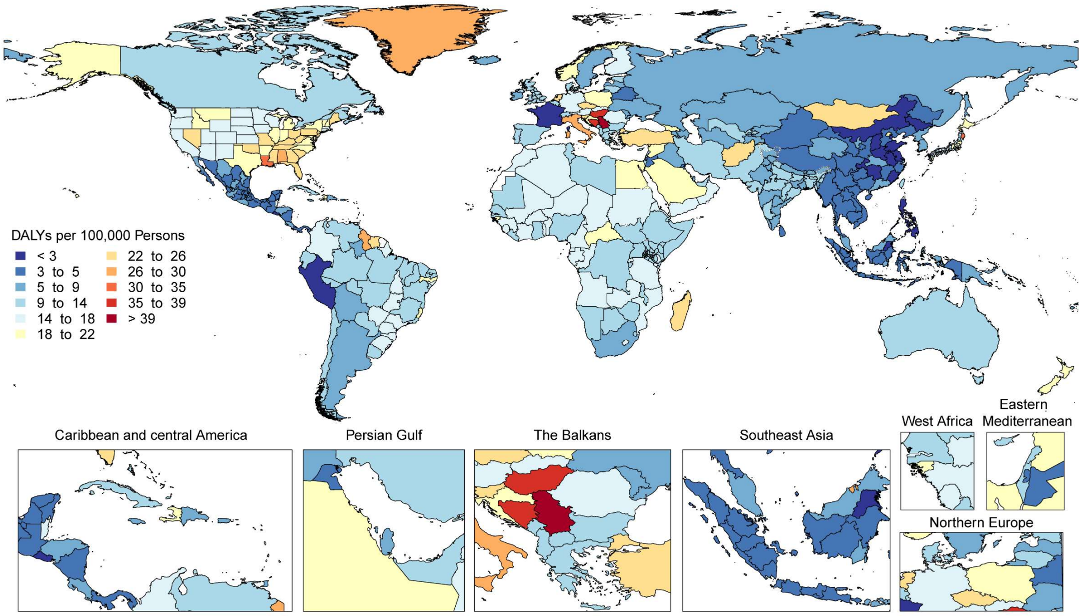

Map of age-standardized disability-adjusted life years (DALYs) rate of non-rheumatic degenerative mitral valve disease, 2019.

Supplementary Figure 30. Age-Standardized DALYs due to Non-Rheumatic Degenerative Mitral Valve Disease in 2019 by Region

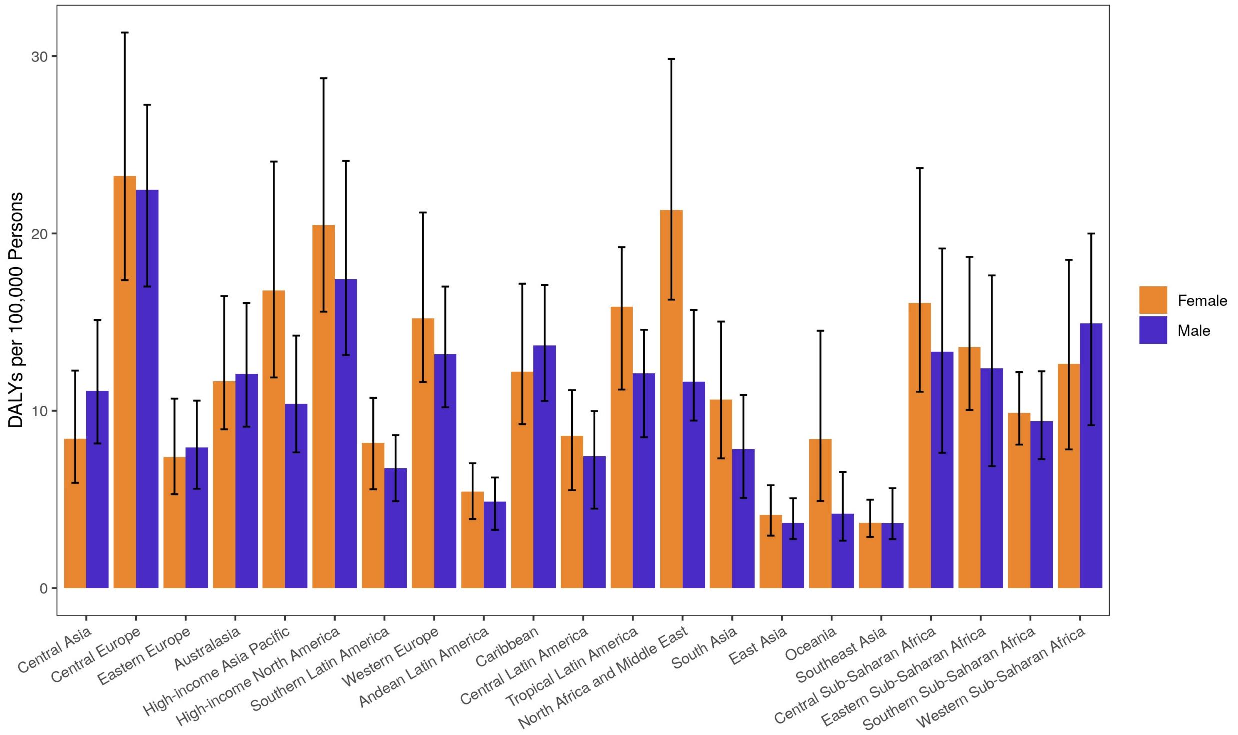

Age-standardized disability-adjusted life years (DALYs) rate of non-rheumatic degenerative mitral valve disease by region and sex with 95% uncertainty intervals, 2019.

Supplementary Figure 31. DALYs due to Endocarditis in 2019 by Age

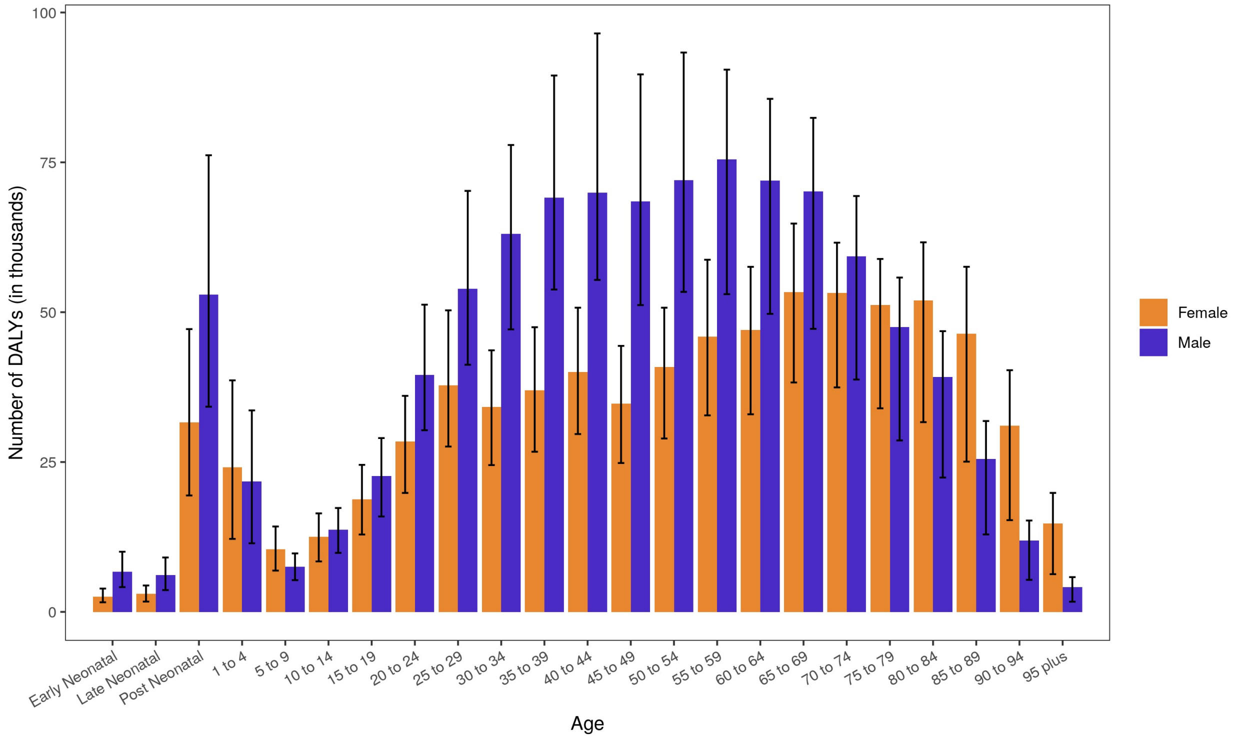

Number of disability-adjusted life years (DALYs) due to endocarditis by age and sex with 95% uncertainty intervals, 2019.

Supplementary Figure 32. Age-Standardized DALYs due to Endocarditis in 2019 by Region

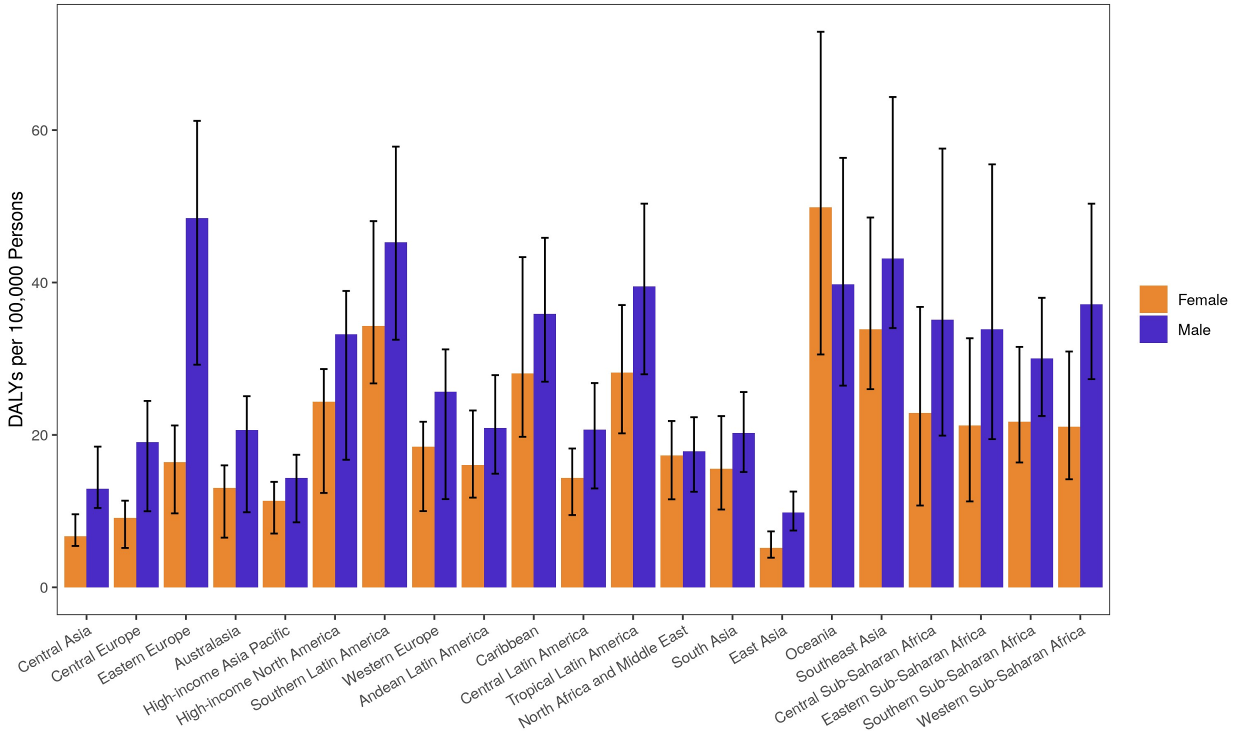

Age-standardized disability-adjusted life years (DALYs) rate of endocarditis by region and sex with 95% uncertainty intervals, 2019.

Supplementary Figure 33. Map of Age-Standardized DALYs due to Endocarditis in 2019

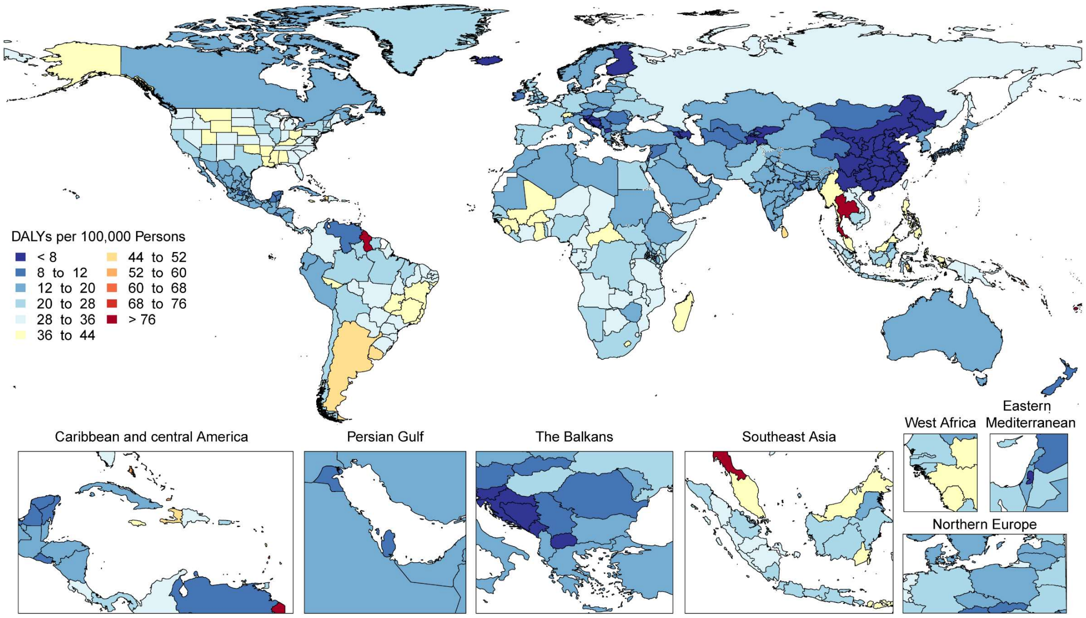

Map of age-standardized disability-adjusted life years (DALYs) rate of endocarditis, 2019.

Supplementary Figure 34. Total Numbers and Rates of Peripheral Artery Disease

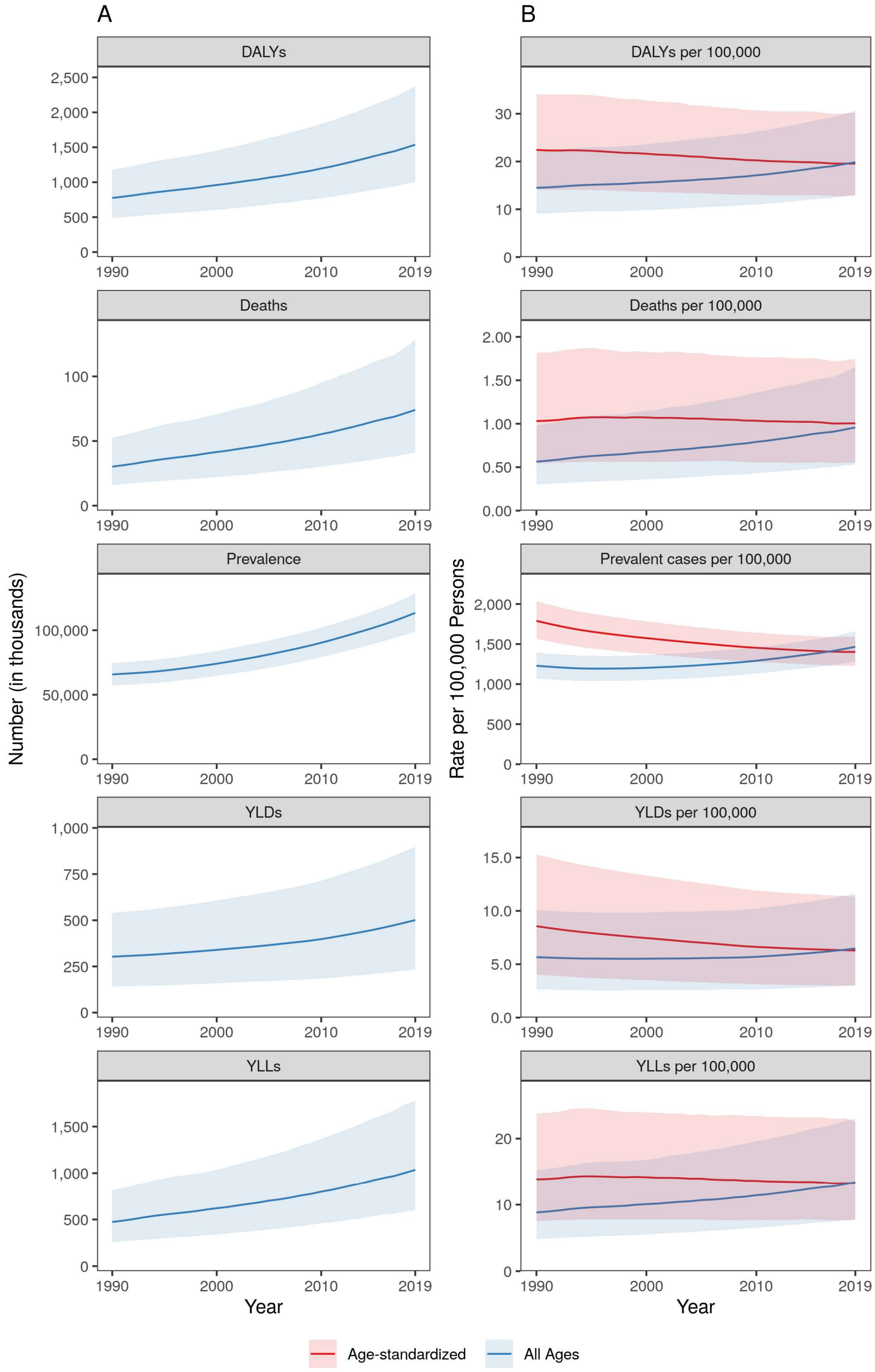

A. Total number of disability-adjusted life years (DALYs), deaths, prevalent cases, years lived with disability (YLDs), and years of life lost (YLLs) due to peripheral artery disease, 1990 to

2019. Shaded regions represent 95% uncertainty intervals. B. Age-standardized and all ages disability-adjusted life years (DALYs), death, prevalence, years lived with disability (YLDs), and years of life lost (YLLs) rates of peripheral artery disease, 1990 to 2019. Shaded regions represent 95% uncertainty intervals.

Supplementary Figure 35. Age-Standardized DALYs due to Peripheral Artery Disease in 2019 by Region

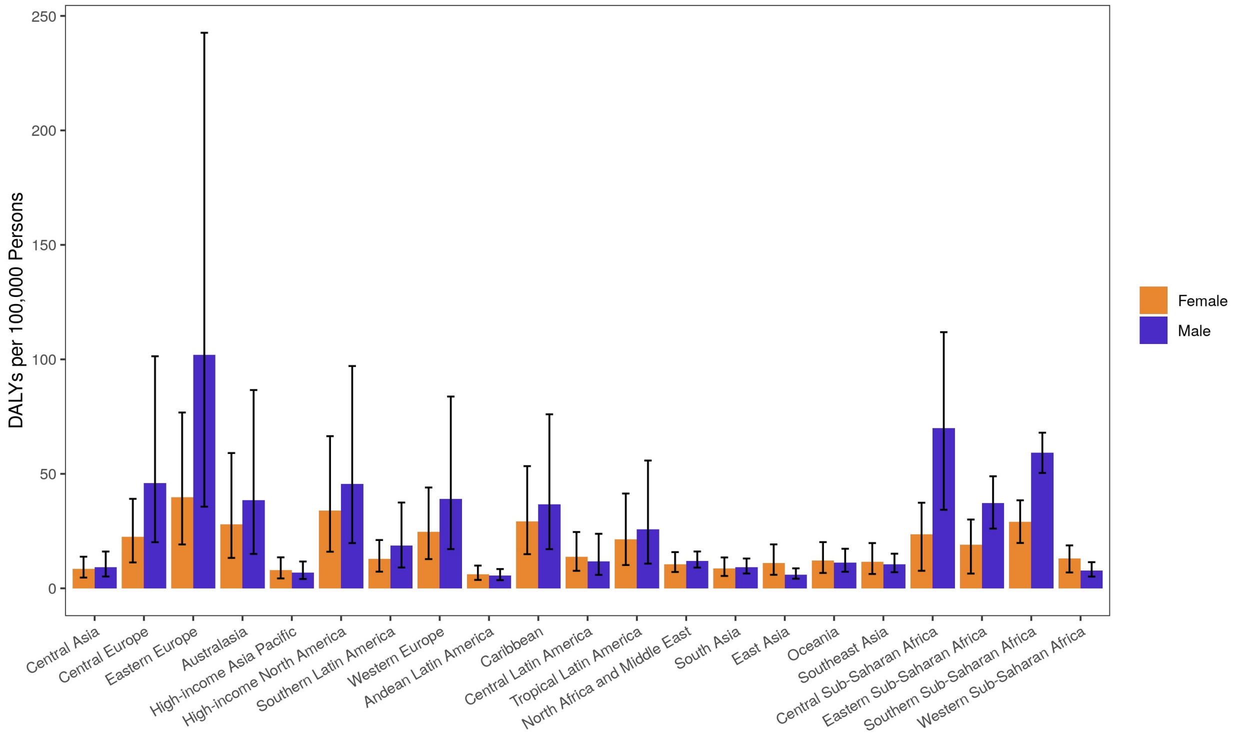

Age-standardized disability-adjusted life years (DALYs) rate of peripheral artery disease by region and sex with 95% uncertainty intervals, 2019.

Supplementary Figure 36. Total Numbers and Rates of High Systolic Blood Pressure

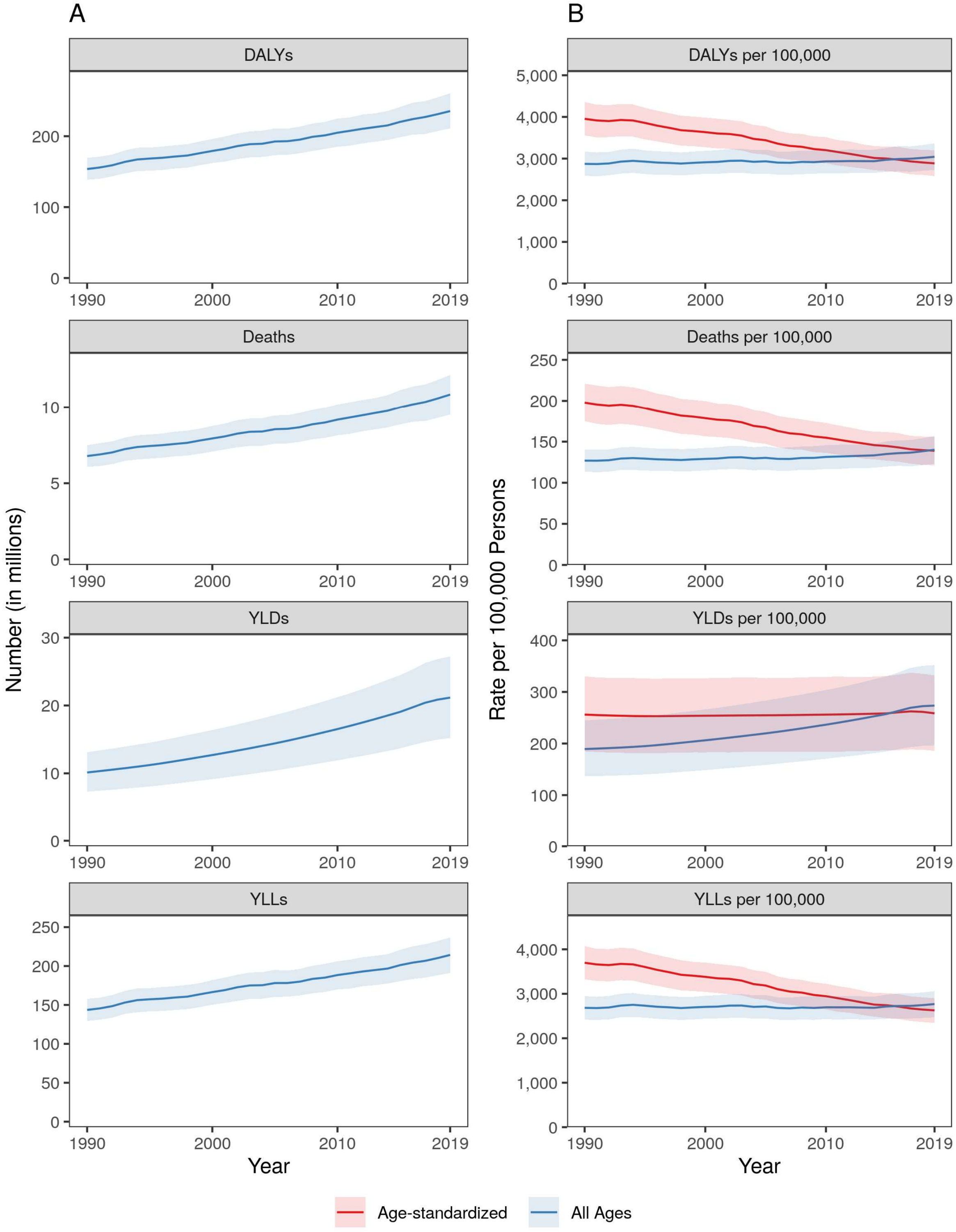

A. Total number of disability-adjusted life years (DALYs), deaths, years lived with disability (YLDs), and years of life lost (YLLs) due to high systolic blood pressure, 1990 to 2019. Shaded

regions represent 95% uncertainty intervals. B. Age-standardized and all ages disability-adjusted life years (DALYs), death, years lived with disability (YLDs), and years of life lost (YLLs) rates of high systolic blood pressure, 1990 to 2019. Shaded regions represent 95% uncertainty intervals.

Supplementary Figure 37. Age-Standardized DALYs due to High Systolic Blood Pressure in 2019 by Region

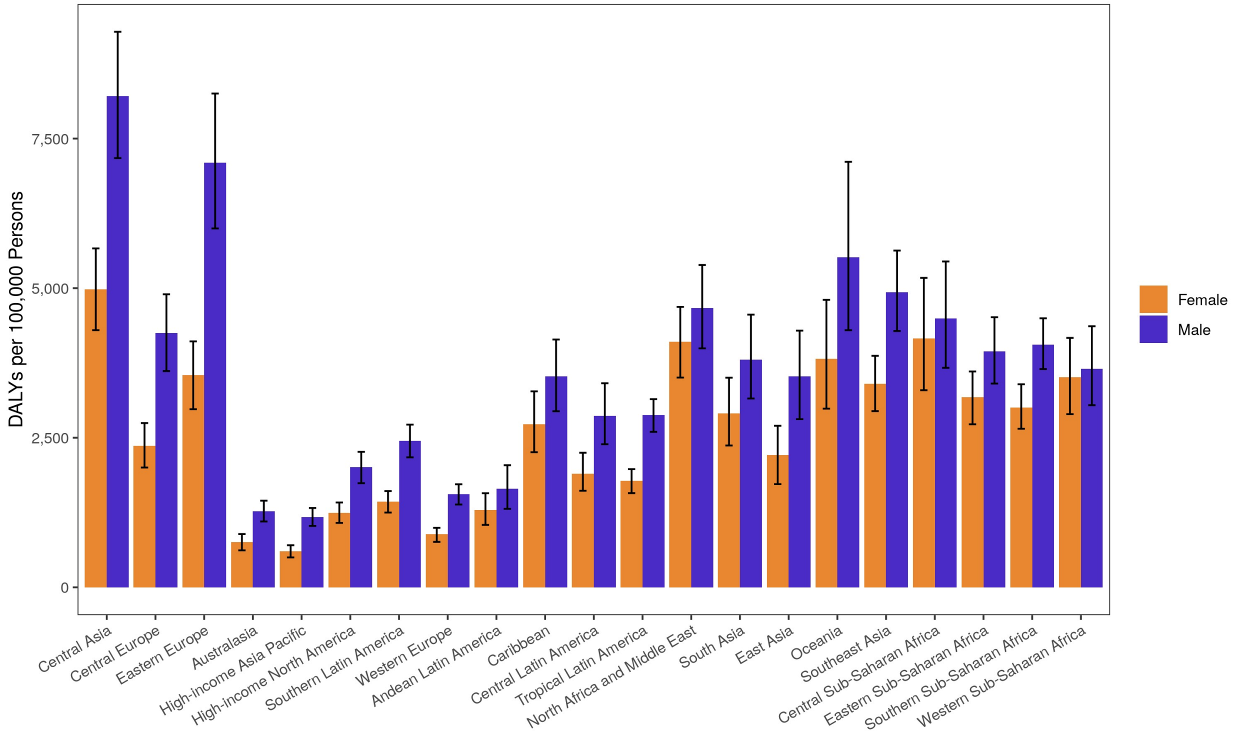

Age-standardized disability-adjusted life years (DALYs) rate of high systolic blood pressure by region and sex with 95% uncertainty intervals, 2019.

Supplementary Figure 38. Map of Age-Standardized DALYs due to High Systolic Blood Pressure in 2019

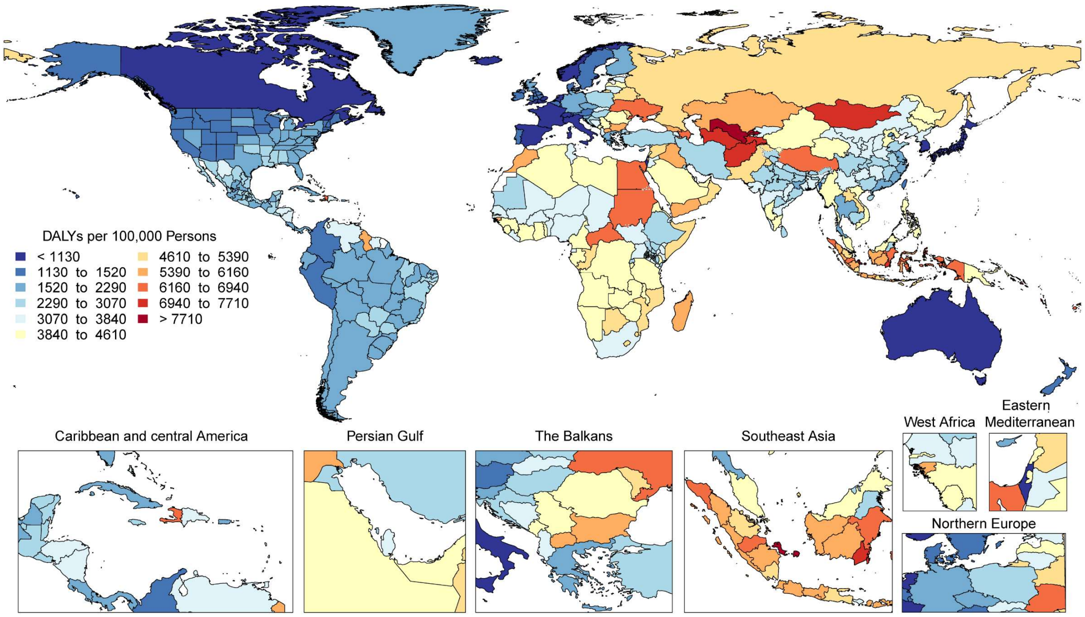

Map of age-standardized disability-adjusted life years (DALYs) rate of high systolic blood pressure, 2019.

Supplementary Figure 39. DALYs due to High Fasting Plasma Glucose in 2019 by Age

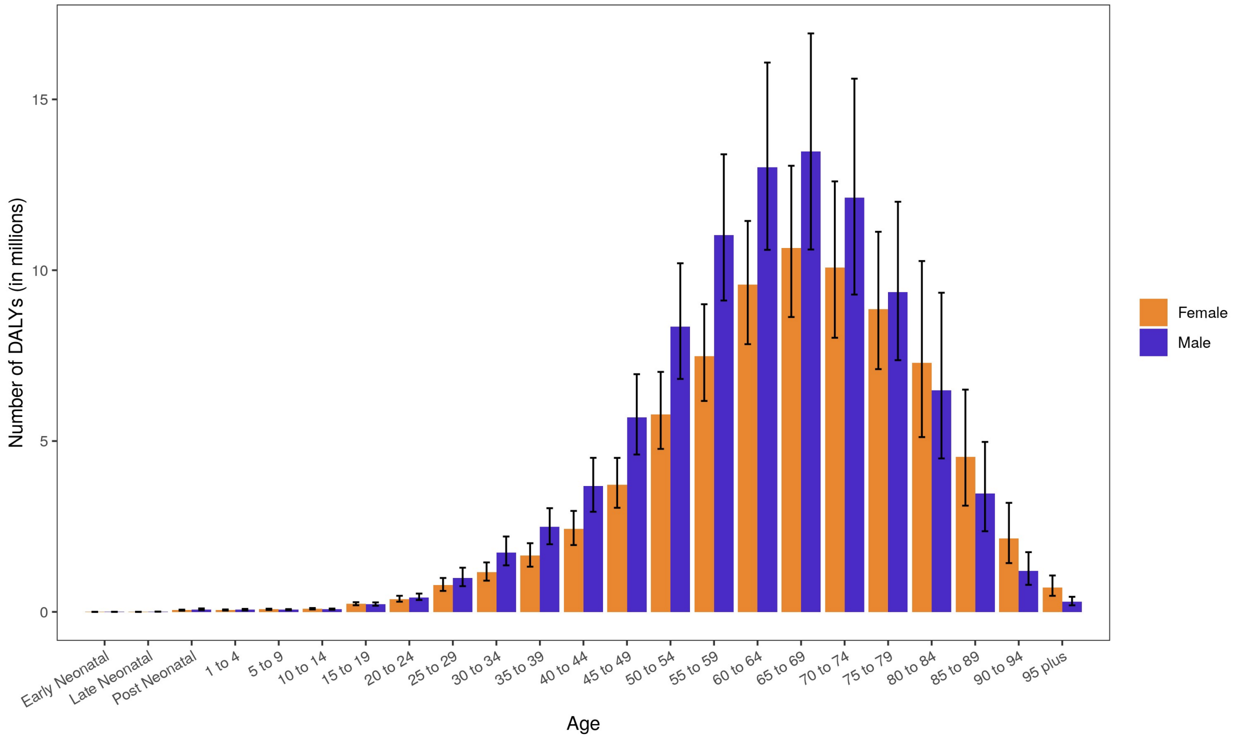

Number of disability-adjusted life years (DALYs) due to high fasting plasma glucose by age and sex with 95% uncertainty intervals, 2019.

Supplementary Figure 40. Age-Standardized DALYs due to High Fasting Plasma Glucose in 2019 by Region

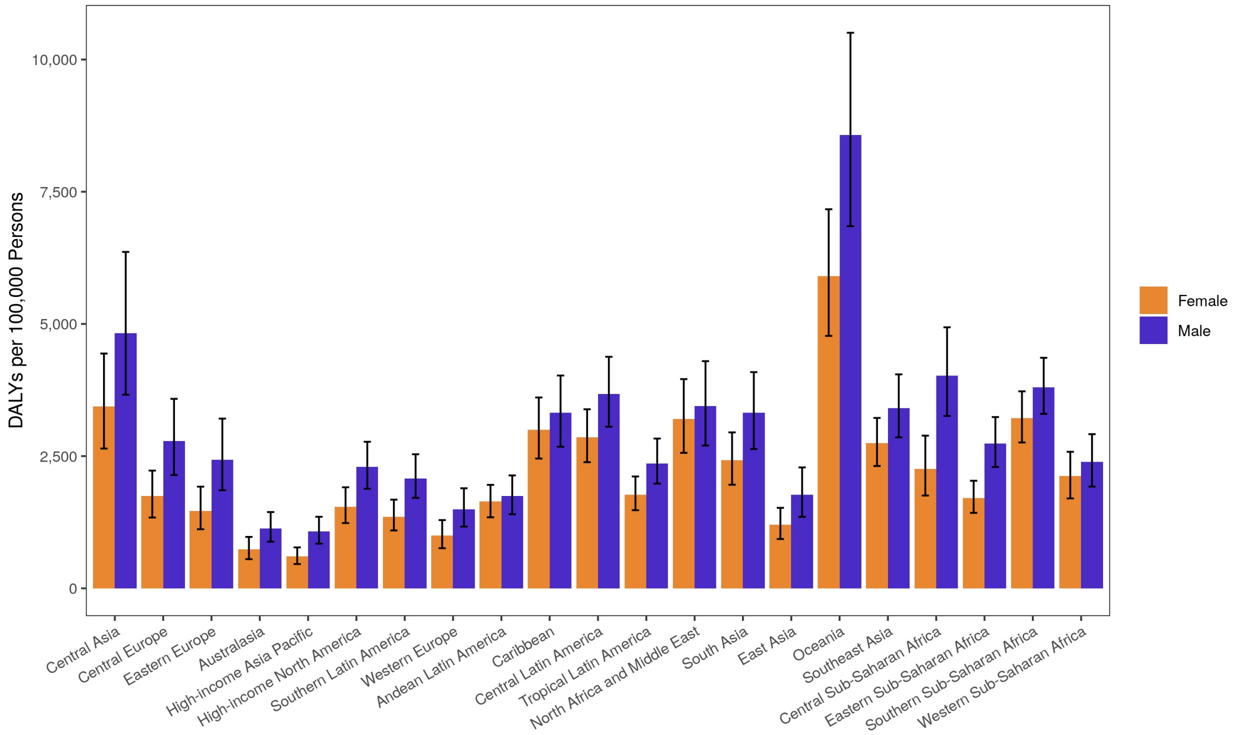

Age-standardized disability-adjusted life years (DALYs) rate of high fasting plasma glucose by region and sex with 95% uncertainty intervals, 2019.

Supplementary Figure 41. Map of Age-Standardized DALYs due to High Fasting Plasma Glucose in 2019

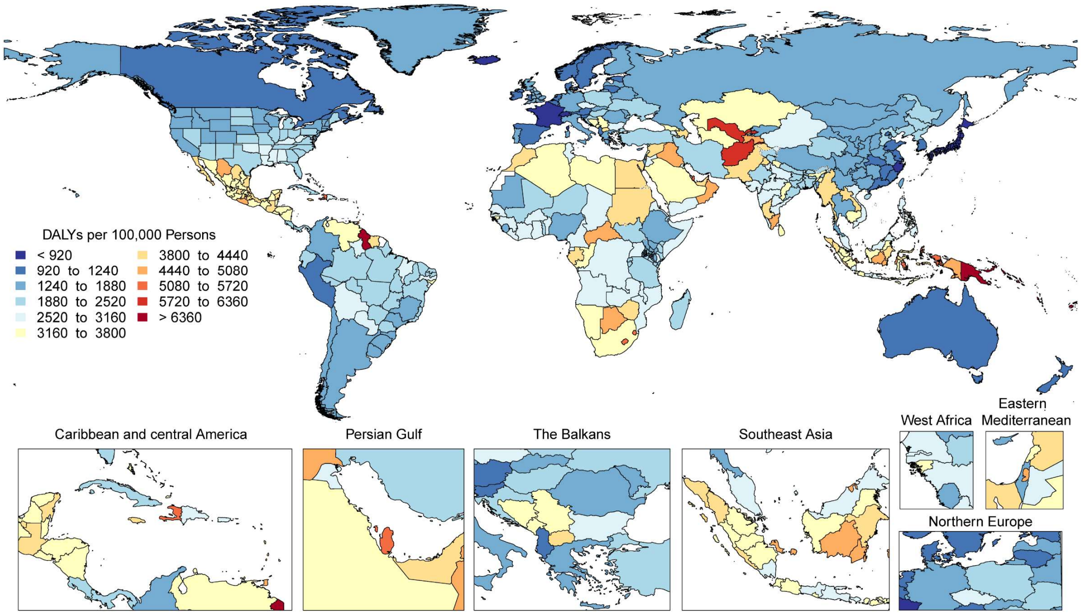

Map of age-standardized disability-adjusted life years (DALYs) rate of high fasting plasma glucose, 2019.

Supplementary Figure 42. Total Numbers and Rates of High LDL Cholesterol

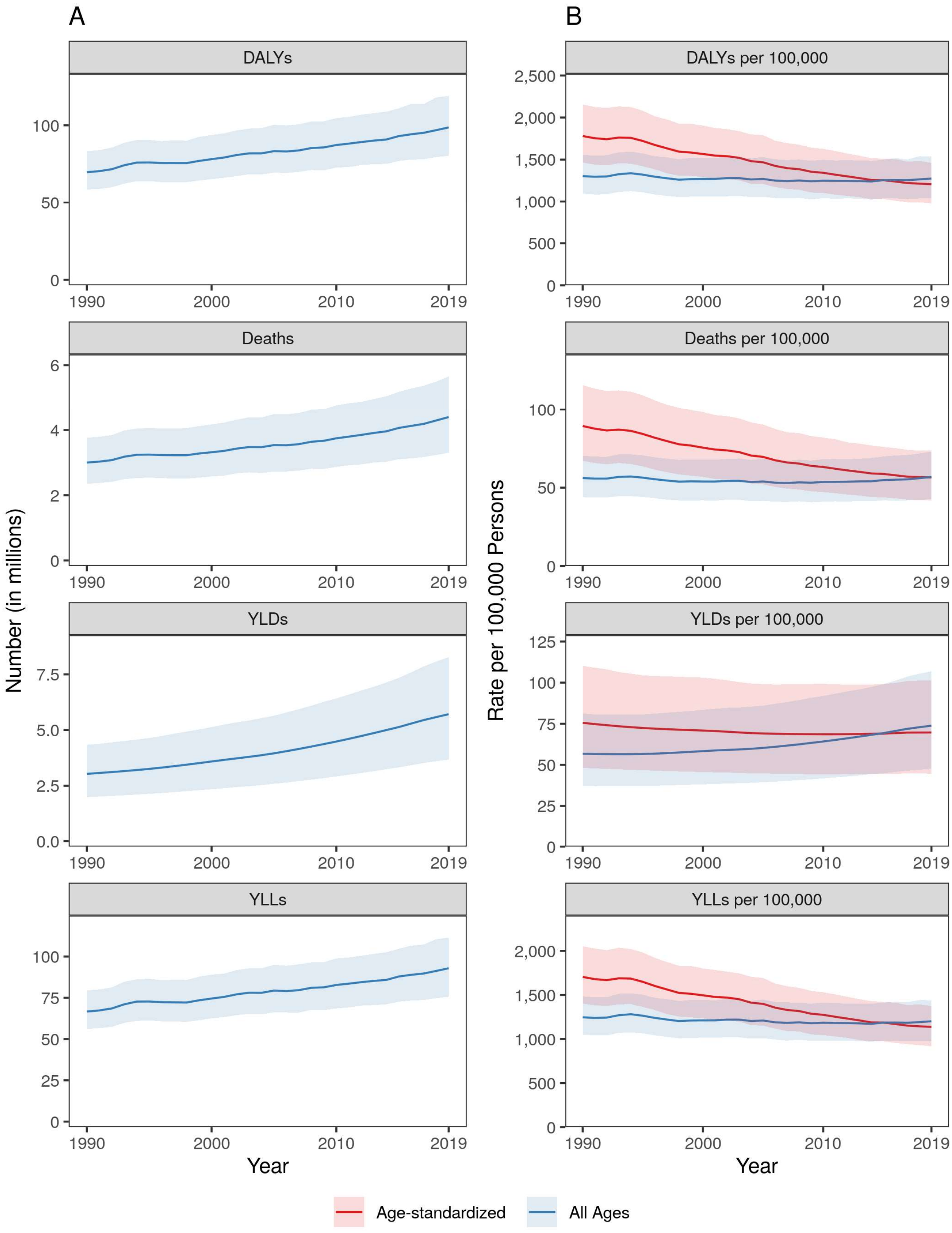

A. Total number of disability-adjusted life years (DALYs), deaths, years lived with disability (YLDs), and years of life lost (YLLs) due to high LDL cholesterol, 1990 to 2019. Shaded

regions represent 95% uncertainty intervals. B. Age-standardized and all ages disability-adjusted life years (DALYs), death, years lived with disability (YLDs), and years of life lost (YLLs) rates of high LDL cholesterol, 1990 to 2019. Shaded regions represent 95% uncertainty intervals.

Supplementary Figure 43. DALYs due to High LDL Cholesterol in 2019 by Age

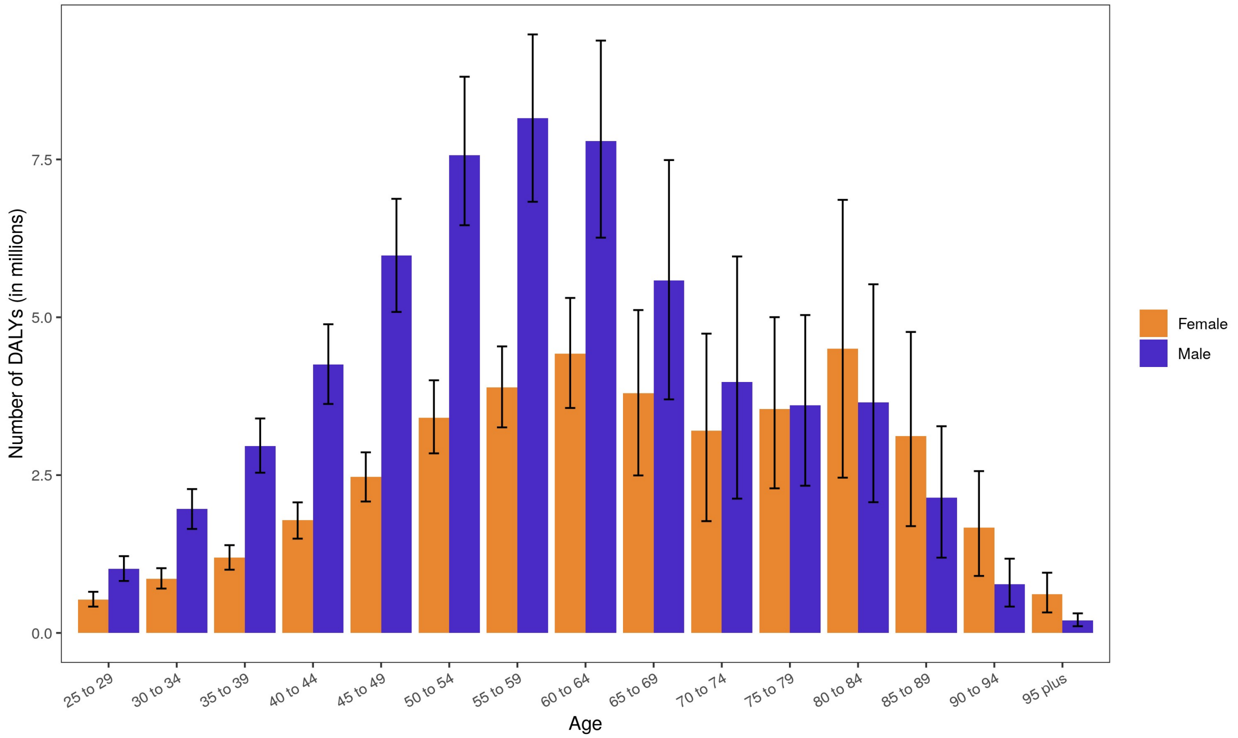

Number of disability-adjusted life years (DALYs) due to high LDL cholesterol by age and sex with 95% uncertainty intervals, 2019. Ages below 25 were removed from the figure as they are not modeled for this risk.

Supplementary Figure 44. Age-Standardized DALYs due to High LDL Cholesterol in 2019 by Region

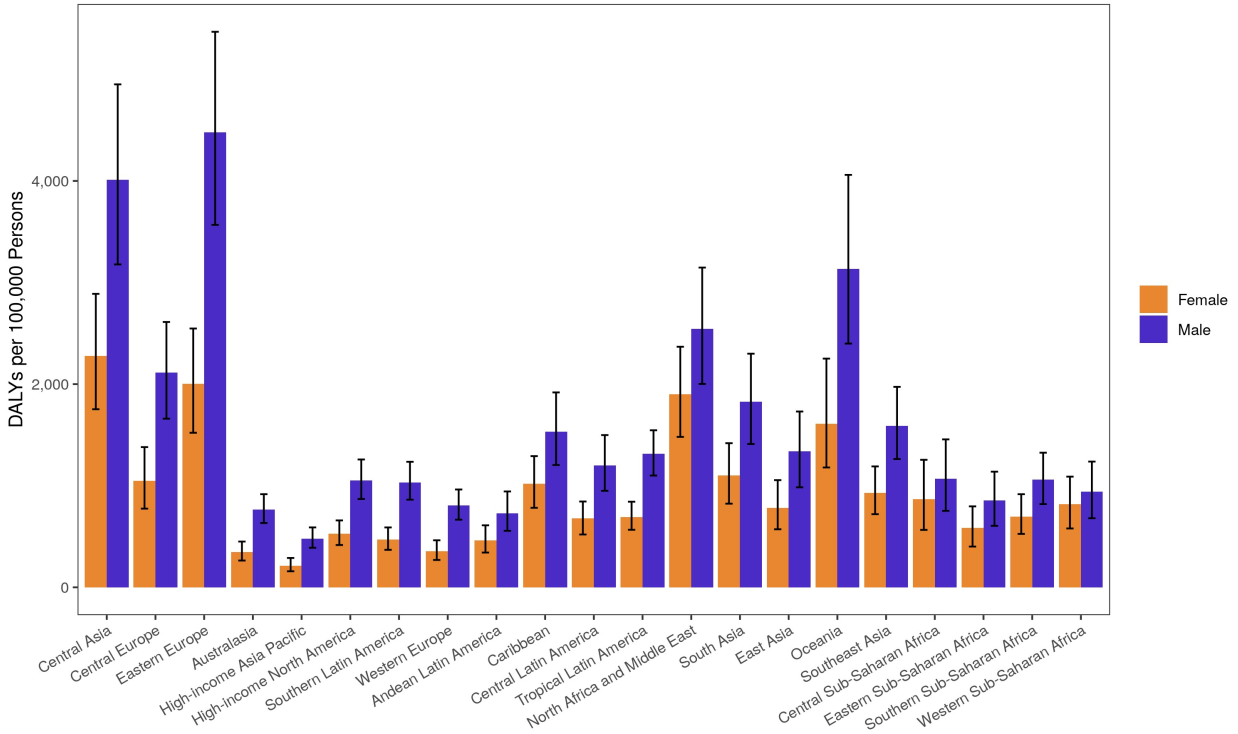

Age-standardized disability-adjusted life years (DALYs) rate of high LDL cholesterol by region and sex with 95% uncertainty intervals, 2019.

Supplementary Figure 45. DALYs due to High Body-mass Index in 2019 by Age

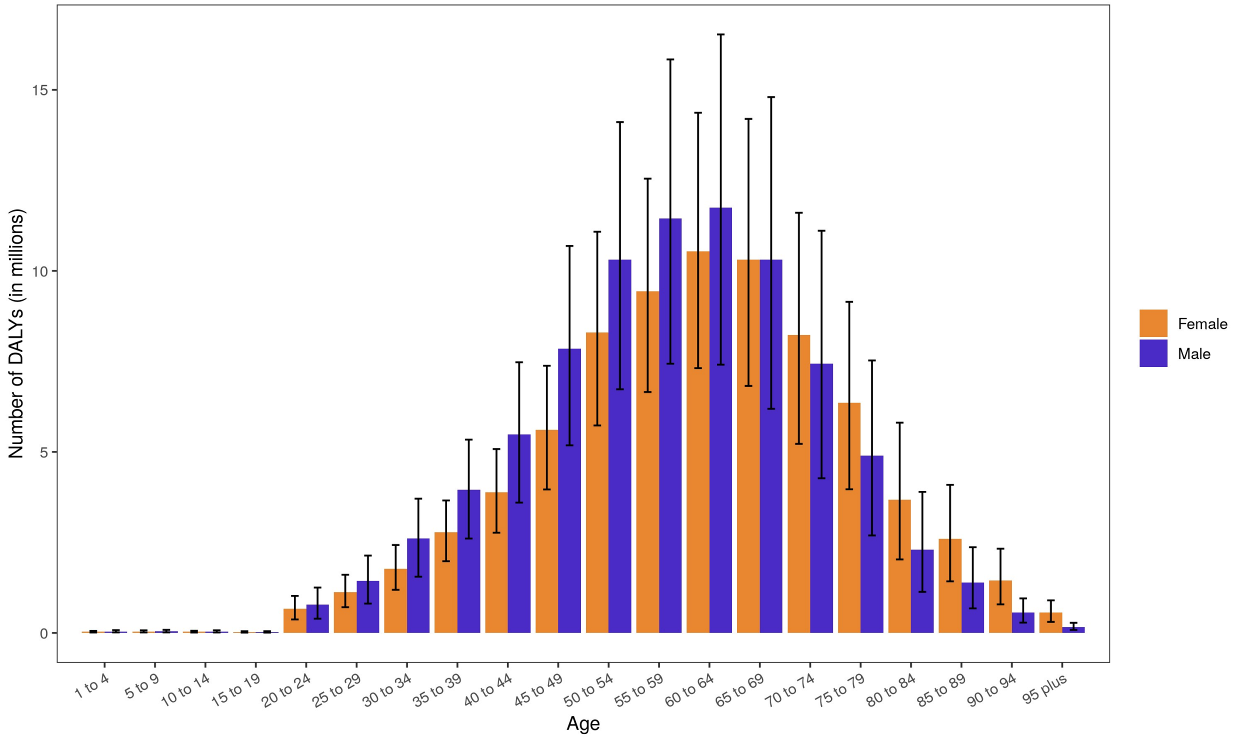

Number of disability-adjusted life years (DALYs) due to high body-mass index by age and sex with 95% uncertainty intervals, 2019. Ages below 1 were removed from the figure as they are not modeled for this risk.

Supplementary Figure 46. Age-Standardized DALYs due to High Body-mass Index in 2019 by Region

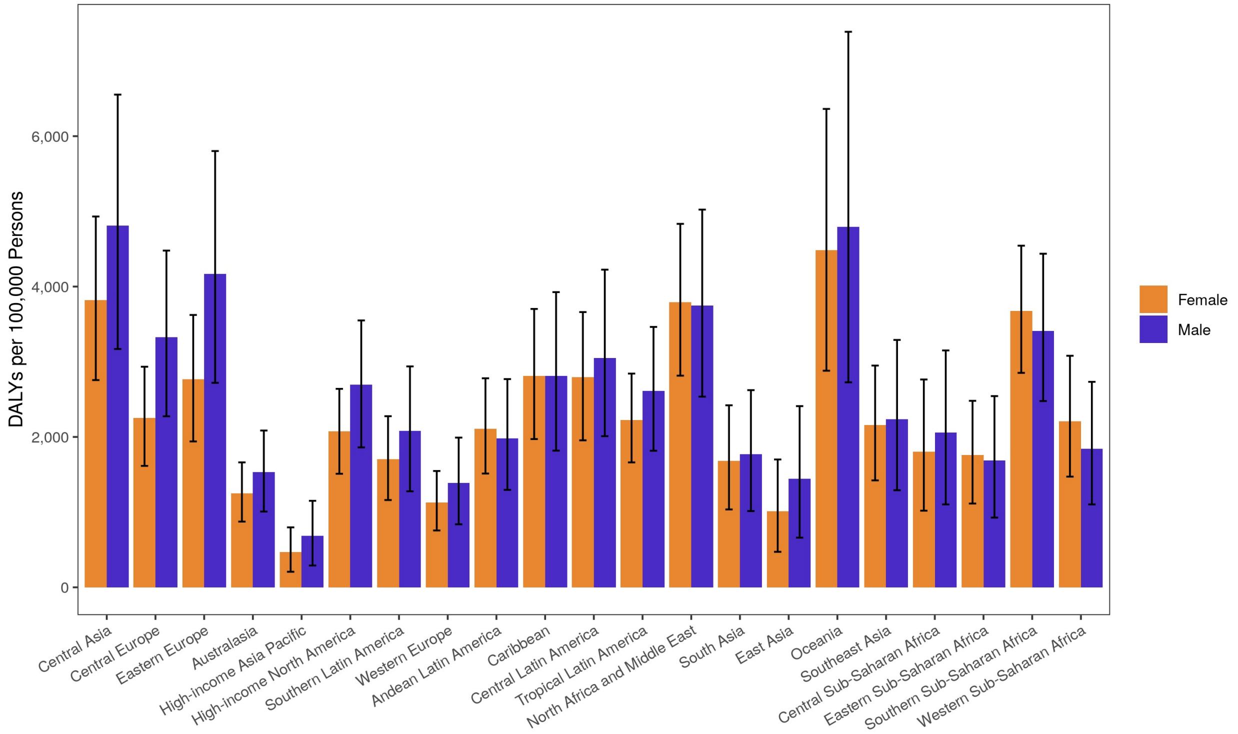

Age-standardized disability-adjusted life years (DALYs) rate of high body-mass index by region and sex with 95% uncertainty intervals, 2019.

Supplementary Figure 47. Map of Age-Standardized DALYs due to High Body-mass Index in 2019

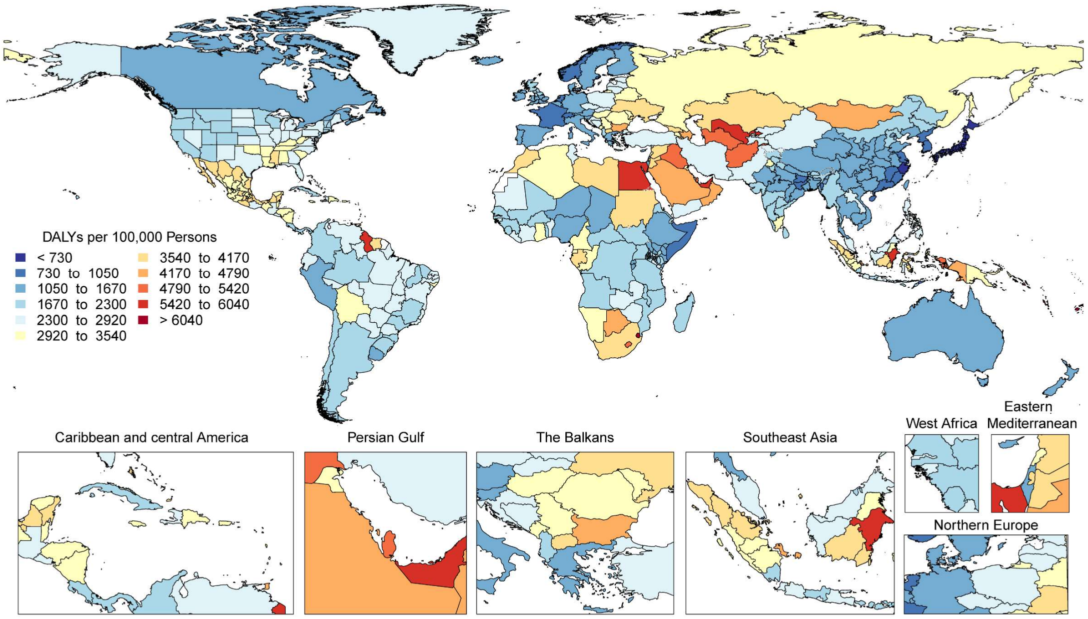

Map of age-standardized disability-adjusted life years (DALYs) rate of high body-mass index, 2019.

Supplementary Figure 48. Total Numbers and Rates of Impaired Kidney Function

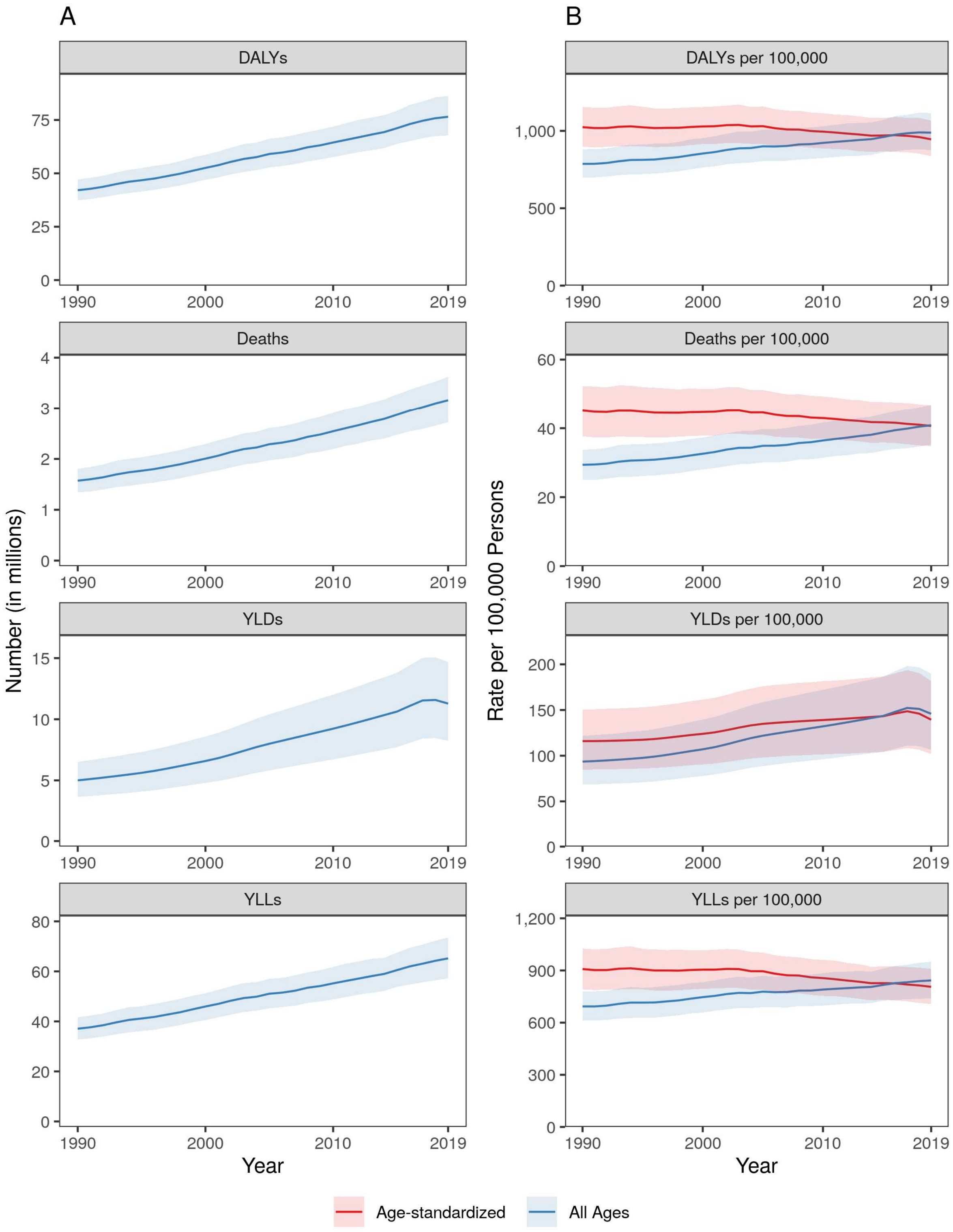

A. Total number of disability-adjusted life years (DALYs), deaths, years lived with disability (YLDs), and years of life lost (YLLs) due to impaired kidney function, 1990 to 2019. Shaded

regions represent 95% uncertainty intervals. B. Age-standardized and all ages disability-adjusted life years (DALYs), death, years lived with disability (YLDs), and years of life lost (YLLs) rates of impaired kidney function, 1990 to 2019. Shaded regions represent 95% uncertainty intervals.

Supplementary Figure 49. Total Numbers and Rates of Chronic Kidney Disease

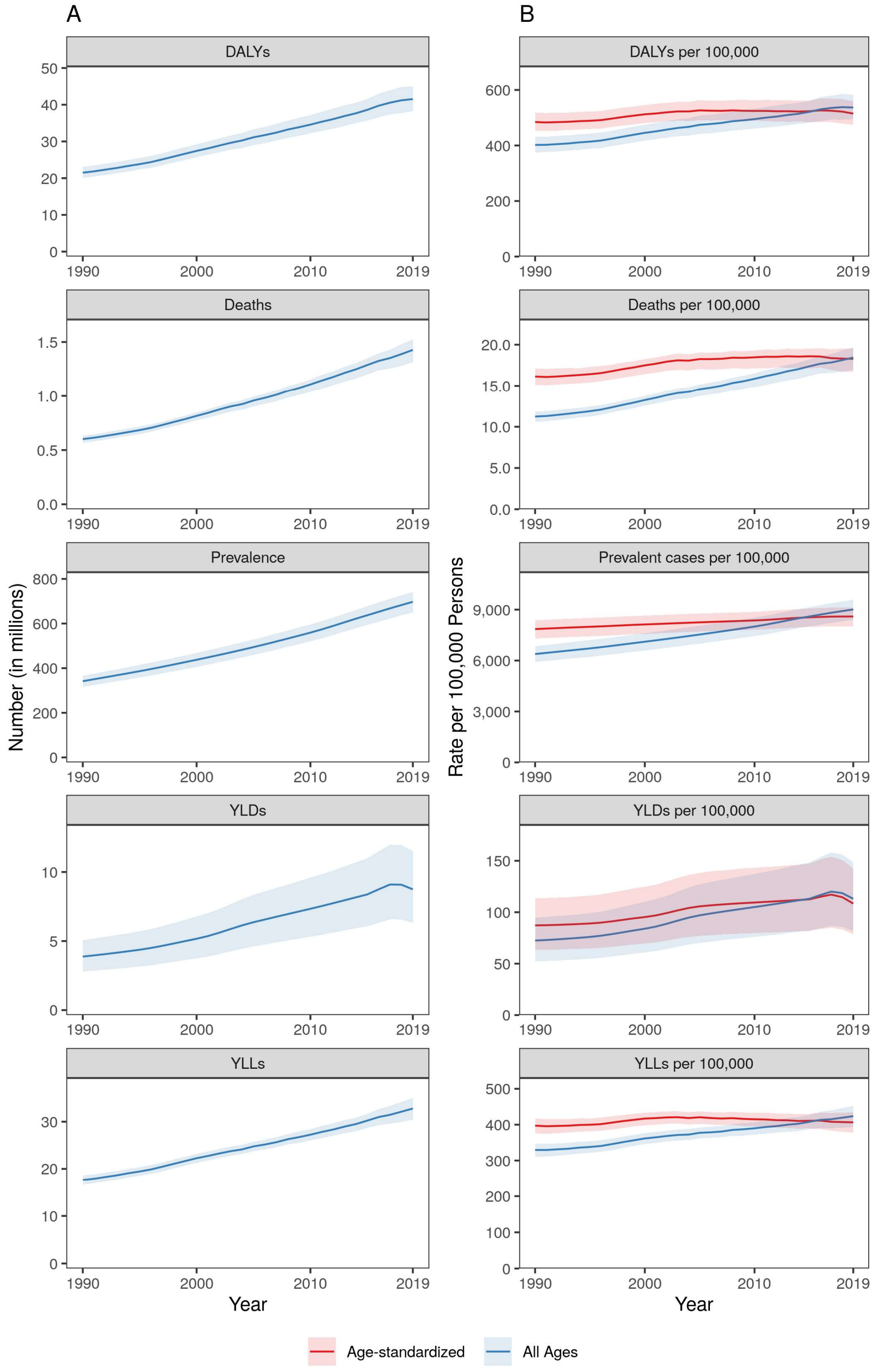

A. Total number of disability-adjusted life years (DALYs), deaths, prevalent cases, years lived with disability (YLDs), and years of life lost (YLLs) due to chronic kidney disease, 1990 to

2019. Shaded regions represent 95% uncertainty intervals. B. Age-standardized and all ages disability-adjusted life years (DALYs), death, prevalence, years lived with disability (YLDs), and years of life lost (YLLs) rates of chronic kidney disease, 1990 to 2019. Shaded regions represent 95% uncertainty intervals.

Supplementary Figure 50. DALYs due to Chronic Kidney Disease in 2019 by Age

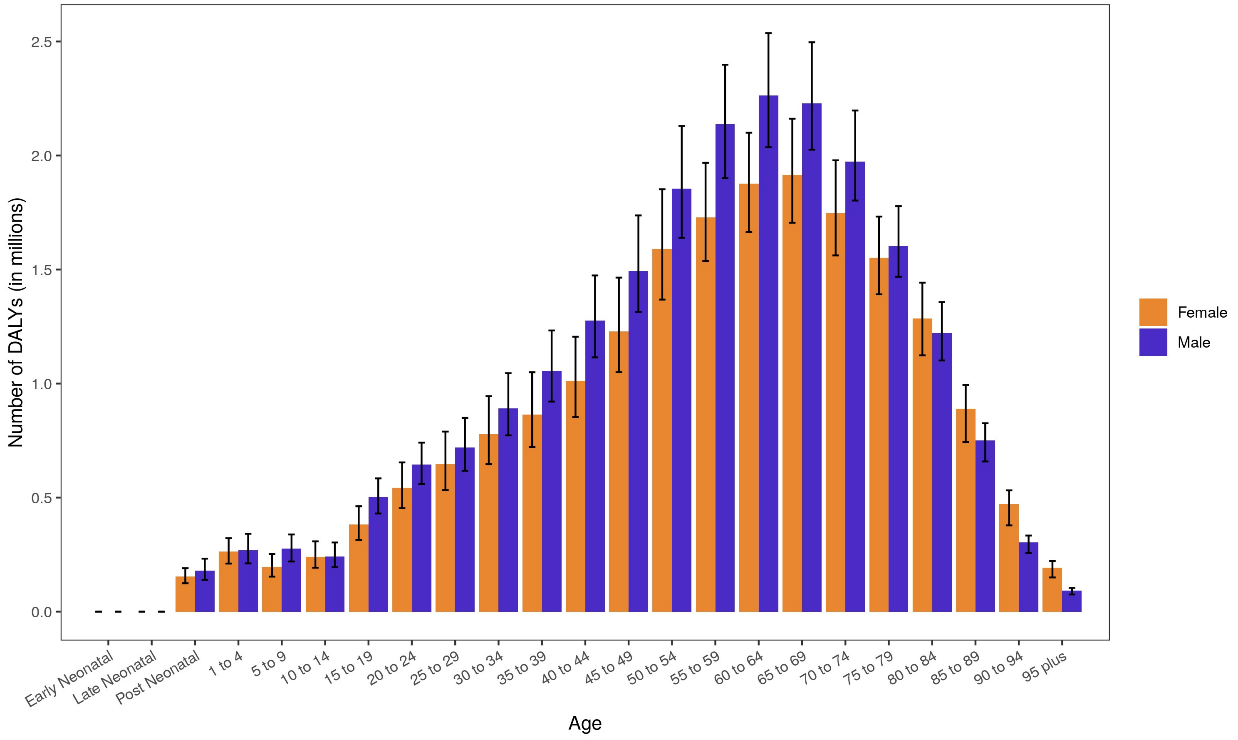

Number of disability-adjusted life years (DALYs) due chronic kidney disease by age and sex with 95% uncertainty intervals, 2019.

Supplementary Figure 51. DALYs due to Impaired Kidney Function in 2019 by Age

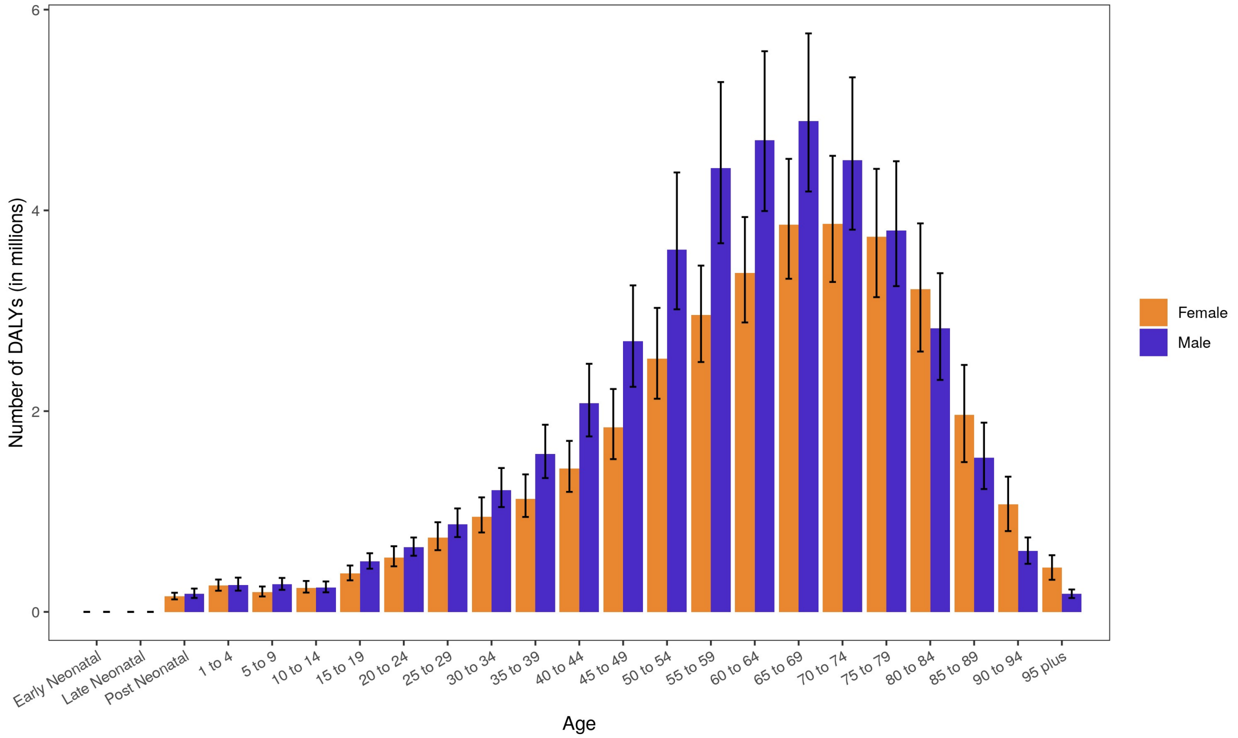

Number of disability-adjusted life years (DALYs) due impaired kidney function by age and sex with 95% uncertainty intervals, 2019.

Supplementary Figure 52. Age-Standardized DALYs due to Impaired Kidney Function in 2019 by Region

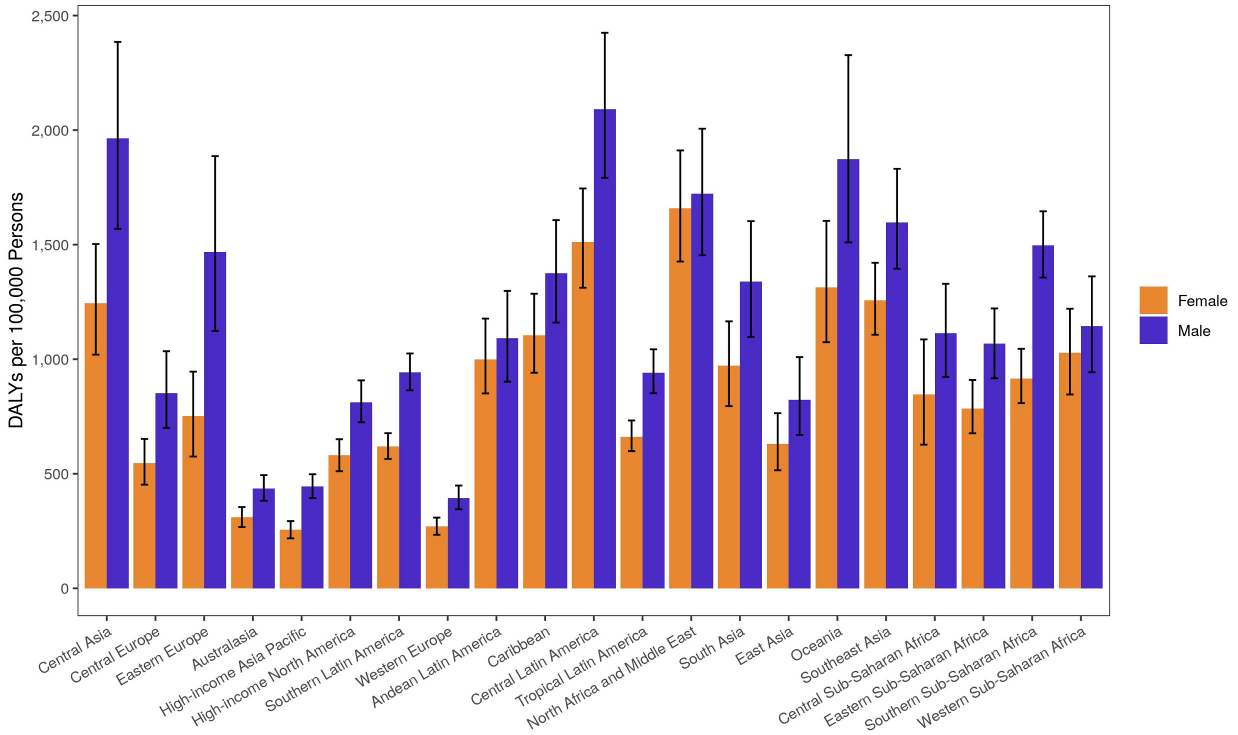

Age-standardized disability-adjusted life years (DALYs) rate of impaired kidney function by region and sex with 95% uncertainty intervals, 2019.

Supplementary Figure 53. Total Numbers and Rates of Ambient Particulate Matter Pollution

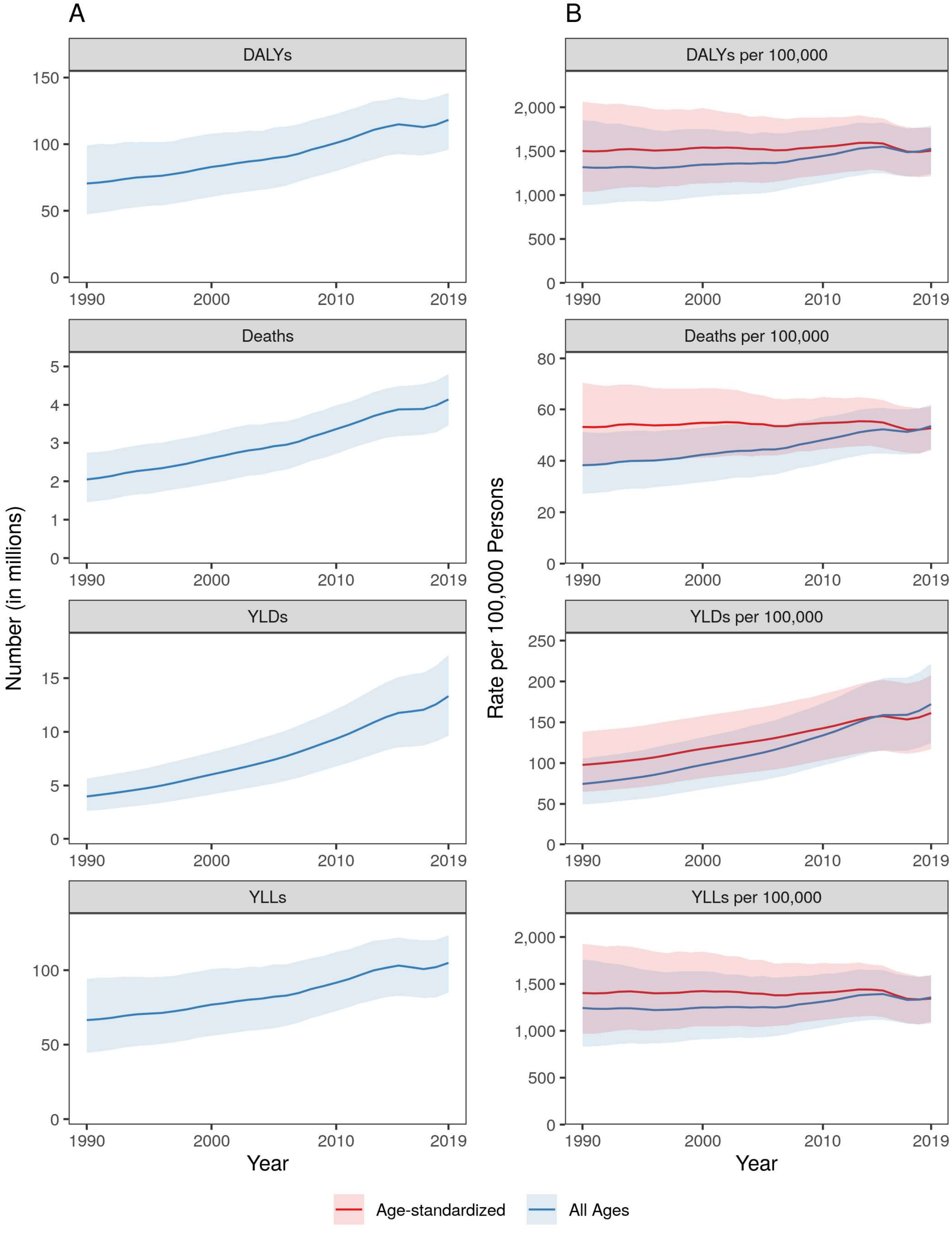

A. Total number of disability-adjusted life years (DALYs), deaths, years lived with disability (YLDs), and years of life lost (YLLs) due to ambient particulate matter pollution, 1990 to 2019.

Shaded regions represent 95% uncertainty intervals. B. Age-standardized and all ages disability-adjusted life years (DALYs), death, years lived with disability (YLDs), and years of life lost (YLLs) rates of ambient particulate matter pollution, 1990 to 2019. Shaded regions represent 95% uncertainty intervals.

Supplementary Figure 54. Total Numbers and Rates of Household Air Pollution From Solid Fuels

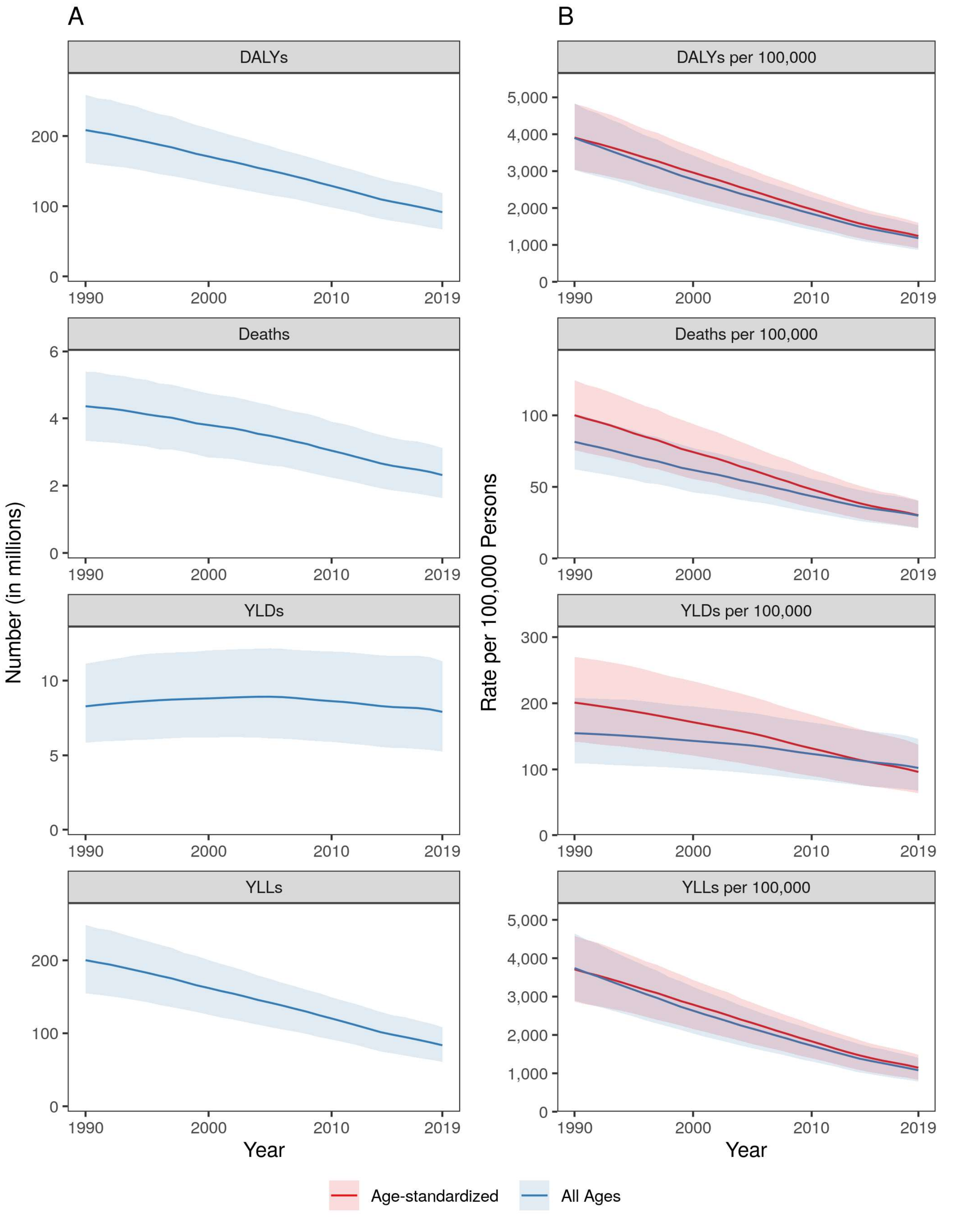

A. Total number of disability-adjusted life years (DALYs), deaths, years lived with disability (YLDs), and years of life lost (YLLs) due to household air pollution from solid fuels, 1990 to

2019. Shaded regions represent 95% uncertainty intervals. B. Age-standardized and all ages disability-adjusted life years (DALYs), death, years lived with disability (YLDs), and years of life lost (YLLs) rates of household air pollution from solid fuels, 1990 to 2019. Shaded regions represent 95% uncertainty intervals.

Supplementary Figure 55. Map of Age-Standardized DALYs due to Household Air Pollution From Solid Fuels in 2019

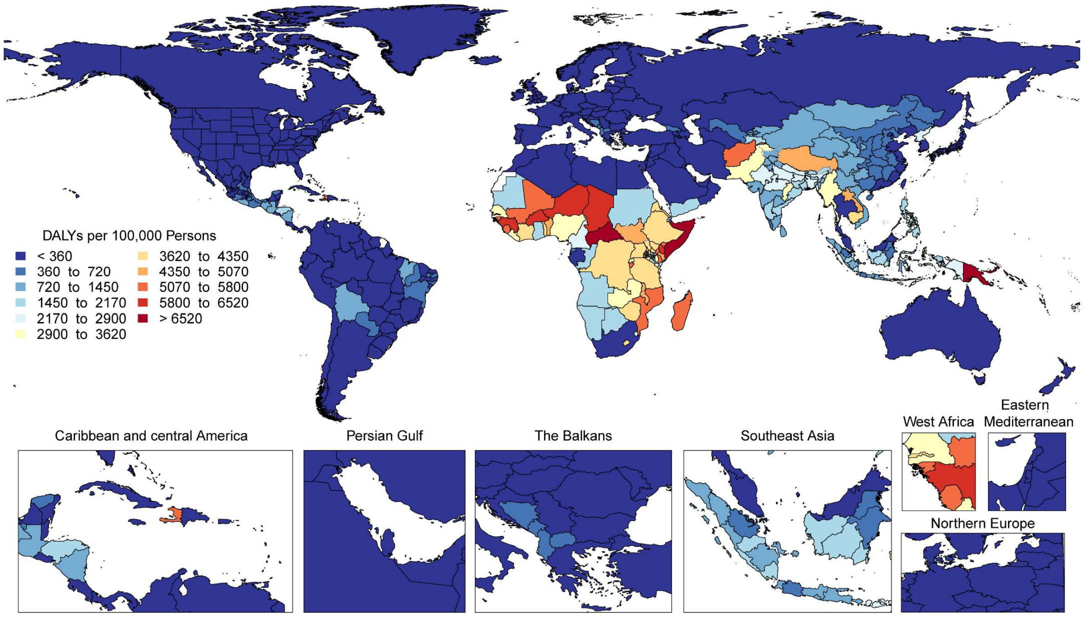

Map of age-standardized disability-adjusted life years (DALYs) rate of household air pollution from solid fuels, 2019.

Supplementary Figure 56. DALYs due to Ambient Particulate Matter Pollution in 2019 by Age

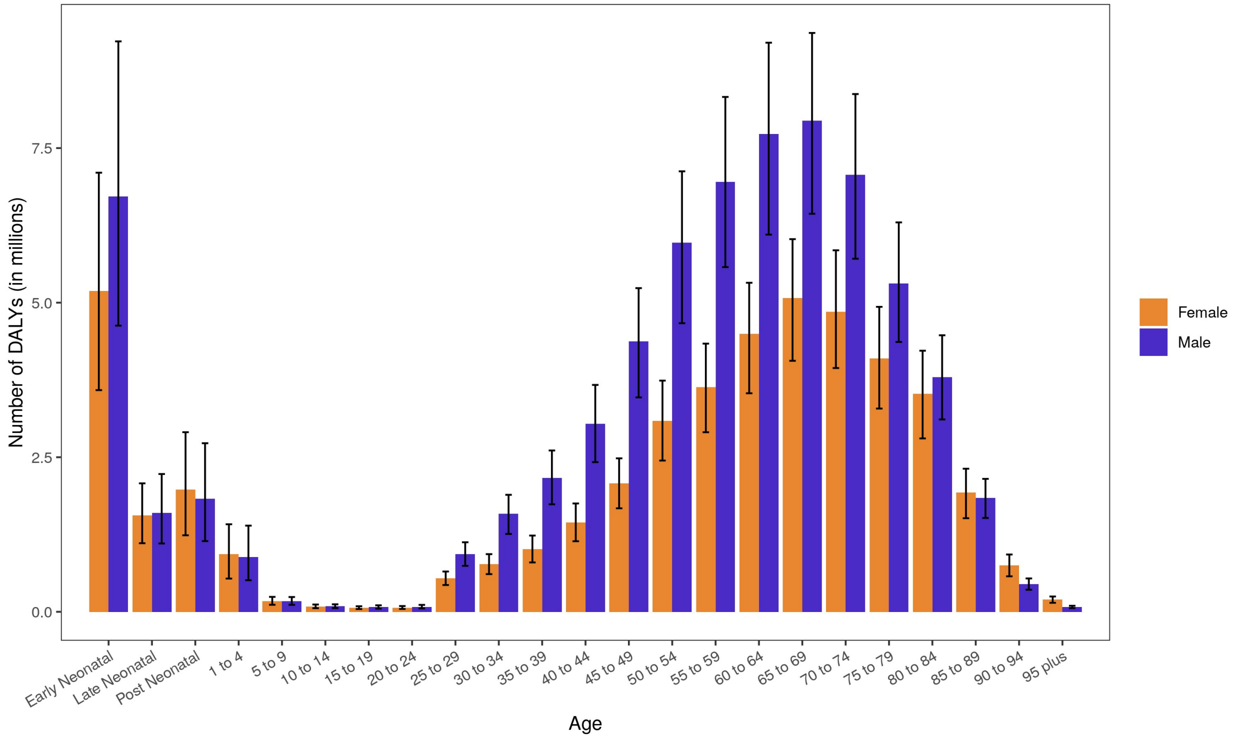

Number of disability-adjusted life years (DALYs) due to ambient particulate matter pollution by age and sex with 95% uncertainty intervals, 2019.

Supplementary Figure 57. DALYs due to Household Air Pollution From Solid Fuels in 2019 by Age

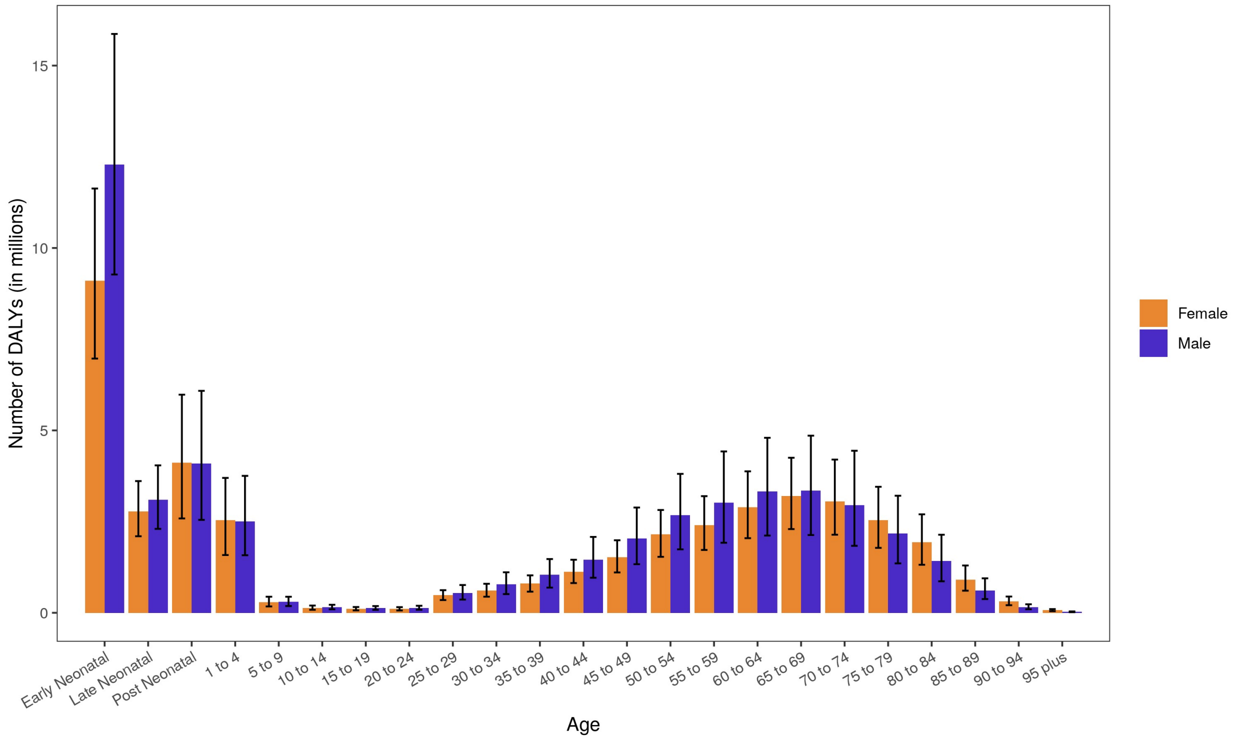

Number of disability-adjusted life years (DALYs) due to household air pollution from solid fuels by age and sex with 95% uncertainty intervals, 2019.

Supplementary Figure 58. DALYs due to Tobacco in 2019 by Age

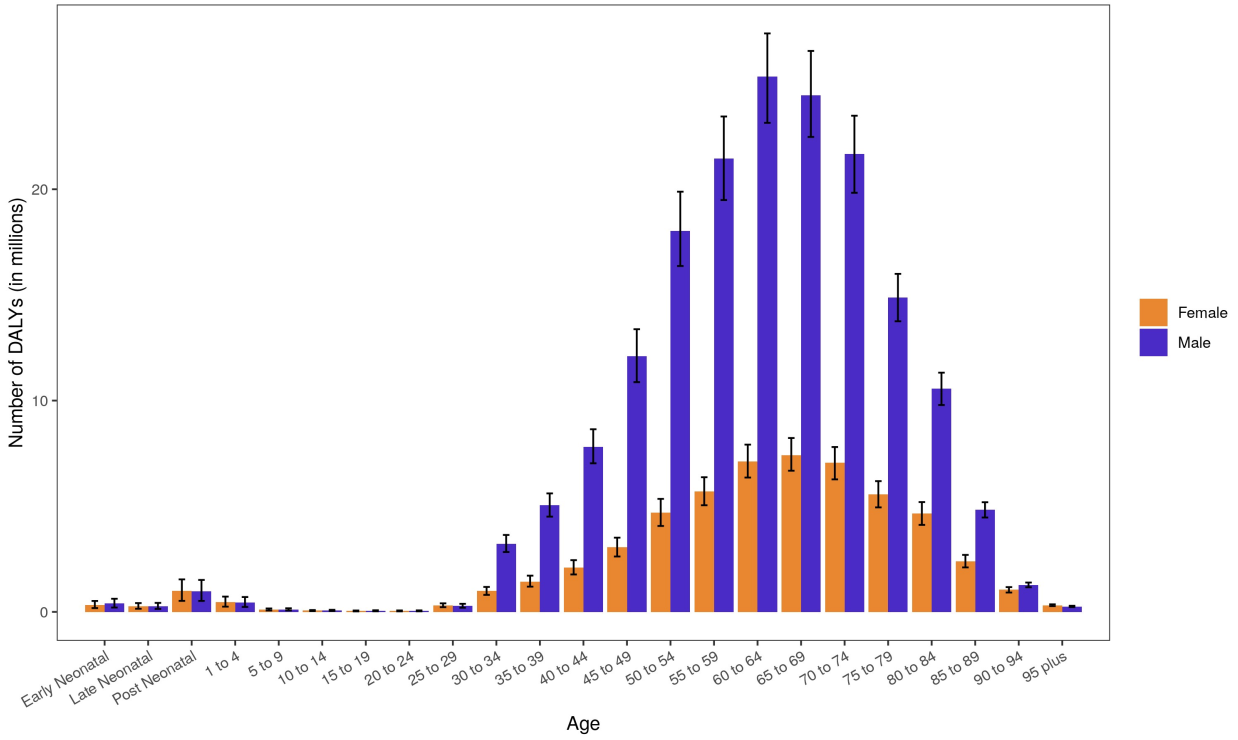

Number of disability-adjusted life years (DALYs) due to tobacco by age and sex with 95% uncertainty intervals, 2019.

Supplementary Figure 59. Age-Standardized DALYs due to Tobacco in 2019 by Region

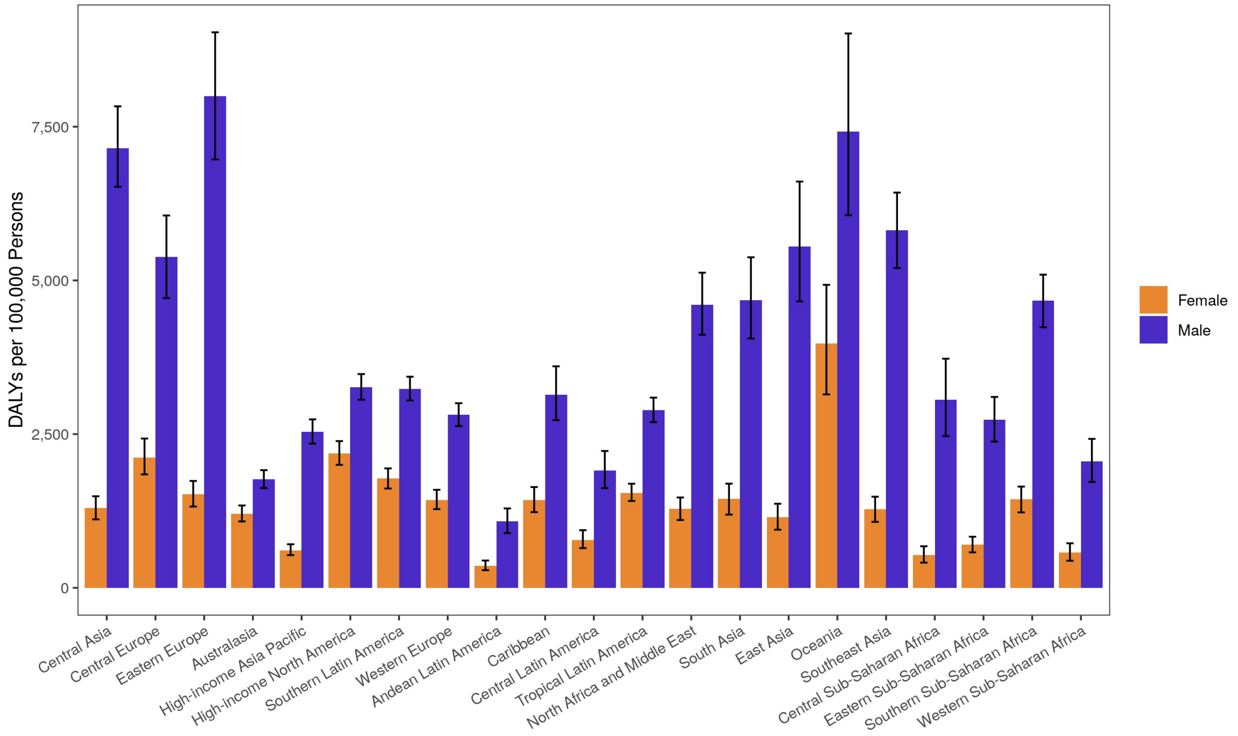

Age-standardized disability-adjusted life years (DALYs) rate of tobacco by region and sex with 95% uncertainty intervals, 2019.

Supplementary Figure 60. DALYs due to Dietary Risks in 2019 by Age

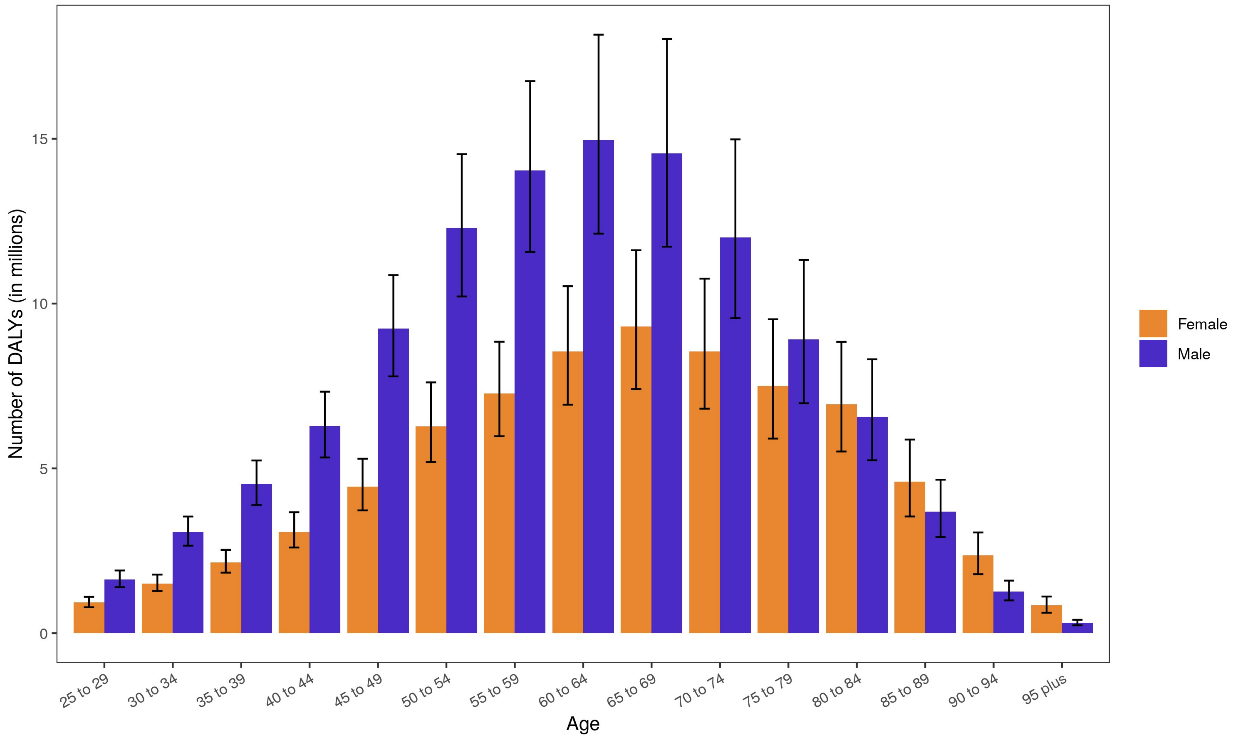

Number of disability-adjusted life years (DALYs) due to dietary risks by age and sex with 95% uncertainty intervals, 2019. Ages below 25 were removed from this figure as they are not modeled for this risk.

Supplementary Figure 61. Age-Standardized DALYs due to Dietary Risks in 2019 by Region

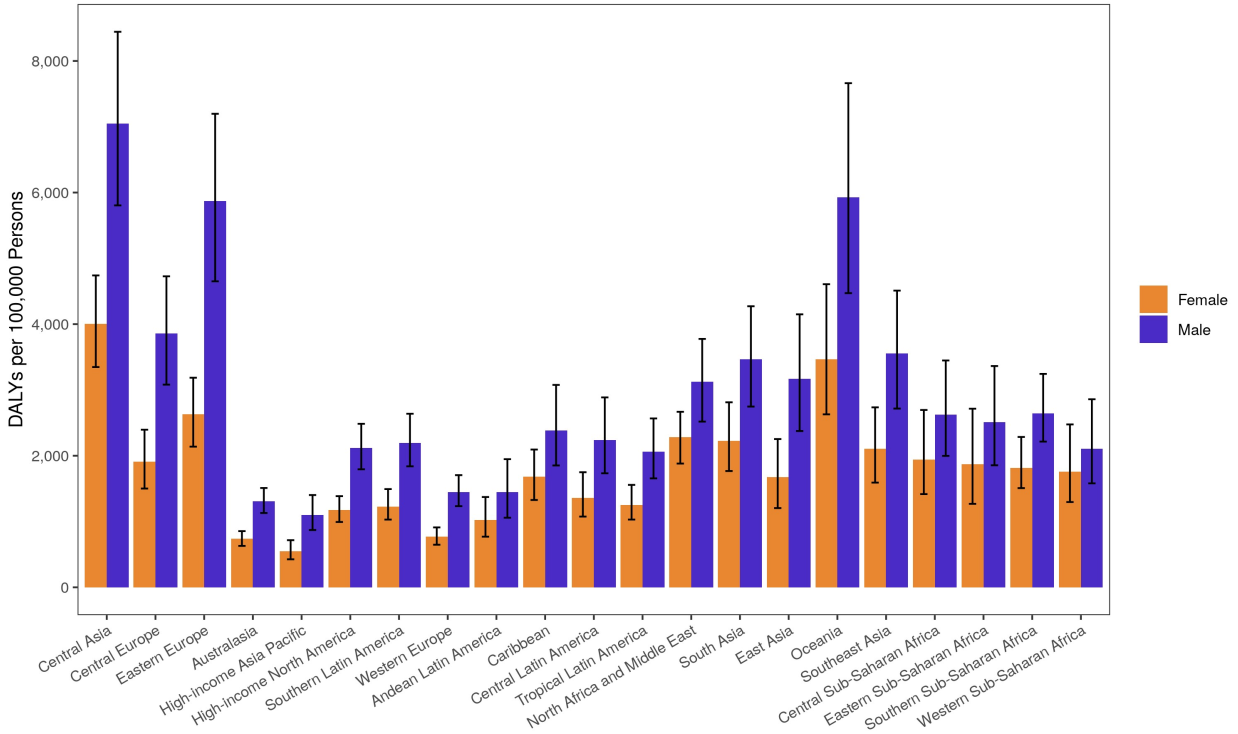

Age-standardized disability-adjusted life years (DALYs) rate of dietary risks by region and sex with 95% uncertainty intervals, 2019.

Supplementary Figure 62. Map of Age-Standardized DALYs due to Dietary Risks in 2019

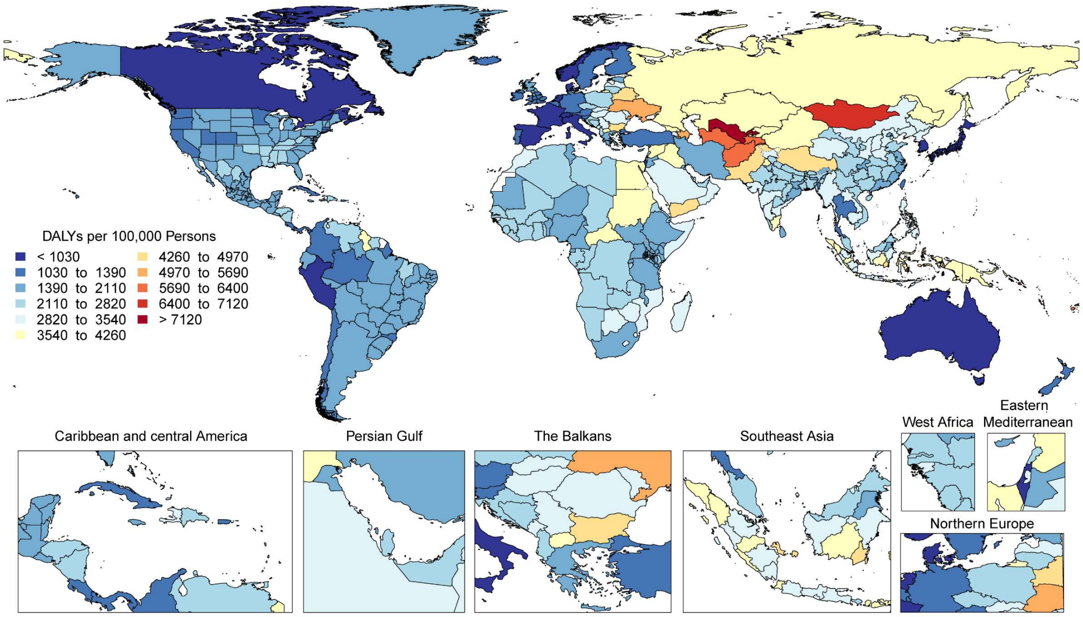

Map of age-standardized disability-adjusted life years (DALYs) rate of dietary risks, 2019.

Supplementary Figure 63. Total Numbers and Rates of Low Physical Activity

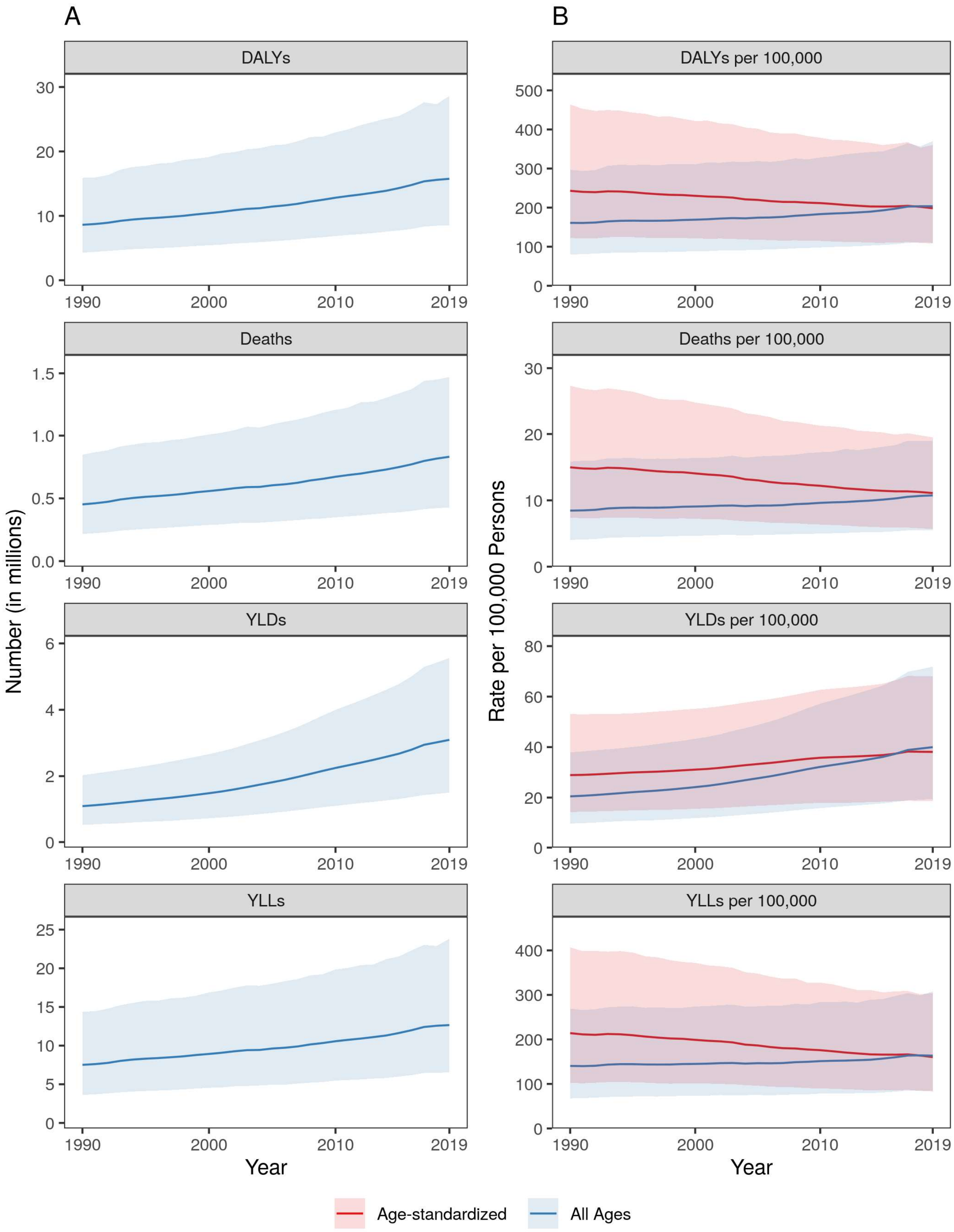

A. Total number of disability-adjusted life years (DALYs), deaths, years lived with disability (YLDs), and years of life lost (YLLs) due to low physical activity, 1990 to 2019. Shaded regions

represent 95% uncertainty intervals. B. Age-standardized and all ages disability-adjusted life years (DALYs), death, years lived with disability (YLDs), and years of life lost (YLLs) rates of low physical activity, 1990 to 2019. Shaded regions represent 95% uncertainty intervals.

Supplementary Figure 64. DALYs due to Low Physical Activity in 2019 by Age

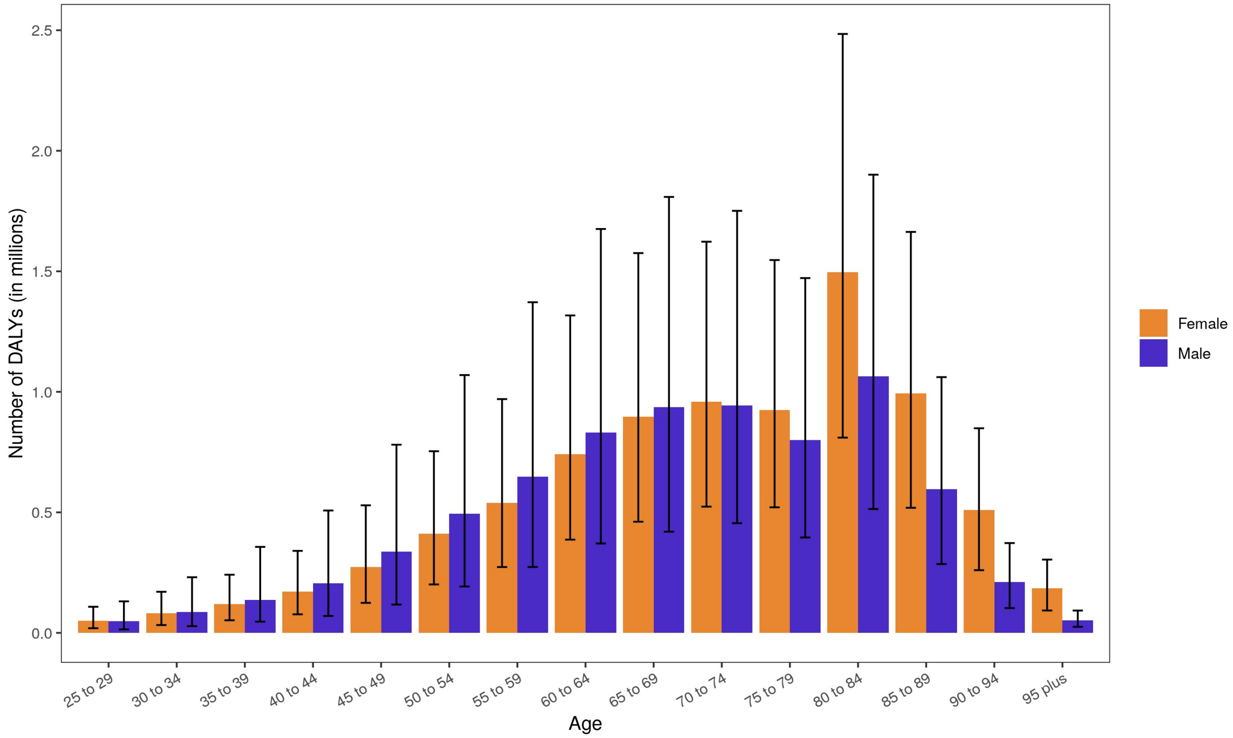

Number of disability-adjusted life years (DALYs) due to low physical activity by age and sex with 95% uncertainty intervals, 2019. Ages below 25 were removed from the figure as they are not modeled for this risk.

Supplementary Figure 65. Age-Standardized DALYs due to Low Physical Activity in 2019 by Region

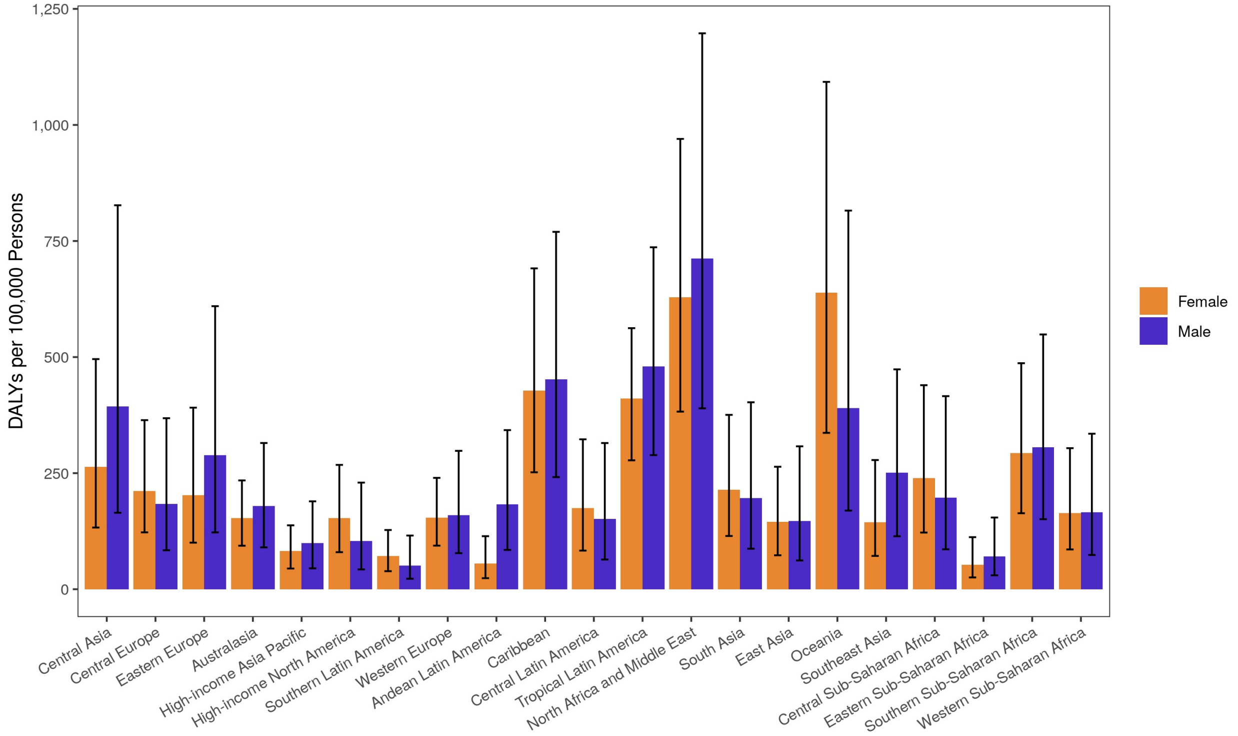

Age-standardized disability-adjusted life years (DALYs) rate of low physical activity by region and sex with 95% uncertainty intervals, 2019.

Supplementary Table 1. Prevalence of High SBP in 1990 and 2019

Year	Age	Prevalence > 110-115 mmHg		Prevalence > 130 mmHg		Prevalence > 140 mmHg	
		Rate per 100,000 (95% UI)	Number of individuals, thousands (95% UI)	Rate per 100,000 (95% UI)	Number of individuals, thousands (95% UI)	Rate per 100,000 (95% UI)	Number of individuals, thousands (95% UI)
**1990**	**All Ages**	84,481 (81,641 to 87,579)	2,182,653 (2,109,290 to 2,262,697)	32,678 (30,972 to 34,487)	844,274 (800,181 to 890,997)	15,057 (14,158 to 16,024)	389,006 (365,795 to 413,992)
**2019**		88,971 (86,950 to 91,029)	4,055,344 (3,963,223 to 4,149,135)	39,187 (37,127 to 41,259)	1,786,158 (1,692,277 to 1,880,619)	18,172 (16,855 to 19,480)	828,289 (768,279 to 887,920)
**1990**	**Age-standardized**	85,810 (83,309 to 88,498)	---	36,075 (34,430 to 37,878)	---	17,322 (16,323 to 18,361)	---
**2019**		88,879 (86,857 to 90,960)	---	38,959 (36,933 to 40,991)	---	18,031 (16,756 to 19,288)	---

Using the GBD 2019 distribution of systolic blood pressure (SBP) at the global level in 1990 and 2019, the prevalence of SBP above 110-115 mmHg, 130 mmHg, and 140 mmHg was estimated by calculating the area under the curve above each level. SBP exposure was modeled for ages 25 and above.
