# Supplementary material for: Global Burden of Cardiovascular Diseases and Risk Factors, 1990–2019: Update From the GBD 2019 Study
Source: J Am Coll Cardiol. 2020 Dec 22;76(25):2982–3021. doi: 10.1016/j.jacc.2020.11.010 (PMC7755038; doi:10.1016/j.jacc.2020.11.010)
Supplement: Supplemental Data 3 [file mmc3.docx]

Declarations

Dr. Valgimigli reports grants and personal fees from Terumo; personal fees from Astra Zeneca, Alvimedica/CID, Abbott Vascular, Daiichi Sankyo, Opsens, Bayer, CoreFLOW, IDORSIA PHARMACEUTICALS LTD, Universität Basel | Dept. Klinische Forschung, Vifor, Bristol Myers Squib SA, iVascular, Medscape, and Biotronik, outside the submitted work. V. Aboyans reports personal fees from AstraZeneca, Bayer Healthcare, Boehringer Ingelheim, and BMS/Pfizer Alliance, outside the submitted work. Dr. Ancuceanu reports consultancy/ speaker fees from UCB, Sandoz, Abbvie, Zentiva, Teva, Laropharm, CEGEDIM, Angelini, Blessen Pharma, Hofigal, AstraZeneca and Stada. Dr. Antony acknowledges supported from the National Health and Medical Research Council of Australia (NHMRC) Fellowship. A. Baig acknowledges grant FRGS/1 /2017/SKK06iUNISZA/02/1 under Kementerian Pendidikan Malaysia (KPM) and Universiti Sultan Zainal Abidin, in which they are working on vaccine development but after being part of this study, will focus also into meta-analysis of CVD and S. Pneumoniae infection in Malaysia. Dr. Ärnlöv reports personal fees from Astra Zeneca, Boeringer Ingelheim, and Novartis, outside the submitted work. Dr. Béjot reports personal fees from Amgen, AstraZeneca, BMS, Pfizer, Medtronic, MSD; grants and personal fees from Boehringer Ingelheim; personal fees and non-financial support from Servier; and non-financial support from Biogen, outside the submitted work. Dr. Carrero acknowledges support by the Swedish Research Council (grant number 2019-01059) and the Swedish Heart and Lung Foundation. Dr. Carvalho acknowledges UID/MULTI/04378/2019 and UID/QUI/50006/2019 support with funding from FCT/MCTES. Dr. Costa acknowledges grant (SFRH/BHD/110001/2015), received by Portuguese national funds through Fundação para a Ciência e Tecnologia (FCT), IP, under the Norma Transitória DL57/2016/CP1334/CT0006. Dr. De Neve acknowledges support by the Alexander von Humboldt Foundation. A. Douiri acknowledges funding support from the National Institute for Health Research (NIHR) Applied Research Collaboration (ARC) South London at King’s College Hospital NHS Foundation Trust and the Royal College of Physicians, as well as the support from the NIHR Biomedical Research Centre based at Guy’s and St Thomas’ NHS Foundation Trust and King’s College London. Dr. Deuschl reports personal fees from Boston Scientific, Cavion, Functional Neuromodulation, and grants from Medtronic, outside the submitted work. Dr. Duncan and Dr. Schmidt supported in part by the Brazilian National Council for Scientific and Technological Development (CNPq, research fellowship) and the Institute for Health Technology Assessment (IATS; 465518/2014-1). Drs. Ausloos, Pana and Herteliu are partially supported by a grant of the Romanian National Authority for Scientific Research and Innovation, CNDS-UEFISCDI, project number PN-III-P4-ID-PCCF-2016-0084. Drs. Herteliu and Pana are partially supported by a grant of the Romanian National Authority for Scientific Research and Innovation, CNDS-UEFISCDI, project number PN-III-P2-2.1-SOL-2020-2-0351. Dr. Hankey reports personal fees from the American Heart Association, outside the submitted work; and Honoraria from Bayer for lecturing at sponsored scientific symposia about stroke prevention in atrial fibrillation. Dr. Iqbal acknowledges support by the Ministry of Science and Technology (MOST), Taiwan under grant MOST109-2221-E-038-015. Dr. Islam reports grants from NHMRC and grants from National Heart Foundation of Australia, outside the submitted work. Dr. Jeemon reports relationships with ÝBT-Wellcome Trust-India Alliance Clinical and Public Health Intermediate Fellowship (IA/CPHI/14/1/501497). Dr. Jonnagaddala reports grants from National Health and Medical Research Council (NHMRC), outside the submitted work. Dr. Jozwiak reports personal fees from Amgen, ALAB Laboratories, Teva, Synexus, Boehringer Ingelheim, and Zentiva, outside the submitted work. Dr. Katoto acknowledges support by the US National Institutes of Health (NIH)-Fogarty Postdoctoral Fellowship: Grant No. 1D43TW010937-01A1. Dr. Kim acknowledges support by the Research Management Centre, Xiamen University Malaysia [No.: XMUMRF/2020-C6/ITCM/0004]. Dr. Kivimaki reports grants from Medical Research Council (MR/R024227/1), during the conduct of the study. Dr. Krishan reports non-financial support from UGC Centre of Advanced Study, CAS II, awarded to the Department of Anthropology, Panjab University, Chandigarh, India, outside the submitted work. Dr. Kwan reports grants from NHLBI during the conduct of the study. Dr. Lacey acknowledges support from the NIHR Oxford Biomedical Research Centre and the BHF Centre of Research Excellence, Oxford. Dr. Lallukka acknowledges support by the Academy of Finland (Grant #330527). Dr. Li acknowledges support by research funding from China Medical University (TW). Dr. Lim reports grants from Boehringer Ingelheim, outside the submitted work. Dr. Lorkowski reports personal fees from Akcea Therapeutics, amedes, Amgen, Berlin-Chemie, Boehringer Ingelheim Pharma, Daiichi Sankyo, Lilly, MSD Sharp & Dohme, Novo Nordisk, Sanofi-Aventis, Synlab, Unilever, and Upfield; and non-financial support from Preventicus outside the submitted work. Dr. Lorkowski acknowledges additional support from the Competence Cluster for Nutrition and Cardiovascular Health (nutriCARD) Halle-Jena-Leipzig (Germany; German Federal Ministry of Education and Research; grant agreement number 01EA1808A). Dr. Mantovani reports grants and non-financial support from Italian Ministry of Health Ricerca Corrente - IRCCS MultiMedica, during the conduct of the study. Dr. Nazarzadeh acknowledges support by the British Heart Foundation (grant number: FS/19/36/34346). Dr. Norrving reports personal fees from Astra Zeneca and personal fees from Bayer, outside the submitted work. Dr. Nouthe reports personal fees (honorarium) from BMS Pfizer. Dr. Odukoya acknowledges support by the Fogarty International Center of the National Institutes of Health under Award Number K43TW010704. The content is solely the responsibility of the authors and does not necessarily represent the official views of the National Institutes of Health. Dr. Pilgrim reports grants and personal fees from Biotronik, grants and personal fees from Boston Scientific, personal fees from HighLife SAS, and non-financial support from Medtronic, outside the submitted work. Dr. Rakovac reports grants from the Russian Federation in the context of the WHO European Office for the Prevention and Control of Noncommunicable Diseases. WHO is funded by Governments of all Member States, during the conduct of the study; and the authors alone are responsible for the views expressed in this article and they do not necessarily represent the views, decisions or policies of the institutions with which they are affiliated. Dr. Remuzzi reports personal fees and non-financial support from Alnylam, Boeringher Ingelheim, Handok Inc, Incenption Sciences Canada, Alexion Pharmaceuticals Inc, and Janssen Pharmaceutical, outside the submitted work. Dr. Sacco reports personal fees and non-financial support from Allergan, Novartis, Abbott, Teva, and Lilly, outside the submitted work. Dr. Samy acknowledges support from the Egyptian Fulbright Mission Program. Dr. Santric-Milicevic acknowledges supporty by the Ministry of Education, Science and Technological Development of the Republic of Serbia (Contract No. 175087). Dr. Saylan is an employee of Bayer AG. Dr. Schutte reports personal fees from Servier, Takeda, Novartis, and Omron, outside the submitted work. Dr. Sha acknowledges support by the Shenzhen Science and Technology Program (Grant No. KQTD20190929172835662). Dr. Sheikh acknowledges support from the Health Data Research UK BREATHE Hub. Dr. Singh reports personal fees from Crealta/Horizon, Medisys, Fidia, UBM LLC, Trio health, Medscape, WebMD, Clinical Care options, Clearview healthcare partners, Putnam associates, Focus forward, Navigant consulting, Simply Speaking, Spherix, Practice Point communications, the National Institutes of Health and the American College of Rheumatology; ownership in stock options from Amarin, Viking, Moderna and Vaxart pharmaceuticals, and Charlotte’s Web Holdings; non-financial support from FDA Arthritis Advisory Committee; non-financial support from Steering committee of OMERACT, an international organization that develops measures for clinical trials and receives arm’s length funding from 12 pharmaceutical companies; non-financial support from Veterans Affairs Rheumatology Field Advisory Committee; and non-financial support from Editor and the Director of the UAB Cochrane Musculoskeletal Group Satellite Center on Network Meta-analysis, outside the submitted work. Dr. Stortecky reports grants from Edwards Lifesciences, Medtronic, Boston Scientific; and personal fees from Boston Scientific, Teleflex, and BTG, outside the submitted work. Dr. Thrift reports grants from National Health & Medical Research Council (Australia), grants from Heart Foundation (Australia), and grants from Stroke Foundation, outside the submitted work. Dr. Tonelli acknowledges support from the David Freeze Research Chair (University of Calgary). Dr. Truelsen acknowledges the Research Fund of Rigshospitalet, Copenhagen University Hospital (grant E-23677). Dr. Tsilimparis reports grants and personal fees from Cook Medical, outside the submitted work. Dr. Tyrovolas acknowledges support by the Foundation for Education and European Culture, the Miguel Servet Programme (reference CP18/00006) and the Fondos Europeos de Desarrollo Regional. Dr. Vaduganathan reports grants from Harvard Catalyst; personal fees from Bayer AG, Baxter Healthcare, AstraZeneca, Cytokinetics, and Relypsa; grants and personal fees from Amgen and Boehringer Ingelheim, outside the submitted work. Dr. Yan acknowledges support by the National Natural Sciences Foundation of China grant no. 71774075. Dr. Yilgwan acknowledges support as a recipient of the STAMINA Mentored research seed grant. Dr. Zaman acknowledges support by scholarship from the Australian Government research-training program (RTP) in support of his academic career. Dr. Zhang acknowledges support by Science and Technology Research Project of Hubei Provincial Department of Education (Grant No. Q20201104), and Outstanding Young and Middle-aged Technology Innovation Team Project of Hubei Provincial Department of Education (Grant No. T2020003).

Acknowledgments

Dr. Bennett acknowledges support by the UK National Institute of Health Research (NIHR) Oxford Biomedical Research Centre. The views expressed are those of the authors and not necessarily those of the NIHR or the Department of Health and Social Care. Dr. Jeemon acknowledges Wellcome Trust/DBT India Alliance Clinical and Public Health Intermediate Fellowship [IA/CPHI/14/1/501497].
